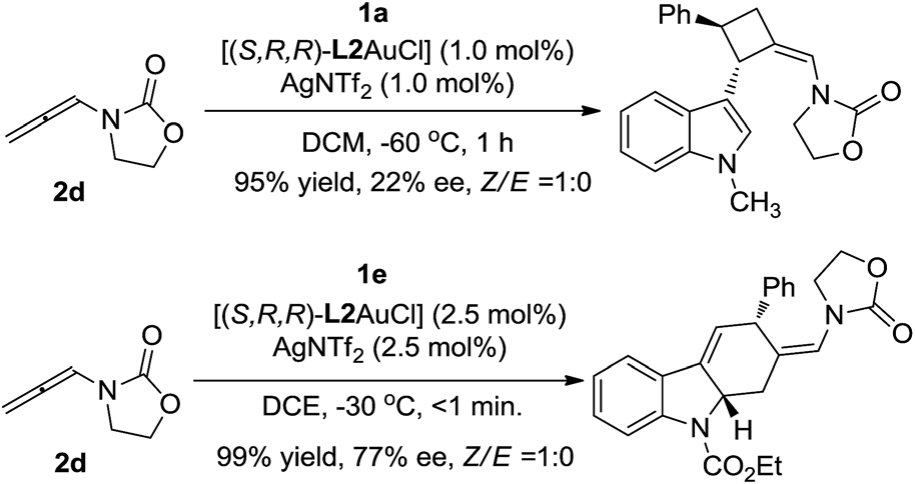# Enantioselective gold-catalyzed intermolecular [2+2] *versus* [4+2]-cycloadditions of 3-styrylindoles with *N*-allenamides: observation of interesting substituent effects†
†Electronic supplementary information (ESI) available: Data for the new compounds, experimental procedures and theoretical studies on the mechanisms. CCDC 1036866 and 1036867. For ESI and crystallographic data in CIF or other electronic format see DOI: 10.1039/c5sc01827g


**DOI:** 10.1039/c5sc01827g

**Published:** 2015-06-23

**Authors:** Yidong Wang, Peichao Zhang, Yuan Liu, Fei Xia, Junliang Zhang

**Affiliations:** a Shanghai Key Laboratory of Green Chemistry and Chemical Processes , School of Chemistry and Molecular Engineering , East China Normal University , 3663 N. Zhongshan Road , Shanghai 200062 , China . Email: fxia@chem.ecnu.edu.cn ; Email: jlzhang@chem.ecnu.edu.cn; b State Key Laboratory of Organometallic Chemistry , Shanghai Institute of Organic Chemistry , CAS , 345 Lingling Road , Shanghai 200032 , China

## Abstract

A highly enantioselective [2+2] *versus* a [4+2]-cycloaddition of 3-styrylindoles to *N*-allenamides catalyzed by identical gold(i)/chiral phosphoramidite complexes is presented, which provides facile access to synthetically valuable, optically active substituted cyclobutanes and tetrahydrocarbazoles. The cycloaddition mode unexpectedly depends on the electronic nature of the *N*-substituent 3-styrylindoles, the origin of which could be well rationalized using DFT calculations and experimental results. To the best of our knowledge, the present work represents the first example of such an impressive substituent effect in tuning the reaction mode with high chemo-, regio- and enantioselectivity in asymmetric gold catalysis.

## Abstract 2

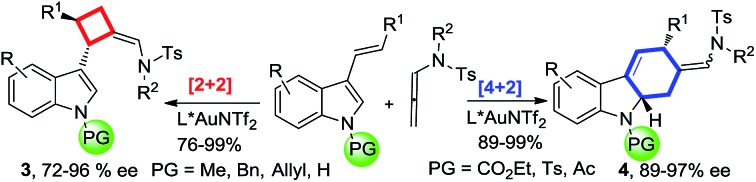
The cycloaddition mode ([2+2] *vs.* [4+2]) can be unexpectedly switched by the simple modification of the *N*-substituent of the 3-styrylindoles.

## Introduction

**Scheme 1:**
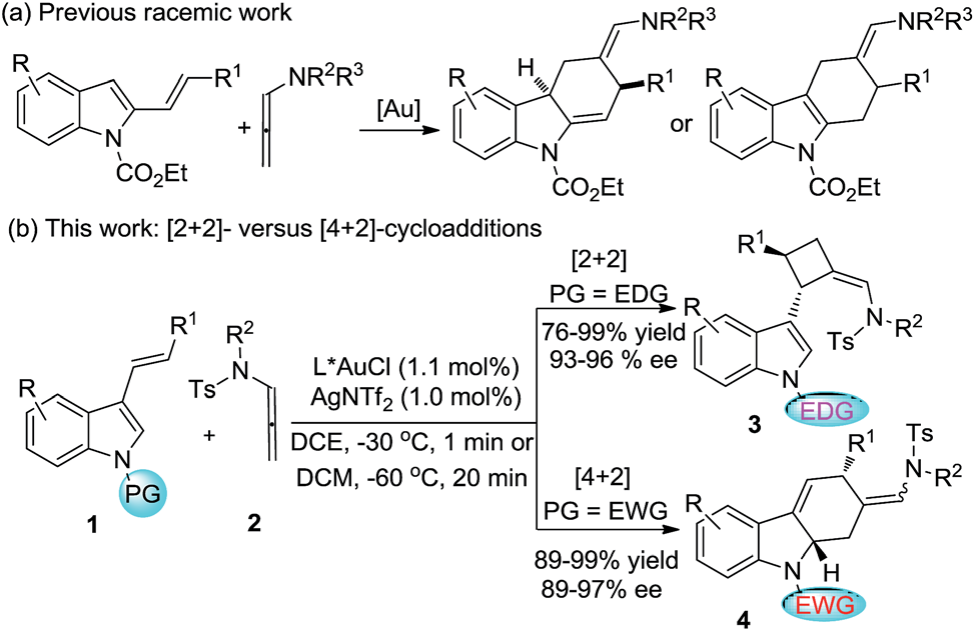
Previous work and this work.

**Fig. 1:**
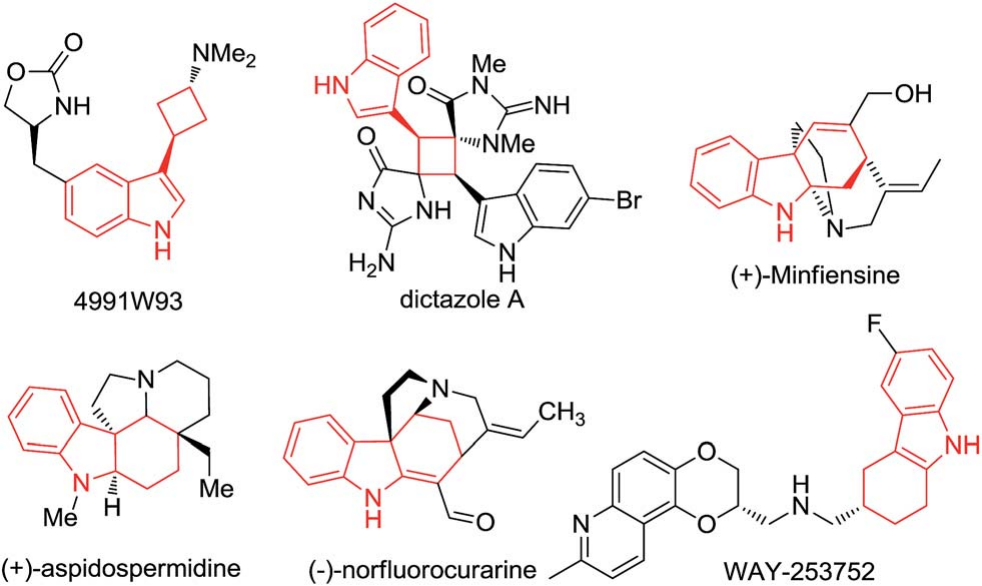
Natural products and bioactive compounds containing the tetrahydrocarbazole and 3-cyclobutylindole scaffolds.

Over the past decade, gold-catalyzed intermolecular cycloadditions of two unsaturated components have shown their power for rapid access to various cyclic and polycyclic ring systems in an extremely efficient and stereoselective manner.^1^ In this context, due to their unique reactivity and ease of accessibility, *N*-allenamides^2^ have emerged as unique partners for gold-catalyzed [2+2],^3^ [3+2],^4^ [4+2],^5^ and cascade cycloadditions,^6^ leading to synthetically useful four to seven membered carbocyclic or heterocyclic scaffolds. However, gold-catalyzed cycloadditions still pose challenges with respect to the cycloaddition mode as well as the enantioselectivity,^7^ only a few elegant examples of enantioselective cycloadditions involving *N*-allenamides have been reported to date. For example, González and co-workers3*e* achieved the first asymmetric [2+2]-cycloaddition with alkenes, leading to optically active cyclobutanes^8^ in good yields. Recently, Mascareñas and López5*c* explored the elegant asymmetric [4+2]-cycloaddition with 1,3-dienes, leading to cyclohexenes and a cascade cycloaddition with the alkene tethered ketone.^6^ An efficient asymmetric [3+2]-cycloaddition with nitrones was successfully realized by Chen.4*b* Very recently, Rossi, Vicente and their co-workers developed a seminal gold(i)-catalyzed intermolecular [4+2]-cycloaddition of 2-vinylindoles with *N*-allenamides, furnishing good yields of racemic tetrahydrocarbazoles (Scheme 1a).5*b* However, to the best of our knowledge, a cycloaddition mode which depends on the electronic nature of the substituent is unprecedented in asymmetric gold catalysis. As a part of our continual program in developing asymmetric gold-catalyzed cycloadditions,^9^ we report the gold catalyzed enantioselective [2+2] and [4+2] cycloadditions of 3-styrylindoles with *N*-allenamides (Scheme 1b), furnishing synthetically valuable tetrahydrocarbazole^10^,^11^ and 3-cyclobutylindole^12^,^13^ scaffolds, respectively, which frequently appear in natural products and bioactive compounds (Fig. 1).

## Results and discussion

**Scheme 2:**
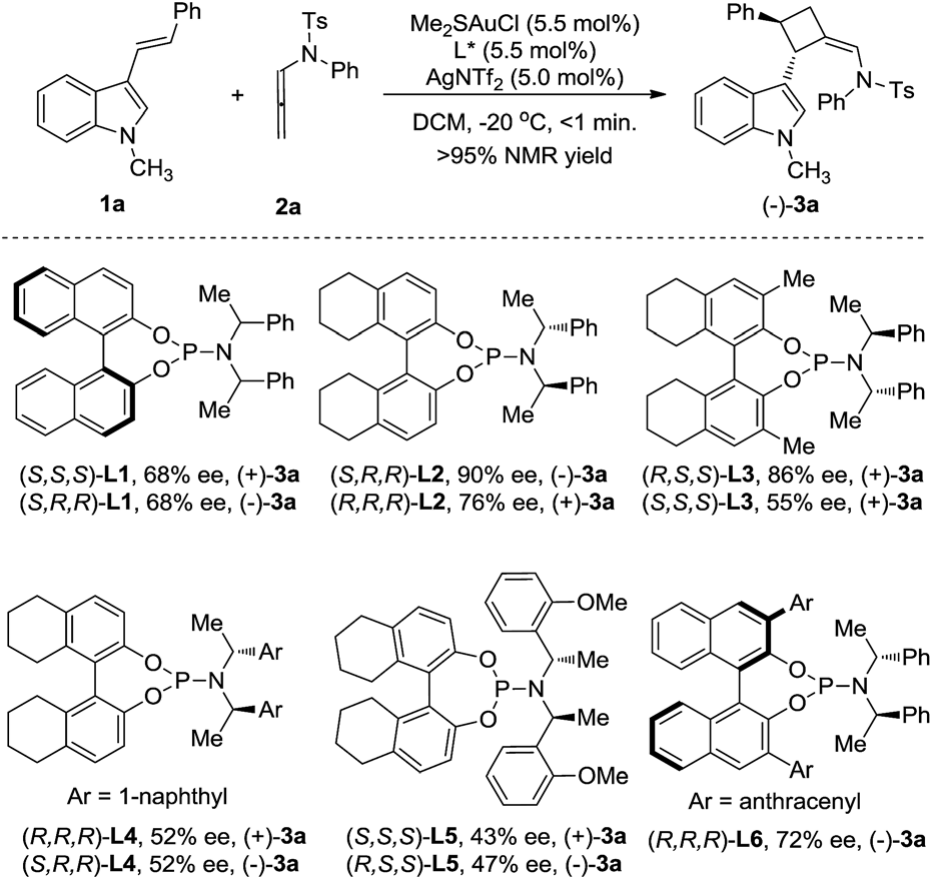
Investigation of the chiral ligands.

3-Styrylindole **1a** and *N*-allenamide **2a** were initially chosen as the model substrates to examine a series of chiral ligands (Scheme 2). It was surprising to find that the unexpected [2+2]-cycloadduct **3a**, rather than the [4+2]-cycloadduct, was always furnished in excellent yields (>95%) under all of the screened reaction conditions. Using BINOL-derived phosphoramidite ligand (*S*,*S*,*S*)-**L1** and its diastereomer (*S*,*R*,*R*)-**L1**, the reactions could give (+)-**3a** or its enantiomer (–)-**3a** with the same enantioselectivity (68% ee). Gratifyingly, the enantioselectivity could be significantly improved by the use of the H_8_-BINOL-derived phosphoramidite (*S*,*R*,*R*)-**L2**, furnishing (–)-**3a** in 90% ee. Notably, (+)-**3a** was obtained in 76% ee with the application of (*R*,*R*,*R*)-**L2** as the chiral ligand. Accordingly, the introduction of a methyl group to the 3- and 3′-positions of the BINOL moiety of **L2**, denoted as **L3**, could not increase the enantioselectivity. The changes of the *N*-substituents in **L4** or **L5** could not induce a high enantioselectivity either. The BINOL-derived ligand (*R*,*R*,*R*)-**L6** with 9-anthracenyl at the 3,3′-positions still could not improve the enantioselectivity. The further variation of other reaction parameters including the solvents, silver salts and concentration did not improve the result, but running the reaction at –60 °C led to the ee increasing to 96%.^14^ It should be noted that the reaction is easy to handle, and it works well in wet solvents under air without any detriment to the enantioselectivity or yield.

With the optimal reaction conditions in hand, a variety of 3-styrylindoles, **1**, with different *N*-protecting groups were prepared and investigated (Table 1). To our delight, those 3-styrylindoles with Bn (**1b**), allyl (**1c**) and free H (**1d**) groups could smoothly undergo the [2+2]-cycloaddition, furnishing the corresponding cycloadducts **3b–3d** in 72–92% ee as the single (*Z*)-stereoisomer (Table 1, entries 2–4). To our surprise, the [4+2]-cycloadducts **4a–4c** rather than the [2+2]-cycloadducts were obtained in 67–95% yields with up to 95% ee and 7.3 : 1 *Z*/*E*-selectivity with the use of an identical chiral gold catalyst under condition B (DCE, –30 °C, <1 min) when employing 3-styrylindoles **1e–1g** with electron-withdrawing *N*-protecting groups such as CO_2_Et, Ts and acyl (Table 1, entries 5–7). It should be noted that the [4+2]-cycloaddition takes place with a slightly lower *Z*/*E* selectivity but with almost the same enantioselectivity under condition A.

**Table 1:** The *N*-substituent effect of 3-styrylindoles^*a*^

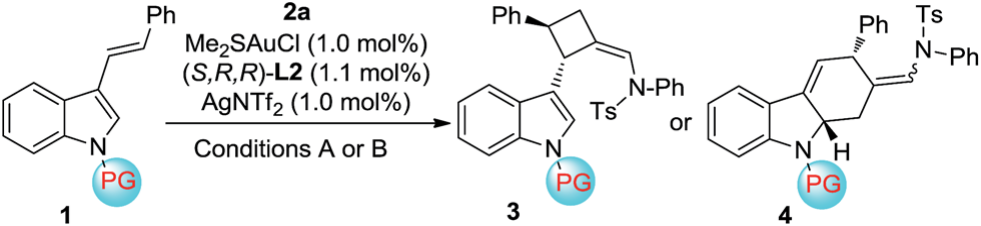						
Entry	PG	Conditions	Product	Yield	*Z*/*E*	ee (%)
1	Me (**1a**)	A	**3a**	99%	1 : 0	96
2	Bn (**1b**)	A	**3b**	94%	1 : 0	92
3	Allyl (**1c**)	A	**3c**	93%	1 : 0	91
4	H (**1d**)	A	**3d**	95%	1 : 0	72
5	CO_2_Et (**1e**)	B	**4a**	95%	5.3 : 1	95, 92
6	Ts (**1f**)	B	**4b**	86%	4.2 : 1	91, —
7	Ac (**1g**)	B	**4c**	67%	7.3 : 1	94, —

^*a*^Condition A: DCM, –60 °C, 20 min; condition B: DCE, –30 °C, <1 min.

The scope of the 3-styrylindole for the asymmetric [2+2]-cycloaddition reaction was found to be general (Table 2). For example, the styryl moieties with both electron-rich and electron-deficient aryl groups are compatible, furnishing the desired [2+2]-cycloadducts in 96–99% yield with excellent ee (95–96% ee) (Table 2, entries 1–5). Moreover, different substituents, such as MeO, Me and Br, could be introduced to different positions of the indole moiety and the corresponding [2+2]-cycloadducts could be obtained in 76–99% yield with excellent enantioselectivities (Table 2, entries 6–9); however, the reaction required either a higher catalyst loading (5 mol%) or a much longer reaction time (12 h) for those 3-styrylindoles with electron-donating groups such as MeO and Me (Table 2, entries 6 and 7). In particular, besides styryl, the R^1^ could be a heteroaryl, such as thienyl (**1q**), and the reaction also worked very well to give the desired product **3n** in 90% yield with 93% ee (Table 2, entry 10). Gratifyingly, the reaction of **1r** with an aliphatic R^1^ group could produce the desired [2+2] cycloadduct **3o** in 58% yield with an acceptable 82% ee with the use of (*R*,*R*,*R*)-**L6** as the chiral ligand, but the reaction of **1s** with a terminal olefin was very messy (Table 2, entries 11–12). Finally, the reaction scope of this asymmetric [2+2]-cycloaddition was evaluated by variation of R^2^ on the allenamides, **2**. The desired products **3q–3r** could be produced in close to quantitative yield with 96% ee (Table 2, entries 13–14).

**Table 2:** Asymmetric [2+2]-cycloaddition

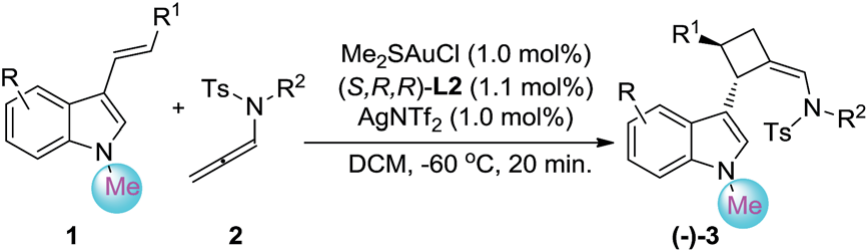					
Entry	**1**, R^1^, R	**2**, R^2^	**3**	Yield^*a*^	ee
1	**1h**, 4-BrC_6_H_4_/H	**2a**, Ph	**3e**	98%	96%
2	**1i**, 4-ClC_6_H_4_/H	**2a**	**3f**	98%	96%
3	**1j**, 4-CH_3_C_6_H_4_/H	**2a**	**3g**	97%	95%
4	**1k**, 4-CF_3_C_6_H_4_/H	**2a**	**3h**	99%	96%
5^*b*^	**1l**, 4-CH_3_OC_6_H_4_/H	**2a**	**3i**	96%	95%
6^*c*^	**1m**, Ph/5-CH_3_O	**2a**	**3j**	76%	96%
7^*c*^	**1n**, Ph/5-CH_3_	**2a**	**3k**	91%	95%
8	**1o**, Ph/5-Br	**2a**	**3l**	99%	95%
9	**1p**, Ph/4-Br	**2a**	**3m**	93%	95%
10^*c*^ ^,^^*d*^	**1q**, 2-thienyl/H	**2a**	**3n**	90%	93%
11^*e*^	**1r**, *n*-Pr/H	**2a**	**3o**	58%	82%
12^*f*^	**1s**, H/H	**2a**	**3p**	—	—
13	**1a**	**2b**, 4-CH_3_OC_6_H_4_	**3q**	98%	96%
14	**1a**	**2c**, 4-BrC_6_H_4_	**3r**	99%	96%

^*a*^Isolated yield.

^*b*^Reacting for 30 min.

^*c*^5 mol% of catalyst.

^*d*^–50 °C for 10 min, then –20 °C for another 5 min.

^*e*^(*R*,*R*,*R*)-**L6** was used.

^*f*^The reaction was messy.

In addition to the asymmetric [2+2]-cycloadditions, various substituted *N*-CO_2_Et 3-styrylindoles are applicable to [4+2]-cycloadditions, leading to the optically active tetrahydrocarbazoles in good to excellent yields with a *Z* : *E* selectivity of up to 14 : 1. Gratifyingly, both the *Z*- and *E*-isomers could be obtained in high levels of enantioselectivity (88–97% ee). However, substrates **1r′** with aliphatic R^1^ and **1s′** with a terminal olefin could not give the corresponding [4+2]-cycloadducts, which is attributed to the lower reactivity of the aliphatic alkenylindole (Table 3, entries 11–12).

**Table 3:** Asymmetric [4+2]-cycloaddition

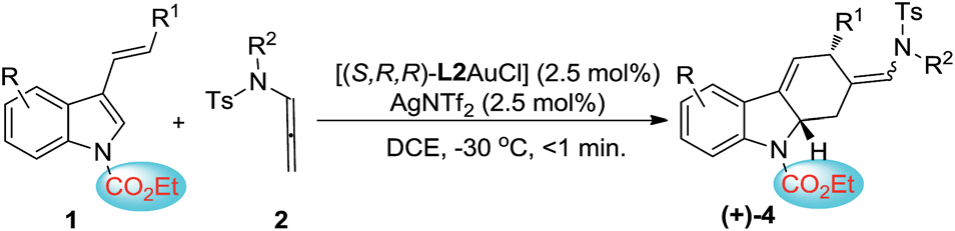						
Entry	**1′** ^*a*^, R^1^, R	**2**, R^2^	**4**	*Z* : *E*	Total yield	ee (%) of *Z*, *E*
1	**1h′**, 4-BrC_6_H_4_/H	**2a**	**4d**	3.5 : 1	98%	97, 90
2	**1i′**, 4-ClC_6_H_4_/H	**2a**	**4e**	3.4 : 1	95%	95, 90
3	**1j′**, 4-CH_3_C_6_H_4_/H	**2a**	**4f**	5.6 : 1	95%	96, 90
4	**1k′**, 4-CF_3_C_6_H_4_/H	**2a**	**4g**	3.8 : 1	82%	97, —^*b*^
5	**1l′**, 4-CH_3_OC_6_H_4_/H	**2a**	**4h**	3.0 : 1	88%	95, —^*b*^
6	**1m′**, Ph/5-CH_3_O	**2a**	**4i**	4.7 : 1	88%	97, 92
7	**1n′**, Ph/5-CH_3_	**2a**	**4j**	5.0 : 1	92%	96, 88
8	**1o′**, Ph/5-Br	**2a**	**4k**	5.2 : 1	97%	97, 92
9	**1p′**, Ph/4-Br	**2a**	**4l**	6.1 : 1	99%	97, 92
10	**1q′**, 2-thienyl/H	**2a**	**4m**	1 : 1	90%	93, 91
11	**1r′**, *n*-Pr/H	**2a**	**4n**	—	0%^*c*^	—, —
12	**1s′**, H/H	**2a**	**4o**	—	0%^*c*^	—, —
13	**1e**	**2b**	**4p**	10 : 1	89%^*d*^	90, —
14	**1e**	**2c**	**4q**	14 : 1	92%^*d*^	89, —

^*a*^ **1h′** means the substrate is bearing the same substituents at R and R^1^ as **1h** in Table 2 but has a different *N*-substituent.

^*b*^The ee could not be determined by chiral HPLC.

^*c*^Only the dimer of **2a** was isolated.

^*d*^Yield of the isolated (*Z*)-isomer.

**Scheme 3:**
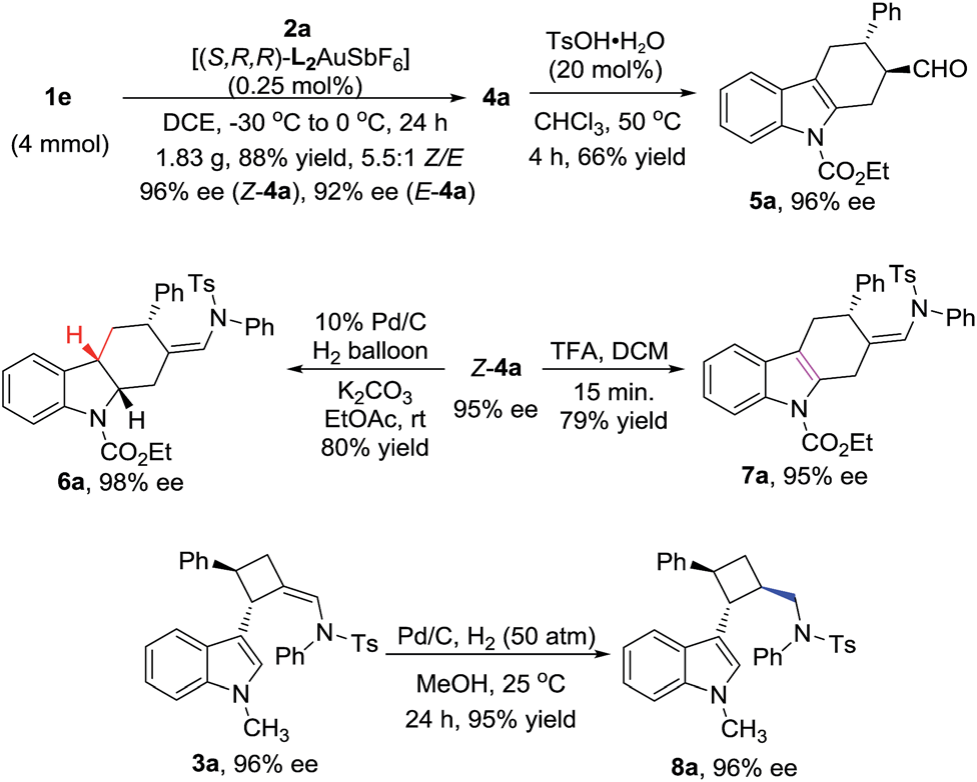
Gram-scale synthesis and synthetic applications.

To test the practicality of the new methodology, a gram-scale reaction was carried out. To our delight, the catalyst loading could be reduced to only 0.25 mol% on a 4 mmol scale to furnish 1.83 g of **4a** in 88% yield with a 5.5 : 1 *Z*/*E* ratio and excellent enantioselectivity (*Z*-**4a**, 96% ee, and *E*-**4a**, 92% ee), indicating that this transformation is easy to scale-up to gram scale without a loss in the efficiency or enantioselectivity (Scheme 3). To showcase the synthetic applications, three transformations of product **4a** were carried out. The first transformation was the process involving the selective hydrolysis of the *exo* enamide moiety and the endocyclic double bond migration of **4a** under the catalysis of TsOH at 50 °C, leading to the formation of aldehyde **5a** in 66% yield with 96% ee. The second transformation was the selective hydrogenation of the endocyclic double bond of *Z*-**4a** at room temperature under the catalysis of 10 mol% Pd/C; the optically active hexahydrocarbazole **6a** with three chiral stereocenters could be isolated in 80% yield with 98% ee. The last transformation was the selective endocyclic double bond migration under acidic conditions, producing a new type of tetrahydrocarbazole **7a** in 79% yield without the loss of the chiral information. Meanwhile, the *exo* enamide of **3a** could also be hydrogenated at high pressure, and the highly strained cyclobutane **8a** with 96% ee could be produced in 95% yield.

**Fig. 2:**
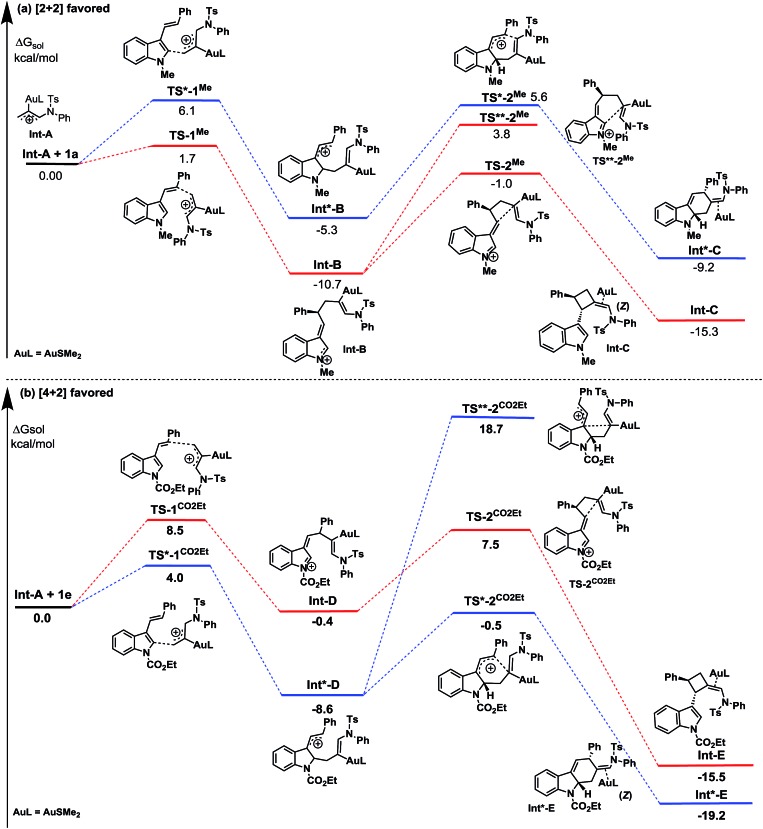
The free energy profiles Δ*G*_sol_ for the reaction of 3-styrylindoles **1a** and **1e** with the Au-allyl cation species **Int-A**.

**Fig. 3:**
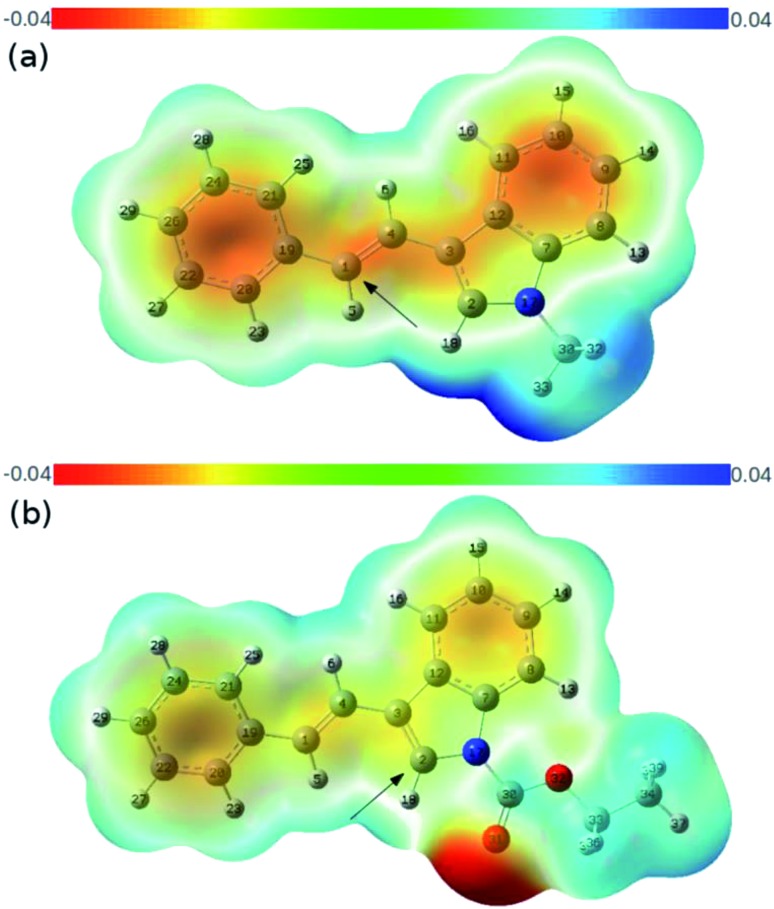
The calculated contour maps of the electrostatic potentials corresponding to (a) **1a** and (b) **1e** in Fig. 2, respectively.

To rationalize the origin of this dramatic substituent effect on the cycloaddition mode under the catalysis of gold,^15^ we performed Density Functional Theory (DFT) calculations using the M06 method to calculate the reaction pathway. We presumed that both cycloadditions were stepwise reactions.^3^–^6^ The calculated free energy profiles of the cycloaddition reactions of the 3-styrylindole **1a** with **Int-A** at the temperature 298 K are shown in Fig. 2(a). This figure shows the cycloaddition reaction of *N*-methyl 3-styrylindole **1a** and **Int A**. Fig. 3(a) clearly indicates that the electrostatic potential in the vicinity of the *N*-CH_3_ is positive due to the effect of the electron-donating methyl. In contrast, the moiety of the double bond in styryl exhibits a negative electrostatic potential, which tends to interact with positively charged species (*E***_TS-1^Me^_** = 1.7 kcal mol^–1^*vs. E*_TS*-1^Me^_ = 6.1 kcal mol^–1^), leading to a quite stable iminium **Int-B** with a releasing energy of 10.7 kcal mol^–1^. For the second reaction step of the ring closure, the barrier of **TS-2^Me^** is still lower than the corresponding TS*-2^Me^ by 1.2 kcal mol^–1^ (*E***_TS-2^Me^_** = 9.7 kcal mol^–1^*vs. E*_TS*-2^Me^_ = 10.9 kcal mol^–1^). In addition, the formation of the [2+2] cycloaddition may be favoured by the stereoelectronic effect in energetics (Δ*E*_**TS-2^Me^**–TS**-2^Me^_ = 4.8 kcal mol^–1^). Furthermore, **Int-C** that leads to the [2+2] cycloadduct is more stable than Int*-C by 6.1 kcal mol^–1^. Moreover, the complete (*Z*)-selectivity of the [2+2] cycloaddition product can also be easily explained using the computational calculations (please see the details in the ESI†). Thus, the DFT calculations indicate that the (*Z*)-isomer of the [2+2]-cycloadduct is highly controlled with the *N*-methyl 3-styrylindole **1a** as the substrate and the [2+2] cycloaddition is more favorable than the [4+2] cycloaddition with the *N*-methyl 3-styrylindole **1a** as the substrate.

This is in contrast to when the *N*-CO_2_Et 3-styrylindole **1e** was used as a substrate (Fig. 2(b)). Fig. 3(b) shows that the calculated electrostatic potential of the *N*-CO_2_Et in **1e** is completely contrary to that of **1a**. Due to the electron-withdrawing effect of *N*-CO_2_Et, the vicinity of the oxygen atom of the carbonyl group exhibits a large negative electrostatic potential, which strongly induces an electrostatic interaction with the Au-allyl cation species **Int-A**. Thus, the first addition of **Int-A** occurs at the carbonic position of the indole ring in **1e**, rather than at the double bond of the styryl, which leads to the intermediate Int*-D (Δ*E*_TS*-1CO_2_Et–**TS-1CO_2_Et**_ = –4.5 kcal mol^–1^). The barriers of TS*-2^CO_2_Et^ leading to the [4+2]-cycloadduct and **TS-2^CO_2_Et^** leading to the [2+2]-cycloadduct are very close, with energies of 8.1 and 7.9 kcal mol^–1^, respectively. By avoiding the drastic steric effects in TS**-2^CO_2_Et^, the Int*-D proceeds through the transition state TS*-2^CO_2_Et^ (Δ*E*_TS*-2CO_2_Et–TS**-2CO_2_Et_ = –19.2 kcal mol^–1^). In addition, the Int*-E is thermodynamically more favorable than **Int-E**. Moreover, the results in which (*Z*) and (*E*)-[4+2] cycloaddition isomers are both obtained, are due to the small steric factor (the *exo*-double bond of the six-membered ring) and influenced by the substrates,^16^ catalysts and ligands. In short, the current DFT calculations could well elucidate the chemoselectivity of the 3-styrylindole with different electron-donating and electron-withdrawing groups.

## Conclusions

In summary, we have developed a gold(i)-catalyzed highly enantioselective intermolecular [2+2] or [4+2]-cycloaddition of various substituted 3-styrylindoles with *N*-allenamides. The discrimination between the anticipated [4+2]-product and the unexpected [2+2]-cycloaddition adduct is dependent on the electronic nature of the *N*-substituent of the 3-styrylindoles, which is well rationalized by DFT calculations. With electron-withdrawing groups, such as CO_2_Et, Ts and Ac, the [4+2] cycloaddition occurs; in contrast, the [2+2]-cycloadditions take place with electron-donating groups such as methyl, benzyl, allyl and even H groups. The salient features of the present method, including using readily available starting materials and catalysts, high enantioselectivity, good yields, the ease of scale-up to gram scale, and atom-economy, make it give extremely facile access to optically active substituted cyclobutanes and tetrahydrocarbazoles, which are highly valuable building blocks in organic synthesis. Further studies, including the design of a new chiral ligand to address the unsatisfactory enantioselectivity issue of the oxazolidinone-based allenamide,^16^ are under way in this laboratory and will be reported in due course.

## Supplementary Material

Supplementary information

Supplementary information

Crystal structure data
