# Supplementary material for: Enantioselective gold-catalyzed intermolecular [2+2] versus [4+2]-cycloadditions of 3-styrylindoles with N-allenamides: observation of interesting substituent effects
Source: Chem Sci. 2015 Jun 23;6(10):5564–70. doi: 10.1039/c5sc01827g (PMC5949851; doi:10.1039/c5sc01827g)
Supplement: Supplementary file 1 [file SC-006-C5SC01827G-s001.pdf]

Electronic Supplementary Material (ESI) for Chemical Science.
This journal is © The Royal Society of Chemistry 2015

wyd-4-30-2 H

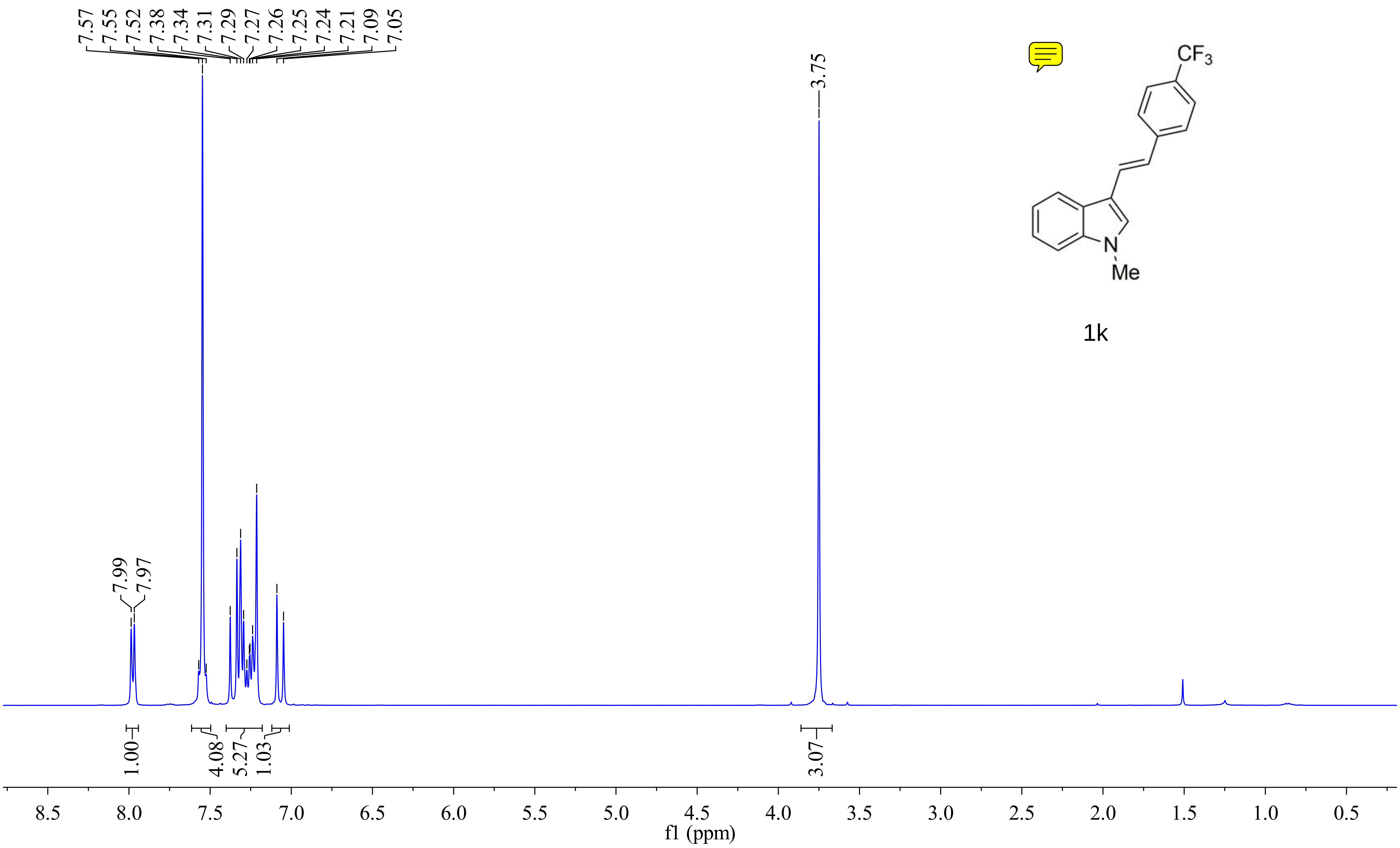

wyd-4-30-2 F

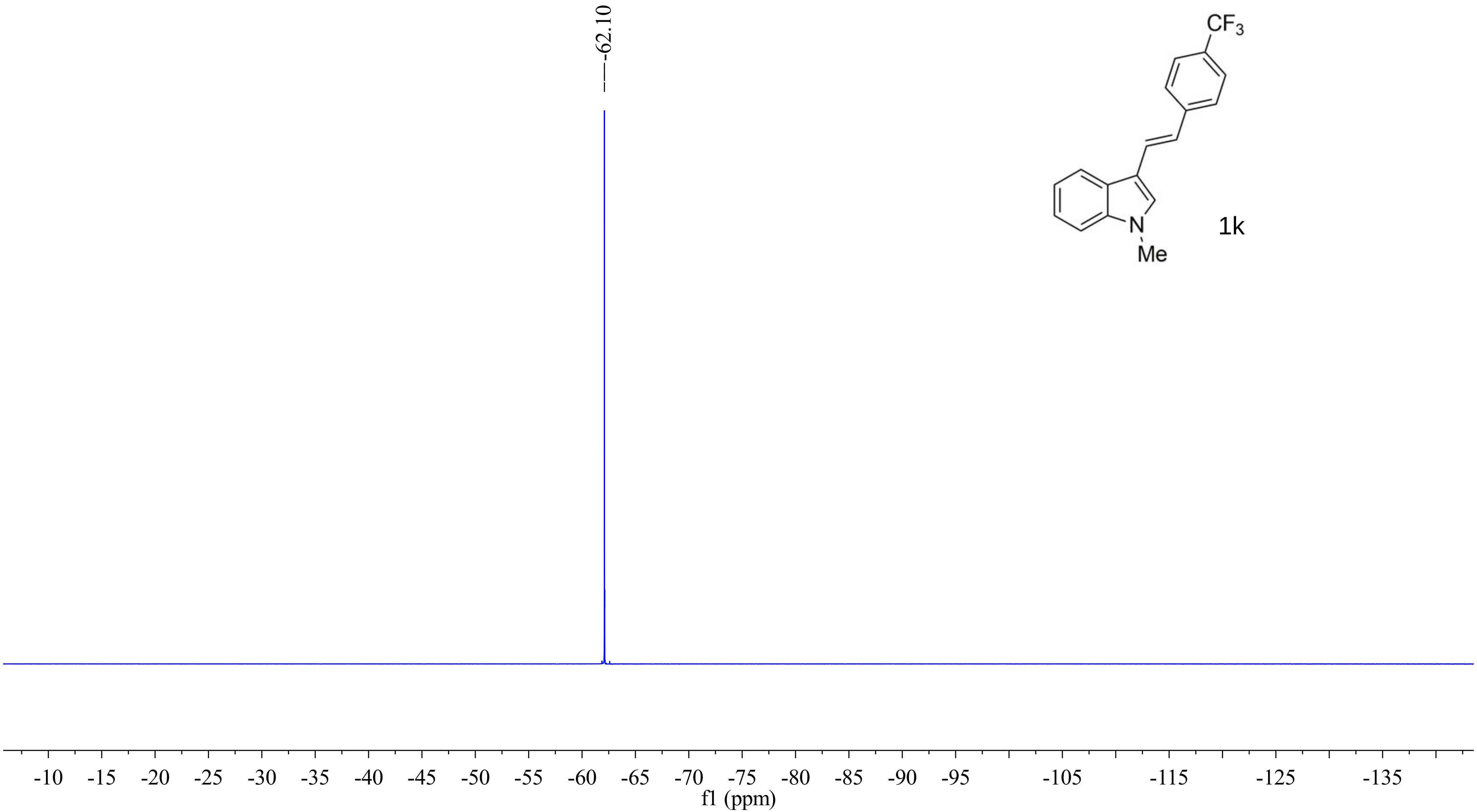

-62.10
-10
-15
-20
-25
-30
-35
-40
-45
-50
-55
-60
-65
-70
-75
-80
-85
-90
-95
-105
-115
-125
-135
f1 (ppm)
CF3
N
Me

1k

wyd 4-30-2 C

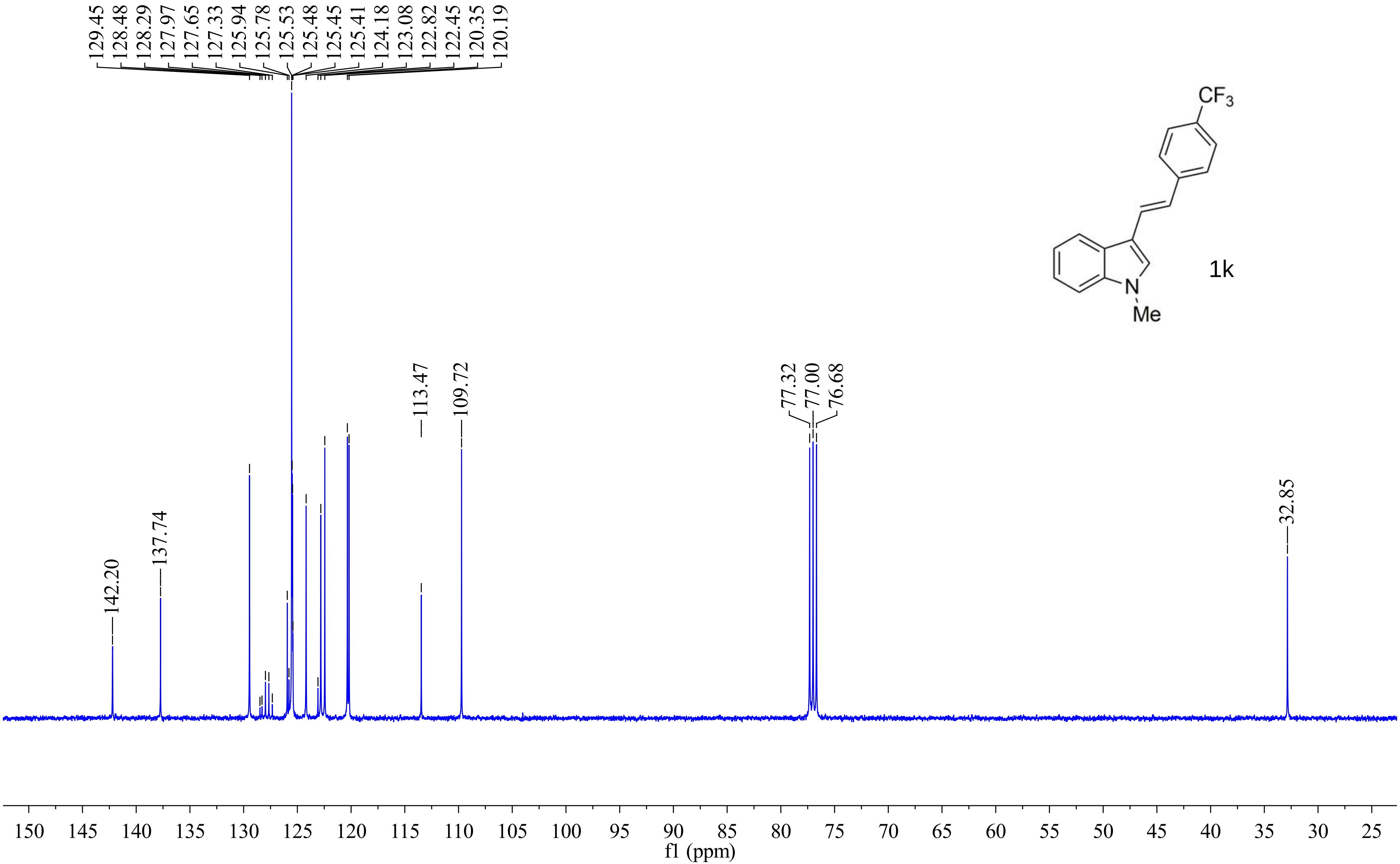

129.45
128.48
128.29
127.97
127.65
127.33
125.94
125.78
125.53
125.48
125.45
125.41
124.18
123.08
122.82
122.45
120.35
120.19
142.20
137.74
113.47
109.72
77.32
77.00
76.68
32.85
CF3
N
Me
1k
150 145 140 135 130 125 120 115 110 105 100 95 90 85 80 75 70 65 60 55 50 45 40 35 30 25
f1 (ppm)

wyd-6-49-2 H

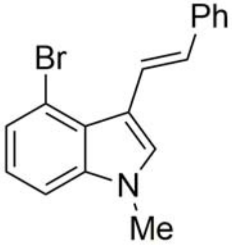

Ph
Br
N
Me

1p

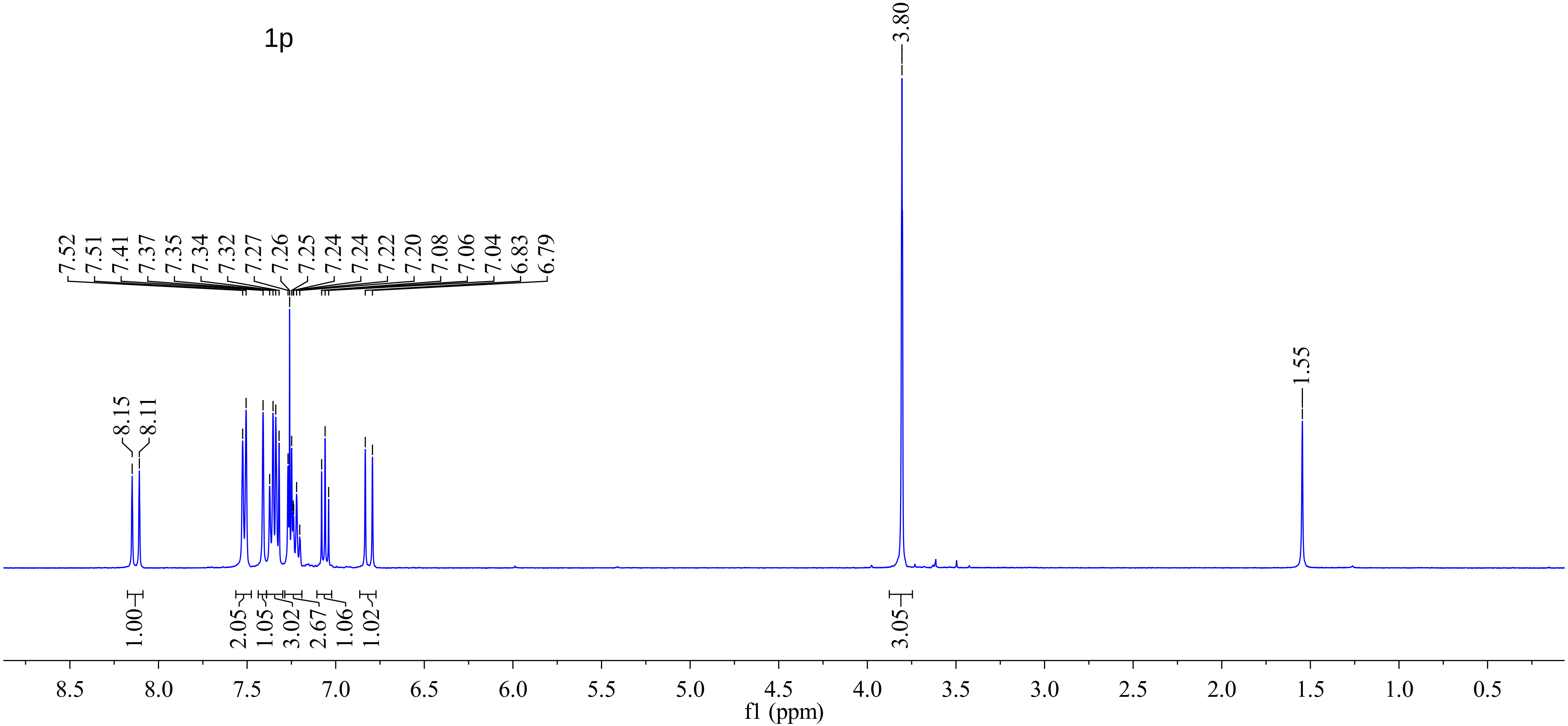

7.52
7.51
7.41
7.37
7.35
7.34
7.32
7.27
7.26
7.25
7.24
7.24
7.22
7.20
7.08
7.06
7.04
6.83
6.79
8.15
8.11
3.80
1.55
1.00
2.05
1.05
3.02
2.67
1.06
1.02
3.05
8.5
8.0
7.5
7.0
6.5
6.0
5.5
5.0
4.5
4.0
3.5
3.0
2.5
2.0
1.5
1.0
0.5
f1 (ppm)

wyd-6-49-2 C

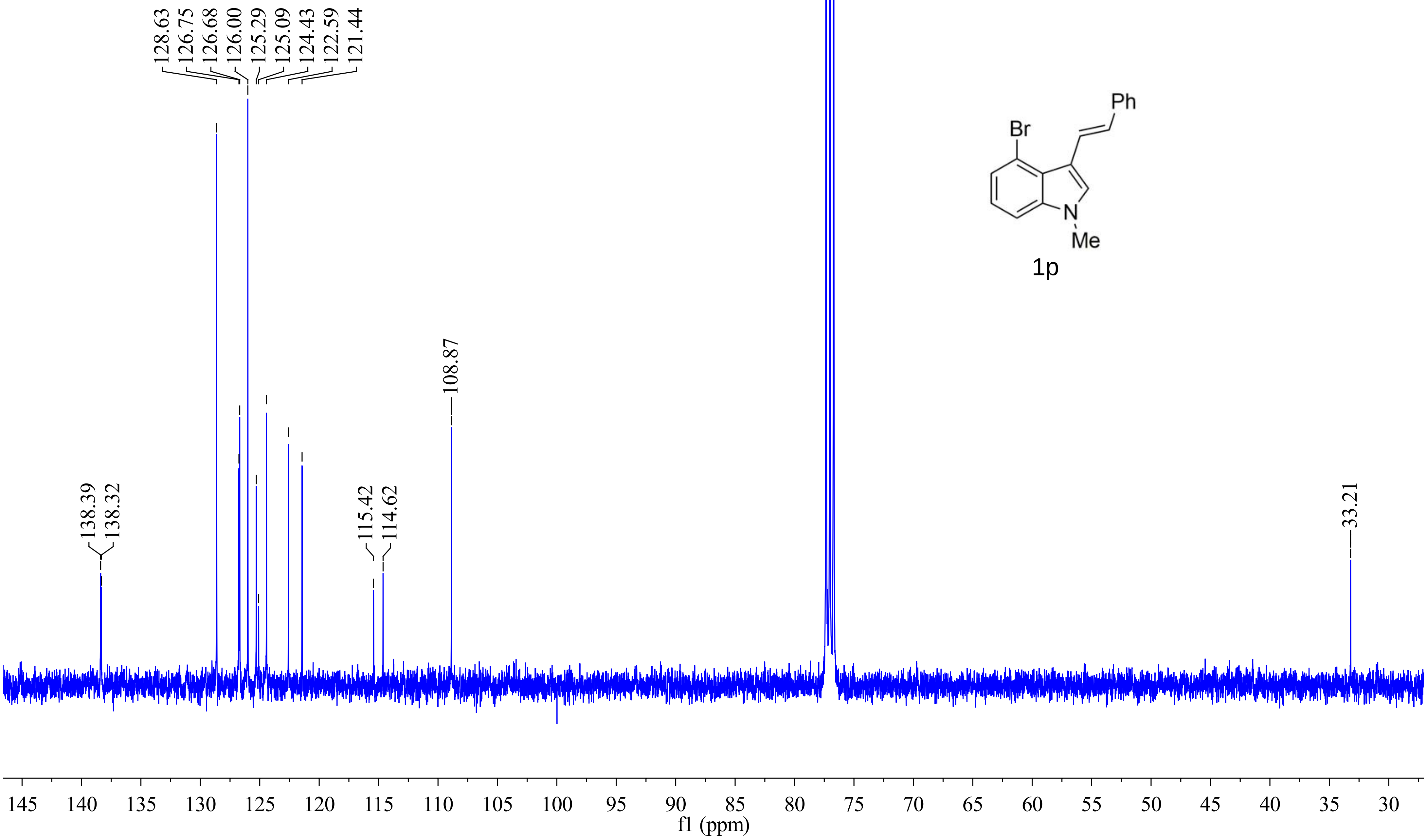
128.63
126.75
126.68
126.00
125.29
125.09
124.43
122.59
121.44
108.87
138.39
138.32
115.42
114.62
33.21
Br
Ph
N
Me
1p
145 140 135 130 125 120 115 110 105 100 95 90 85 80 75 70 65 60 55 50 45 40 35 30
f1 (ppm)

zpc-1-56 H

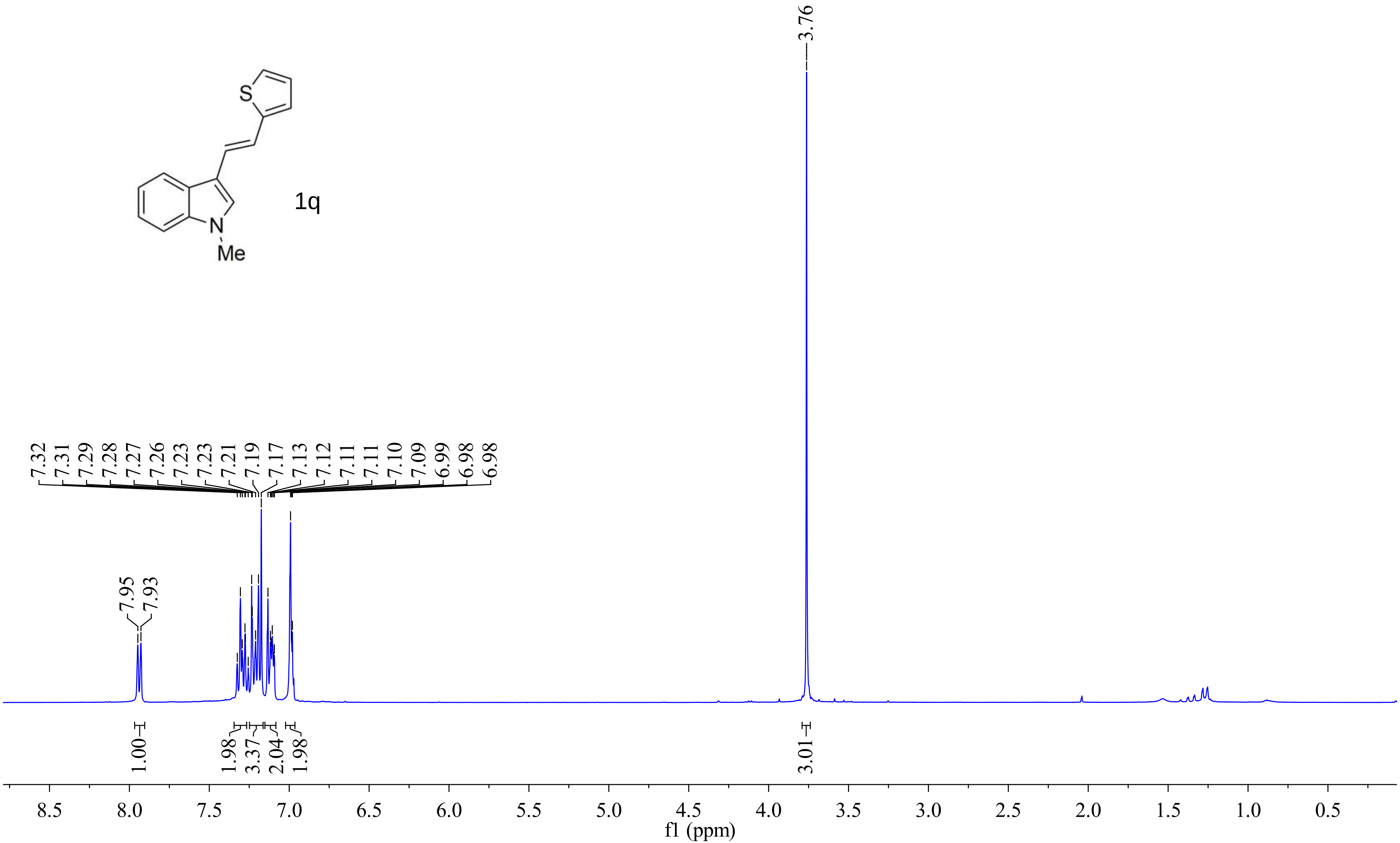
7.32
7.31
7.29
7.28
7.27
7.26
7.23
7.23
7.21
7.19
7.17
7.13
7.12
7.11
7.11
7.10
7.09
6.99
6.98
6.98
7.95
7.93
3.76
1.00
1.98
3.37
2.04
1.98
3.01
S
N
Me
1q
8.5
8.0
7.5
7.0
6.5
6.0
5.5
5.0
4.5
4.0
3.5
3.0
2.5
2.0
1.5
1.0
0.5
f1 (ppm)

zpc-1-56 H

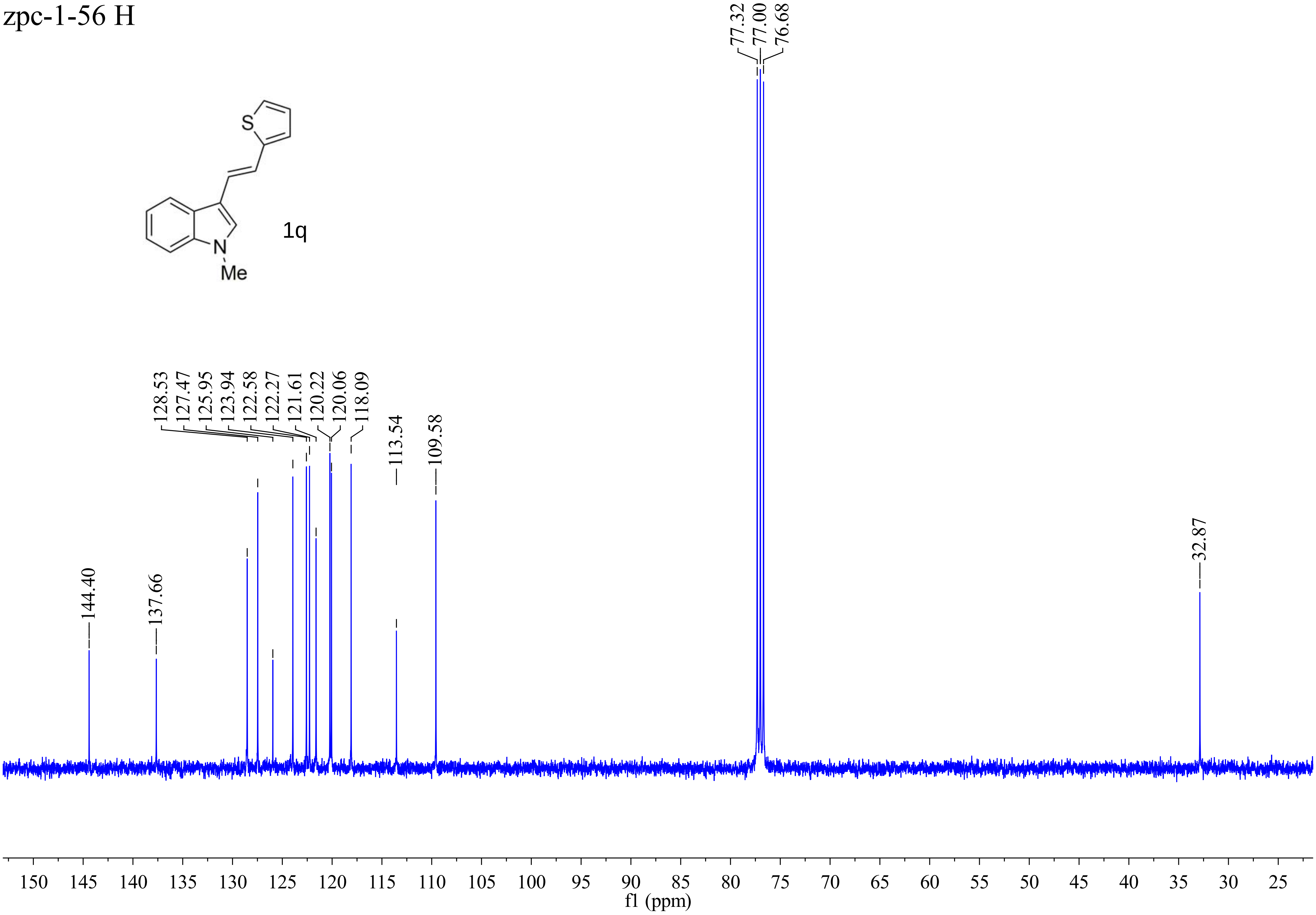
77.32
77.00
76.68
S
N
Me
1q
128.53
127.47
125.95
123.94
122.58
122.27
121.61
120.22
120.06
118.09
113.54
109.58
144.40
137.66
32.87
150 145 140 135 130 125 120 115 110 105 100 95 90 85 80 75 70 65 60 55 50 45 40 35 30 25
f1 (ppm)

wyd-7-12 H

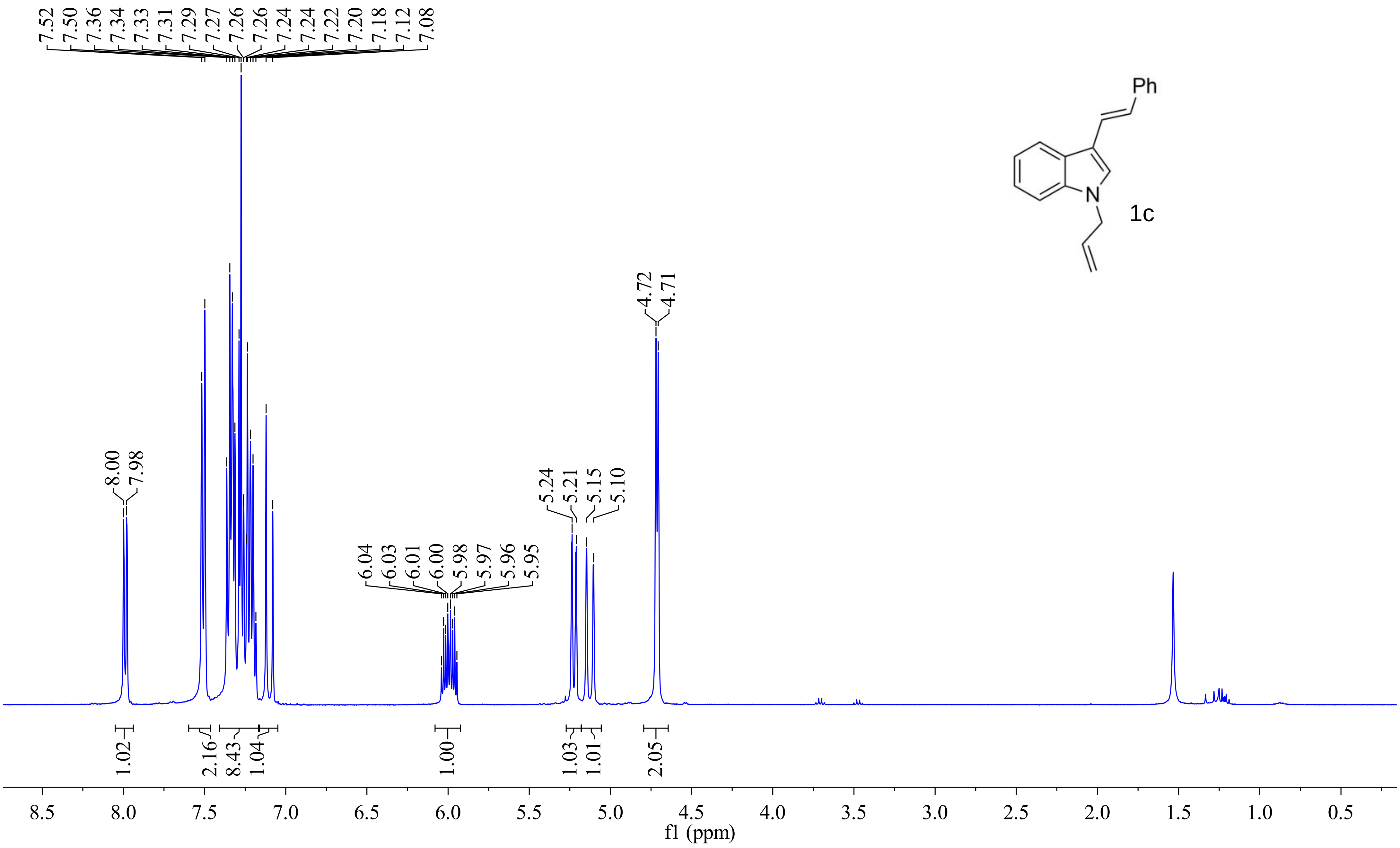
7.52
7.50
7.36
7.34
7.33
7.31
7.29
7.27
7.26
7.26
7.24
7.24
7.22
7.20
7.18
7.12
7.08
8.00
7.98
6.04
6.03
6.01
6.00
5.98
5.97
5.96
5.95
5.24
5.21
5.15
5.10
4.72
4.71
Ph
N
1c
1.02
2.16
8.43
1.04
1.00
1.03
1.01
2.05
8.5
8.0
7.5
7.0
6.5
6.0
5.5
5.0
4.5
4.0
3.5
3.0
2.5
2.0
1.5
1.0
0.5
f1 (ppm)

wyd-7-12 C

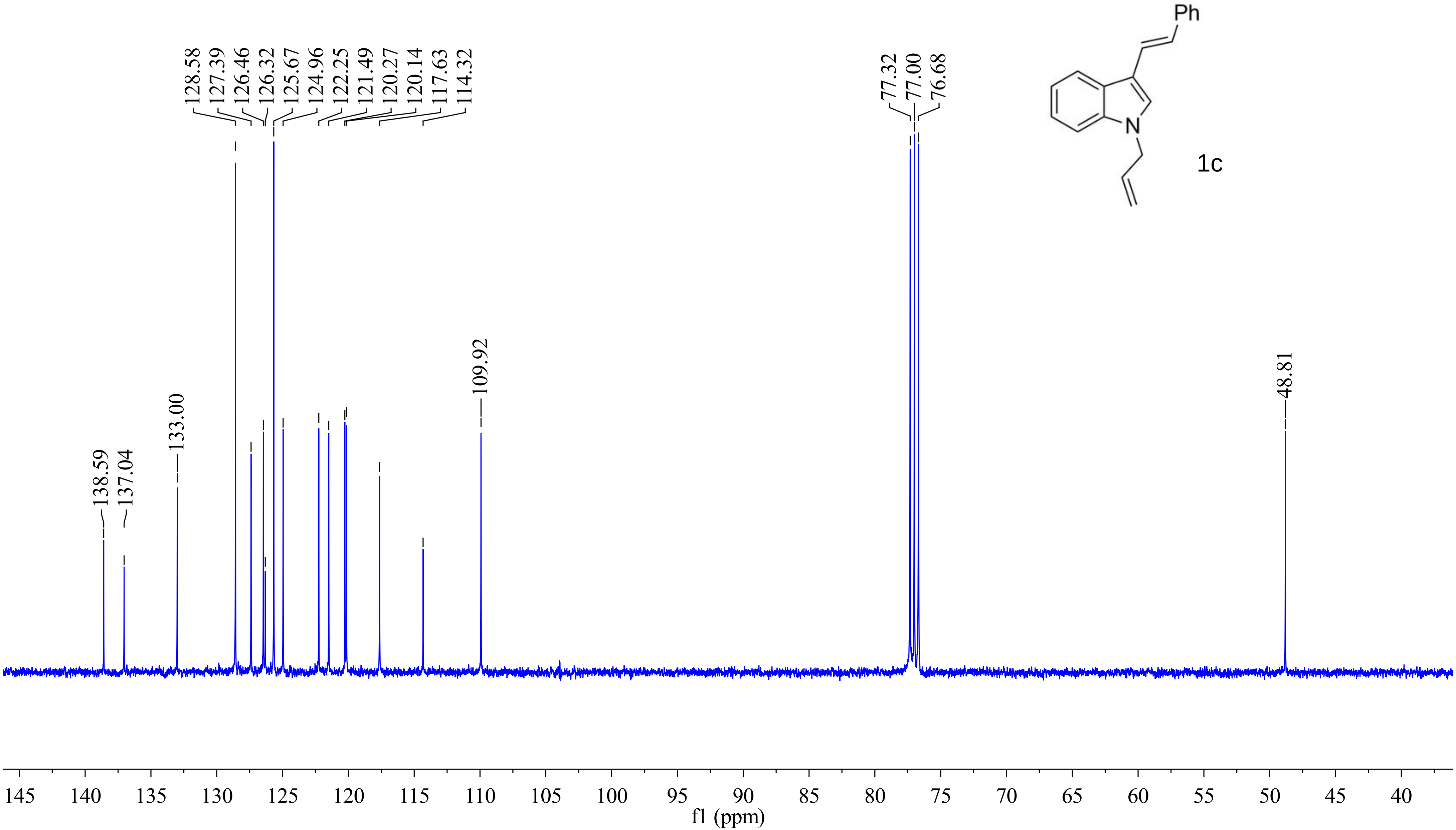
128.58
127.39
126.46
126.32
125.67
124.96
122.25
121.49
120.27
120.14
117.63
114.32
77.32
77.00
76.68
138.59
137.04
133.00
109.92
48.81
145 140 135 130 125 120 115 110 105 100 95 90 85 80 75 70 65 60 55 50 45 40
f1 (ppm)
Ph
N

1c

wyd-4-123-E H

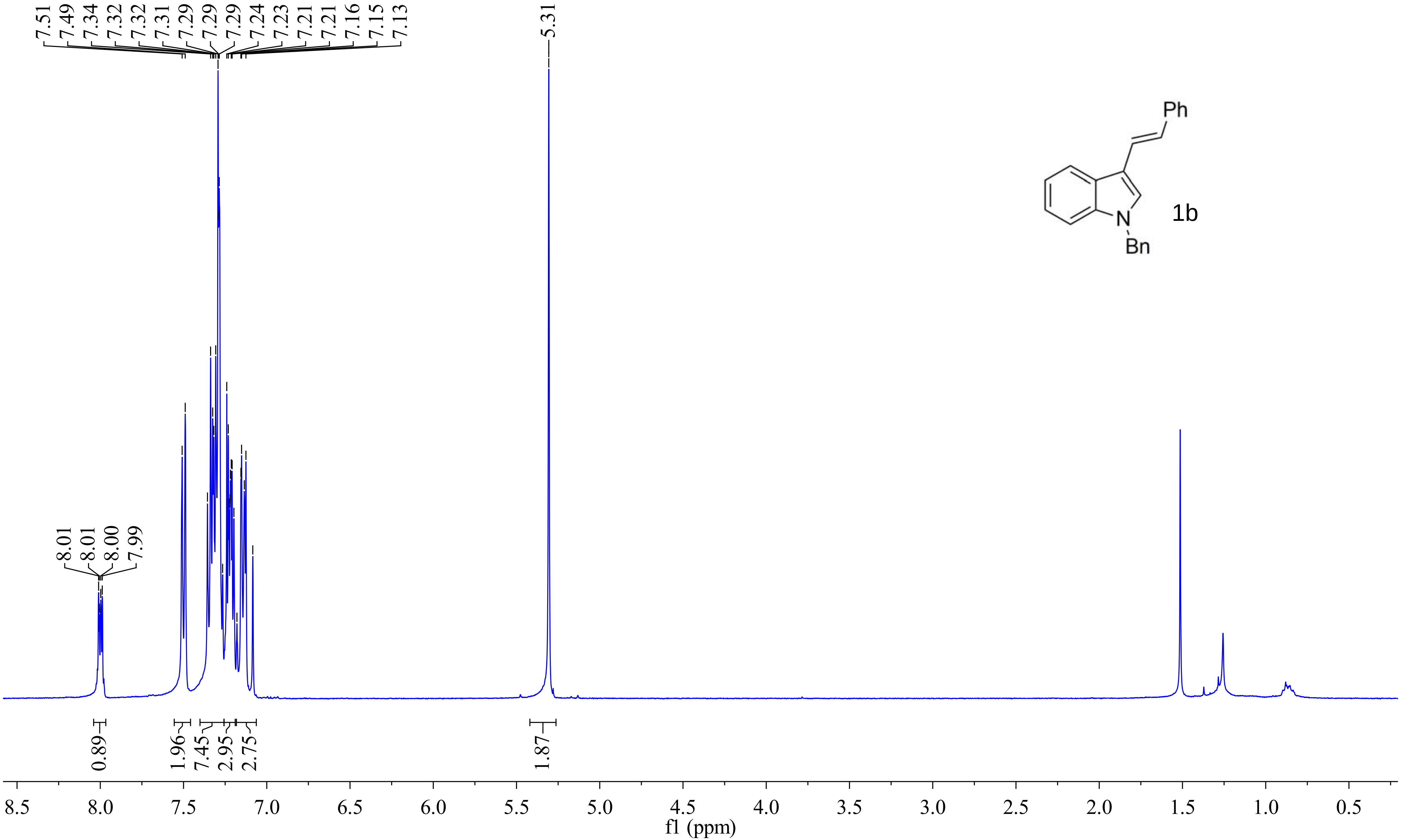
7.51
7.49
7.34
7.32
7.32
7.31
7.29
7.29
7.29
7.24
7.23
7.21
7.21
7.16
7.15
7.13
5.31
8.01
8.01
8.00
7.99
Ph
N
Bn
1b
0.89
1.96
7.45
2.95
2.75
1.87
8.5
8.0
7.5
7.0
6.5
6.0
5.5
5.0
4.5
4.0
3.5
3.0
2.5
2.0
1.5
1.0
0.5
f1 (ppm)

wyd-4-123-E C

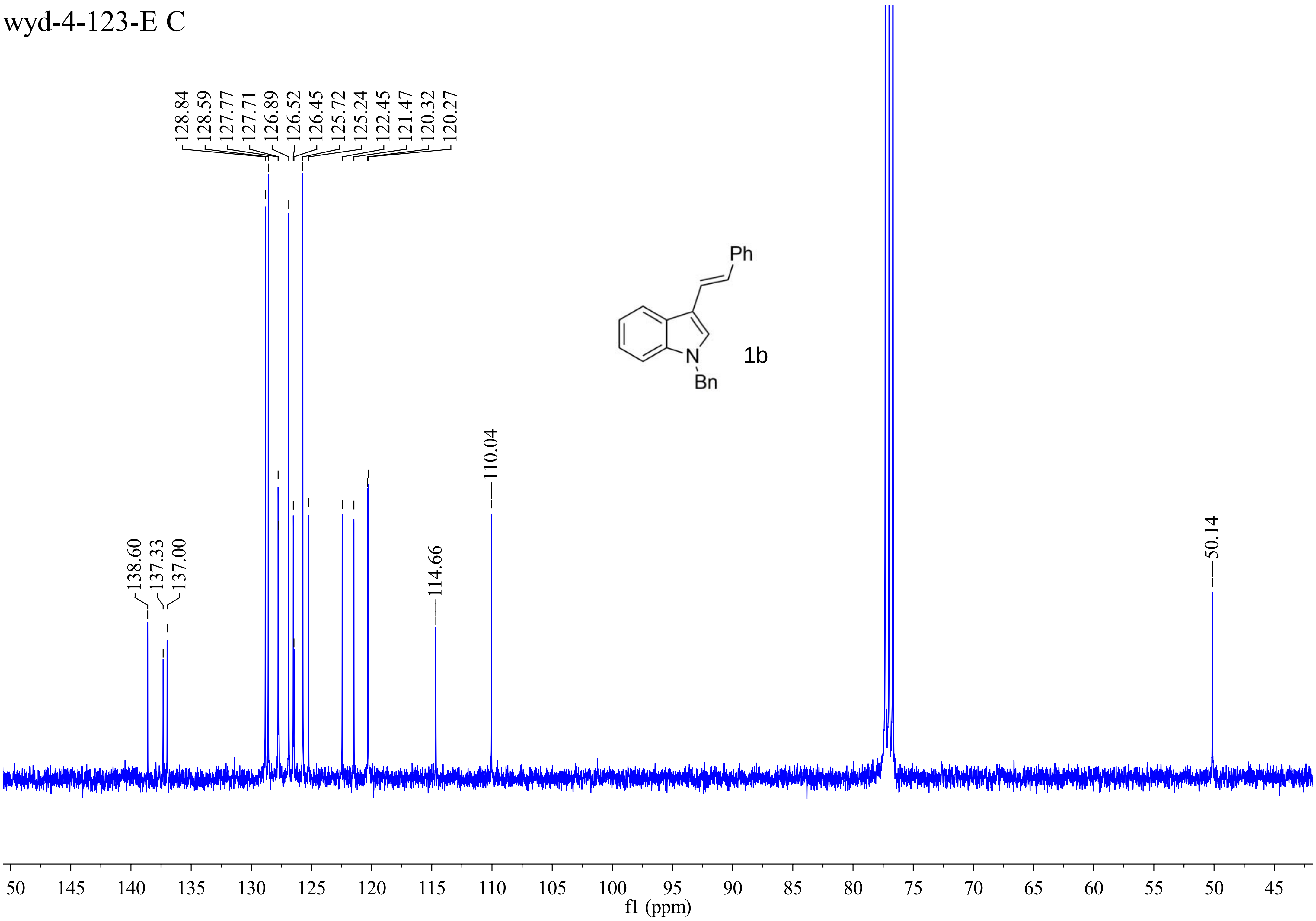
128.84
128.59
127.77
127.71
126.89
126.52
126.45
125.72
125.24
122.45
121.47
120.32
120.27
138.60
137.33
137.00
114.66
110.04
50.14
Ph
N
Bn
1b
150
145
140
135
130
125
120
115
110
105
100
95
90
85
80
75
70
65
60
55
50
45
f1 (ppm)

wyd-6-51-2 H

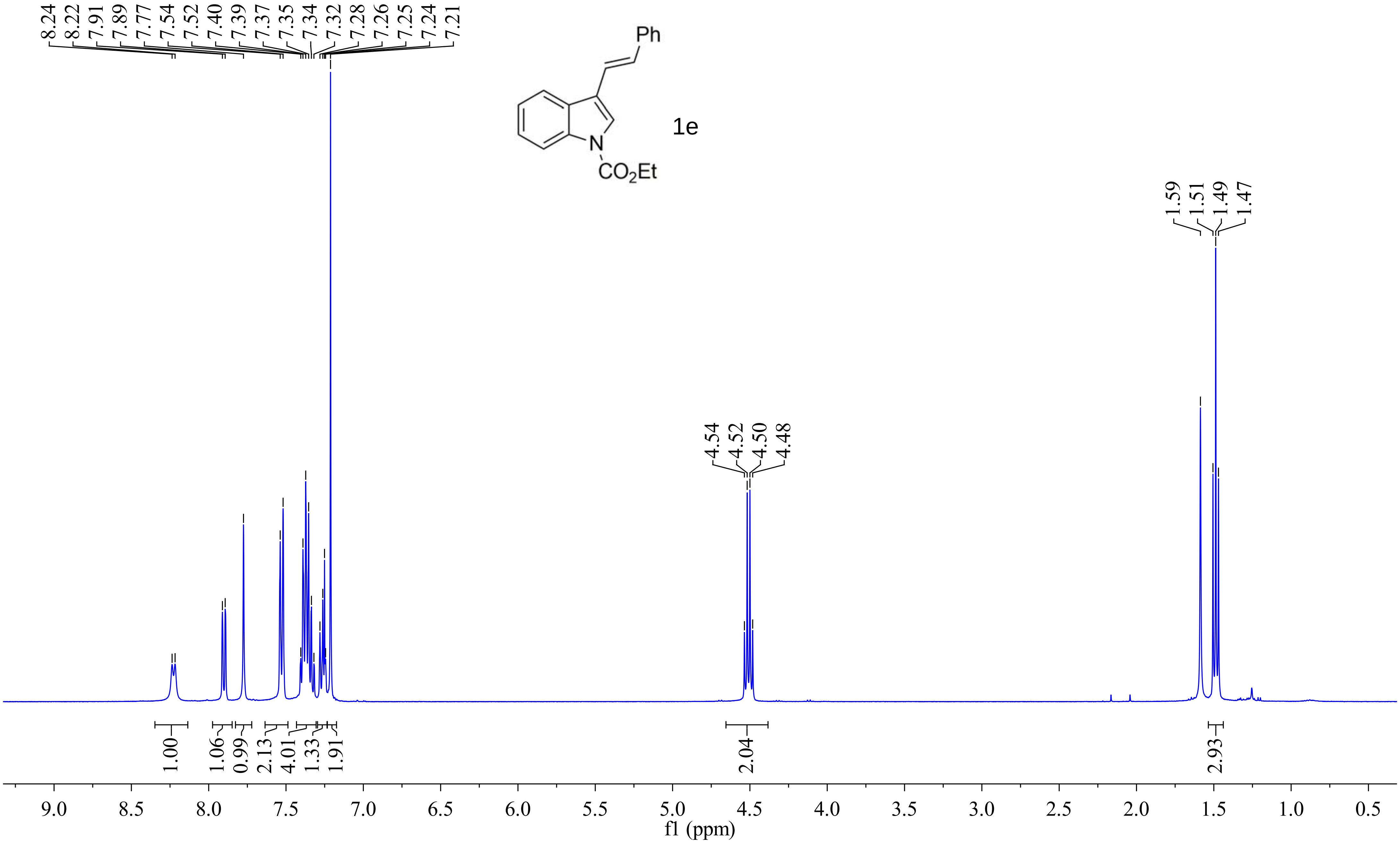
8.24
8.22
7.91
7.89
7.77
7.54
7.52
7.40
7.39
7.37
7.35
7.34
7.32
7.28
7.26
7.25
7.24
7.21
Ph
N
CO2Et
1e
4.54
4.52
4.50
4.48
1.59
1.51
1.49
1.47
1.00
1.06
0.99
2.13
4.01
1.33
1.91
2.04
2.93
9.0
8.5
8.0
7.5
7.0
6.5
6.0
5.5
5.0
4.5
4.0
3.5
3.0
2.5
2.0
1.5
1.0
0.5
f1 (ppm)

wyd-6-51-2 c

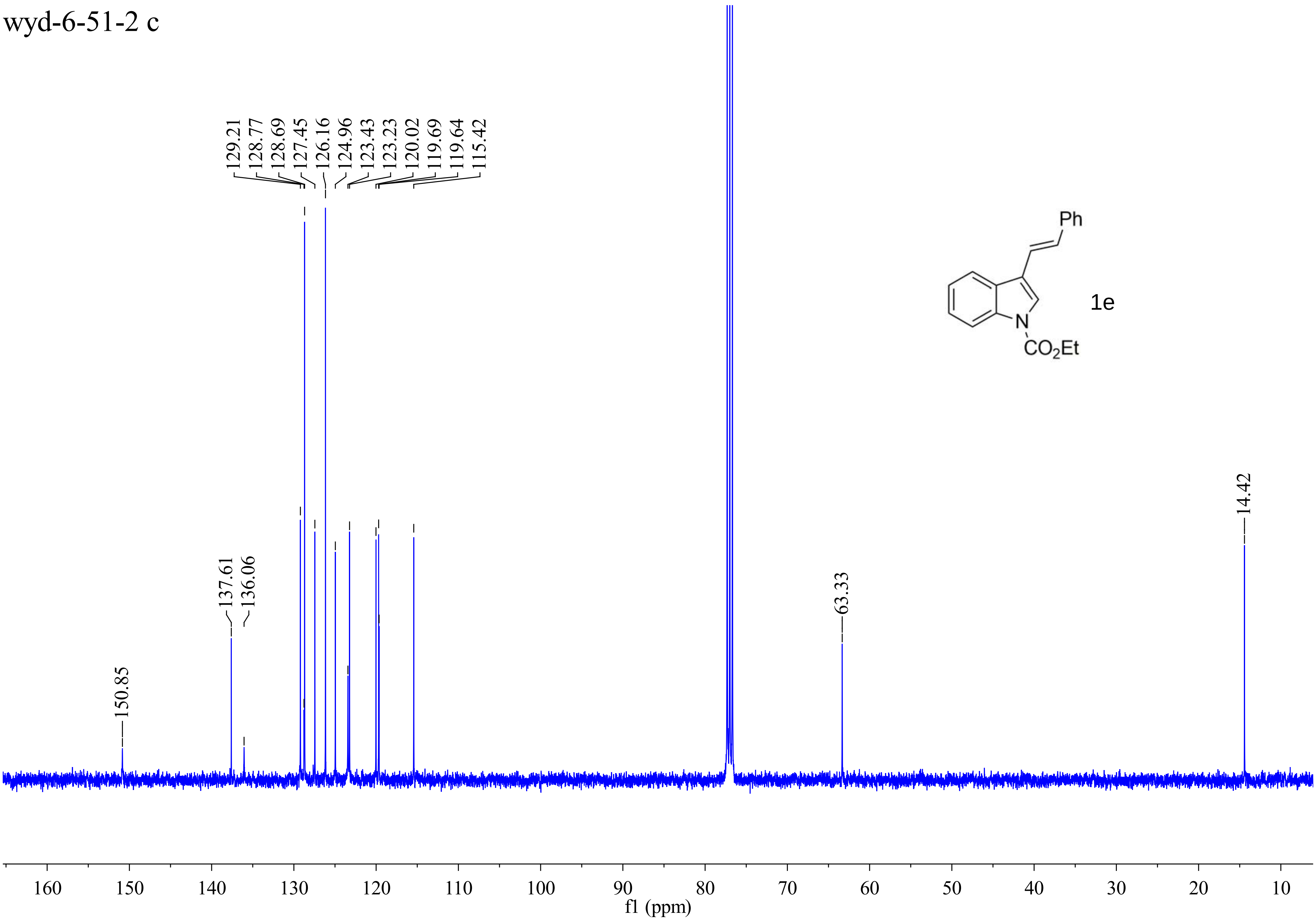

129.21
128.77
128.69
127.45
126.16
124.96
123.43
123.23
120.02
119.69
119.64
115.42
150.85
137.61
136.06
63.33
14.42
Ph
N
CO_2Et
1e
160 150 140 130 120 110 100 90 80 70 60 50 40 30 20 10
f1 (ppm)

zpc-1-15 H

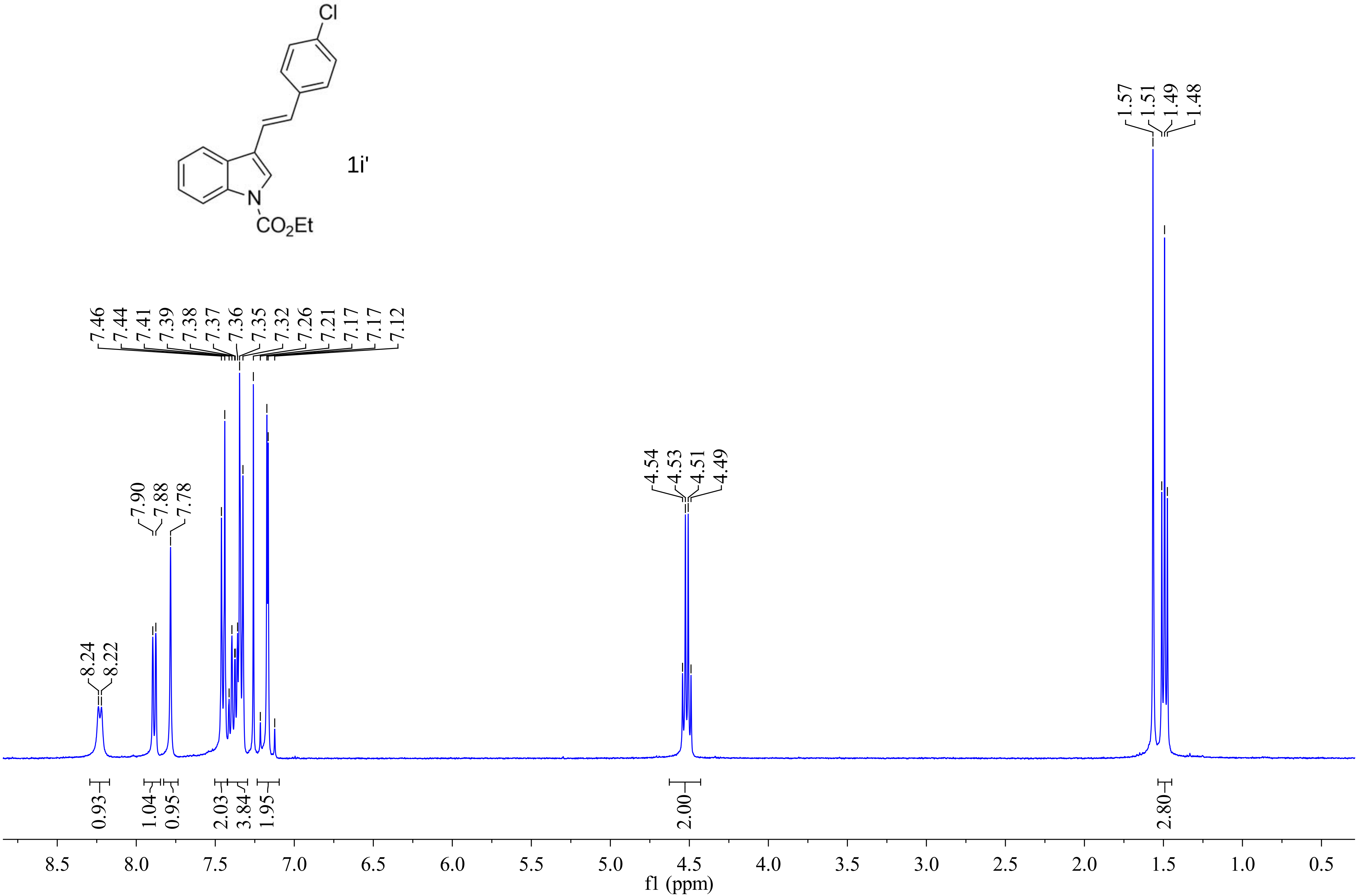
Cl
N
CO2Et
1i'
7.46 7.44 7.41 7.39 7.38 7.37 7.36 7.35 7.32 7.26 7.21 7.17 7.17 7.12
7.90 7.88 7.78
8.24 8.22
4.54 4.53 4.51 4.49
1.57 1.51 1.49 1.48
0.93 1.04 0.95 2.03 3.84 1.95 2.00 2.80
8.5 8.0 7.5 7.0 6.5 6.0 5.5 5.0 4.5 4.0 3.5 3.0 2.5 2.0 1.5 1.0 0.5
f1 (ppm)

zpc-1-15 C

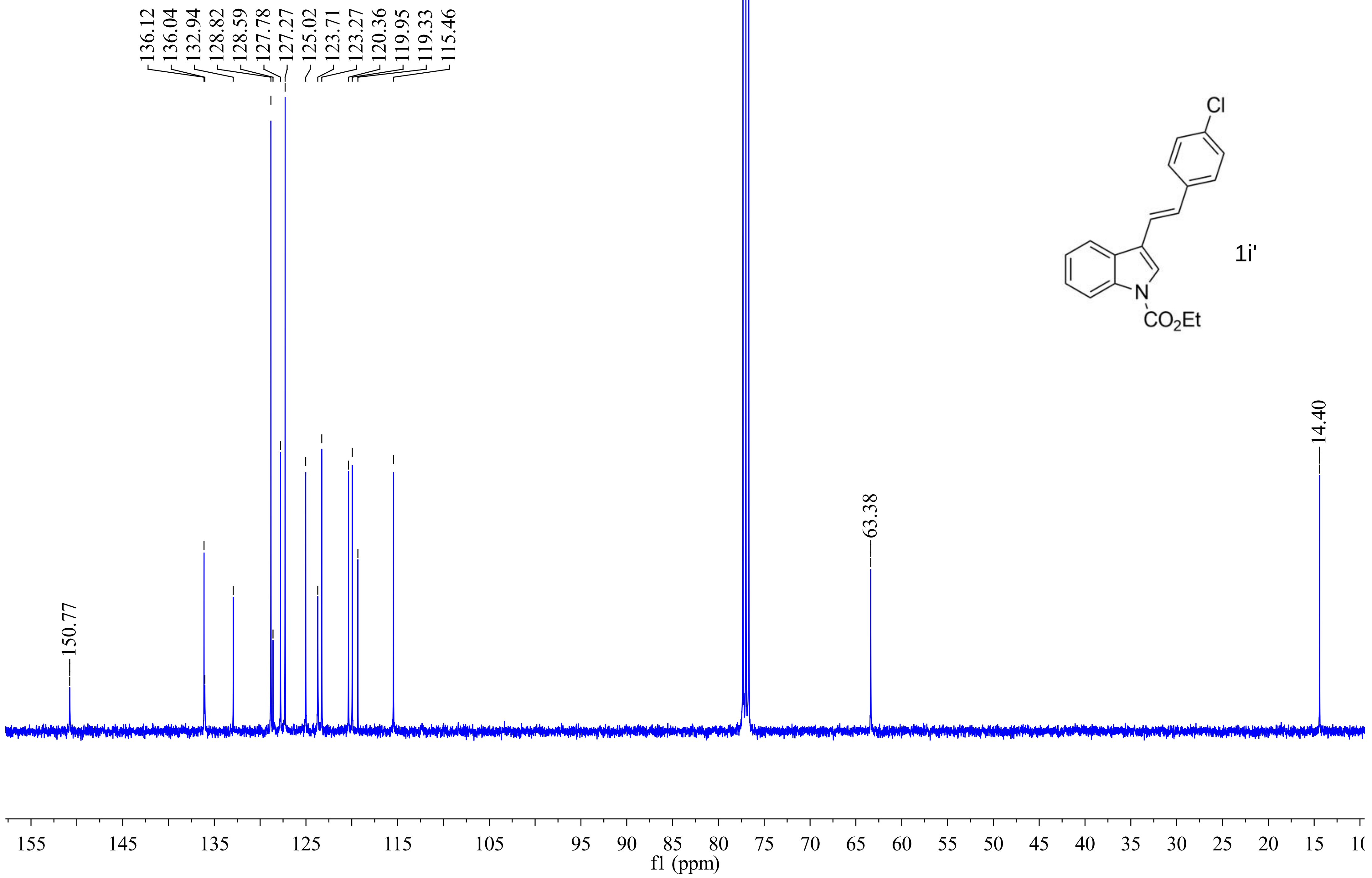
136.12
136.04
132.94
128.82
128.59
127.78
127.27
125.02
123.71
123.27
120.36
119.95
119.33
115.46
150.77
63.38
14.40
155
145
135
125
115
105
95
90
85
80
75
70
65
60
55
50
45
40
35
30
25
20
15
f1 (ppm)
Cl
N
CO2Et

1i'

zpc-1-13 H

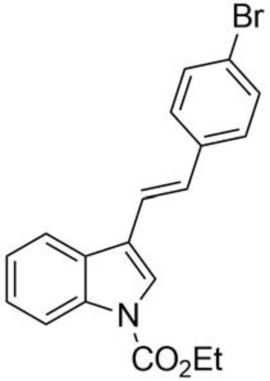
Br
N
CO2Et

1h'

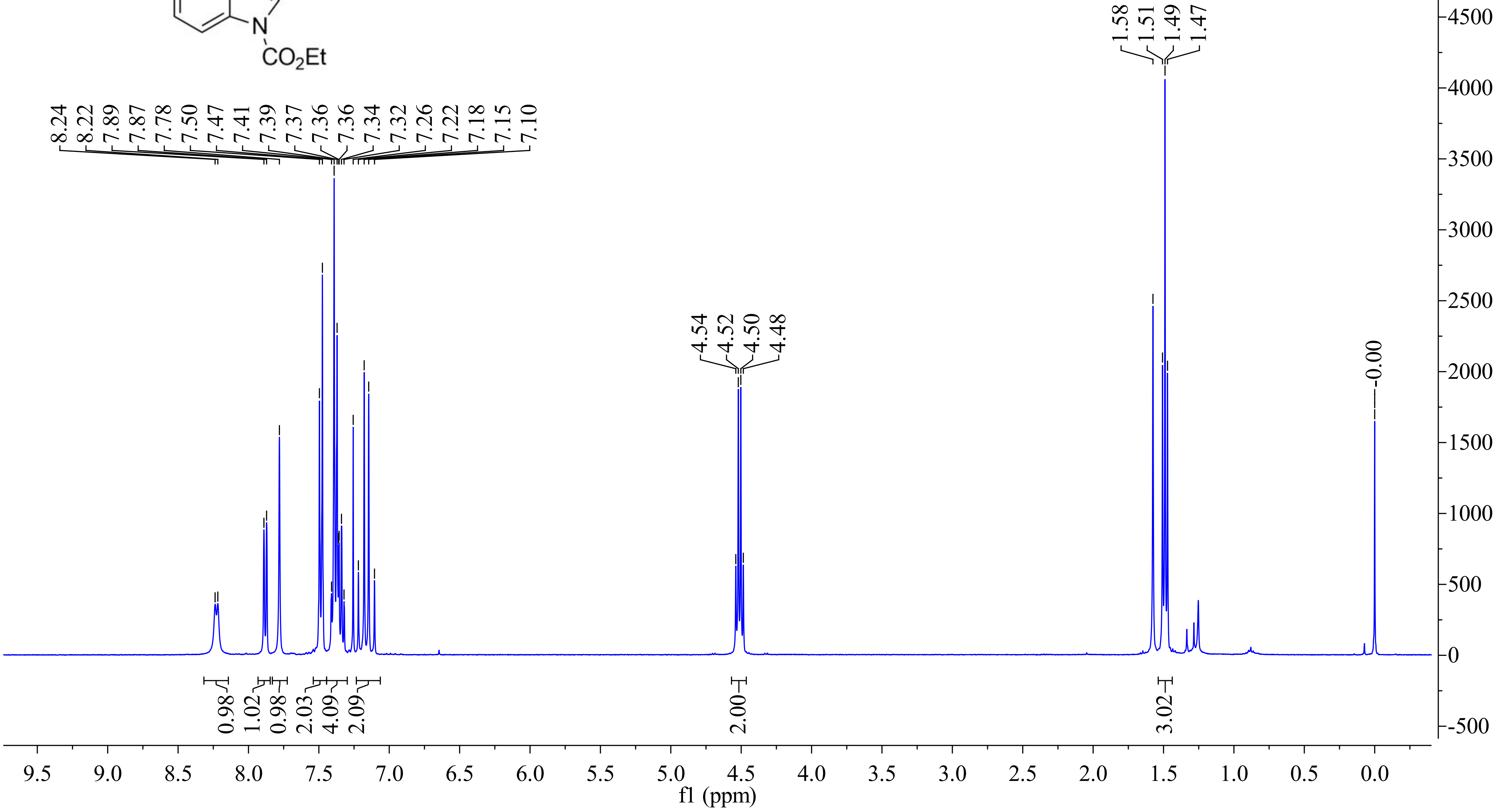
8.24 8.22 7.89 7.87 7.78 7.50 7.47 7.41 7.39 7.37 7.36 7.36 7.34 7.32 7.26 7.22 7.18 7.15 7.10
4.54 4.52 4.50 4.48
1.58 1.51 1.49 1.47
-0.00
0.98 1.02 0.98 2.03 4.09 2.09
2.00
3.02
9.5 9.0 8.5 8.0 7.5 7.0 6.5 6.0 5.5 5.0 4.5 4.0 3.5 3.0 2.5 2.0 1.5 1.0 0.5 0.0
f1 (ppm)
6000 5500 5000 4500 4000 3500 3000 2500 2000 1500 1000 500 0 -500

zpc-1-13 C

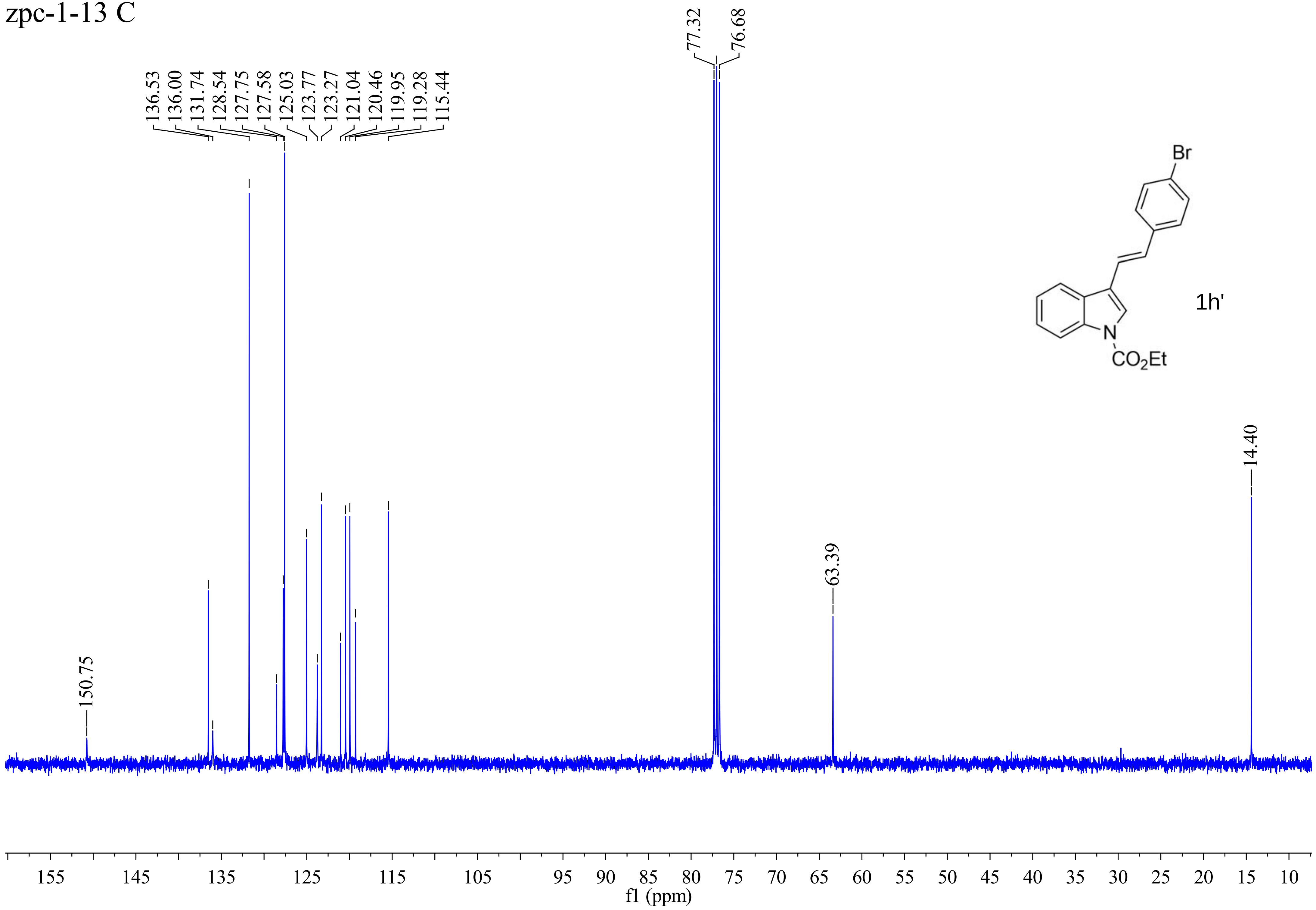

136.53
136.00
131.74
128.54
127.75
127.58
125.03
123.77
123.27
121.04
120.46
119.95
119.28
115.44
77.32
76.68
150.75
63.39
14.40
Br
N
CO2Et
1h'
155 145 135 125 115 105 95 90 85 80 75 70 65 60 55 50 45 40 35 30 25 20 15 10
f1 (ppm)

zpc-1-18 H

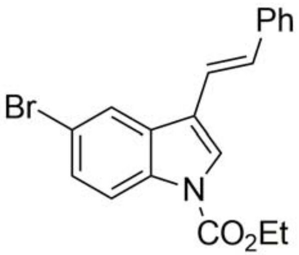
Ph
Br
N
CO2Et

1o'

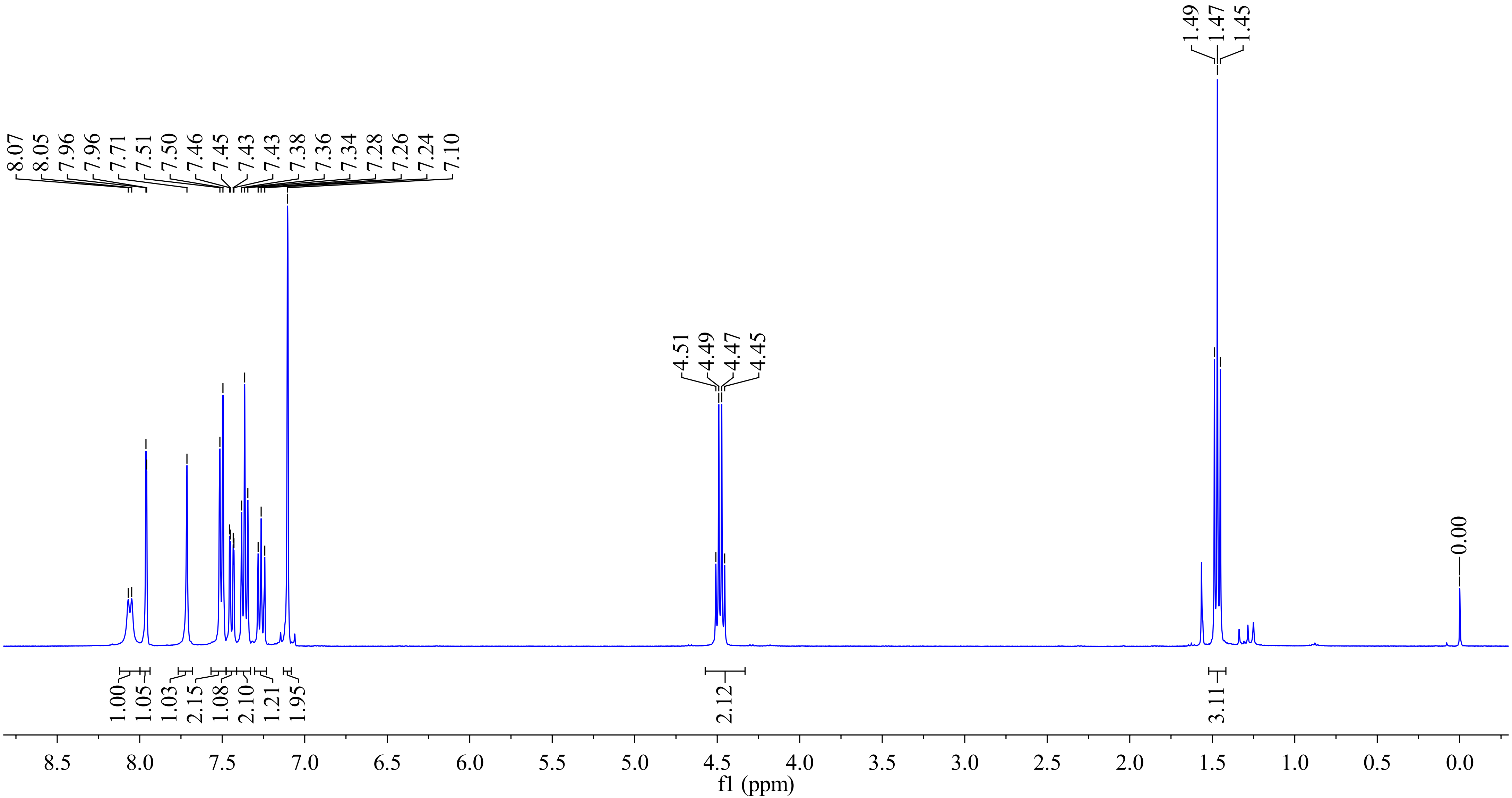
8.07
8.05
7.96
7.96
7.71
7.51
7.50
7.46
7.45
7.45
7.43
7.43
7.38
7.36
7.34
7.28
7.26
7.24
7.10
4.51
4.49
4.47
4.45
1.49
1.47
1.45
0.00
1.00
1.05
1.03
2.15
1.08
2.10
1.21
1.95
2.12
3.11
8.5
8.0
7.5
7.0
6.5
6.0
5.5
5.0
4.5
4.0
3.5
3.0
2.5
2.0
1.5
1.0
0.5
0.0
f1 (ppm)

zpc-1-18 C

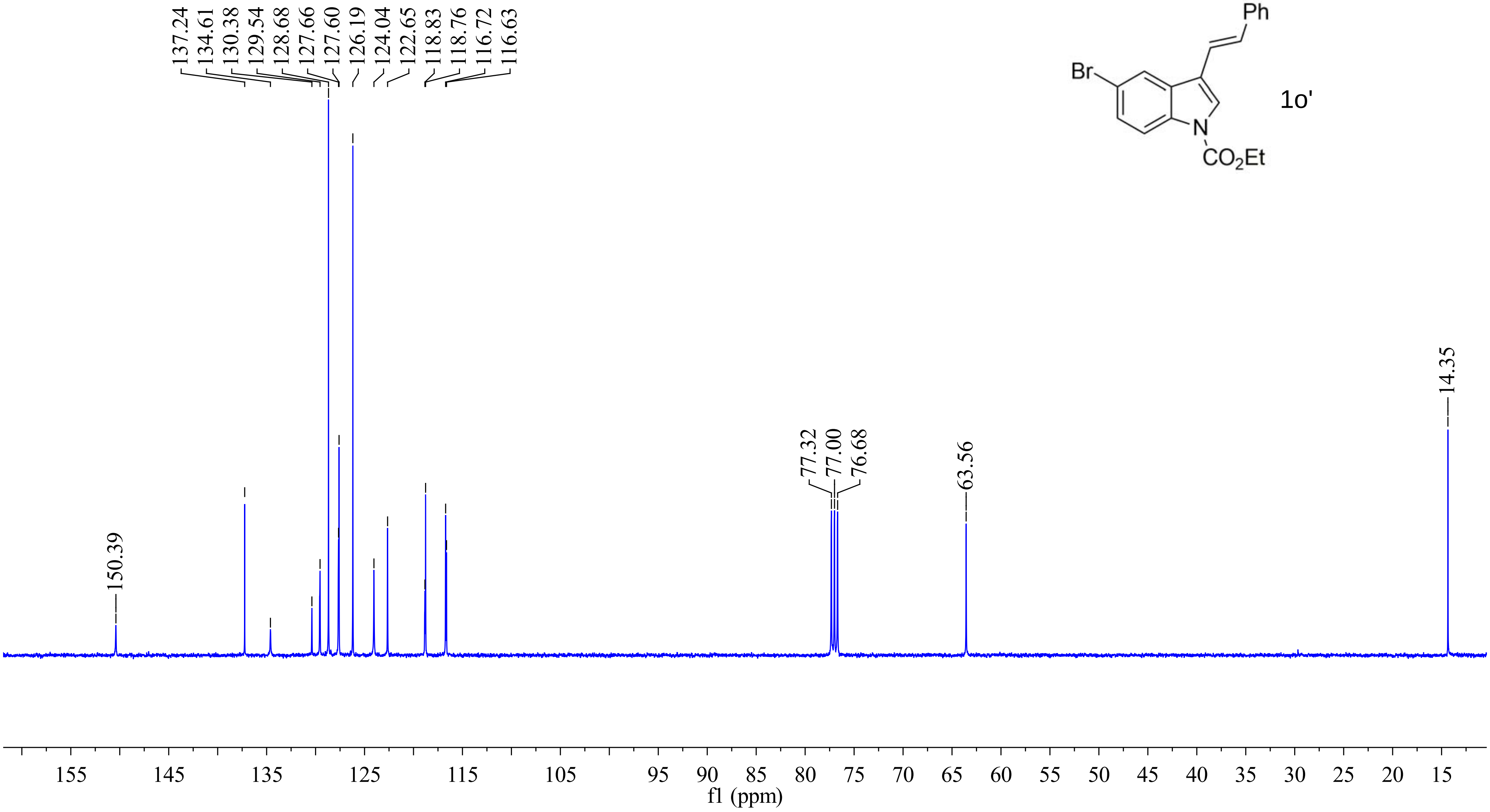
137.24
134.61
130.38
129.54
128.68
127.66
127.60
126.19
124.04
122.65
118.83
118.76
116.72
116.63
150.39
77.32
77.00
76.68
63.56
14.35
Ph
Br
N
CO_2Et
1o'
155 145 135 125 115 105 95 90 85 80 75 70 65 60 55 50 45 40 35 30 25 20 15
f1 (ppm)

wyd-6-65 H

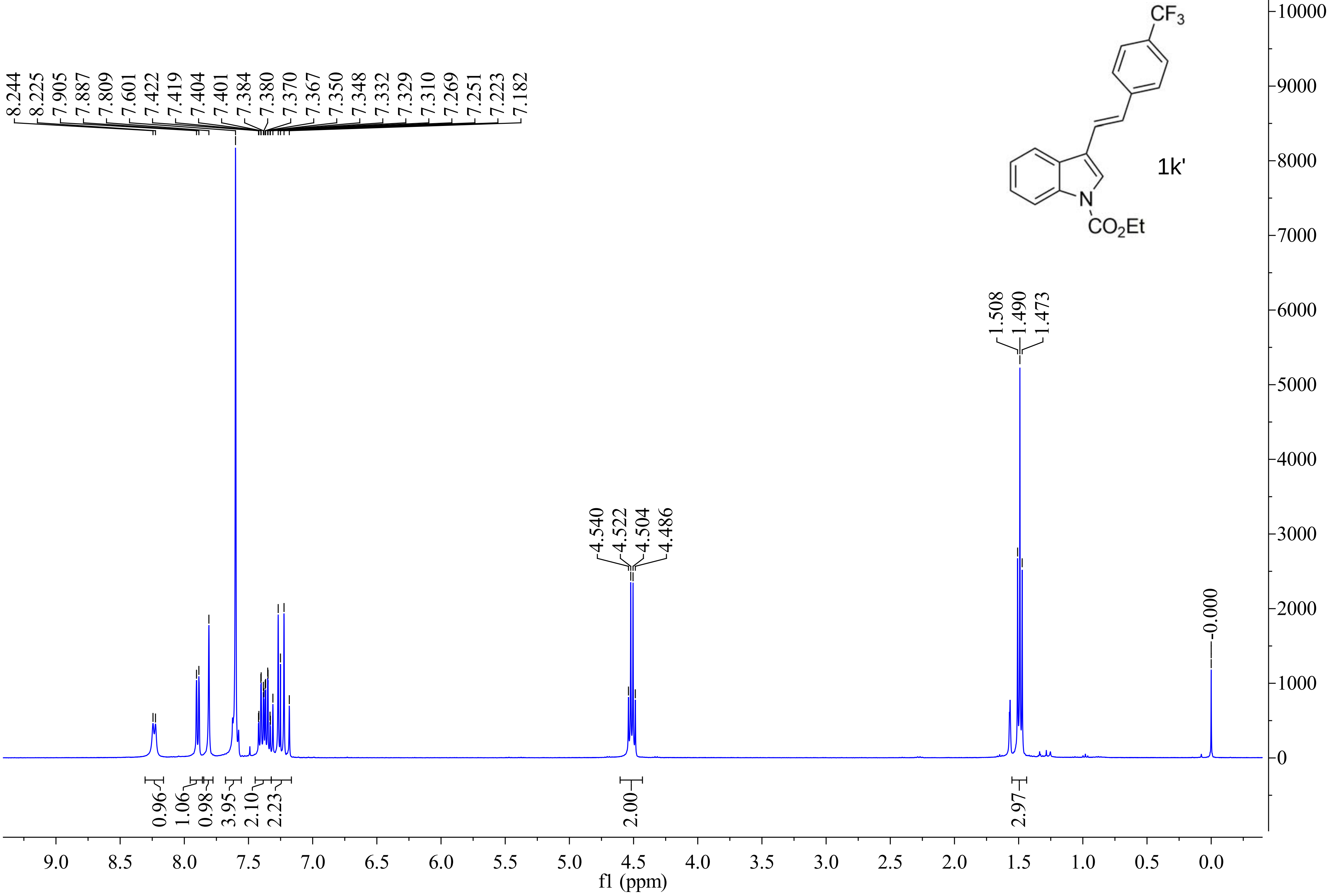

8.244
8.225
7.905
7.887
7.809
7.601
7.422
7.419
7.404
7.401
7.384
7.380
7.370
7.367
7.350
7.348
7.332
7.329
7.310
7.269
7.251
7.223
7.182
4.540
4.522
4.504
4.486
1.508
1.490
1.473
-0.000
0.96
1.06
0.98
3.95
2.10
2.23
2.00
2.97
CF3
N
CO2Et
1k'
f1 (ppm)

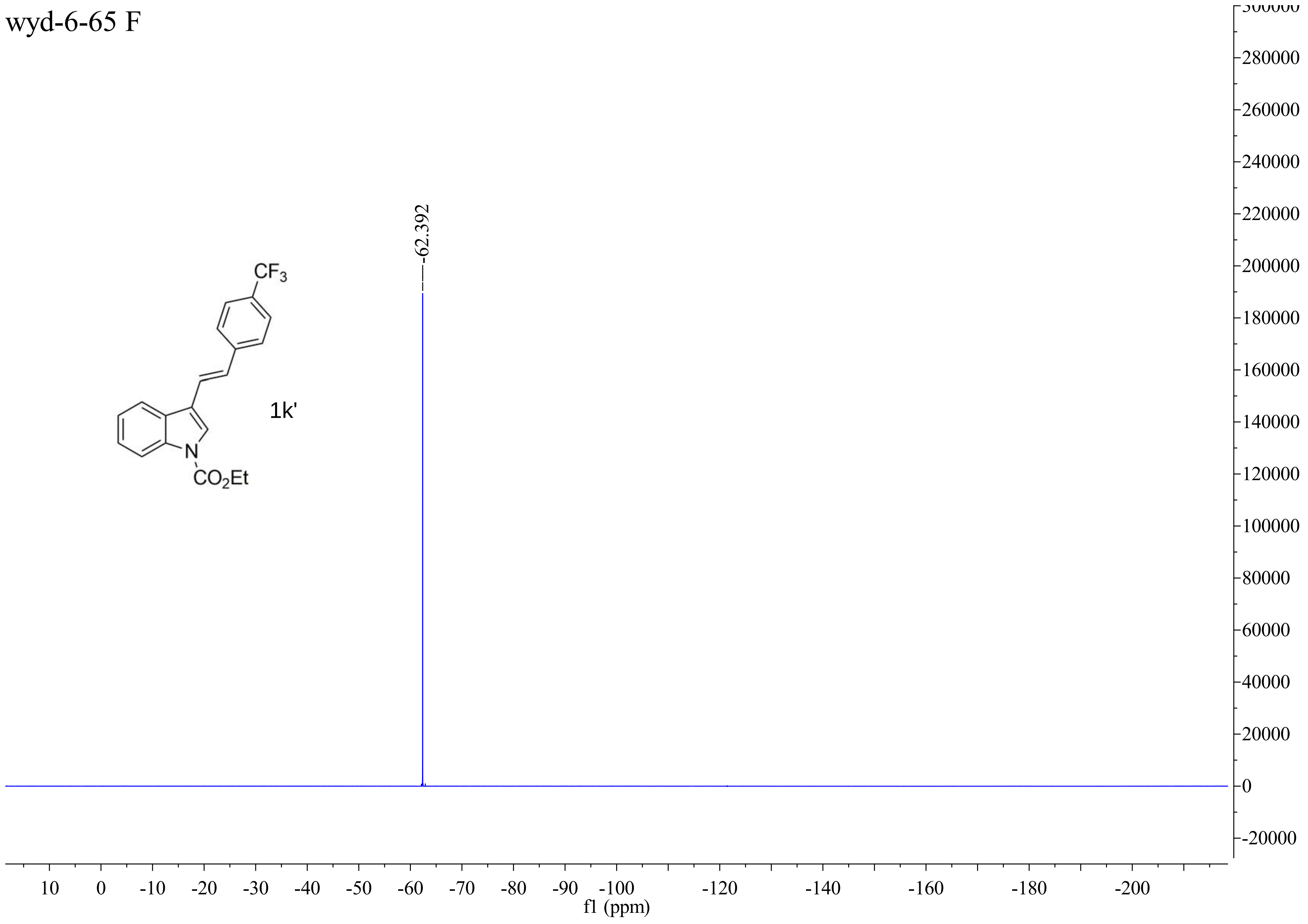

wyd-6-65 F
-62.392
CF3
N
CO2Et
1k'
f1 (ppm)
10
0
-10
-20
-30
-40
-50
-60
-70
-80
-90
-100
-120
-140
-160
-180
-200
300000
280000
260000
240000
220000
200000
180000
160000
140000
120000
100000
80000
60000
40000
20000
0
-20000

wyd-6-65 C

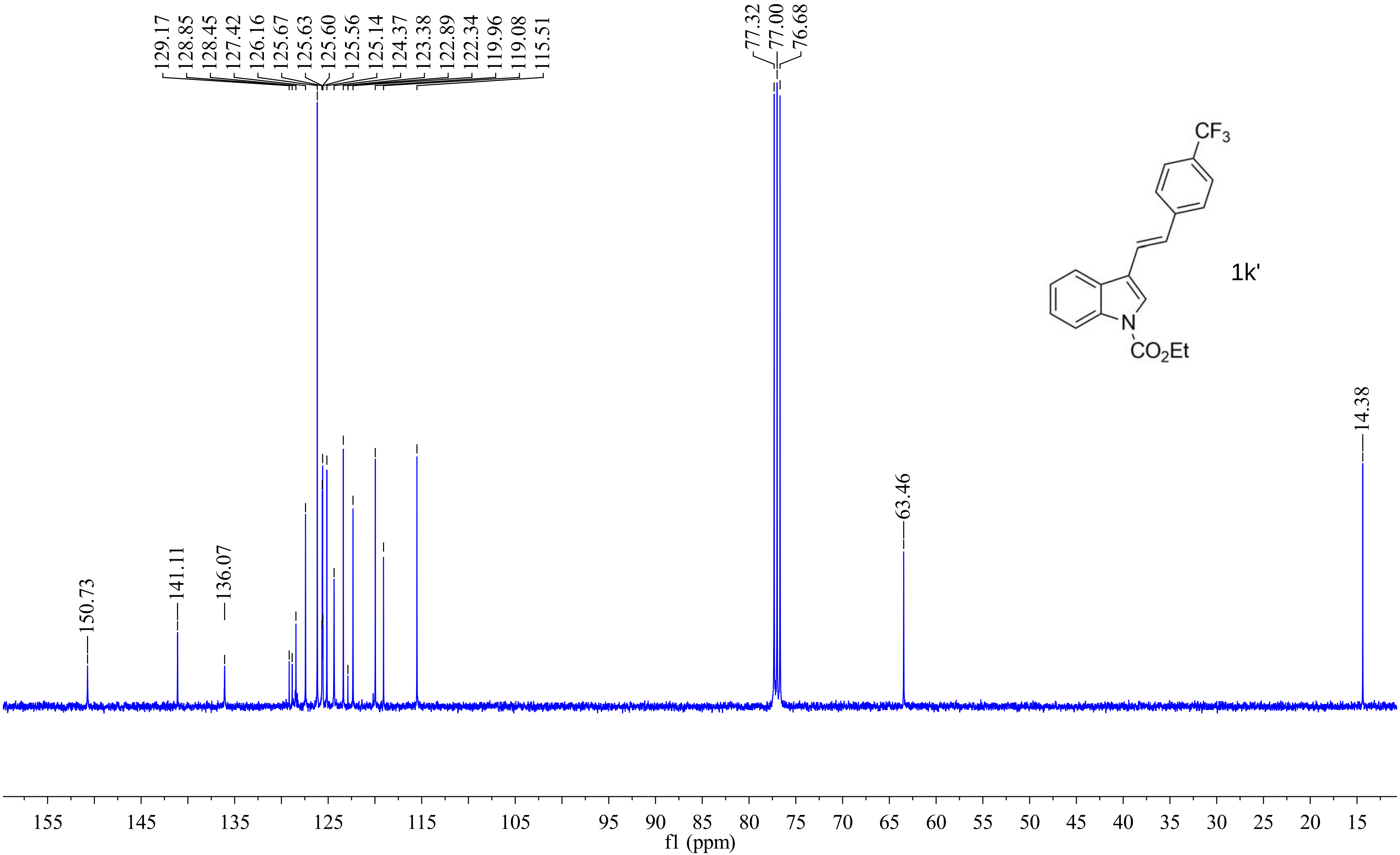
129.17
128.85
128.45
127.42
126.16
125.67
125.63
125.60
125.56
125.14
124.37
123.38
122.89
122.34
119.96
119.08
115.51
77.32
77.00
76.68
150.73
141.11
136.07
63.46
14.38
CF3
N
CO2Et
1k'
155
145
135
125
115
105
95
90
85
80
75
70
65
60
55
50
45
40
35
30
25
20
15
f1 (ppm)

wyd-6-66 H

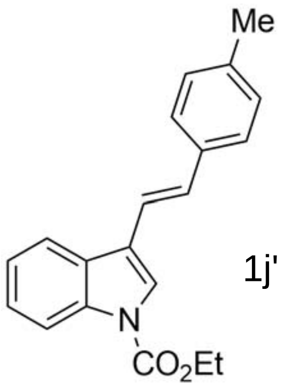
Me
N
CO2Et

1j'

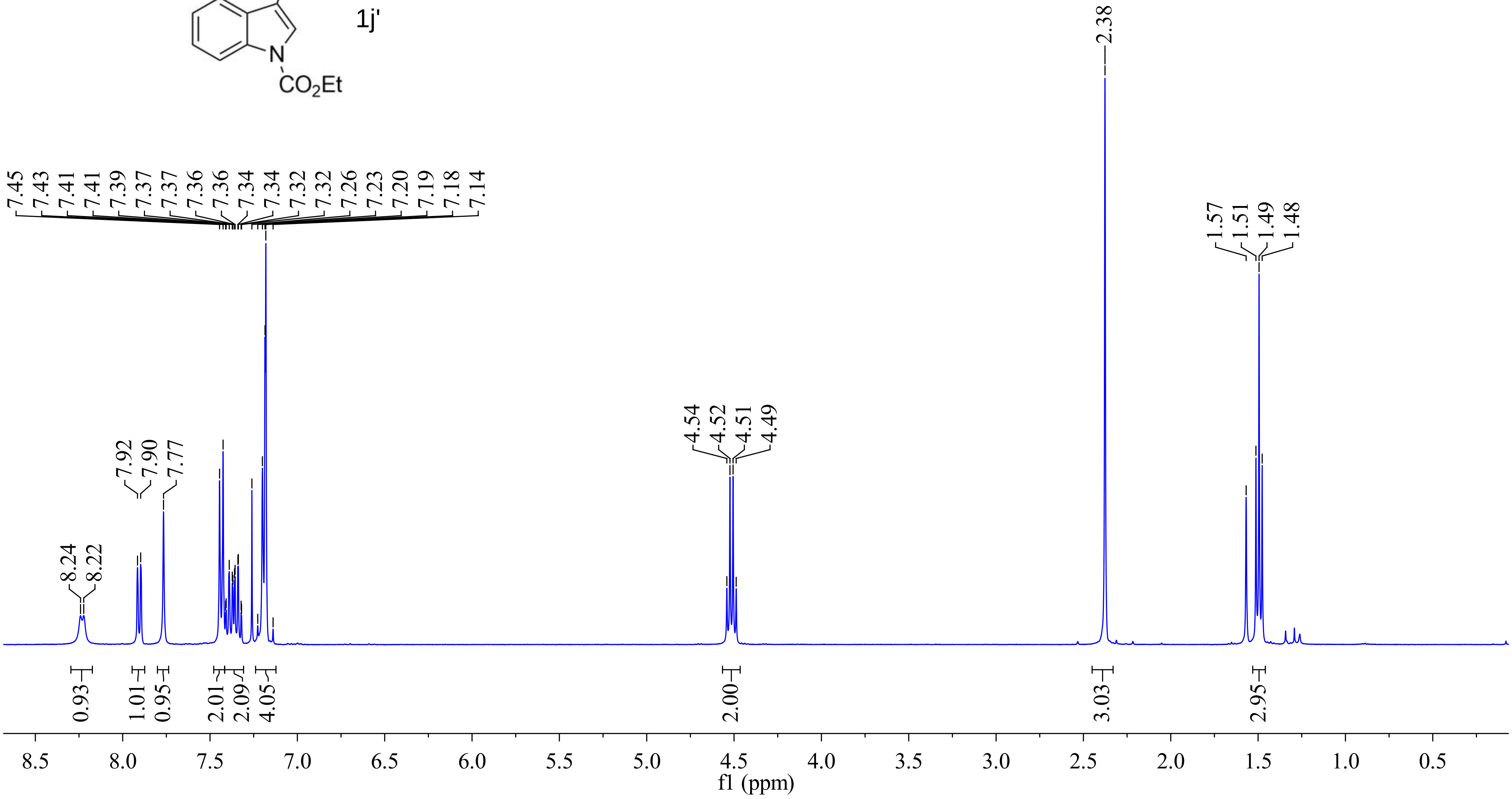
2.38
7.45 7.43 7.41 7.41 7.39 7.37 7.37 7.36 7.36 7.34 7.34 7.32 7.32 7.26 7.23 7.20 7.19 7.18 7.14
1.57 1.51 1.49 1.48
4.54 4.52 4.51 4.49
7.92 7.90 7.77
8.24 8.22
0.93 1.01 0.95 2.01 2.09 4.05 2.00 3.03 2.95
8.5 8.0 7.5 7.0 6.5 6.0 5.5 5.0 4.5 4.0 3.5 3.0 2.5 2.0 1.5 1.0 0.5
f1 (ppm)

wyd-6-66 C

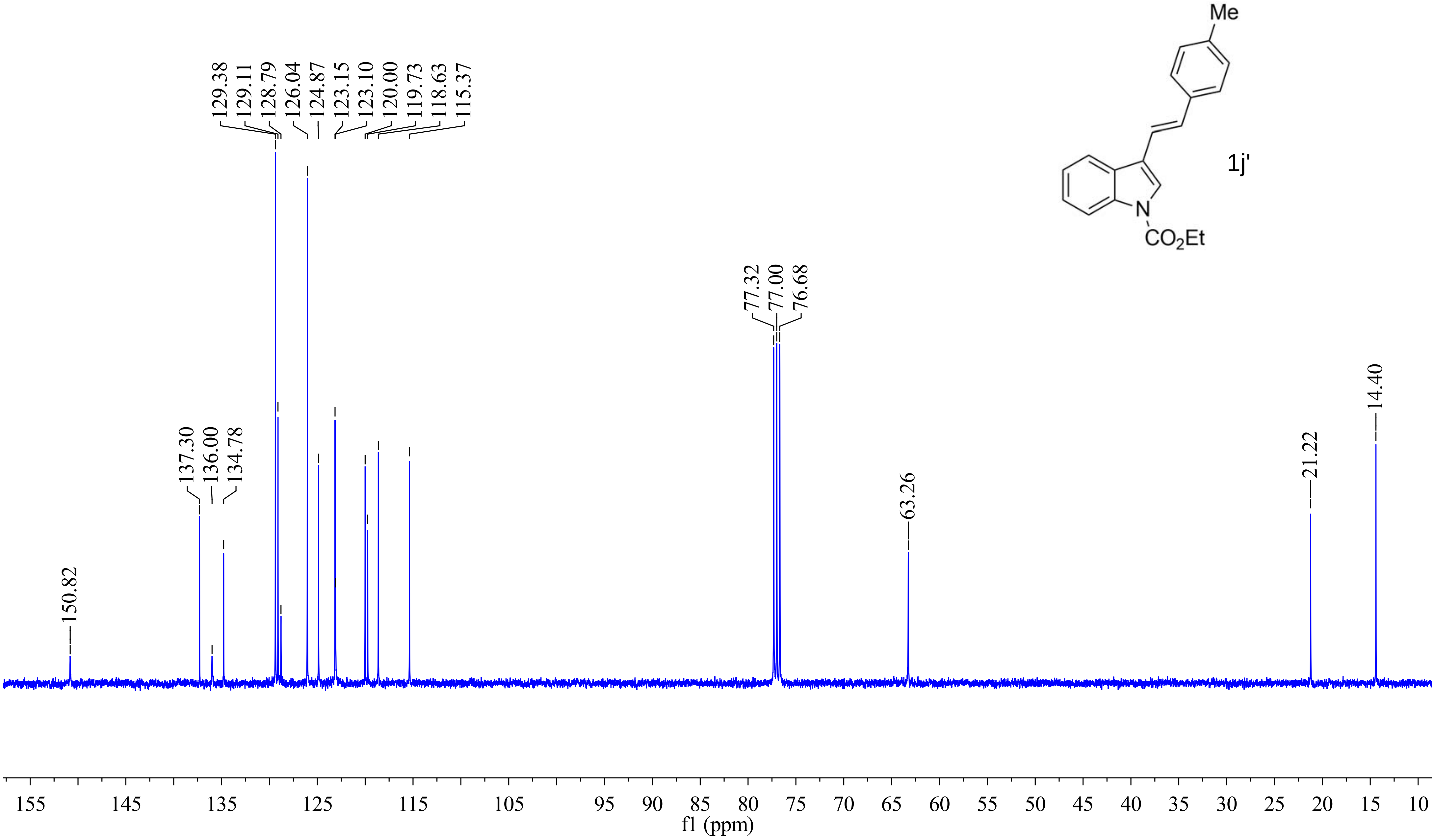

Me
1j'
N
CO2Et
129.38
129.11
128.79
126.04
124.87
123.15
123.10
120.00
119.73
118.63
115.37
77.32
77.00
76.68
150.82
137.30
136.00
134.78
63.26
21.22
14.40
155
145
135
125
115
105
95
90
85
80
75
70
65
60
55
50
45
40
35
30
25
20
15
10
f1 (ppm)

zpc-1-59 H

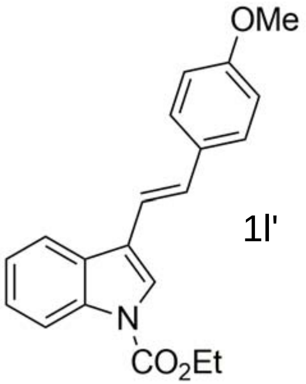

OMe
N
CO2Et

1l'

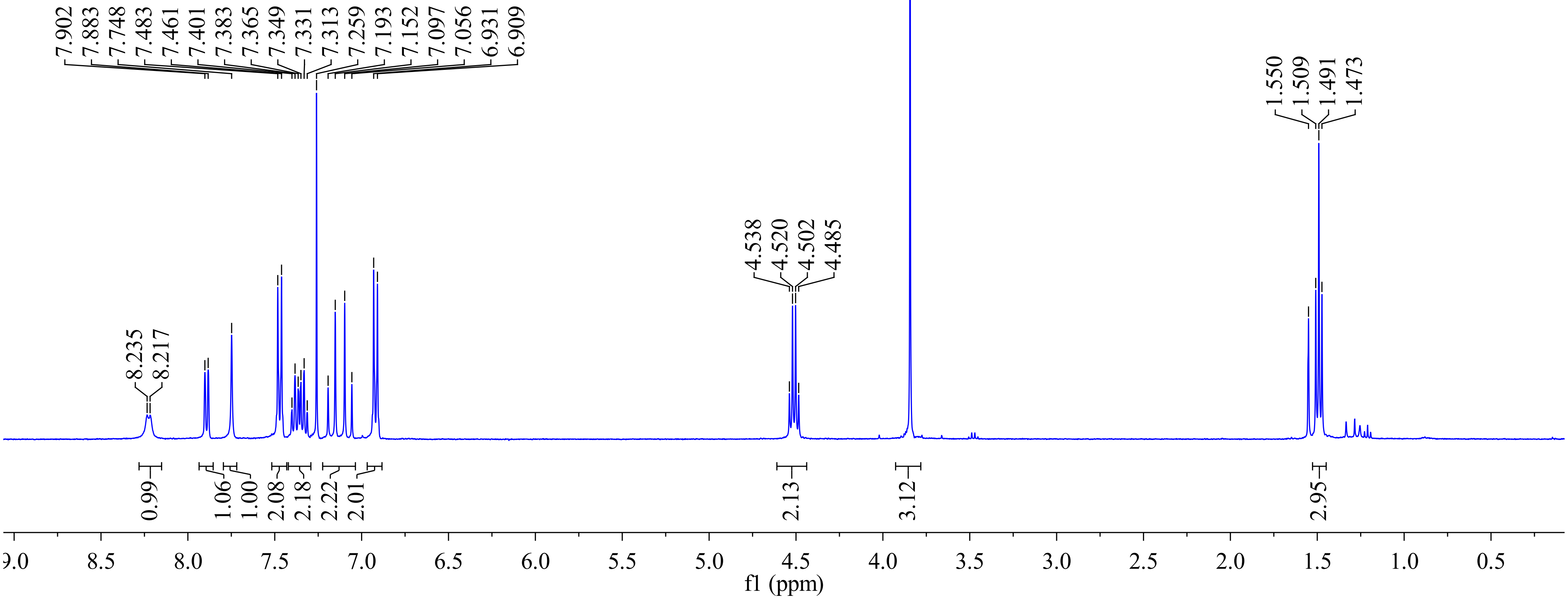

7.902
7.883
7.748
7.483
7.461
7.401
7.383
7.365
7.349
7.331
7.313
7.259
7.193
7.152
7.097
7.056
6.931
6.909
3.844
1.550
1.509
1.491
1.473
4.538
4.520
4.502
4.485
8.235
8.217
0.99
1.06
1.00
2.08
2.18
2.22
2.01
2.13
3.12
2.95
9.0
8.5
8.0
7.5
7.0
6.5
6.0
5.5
5.0
4.5
4.0
3.5
3.0
2.5
2.0
1.5
1.0
0.5
f1 (ppm)

zpc-1-59 C

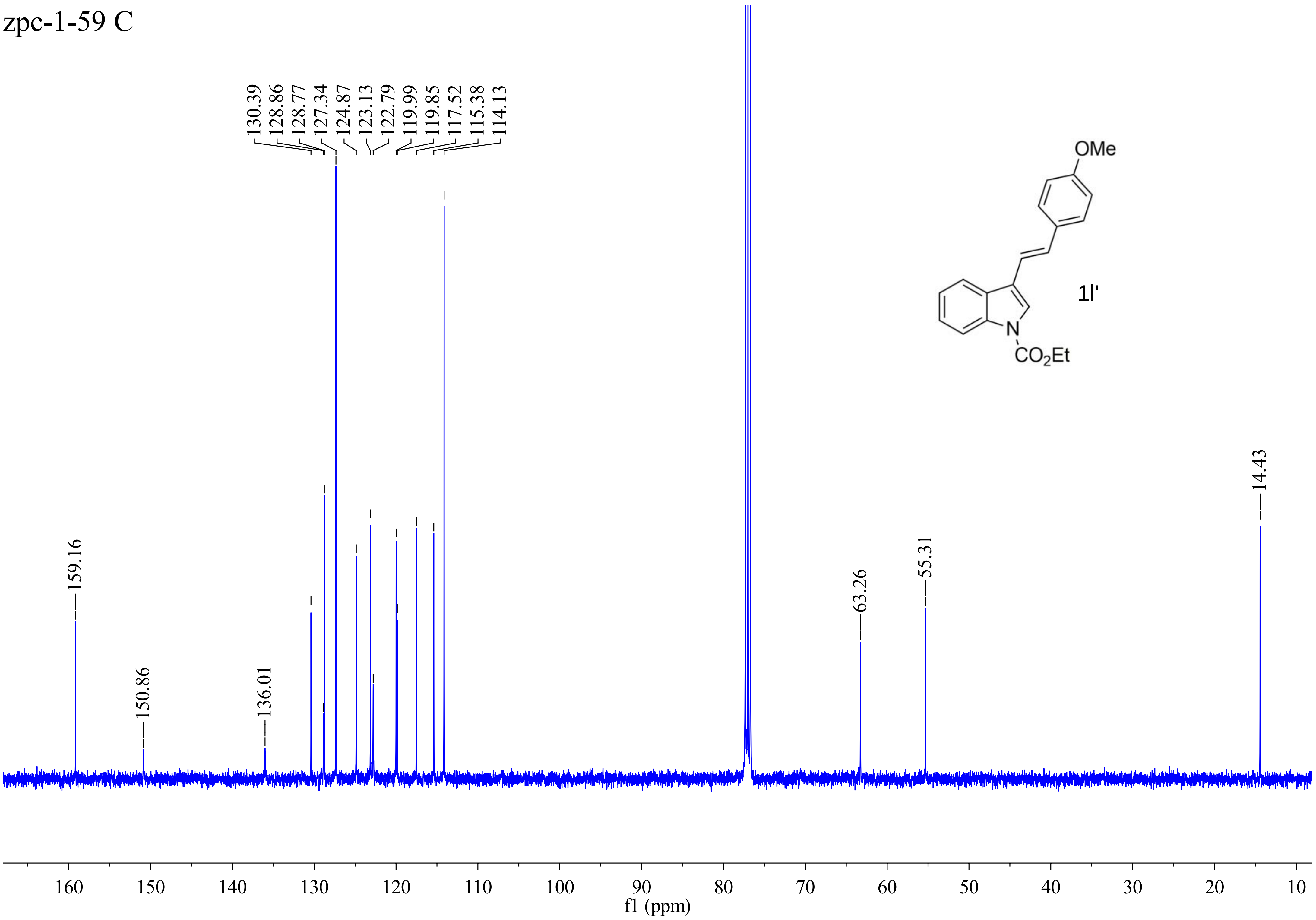
130.39
128.86
128.77
127.34
124.87
123.13
122.79
119.99
119.85
117.52
115.38
114.13
159.16
150.86
136.01
63.26
55.31
14.43
OMe
N
CO_2Et
1l'
160 150 140 130 120 110 100 90 80 70 60 50 40 30 20 10
f1 (ppm)

wyd-6-73 H

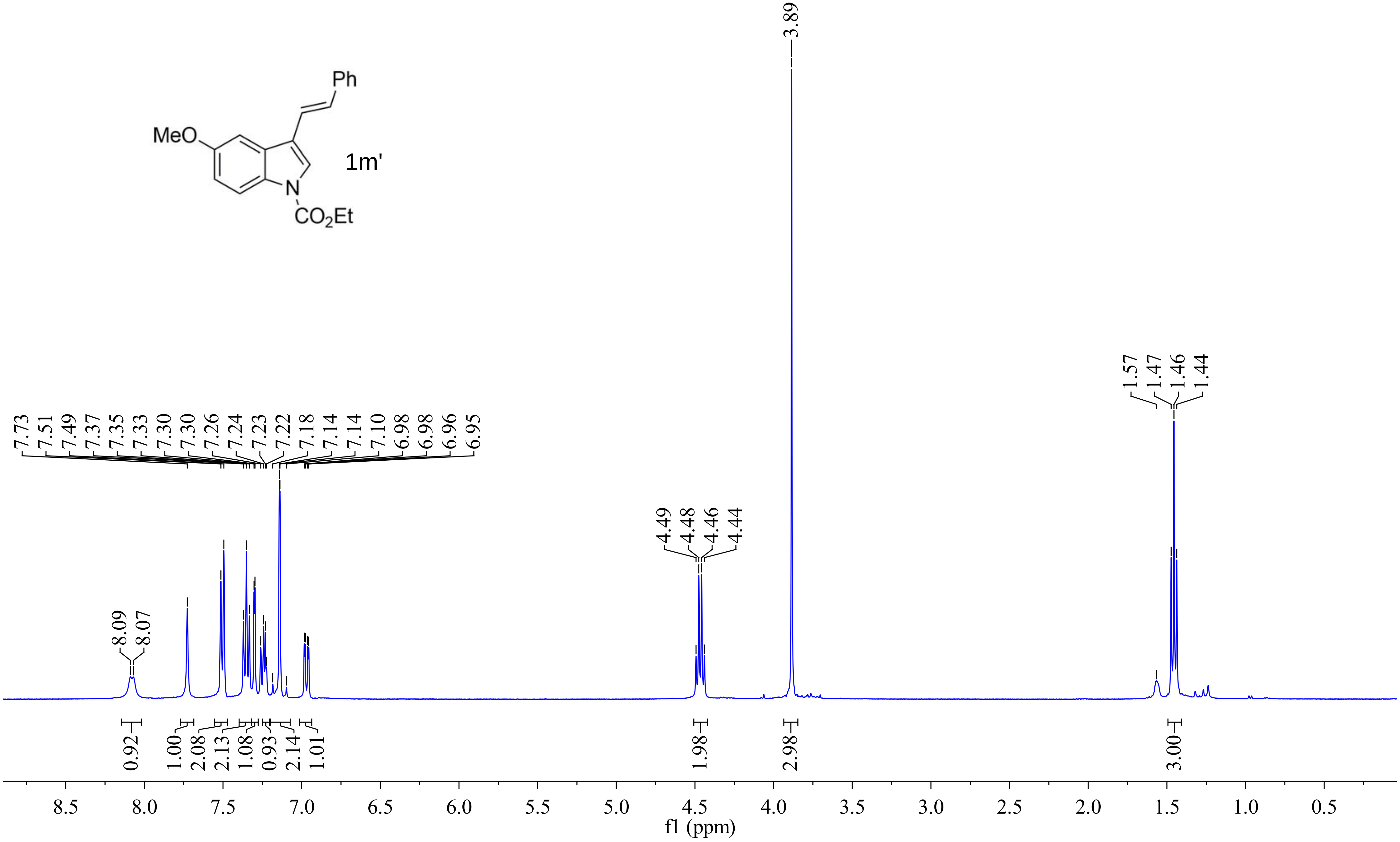
Ph
MeO
N
CO2Et
1m'
3.89
1.57 1.47 1.46 1.44
7.73 7.51 7.49 7.37 7.35 7.33 7.30 7.30 7.26 7.24 7.23 7.22 7.18 7.14 7.14 7.10 6.98 6.98 6.96 6.95
4.49 4.48 4.46 4.44
8.09 8.07
0.92 1.00 2.08 2.13 1.08 0.93 2.14 1.01
1.98
2.98
3.00
8.5 8.0 7.5 7.0 6.5 6.0 5.5 5.0 4.5 4.0 3.5 3.0 2.5 2.0 1.5 1.0 0.5
f1 (ppm)

wyd-6-73 C

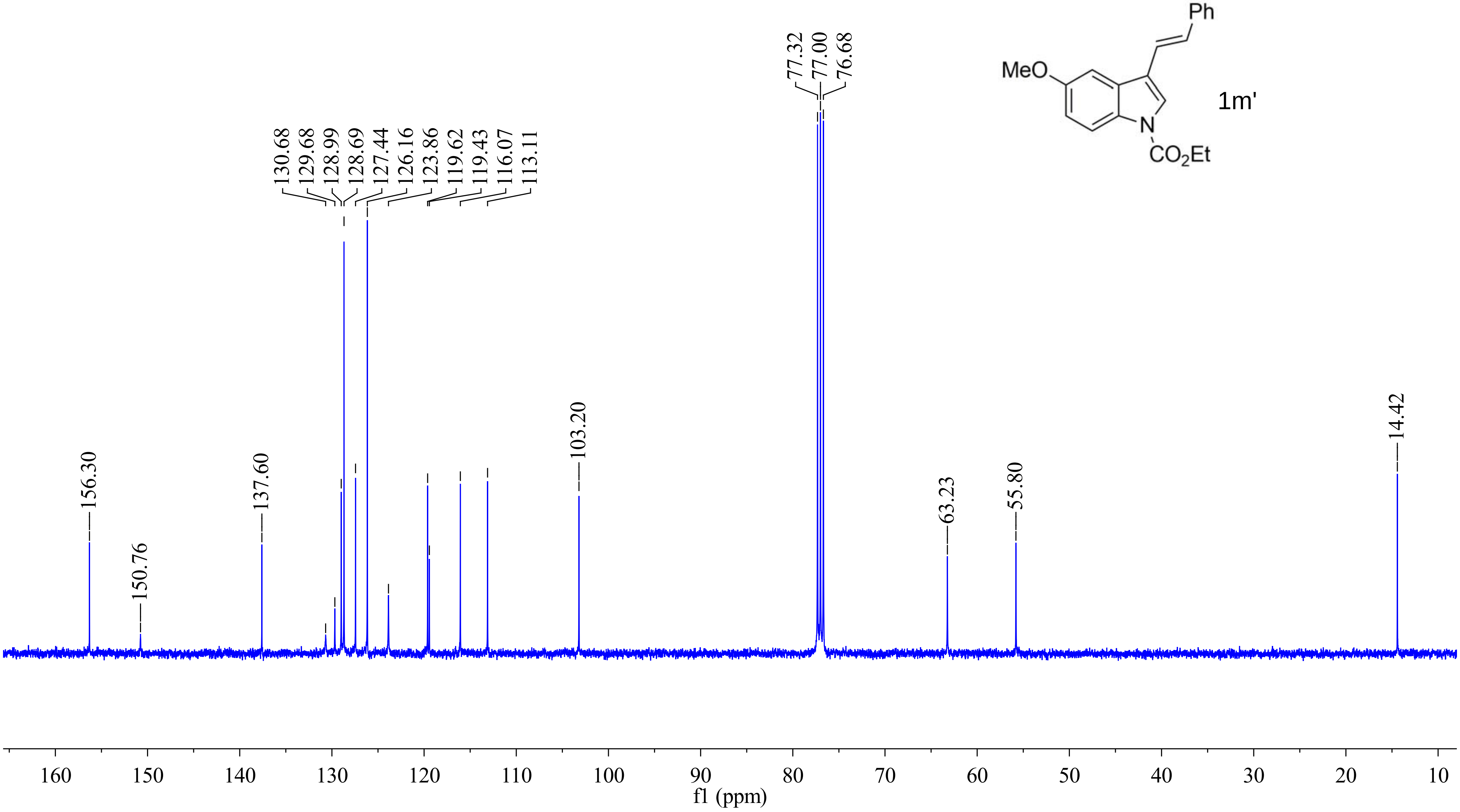

MeO
Ph
N
CO_2Et
1m'
156.30
150.76
137.60
130.68
129.68
128.99
128.69
127.44
126.16
123.86
119.62
119.43
116.07
113.11
103.20
77.32
77.00
76.68
63.23
55.80
14.42
160
150
140
130
120
110
100
90
80
70
60
50
40
30
20
10
f1 (ppm)

zpc-1-75-2 H

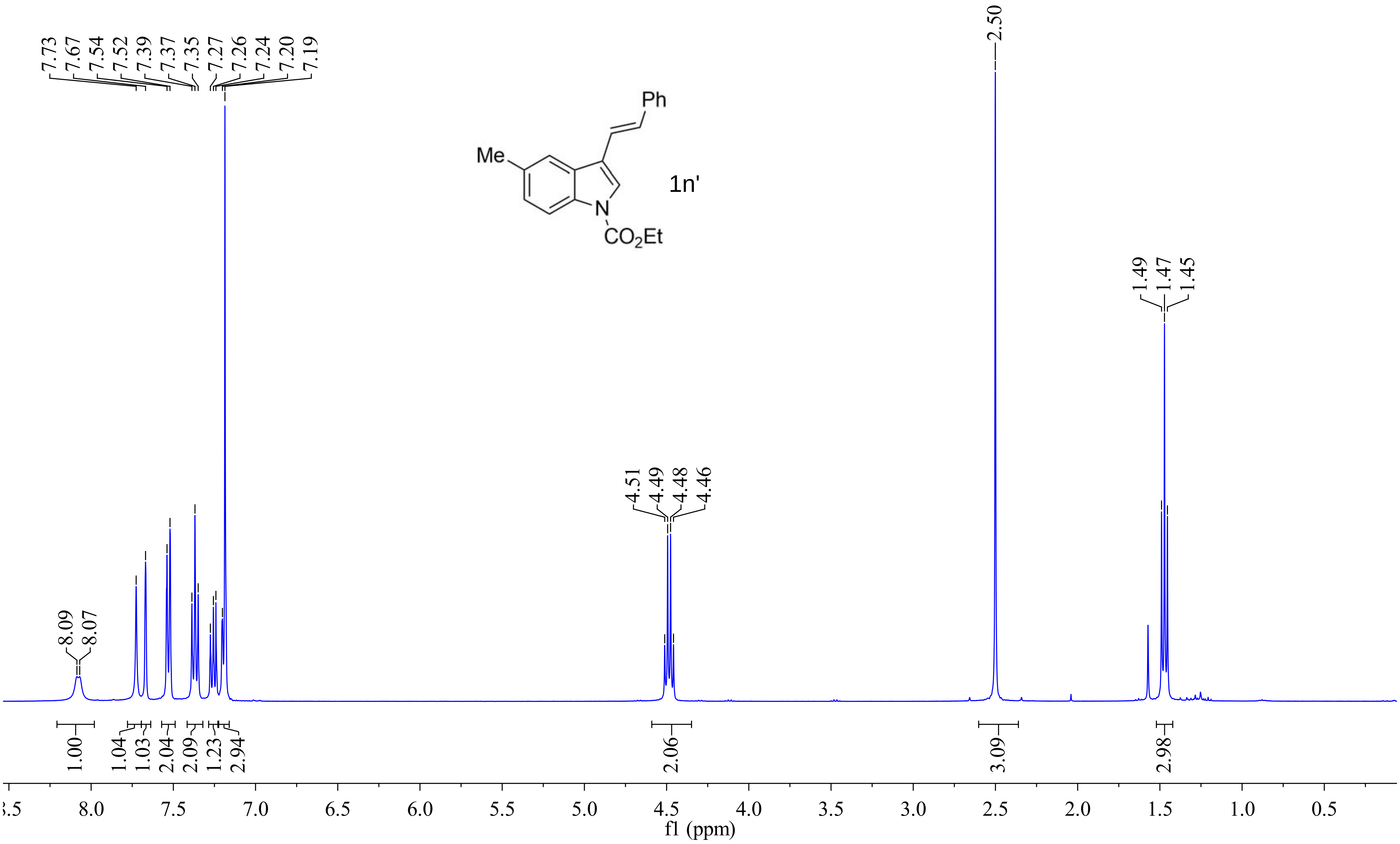
7.73
7.67
7.54
7.52
7.39
7.37
7.35
7.27
7.26
7.24
7.20
7.19
2.50
Ph
Me
N
CO2Et
1n'
1.49
1.47
1.45
4.51
4.49
4.48
4.46
8.09
8.07
1.00
1.04
1.03
2.04
2.09
1.23
2.94
2.06
3.09
2.98
8.0
7.5
7.0
6.5
6.0
5.5
5.0
4.5
4.0
3.5
3.0
2.5
2.0
1.5
1.0
0.5
f1 (ppm)

zpc-1-75-2 C

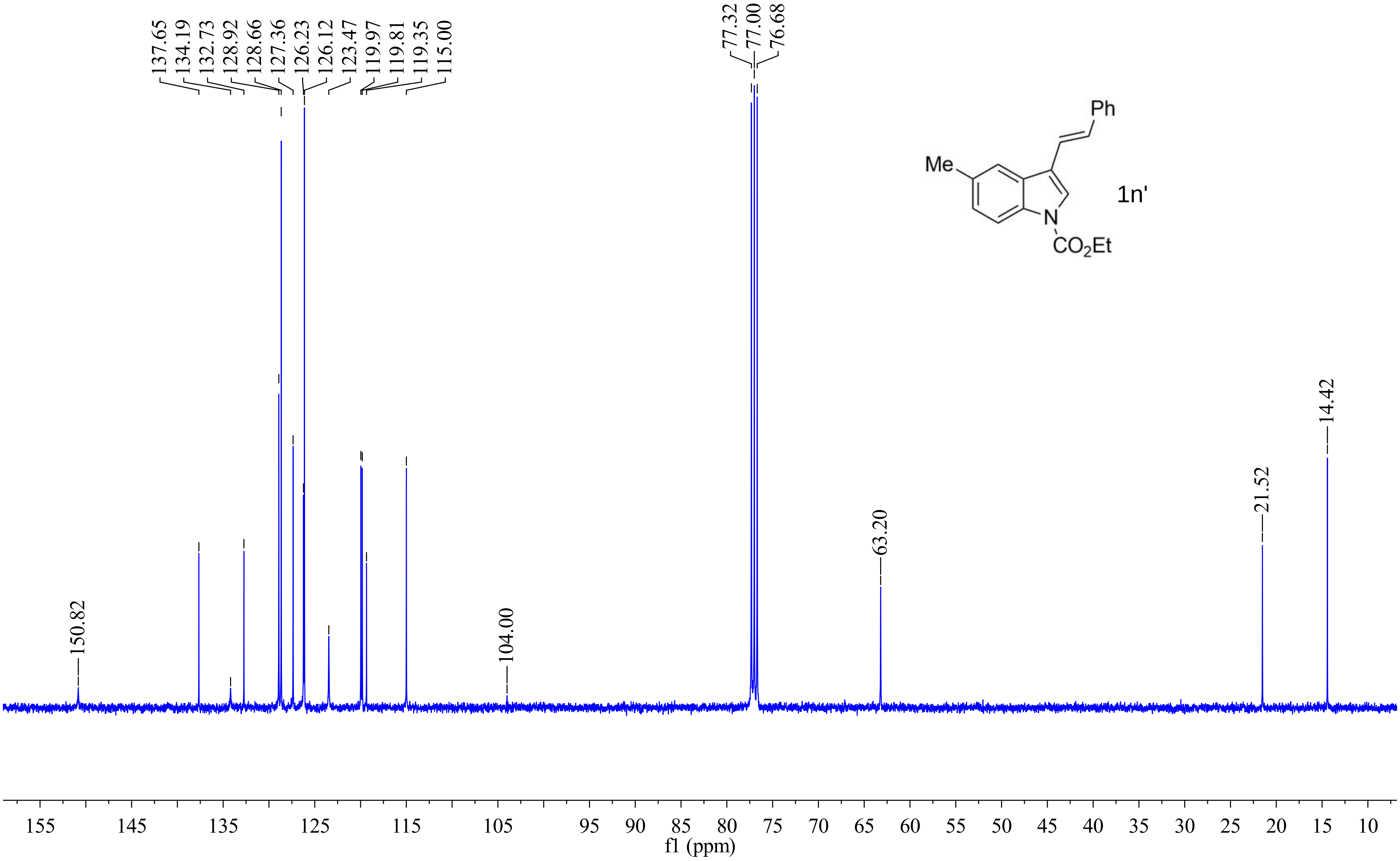
137.65
134.19
132.73
128.92
128.66
127.36
126.23
126.12
123.47
119.97
119.81
119.35
115.00
77.32
77.00
76.68
150.82
104.00
63.20
21.52
14.42
Ph
Me
N
CO2Et
1n'
155 145 135 125 115 105 95 90 85 80 75 70 65 60 55 50 45 40 35 30 25 20 15 10
f1 (ppm)

wyd-6-72 H

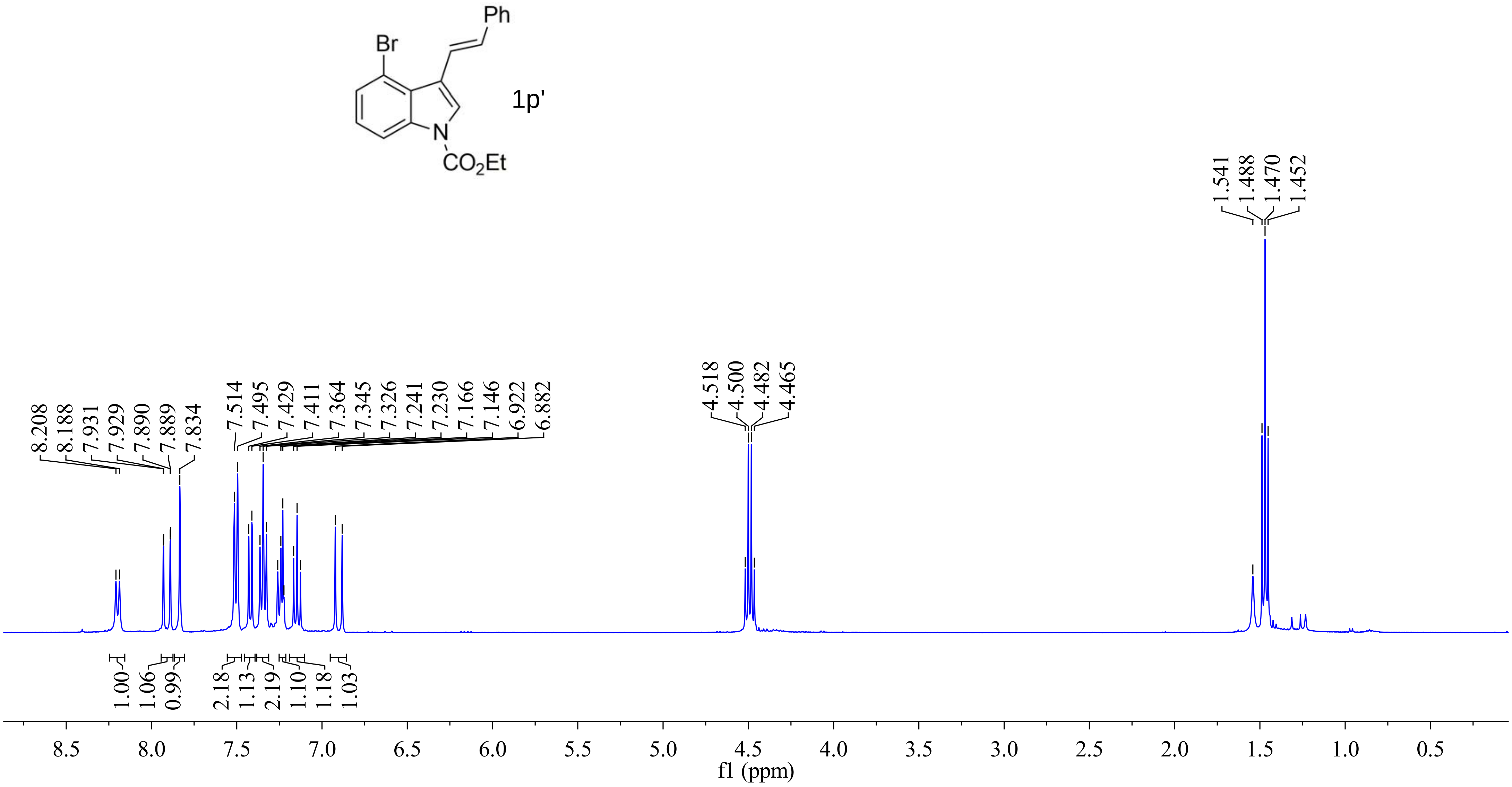
Br
Ph
N
CO_2Et
1p'
8.208
8.188
7.931
7.929
7.890
7.889
7.834
7.514
7.495
7.429
7.411
7.364
7.345
7.326
7.241
7.230
7.166
7.146
6.922
6.882
4.518
4.500
4.482
4.465
1.541
1.488
1.470
1.452
1.00
1.06
0.99
2.18
1.13
2.19
1.10
1.18
1.03
8.5
8.0
7.5
7.0
6.5
6.0
5.5
5.0
4.5
4.0
3.5
3.0
2.5
2.0
1.5
1.0
0.5
f1 (ppm)

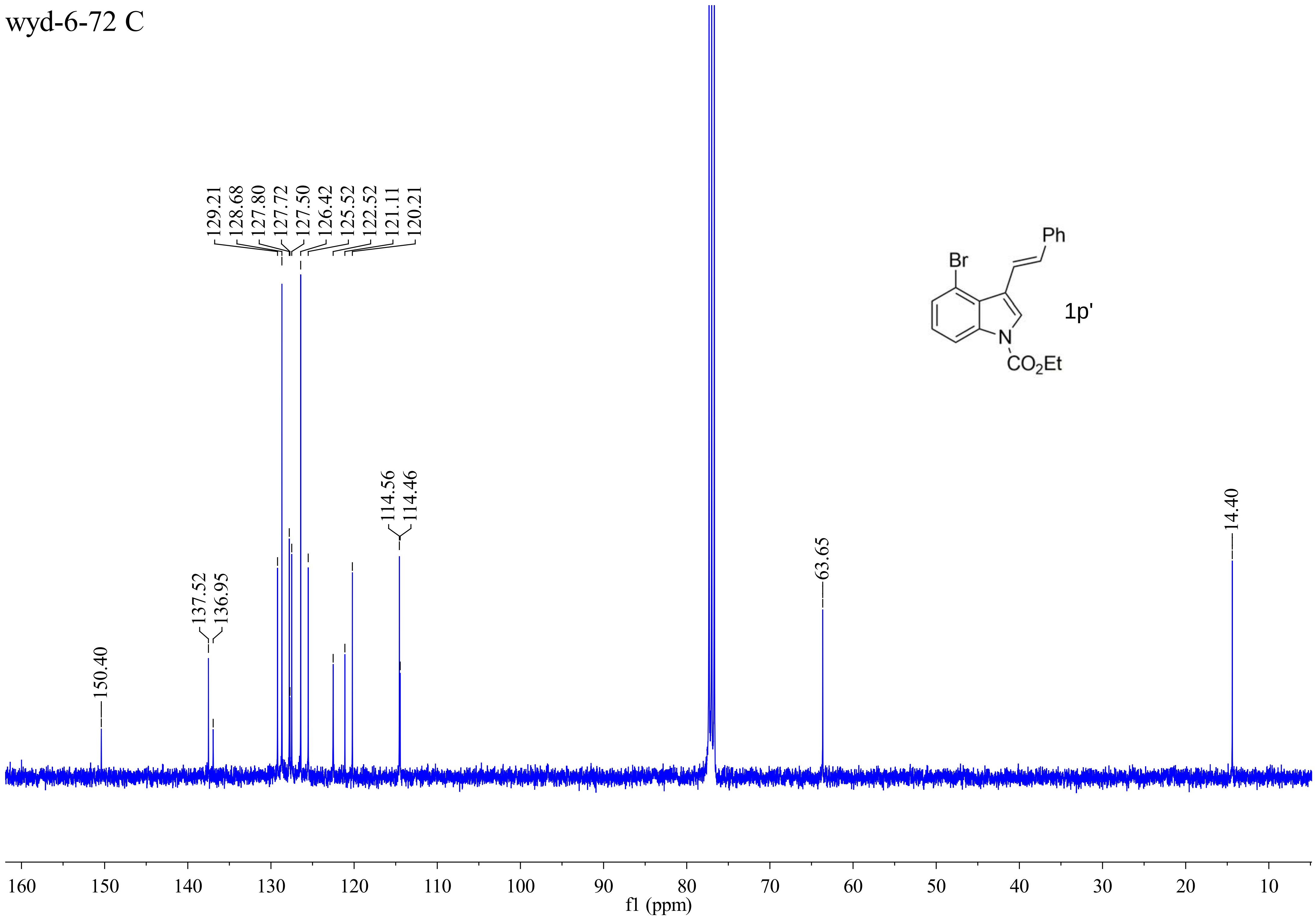
wyd-6-72 C
150.40
137.52
136.95
129.21
128.68
127.80
127.72
127.50
126.42
125.52
122.52
121.11
120.21
114.56
114.46
63.65
14.40
Br
Ph
N
CO2Et
1p'
160
150
140
130
120
110
100
90
80
70
60
50
40
30
20
10
f1 (ppm)

zpc-1-55 H

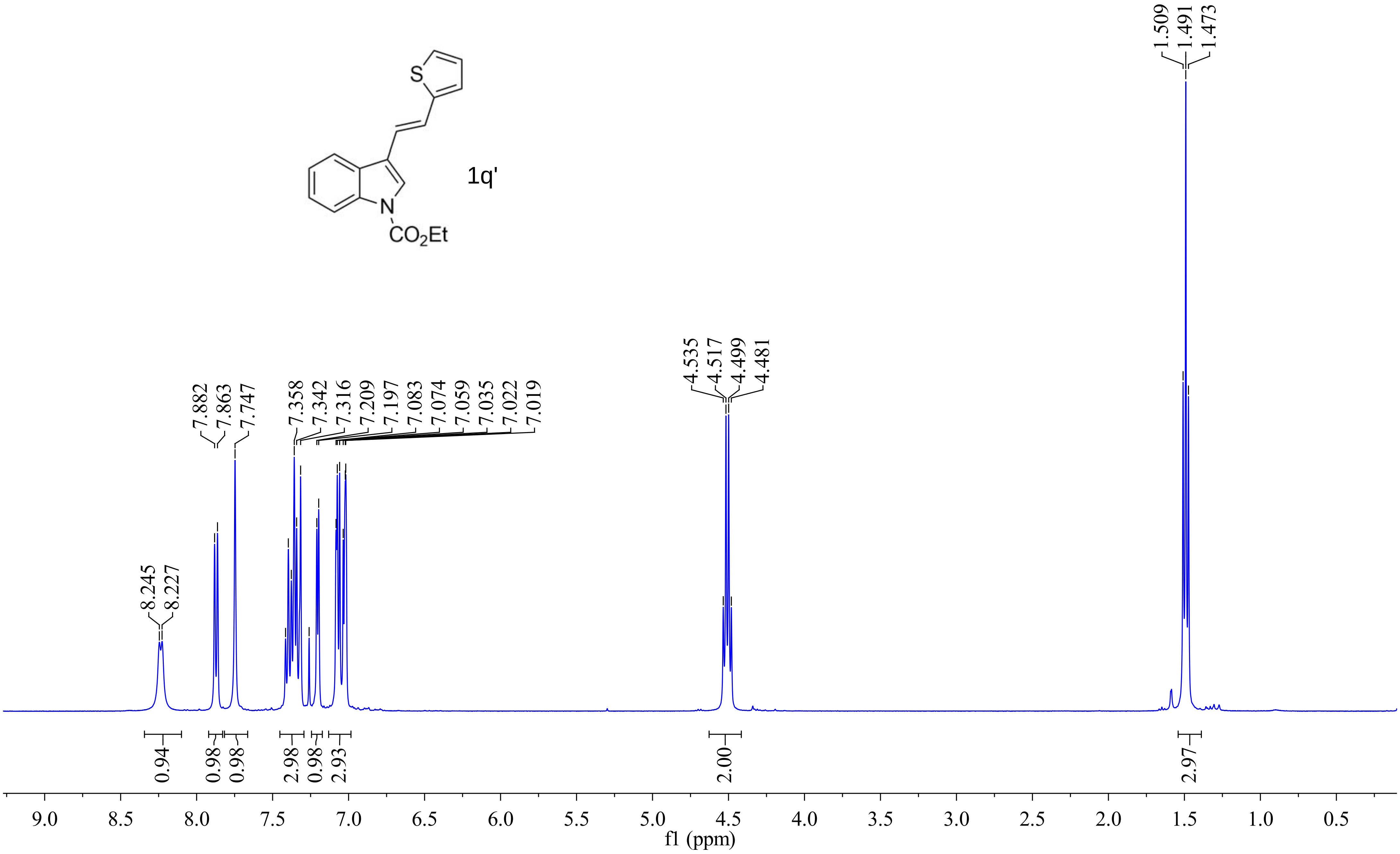

1q'
S
N
CO2Et
1.509
1.491
1.473
4.535
4.517
4.499
4.481
7.882
7.863
7.747
7.358
7.342
7.316
7.209
7.197
7.083
7.074
7.059
7.035
7.022
7.019
8.245
8.227
0.94
0.98
0.98
2.98
0.98
2.93
2.00
2.97
9.0
8.5
8.0
7.5
7.0
6.5
6.0
5.5
5.0
4.5
4.0
3.5
3.0
2.5
2.0
1.5
1.0
0.5
f1 (ppm)

zpc-1-55 C

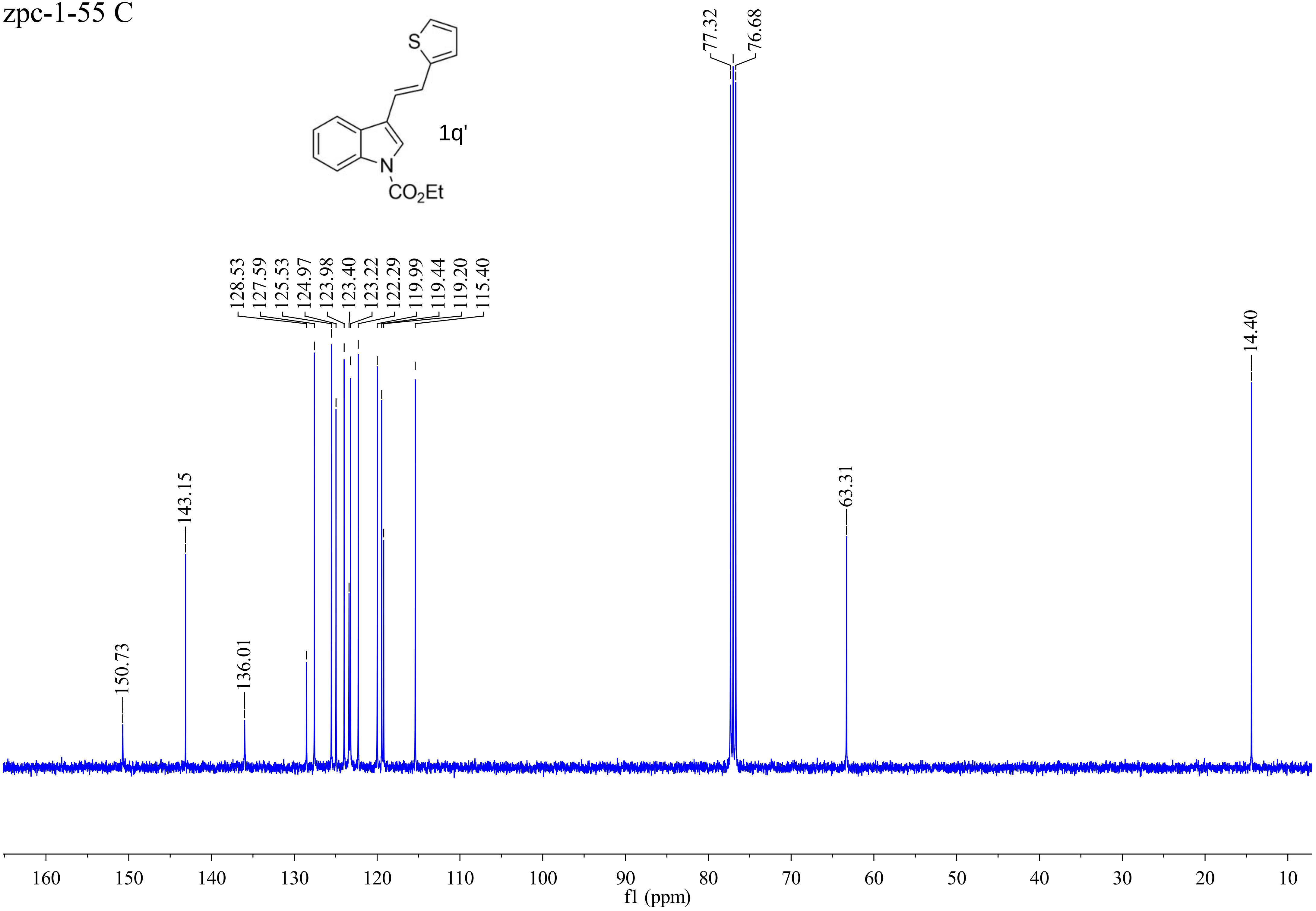

1q'
S
N
CO_2Et
77.32
76.68
128.53
127.59
125.53
124.97
123.98
123.40
123.22
122.29
119.99
119.44
119.20
115.40
14.40
143.15
63.31
150.73
136.01
160 150 140 130 120 110 100 90 80 70 60 50 40 30 20 10
f1 (ppm)

wyd-6-114-2 H

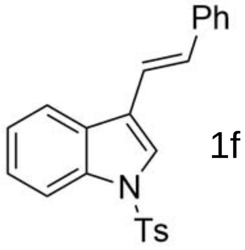

1f

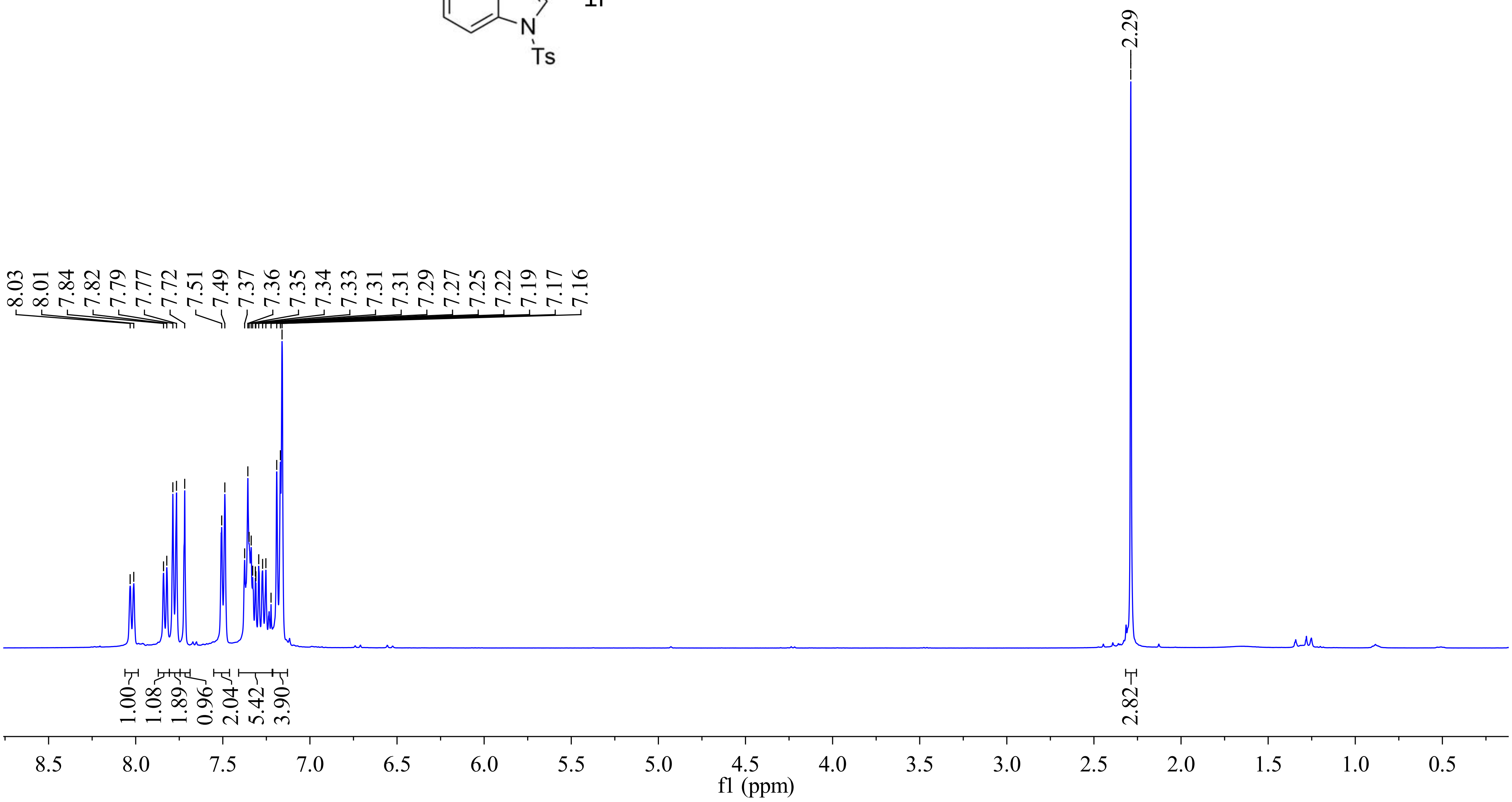

wyd-6-114-2 C

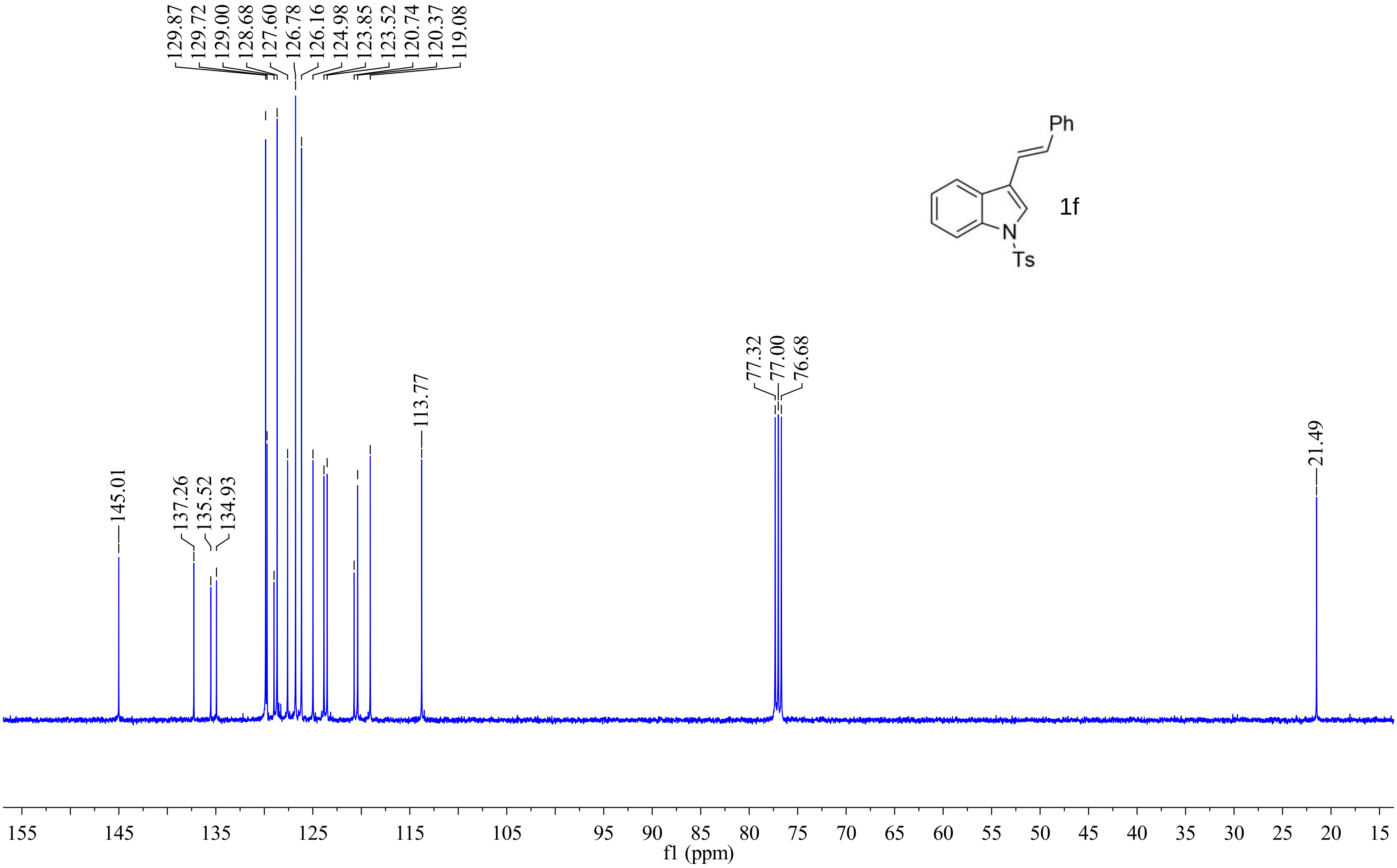

129.87
129.72
129.00
128.68
127.60
126.78
126.16
124.98
123.85
123.52
120.74
120.37
119.08
145.01
137.26
135.52
134.93
113.77
77.32
77.00
76.68
21.49
Ph
N
Ts
1f
155 145 135 125 115 105 95 90 85 80 75 70 65 60 55 50 45 40 35 30 25 20 15
f1 (ppm)

wyd-7-13 H

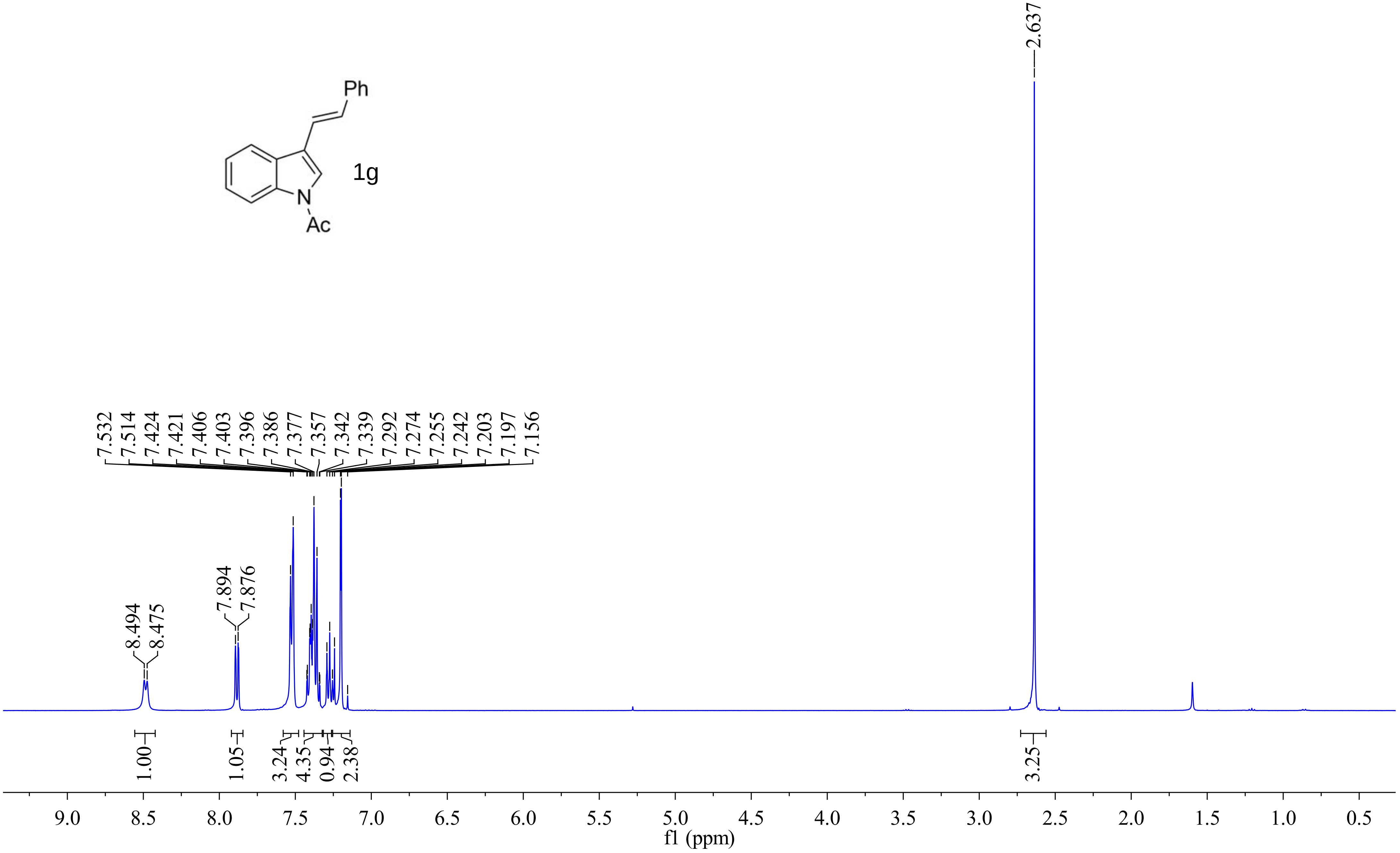
Ph
N
Ac
1g
2.637
7.532 7.514 7.424 7.421 7.406 7.403 7.396 7.386 7.377 7.357 7.342 7.339 7.292 7.274 7.255 7.242 7.203 7.197 7.156
8.494 8.475
7.894 7.876
1.00 1.05 3.24 4.35 0.94 2.38 3.25
9.0 8.5 8.0 7.5 7.0 6.5 6.0 5.5 5.0 4.5 4.0 3.5 3.0 2.5 2.0 1.5 1.0 0.5
f1 (ppm)

wyd-7-13 C

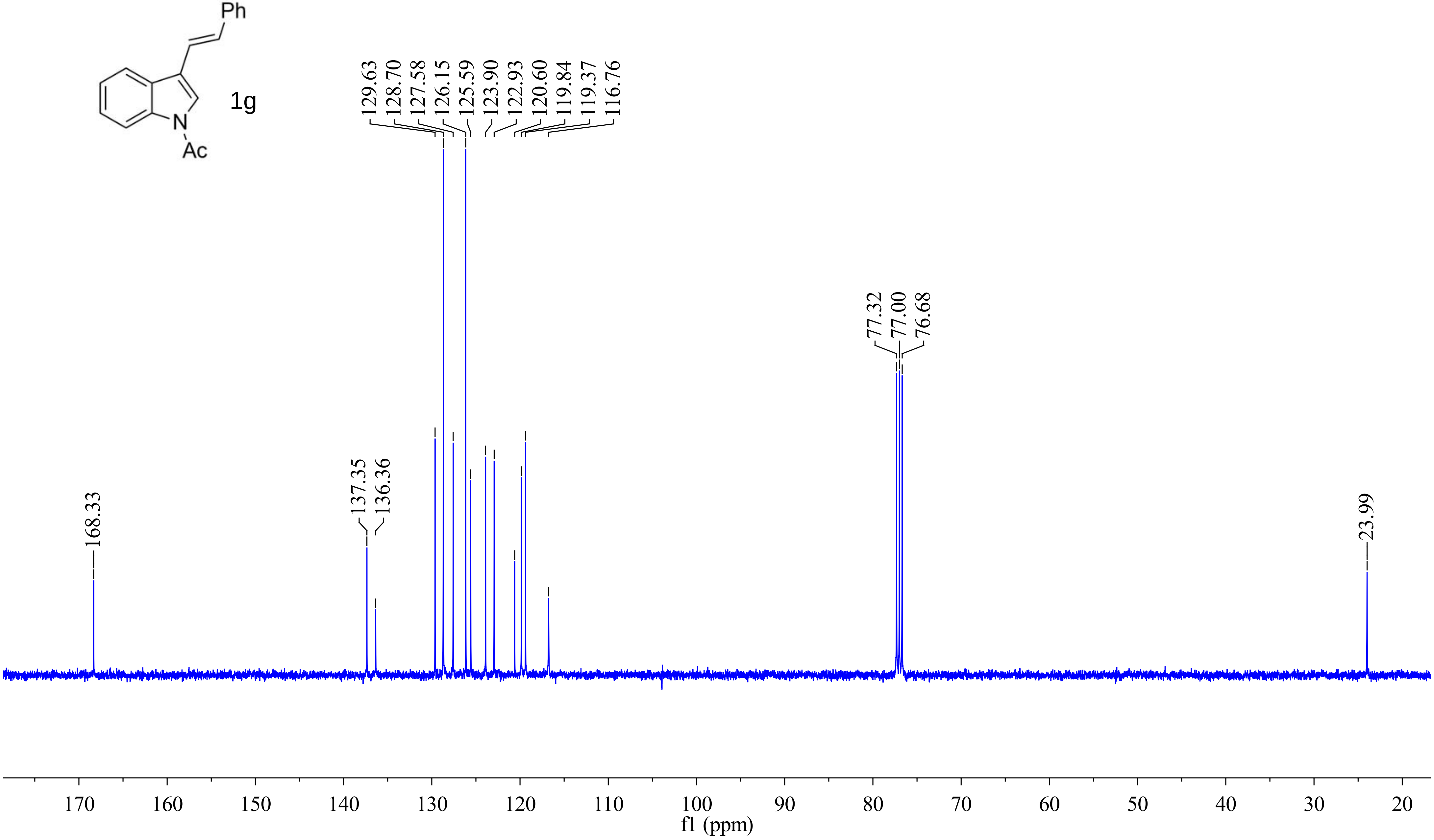
Ph
N
Ac
1g
129.63
128.70
127.58
126.15
125.59
123.90
122.93
120.60
119.84
119.37
116.76
77.32
77.00
76.68
168.33
137.35
136.36
23.99
170
160
150
140
130
120
110
100
90
80
70
60
50
40
30
20
f1 (ppm)

zpc-1-20 H

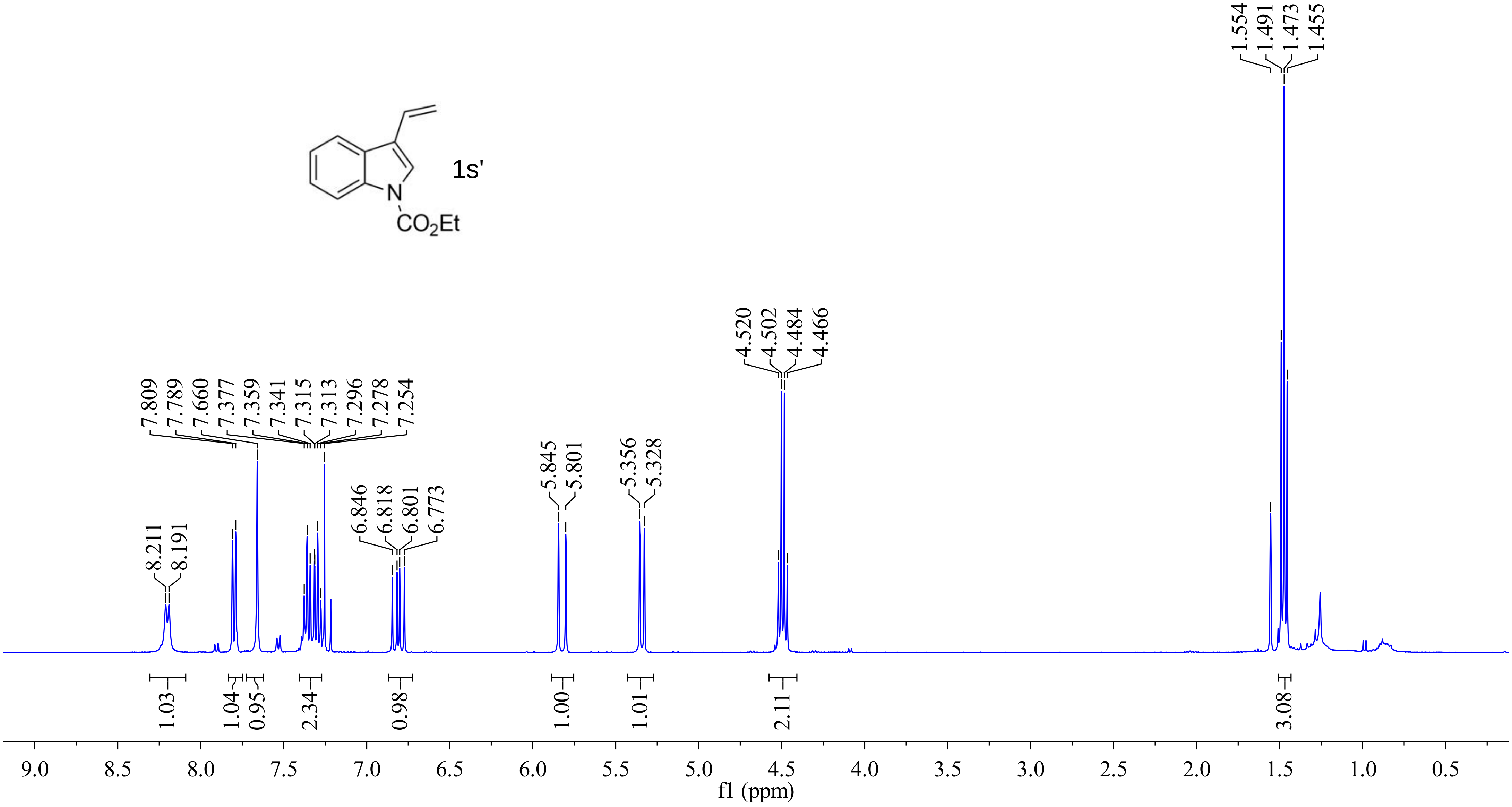

1s'
CO_2Et
N
8.211
8.191
7.809
7.789
7.660
7.377
7.359
7.341
7.315
7.313
7.296
7.278
7.254
6.846
6.818
6.801
6.773
5.845
5.801
5.356
5.328
4.520
4.502
4.484
4.466
1.554
1.491
1.473
1.455
1.03
1.04
0.95
2.34
0.98
1.00
1.01
2.11
3.08
9.0 8.5 8.0 7.5 7.0 6.5 6.0 5.5 5.0 4.5 4.0 3.5 3.0 2.5 2.0 1.5 1.0 0.5
f1 (ppm)

zpc-1-20 C

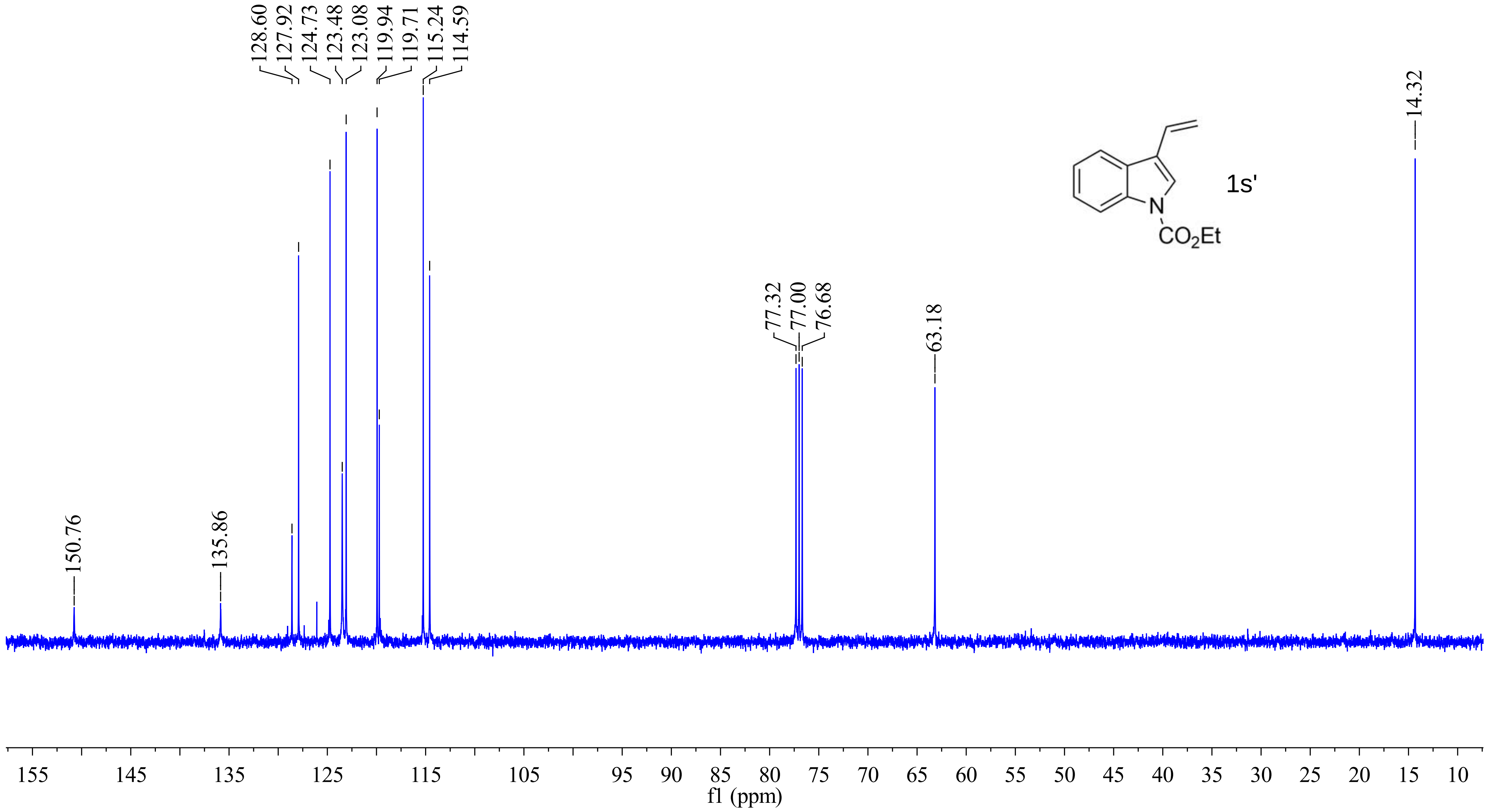

128.60
127.92
124.73
123.48
123.08
119.94
119.71
115.24
114.59
14.32
1s'
N
CO2Et
77.32
77.00
76.68
63.18
150.76
135.86
155
145
135
125
115
105
95
90
85
80
75
70
65
60
55
50
45
40
35
30
25
20
15
10
f1 (ppm)

wyd-7-45 H

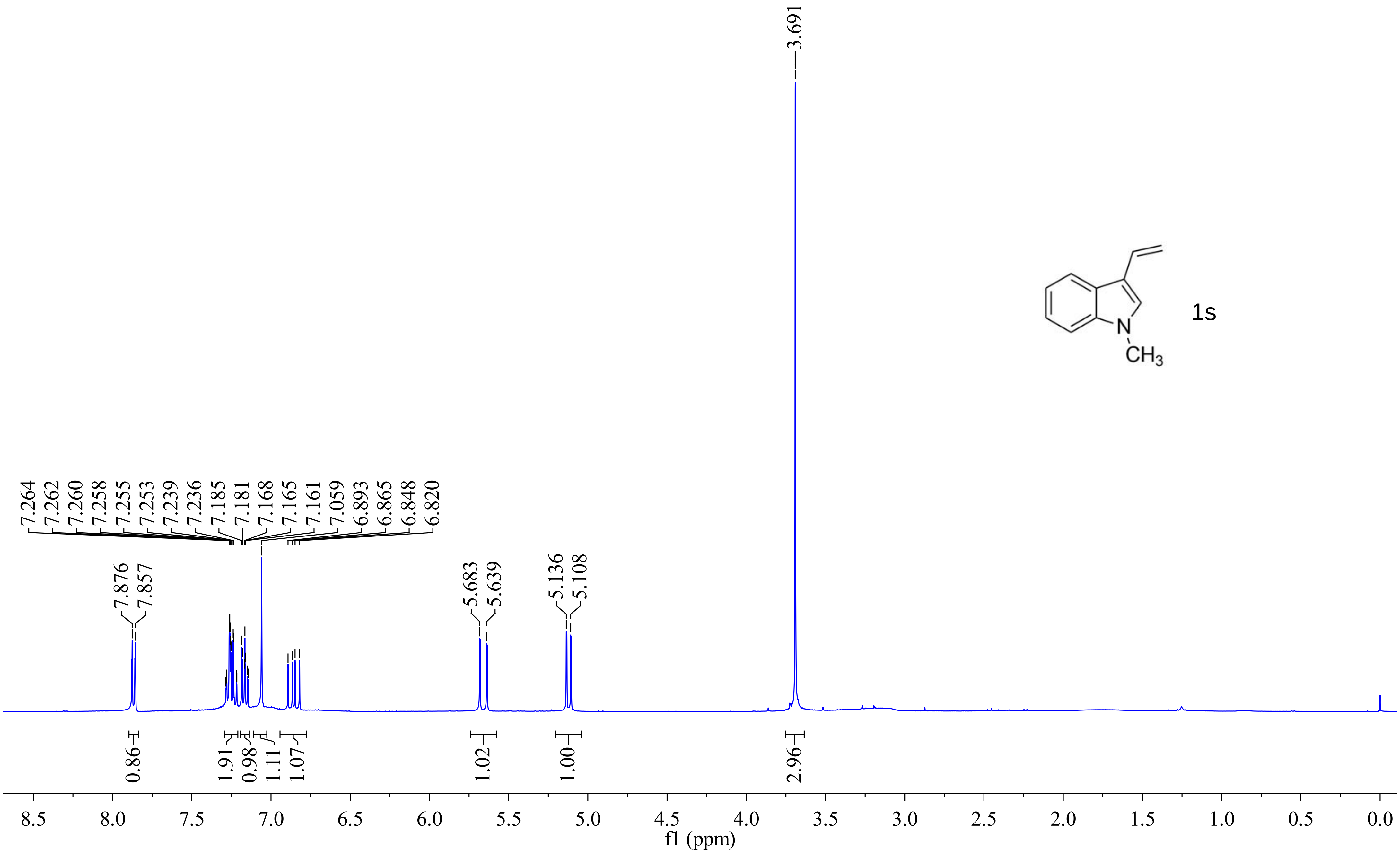
3.691
7.264
7.262
7.260
7.258
7.255
7.253
7.239
7.236
7.185
7.181
7.168
7.165
7.161
7.059
6.893
6.865
6.848
6.820
7.876
7.857
5.683
5.639
5.136
5.108
N
CH3
1s
0.86
1.91
0.98
1.11
1.07
1.02
1.00
2.96
8.5
8.0
7.5
7.0
6.5
6.0
5.5
5.0
4.5
4.0
3.5
3.0
2.5
2.0
1.5
1.0
0.5
0.0
f1 (ppm)

wyd-7-45 C

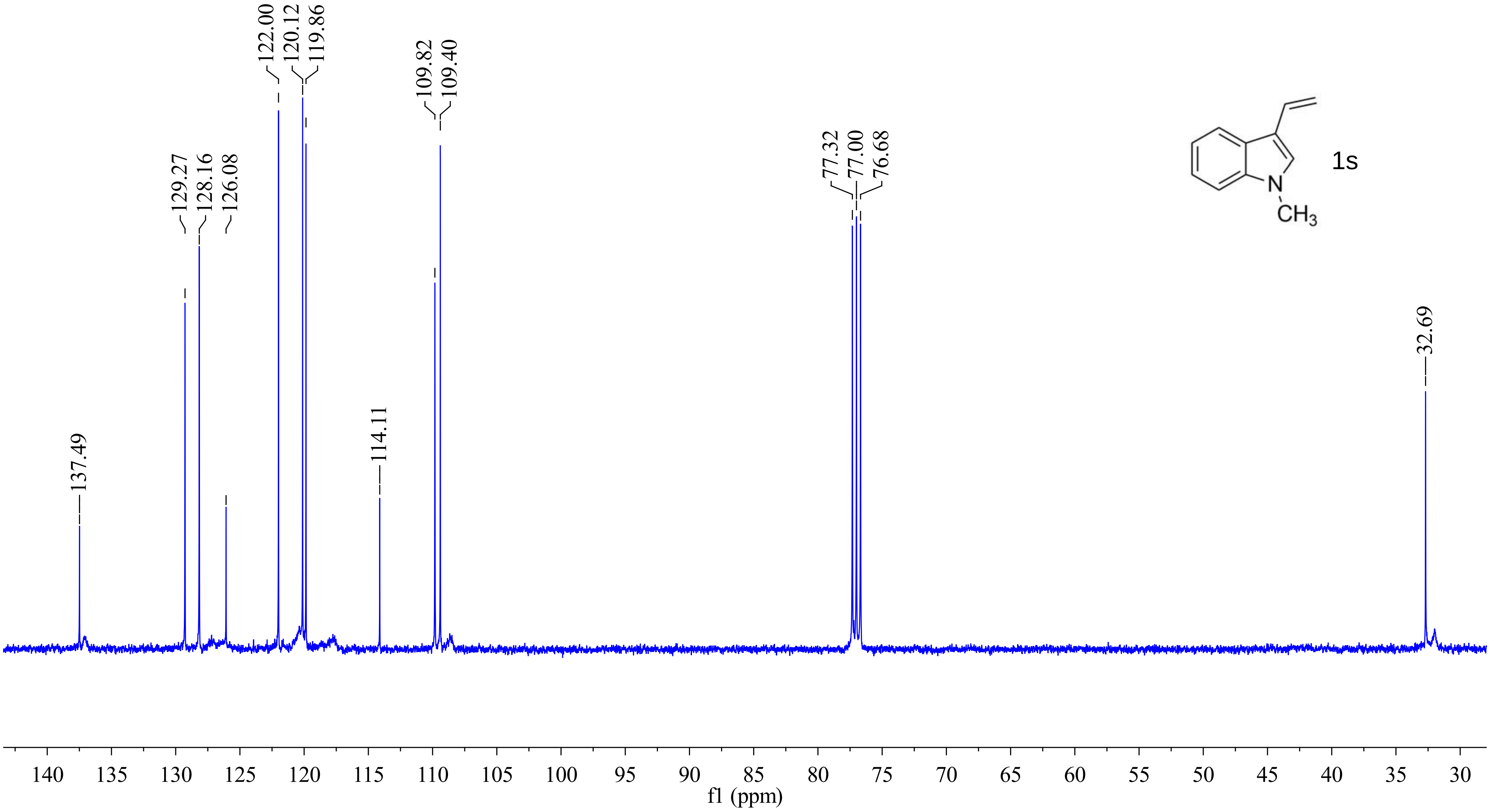
122.00
120.12
119.86
109.82
109.40
129.27
128.16
126.08
77.32
77.00
76.68
137.49
114.11
32.69
1s
N
CH3
140 135 130 125 120 115 110 105 100 95 90 85 80 75 70 65 60 55 50 45 40 35 30
f1 (ppm)

wyd-7-39 H

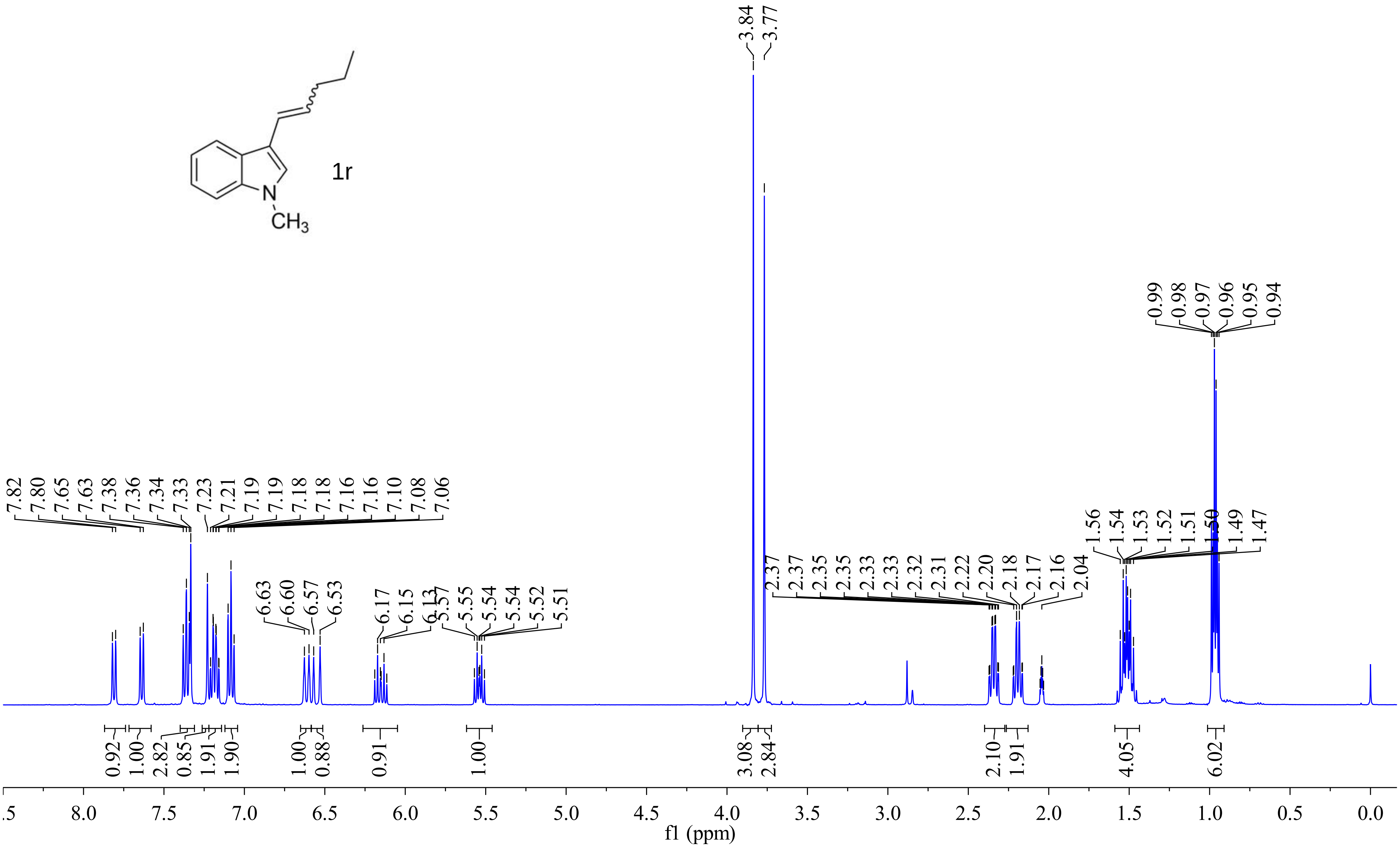
1r
N
CH3
7.82 7.80 7.65 7.63 7.38 7.36 7.34 7.33 7.23 7.21 7.19 7.19 7.18 7.18 7.16 7.16 7.10 7.08 7.06
6.63 6.60 6.57 6.53
6.17 6.15 6.13 5.57 5.55 5.54 5.54 5.52 5.51
3.84 3.77
2.37 2.37 2.35 2.35 2.33 2.33 2.32 2.31 2.22 2.20 2.18 2.17 2.16 2.04
1.56 1.54 1.53 1.52 1.51 1.50 1.49 1.47
0.99 0.98 0.97 0.96 0.95 0.94
0.92 1.00 2.82 0.85 1.91 1.90 1.00 0.88 0.91 1.00 3.08 2.84 2.10 1.91 4.05 6.02
.5 8.0 7.5 7.0 6.5 6.0 5.5 5.0 4.5 4.0 3.5 3.0 2.5 2.0 1.5 1.0 0.5 0.0
f1 (ppm)

wyd-7-39 C

1r

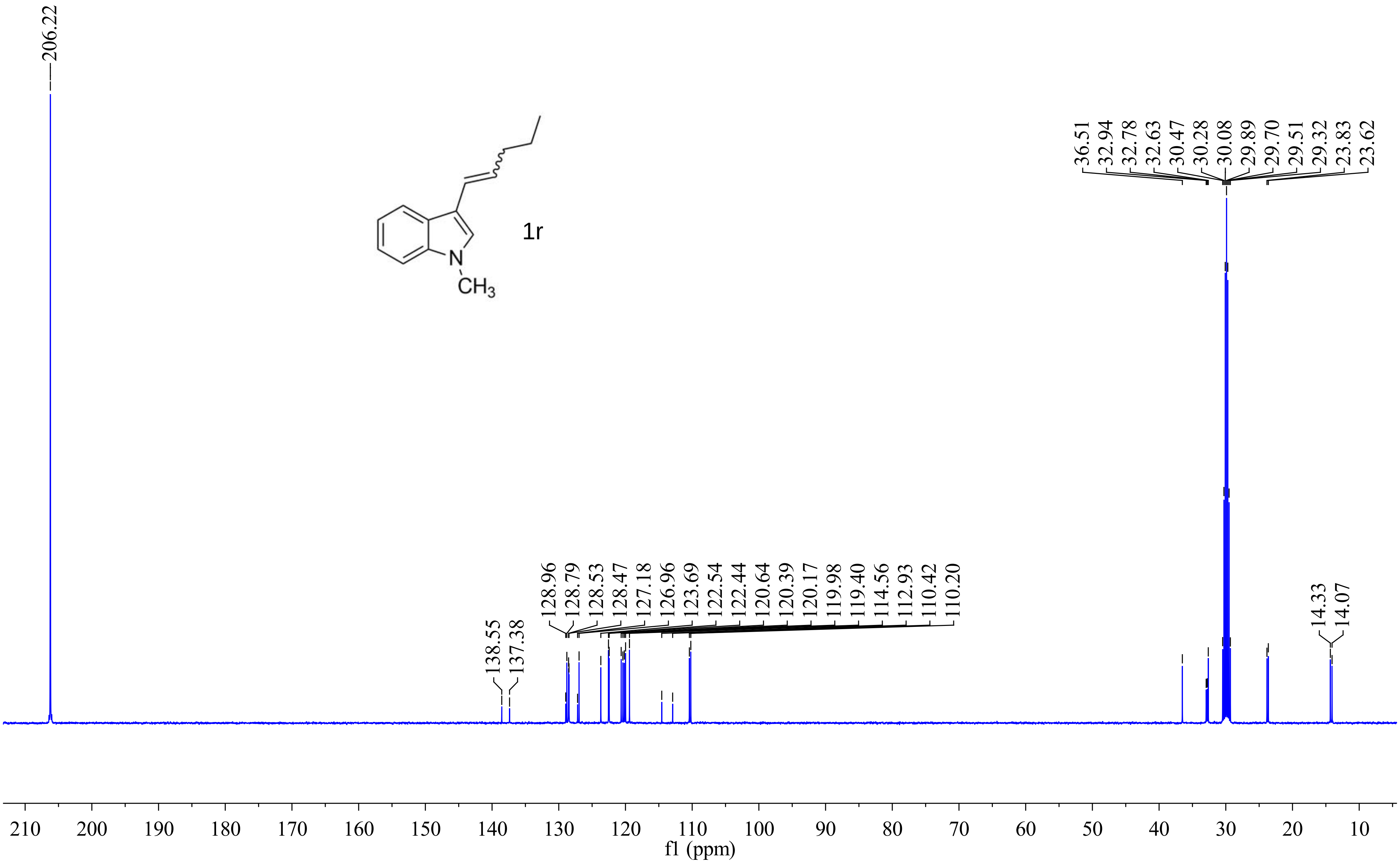

206.22
138.55
137.38
128.96
128.79
128.53
128.47
127.18
126.96
123.69
122.54
122.44
120.64
120.39
120.17
119.98
119.40
114.56
112.93
110.42
110.20
36.51
32.94
32.78
32.63
30.47
30.28
30.08
29.89
29.70
29.51
29.32
23.83
23.62
14.33
14.07
210 200 190 180 170 160 150 140 130 120 110 100 90 80 70 60 50 40 30 20 10
f1 (ppm)

wyd-7-40 H

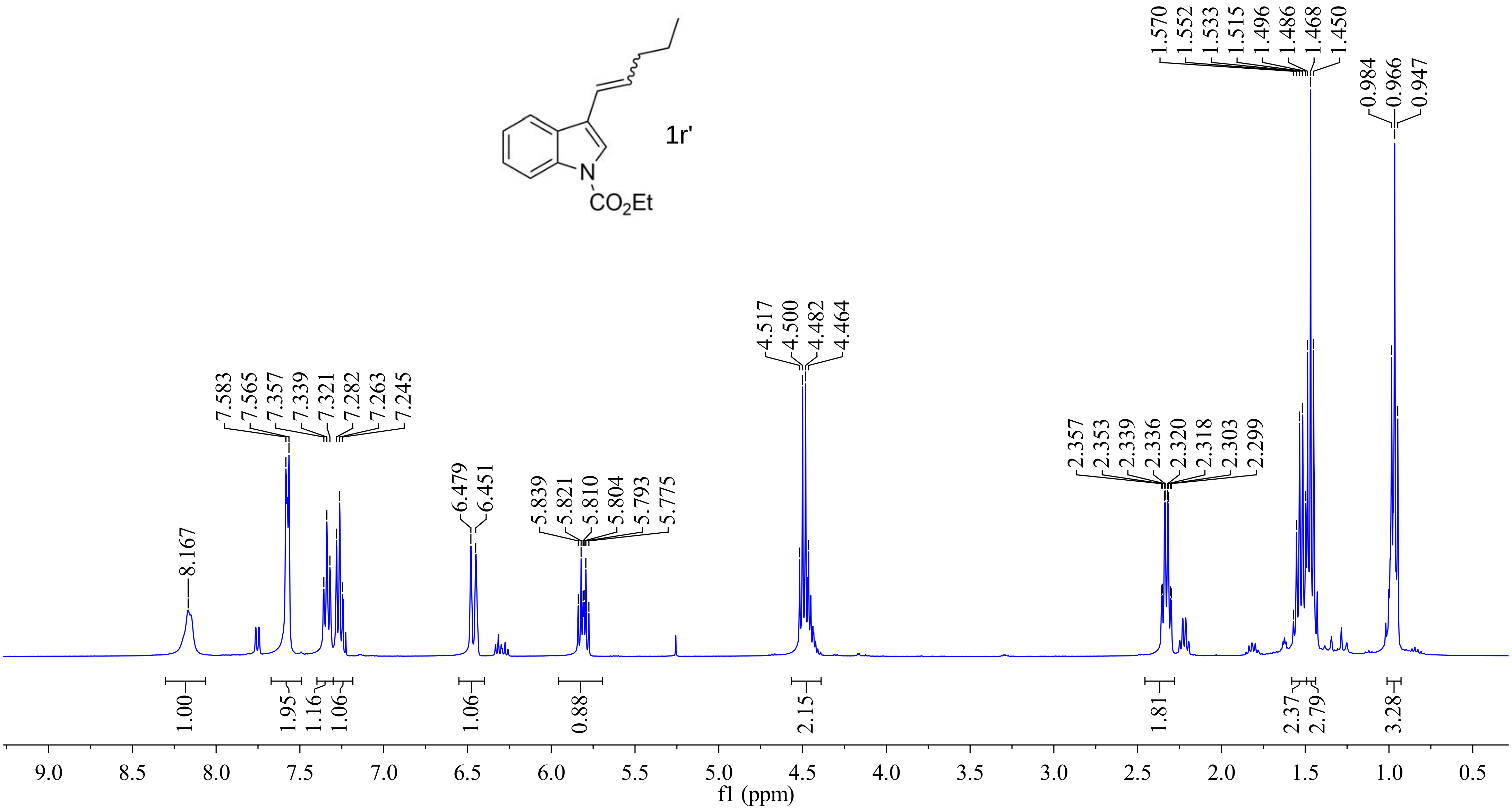
N
CO2Et
1r'
8.167
7.583
7.565
7.357
7.339
7.321
7.282
7.263
7.245
6.479
6.451
5.839
5.821
5.810
5.804
5.793
5.775
4.517
4.500
4.482
4.464
2.357
2.353
2.339
2.336
2.320
2.318
2.303
2.299
1.570
1.552
1.533
1.515
1.496
1.486
1.468
1.450
0.984
0.966
0.947
1.00
1.95
1.16
1.06
1.06
0.88
2.15
1.81
2.37
2.79
3.28
9.0
8.5
8.0
7.5
7.0
6.5
6.0
5.5
5.0
4.5
4.0
3.5
3.0
2.5
2.0
1.5
1.0
0.5
f1 (ppm)

wyd-7-40 C

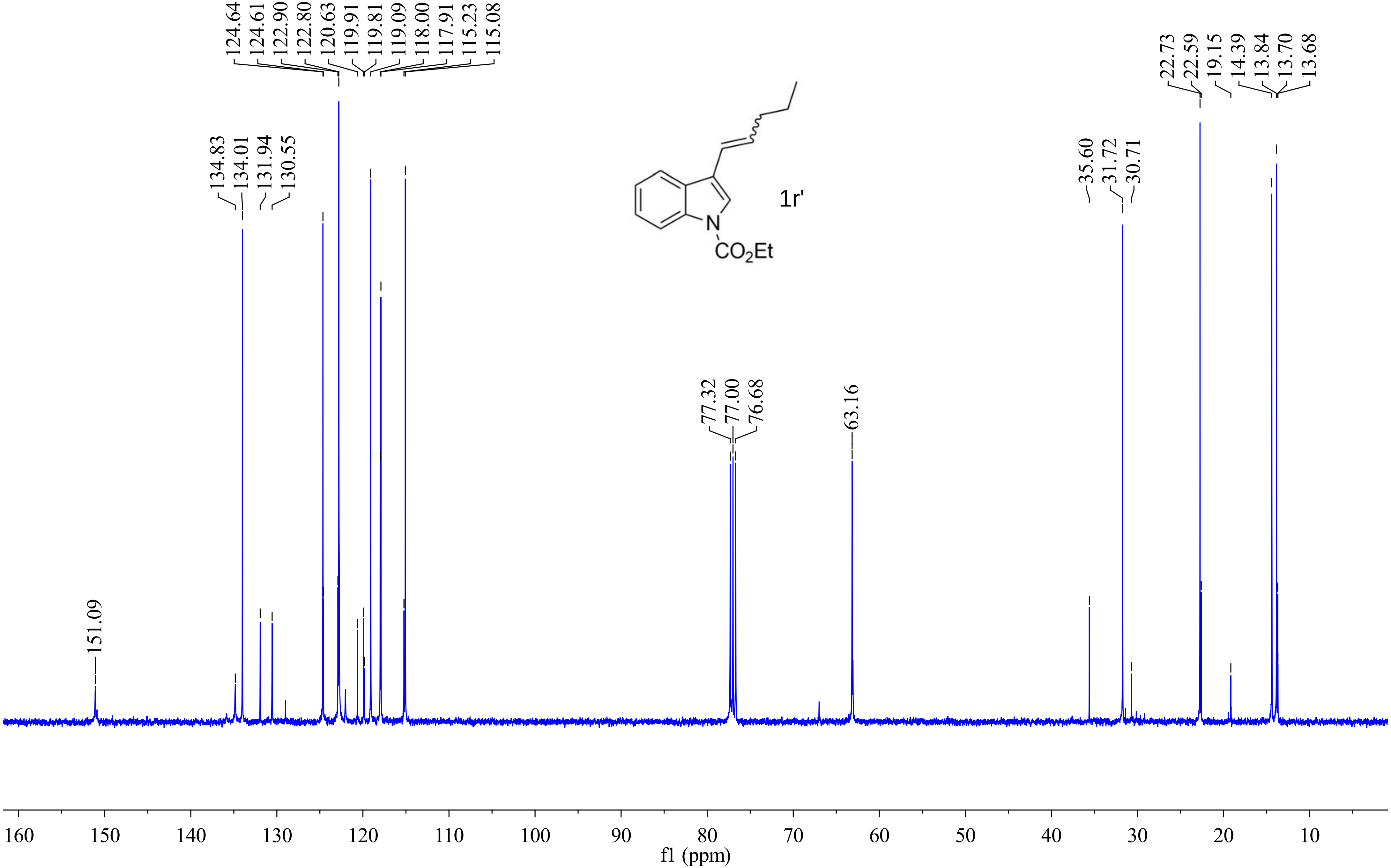

124.64
124.61
122.90
122.80
120.63
119.91
119.81
119.09
118.00
117.91
115.23
115.08
134.83
134.01
131.94
130.55
N
CO2Et
1r'
22.73
22.59
19.15
14.39
13.84
13.70
13.68
35.60
31.72
30.71
77.32
77.00
76.68
63.16
151.09
160
150
140
130
120
110
100
90
80
70
60
50
40
30
20
10
f1 (ppm)

wyd-5-44 H

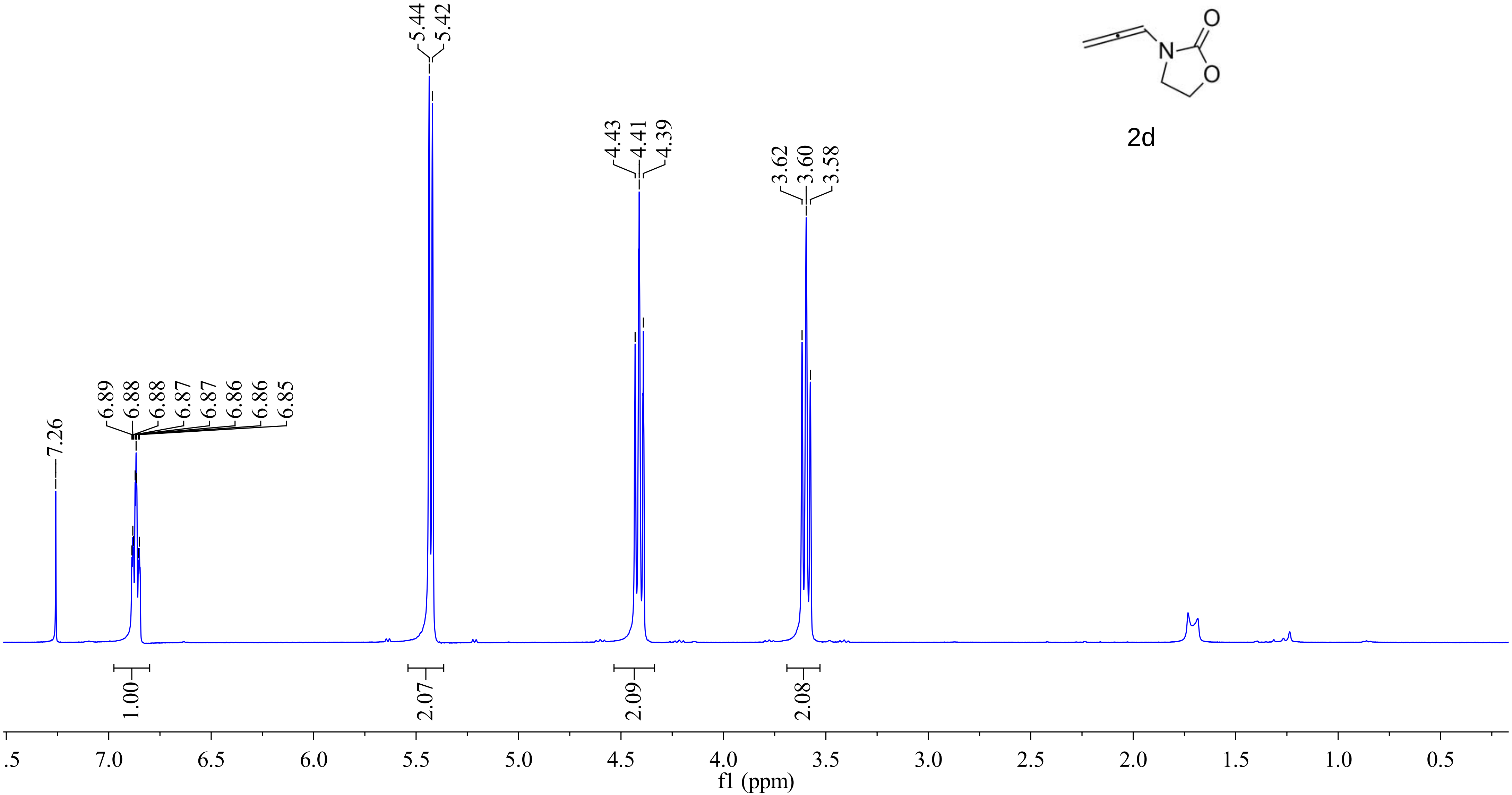

7.26
6.89
6.88
6.88
6.87
6.87
6.86
6.86
6.85
5.44
5.42
4.43
4.41
4.39
3.62
3.60
3.58
1.00
2.07
2.09
2.08
.5
7.0
6.5
6.0
5.5
5.0
4.5
4.0
3.5
3.0
2.5
2.0
1.5
1.0
0.5
f1 (ppm)
N
O
O

2d

wyd-5-44 C

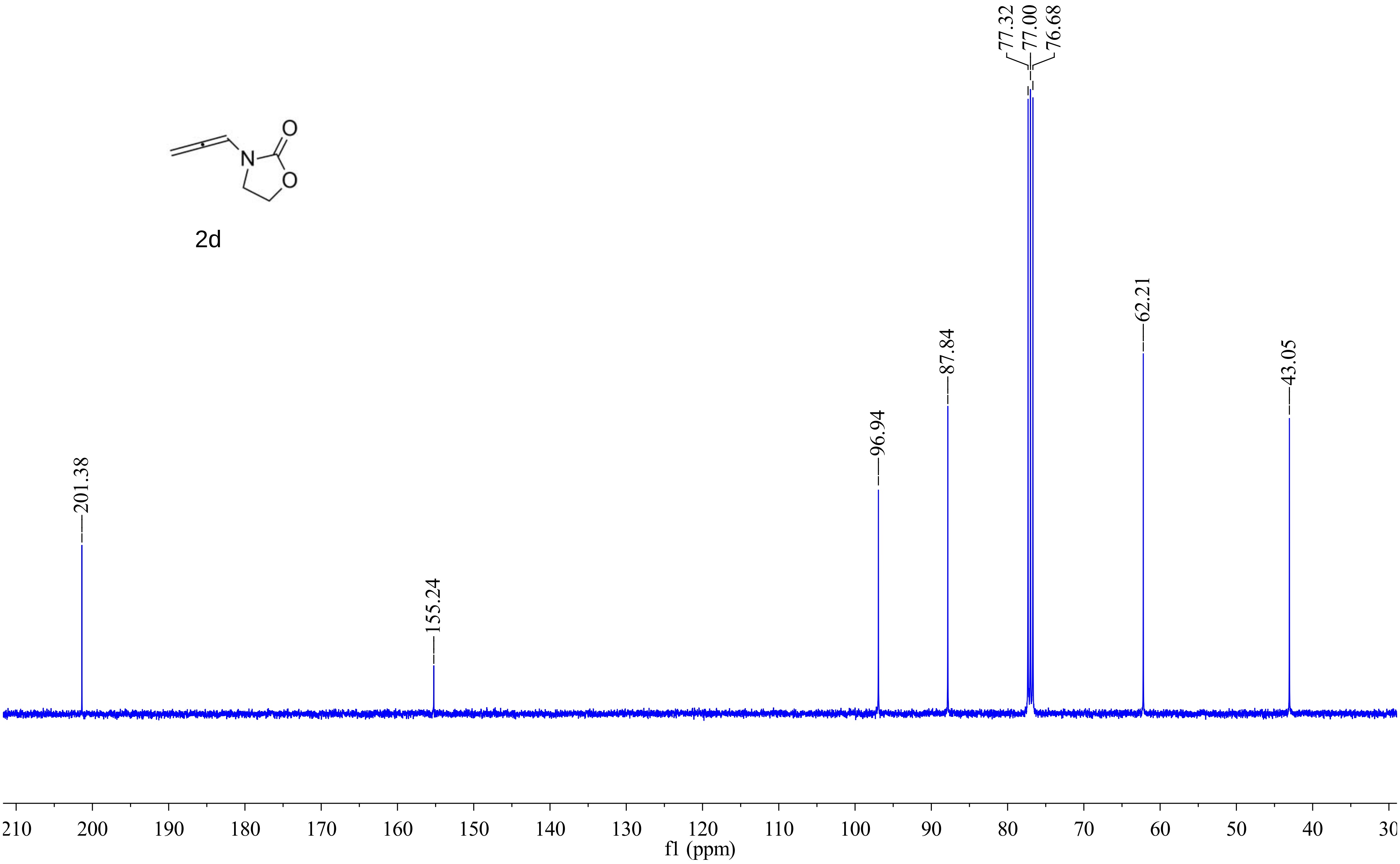
2d
201.38
155.24
96.94
87.84
77.32
77.00
76.68
62.21
43.05
210 200 190 180 170 160 150 140 130 120 110 100 90 80 70 60 50 40 30
f1 (ppm)

wyd-4-141 H

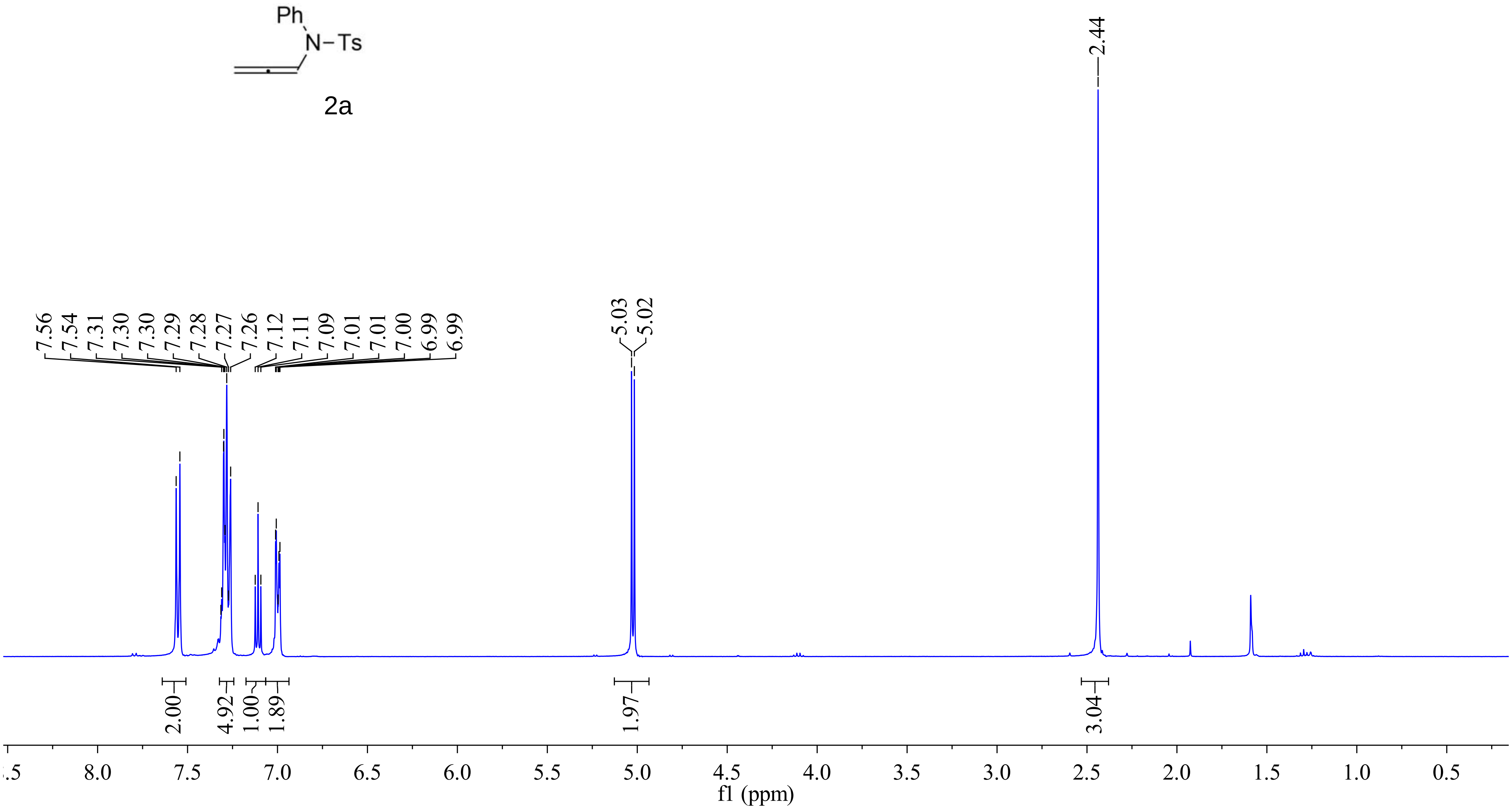

Ph
N–Ts
2a
7.56
7.54
7.31
7.30
7.30
7.29
7.28
7.27
7.26
7.12
7.11
7.09
7.01
7.01
7.00
6.99
6.99
5.03
5.02
2.44
2.00
4.92
1.00
1.89
1.97
3.04
8.0 7.5 7.0 6.5 6.0 5.5 5.0 4.5 4.0 3.5 3.0 2.5 2.0 1.5 1.0 0.5
f1 (ppm)

wyd-4-141 C

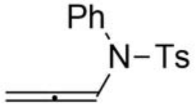
Ph
N–Ts

2a

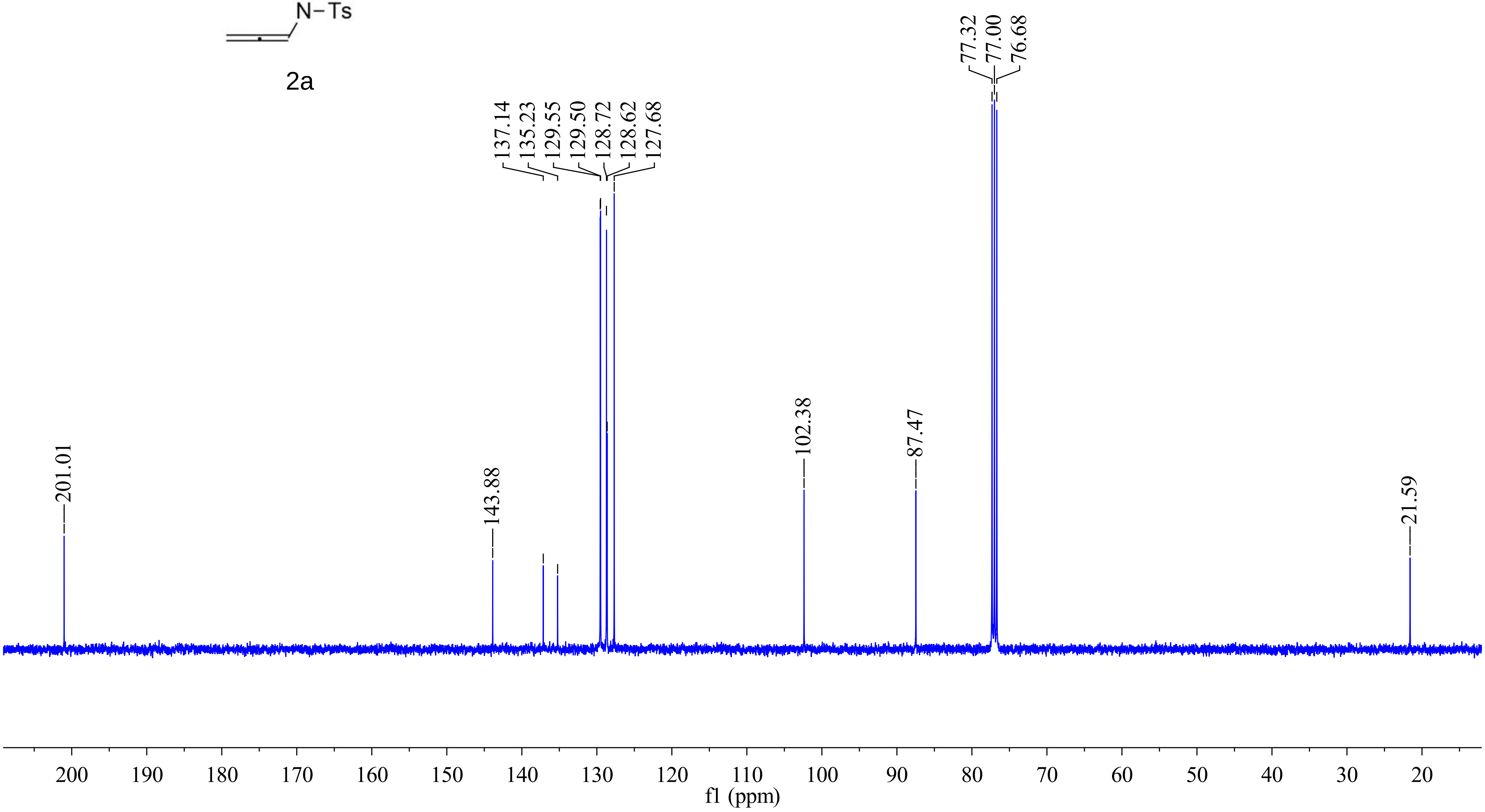
201.01
143.88
137.14
135.23
129.55
129.50
128.72
128.62
127.68
102.38
87.47
77.32
77.00
76.68
21.59
200
190
180
170
160
150
140
130
120
110
100
90
80
70
60
50
40
30
20
f1 (ppm)

zpc-1-2

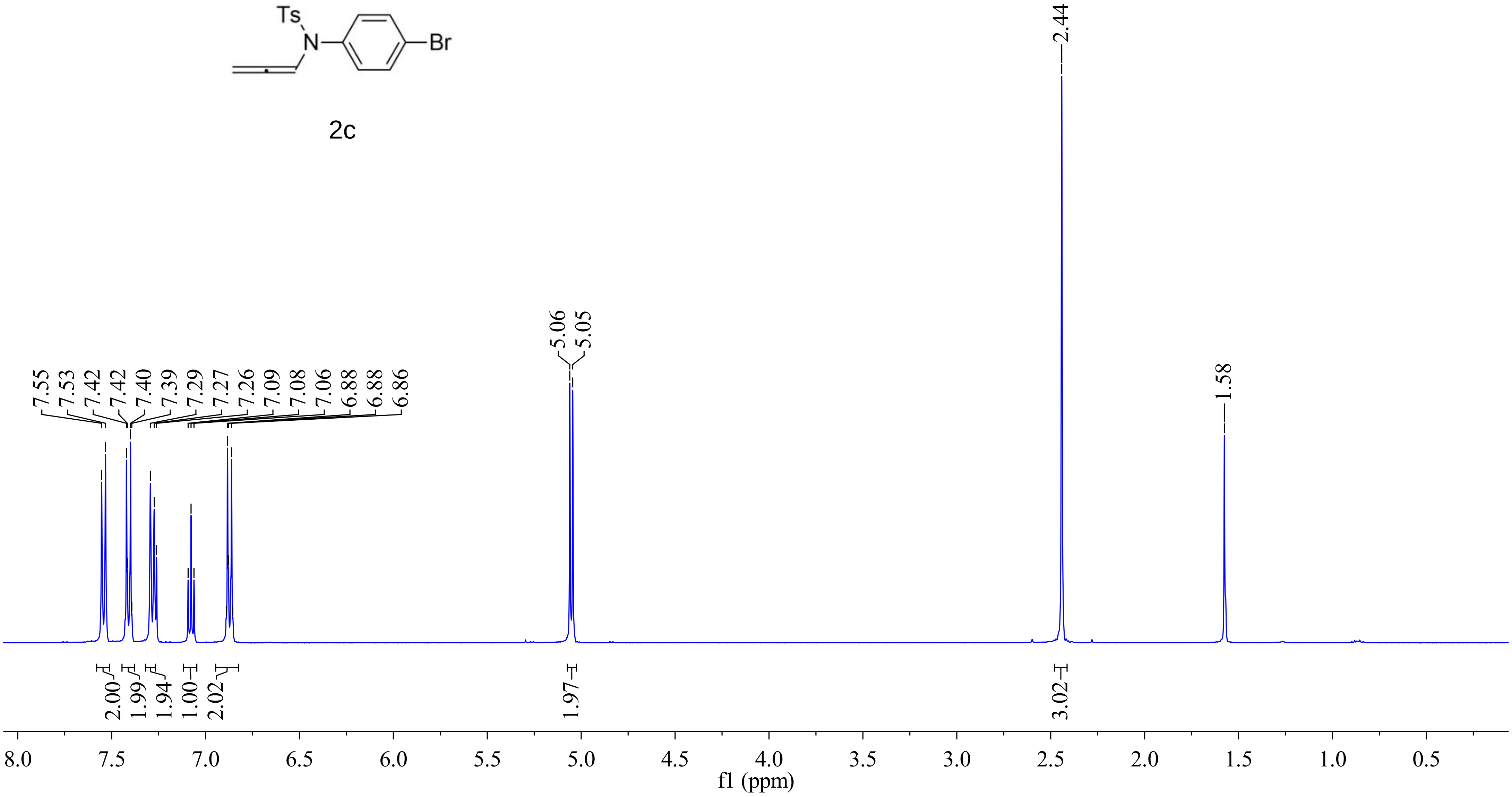
Ts
N
Br
2c
7.55
7.53
7.42
7.42
7.40
7.39
7.29
7.27
7.26
7.09
7.08
7.06
6.88
6.88
6.86
5.06
5.05
2.44
1.58
2.00
1.99
1.94
1.00
2.02
1.97
3.02
8.0
7.5
7.0
6.5
6.0
5.5
5.0
4.5
4.0
3.5
3.0
2.5
2.0
1.5
1.0
0.5
f1 (ppm)

zpc-1-2

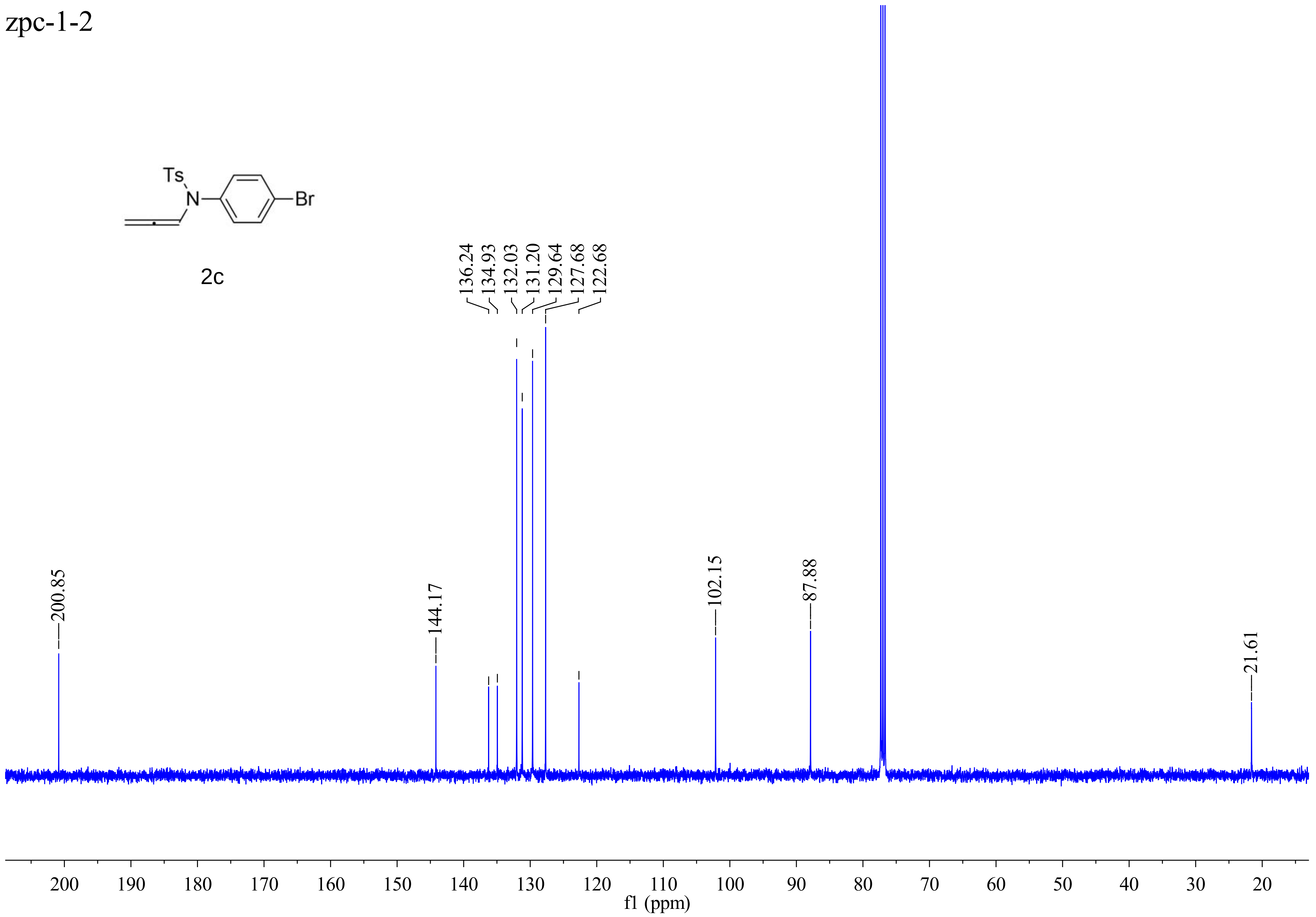

Ts
N
Br
2c
200.85
144.17
136.24
134.93
132.03
131.20
129.64
127.68
122.68
102.15
87.88
21.61
200
190
180
170
160
150
140
130
120
110
100
90
80
70
60
50
40
30
20
f1 (ppm)

wyd-6-10 H

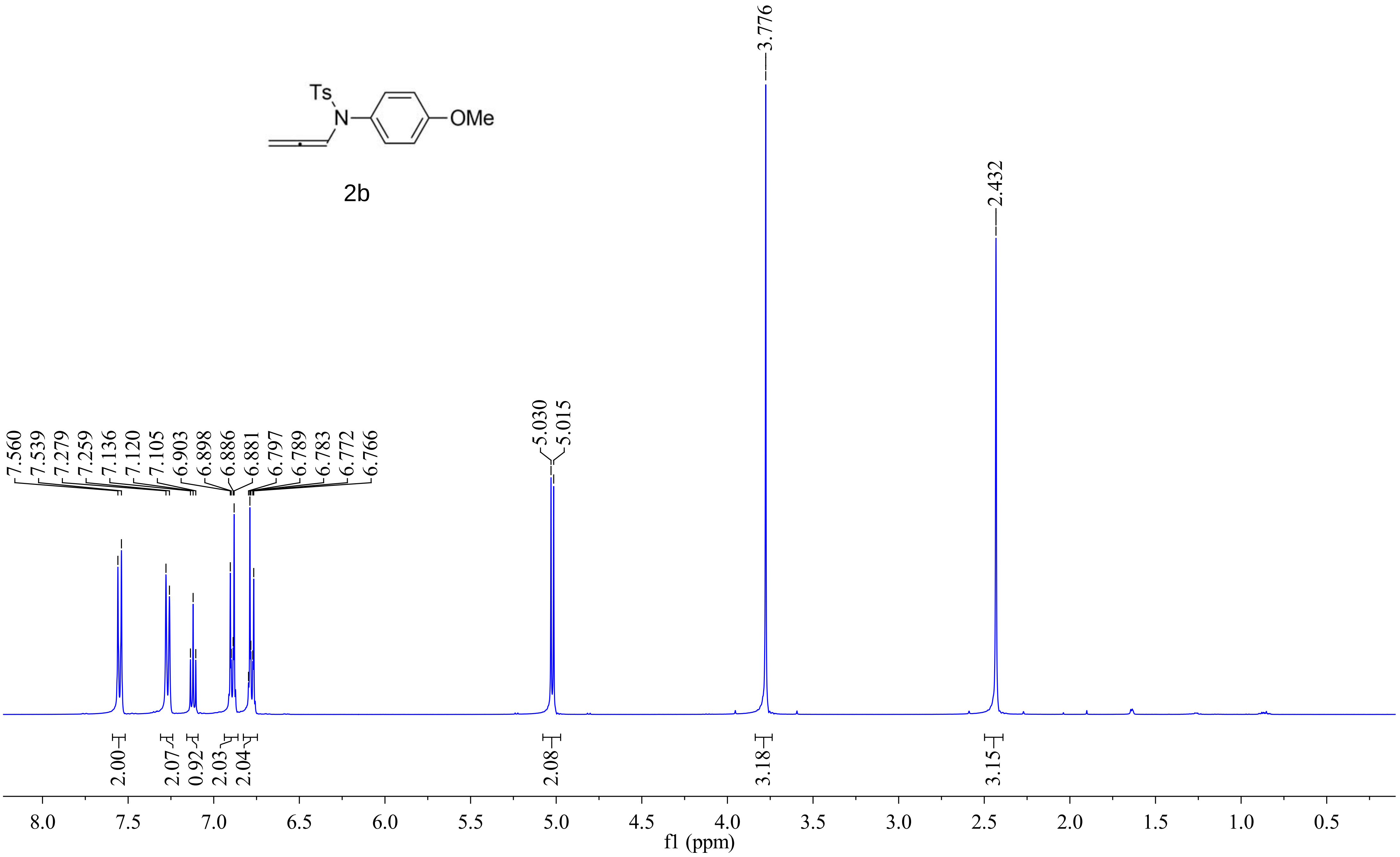
Ts
N
OMe
2b
7.560
7.539
7.279
7.259
7.136
7.120
7.105
6.903
6.898
6.886
6.881
6.797
6.789
6.783
6.772
6.766
5.030
5.015
3.776
2.432
2.00
2.07
0.92
2.03
2.04
2.08
3.18
3.15
8.0 7.5 7.0 6.5 6.0 5.5 5.0 4.5 4.0 3.5 3.0 2.5 2.0 1.5 1.0 0.5
f1 (ppm)

wyd-6-10 C

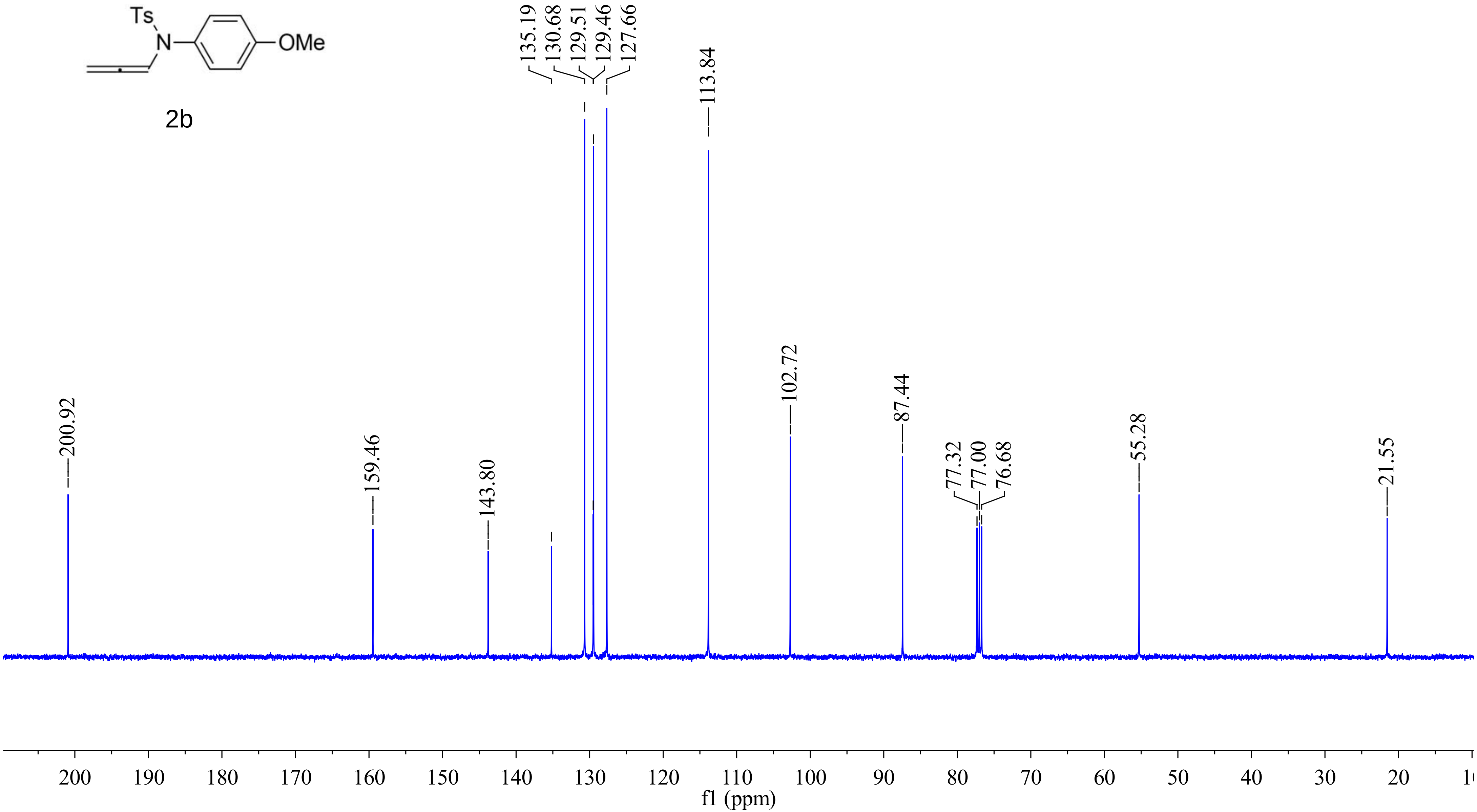
Ts
N
OMe
2b
135.19
130.68
129.51
129.46
127.66
113.84
200.92
159.46
143.80
102.72
87.44
77.32
77.00
76.68
55.28
21.55
200
190
180
170
160
150
140
130
120
110
100
90
80
70
60
50
40
30
20
f1 (ppm)

wyd-6-20 H

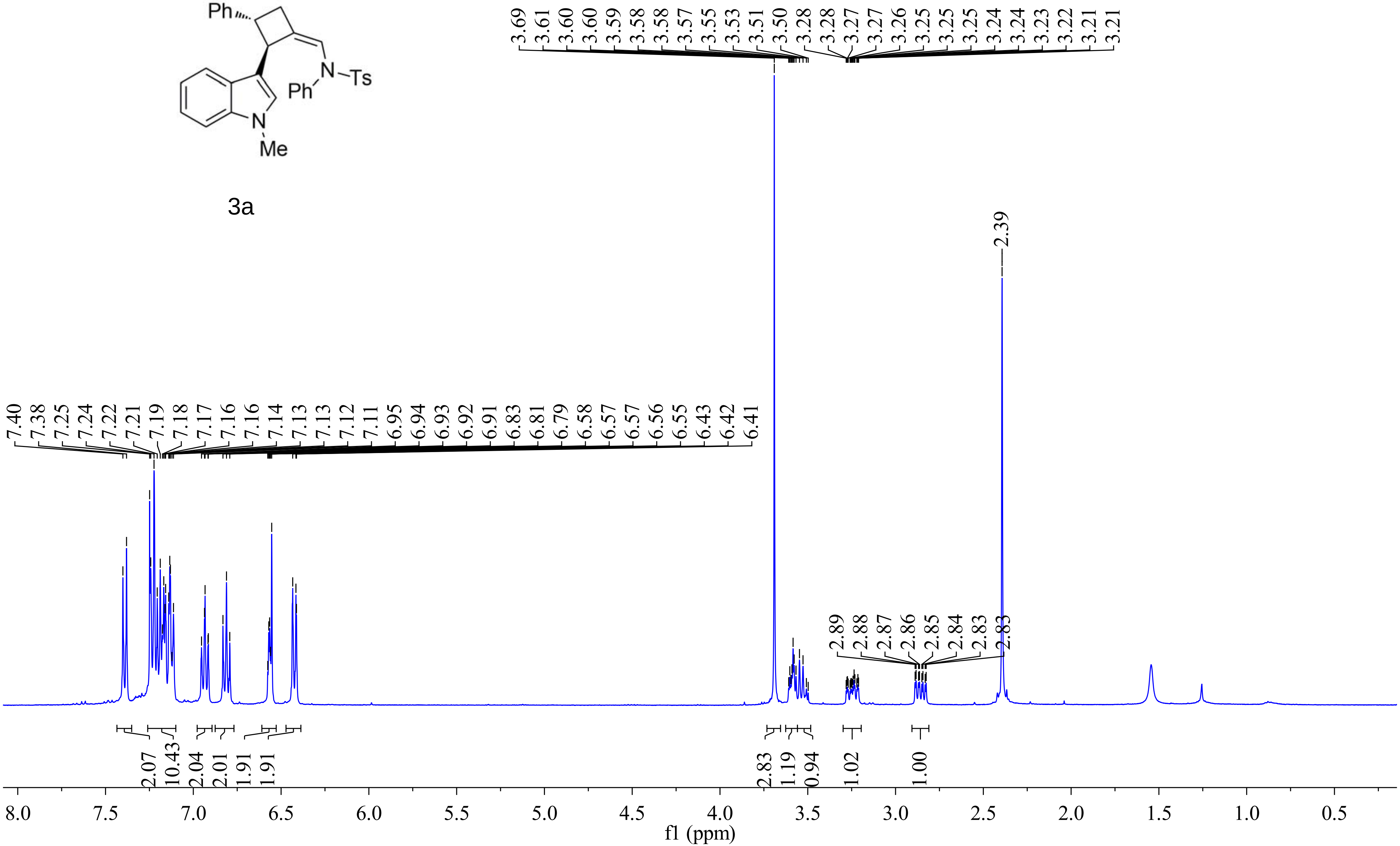
Ph
N
Ts
Ph
N
Me
3a
3.69
3.61
3.60
3.60
3.59
3.58
3.58
3.57
3.55
3.53
3.51
3.50
3.28
3.28
3.27
3.27
3.26
3.25
3.25
3.25
3.24
3.24
3.23
3.22
3.21
3.21
2.39
7.40
7.38
7.25
7.24
7.22
7.21
7.19
7.18
7.17
7.16
7.16
7.14
7.13
7.13
7.12
7.11
6.95
6.94
6.93
6.92
6.91
6.83
6.81
6.79
6.58
6.57
6.57
6.56
6.55
6.43
6.42
6.41
2.89
2.88
2.87
2.86
2.85
2.84
2.83
2.83
2.07
10.43
2.04
2.01
1.91
1.91
2.83
1.19
0.94
1.02
1.00
8.0
7.5
7.0
6.5
6.0
5.5
5.0
4.5
4.0
3.5
3.0
2.5
2.0
1.5
1.0
0.5
f1 (ppm)

wyd-6-20 C

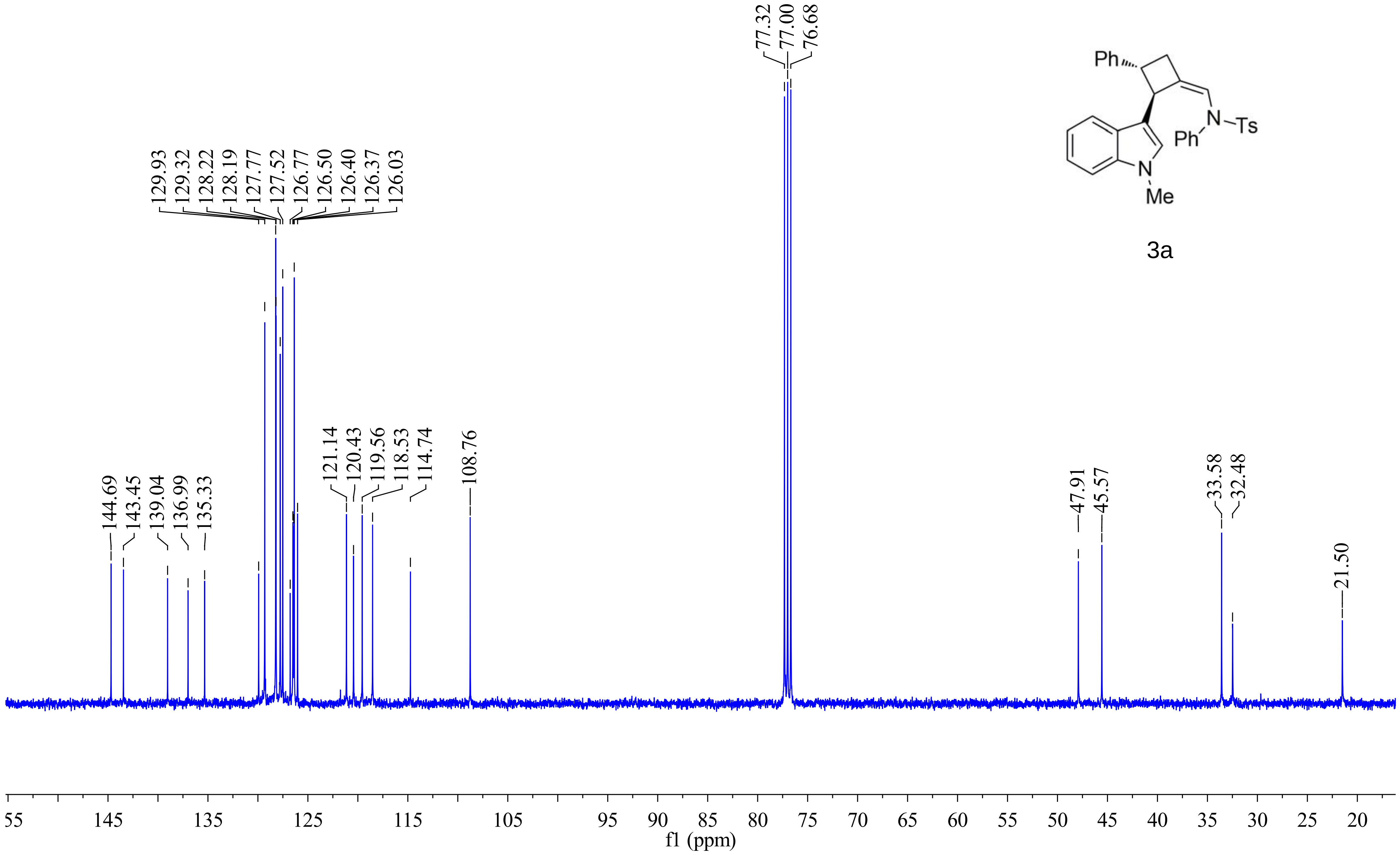

129.93
129.32
128.22
128.19
127.77
127.52
126.77
126.50
126.40
126.37
126.03
144.69
143.45
139.04
136.99
135.33
121.14
120.43
119.56
118.53
114.74
108.76
77.32
77.00
76.68
47.91
45.57
33.58
32.48
21.50
55 145 135 125 115 105 95 90 85 80 75 70 65 60 55 50 45 40 35 30 25 20
f1 (ppm)
Ph
N
Ts
Ph
N
Me

3a

wyd-6-45 H

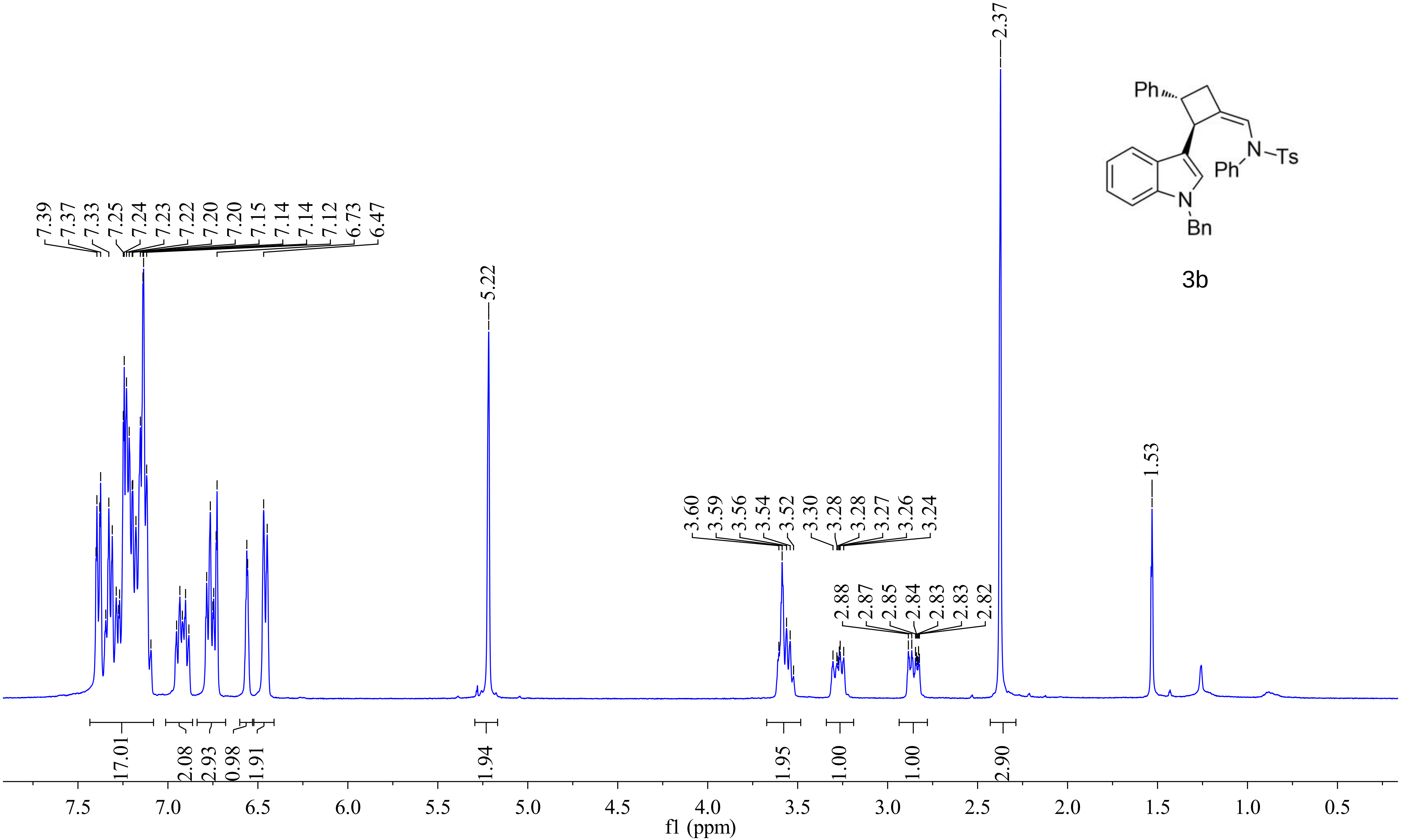

7.39
7.37
7.33
7.25
7.24
7.23
7.22
7.20
7.20
7.15
7.14
7.14
7.12
6.73
6.47
5.22
3.60
3.59
3.56
3.54
3.52
3.30
3.28
3.28
3.27
3.26
3.24
2.88
2.87
2.85
2.84
2.83
2.83
2.82
2.37
1.53
Ph
N
Ts
Ph
N
Bn
3b
17.01
2.08
2.93
0.98
1.91
1.94
1.95
1.00
1.00
2.90
7.5
7.0
6.5
6.0
5.5
5.0
4.5
4.0
3.5
3.0
2.5
2.0
1.5
1.0
0.5
f1 (ppm)

wyd-6-45 C

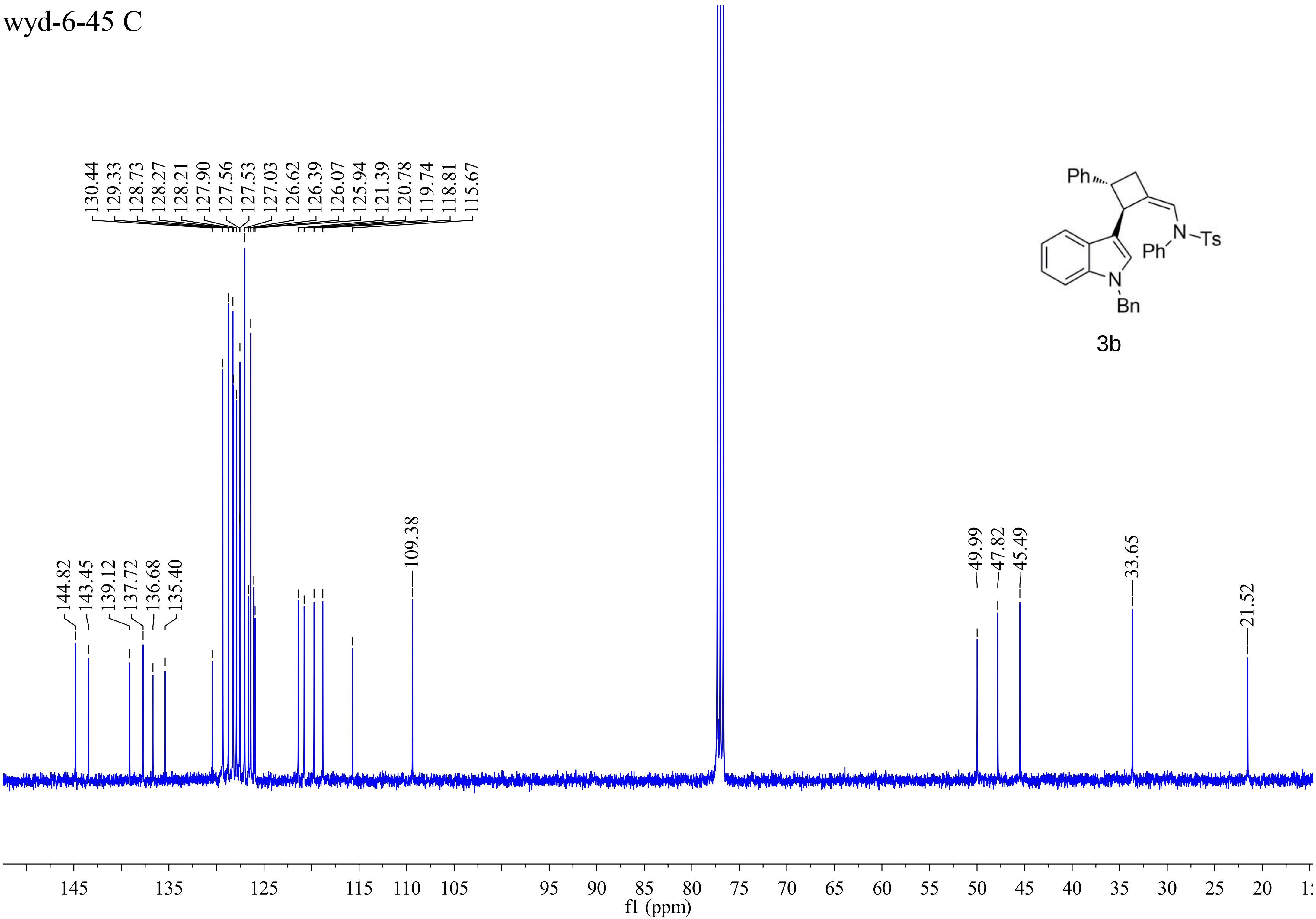

130.44
129.33
128.73
128.27
128.21
127.90
127.56
127.53
127.03
126.62
126.39
126.07
125.94
121.39
120.78
119.74
118.81
115.67
144.82
143.45
139.12
137.72
136.68
135.40
109.38
49.99
47.82
45.49
33.65
21.52
Ph
N
Ts
Ph
N
Bn
3b
fl (ppm)

wyd-7-14 H

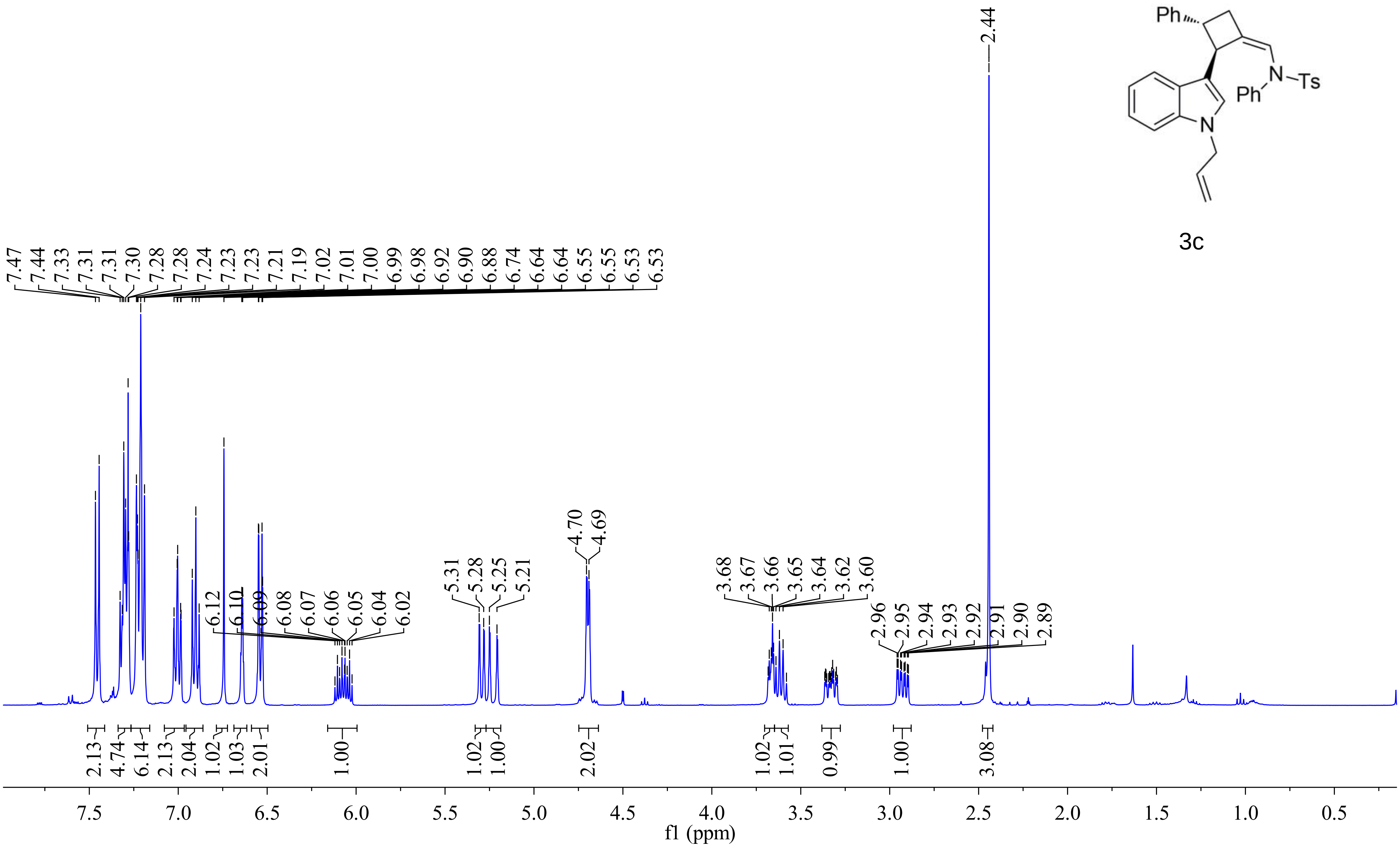

7.47 7.44 7.33 7.31 7.31 7.30 7.28 7.28 7.24 7.23 7.23 7.21 7.19 7.02 7.01 7.00 6.99 6.98 6.92 6.90 6.88 6.74 6.64 6.64 6.55 6.55 6.53 6.53
6.12 6.10 6.09 6.08 6.07 6.06 6.05 6.04 6.02
5.31 5.28 5.25 5.21
4.70 4.69
3.68 3.67 3.66 3.65 3.64 3.62 3.60
2.96 2.95 2.94 2.93 2.92 2.91 2.90 2.89
2.44
2.13 4.74 6.14 2.13 2.04 1.02 1.03 2.01 1.00 1.02 1.00 2.02 1.02 1.01 0.99 1.00 3.08
7.5 7.0 6.5 6.0 5.5 5.0 4.5 4.0 3.5 3.0 2.5 2.0 1.5 1.0 0.5
f1 (ppm)
Ph
N
Ts
Ph
N
3c

wyd-7-14 C

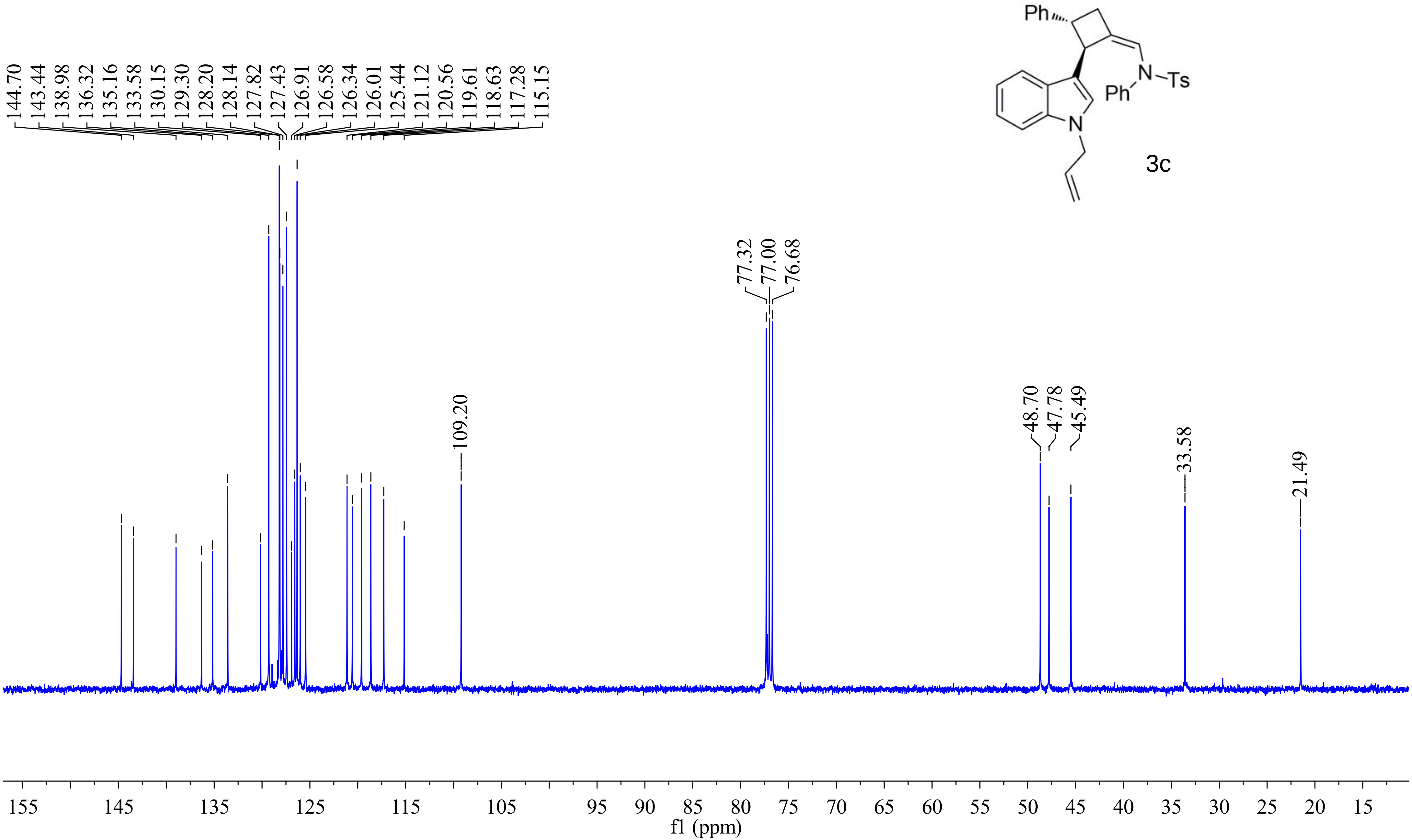
144.70
143.44
138.98
136.32
135.16
133.58
130.15
129.30
128.20
128.14
127.82
127.43
126.91
126.58
126.34
126.01
125.44
121.12
120.56
119.61
118.63
117.28
115.15
109.20
77.32
77.00
76.68
48.70
47.78
45.49
33.58
21.49
155
145
135
125
115
105
95
90
85
80
75
70
65
60
55
50
45
40
35
30
25
20
15
f1 (ppm)
Ph
N
Ts
Ph
N

3c

wyd-6-102 H CH2Cl2

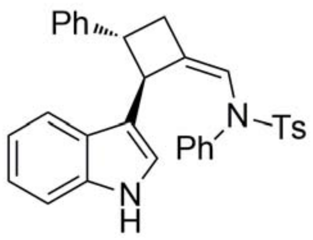

3d

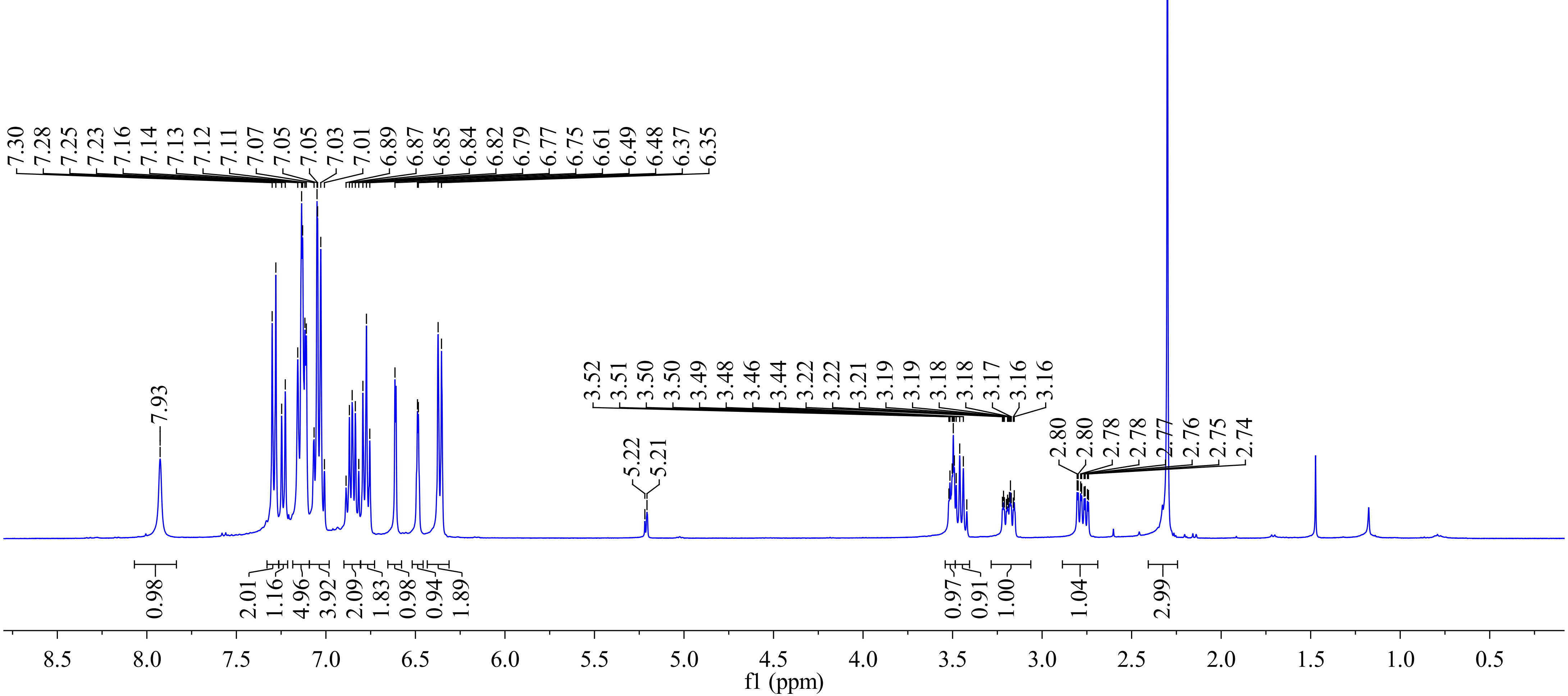

wyd-6-102 C CD2Cl2

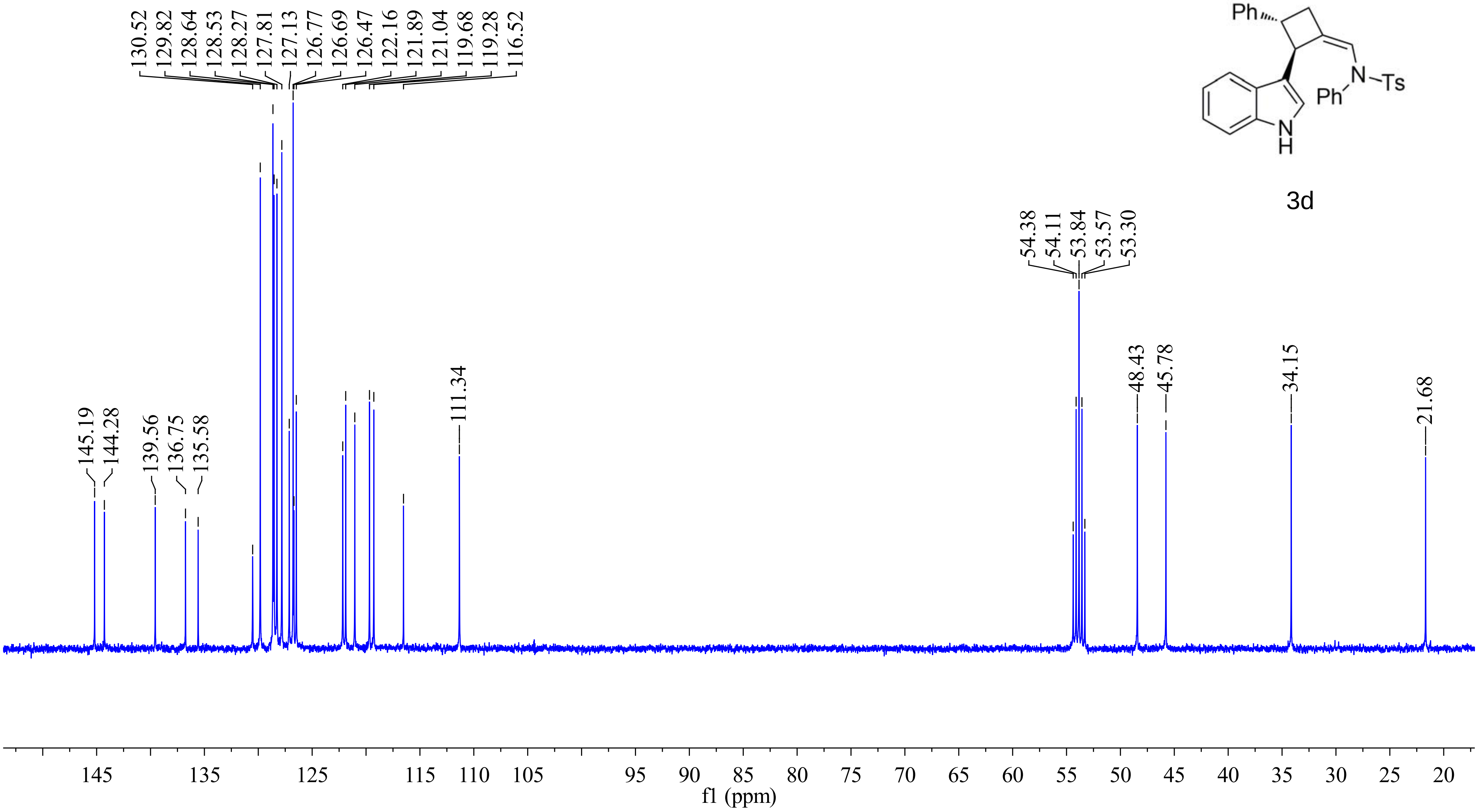

3d

wyd-6-22 H

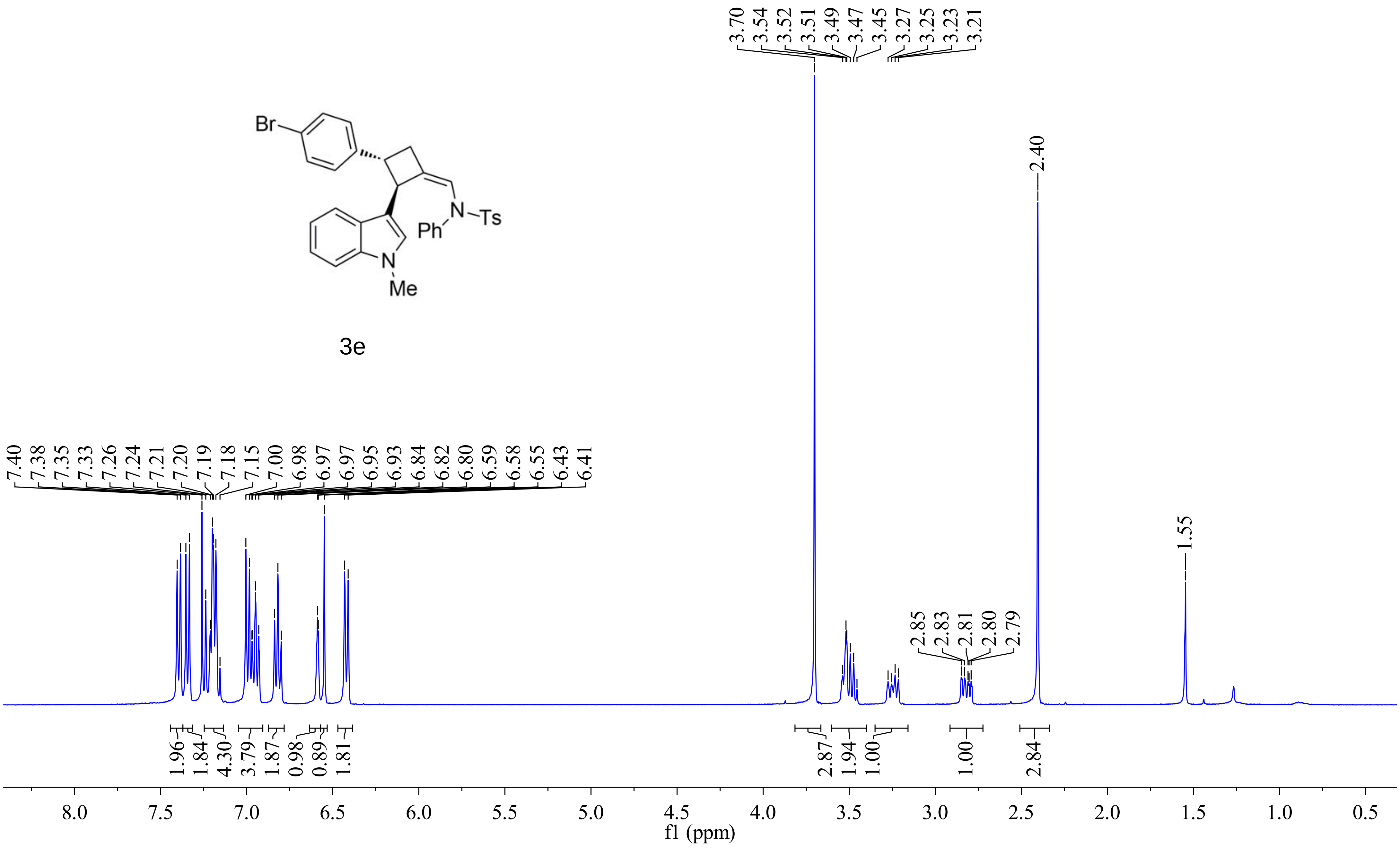
Br
N–Ts
Ph
N
Me
3e
7.40 7.38 7.35 7.33 7.26 7.24 7.21 7.20 7.19 7.18 7.15 7.00 6.98 6.97 6.97 6.95 6.93 6.84 6.82 6.80 6.59 6.58 6.55 6.43 6.41
3.70 3.54 3.52 3.51 3.49 3.47 3.45 3.27 3.25 3.23 3.21
2.85 2.83 2.81 2.80 2.79
2.40
1.55
1.96 1.84 4.30 3.79 1.87 0.98 0.89 1.81
2.87 1.94 1.00 1.00 2.84
8.0 7.5 7.0 6.5 6.0 5.5 5.0 4.5 4.0 3.5 3.0 2.5 2.0 1.5 1.0 0.5
fl (ppm)

wyd-6-22 C

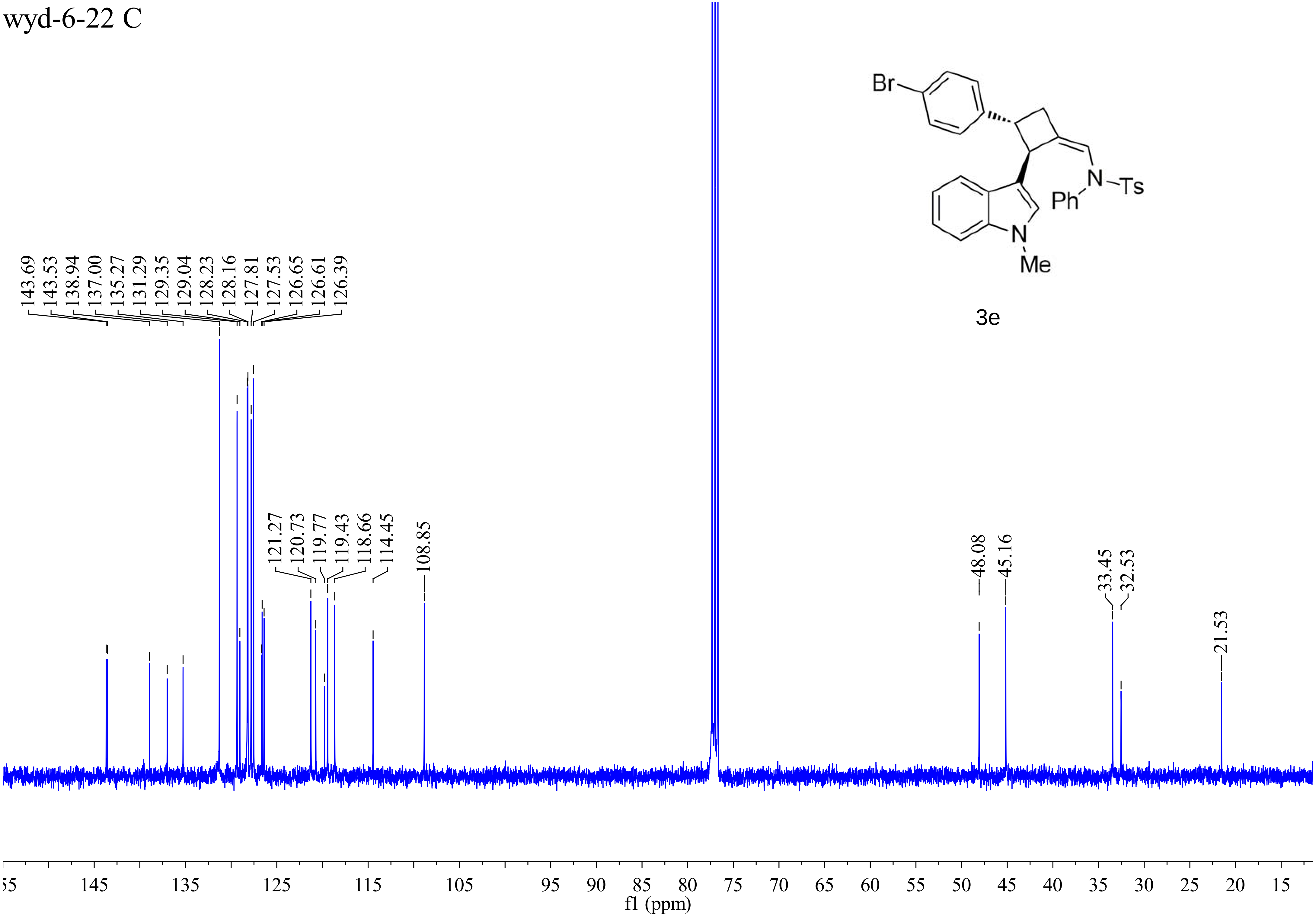

143.69
143.53
138.94
137.00
135.27
131.29
129.35
129.04
128.23
128.16
127.81
127.53
126.65
126.61
126.39
121.27
120.73
119.77
119.43
118.66
114.45
108.85
48.08
45.16
33.45
32.53
21.53
Br
N
Ts
Ph
N
Me
3e
fl (ppm)

wyd-6-15 H

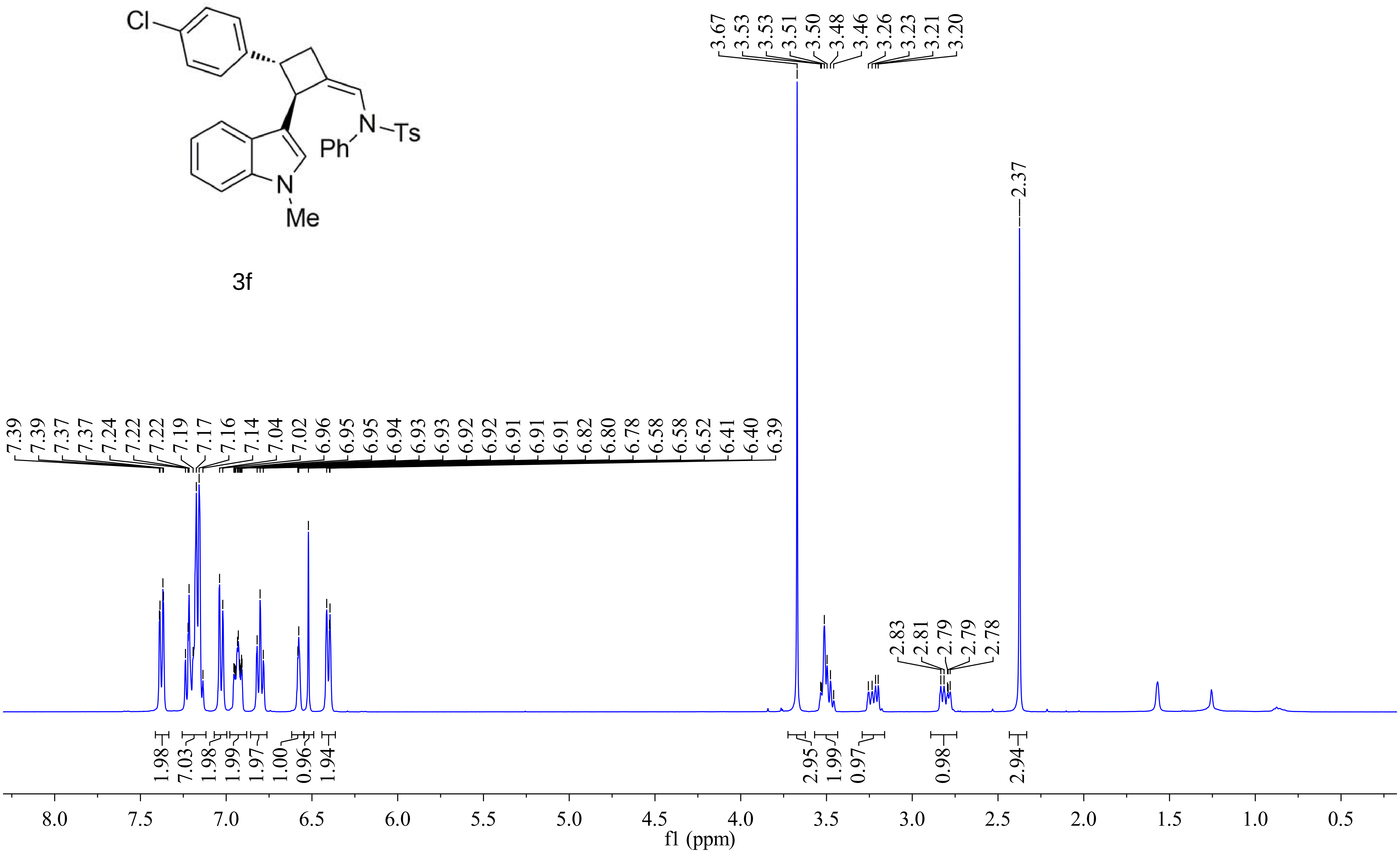

Cl
N
Ts
Ph
N
Me
3f
3.67
3.53
3.53
3.51
3.50
3.48
3.46
3.26
3.23
3.21
3.20
2.37
7.39
7.39
7.37
7.37
7.24
7.22
7.22
7.19
7.17
7.16
7.14
7.04
7.02
6.96
6.95
6.95
6.94
6.93
6.93
6.92
6.92
6.91
6.91
6.91
6.82
6.80
6.78
6.58
6.58
6.52
6.41
6.40
6.39
2.83
2.81
2.79
2.79
2.78
1.98
7.03
1.98
1.99
1.97
1.00
0.96
1.94
2.95
1.99
0.97
0.98
2.94
8.0
7.5
7.0
6.5
6.0
5.5
5.0
4.5
4.0
3.5
3.0
2.5
2.0
1.5
1.0
0.5
f1 (ppm)

wyd-6-15 C

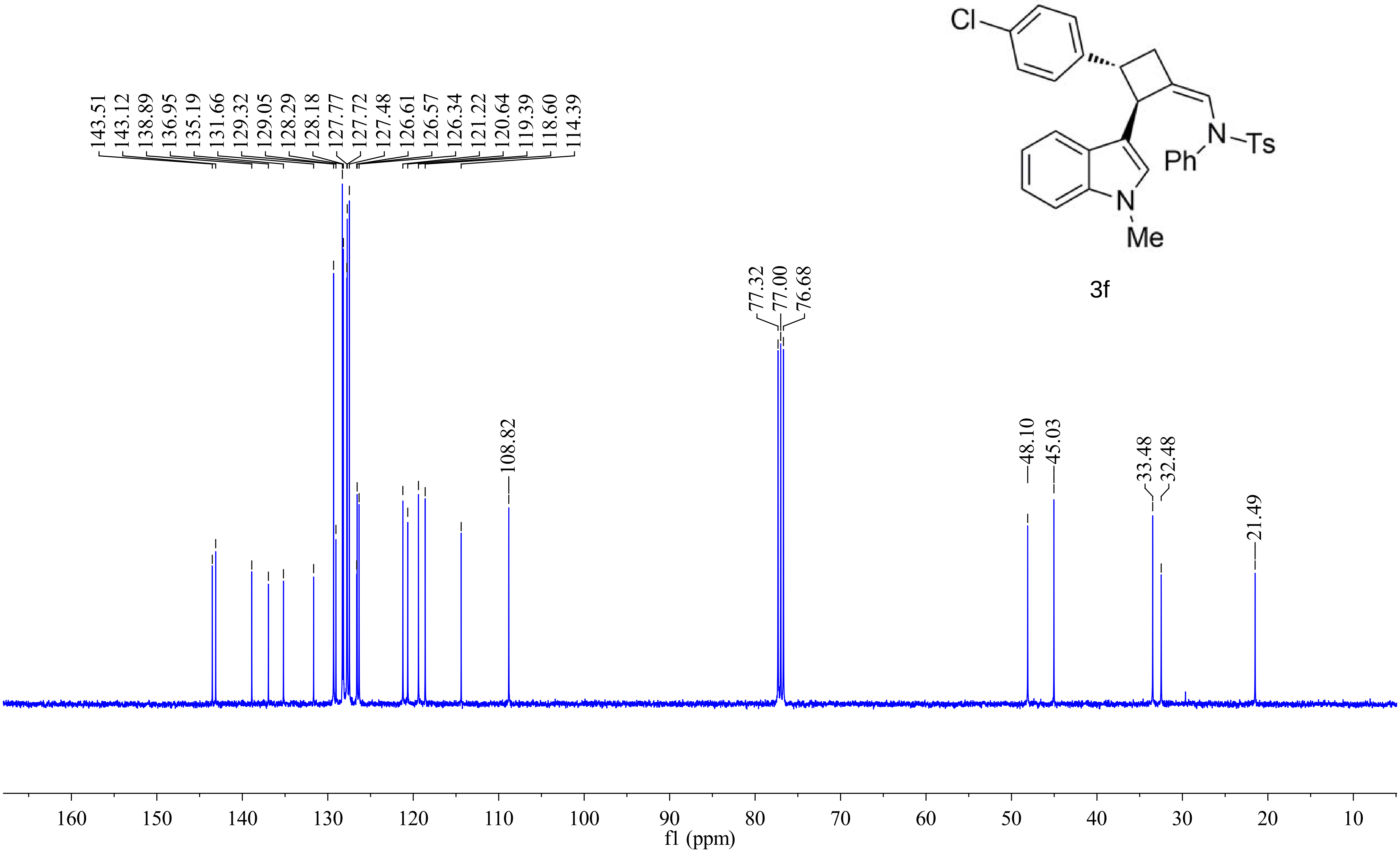
Cl
N
Ts
Ph
N
Me
143.51
143.12
138.89
136.95
135.19
131.66
129.32
129.05
128.29
128.18
127.77
127.72
127.48
126.61
126.57
126.34
121.22
120.64
119.39
118.60
114.39
108.82
77.32
77.00
76.68
48.10
45.03
33.48
32.48
21.49
160
150
140
130
120
110
100
90
80
70
60
50
40
30
20
10
f1 (ppm)

3f

wyd-6-21 H

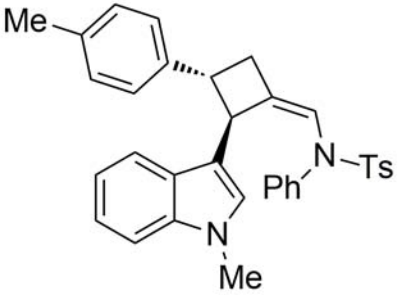
Me
N
Ts
Ph
N
Me

3g

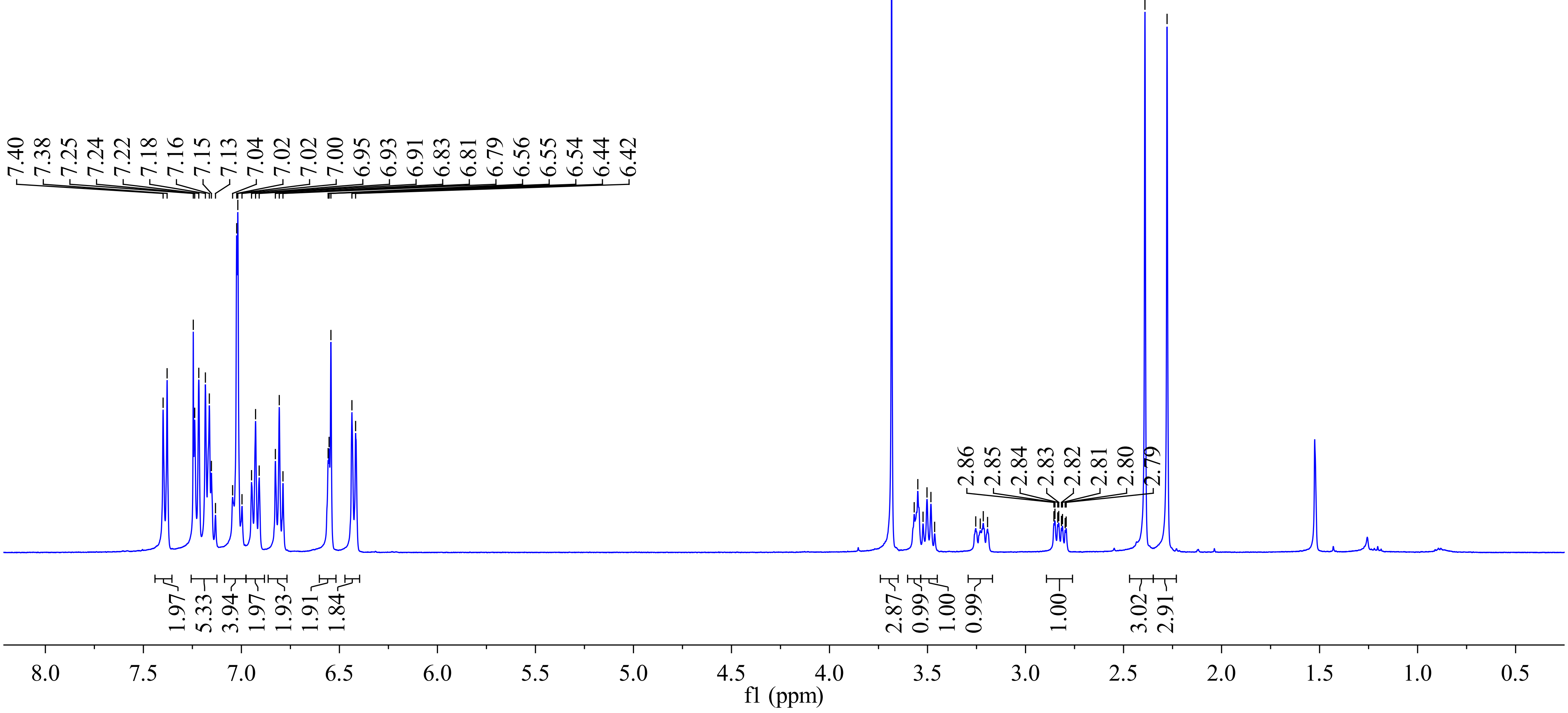
3.68 3.57 3.55 3.52 3.50 3.48 3.46 3.25 3.23 3.22 3.19
2.39 2.28
7.40 7.38 7.25 7.24 7.22 7.18 7.16 7.15 7.13 7.04 7.02 7.02 7.00 6.95 6.93 6.91 6.83 6.81 6.79 6.56 6.55 6.54 6.44 6.42
2.86 2.85 2.84 2.83 2.82 2.81 2.80 2.79
1.97 5.33 3.94 1.97 1.93 1.91 1.84
2.87 0.99 1.00 0.99 1.00 3.02 2.91
8.0 7.5 7.0 6.5 6.0 5.5 5.0 4.5 4.0 3.5 3.0 2.5 2.0 1.5 1.0 0.5
f1 (ppm)

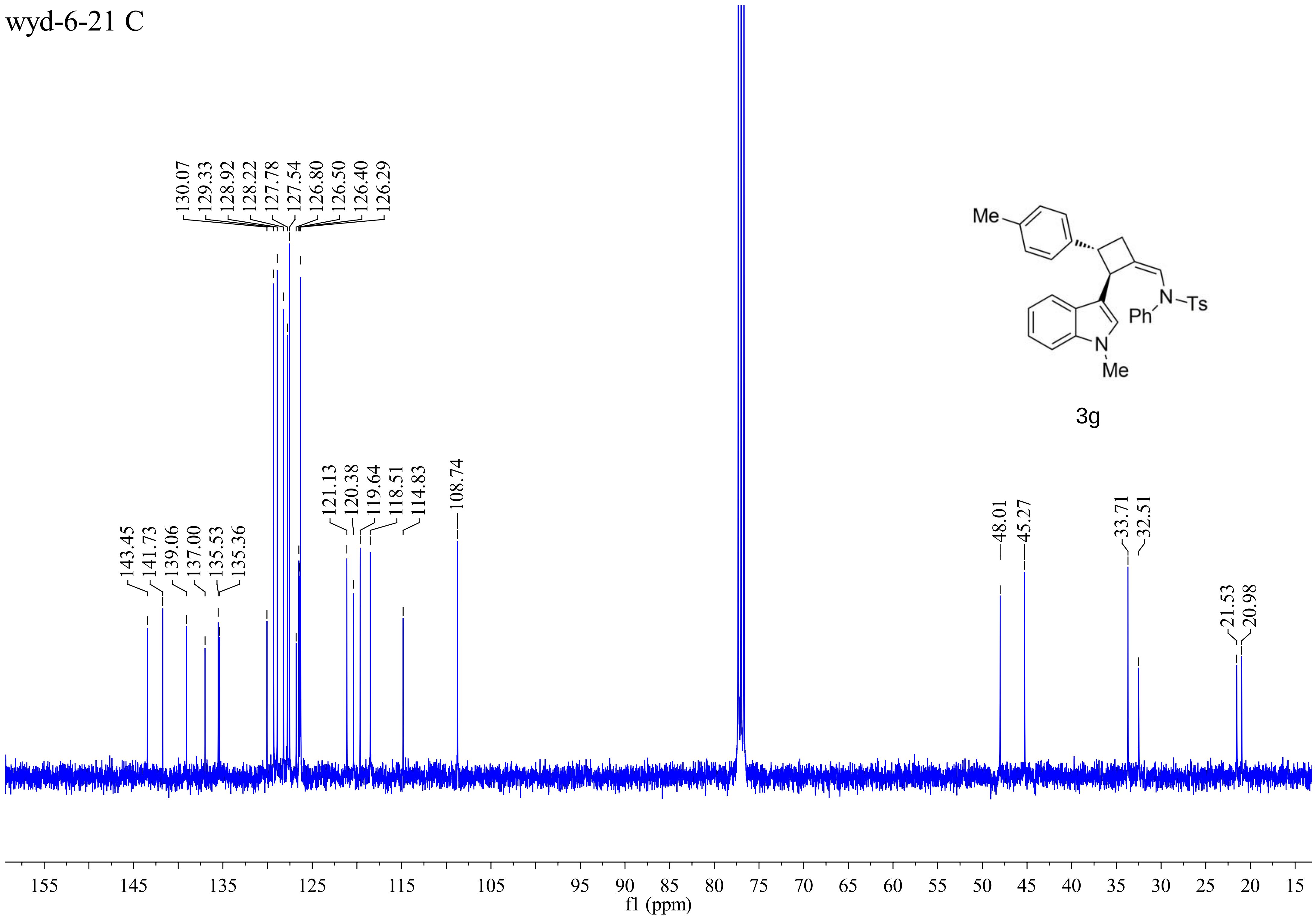
wyd-6-21 C
130.07
129.33
128.92
128.22
127.78
127.54
126.80
126.50
126.40
126.29
143.45
141.73
139.06
137.00
135.53
135.36
121.13
120.38
119.64
118.51
114.83
108.74
48.01
45.27
33.71
32.51
21.53
20.98
Me
N
Ts
Ph
N
Me
3g
155 145 135 125 115 105 95 90 85 80 75 70 65 60 55 50 45 40 35 30 25 20 15
f1 (ppm)

wyd-6-14 H

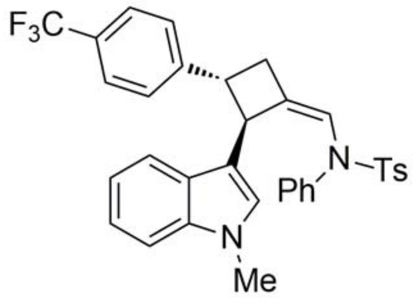
F3C
N
Ts
Ph
N
Me

3h

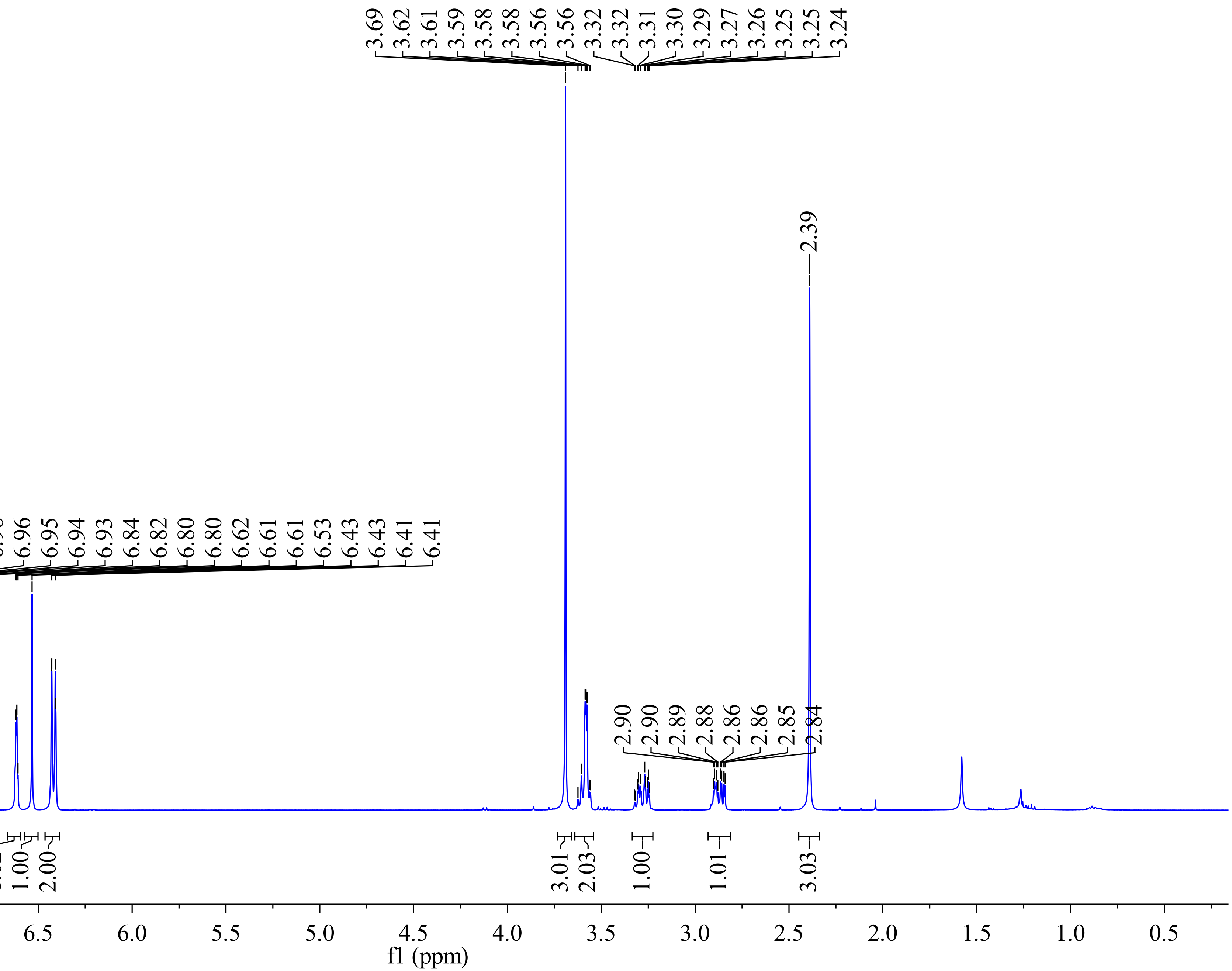
3.69
3.62
3.61
3.59
3.58
3.58
3.56
3.56
3.32
3.32
3.31
3.30
3.29
3.27
3.26
3.25
3.25
3.24
2.39
7.48
7.46
7.40
7.38
7.26
7.24
7.23
7.22
7.19
7.18
7.17
7.16
6.98
6.98
6.96
6.95
6.94
6.93
6.84
6.82
6.80
6.80
6.62
6.61
6.61
6.53
6.43
6.43
6.41
6.41
2.90
2.90
2.89
2.88
2.86
2.86
2.85
2.84
2.03
2.05
6.76
2.05
2.02
1.02
1.00
2.00
3.01
2.03
1.00
1.01
3.03
8.5
8.0
7.5
7.0
6.5
6.0
5.5
5.0
4.5
4.0
3.5
3.0
2.5
2.0
1.5
1.0
0.5
f1 (ppm)

wyd-6-14 F

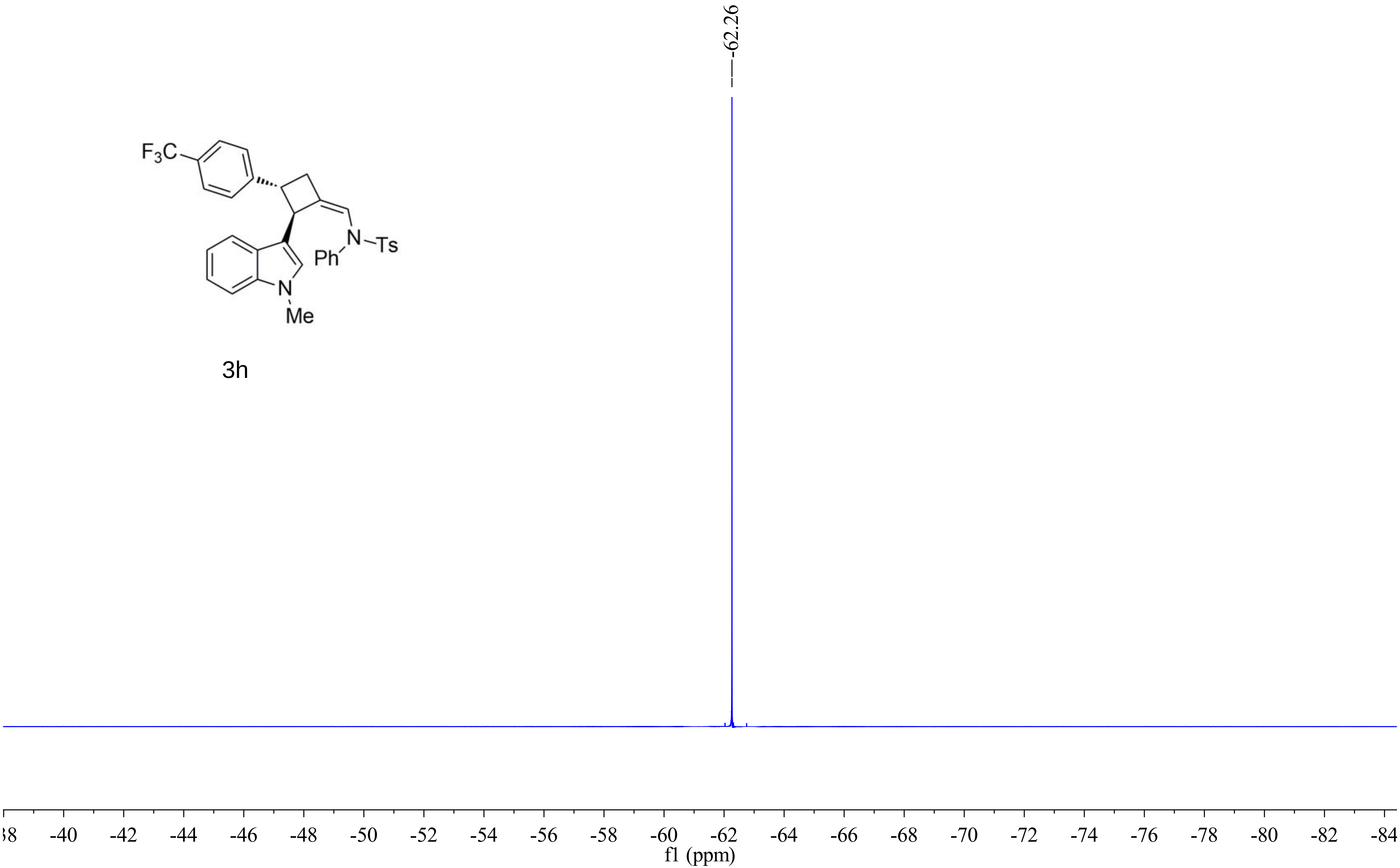

-62.26
-38
-40
-42
-44
-46
-48
-50
-52
-54
-56
-58
-60
-62
-64
-66
-68
-70
-72
-74
-76
-78
-80
-82
-84
f1 (ppm)

3h

wyd-6-14 C

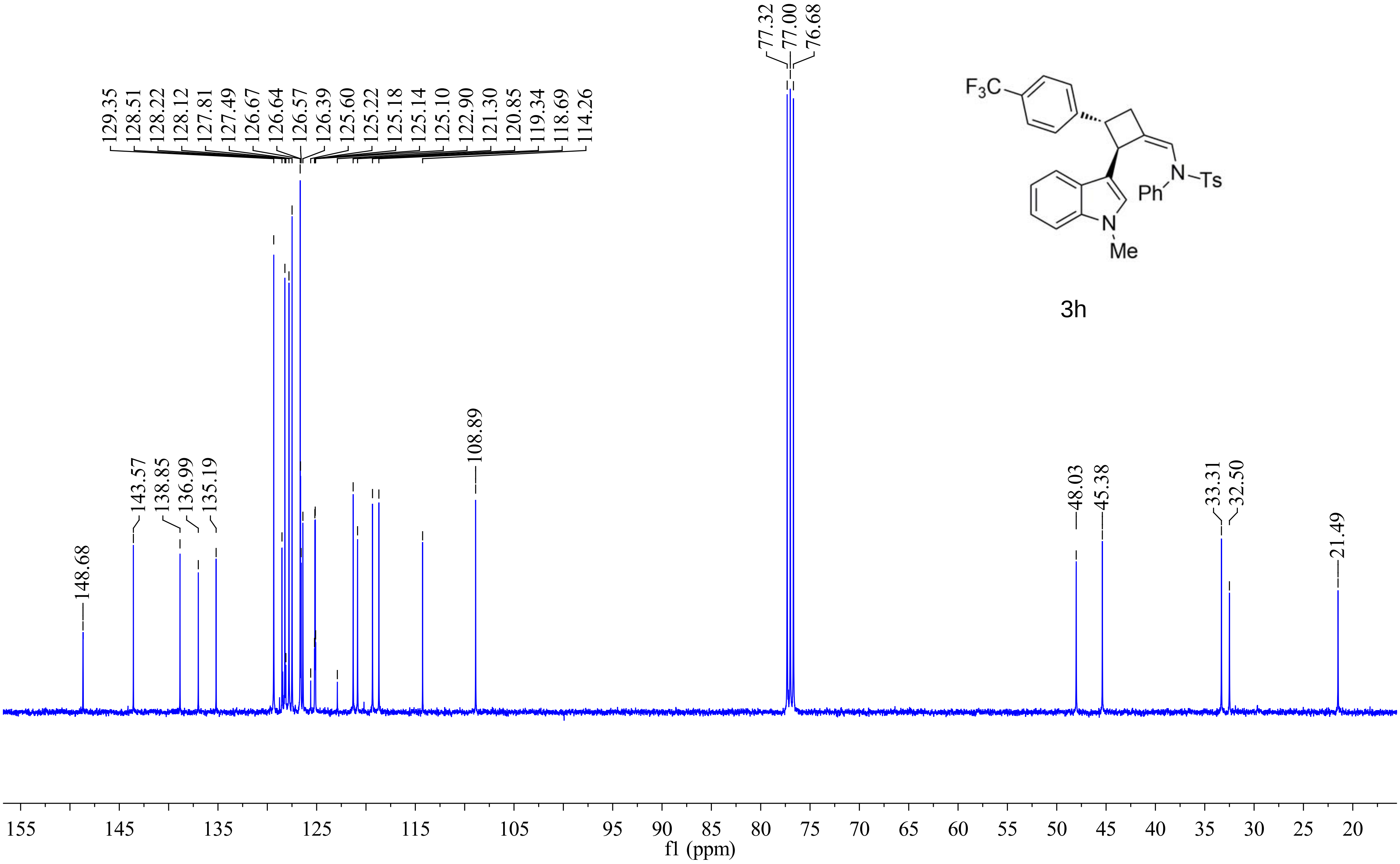

77.32
77.00
76.68
129.35
128.51
128.22
128.12
127.81
127.49
126.67
126.64
126.57
126.39
125.60
125.22
125.18
125.14
125.10
122.90
121.30
120.85
119.34
118.69
114.26
108.89
148.68
143.57
138.85
136.99
135.19
48.03
45.38
33.31
32.50
21.49
155 145 135 125 115 105 95 90 85 80 75 70 65 60 55 50 45 40 35 30 25 20
f1 (ppm)
F3C
Ph
N
Ts
N
Me

3h

wyd-6-16 H

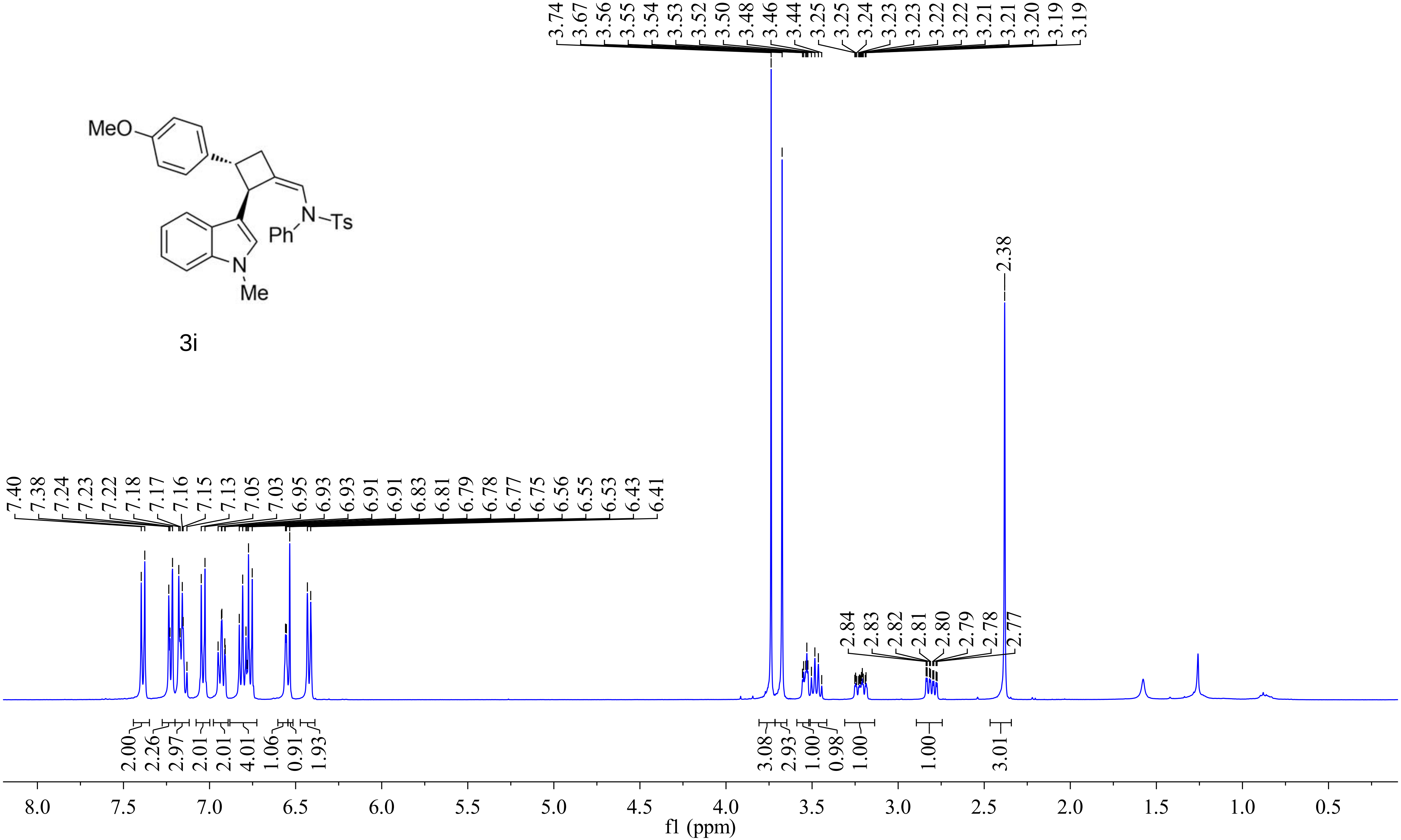

3.74 3.67 3.56 3.55 3.54 3.53 3.52 3.50 3.48 3.46 3.44 3.25 3.25 3.24 3.23 3.23 3.22 3.22 3.21 3.21 3.20 3.19 3.19
MeO
N
Ts
Ph
N
Me
3i
2.38
7.40 7.38 7.24 7.23 7.22 7.18 7.17 7.16 7.15 7.13 7.05 7.03 6.95 6.93 6.93 6.91 6.91 6.83 6.81 6.79 6.78 6.77 6.75 6.56 6.55 6.53 6.43 6.41
2.84 2.83 2.82 2.81 2.80 2.79 2.78 2.77
2.00 2.26 2.97 2.01 2.01 4.01 1.06 0.91 1.93
3.08 2.93 1.00 0.98 1.00
1.00
3.01
8.0 7.5 7.0 6.5 6.0 5.5 5.0 4.5 4.0 3.5 3.0 2.5 2.0 1.5 1.0 0.5
f1 (ppm)

wyd-6-16 C

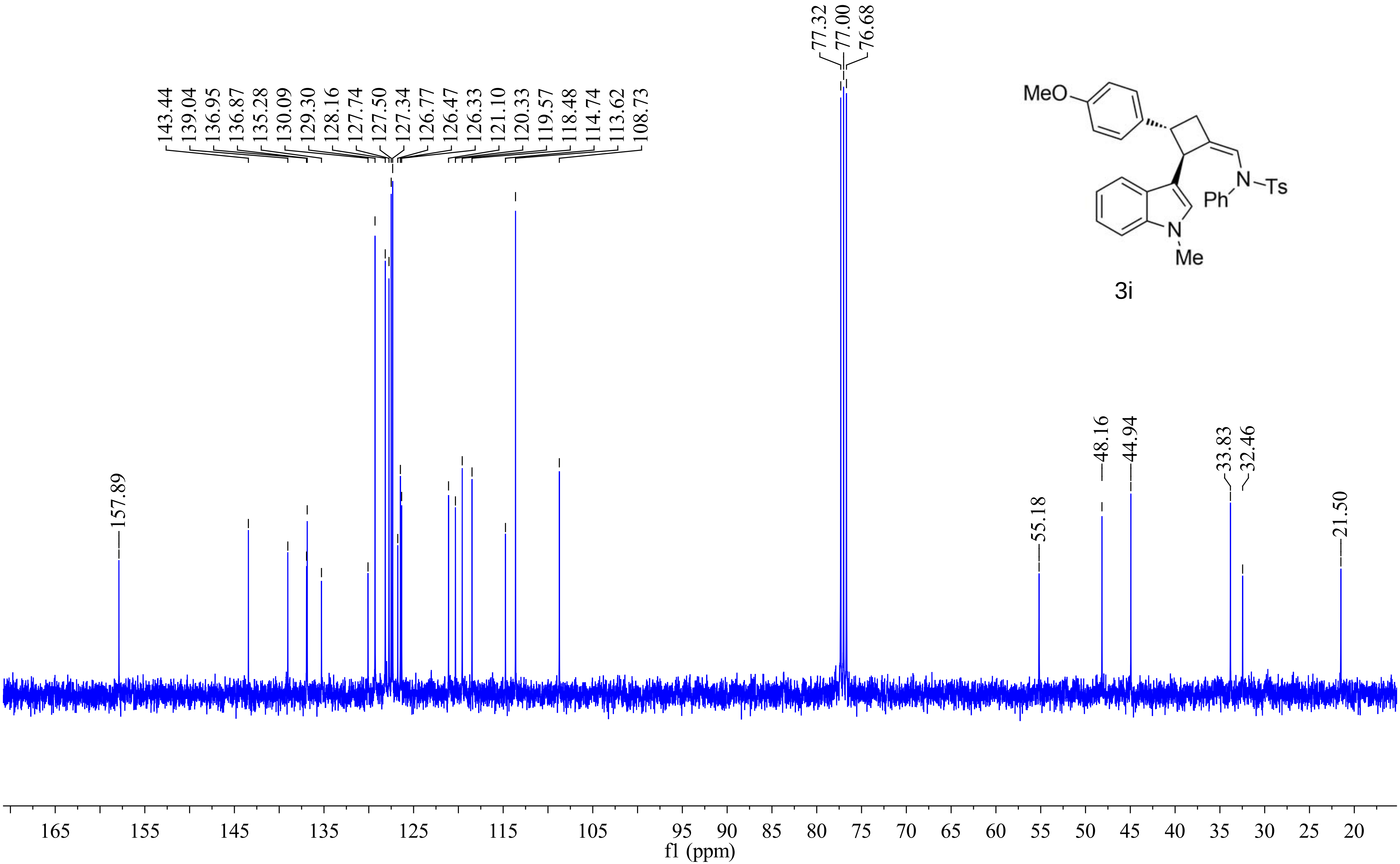
143.44
139.04
136.95
136.87
135.28
130.09
129.30
128.16
127.74
127.50
127.34
126.77
126.47
126.33
121.10
120.33
119.57
118.48
114.74
113.62
108.73
77.32
77.00
76.68
157.89
55.18
48.16
44.94
33.83
32.46
21.50
165 155 145 135 125 115 105 95 90 85 80 75 70 65 60 55 50 45 40 35 30 25 20
f1 (ppm)
MeO
N
Ts
Ph
N
Me

3i

wyd-6-17 H

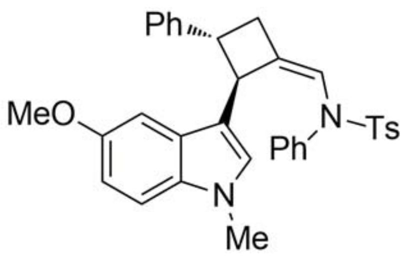

Ph
MeO
N
Ts
Ph
N
Me

3j

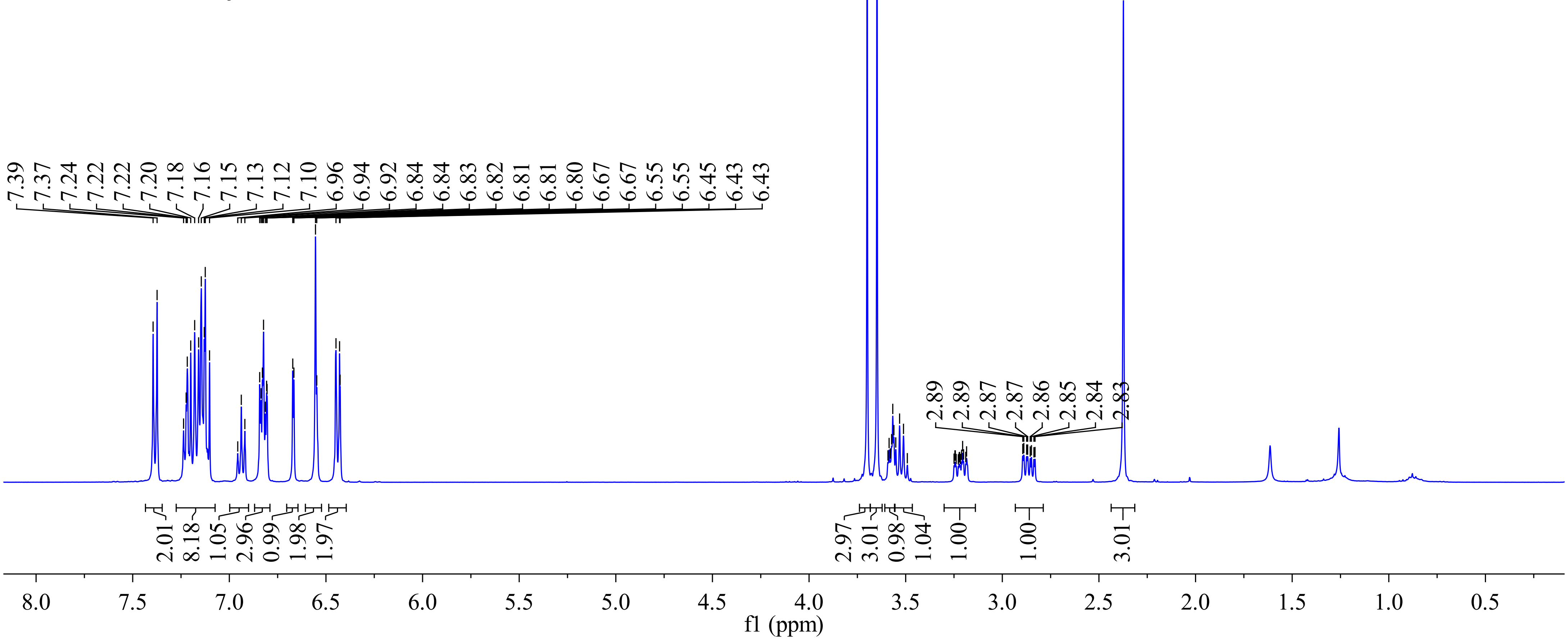

7.39 7.37 7.24 7.22 7.22 7.20 7.18 7.16 7.15 7.13 7.12 7.10 6.96 6.94 6.92 6.84 6.84 6.83 6.82 6.81 6.81 6.80 6.67 6.67 6.55 6.55 6.45 6.43 6.43
3.70 3.65 3.59 3.59 3.58 3.57 3.57 3.56 3.55 3.53 3.51 3.49 3.25 3.25 3.24 3.24 3.23 3.22 3.22 3.22 3.21 3.21 3.20 3.19 3.18
2.89 2.89 2.87 2.87 2.86 2.85 2.84 2.83
2.37
2.01 8.18 1.05 2.96 0.99 1.98 1.97 2.97 3.01 0.98 1.04 1.00 1.00 3.01
8.0 7.5 7.0 6.5 6.0 5.5 5.0 4.5 4.0 3.5 3.0 2.5 2.0 1.5 1.0 0.5
f1 (ppm)

wyd-6-17 C

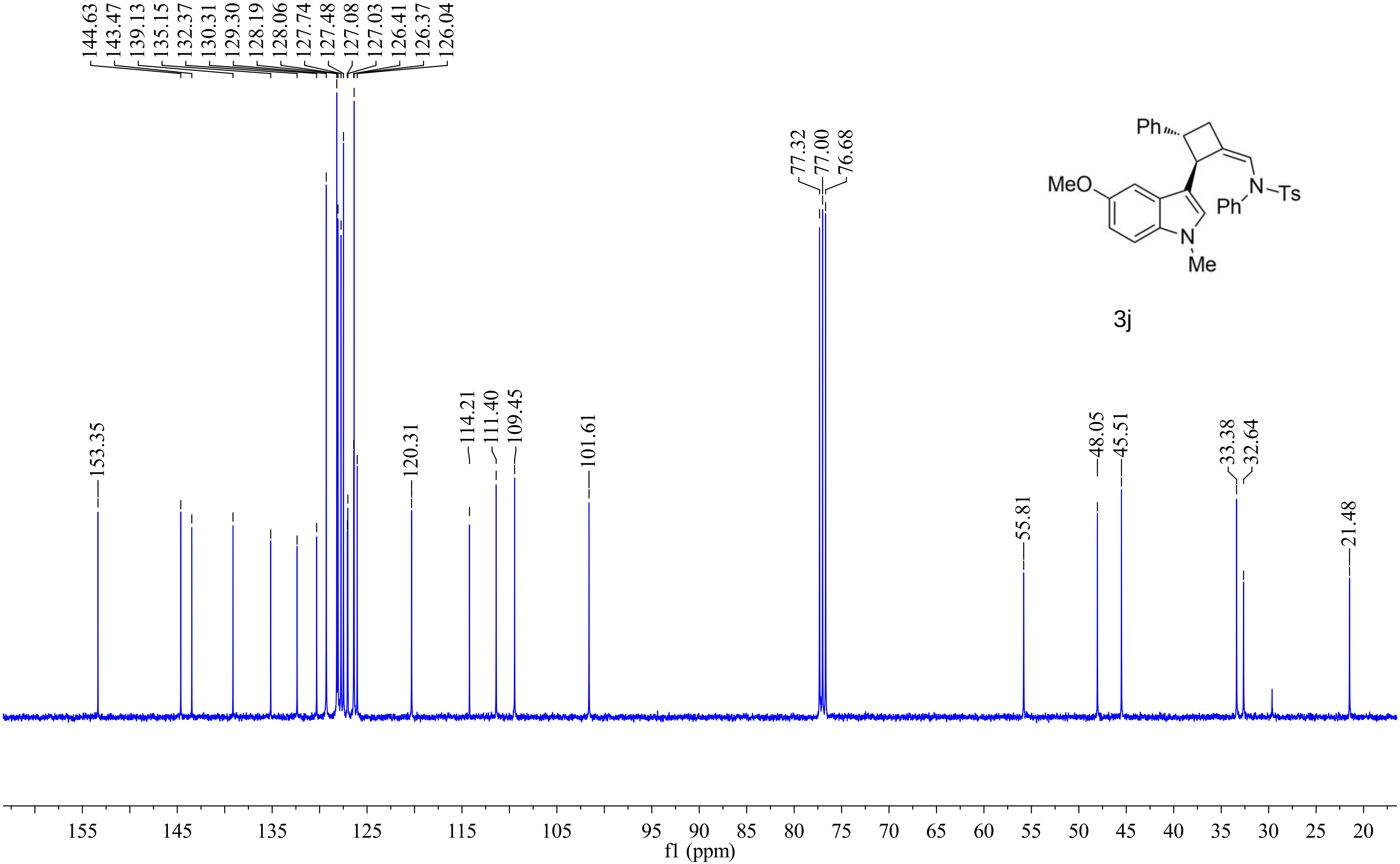

144.63
143.47
139.13
135.15
132.37
130.31
129.30
128.19
128.06
127.74
127.48
127.08
127.03
126.41
126.37
126.04
153.35
120.31
114.21
111.40
109.45
101.61
77.32
77.00
76.68
55.81
48.05
45.51
33.38
32.64
21.48
Ph
MeO
N
Ts
Ph
N
Me
3j
155
145
135
125
115
105
95
90
85
80
75
70
65
60
55
50
45
40
35
30
25
20
f1 (ppm)

wyd-6-23 H

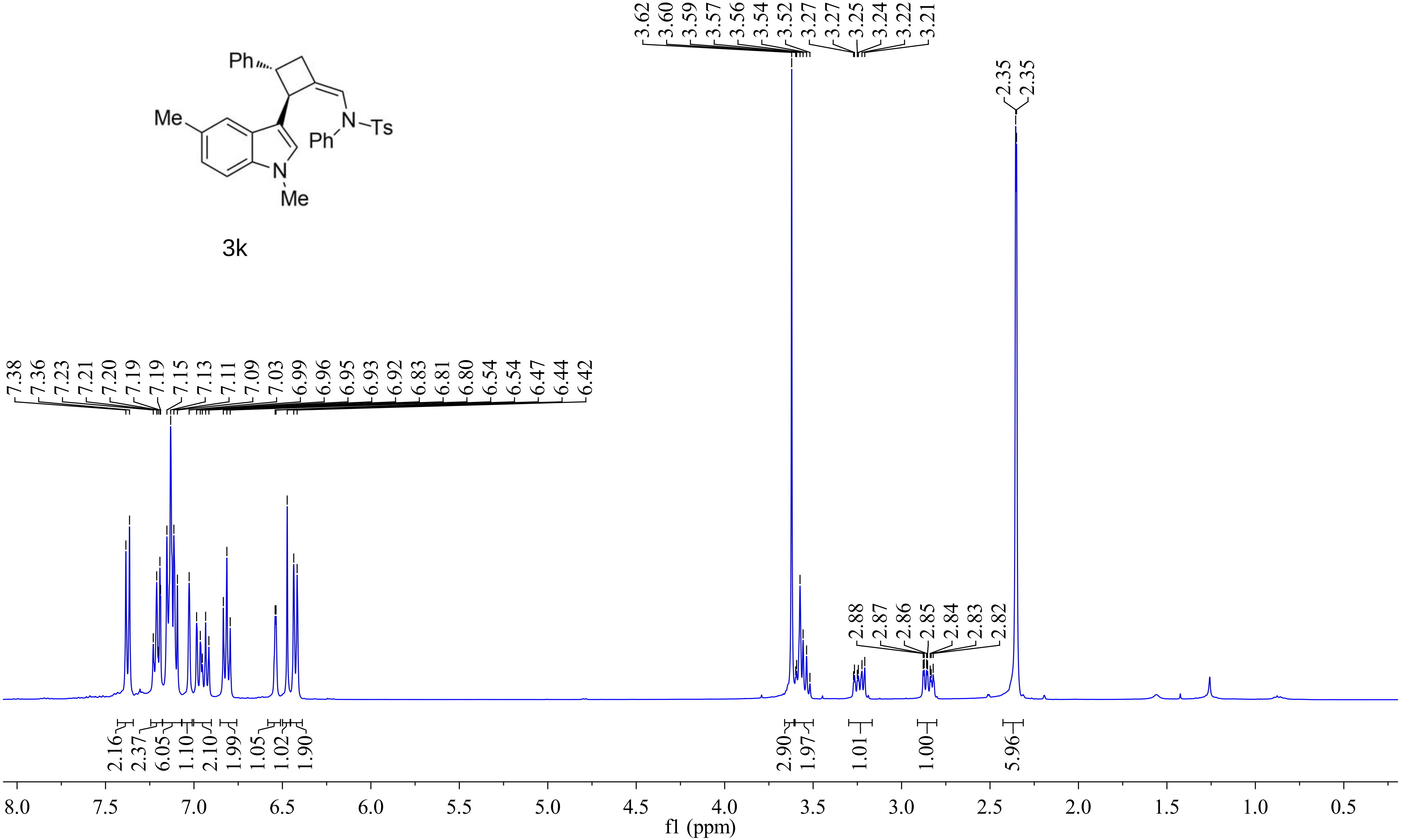
Ph
Me
N
Ts
Ph
N
Me
3k
7.38
7.36
7.23
7.21
7.20
7.19
7.19
7.15
7.13
7.11
7.09
7.03
6.99
6.96
6.95
6.93
6.92
6.83
6.81
6.80
6.54
6.54
6.47
6.44
6.42
3.62
3.60
3.59
3.57
3.56
3.54
3.52
3.27
3.27
3.25
3.24
3.22
3.21
2.88
2.87
2.86
2.85
2.84
2.83
2.82
2.35
2.35
2.16
2.37
6.05
1.10
2.10
1.99
1.05
1.02
1.90
2.90
1.97
1.01
1.00
5.96
8.0
7.5
7.0
6.5
6.0
5.5
5.0
4.5
4.0
3.5
3.0
2.5
2.0
1.5
1.0
0.5
f1 (ppm)

wyd-6-23 C

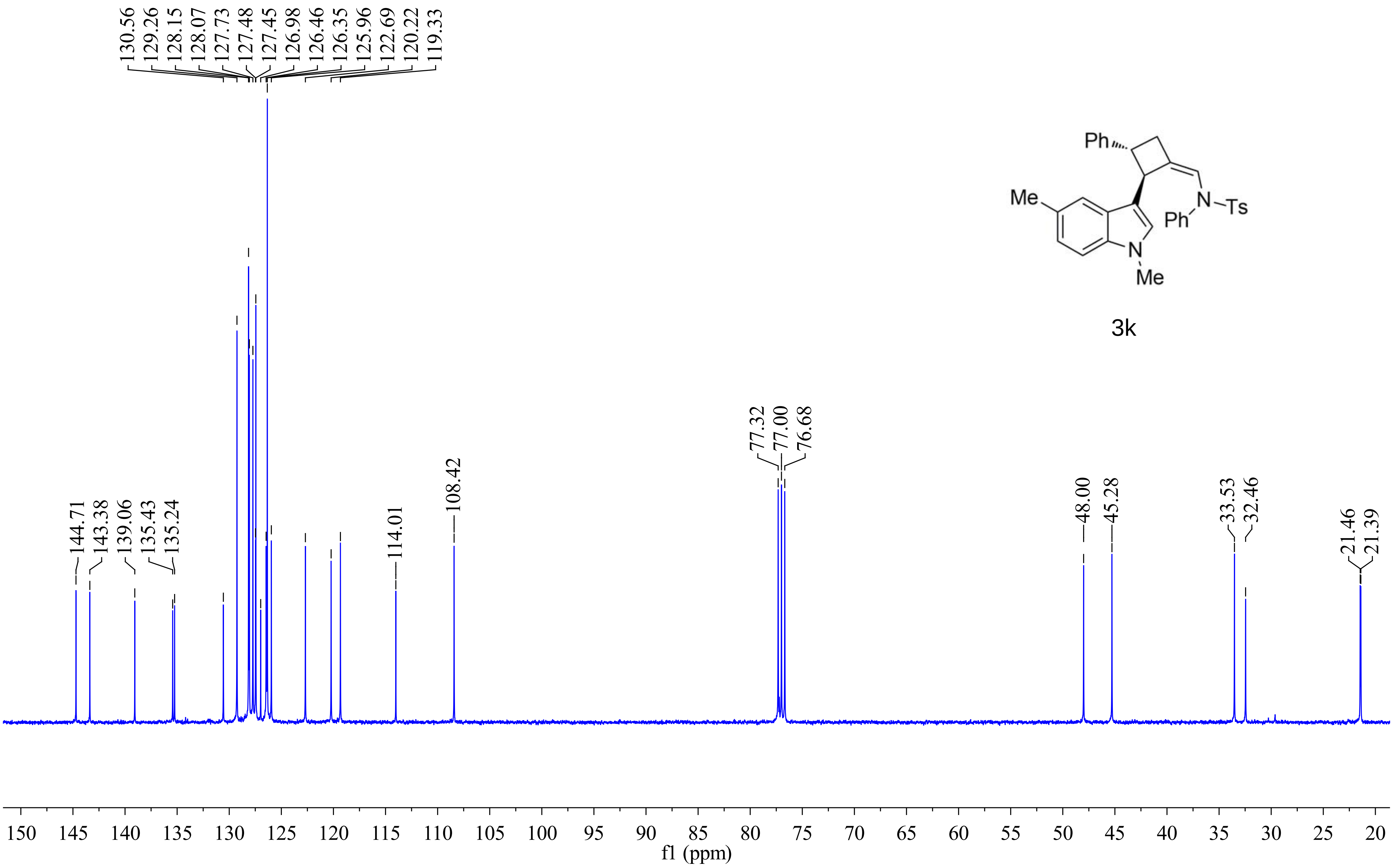
130.56
129.26
128.15
128.07
127.73
127.48
127.45
126.98
126.46
126.35
125.96
122.69
120.22
119.33
144.71
143.38
139.06
135.43
135.24
114.01
108.42
77.32
77.00
76.68
48.00
45.28
33.53
32.46
21.46
21.39
Ph
Me
N
Ts
Ph
N
Me
3k
150 145 140 135 130 125 120 115 110 105 100 95 90 85 80 75 70 65 60 55 50 45 40 35 30 25 20
f1 (ppm)

wyd-6-18 H

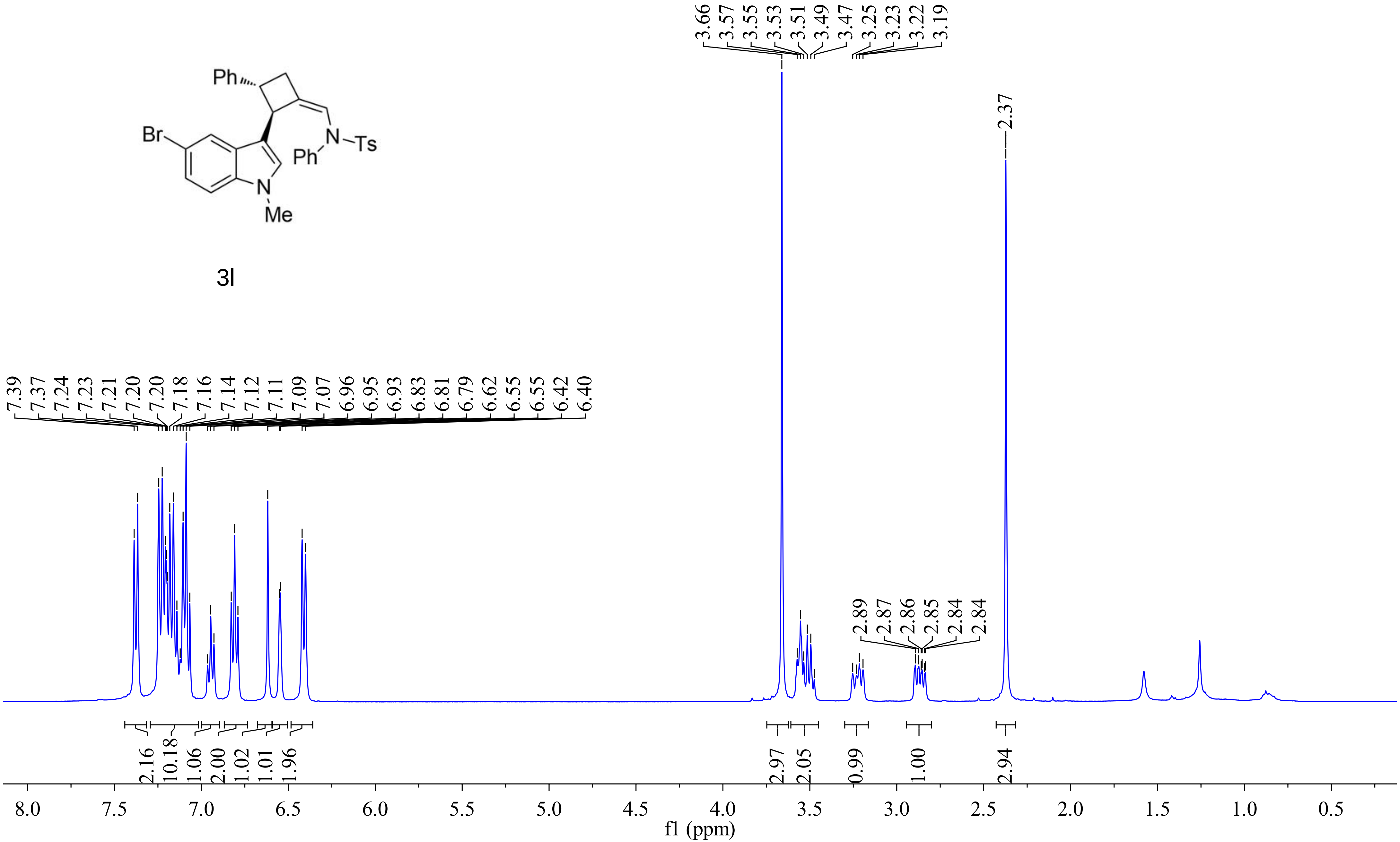
Ph
Br
N
Ts
Ph
N
Me
3l
7.39 7.37 7.24 7.23 7.21 7.20 7.20 7.18 7.16 7.14 7.12 7.11 7.09 7.07 6.96 6.95 6.93 6.83 6.81 6.79 6.62 6.55 6.55 6.42 6.40
3.66 3.57 3.55 3.53 3.51 3.49 3.47 3.25 3.23 3.22 3.19
2.89 2.87 2.86 2.85 2.84 2.84
2.37
2.16 10.18 1.06 2.00 1.02 1.01 1.96 2.97 2.05 0.99 1.00 2.94
8.0 7.5 7.0 6.5 6.0 5.5 5.0 4.5 4.0 3.5 3.0 2.5 2.0 1.5 1.0 0.5
f1 (ppm)

wyd-6-18 C

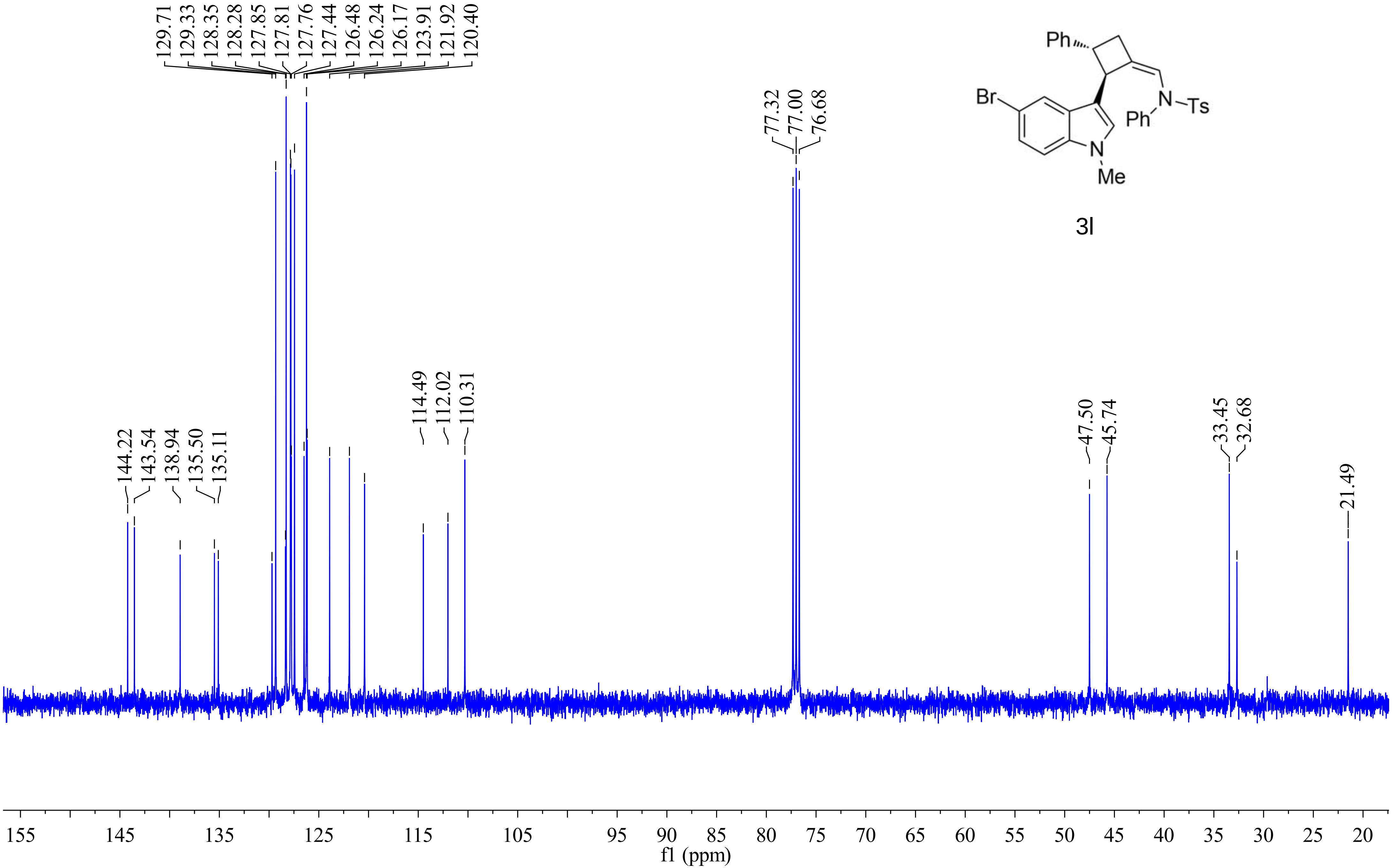
129.71
129.33
128.35
128.28
127.85
127.81
127.76
127.44
126.48
126.24
126.17
123.91
121.92
120.40
77.32
77.00
76.68
144.22
143.54
138.94
135.50
135.11
114.49
112.02
110.31
47.50
45.74
33.45
32.68
21.49
Ph
Br
N
Ts
Ph
N
Me
3l
155
145
135
125
115
105
95
90
85
80
75
70
65
60
55
50
45
40
35
30
25
20
f1 (ppm)

wyd-6-74 H

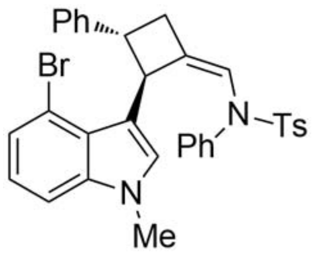

3m

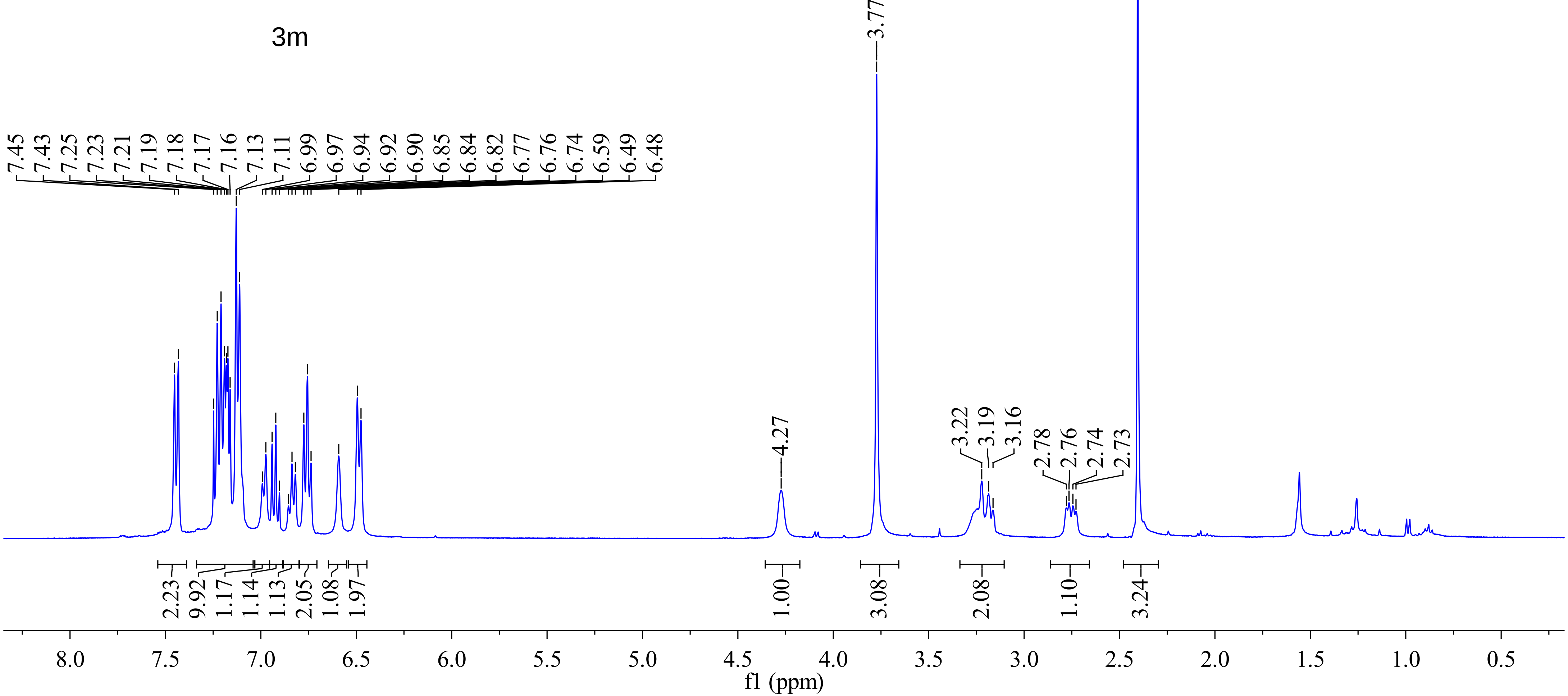

7.45 7.43 7.25 7.23 7.21 7.19 7.18 7.17 7.16 7.13 7.11 6.99 6.97 6.94 6.92 6.90 6.85 6.84 6.82 6.77 6.76 6.74 6.59 6.49 6.48
4.27
3.77
3.22 3.19 3.16
2.78 2.76 2.74 2.73
2.40
2.23 9.92 1.17 1.14 1.13 2.05 1.08 1.97
1.00
3.08
2.08
1.10
3.24
8.0 7.5 7.0 6.5 6.0 5.5 5.0 4.5 4.0 3.5 3.0 2.5 2.0 1.5 1.0 0.5
f1 (ppm)

wyd-6-74 C

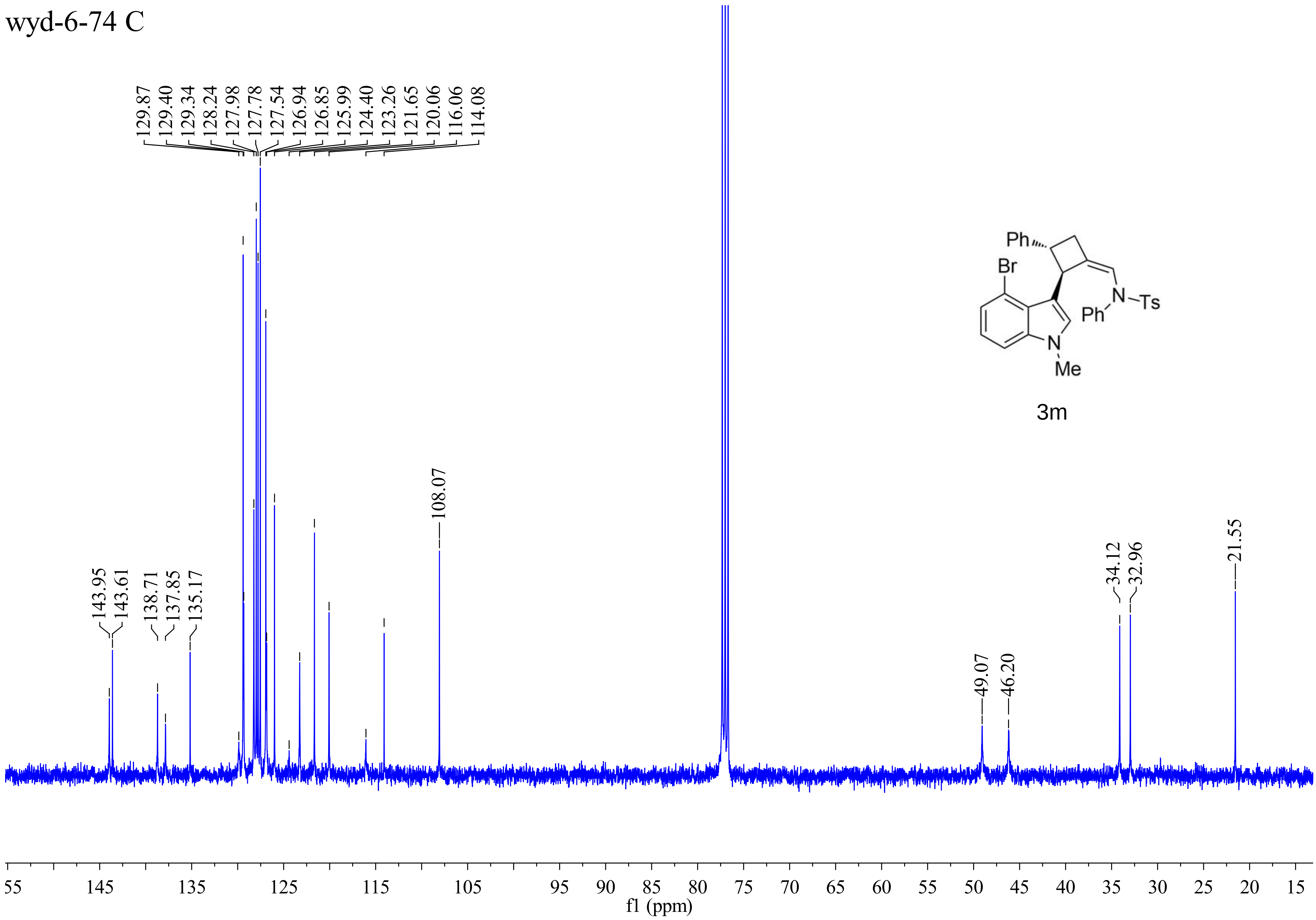

129.87
129.40
129.34
128.24
127.98
127.78
127.54
126.94
126.85
125.99
124.40
123.26
121.65
120.06
116.06
114.08
143.95
143.61
138.71
137.85
135.17
108.07
49.07
46.20
34.12
32.96
21.55
Ph
Br
N
Ts
Ph
N
Me
3m
fl (ppm)

wyd-6-144 H

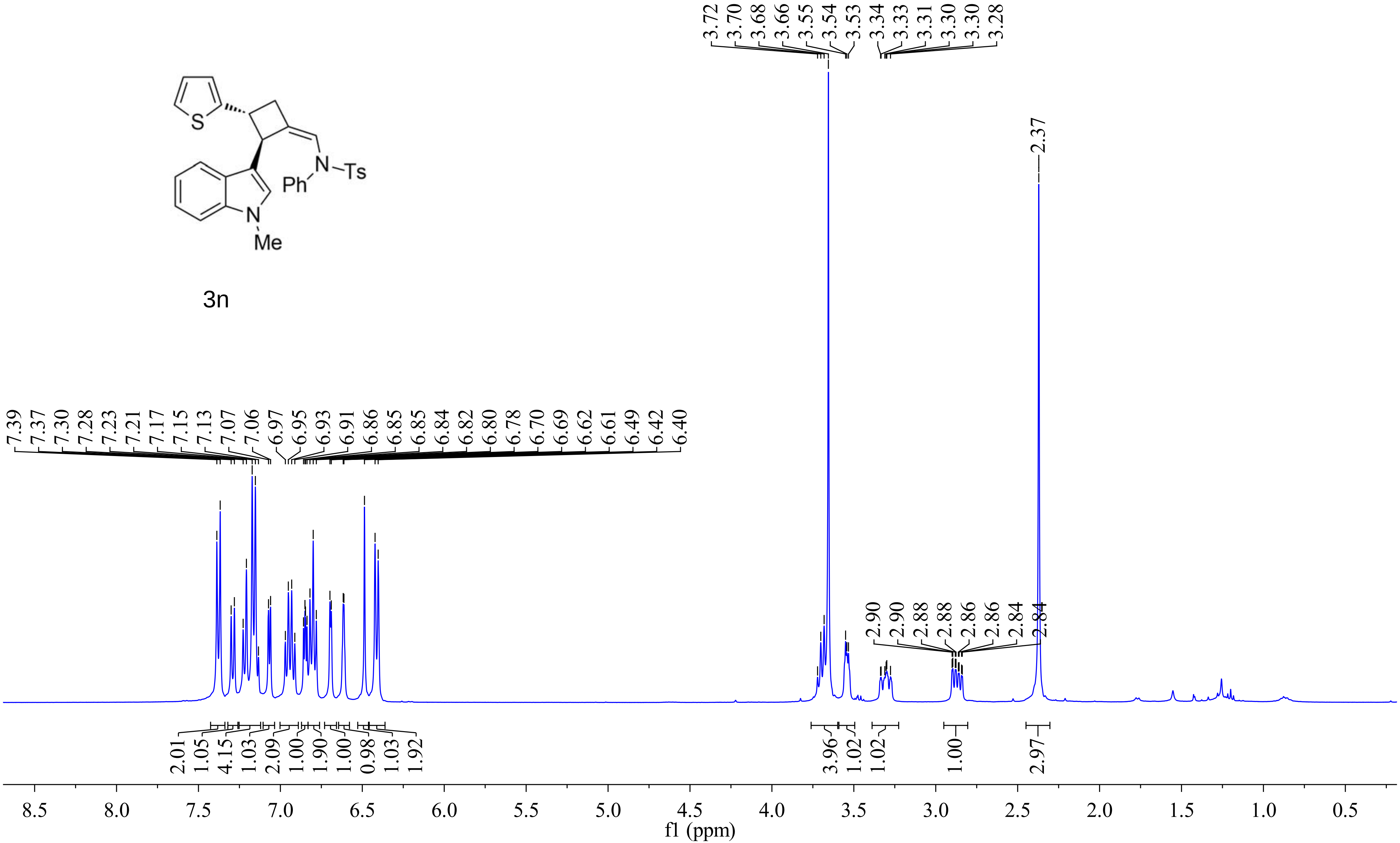
S
N
Ts
Ph
N
Me
7.39 7.37 7.30 7.28 7.23 7.21 7.17 7.15 7.13 7.07 7.06 6.97 6.95 6.93 6.91 6.86 6.85 6.85 6.84 6.82 6.80 6.78 6.70 6.69 6.62 6.61 6.49 6.42 6.40
3.72 3.70 3.68 3.66 3.55 3.54 3.53 3.34 3.33 3.31 3.30 3.30 3.28
2.37
2.90 2.90 2.88 2.88 2.86 2.86 2.84 2.84
2.01 1.05 4.15 1.03 2.09 1.00 1.90 1.00 0.98 1.03 1.92
3.96 1.02 1.02 1.00 2.97
8.5 8.0 7.5 7.0 6.5 6.0 5.5 5.0 4.5 4.0 3.5 3.0 2.5 2.0 1.5 1.0 0.5
f1 (ppm)

3n

wyd-6-144 C

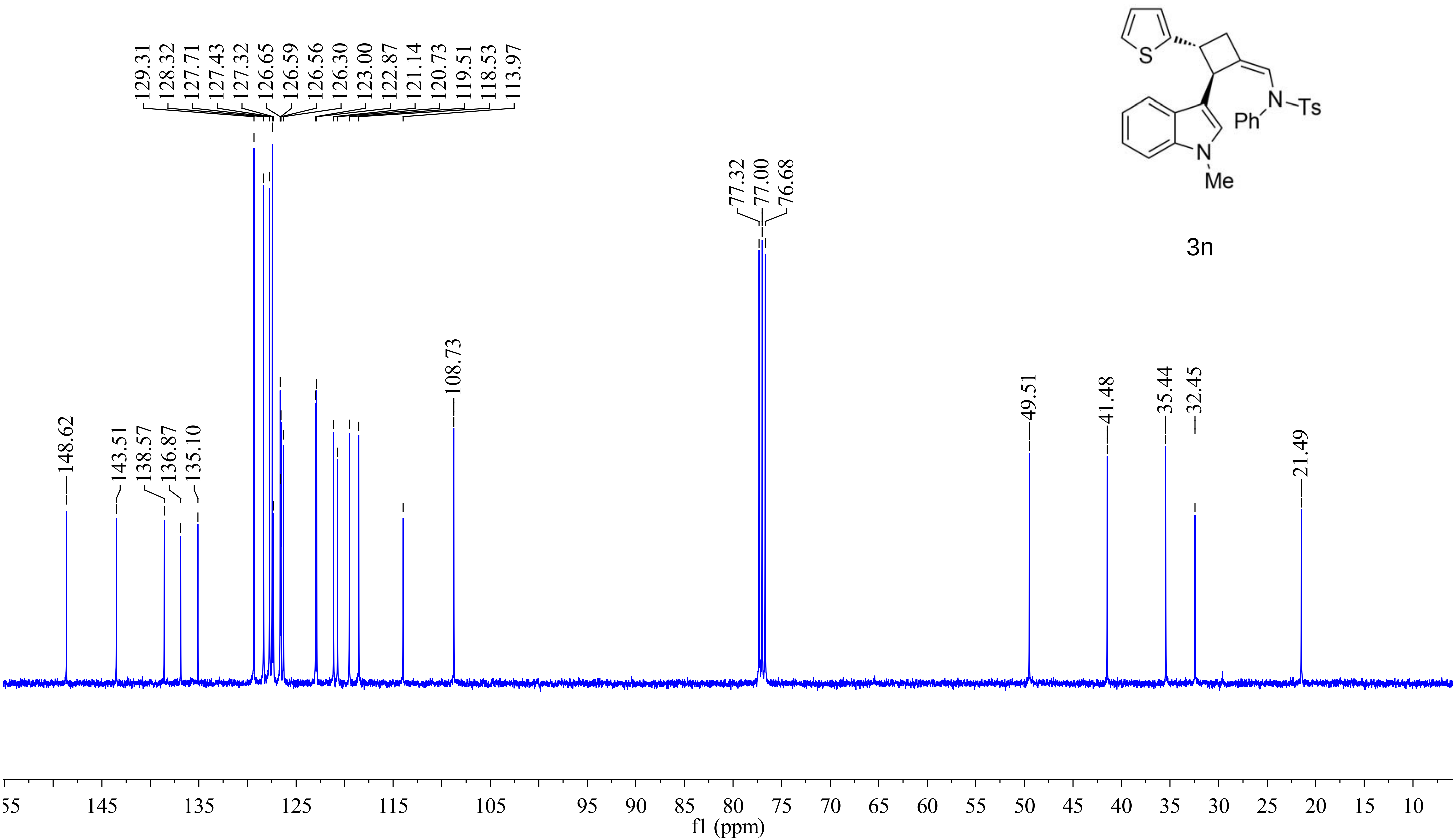

148.62
143.51
138.57
136.87
135.10
129.31
128.32
127.71
127.43
127.32
126.65
126.59
126.56
126.30
123.00
122.87
121.14
120.73
119.51
118.53
113.97
108.73
77.32
77.00
76.68
49.51
41.48
35.44
32.45
21.49
55 145 135 125 115 105 95 90 85 80 75 70 65 60 55 50 45 40 35 30 25 20 15 10
f1 (ppm)
S
N
Ts
Ph
N
Me

3n

wyd-6-48 H

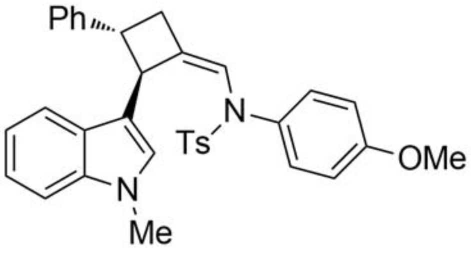
Ph
Ts
N
OMe
N
Me

3q

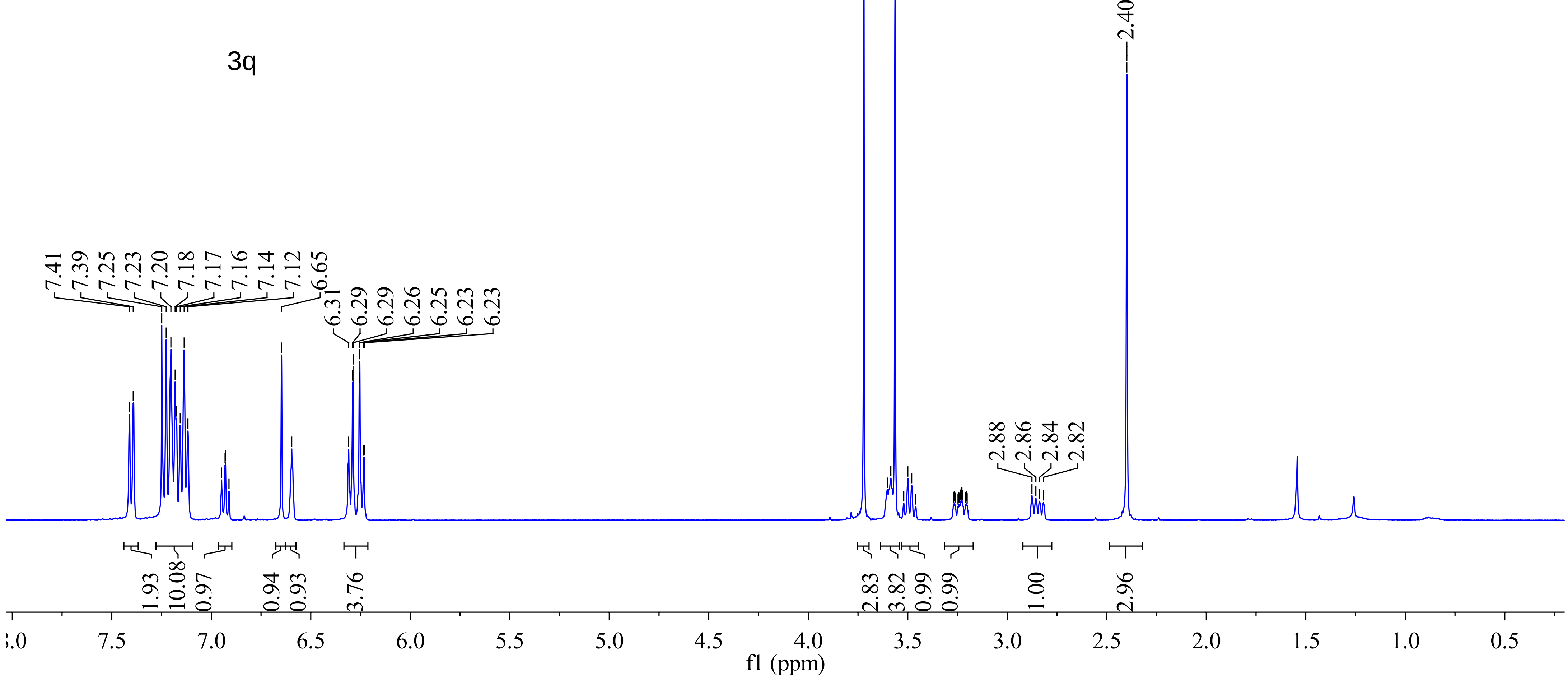
3.72
3.60
3.59
3.56
3.52
3.50
3.48
3.46
3.27
3.27
3.26
3.25
3.24
3.24
3.24
3.23
3.23
3.22
3.21
3.21
3.20
2.40
7.41
7.39
7.25
7.23
7.20
7.18
7.17
7.16
7.14
7.12
6.65
6.31
6.29
6.29
6.26
6.25
6.23
6.23
2.88
2.86
2.84
2.82
1.93
10.08
0.97
0.94
0.93
3.76
2.83
3.82
0.99
0.99
1.00
2.96
8.0
7.5
7.0
6.5
6.0
5.5
5.0
4.5
4.0
3.5
3.0
2.5
2.0
1.5
1.0
0.5
f1 (ppm)

wyd-6-48 C

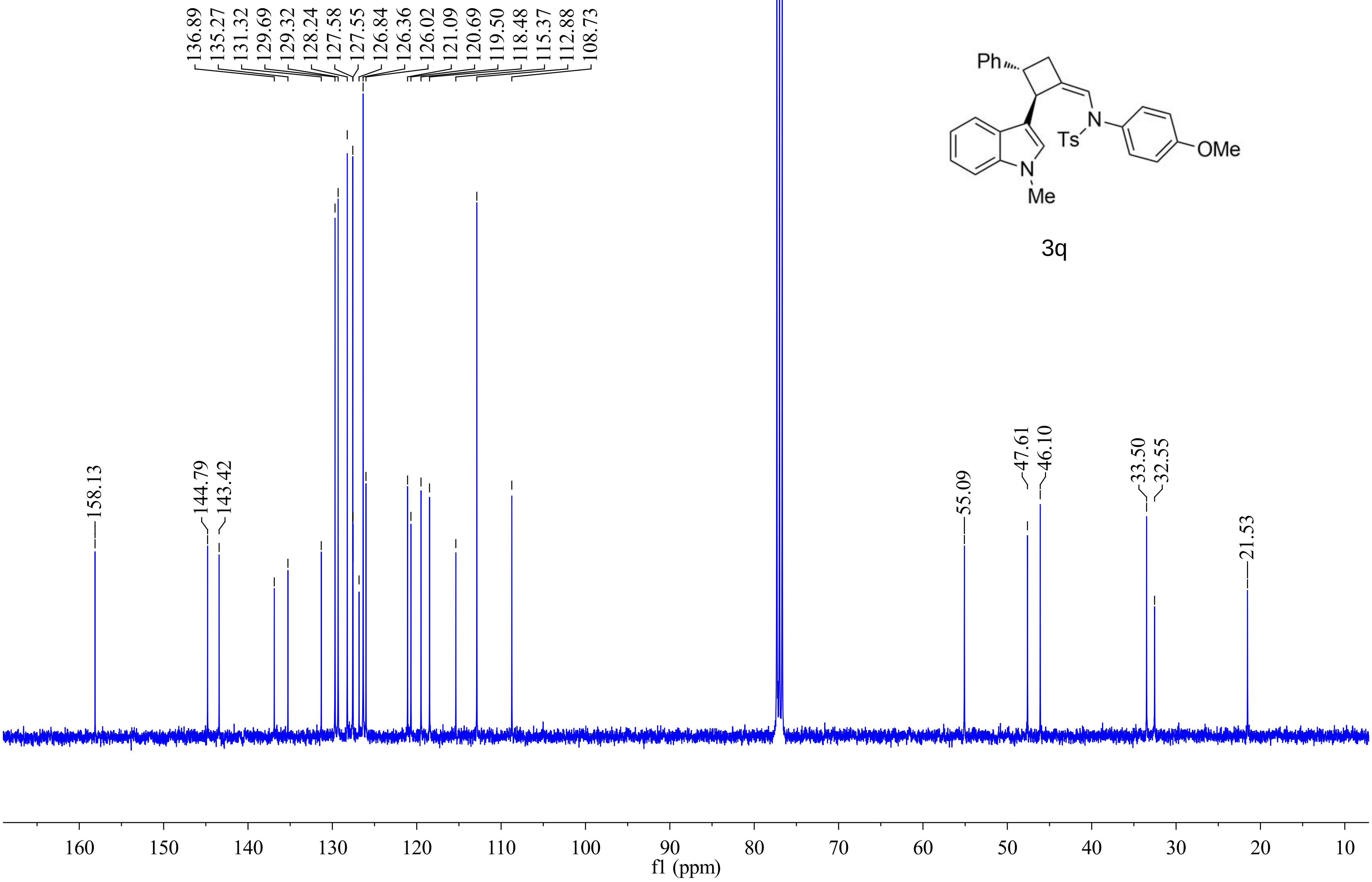
Ph
N
Ts
OMe
N
Me
3q
136.89
135.27
131.32
129.69
129.32
128.24
127.58
127.55
126.84
126.36
126.02
121.09
120.69
119.50
118.48
115.37
112.88
108.73
158.13
144.79
143.42
55.09
47.61
46.10
33.50
32.55
21.53
160
150
140
130
120
110
100
90
80
70
60
50
40
30
20
10
f1 (ppm)

wyd-6-137 H

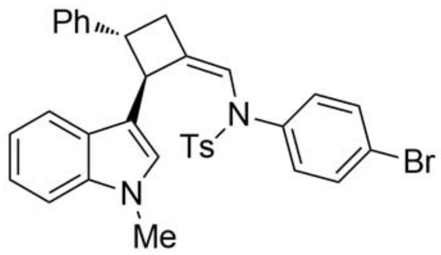

Ph
Ts
N
Br
N
Me

3r

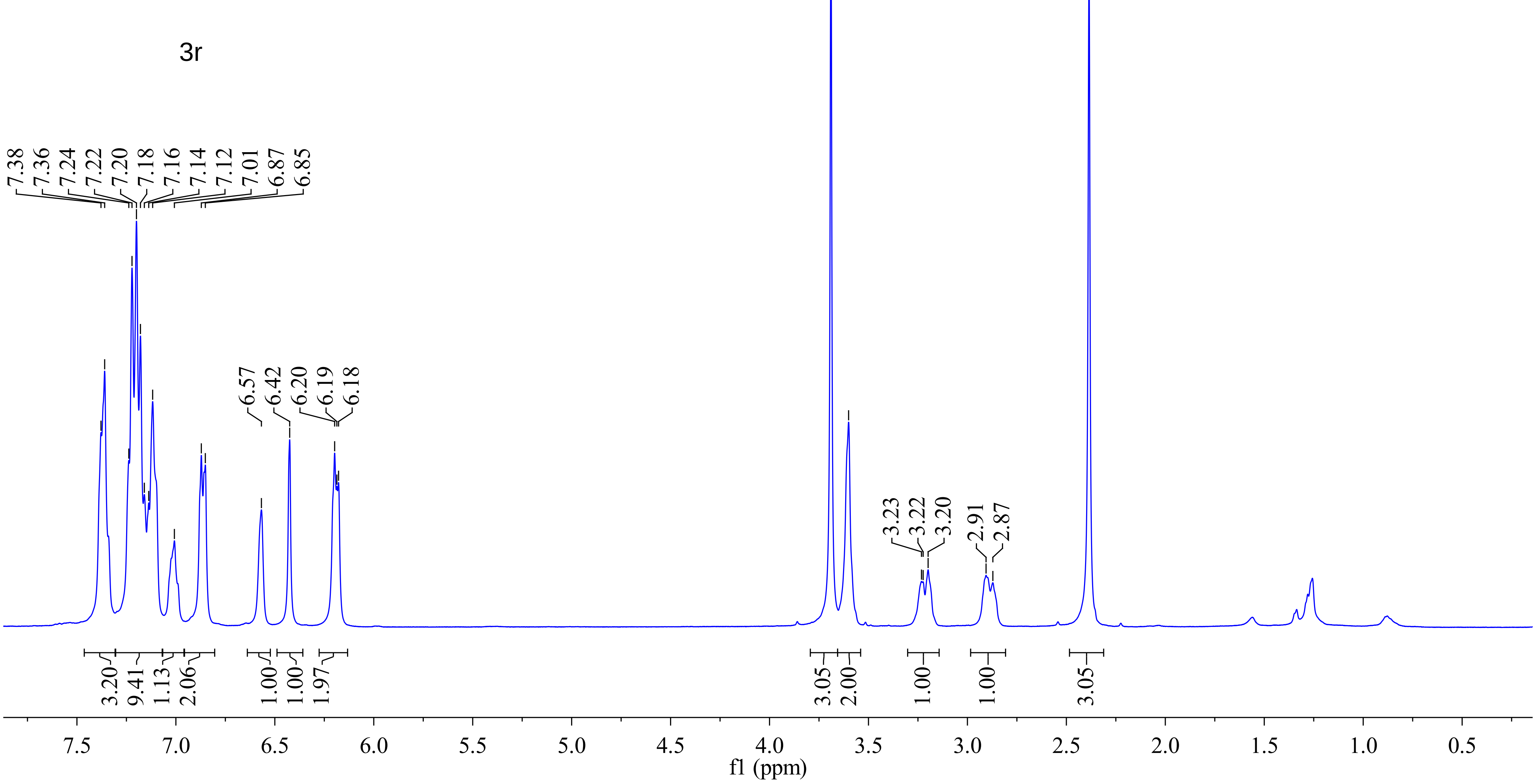

7.38
7.36
7.24
7.22
7.20
7.18
7.16
7.14
7.12
7.01
6.87
6.85
6.57
6.42
6.20
6.19
6.18
3.69
3.60
3.23
3.22
3.20
2.91
2.87
2.39
3.20
9.41
1.13
2.06
1.00
1.00
1.97
3.05
2.00
1.00
1.00
3.05
7.5
7.0
6.5
6.0
5.5
5.0
4.5
4.0
3.5
3.0
2.5
2.0
1.5
1.0
0.5
f1 (ppm)

wyd-6-137 C

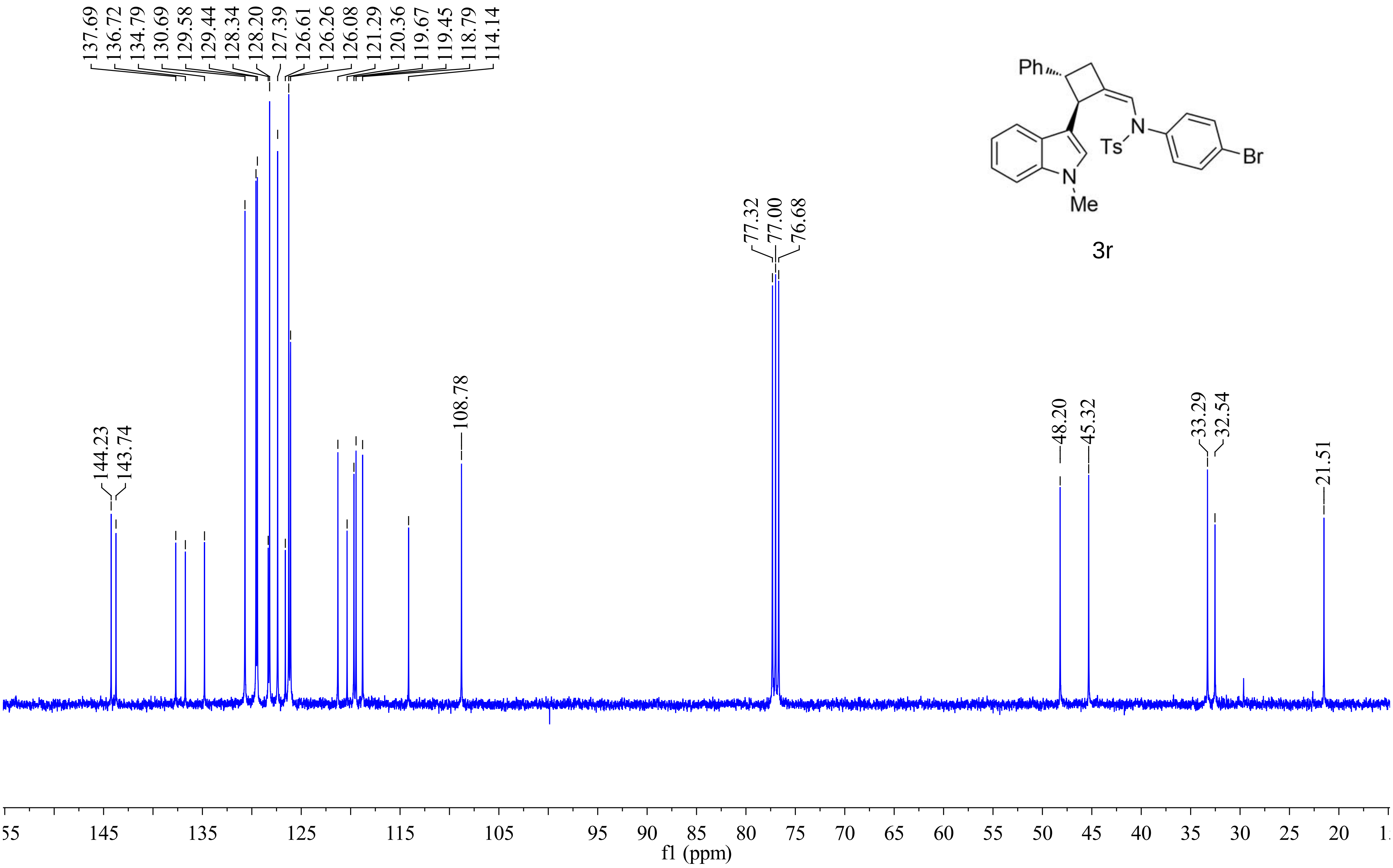

137.69
136.72
134.79
130.69
129.58
129.44
128.34
128.20
127.39
126.61
126.26
126.08
121.29
120.36
119.67
119.45
118.79
114.14
144.23
143.74
108.78
77.32
77.00
76.68
48.20
45.32
33.29
32.54
21.51
Ph
Ts
N
Br
N
Me
3r
55 145 135 125 115 105 95 90 85 80 75 70 65 60 55 50 45 40 35 30 25 20
fl (ppm)

wyd-7-5 H

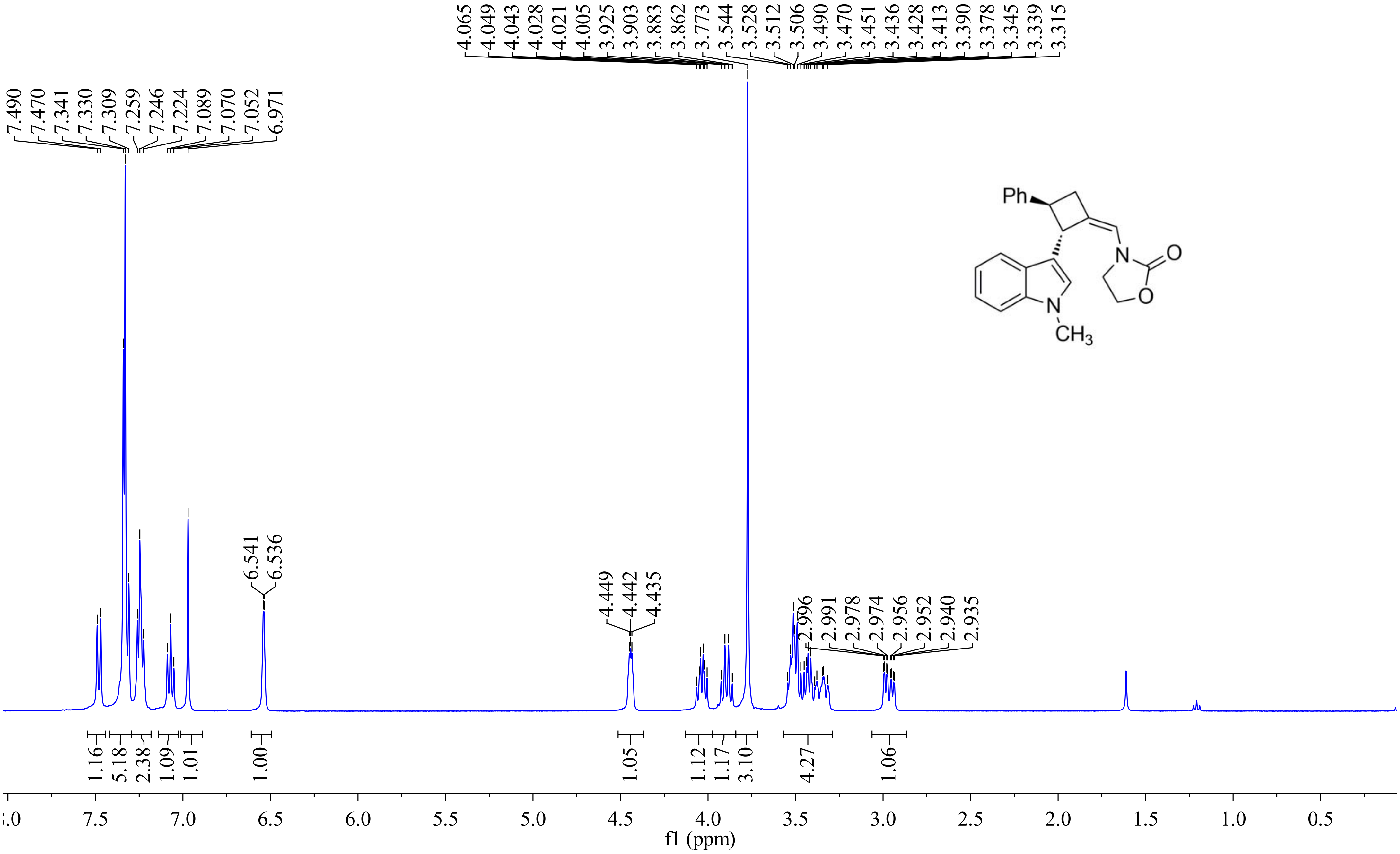
7.490
7.470
7.341
7.330
7.309
7.259
7.246
7.224
7.089
7.070
7.052
6.971
6.541
6.536
4.449
4.442
4.435
4.065
4.049
4.043
4.028
4.021
4.005
3.925
3.903
3.883
3.862
3.773
3.544
3.528
3.512
3.506
3.490
3.470
3.451
3.436
3.428
3.413
3.390
3.378
3.345
3.339
3.315
2.996
2.991
2.978
2.974
2.956
2.952
2.940
2.935
1.16
5.18
2.38
1.09
1.01
1.00
1.05
1.12
1.17
3.10
4.27
1.06
7.5
7.0
6.5
6.0
5.5
5.0
4.5
4.0
3.5
3.0
2.5
2.0
1.5
1.0
0.5
f1 (ppm)
Ph
N
O
O
N
CH3

wyd-7-5 C

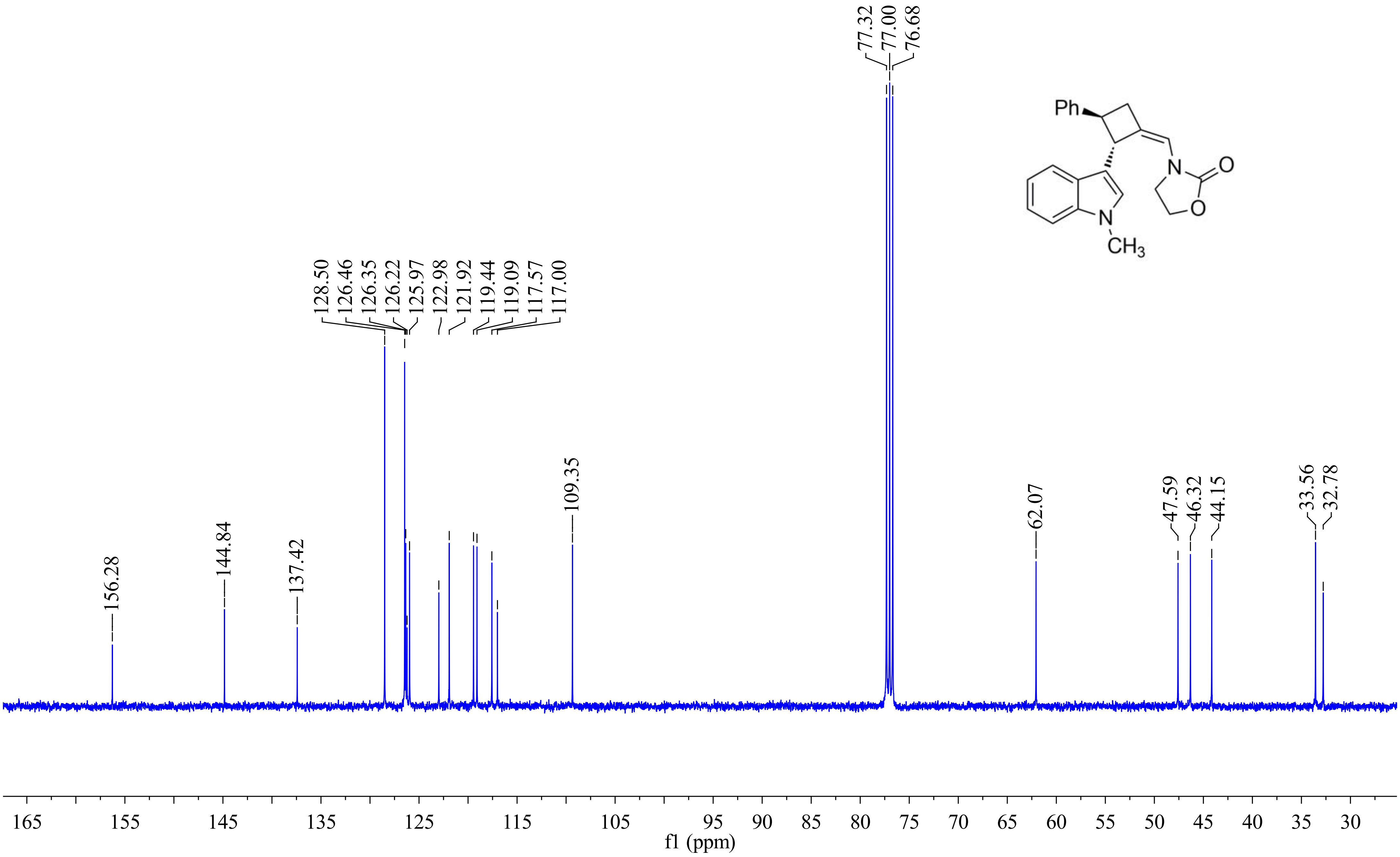

77.32
77.00
76.68
Ph
N
O
O
N
CH3
128.50
126.46
126.35
126.22
125.97
122.98
121.92
119.44
119.09
117.57
117.00
109.35
156.28
144.84
137.42
62.07
47.59
46.32
44.15
33.56
32.78
165
155
145
135
125
115
105
95
90
85
80
75
70
65
60
55
50
45
40
35
30
f1 (ppm)

wyd-7-41 H

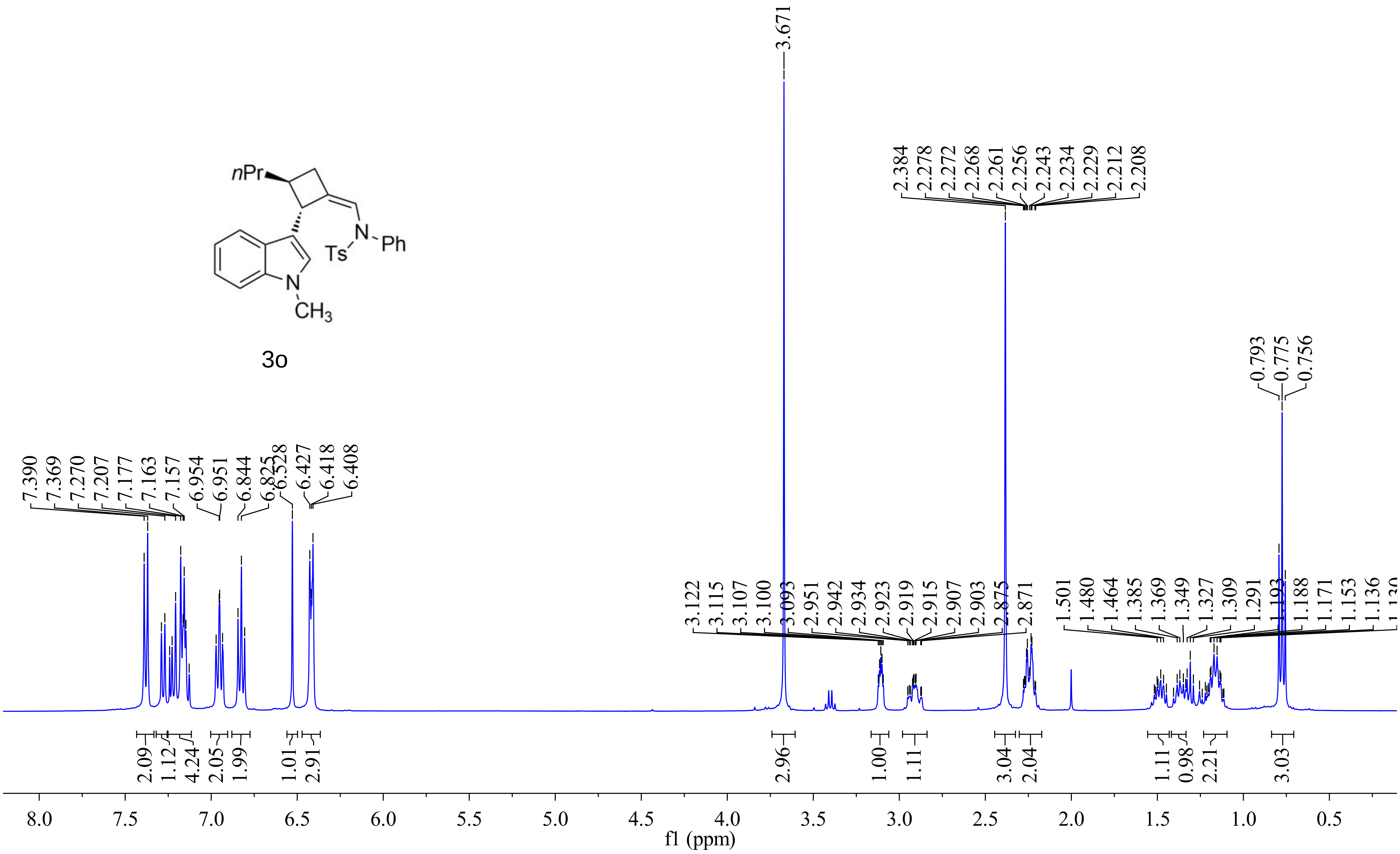
nPr
N
Ph
Ts
N
CH3
3o
7.390 7.369 7.270 7.207 7.177 7.163 7.157 6.954 6.951 6.844 6.825 6.528 6.427 6.418 6.408
3.671
2.384 2.278 2.272 2.268 2.261 2.256 2.243 2.234 2.229 2.212 2.208
3.122 3.115 3.107 3.100 3.093 2.951 2.942 2.934 2.923 2.919 2.915 2.907 2.903 2.875 2.871
1.501 1.480 1.464 1.385 1.369 1.349 1.327 1.309 1.291 1.188 1.171 1.153 1.136
0.793 0.775 0.756
2.09 1.12 4.24 2.05 1.99 1.01 2.91 2.96 1.00 1.11 3.04 2.04 1.11 0.98 2.21 3.03
8.0 7.5 7.0 6.5 6.0 5.5 5.0 4.5 4.0 3.5 3.0 2.5 2.0 1.5 1.0 0.5
f1 (ppm)

wyd-7-41 C

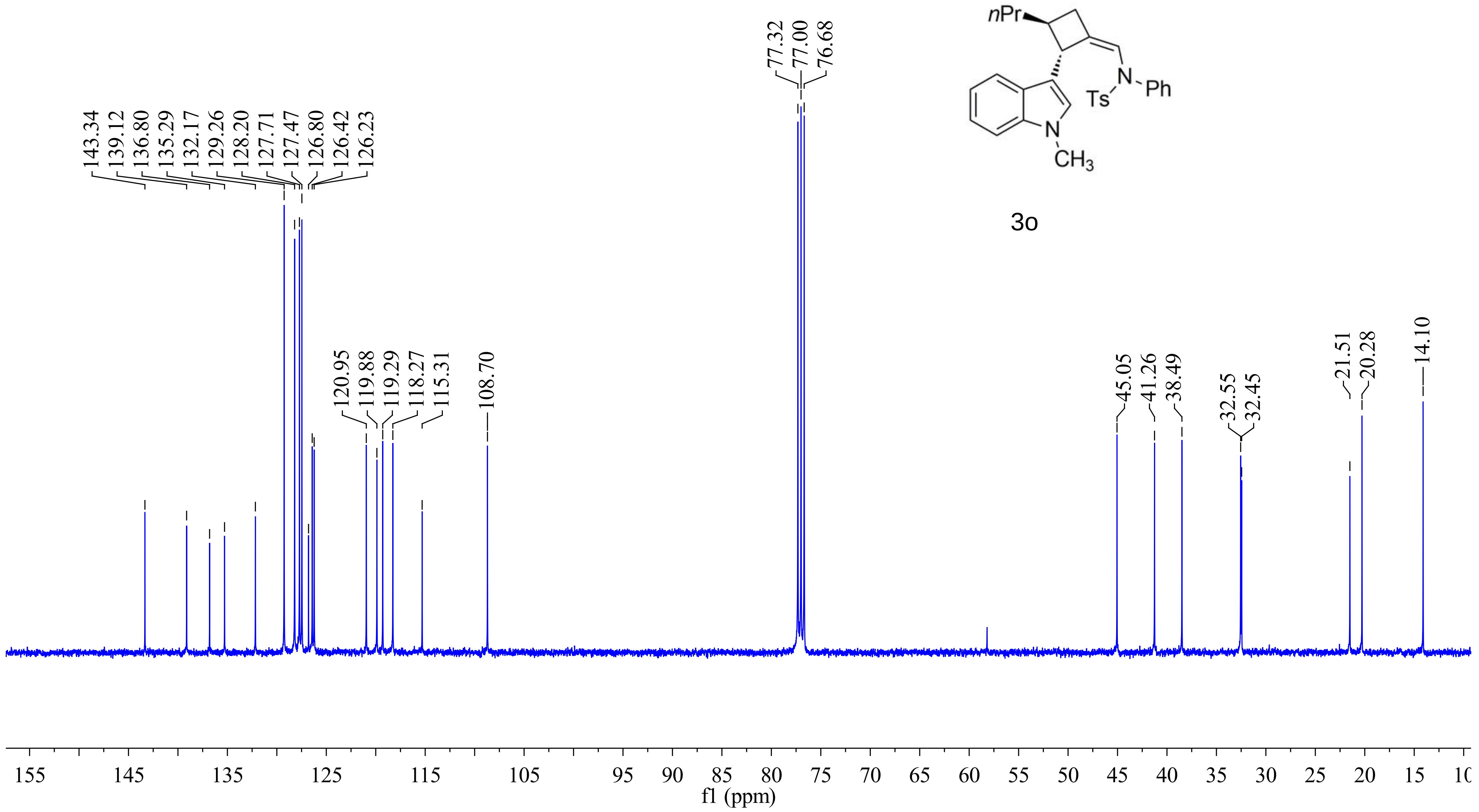

143.34
139.12
136.80
135.29
132.17
129.26
128.20
127.71
127.47
126.80
126.42
126.23
120.95
119.88
119.29
118.27
115.31
108.70
77.32
77.00
76.68
45.05
41.26
38.49
32.55
32.45
21.51
20.28
14.10
nPr
Ts
N
Ph
CH3
3o
155
145
135
125
115
105
95
90
85
80
75
70
65
60
55
50
45
40
35
30
25
20
15
10
f1 (ppm)

wyd-6-93-1 H

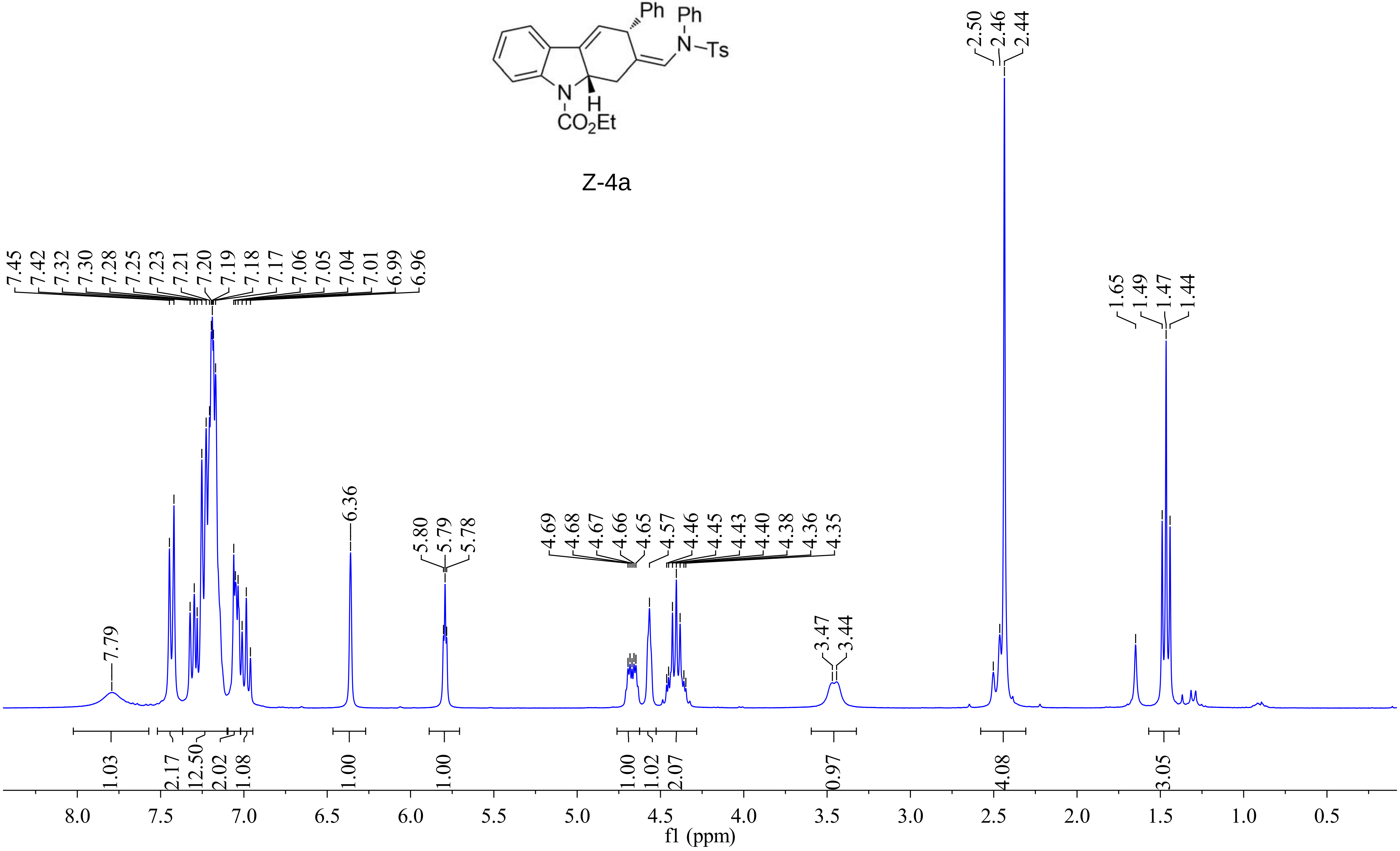

Ph
Ph
N–Ts
N
H
CO2Et
Z-4a
7.45 7.42 7.32 7.30 7.28 7.25 7.23 7.21 7.20 7.19 7.18 7.17 7.06 7.05 7.04 7.01 6.99 6.96
7.79
6.36
5.80 5.79 5.78
4.69 4.68 4.67 4.66 4.65 4.57 4.46 4.45 4.43 4.40 4.38 4.36 4.35
3.47 3.44
2.50 2.46 2.44
1.65 1.49 1.47 1.44
1.03 2.17 12.50 2.02 1.08 1.00 1.00 1.00 1.02 2.07 0.97 4.08 3.05
8.0 7.5 7.0 6.5 6.0 5.5 5.0 4.5 4.0 3.5 3.0 2.5 2.0 1.5 1.0 0.5
f1 (ppm)

wyd-6-93-1 C

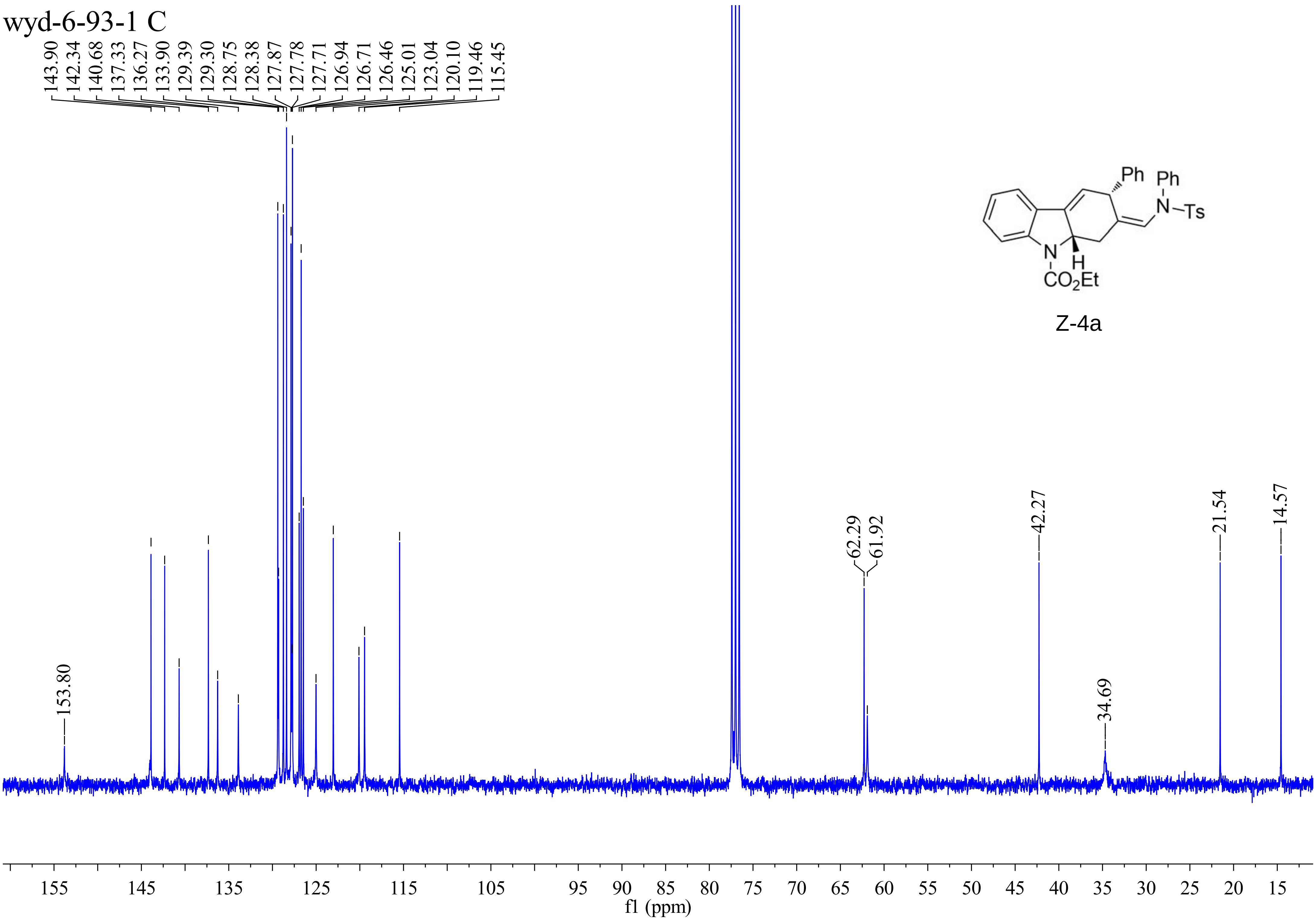
143.90
142.34
140.68
137.33
136.27
133.90
129.39
129.30
128.75
128.38
127.87
127.78
127.71
126.94
126.71
126.46
125.01
123.04
120.10
119.46
115.45
153.80
62.29
61.92
42.27
34.69
21.54
14.57
Ph
Ph
N
Ts
N
H
CO2Et
Z-4a
155
145
135
125
115
105
95
90
85
80
75
70
65
60
55
50
45
40
35
30
25
20
15
f1 (ppm)

wyd-6-141-2 H

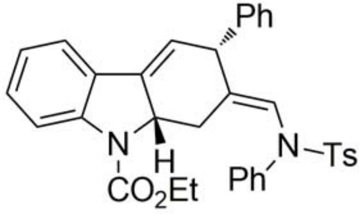

E-4a

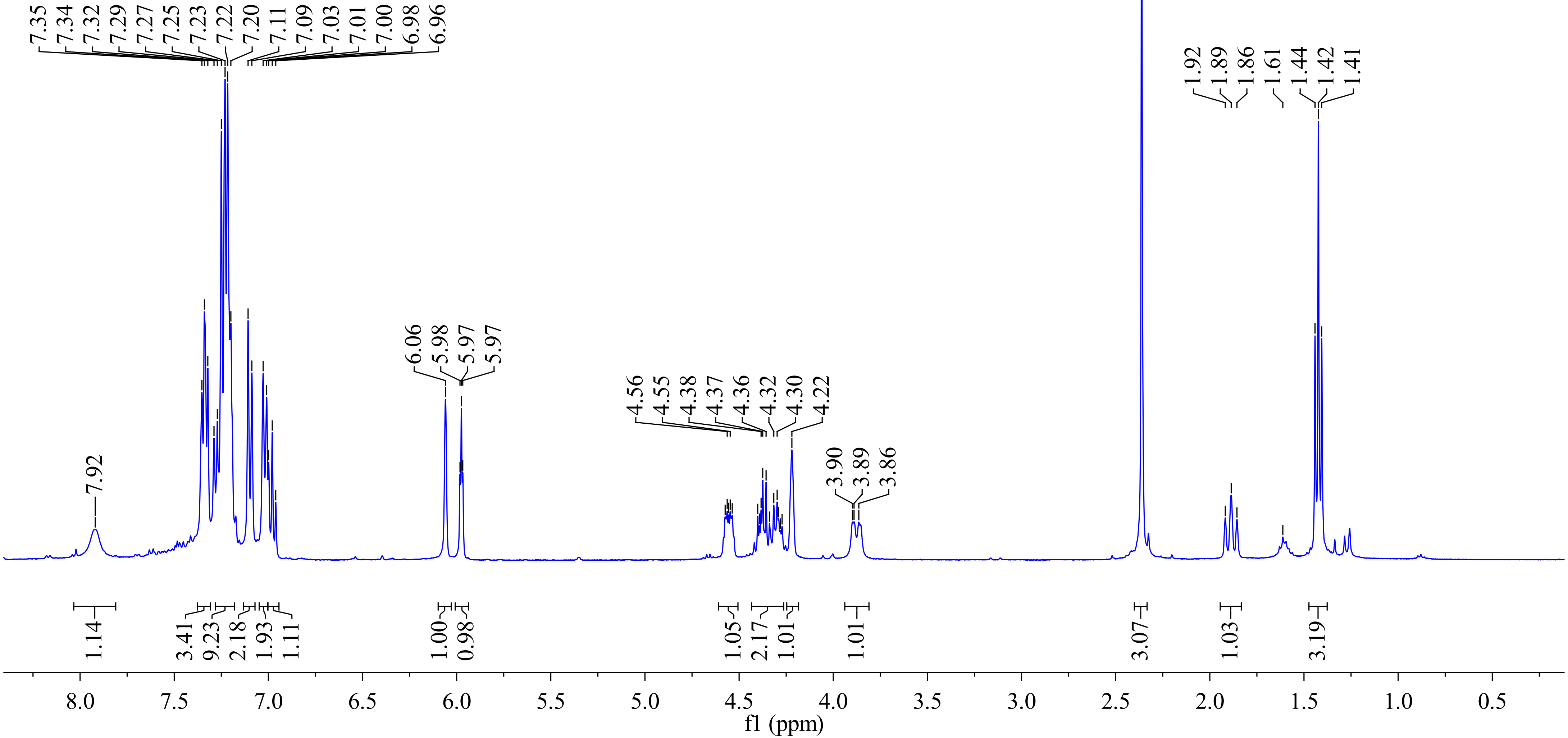

wyd-6-141-2 C

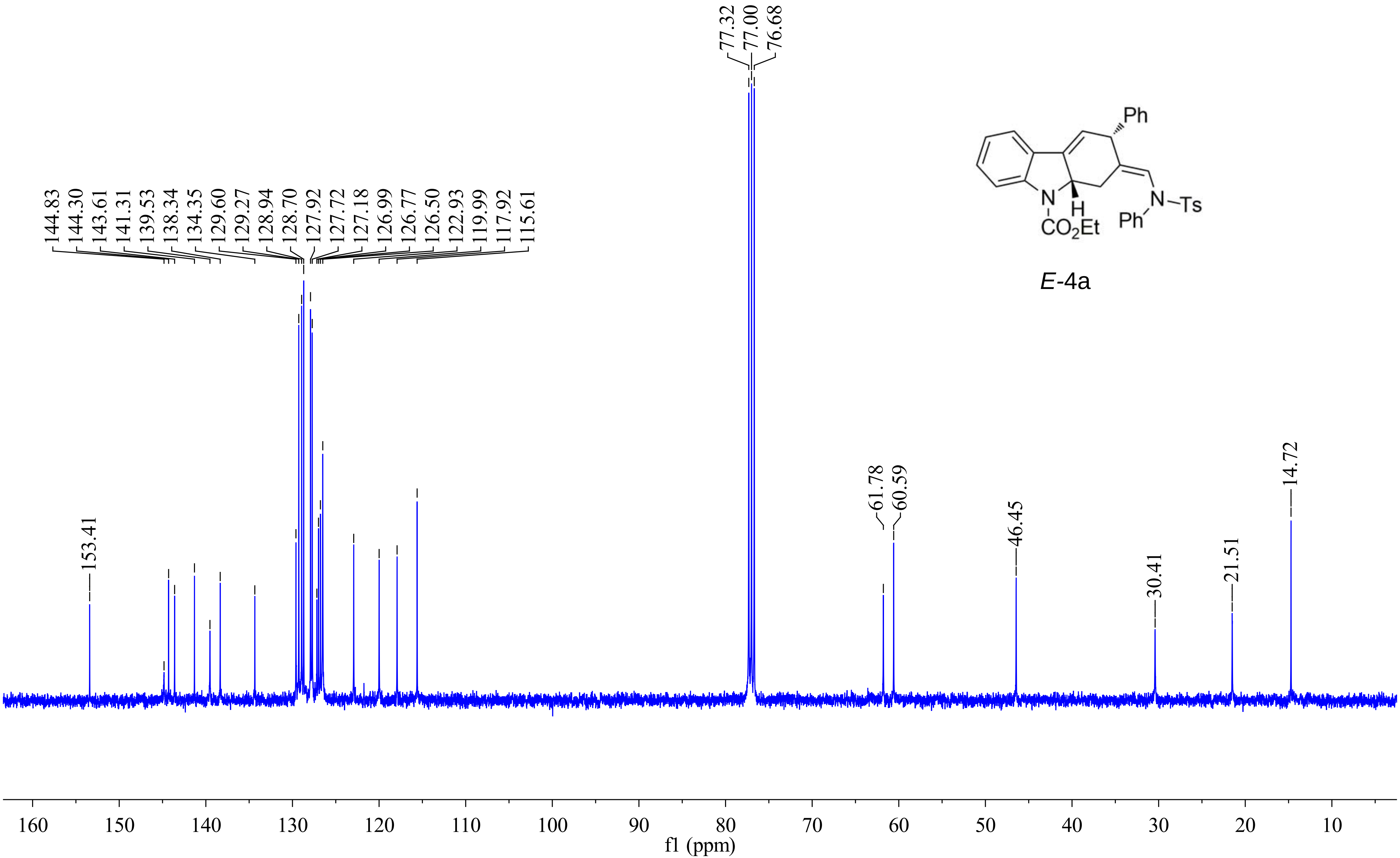
153.41
144.83
144.30
143.61
141.31
139.53
138.34
134.35
129.60
129.27
128.94
128.70
127.92
127.72
127.18
126.99
126.77
126.50
122.93
119.99
117.92
115.61
77.32
77.00
76.68
61.78
60.59
46.45
30.41
21.51
14.72
160 150 140 130 120 110 100 90 80 70 60 50 40 30 20 10
f1 (ppm)
Ph
N
H
CO2Et
Ph
N
Ts
E-4a

wyd-6-120-1 H

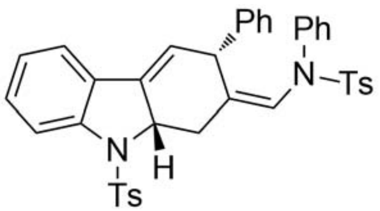

4b

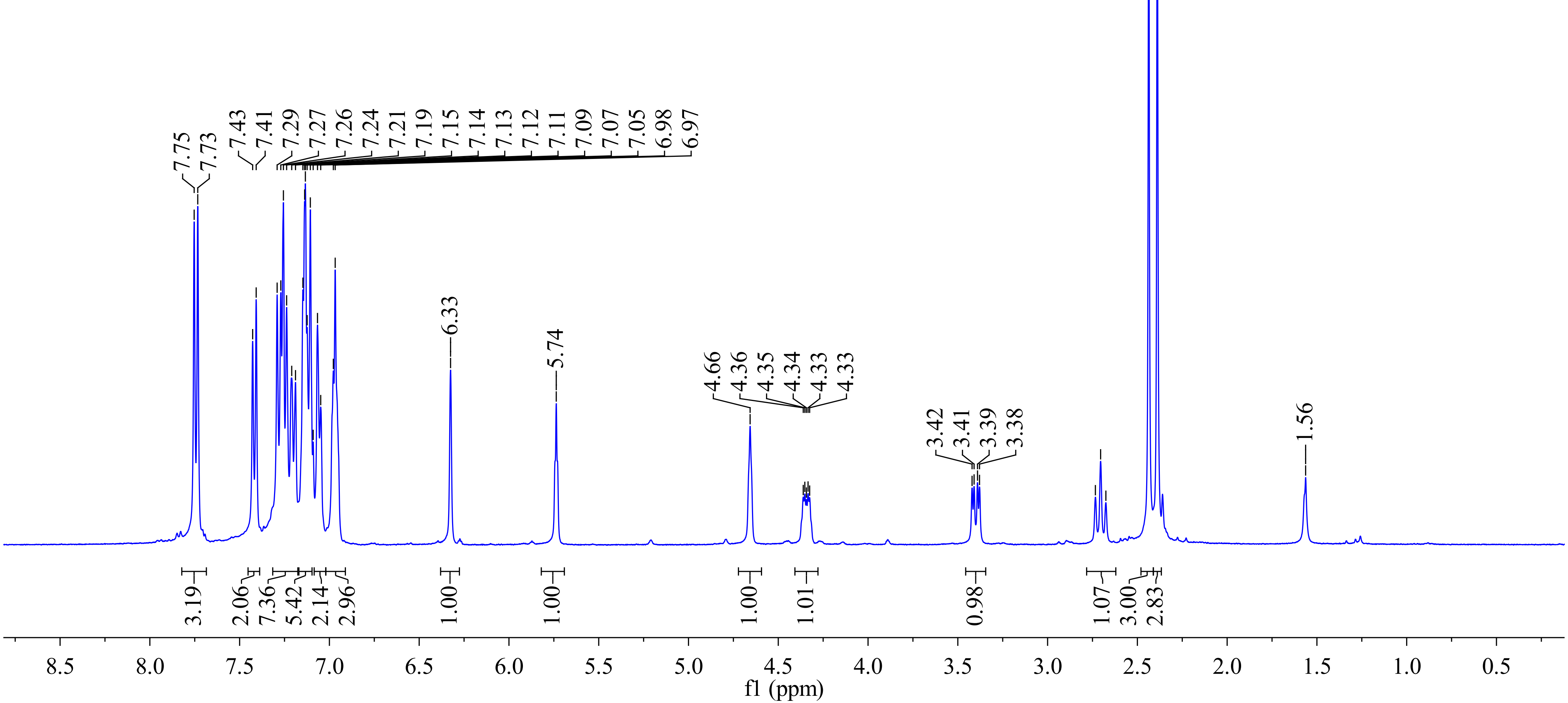

wyd-6-120-1 C

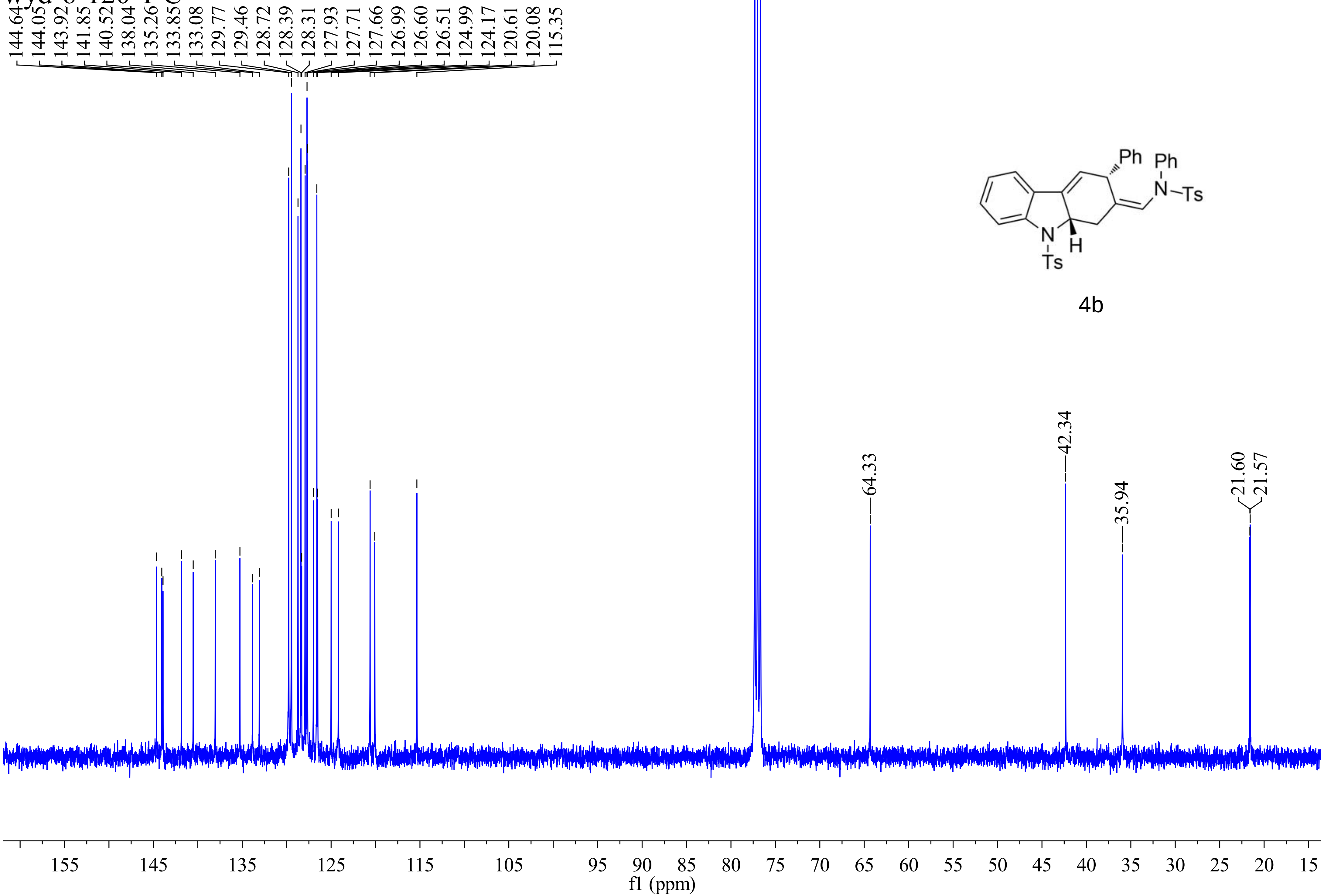

4b

wyd-7-15-2 H

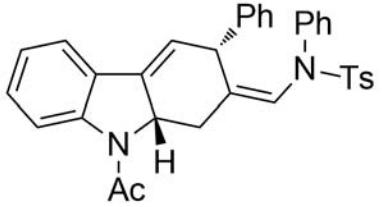

4c

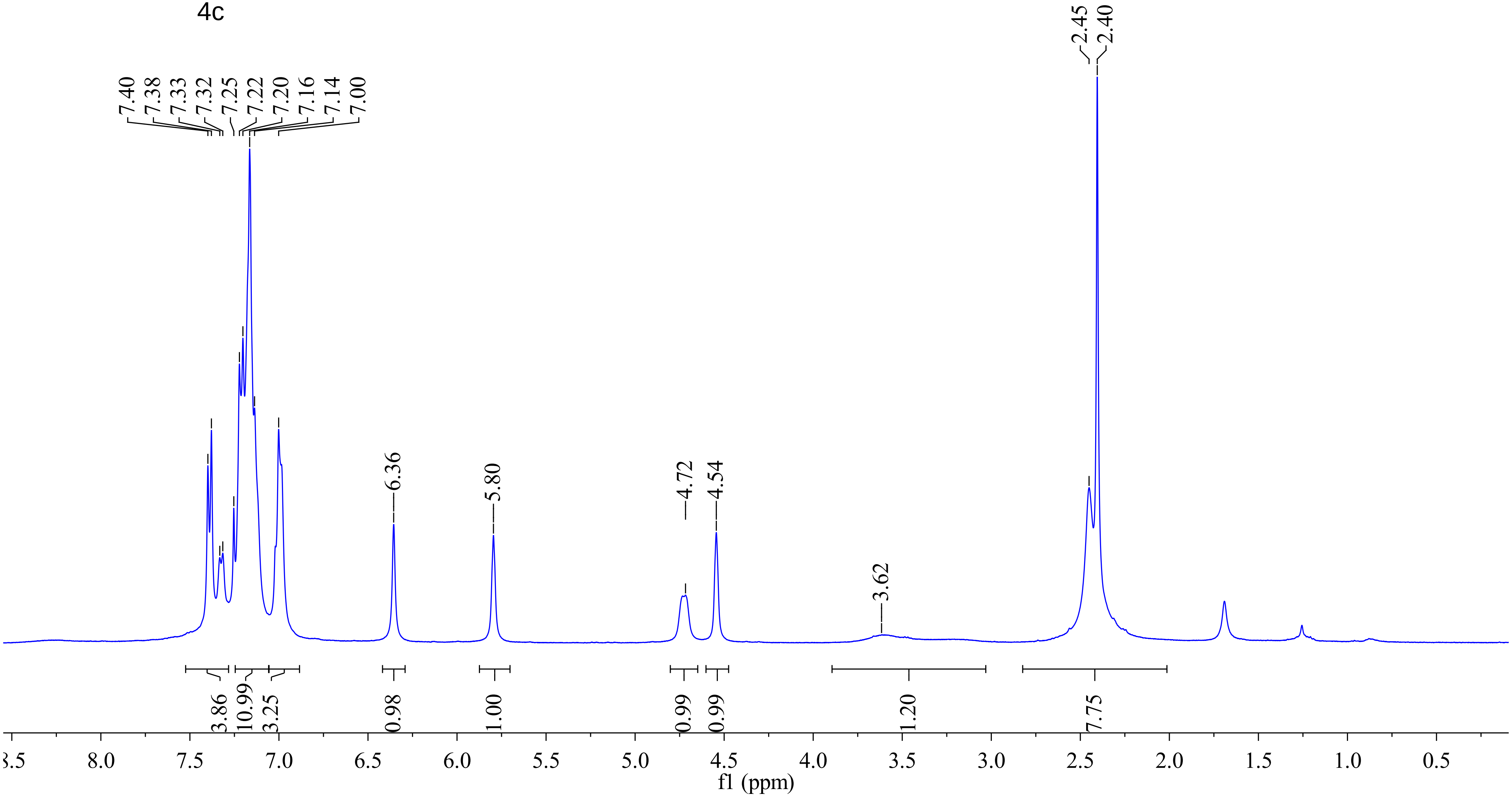

7.40
7.38
7.33
7.32
7.25
7.22
7.20
7.16
7.14
7.00
6.36
5.80
4.72
4.54
3.62
2.45
2.40
3.86
10.99
3.25
0.98
1.00
0.99
0.99
1.20
7.75
8.5
8.0
7.5
7.0
6.5
6.0
5.5
5.0
4.5
4.0
3.5
3.0
2.5
2.0
1.5
1.0
0.5
f1 (ppm)

WYD-7-15-2C

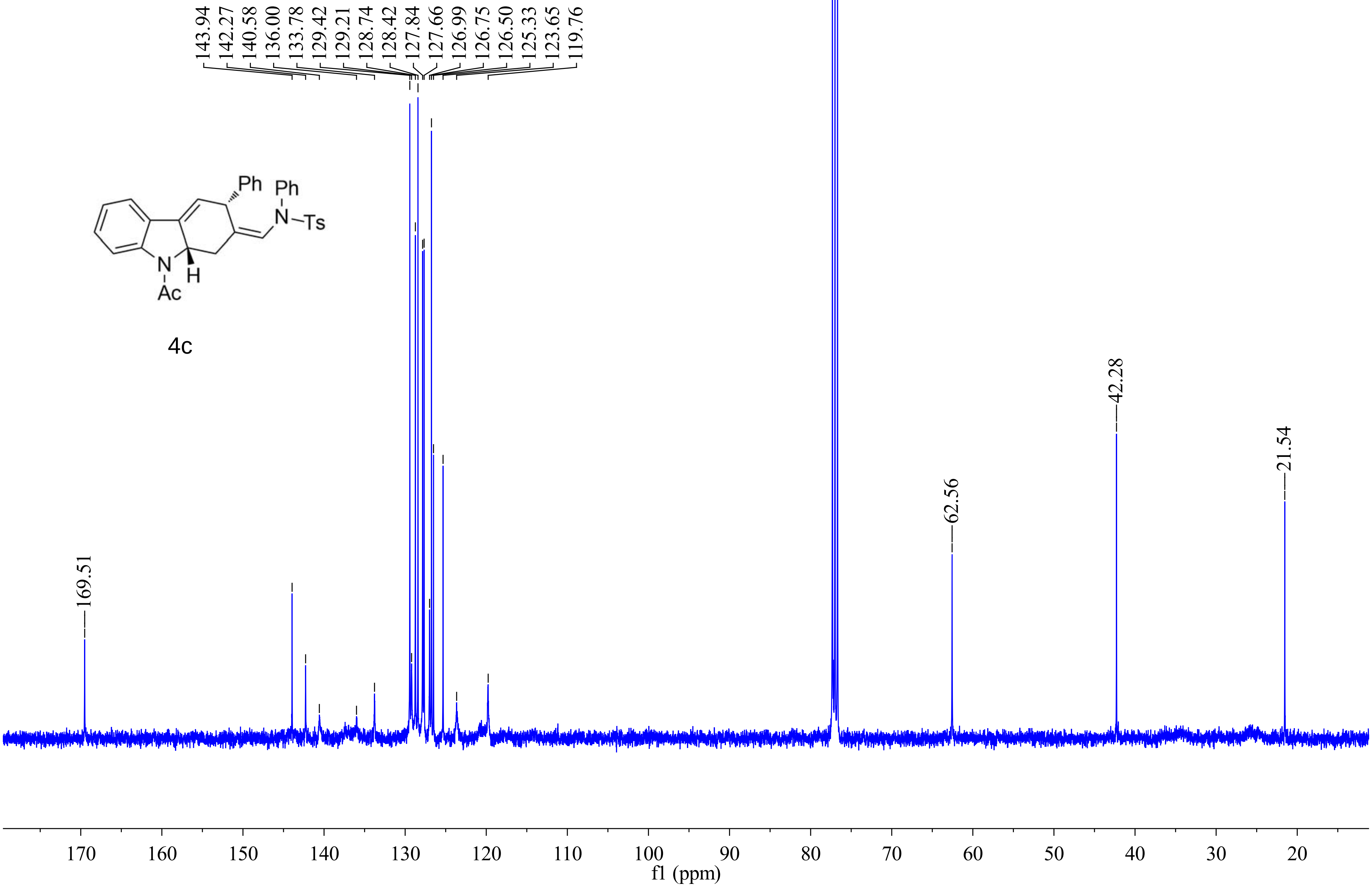
143.94
142.27
140.58
136.00
133.78
129.42
129.21
128.74
128.42
127.84
127.66
126.99
126.75
126.50
125.33
123.65
119.76
169.51
62.56
42.28
21.54
170
160
150
140
130
120
110
100
90
80
70
60
50
40
30
20
f1 (ppm)
Ph
Ph
N
Ts
N
H
Ac
4c

wyd-6-83-1 H

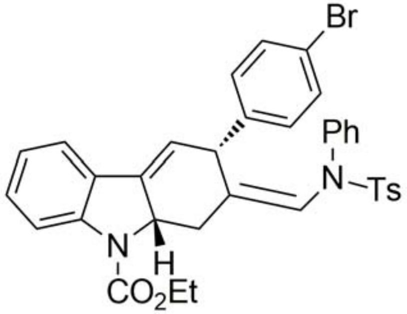

4d

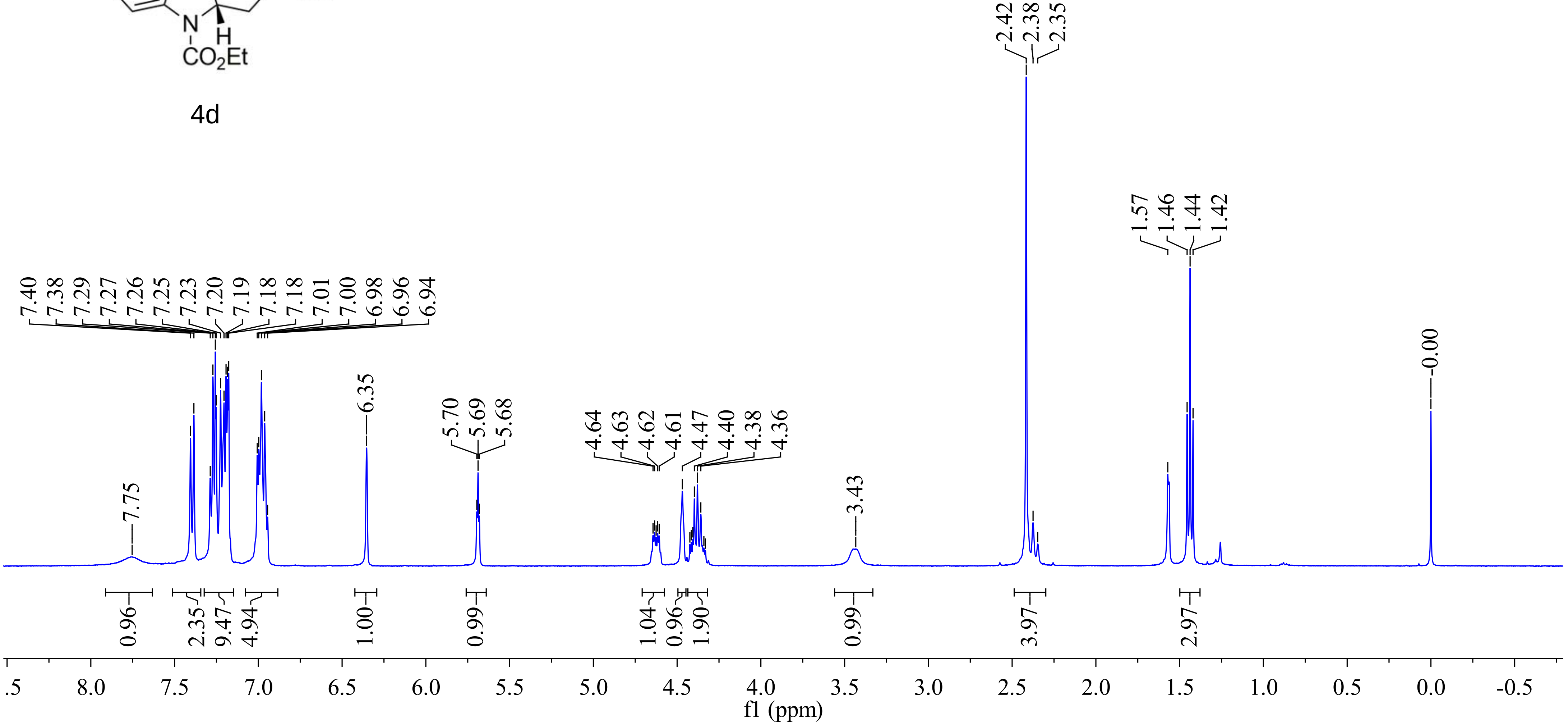
7.40
7.38
7.29
7.27
7.26
7.25
7.23
7.20
7.19
7.18
7.18
7.01
7.00
6.98
6.96
6.94
7.75
6.35
5.70
5.69
5.68
4.64
4.63
4.62
4.61
4.47
4.40
4.38
4.36
3.43
2.42
2.38
2.35
1.57
1.46
1.44
1.42
-0.00
0.96
2.35
9.47
4.94
1.00
0.99
1.04
0.96
1.90
0.99
3.97
2.97
f1 (ppm)

wyd-6-83-1 C

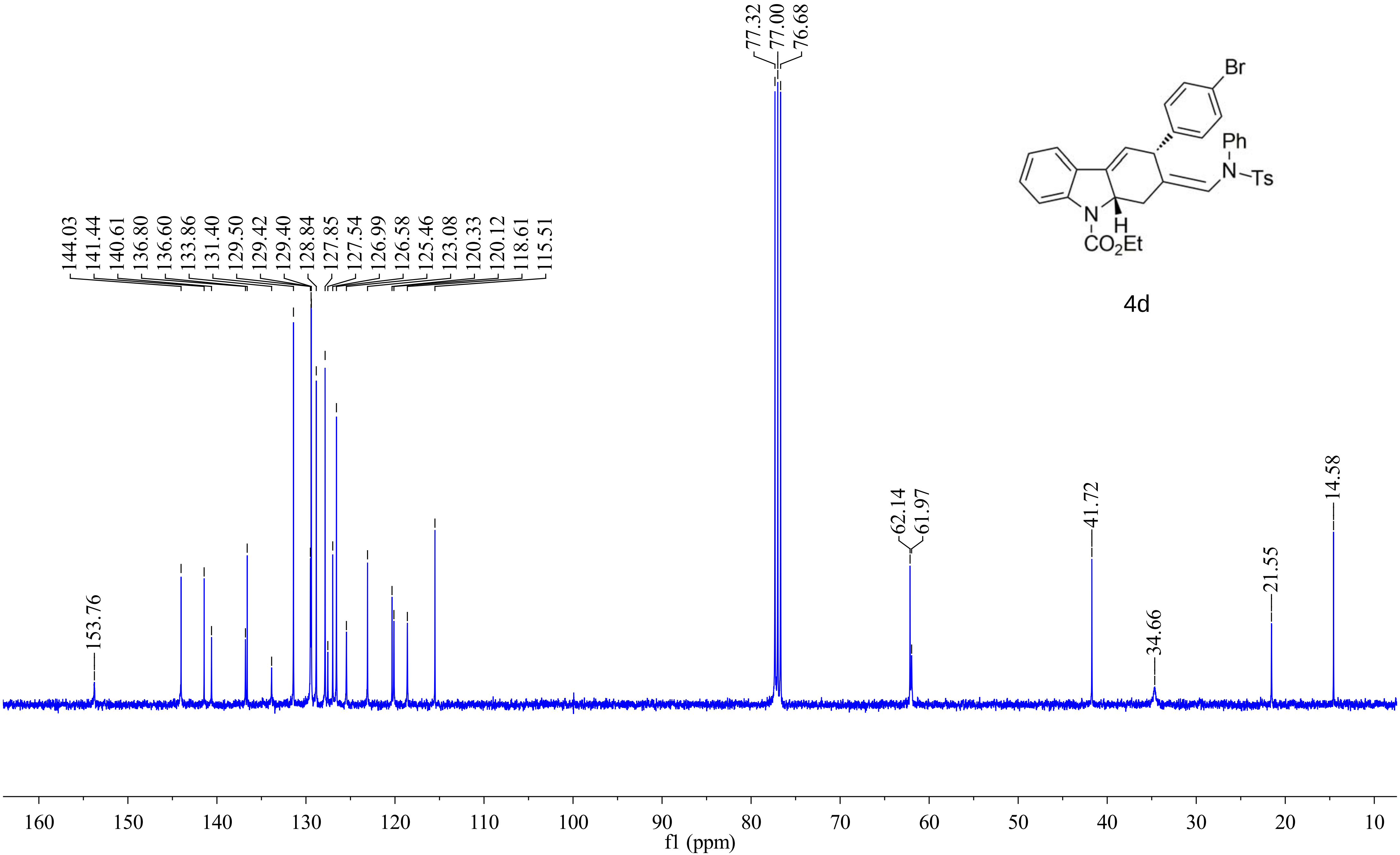
144.03
141.44
140.61
136.80
136.60
133.86
131.40
129.50
129.42
129.40
128.84
127.85
127.54
126.99
126.58
125.46
123.08
120.33
120.12
118.61
115.51
77.32
77.00
76.68
153.76
62.14
61.97
41.72
34.66
21.55
14.58
160
150
140
130
120
110
100
90
80
70
60
50
40
30
20
10
f1 (ppm)
Br
Ph
N
Ts
N
H
CO_2Et

4d

wyd-6-84-1 H

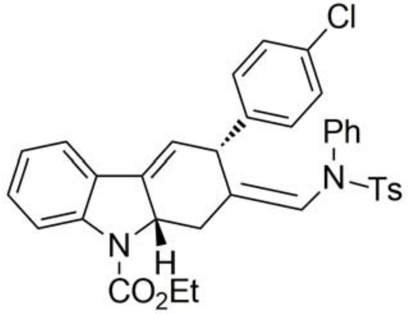

Cl
Ph
N
Ts
N
H
CO2Et

4e

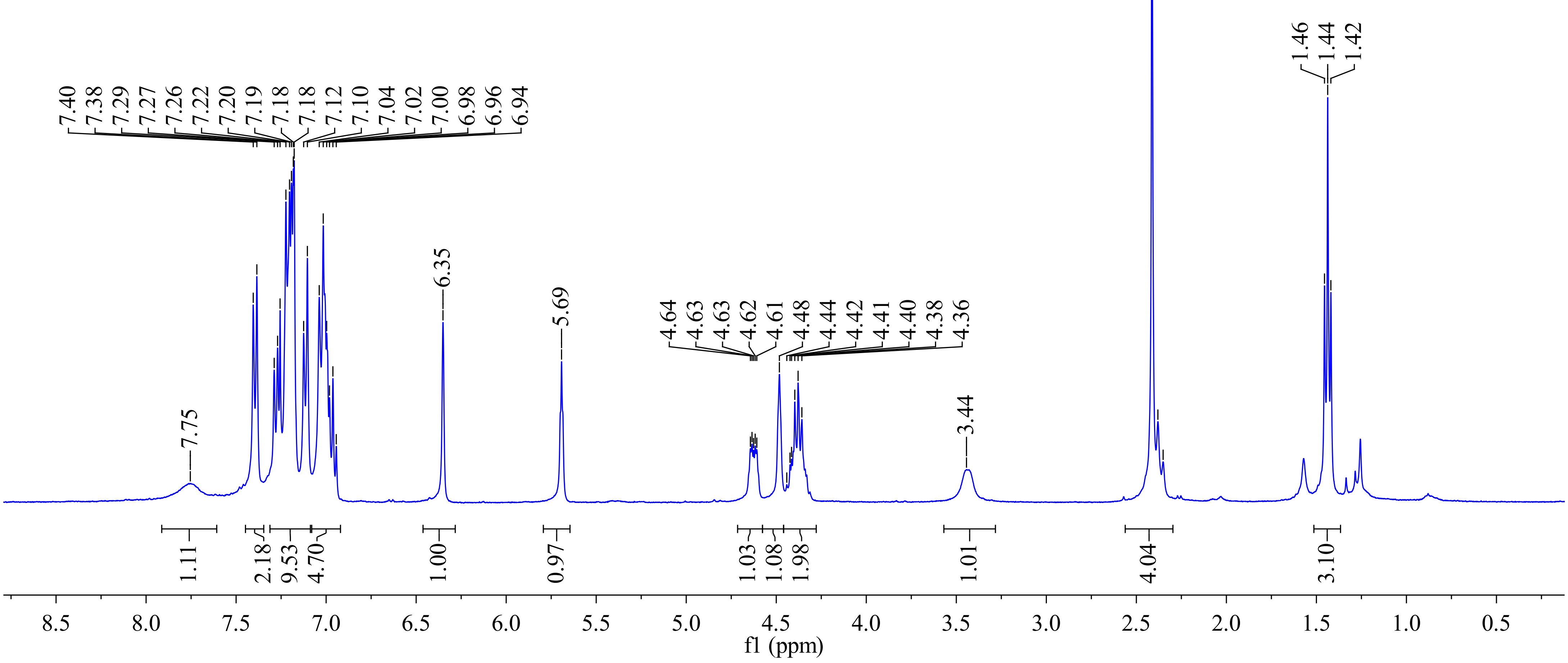

7.40 7.38 7.29 7.27 7.26 7.22 7.20 7.19 7.18 7.18 7.12 7.10 7.04 7.02 7.00 6.98 6.96 6.94
7.75
6.35
5.69
4.64 4.63 4.63 4.62 4.61 4.48 4.44 4.42 4.41 4.40 4.38 4.36
3.44
2.41 2.38 2.35
1.46 1.44 1.42
1.11 2.18 9.53 4.70 1.00 0.97 1.03 1.08 1.98 1.01 4.04 3.10
8.5 8.0 7.5 7.0 6.5 6.0 5.5 5.0 4.5 4.0 3.5 3.0 2.5 2.0 1.5 1.0 0.5
f1 (ppm)

wyd-6-84-1 C

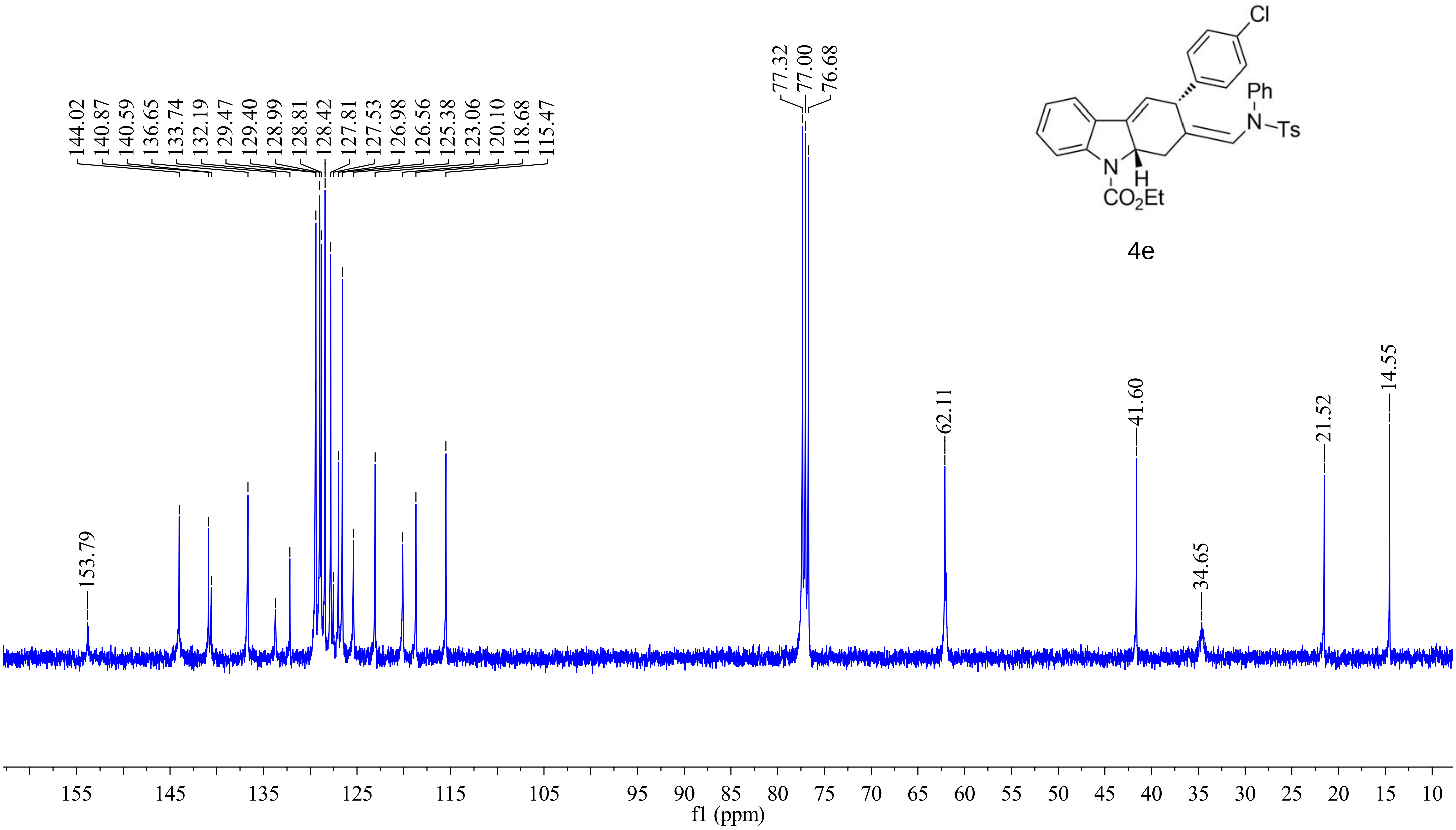
144.02
140.87
140.59
136.65
133.74
132.19
129.47
129.40
128.99
128.81
128.42
127.81
127.53
126.98
126.56
125.38
123.06
120.10
118.68
115.47
77.32
77.00
76.68
153.79
62.11
41.60
34.65
21.52
14.55
Cl
Ph
N
Ts
N
H
CO2Et
4e
155 145 135 125 115 105 95 90 85 80 75 70 65 60 55 50 45 40 35 30 25 20 15 10
f1 (ppm)

wyd-6-86-1 H

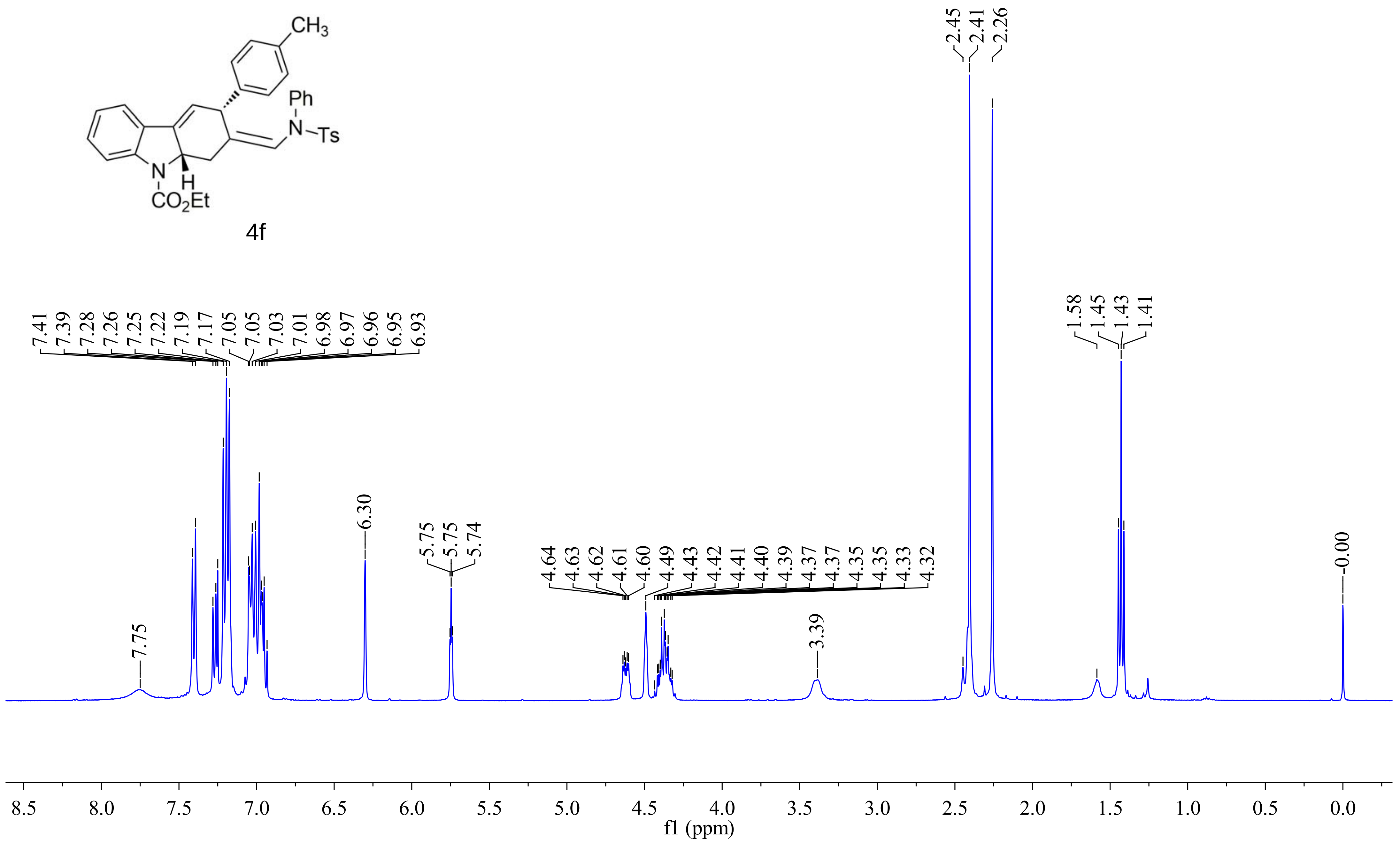

CH3
Ph
N
Ts
N
H
CO2Et
4f
7.41 7.39 7.28 7.26 7.25 7.22 7.19 7.17 7.05 7.05 7.03 7.01 6.98 6.97 6.96 6.95 6.93
7.75
6.30
5.75 5.75 5.74
4.64 4.63 4.62 4.61 4.60 4.49 4.43 4.42 4.41 4.40 4.39 4.37 4.37 4.35 4.35 4.33 4.32
3.39
2.45 2.41 2.26
1.58 1.45 1.43 1.41
-0.00
8.5 8.0 7.5 7.0 6.5 6.0 5.5 5.0 4.5 4.0 3.5 3.0 2.5 2.0 1.5 1.0 0.5 0.0
f1 (ppm)

wyd-6-86-1 C

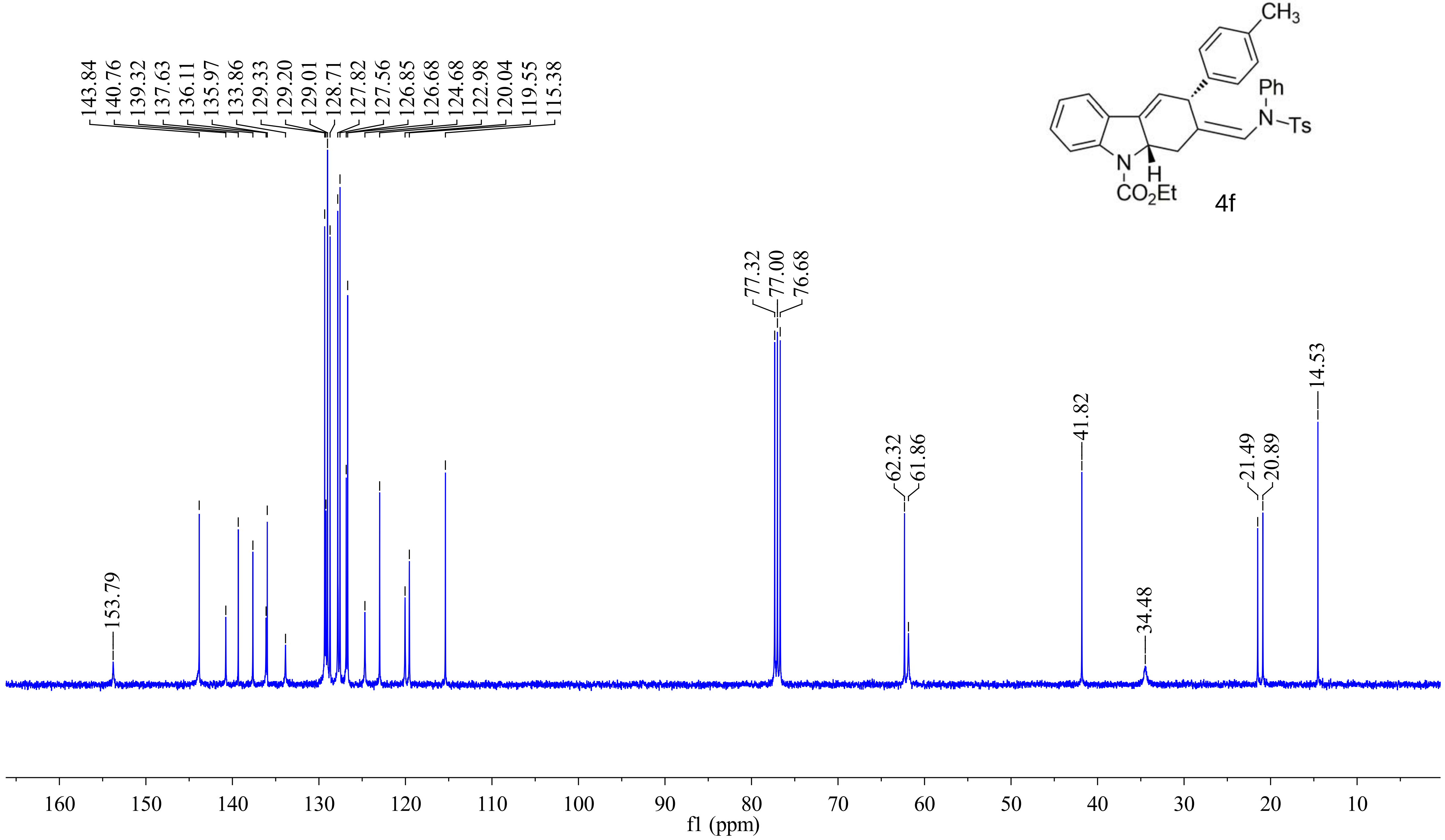

143.84
140.76
139.32
137.63
136.11
135.97
133.86
129.33
129.20
129.01
128.71
127.82
127.56
126.85
126.68
124.68
122.98
120.04
119.55
115.38
77.32
77.00
76.68
153.79
62.32
61.86
41.82
34.48
21.49
20.89
14.53
CH3
Ph
N
Ts
N
H
CO2Et
4f
160
150
140
130
120
110
100
90
80
70
60
50
40
30
20
10
f1 (ppm)

wyd-6-85-1 H

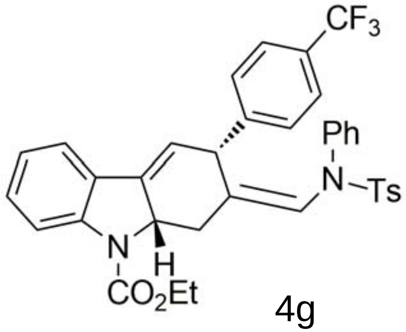

4g

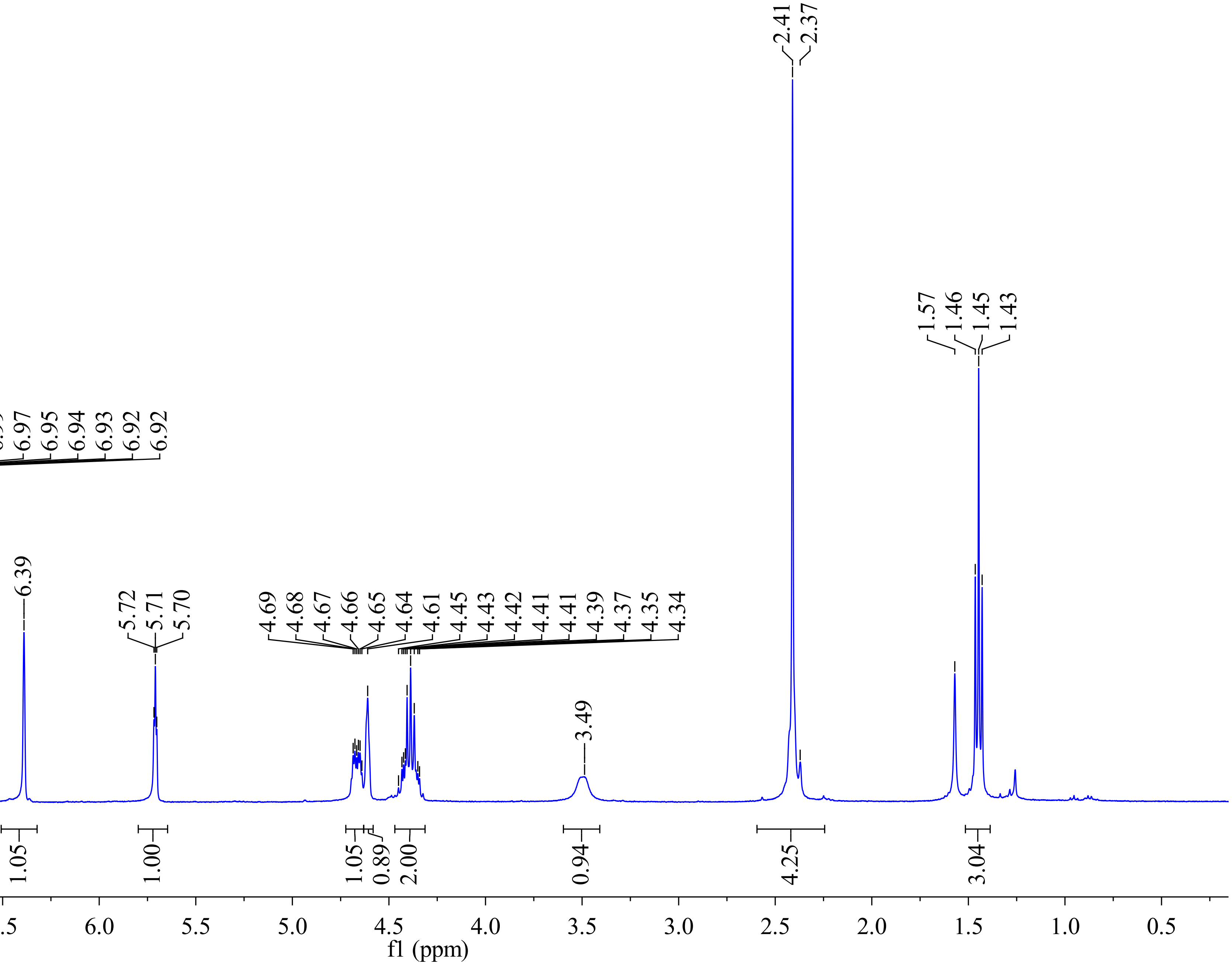

7.41
7.39
7.37
7.29
7.28
7.26
7.24
7.22
7.20
7.18
7.14
7.14
7.13
6.99
6.97
6.95
6.94
6.93
6.92
6.92
7.77
6.39
5.72
5.71
5.70
4.69
4.68
4.67
4.66
4.65
4.64
4.61
4.45
4.43
4.42
4.41
4.41
4.39
4.37
4.35
4.34
3.49
2.41
2.37
1.57
1.46
1.45
1.43
1.14
4.25
1.33
4.93
2.71
0.88
1.94
1.05
1.00
1.05
0.89
2.00
0.94
4.25
3.04
8.0
7.5
7.0
6.5
6.0
5.5
5.0
4.5
4.0
3.5
3.0
2.5
2.0
1.5
1.0
0.5
f1 (ppm)

wyd-6-85-1 F

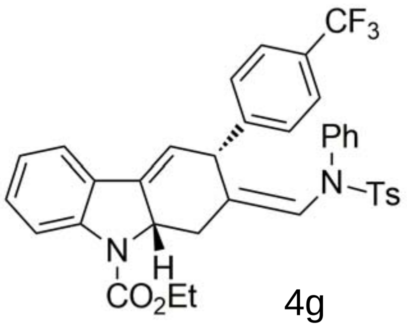

4g

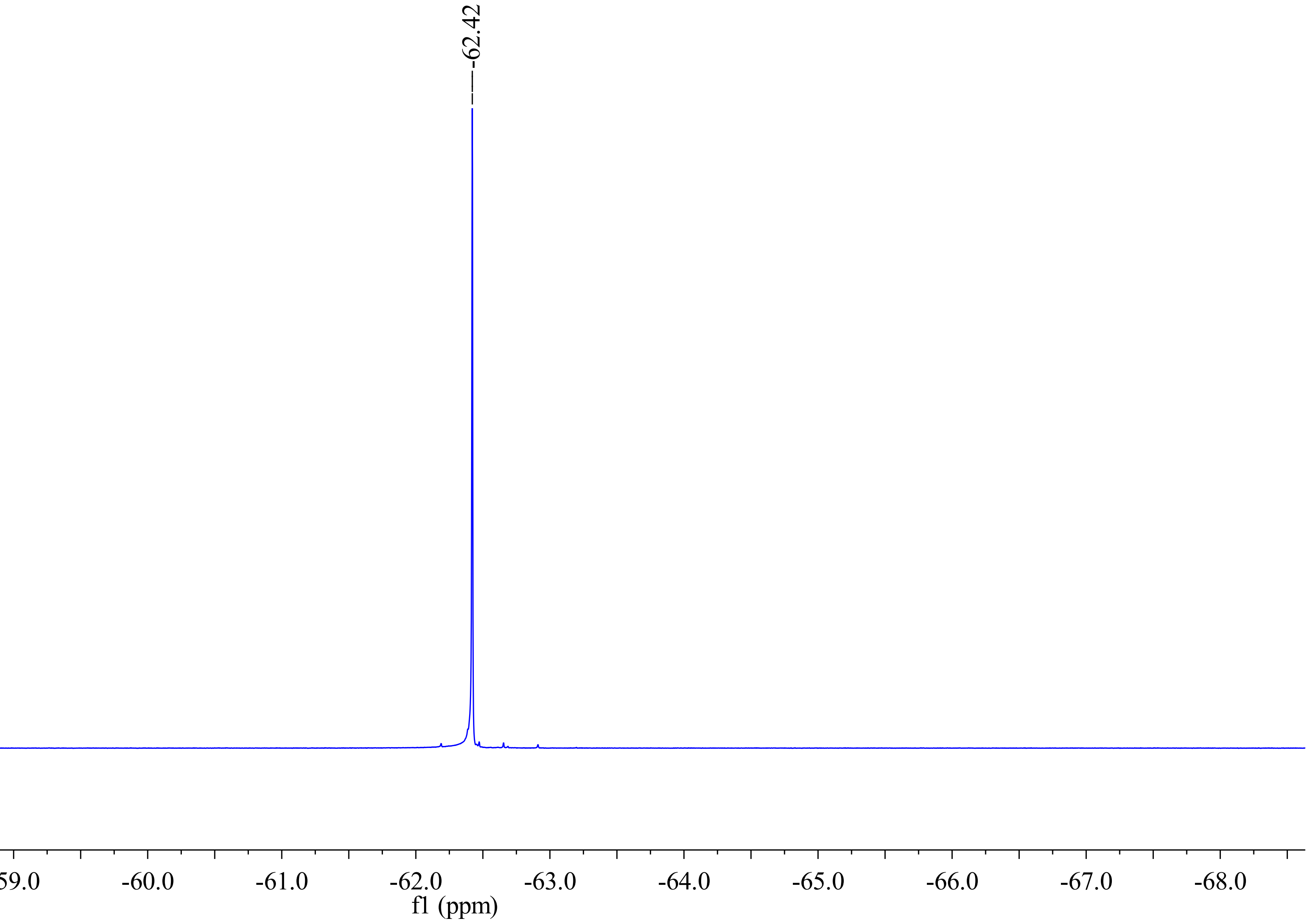

wyd-6-85-1 C

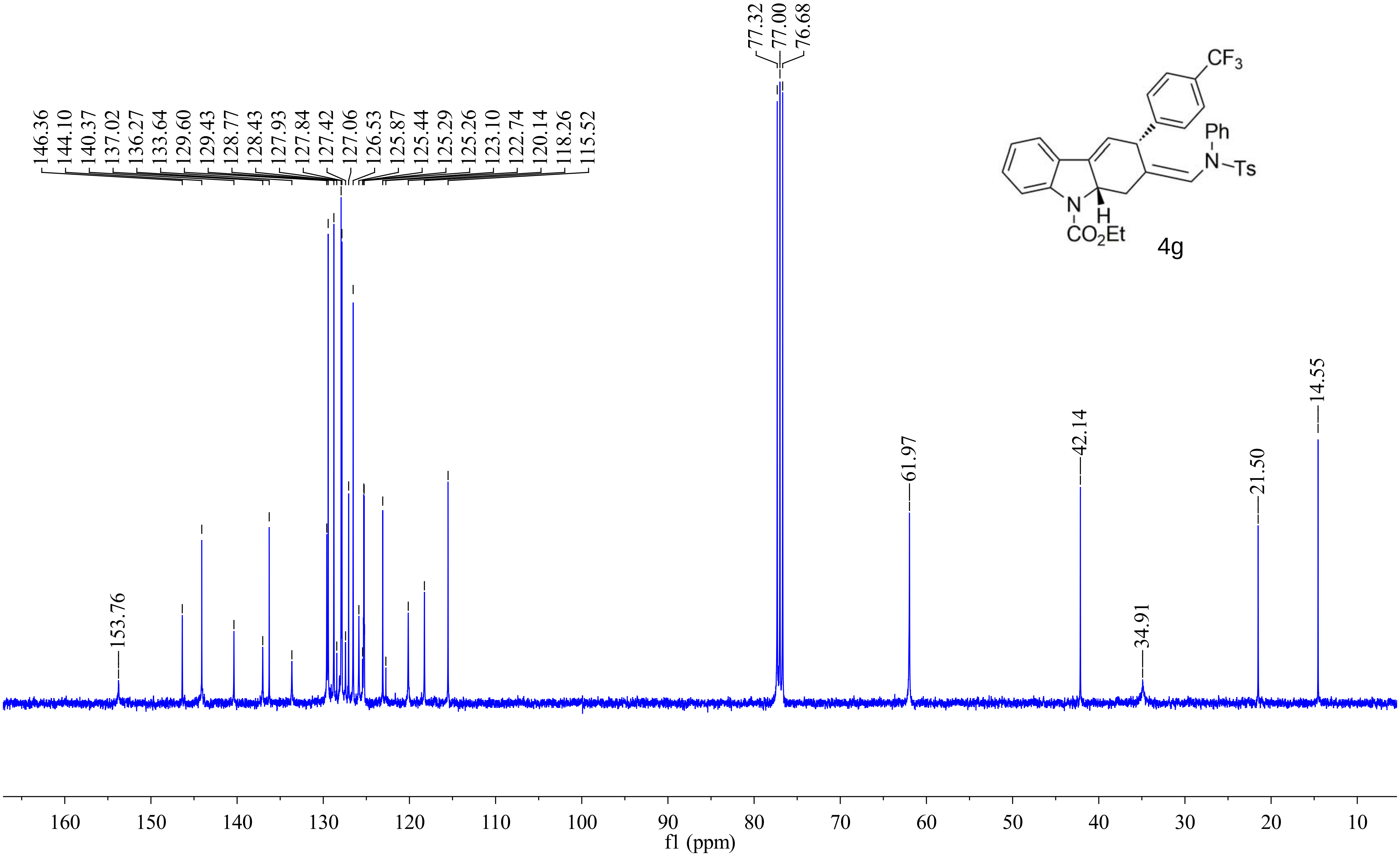

146.36
144.10
140.37
137.02
136.27
133.64
129.60
129.43
128.77
128.43
127.93
127.84
127.42
127.06
126.53
125.87
125.44
125.29
125.26
123.10
122.74
120.14
118.26
115.52
77.32
77.00
76.68
153.76
61.97
42.14
34.91
21.50
14.55
CF3
Ph
N
Ts
N
H
CO2Et
4g
160
150
140
130
120
110
100
90
80
70
60
50
40
30
20
10
f1 (ppm)

wyd-6-148-1 H

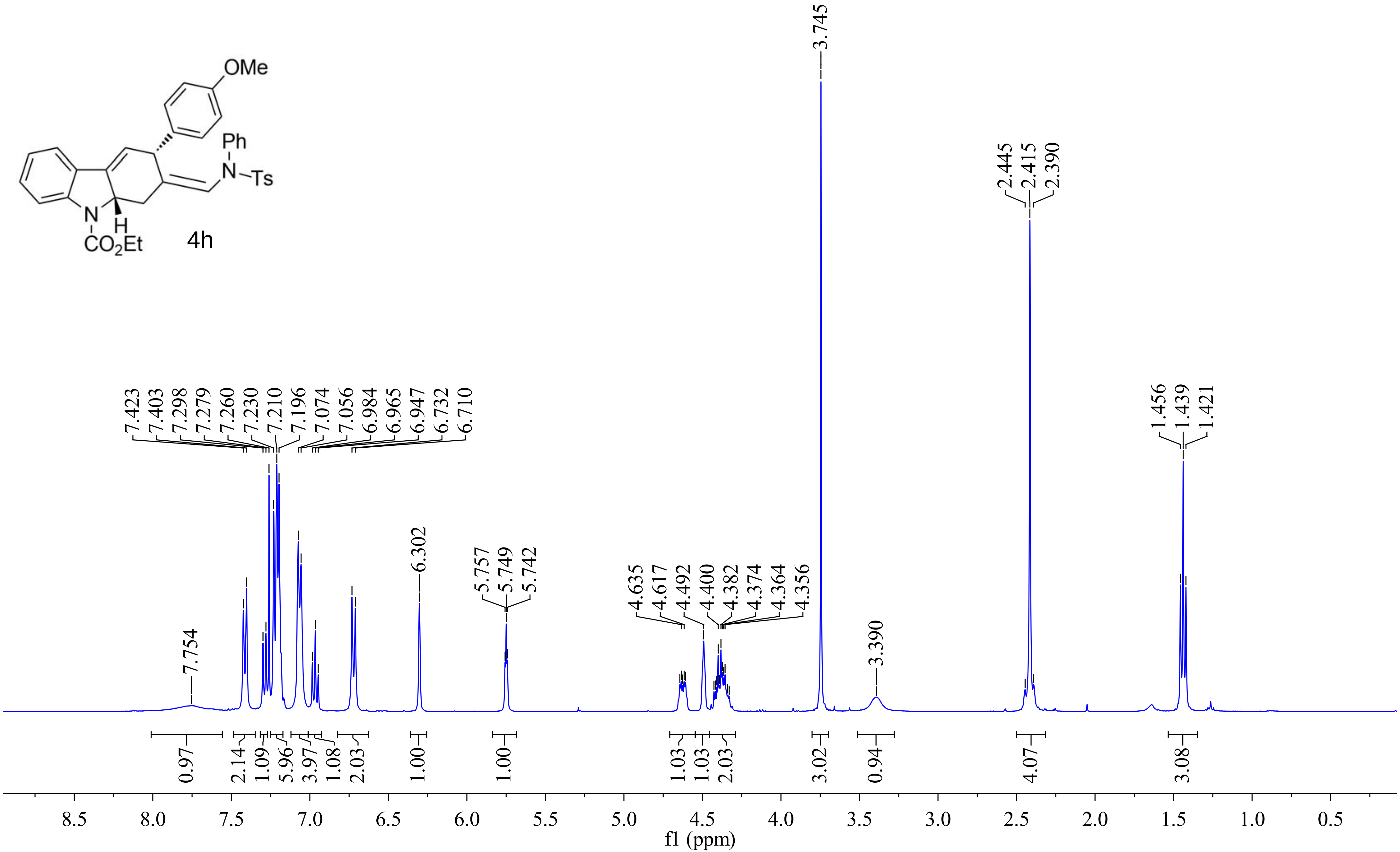

OMe
Ph
N
Ts
N
H
CO2Et
4h
7.754
7.423
7.403
7.298
7.279
7.260
7.230
7.210
7.196
7.074
7.056
6.984
6.965
6.947
6.732
6.710
6.302
5.757
5.749
5.742
4.635
4.617
4.492
4.400
4.382
4.374
4.364
4.356
3.745
3.390
2.445
2.415
2.390
1.456
1.439
1.421
0.97
2.14
1.09
5.96
3.97
1.08
2.03
1.00
1.00
1.03
1.03
2.03
3.02
0.94
4.07
3.08
8.5
8.0
7.5
7.0
6.5
6.0
5.5
5.0
4.5
4.0
3.5
3.0
2.5
2.0
1.5
1.0
0.5
f1 (ppm)

wyd-6-148-1 C

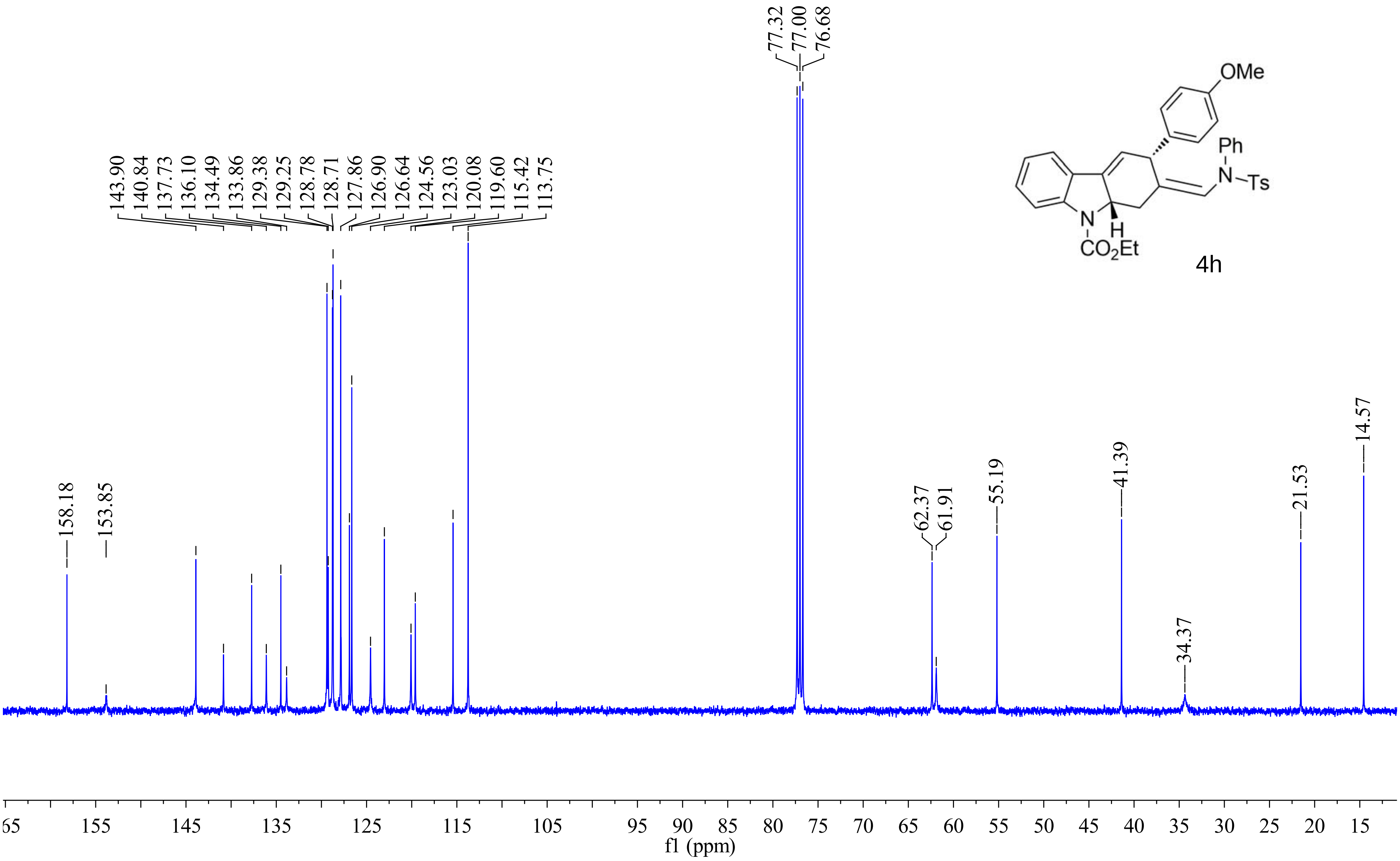

143.90
140.84
137.73
136.10
134.49
133.86
129.38
129.25
128.78
128.71
127.86
126.90
126.64
124.56
123.03
120.08
119.60
115.42
113.75
77.32
77.00
76.68
158.18
153.85
62.37
61.91
55.19
41.39
34.37
21.53
14.57
OMe
Ph
N
Ts
N
H
CO_2Et
4h
65
155
145
135
125
115
105
95
90
85
80
75
70
65
60
55
50
45
40
35
30
25
20
15
f1 (ppm)

wyd-6-90-1 H

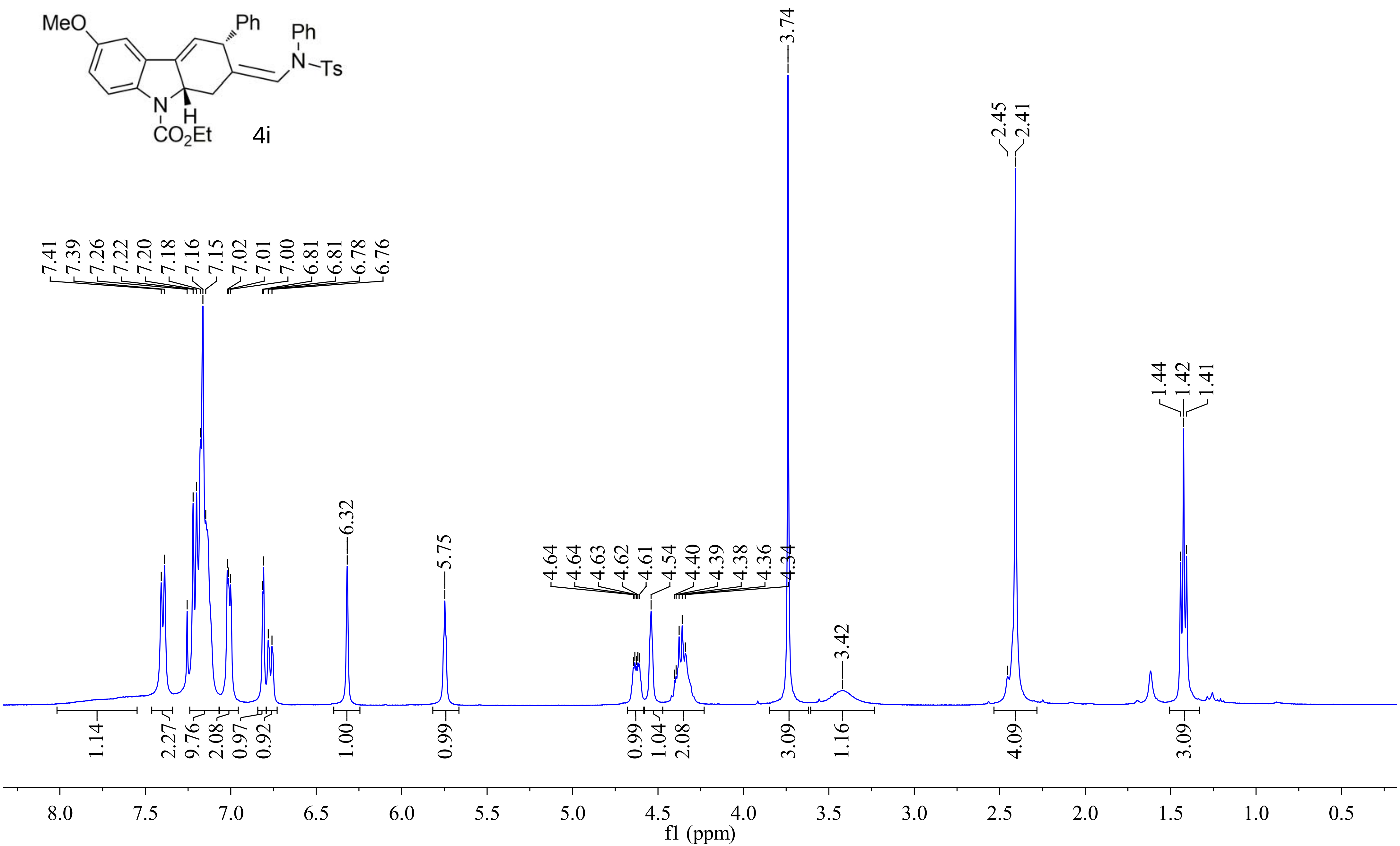
MeO
Ph
Ph
N
Ts
N
H
CO2Et
4i
7.41 7.39 7.26 7.22 7.20 7.18 7.16 7.15 7.02 7.01 7.00 6.81 6.81 6.78 6.76
6.32
5.75
4.64 4.64 4.63 4.62 4.61 4.54 4.40 4.39 4.38 4.36 4.34
3.74
3.42
2.45 2.41
1.44 1.42 1.41
1.14 2.27 9.76 2.08 0.97 0.92 1.00 0.99 0.99 1.04 2.08 3.09 1.16 4.09 3.09
8.0 7.5 7.0 6.5 6.0 5.5 5.0 4.5 4.0 3.5 3.0 2.5 2.0 1.5 1.0 0.5
f1 (ppm)

wyd-6-90-1 C

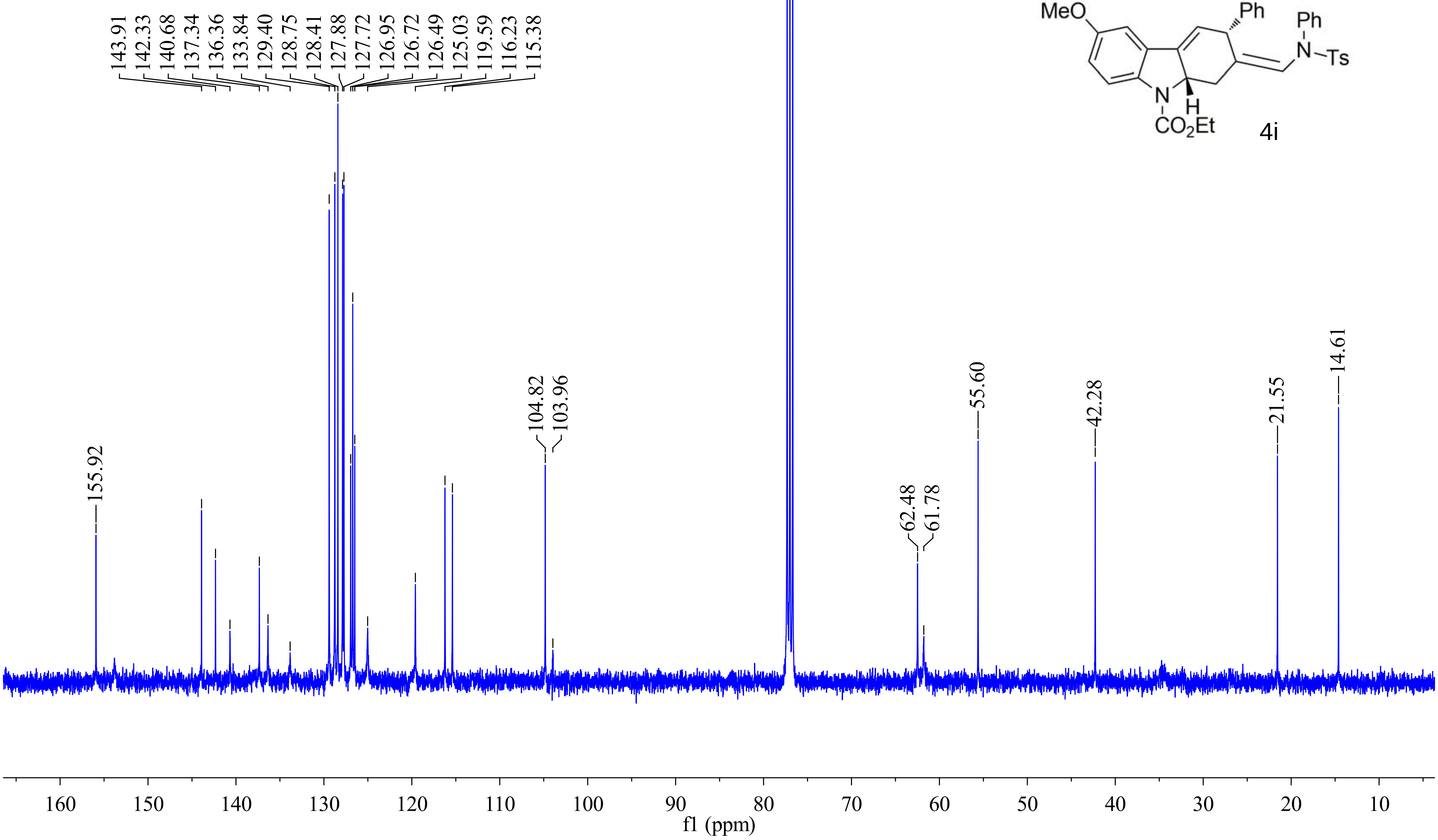

143.91
142.33
140.68
137.34
136.36
133.84
129.40
128.75
128.41
127.88
127.72
126.95
126.72
126.49
125.03
119.59
116.23
115.38
155.92
104.82
103.96
62.48
61.78
55.60
42.28
21.55
14.61
MeO
Ph
Ph
N
Ts
N
H
CO_2Et
4i
160
150
140
130
120
110
100
90
80
70
60
50
40
30
20
10
f1 (ppm)

wyd-7-16-1 H

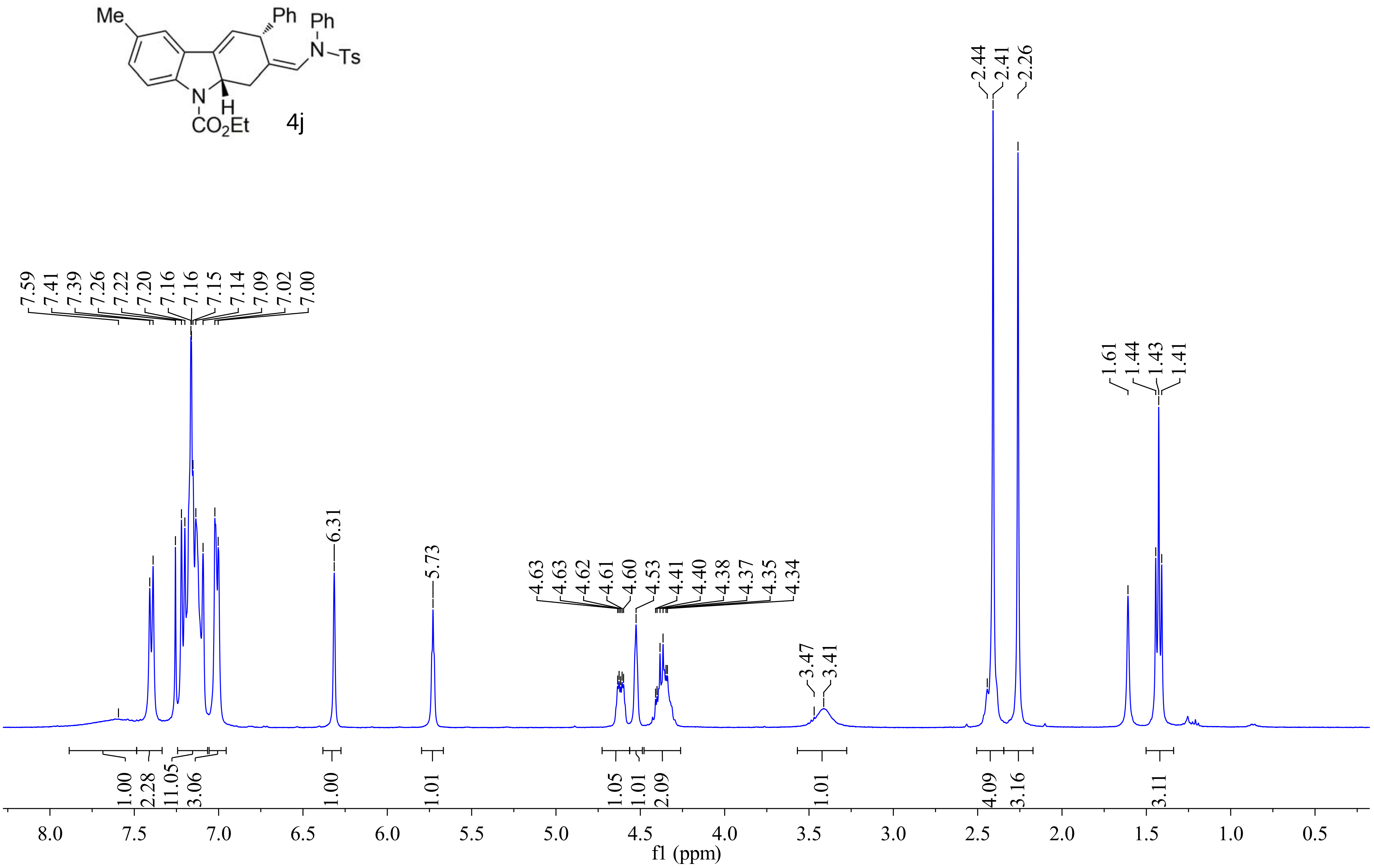
Me
Ph
Ph
N
Ts
N
H
CO2Et
4j
7.59 7.41 7.39 7.26 7.22 7.20 7.16 7.16 7.15 7.14 7.09 7.02 7.00
6.31
5.73
4.63 4.63 4.62 4.61 4.60 4.53 4.41 4.40 4.38 4.37 4.35 4.34
3.47 3.41
2.44 2.41 2.26
1.61 1.44 1.43 1.41
1.00 2.28 11.05 3.06
1.00
1.01
1.05 1.01 2.09
1.01
4.09 3.16
3.11
8.0 7.5 7.0 6.5 6.0 5.5 5.0 4.5 4.0 3.5 3.0 2.5 2.0 1.5 1.0 0.5
f1 (ppm)

wyd-7-16-1 C

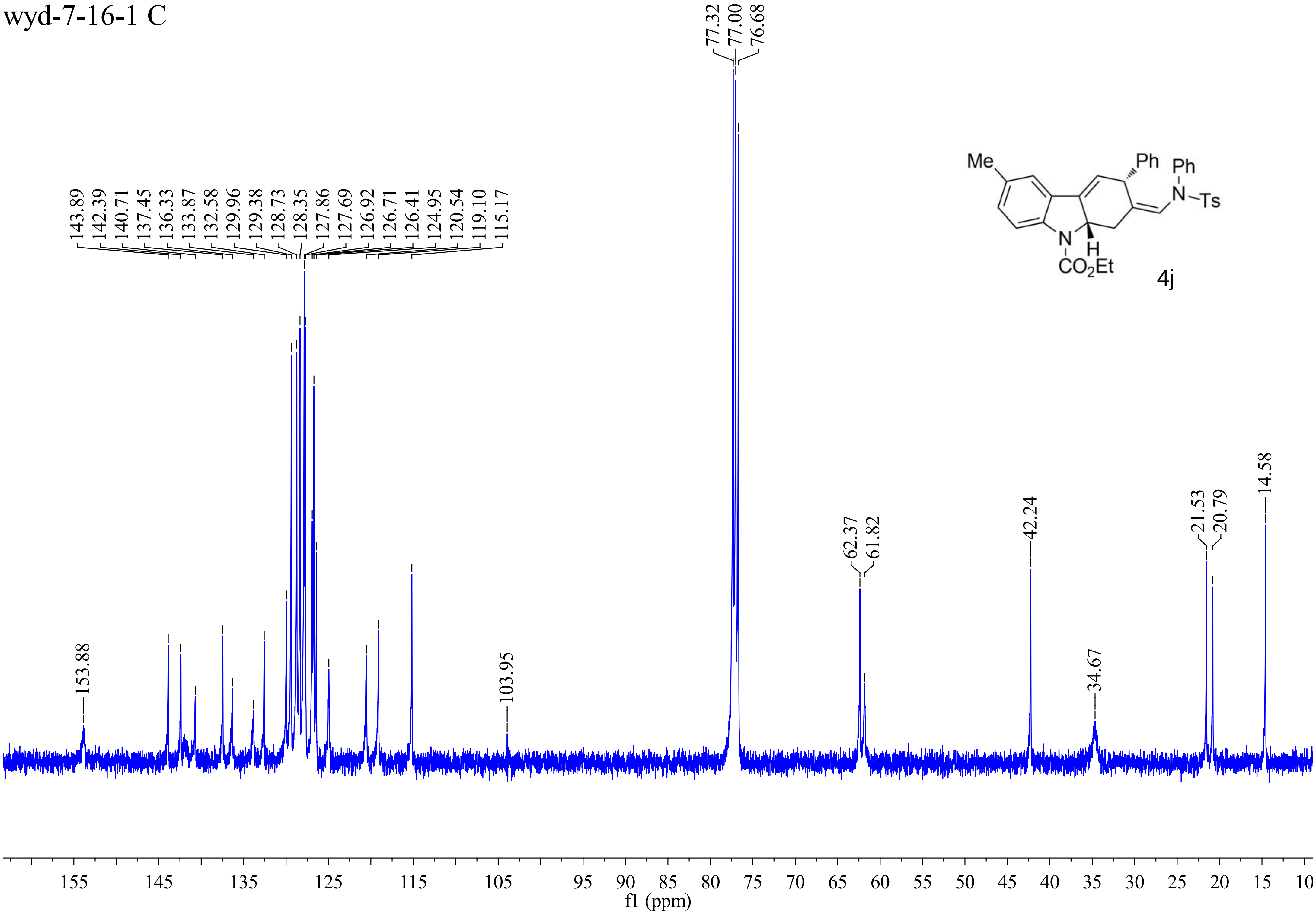

77.32
77.00
76.68
143.89
142.39
140.71
137.45
136.33
133.87
132.58
129.96
129.38
128.73
128.35
127.86
127.69
126.92
126.71
126.41
124.95
120.54
119.10
115.17
153.88
103.95
62.37
61.82
42.24
34.67
21.53
20.79
14.58
Me
Ph
Ph
N
Ts
N
H
CO_2Et
4j
155 145 135 125 115 105 95 90 85 80 75 70 65 60 55 50 45 40 35 30 25 20 15 10
f1 (ppm)

wyd-6-88-1 H

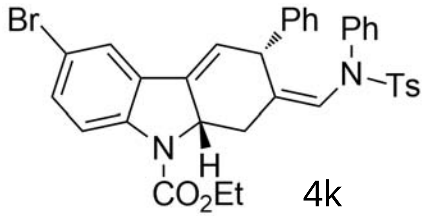
Br
Ph
Ph
N
Ts
N
H
CO2Et

4k

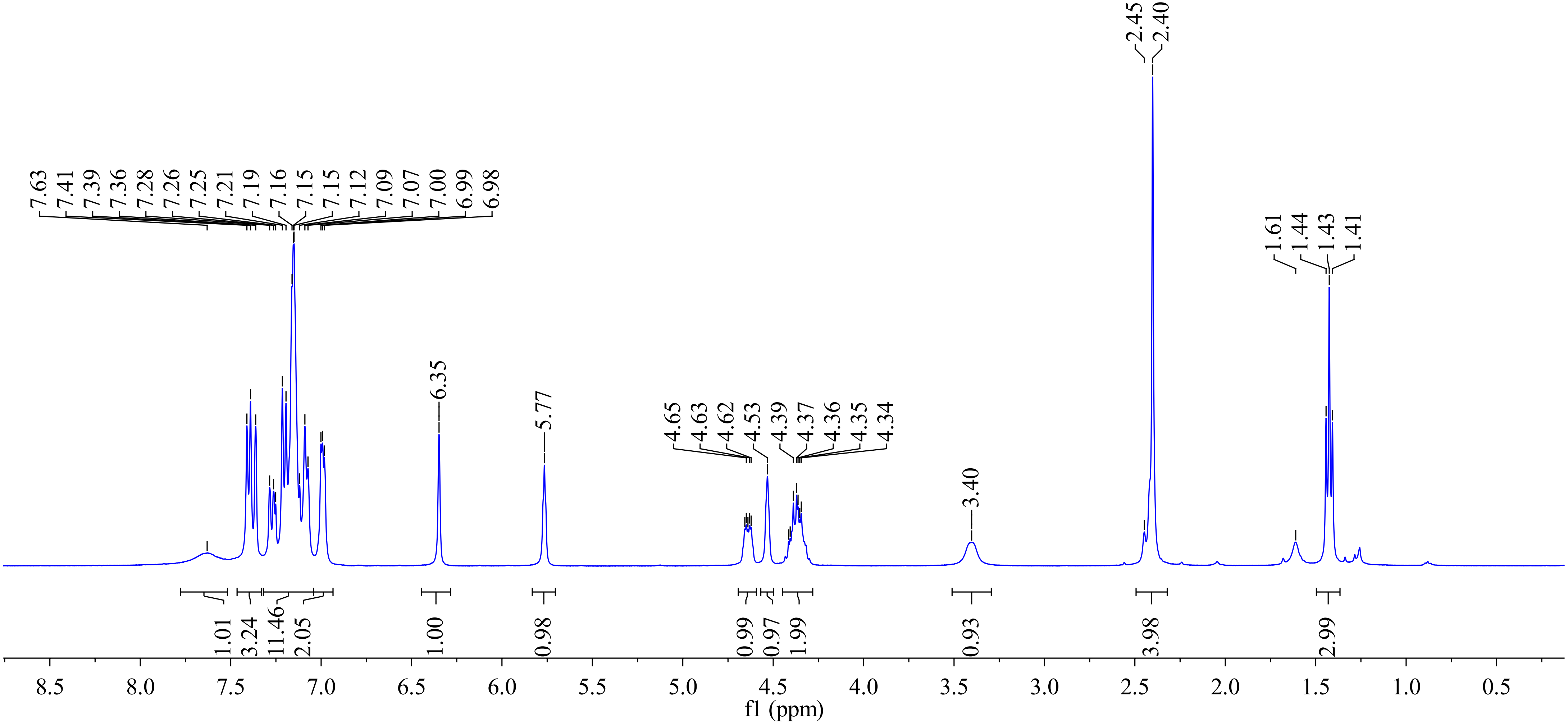
7.63
7.41
7.39
7.36
7.28
7.26
7.25
7.21
7.19
7.16
7.15
7.15
7.12
7.09
7.07
7.00
6.99
6.98
6.35
5.77
4.65
4.63
4.62
4.53
4.39
4.37
4.36
4.35
4.34
3.40
2.45
2.40
1.61
1.44
1.43
1.41
1.01
3.24
11.46
2.05
1.00
0.98
0.99
0.97
1.99
0.93
3.98
2.99
8.5
8.0
7.5
7.0
6.5
6.0
5.5
5.0
4.5
4.0
3.5
3.0
2.5
2.0
1.5
1.0
0.5
f1 (ppm)

wyd-6-88-1 C
wyd-6-88-1 C

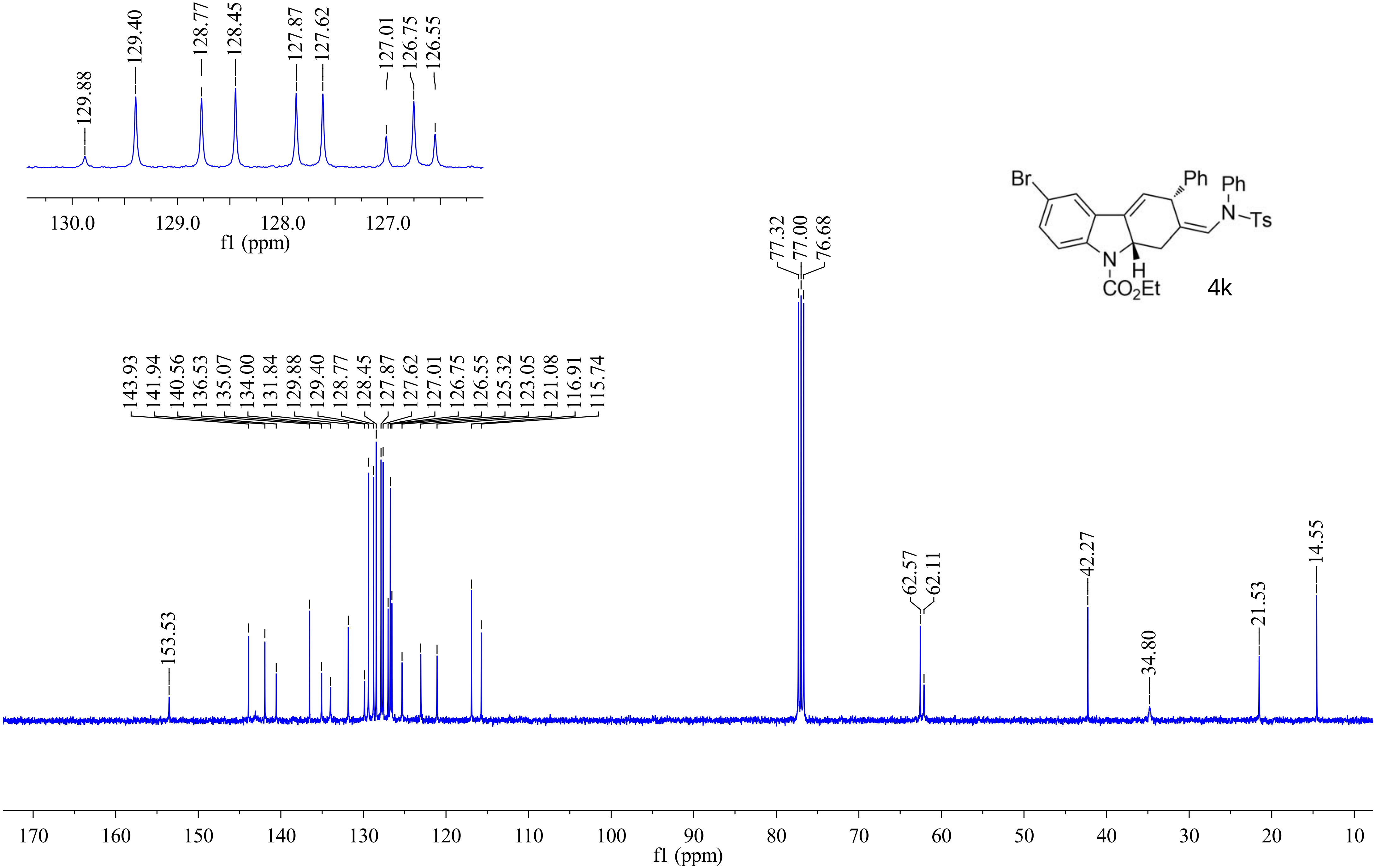

wyd-6-89-1 H

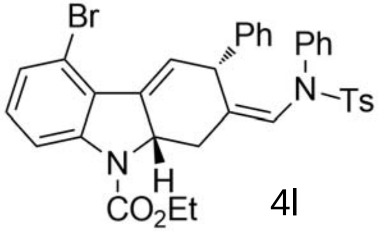

Br
Ph
Ph
N
Ts
N
H
CO2Et

4l

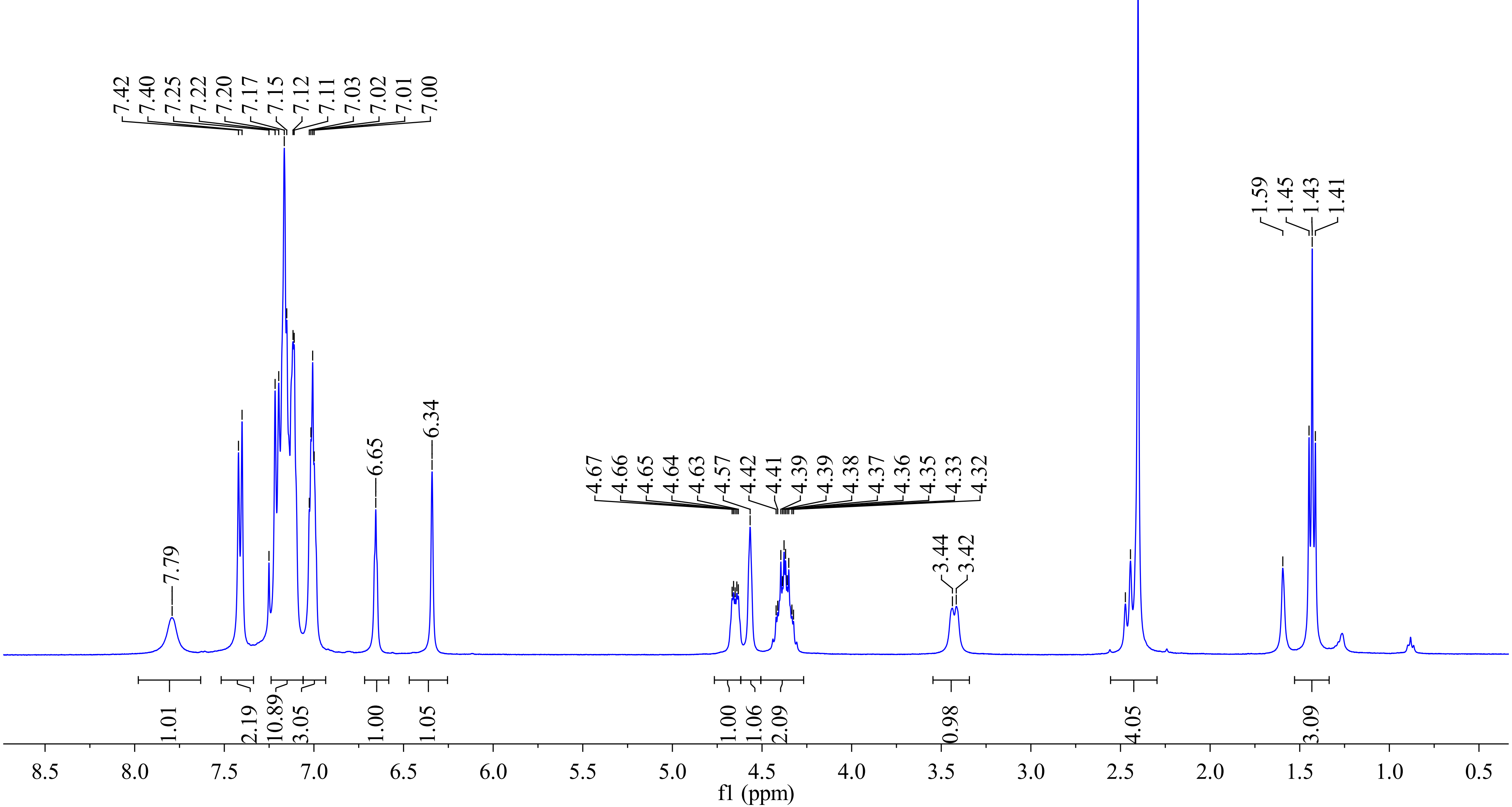

7.42 7.40 7.25 7.22 7.20 7.17 7.15 7.12 7.11 7.03 7.02 7.01 7.00
7.79
6.65
6.34
4.67 4.66 4.65 4.64 4.63 4.57 4.42 4.41 4.39 4.39 4.38 4.37 4.36 4.35 4.33 4.32
3.44 3.42
2.47 2.44 2.40
1.59 1.45 1.43 1.41
1.01 2.19 10.89 3.05 1.00 1.05 1.00 1.06 2.09 0.98 4.05 3.09
8.5 8.0 7.5 7.0 6.5 6.0 5.5 5.0 4.5 4.0 3.5 3.0 2.5 2.0 1.5 1.0 0.5
f1 (ppm)

wyd-6-89-1 C

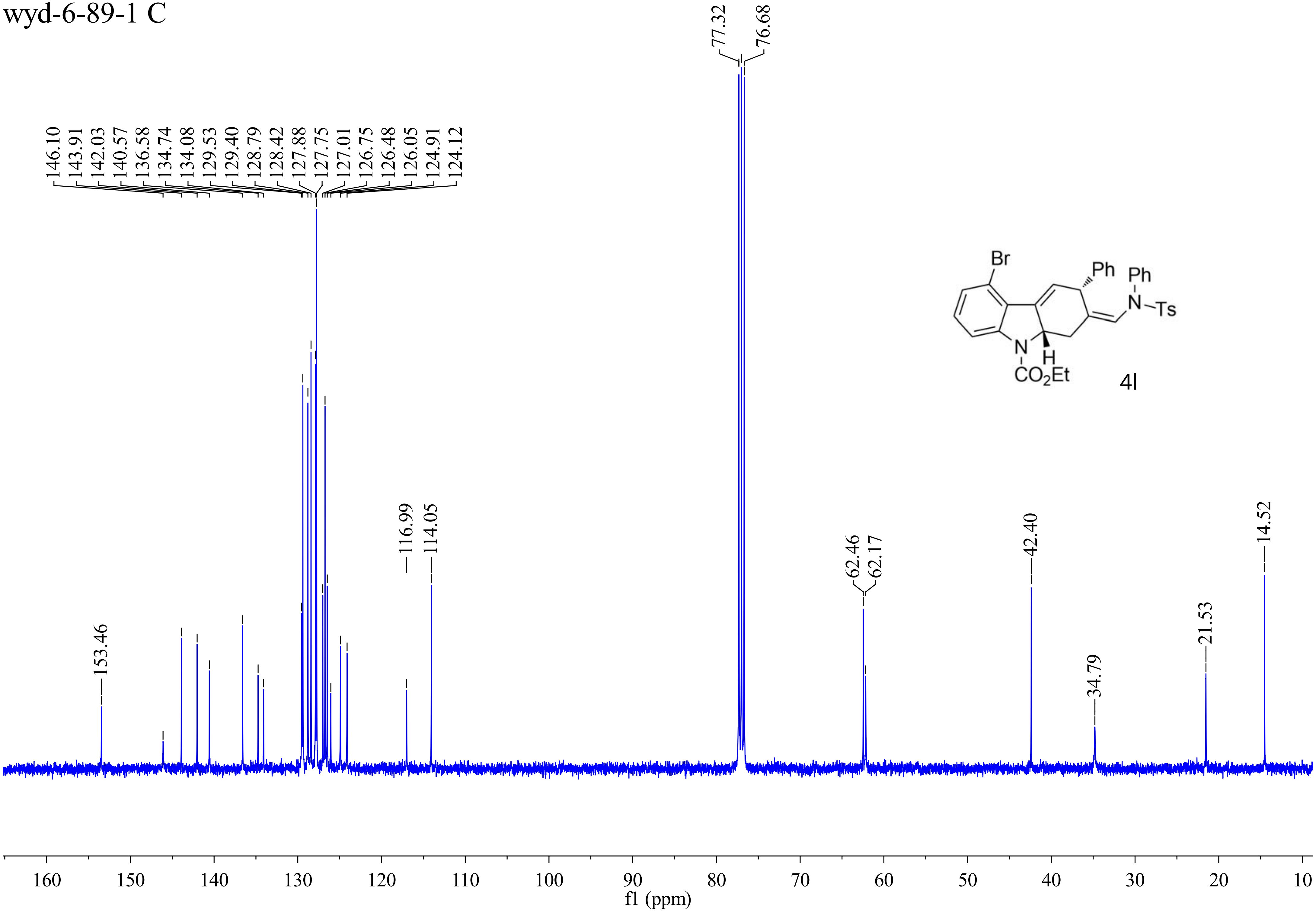

77.32
76.68
146.10
143.91
142.03
140.57
136.58
134.74
134.08
129.53
129.40
128.79
128.42
127.88
127.75
127.01
126.75
126.48
126.05
124.91
124.12
116.99
114.05
153.46
62.46
62.17
42.40
34.79
21.53
14.52
Br
Ph
Ph
N
Ts
N
H
CO_2Et
4l
160
150
140
130
120
110
100
90
80
70
60
50
40
30
20
10
f1 (ppm)

wyd-6-145-1 H

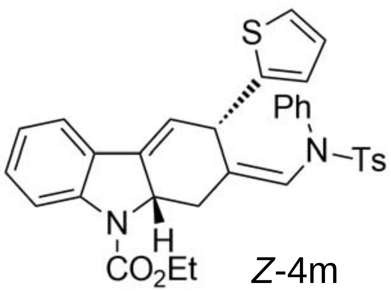

Z-4m

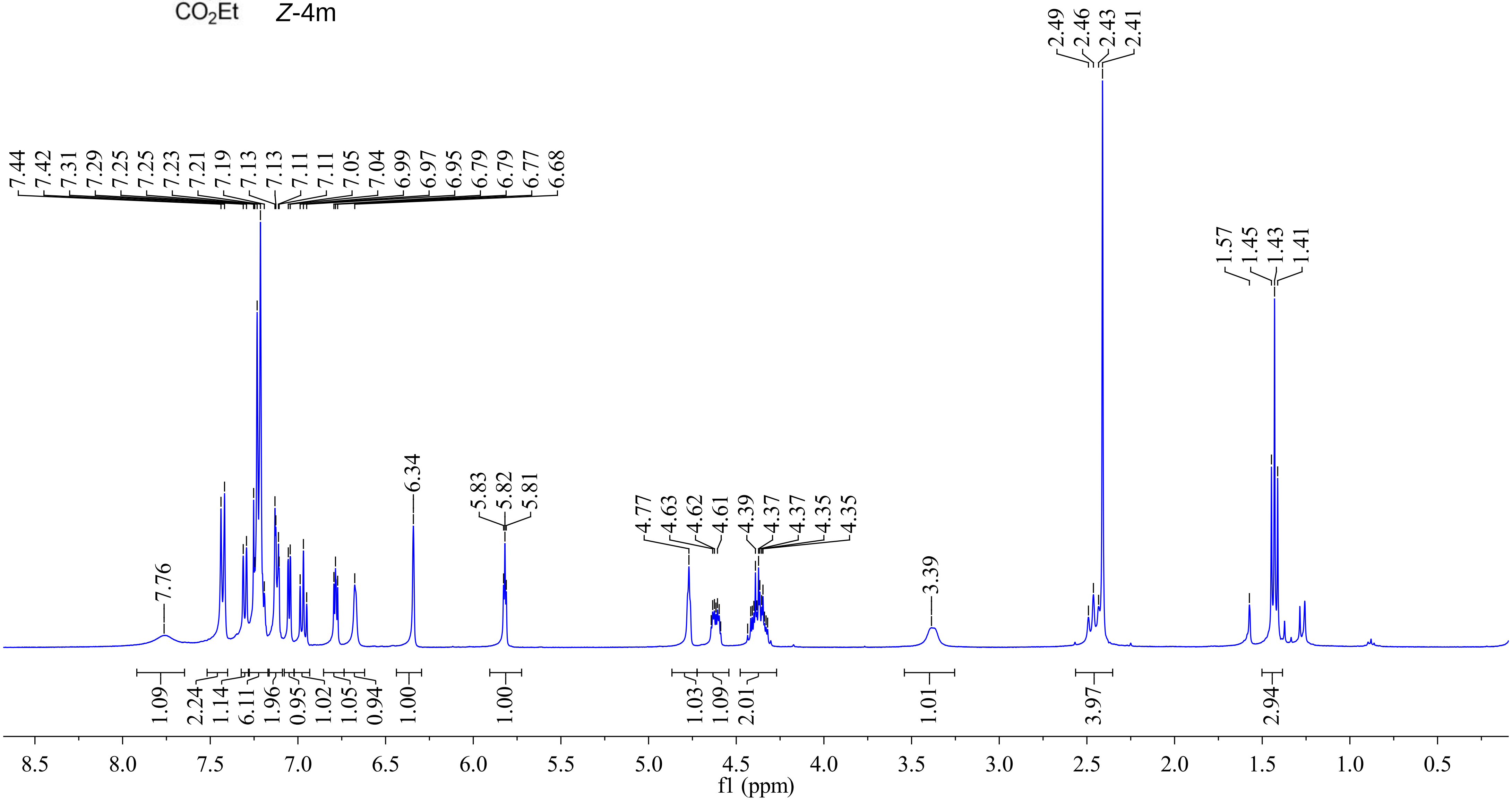

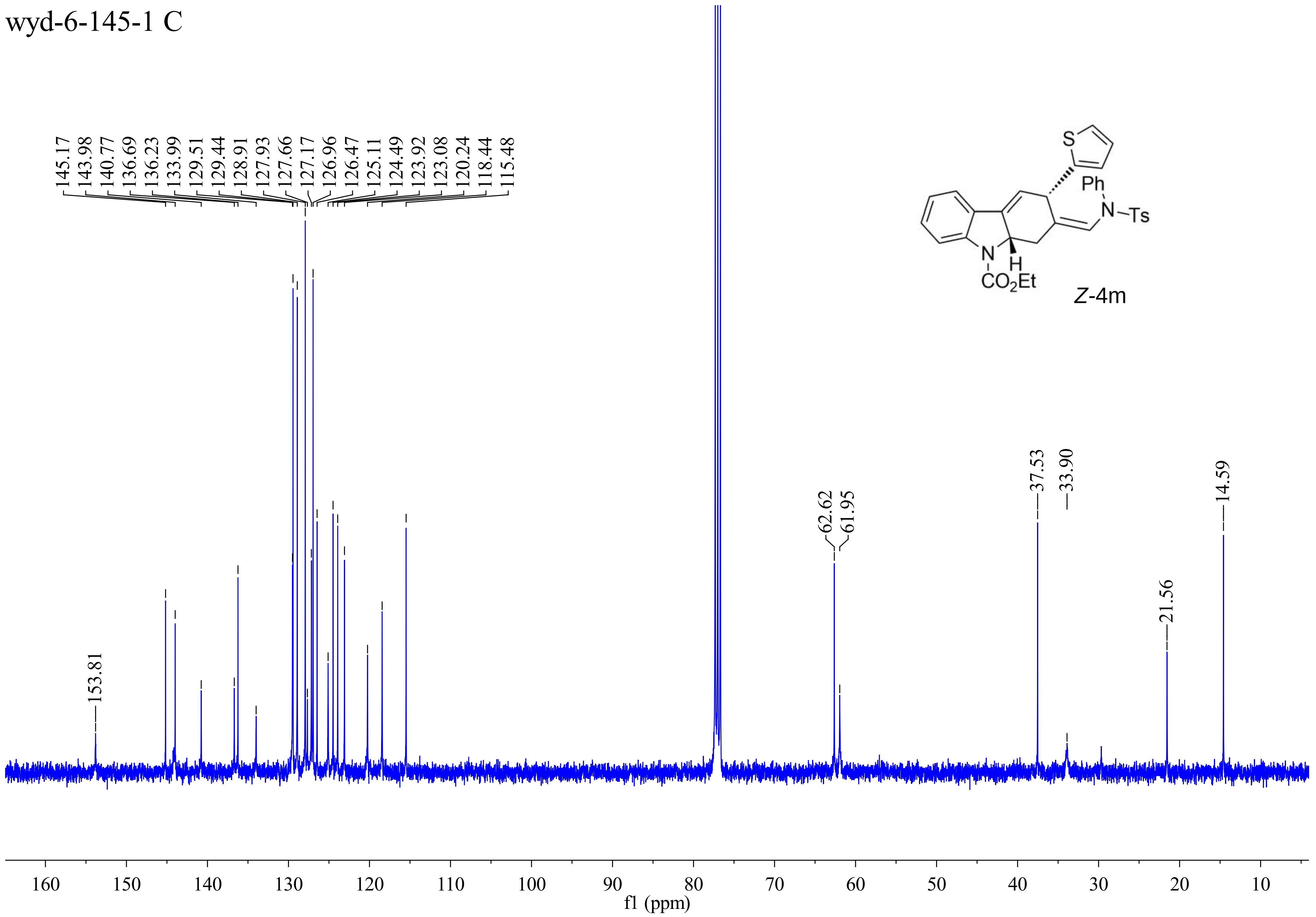

wyd-6-145-1 C
145.17
143.98
140.77
136.69
136.23
133.99
129.51
129.44
128.91
127.93
127.66
127.17
126.96
126.47
125.11
124.49
123.92
123.08
120.24
118.44
115.48
153.81
62.62
61.95
37.53
33.90
21.56
14.59
S
Ph
N
Ts
N
H
CO2Et
Z-4m
160
150
140
130
120
110
100
90
80
70
60
50
40
30
20
10
f1 (ppm)

wyd-6-145-2 H

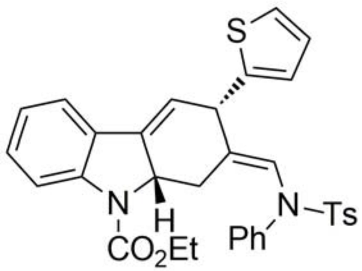

E-4m

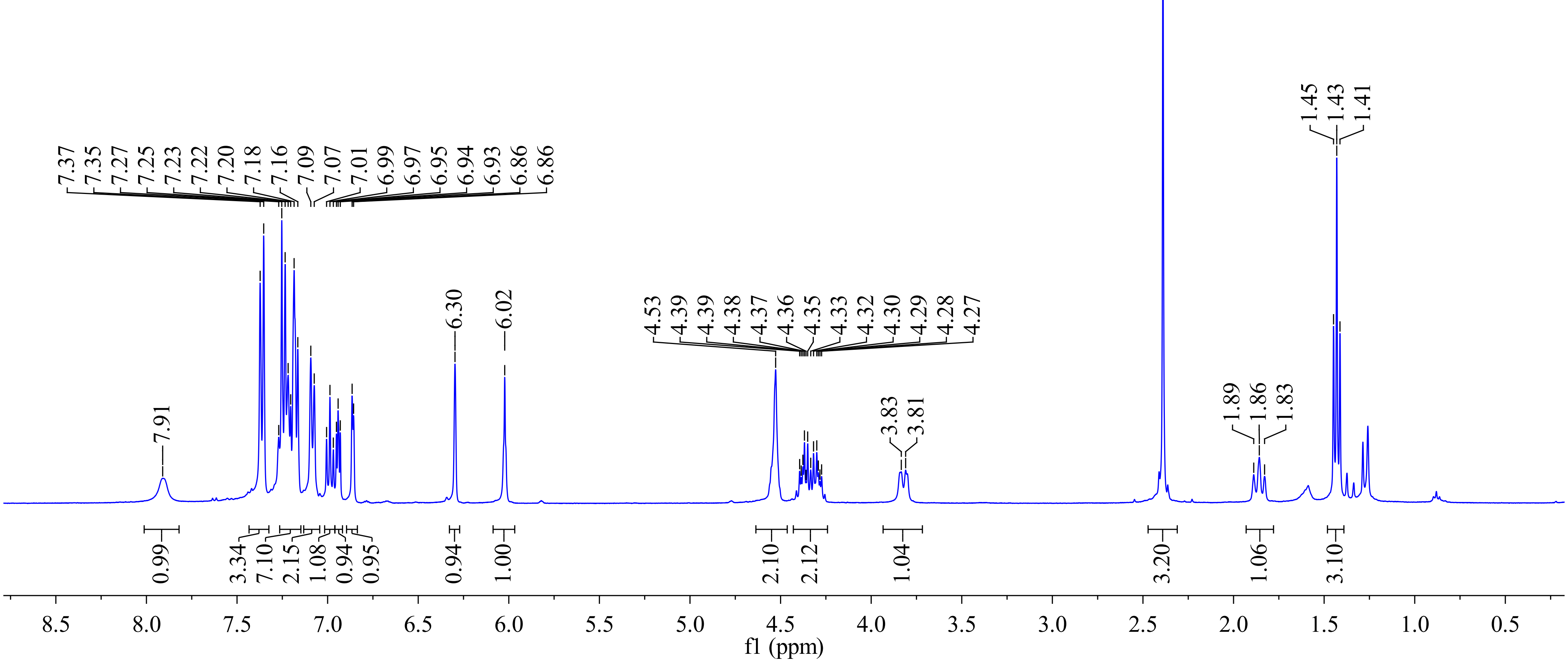

wyd-6-145-2 C

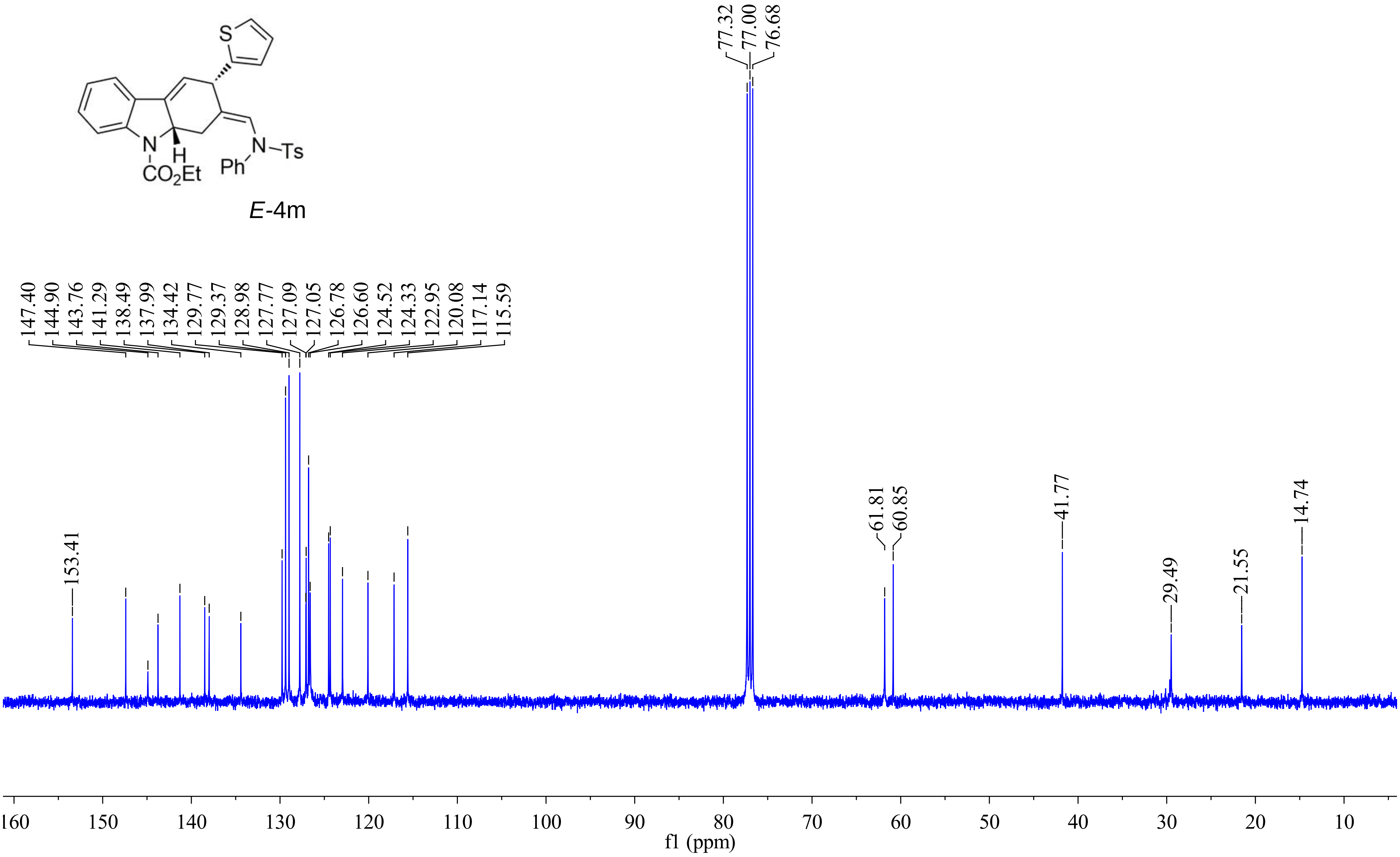

S
N
H
CO2Et
N
Ts
Ph
E-4m
77.32
77.00
76.68
147.40
144.90
143.76
141.29
138.49
137.99
134.42
129.77
129.37
128.98
127.77
127.09
127.05
126.78
126.60
124.52
124.33
122.95
120.08
117.14
115.59
153.41
61.81
60.85
41.77
29.49
21.55
14.74
160
150
140
130
120
110
100
90
80
70
60
50
40
30
20
10
f1 (ppm)

wyd-6-91-1 H

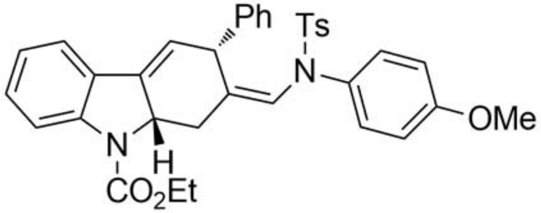
Ph
Ts
N
OMe
N
H
CO2Et

4p

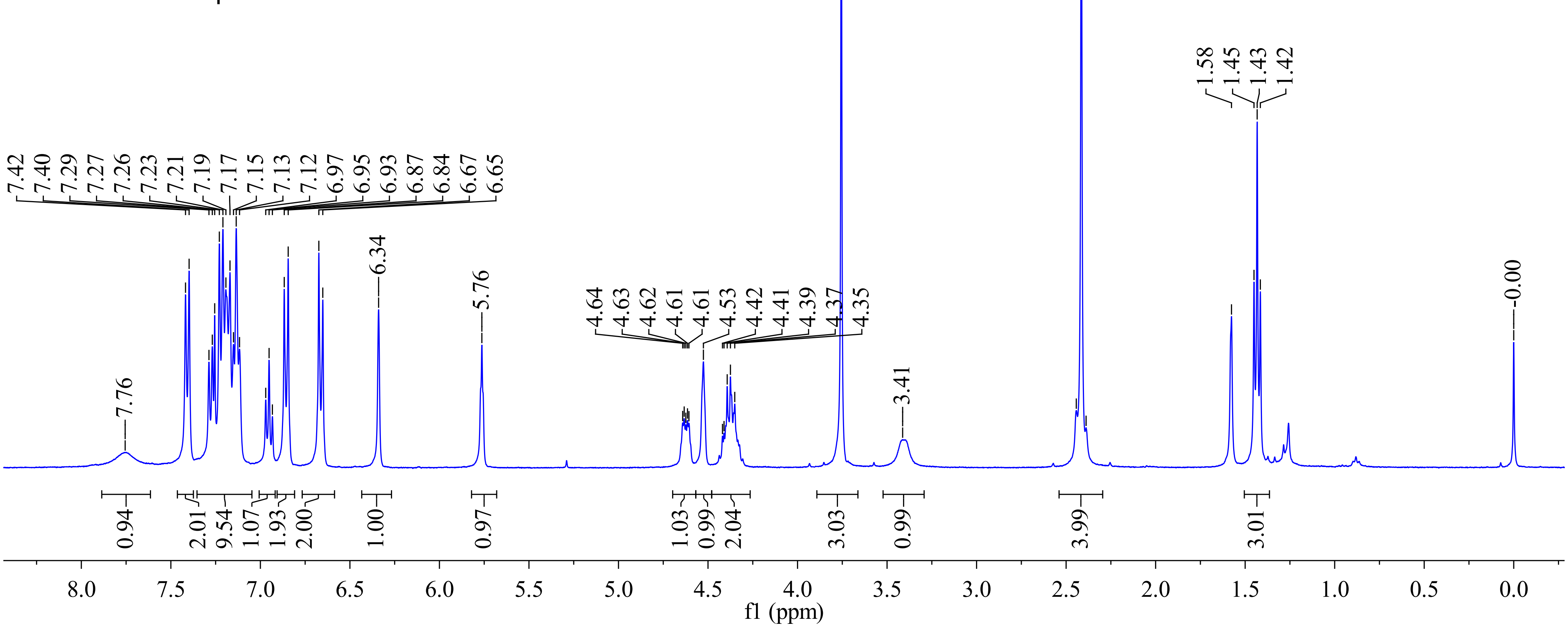
3.76
2.44
2.41
2.39
1.58
1.45
1.43
1.42
7.42
7.40
7.29
7.27
7.26
7.23
7.21
7.19
7.17
7.15
7.13
7.12
6.97
6.95
6.93
6.87
6.84
6.67
6.65
6.34
5.76
4.64
4.63
4.62
4.61
4.61
4.53
4.42
4.41
4.39
4.37
4.35
3.41
-0.00
7.76
0.94
2.01
9.54
1.07
1.93
2.00
1.00
0.97
1.03
0.99
2.04
3.03
0.99
3.99
3.01
8.0
7.5
7.0
6.5
6.0
5.5
5.0
4.5
4.0
3.5
3.0
2.5
2.0
1.5
1.0
0.5
0.0
f1 (ppm)

wyd-6-91-1 C

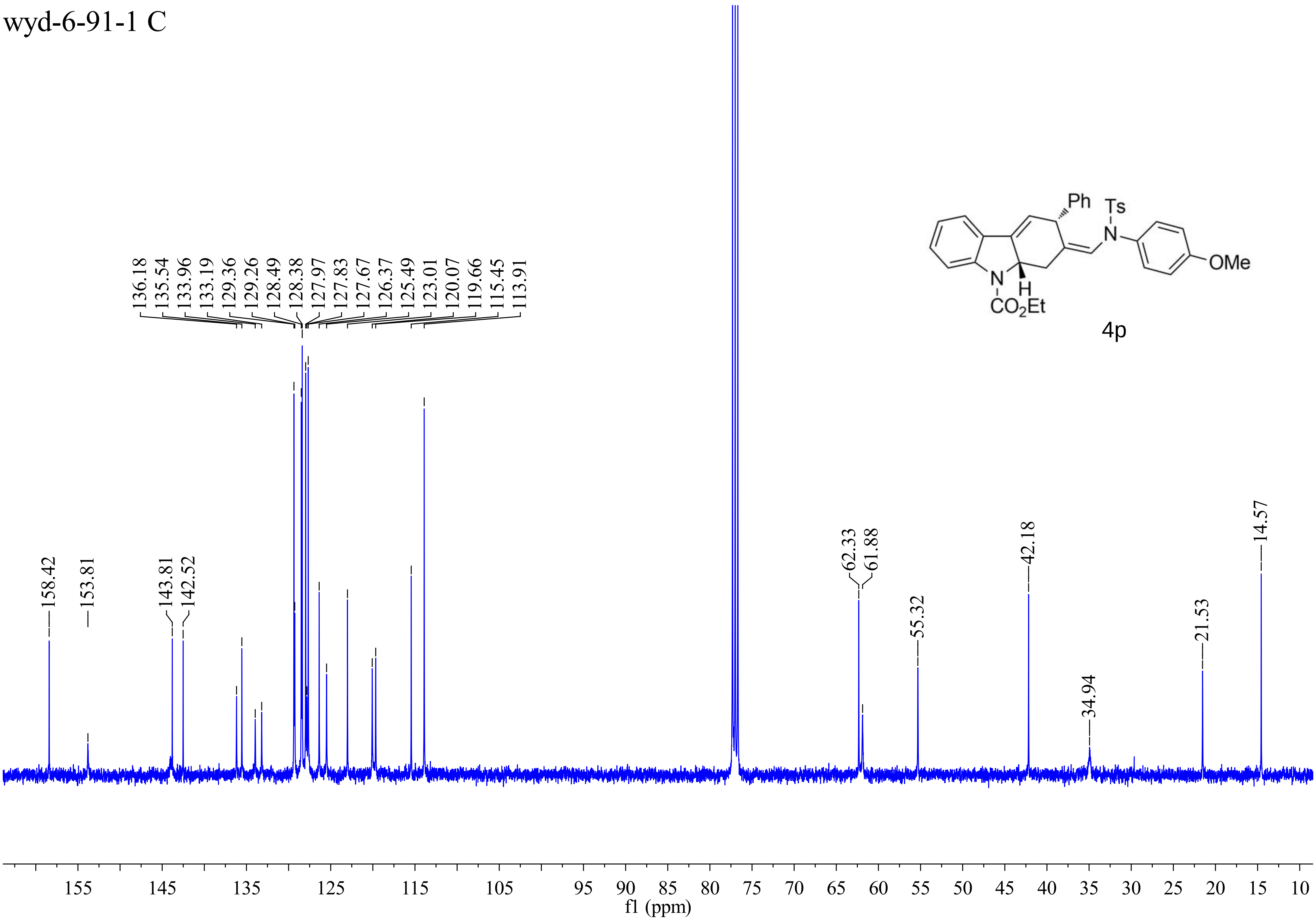
158.42
153.81
143.81
142.52
136.18
135.54
133.96
133.19
129.36
129.26
128.49
128.38
127.97
127.83
127.67
126.37
125.49
123.01
120.07
119.66
115.45
113.91
62.33
61.88
55.32
42.18
34.94
21.53
14.57
Ph
Ts
N
OMe
N
H
CO2Et
4p
155
145
135
125
115
105
95
90
85
80
75
70
65
60
55
50
45
40
35
30
25
20
15
10
f1 (ppm)

wyd-6-92-1 H

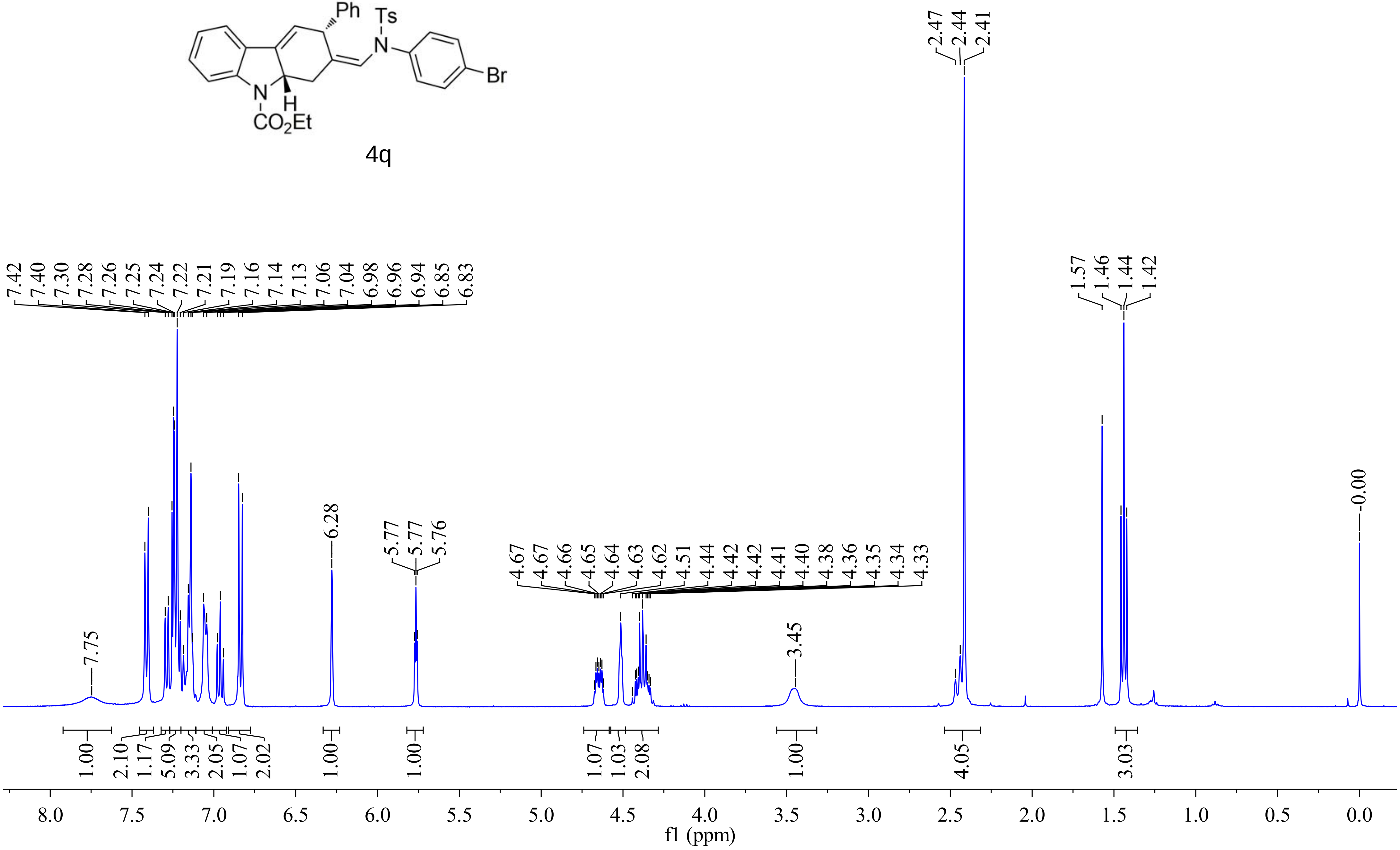
Ph
Ts
N
Br
N
H
CO2Et
4q
7.42 7.40 7.30 7.28 7.26 7.25 7.24 7.22 7.21 7.19 7.16 7.14 7.13 7.06 7.04 6.98 6.96 6.94 6.85 6.83
7.75
6.28
5.77 5.77 5.76
4.67 4.67 4.66 4.65 4.64 4.63 4.62 4.51 4.44 4.42 4.42 4.41 4.40 4.38 4.36 4.35 4.34 4.33
3.45
2.47 2.44 2.41
1.57 1.46 1.44 1.42
-0.00
1.00 2.10 1.17 5.09 3.33 2.05 1.07 2.02 1.00 1.00 1.07 1.03 2.08 1.00 4.05 3.03
8.0 7.5 7.0 6.5 6.0 5.5 5.0 4.5 4.0 3.5 3.0 2.5 2.0 1.5 1.0 0.5 0.0
f1 (ppm)

wyd-6-92-1 C

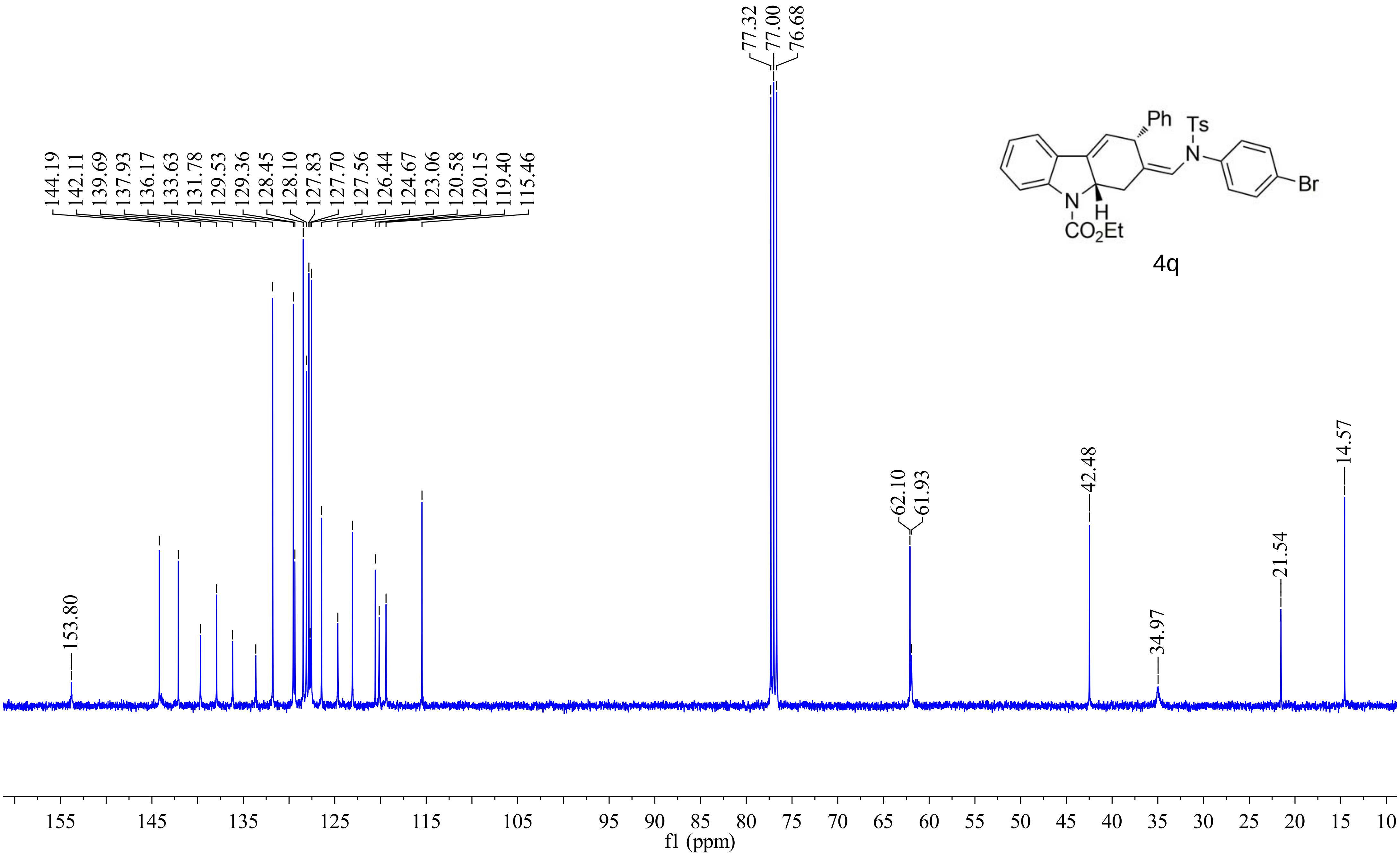

77.32
77.00
76.68
144.19
142.11
139.69
137.93
136.17
133.63
131.78
129.53
129.36
128.45
128.10
127.83
127.70
127.56
126.44
124.67
123.06
120.58
120.15
119.40
115.46
153.80
62.10
61.93
42.48
34.97
21.54
14.57
Ph
Ts
N
Br
N
H
CO_2Et
4q
155 145 135 125 115 105 95 90 85 80 75 70 65 60 55 50 45 40 35 30 25 20 15 10
f1 (ppm)

wyd-6-124 H

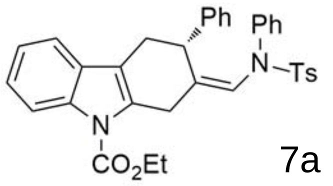
Ph
Ph
N
Ts
N
CO2Et
7a

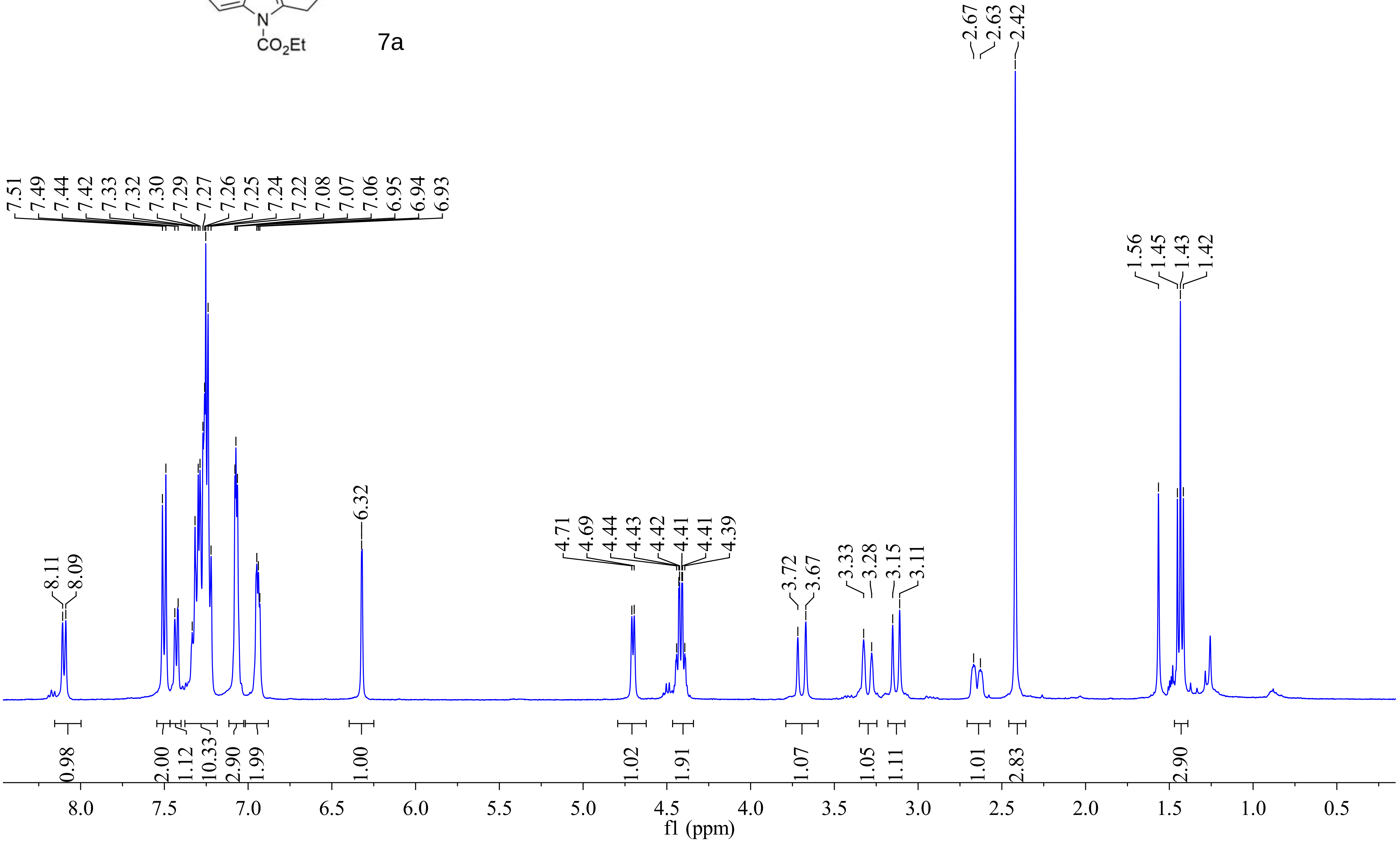
7.51 7.49 7.44 7.42 7.33 7.32 7.30 7.29 7.27 7.26 7.25 7.24 7.22 7.08 7.07 7.06 6.95 6.94 6.93
8.11 8.09
6.32
4.71 4.69 4.44 4.43 4.42 4.41 4.41 4.39
3.72 3.67
3.33 3.28 3.15 3.11
2.67 2.63 2.42
1.56 1.45 1.43 1.42
0.98 2.00 1.12 10.33 2.90 1.99 1.00 1.02 1.91 1.07 1.05 1.11 1.01 2.83 2.90
8.0 7.5 7.0 6.5 6.0 5.5 5.0 4.5 4.0 3.5 3.0 2.5 2.0 1.5 1.0 0.5
f1 (ppm)

wyd-6-124 C

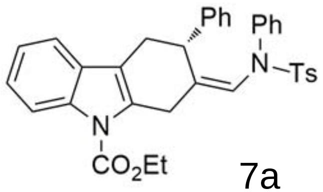
Ph
Ph
N
Ts
N
CO2Et

7a

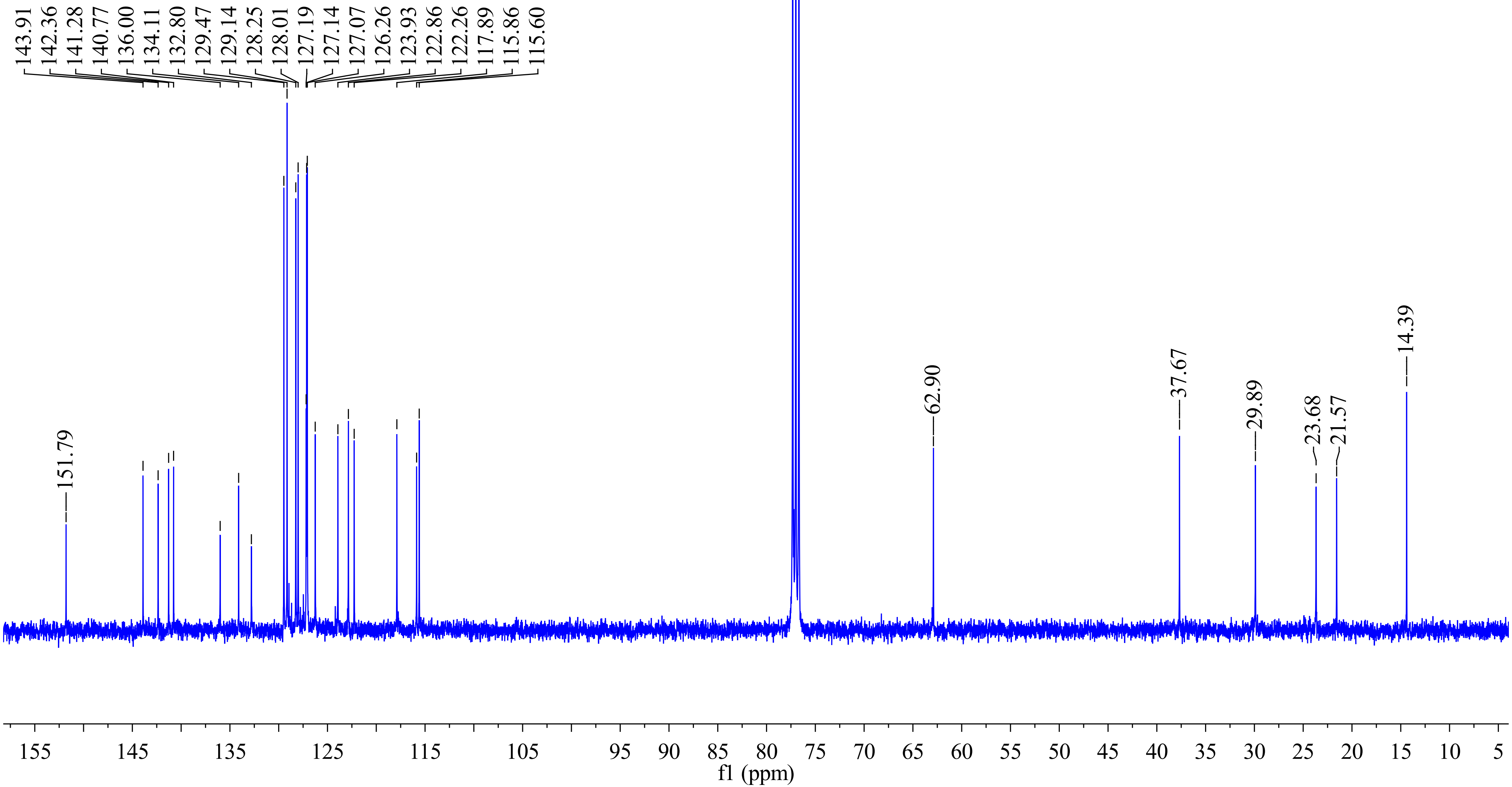
143.91
142.36
141.28
140.77
136.00
134.11
132.80
129.47
129.14
128.25
128.01
127.19
127.14
127.07
126.26
123.93
122.86
122.26
117.89
115.86
115.60
151.79
62.90
37.67
29.89
23.68
21.57
14.39
155 145 135 125 115 105 95 90 85 80 75 70 65 60 55 50 45 40 35 30 25 20 15 10 5
f1 (ppm)

wyd-6-130-2 H

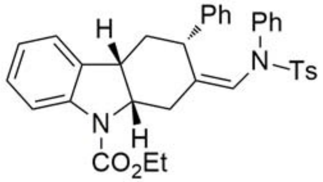

6a

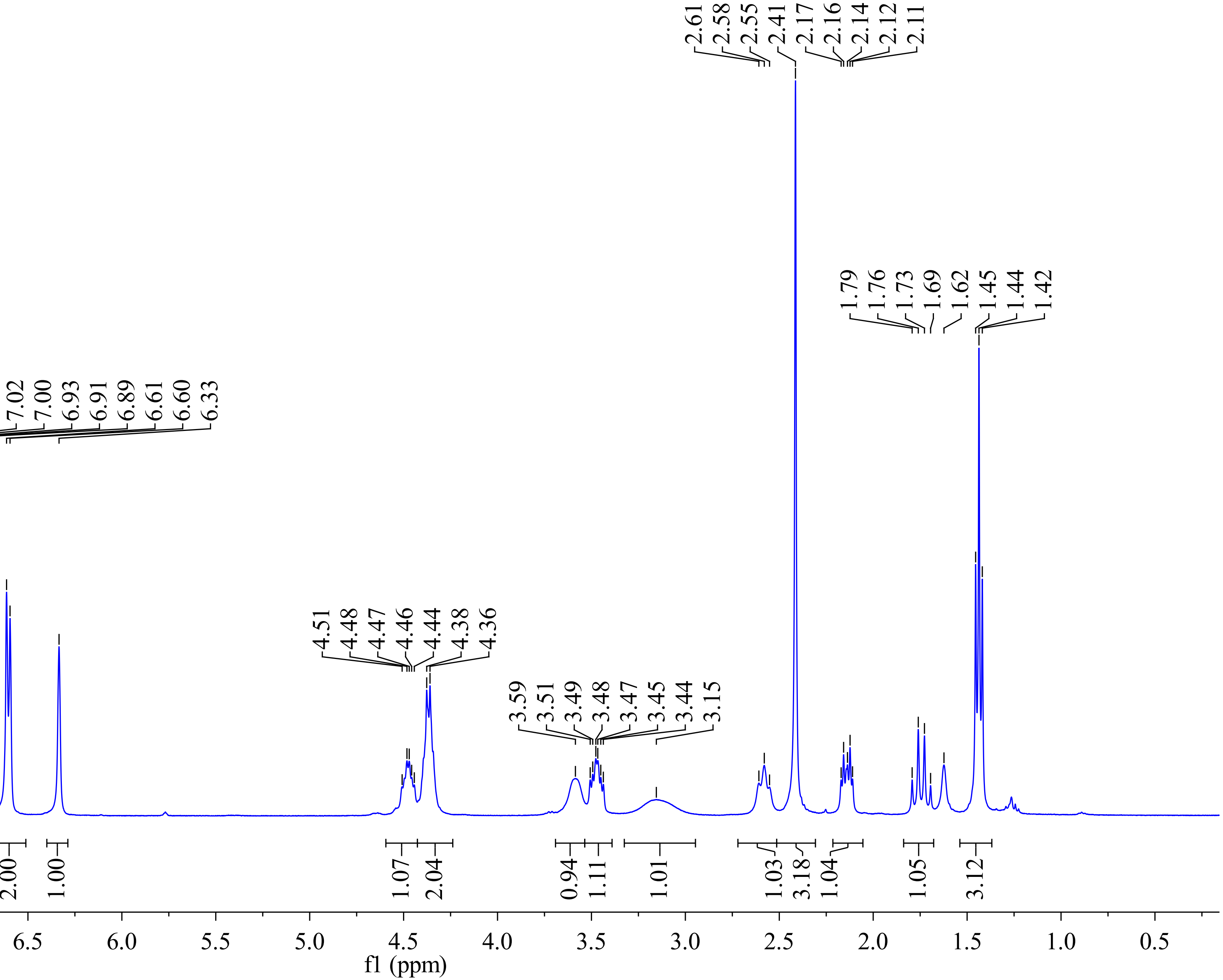

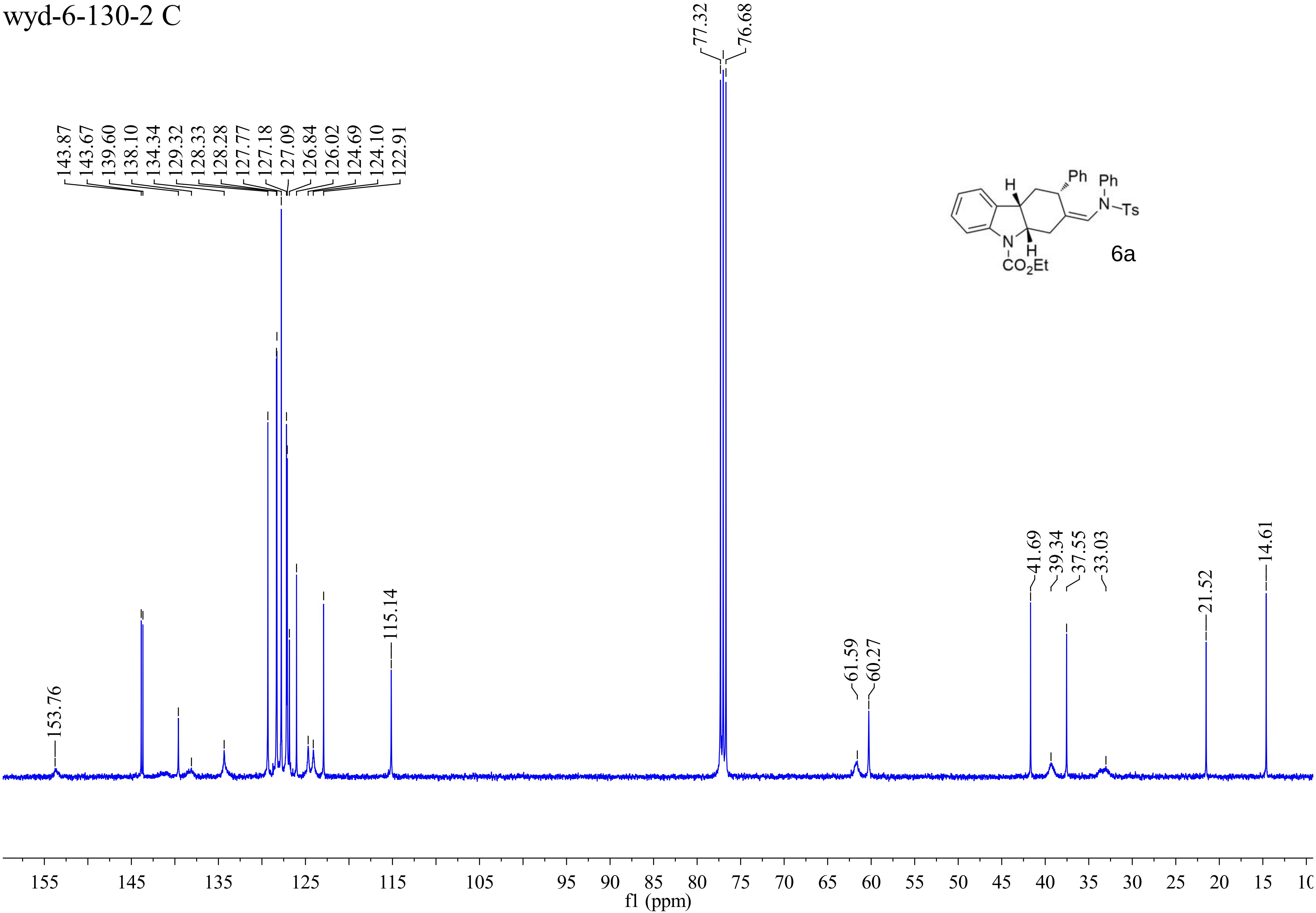
wyd-6-130-2 C
143.87
143.67
139.60
138.10
134.34
129.32
128.33
128.28
127.77
127.18
127.09
126.84
126.02
124.69
124.10
122.91
77.32
76.68
153.76
115.14
61.59
60.27
41.69
39.34
37.55
33.03
21.52
14.61
H
H
Ph
Ph
N
Ts
N
CO2Et
6a
155
145
135
125
115
105
95
90
85
80
75
70
65
60
55
50
45
40
35
30
25
20
15
10
f1 (ppm)

WYD-7-48-1

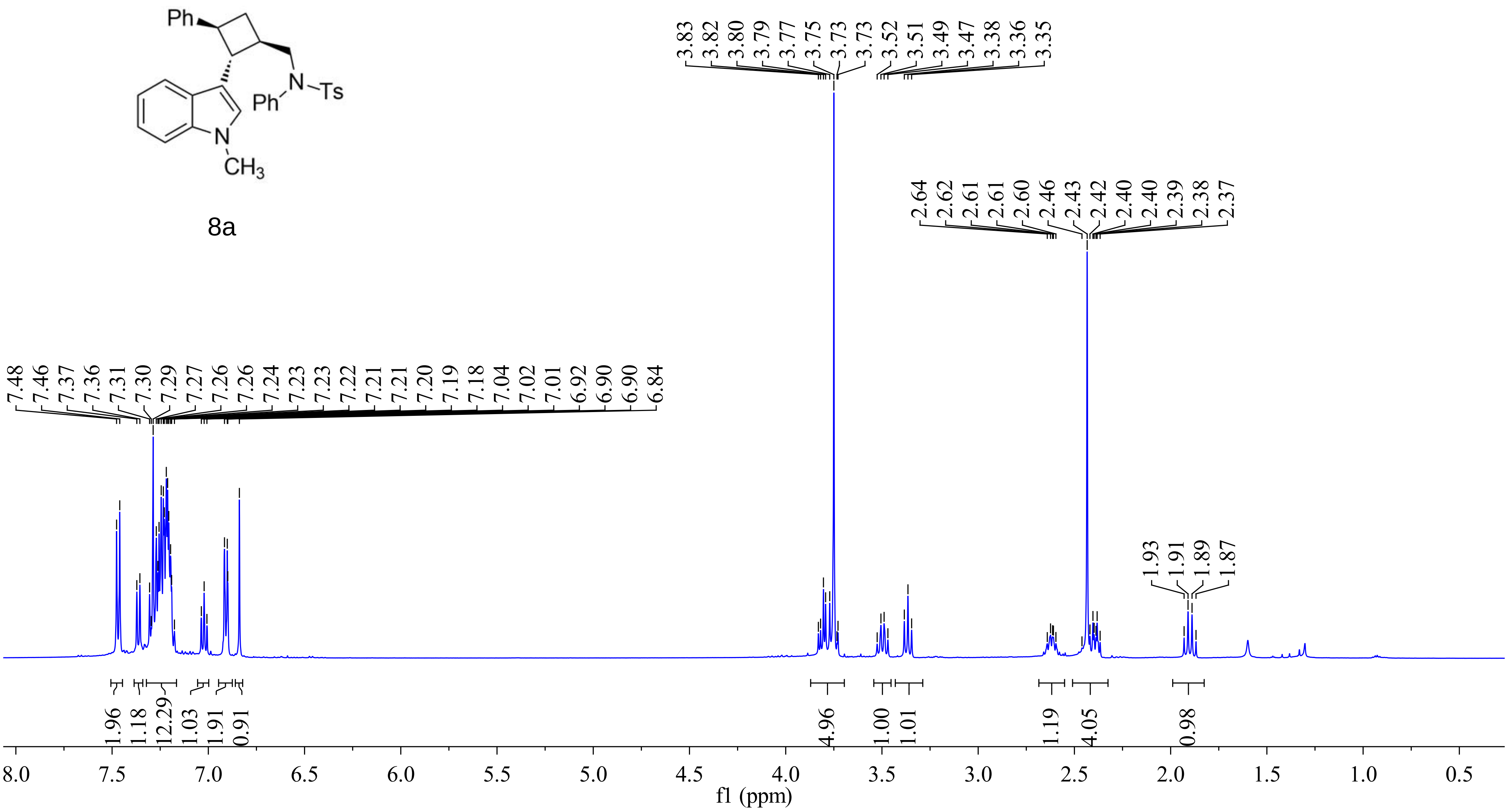
Ph
N
Ts
Ph
N
CH3
8a
7.48 7.46 7.37 7.36 7.31 7.30 7.29 7.27 7.26 7.26 7.24 7.23 7.23 7.22 7.21 7.21 7.20 7.19 7.18 7.04 7.02 7.01 6.92 6.90 6.90 6.84
3.83 3.82 3.80 3.79 3.77 3.75 3.73 3.73 3.52 3.51 3.49 3.47 3.38 3.36 3.35
2.64 2.62 2.61 2.61 2.60 2.46 2.43 2.42 2.40 2.40 2.39 2.38 2.37
1.93 1.91 1.89 1.87
1.96 1.18 12.29 1.03 1.91 0.91
4.96 1.00 1.01
1.19 4.05
0.98
8.0 7.5 7.0 6.5 6.0 5.5 5.0 4.5 4.0 3.5 3.0 2.5 2.0 1.5 1.0 0.5
f1 (ppm)

WYD-7-48-1

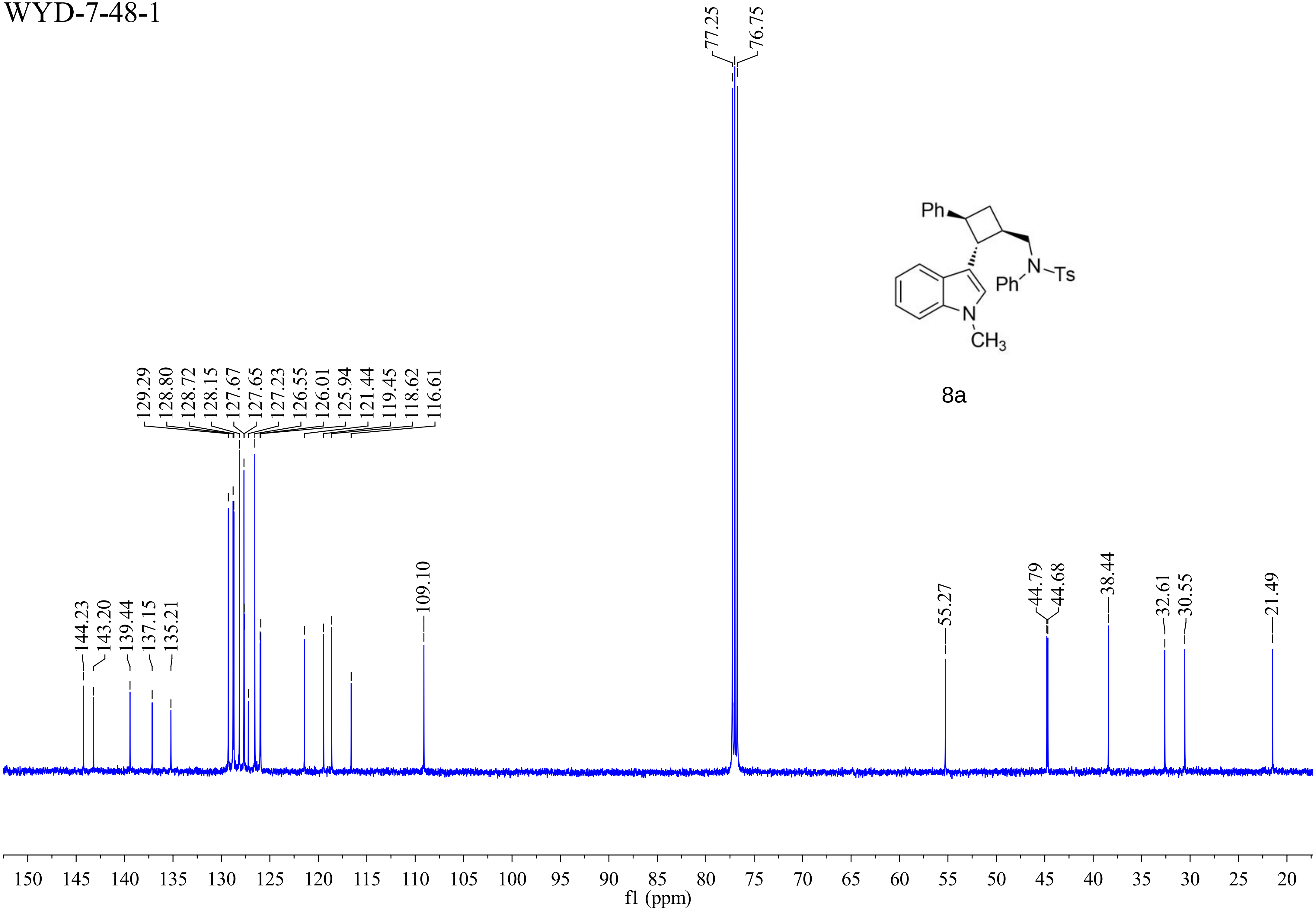

77.25
76.75
129.29
128.80
128.72
128.15
127.67
127.65
127.23
126.55
126.01
125.94
121.44
119.45
118.62
116.61
109.10
144.23
143.20
139.44
137.15
135.21
55.27
44.79
44.68
38.44
32.61
30.55
21.49
Ph
N
Ts
Ph
N
CH3
8a
150 145 140 135 130 125 120 115 110 105 100 95 90 85 80 75 70 65 60 55 50 45 40 35 30 25 20
f1 (ppm)

wyd-7-53 H

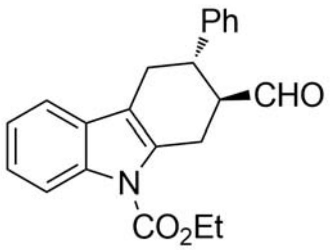
Ph
CHO
N
CO_2Et

5a

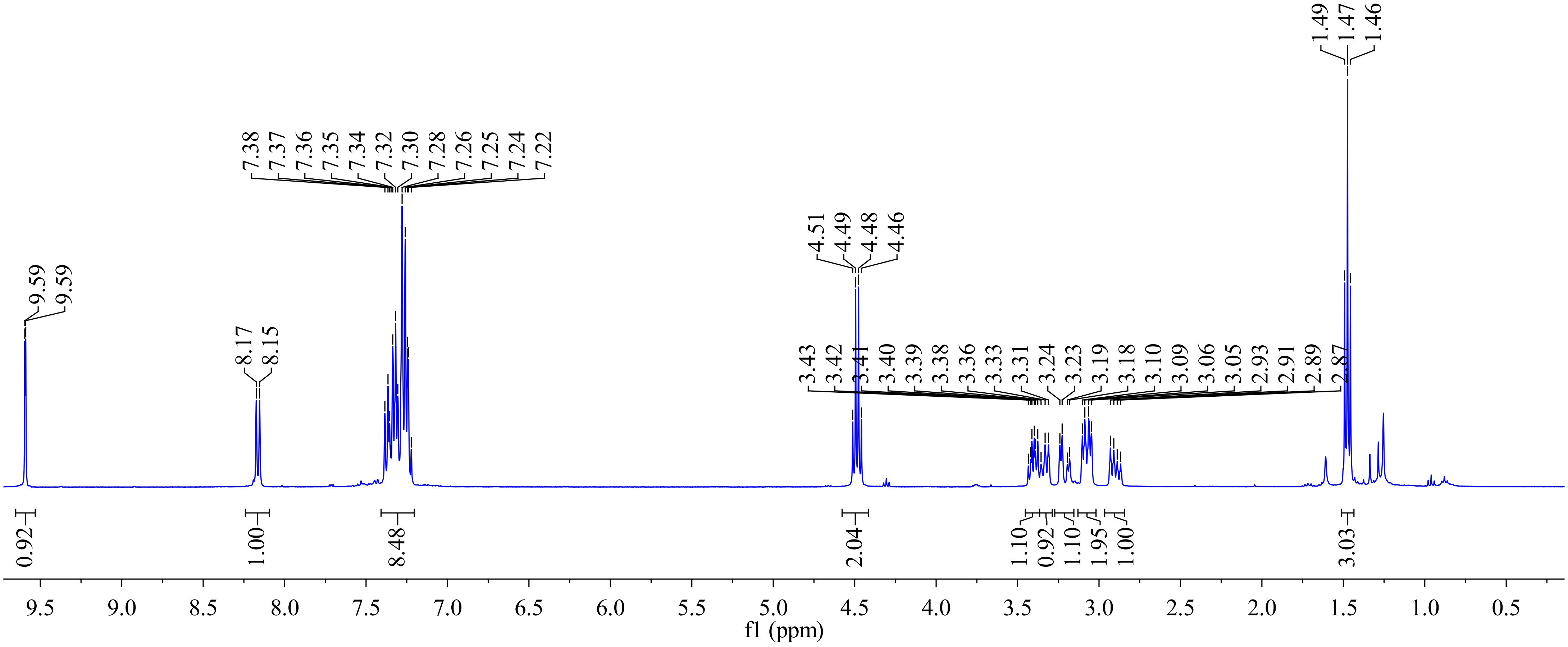
9.59
9.59
8.17
8.15
7.38
7.37
7.36
7.35
7.34
7.32
7.30
7.28
7.26
7.25
7.24
7.22
4.51
4.49
4.48
4.46
3.43
3.42
3.41
3.40
3.39
3.38
3.36
3.33
3.31
3.24
3.23
3.19
3.18
3.10
3.09
3.06
3.05
2.93
2.91
2.89
2.87
1.49
1.47
1.46
0.92
1.00
8.48
2.04
1.10
0.92
1.10
1.95
1.00
3.03
9.5
9.0
8.5
8.0
7.5
7.0
6.5
6.0
5.5
5.0
4.5
4.0
3.5
3.0
2.5
2.0
1.5
1.0
0.5
f1 (ppm)

wyd-7-53 C

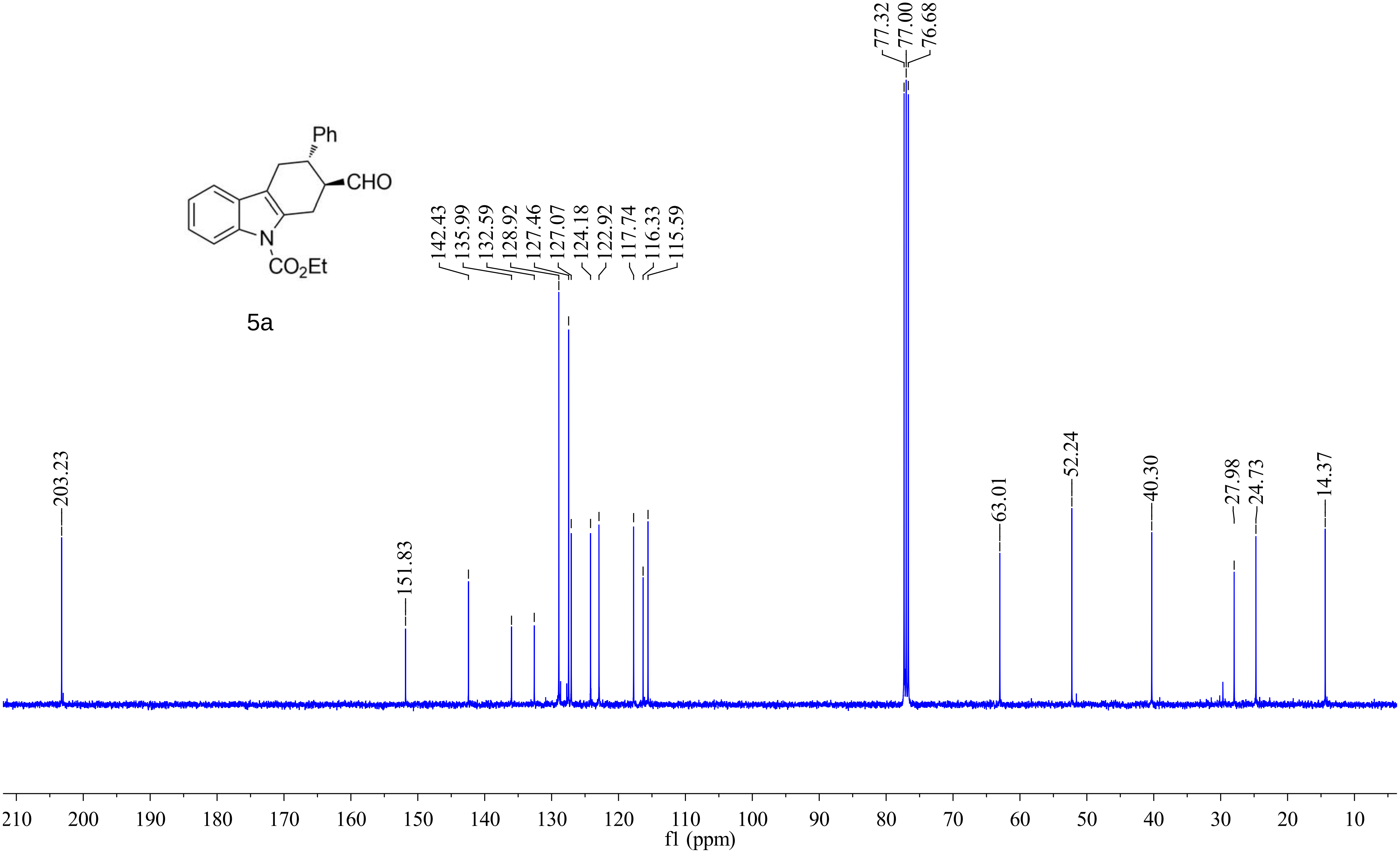
Ph
CHO
N
CO_2Et
5a
203.23
151.83
142.43
135.99
132.59
128.92
127.46
127.07
124.18
122.92
117.74
116.33
115.59
77.32
77.00
76.68
63.01
52.24
40.30
27.98
24.73
14.37
210 200 190 180 170 160 150 140 130 120 110 100 90 80 70 60 50 40 30 20 10
f1 (ppm)
