# Supplementary material for: Enantioselective gold-catalyzed intermolecular [2+2] versus [4+2]-cycloadditions of 3-styrylindoles with N-allenamides: observation of interesting substituent effects
Source: Chem Sci. 2015 Jun 23;6(10):5564–70. doi: 10.1039/c5sc01827g (PMC5949851; doi:10.1039/c5sc01827g)

Electronic Supplementary Material (ESI) for Chemical Science.
This journal is © The Royal Society of Chemistry 2015

SUPPORTING INFORMATION

Yidong Wang, Peichao Zhang, Yuan Liu, Fei Xia and Junliang Zhang*.*

Shanghai Key Laboratory of Green Chemistry and Chemical Processes, Department of Chemistry, East China Normal University, 3663 North Zhongshan Road, Shanghai 200062, P. R. China.

Fax:(+86)-021-6223-3213; E-mail : fxia@chem.ecnu.edu.cn, jlzhang@chem.ecnu.edu.cn

General Methods:

Unless otherwise noted, all reactions were carried out in standard Schlenk techniques with magnetic stirring bar under air. Materials obtained from commercial suppliers were used directly without further purification. ^{1}H NMR spectra were recorded on a BRUKER 400 (400 MHz) spectrometer or a Bruker 300 MHz spectrometer in $CDCl_3$. Chemical shifts are reported in ppm with tetramethylsilane (TMS: 0 ppm) with the solvent resonance as the internal standard. Data are reported as follows: chemical shift, multiplicity (s = singlet, d = doublet, t = triplet, q = quartet, m = multiplet), coupling constants (Hz), and integration. ^{13}C NMR spectra were recorded on a BRUKER 400 (100 MHz) spectrometer and a Bruker 300 (75.0 MHz) spectrometer in $CDCl_3$ with complete proton decoupling. Chemical shifts are reported in ppm with the deuterium solvent as the internal standard (e.g. $CDCl_3$: 77.0 ppm). The $[\alpha]_D$ was recorded using PolAAr 3005 High Accuracy Polarimeter. Infrared (IR) spectra were obtained using a Bruker tensor 27 infrared spectrometer. The ee was recorded using UltiMate 3000 HPLC from Dionex Company.

Anhydrous tetrahydrofuran (THF) was dried with sodium benzophenone and distilled before use; anhydrous toluene, 1,2-Dimethoxyethane (DME) and diethyl ether (Et_2O) was purified by distillation over Na prior to use; AgOTf, $AgSbF_6$, $AgNTf_2$, $AgBF_4$, and $AgPF_6$ were purchased from Alfa-Aesar Company and used directly .

Reactions were monitored by thin layer chromatography (TLC) using silicycle pre-coated silica gel plates. Flash column chromatography was performed on silica gel 60 (particle size 200-400 mesh ASTM, purchased from Yantai, China) and eluted with hexane/ethyl acetate or hexane/ CH_2Cl_2.

Complete optimization data of [2+2]-cycloaddition

Table S1. Examination of solvents.

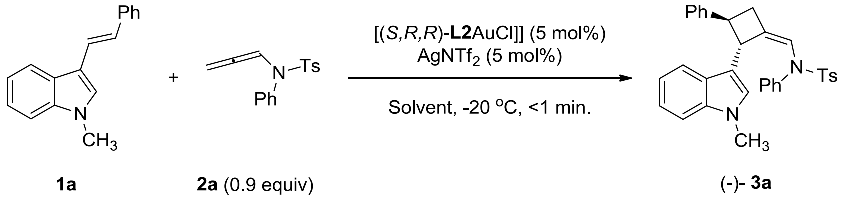

Entry	Solvent	Yield (%)[b]	ee (%)[c]
1	DCM	>95	90
2	DCE	>95	90
3	$CHCl_3$	>95	89
4	THF	>95	86
5	DCM (wet)	>95	90

[a]Reaction conditions: **1a** (0.2 mmol), **2a** (0.18 mmol), [(*S*,*R*,*R*)-L2AuCl] (0.01 mmol), $AgNTf_2$ (0.01 mmol) in dry solvent (4 mL) at -20 °C under Ar for less than 1 min. [b] NMR yield was determined by ^{1}H NMR using $C_2H_2Cl_4$ as internal standard. [c]ee was determined by chiral HPLC.

Table S2. Examination of silver salts.

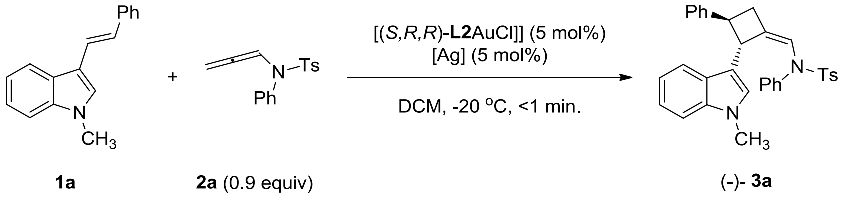

Entry	AgX	NMR Yield [b]	ee (%)
1	$AgNTf_2$	>95%	90%
2	AgOTf	95%	90%
3	$AgBF_4$	95%	90%
4	$AgPF_6$	95%	90%
5	AgOMs	92%	90%
6	AgOTs	93%	90%
7	No Ag	N.R.	-
8	$AgNTf_2$ (10 mol%)	>95%	90%

[a]Reaction conditions: **1a** (0.2 mmol), **2a** (0.18 mmol), [(*S*,*R*,*R*)-L2AuCl] (0.01 mmol), silver salt (0.01 mmol) in dry solvent (4 mL) at -20 °C under Ar for less than 1 min. [b] NMR yield was determined by ^{1}H NMR using $C_2H_2Cl_4$ as internal standard.

Table S3. Examination of catalyst loading and atmosphere. [a]

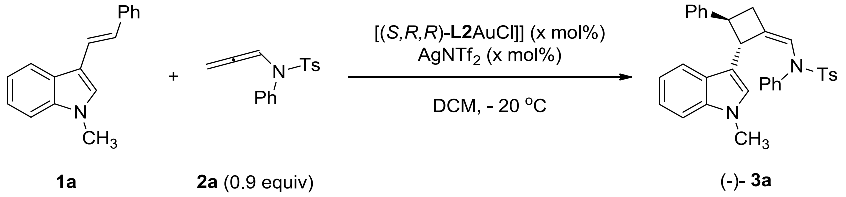

Entry	X	atmosphere	Yield (%)[b]	ee (%)
1	5	Ar	>95	90
2	5	air	>95	90
3	1	air	>95	90

[a]Reaction conditions: **1a** (0.2 mmol), **2a** (0.18 mmol), [(*S*,*R*,*R*)-L2AuCl] (0.01 mmol), $AgNTf_2$ (0.01 mmol) in dry solvent (4 mL) at -20 °C under Ar for less than 1 min. [b] NMR yield was determined by ^{1}H NMR using $C_2H_2Cl_4$ as internal standard.

Table S4. Examination of Temperature.

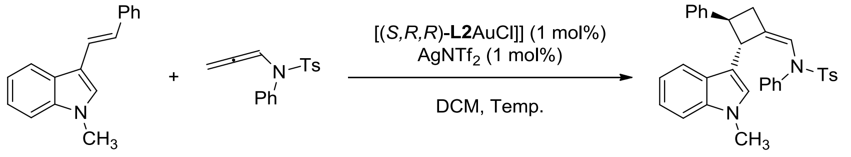

1a **2a** (0.9 equiv) (-)- **3a**

Entry	T (^{o}C)	Time (min.)	Yield (%)	ee (%)
1	-20	1	>95	90
2	-40	1	>95	93
3	-50	1	>95	95
4	-60	20	>95	96

Complete optimization data of [4+2]-cycloaddition

Table S5. Examination of Solvent.[a]

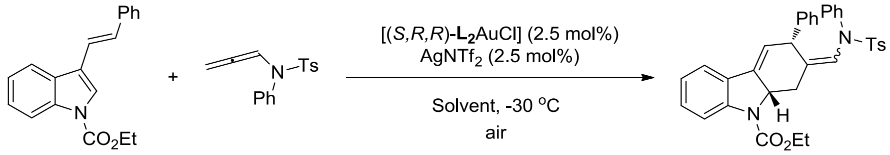

1e **2a** (0.9 equiv) **4a**

Entry	Solvent	*Z:E*[b]	ee (%) *Z:E*
1	DCE	5.3:1	95:92
2	DCM	4.3:1	94:93
3	$CHCl_3$	3.0:1	97:95
4[c]	THF	2.0:1	82:81
5[c]	dioxane	1.7:1	70:76
6[d]	acetone	5:1	95:88
7[c]	Et_2O	1.8:1	71:77
8[e]	toluene	-	-
9[e]	EA	-	-

[a] Unless otherwise specified, the reaction was run with **1e** (0.2 mmol), **2a** (0.18 mmol), [(*S*,*R*,*R*)-L2AuCl] (0.005 mmol), $AgNTf_2$ (0.005 mmol) in solvent (4 mL) for less than 1 min. The solvent here was used directly from chemical company, >95% NMR Yield was obtained. [b] The ratio of *Z:E* was determined by NMR. [c] at rt. [d] reacting for 1h. [e] no reaction.

Table S6. Examination of Ag salts.[a]

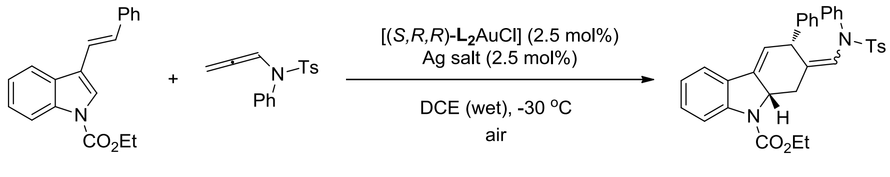

1e **2a** (0.9 equiv) **4a**

Entry	Ag salt	*Z:E*[b]	ee (%) *Z:E*
1	$AgNTf_2$	5.3:1	95:92
2	AgOTf	3.5:1	94:--[c]
3	$AgSbF_6$	3.8:1	94:-- [c]
4	$AgBF_4$	4.3:1	94:-- [c]
5[d]	AgOMs	-	-
6[d]	$C_3F_7O_2Ag$	-	-

[a] Unless otherwise specified, the reaction was run with **1e** (0.2 mmol), **2a** (0.18 mmol), [(*S*,*R*,*R*)-L2AuCl] (0.005 mmol), silver salt (0.005 mmol) in solvent (4 mL) for less than 1 min. >95% NMR Yield was obtained. [b] The ratio of Z:E was Determined determined by NMR. [c] the ee of the minor isomer was not determined. [d] no reaction.

Table S7. Examination of Temperature.[a]

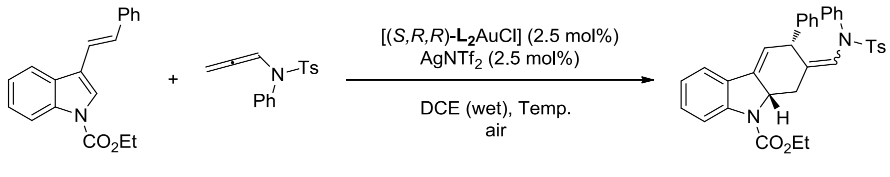

1e **2a** (0.9 equiv) **4a**

Entry	T (°C)	*Z:E*[b]	ee (%) *Z:E*
1	0	2.3:1	92:-- [c]
2	-20	3.9:1	94:--[c]
3	-30	5.3:1	95:92
4[d]	-30	4.3:1	94:-- [c]

[a] Unless otherwise specified, the reaction was run with **1e** (0.2 mmol), **2a** (0.18 mmol), [(*S*,*R*,*R*)-L2AuCl] (0.005 mmol), $AgNTf_2$ (0.005 mmol) in solvent (4 mL), >95% NMR Yield was obtained. [b] The ratio of *Z:E* was Determined determined by NMR. [c] the ee of the minor isomer was not determined. [d] 100 mg 4Å MS was added and the reaction was run in dry DCE under Ar atmosphere.

Figure S1. X-ray of 3f (CCDC 1036866) and (*Z*)-4a (CCDC 1036867).

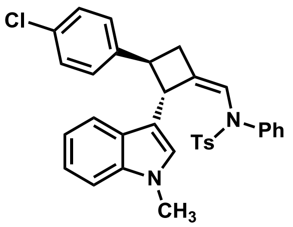

3f

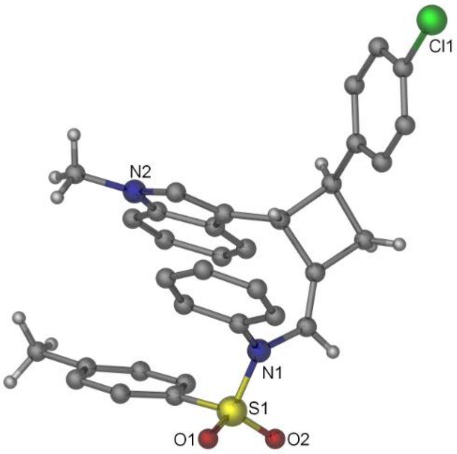

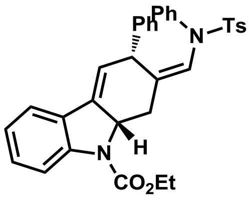

(*Z*)-4a

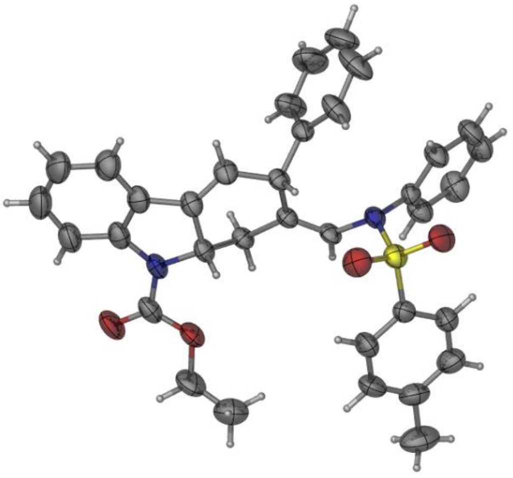

Preparation and Characterization of 3-vinylindoles

All 3-vinylindole substrates were synthesized according to our previous procedure.[1] The spectra of known compounds such as **1a**, **1b** are consistent with the literature, which are not included here except **1a** as a typical procedure.

Typical Procedure for synthesis of 3-styrylindoles.

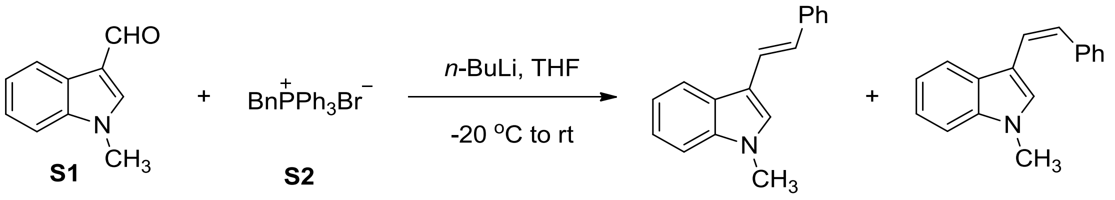

n-BuLi (2.5 M in hexane solution) (9.6 mL, 24 mmol) was slowly added to the suspension of $BnPPh_3Br$ (10.39 g, 24 mmol) in dry THF (130 mL) at -20 °C. The mixture was stirred at room temperature for 2 h followed by the addition of **S1** (3.18 g, 20 mmol) in THF (20 mL) at -20 °C. Then the mixture was stirred at room temperature for 2 hours, monitored by TLC and quenched by saturated solution of NH_4Cl at room temperature. The extracts with ethyl acetate were washed by Saturated salt water and dried over anhydrous sodium sulfate, then the solvent was removed under reduced pressure. The crude product was purified by column chromatography to give (*E*)-Product (2.70 g, 58%) as a white solid and (*Z*)-product (1.86 g, 40%) as a colorless liquid.

1. (*E*)-1-methyl-3-styryl-1H-indole. (1a)[1]

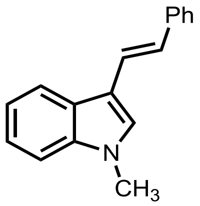

^{1}H NMR (400 MHz, CDCl3): δ 7.98 (d, *J* = 7.6 Hz, 1 H), 7.50 (d, *J* = 8.0 Hz, 2 H), 7.38-7.15 (m, 8 H), 7.08 (d, *J* = 16.4 Hz, 1 H), 3.74 (s, 3 H); **^{13}C NMR** (100 MHz, $CDCl_3$) δ 138.6, 137.6, 128.6, 128.5, 126.4, 126.1, 125.6, 124.7, 122.2, 121.6, 120.2, 120.0, 113.9, 109.5, 32.7.

2. (*E*)-1-methyl-3-(4-(trifluoromethyl)styryl)-1H-indole. (1k)

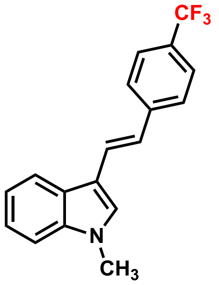

^{1}H NMR (400 MHz, $CDCl_3$): δ 7.98 (d, *J* = 8.0 Hz, 1 H), 7.55 (t, *J* = 9.6 Hz, 4 H), 7.39-7.19 (m, 5 H), 7.07 (d, *J* = 16.4 Hz, 1 H), 3.75 (s, 3H); **^{19}F NMR** (376 MHz, $CDCl_3$) δ -62.10; **^{13}C NMR** (100 MHz, $CDCl_3$): δ 142.2, 137.7, 129.5, 127.8 (q, *J* = 32 Hz), 125.9, 125.53, 125.47 (q, *J* = 3 Hz), 124.4 (q, *J* = 270 Hz), 124.2, 122.8, 122.5, 120.4, 120.2, 113.5, 109,7, 32.9. **MS** (EI): m/z (%): 301 (100); **HRMS** (EI) calculated for $C_{18}H_{14}NF_3$: 301.1078, found: 301.1079.

3. (*E*)-4-bromo-1-methyl-3-styryl-1H-indole. (1p)

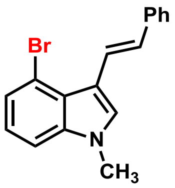

^{1}H NMR (400 MHz, $CDCl_3$): δ = 8.13 (d, *J* = 16.4 Hz, 1 H), 7.52 (d, *J* = 7.6 Hz, 2 H), 7.41 (s, 1 H), 7.38-7.31 (m, 3 H), 7.28-7.19 (m, 2 H), 7.06 (t, *J* = 8.0 Hz, 1 H), 6.81 (d, *J* = 16.4 Hz, 1 H), 3.80 (s, 3 H); **^{13}C NMR** (100 MHz, $CDCl_3$): δ 138.4, 138.3, 128.6, 126.8, 126.7, 126.0, 125.3, 125.1, 124.4, 122.6, 121.4, 115.4, 114.6, 108.9, 33.2. **MS** (EI): m/z (%): 311 (M^+, 100), 313 (M^++2, 99.8); **HRMS** (EI) calculated for $C_{17}H_{14}BrN$: 311.0310, found: 311.0306.

[1] H. Gao, X. Wu, J. Zhang, *Chem. Eur. J.* **2011**, *17*, 2838.
[2] N. P. Grimster, C. Gauntlett, C. R. A. Godfrey, M. J. Gaunt, *Angew. Chem. Int. Ed.* **2005**, *44*, 3125.

4. (*E*)-1-methyl-3-(2-(thiophen-2-yl)vinyl)-1H-indole. (1q)

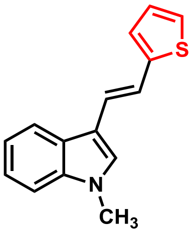

^{1}H NMR (400 MHz, $CDCl_3$) δ 7.94 (d, *J* = 7.6 Hz, 1 H), 7.34-7.27 (m, 2 H), 7.24-7.16 (m, 3 H), 7.15-7.08 (m, 2 H), 7.01-6.96 (m, 2 H), 3.76 (s, 3 H); **^{13}C NMR** (100 MHz, $CDCl_3$) δ 144.4, 137.7, 128.5, 127.5, 126.0, 123.9, 122.6, 122.3, 121.6, 120.2, 120.1, 118.1, 113.5, 109.6, 32.9; **MS** (EI): m/z (%): 239 (100); **HRMS** (EI) calculated for $C_{15}H_{13}NS$: 239.0769, found: 239.0771.

5. 1-methyl-3-vinyl-1H-indole. (1s)

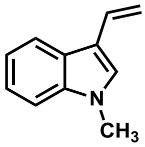

^{1}H NMR (400 MHz, $CDCl_3$) δ 7.87 (d, *J* = 7.6 Hz, 1H), 7.30-7.20 (m, 2H), 7.19-7.13 (m, 1H), 7.06 (s, 3H), 6.86 (q, *J* = 11.2 Hz, 1H), 5.66 (d, *J* = 17.6 Hz, 1H), 5.12 (d, *J* = 11.2 Hz, 1H), 3.69 (s, 3H); **^{13}C NMR** (100 MHz, $CDCl_3$) δ137.5, 129.3, 128.2, 126.1, 122.0, 120.1, 119.9, 114.1, 109.8, 109.4, 32.7.; **MS** (EI): m/z (%): 44 (100), 157 (M^+, 10.44); **HRMS** (EI) calculated for $C_{11}H_{11}N$: 157.0891, found: 157.0893.

6. 1-methyl-3-(pent-1-en-1-yl)-1H-indole. (1r)

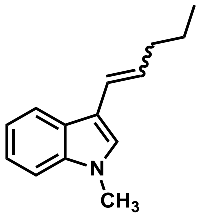

^{1}H NMR (400 MHz, $(CD_3)_2CO$) δ 7.81 (d, *J* = 8.0 Hz, 2H), 7.64 (d, *J* = 8.0 Hz, 2H), 7.40-7.30 (m, 3H), 7.23 (s, 1H), 7.22-7.15 (m, 2H), 7.08 (t, *J* = 7.6 Hz, 2H), 6.61 (d, *J* = 11.6 Hz, 1H), 6.55 (*J* = 16.0 Hz, 1H), 6.20-6.10 (m, 1H), 5.58-5.49 (m, 1H), 3.84 (s, 3H), 3.77 (s, 3H), 2.38-2.30 (m, 2H), 2.23-2.14 (m, 2H), 1.59-1.44 (m, 4H), 1.00-0.92 (m, 6H); **^{13}C NMR** (100 MHz, $(CD_3)_2CO$) δ 206.2, 138.6, 137.4, 129.0, 128.8, 128.5, 128.5, 127.2, 127.0, 123.7, 122.5, 122.4, 120.6, 120.4, 120.2, 120.0, 119.4, 114.6, 112.9, 110.4, 110.2, 36.5, 32.9, 32.8, 32.6, 23.8, 23.6, 14.3, 14.1.; **MS** (EI): m/z (%): 44 (100), 199 (M^+, 4.48); **HRMS** (EI) calculated for $C_{14}H_{17}N$: 199.1361, found: 199.1363.

7. (*E*)-1-benzyl-3-styryl-1H-indole. (1b)

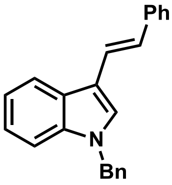

^{1}H NMR (400 MHz, $CDCl_3$) δ 8.03-7.97 (m, 1H), 7.50 (d, *J* = 7.2 Hz, 2H), 7.38-7.27 (m, 8H), 7.25-7.19 (m, 3H), 7.19-7.07 (m, 3H), 5.31 (s, 2H); **^{13}C NMR** (100 MHz, CDCl3) δ 138.6, 137.4, 137.0, 128.9, 128.6, 127.8, 127.74, 126.9, 126.54, 126.48, 125.8, 125.3, 122.5, 121.5, 120.4, 120.3, 114.7, 110.1, 50.2. **HRMS** (ESI) calculated for $C_{23}H_{19}NNa$ [M + Na^+]: 332.1410, found: 332.1418.

8. (*E*)-3-styryl-1H-indole. (1d) [2]

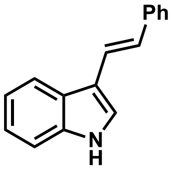

^{1}H NMR (400 MHz, $CDCl_3$) δ 8.16 (s, 1 H), 8.01 (d, *J* = 7.2 Hz, 1 H), 7.52 (d, *J* = 7.6 Hz, 2 H), 7.44-7.29 (m, 5 H), 7.29-7.18 (m, 3 H), 7.14 (d, *J* = 16.4 Hz, 1 H); **^{13}C NMR** (100 MHz, $CDCl_3$) δ 138.5, 136.8, 128.6, 126.6, 125.8, 125.62, 125.58, 123.7, 122.7, 121.6, 120.5, 120.2, 115.6, 111.4.

9. (*E*)-1-allyl-3-styryl-1H-indole. (1c)

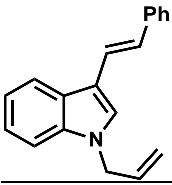

^{1}H NMR (400 MHz, $CDCl_3$) δ 7.99 (d, *J* = 7.6 Hz, 1 H), 7.51 (d, *J* = 7.6 Hz, 2 H), 7.42-7.16 (m, 8 H), 7.10 (d, *J* = 16.4 Hz, 1 H), 6.06-5.93 (m, 1 H), 5.22 (d, *J* = 10.0 Hz, 1 H), 5.12 (d, *J* = 17.2 Hz, 1 H), 4.71 (d, *J* = 5.2 Hz, 2 H); **^{13}C NMR** (100 MHz, $CDCl_3$) δ 138.6, 137.0, 133.0, 128.6, 127.4, 126.5, 126.3, 125.7, 125.0, 122.3, 121.5, 120.3, 120.1, 117.6, 114.3, 109.9, 48.8.

[2] N. P. Grimster, C. Gauntlett, C. R. A. Godfrey, M. J. Gaunt, *Angew. Chem. Int. Ed.* **2005**, *44*, 3125.

HRMS (ESI) calculated for $C_{19}H_{18}N$ [M + H^+]: 260.1434, found: 260.1403.

10. (*E*)-ethyl 3-styryl-1H-indole-1-carboxylate. (1e)

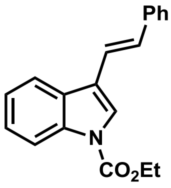

^{1}H NMR (400 MHz, $CDCl_3$) δ 8.23 (d, *J* = 7.6 Hz, 1 H), 7.90 (d, *J* = 7.2 Hz, 1 H), 7.77 (s, 1 H), 7.55-7.51 (m, 2 H), 7.42-7.31 (m, 4 H), 7.29-7.23 (m, 1 H), 7.21 (s, 2 H), 4.51 (q, *J* = 7.2 Hz, 2 H), 1.49 (t, *J* = 7.2 Hz, 3 H); **^{13}C NMR** (100 MHz, $CDCl_3$) δ 150.9, 137.6, 136.1, 129.2, 128.8, 128.7, 127.5, 126.2, 125.0, 123.4, 123.2, 120.0, 119.7, 119.6, 115.4, 63.3, 14.4; **MS** (EI): m/z (%): 291 (M^+, 63.99), 292 (M^++1, 13.19), 218 (100); **HRMS** (EI) calculated for [$C_{19}H_{17}NO_2$]: 291.1259, found: 291.1260.

9. (*E*)-ethyl 3-(4-chlorostyryl)-1H-indole-1-carboxylate . (1i')

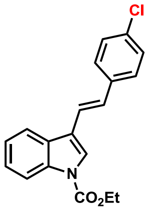

^{1}H NMR (400 MHz, $CDCl_3$) δ 8.23 (d, *J* = 7.6 Hz, 1 H), 7.89 (d, *J* = 7.6 Hz, 1 H), 7.78 (s, 1 H), 7.48-7.42 (m, 2 H), 7.42-7.31 (m, 4 H), 7.17 (q, *J* = 8.4 Hz, 2 H), 4.52 (q, *J* = 7.2 Hz, 2 H), 1.49 (t, *J* = 7.2 Hz, 3 H); **^{13}C NMR** (100 MHz, $CDCl_3$) δ 150.8, 136.1, 136.0, 132.9, 128.8, 128.6, 127.8, 127.3, 125.0, 123.7, 123.3, 120.4, 120.0, 119.3, 115.5, 63.4, 14.4; **MS** (EI): m/z (%): 325 (M^+, 62.74), 327 (M^++2, 21.82), 217 (100); **HRMS** (EI) calculated for $C_{19}H_{16}NO_2Cl$: 325.0870, found: 325.0869.

10. (*E*)-ethyl 3-(4-bromostyryl)-1H-indole-1-carboxylate. (1h')

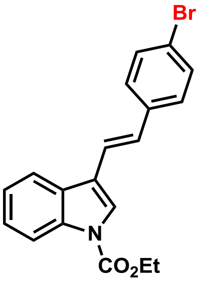

^{1}H NMR (400 MHz, $CDCl_3$) δ 8.23 (d, *J* = 7.6 Hz, 1 H), 7.88 (d, *J* = 7.6 Hz, 1 H), 7.78 (s, 1 H), 7.51-7.46 (m, 2 H), 7.42-7.31 (m, 4 H), 7.16 (q, *J* = 8.4 Hz, 2 H), 4.51 (q, *J* = 7.2 Hz, 2 H), 1.49 (t, *J* = 7.2 Hz, 3 H); **^{13}C NMR** (100 MHz, $CDCl_3$) δ 150.8, 136.5, 136.0, 131.7, 128.5, 127.8, 127.6, 125.0, 123.8, 123.3, 121.0, 120.5, 120.0, 119.3, 115.4, 63.4, 14.4; **MS** (EI): m/z (%): 369 (M^+, 35.16), 371 (M^++2, 34.10), 217 (100); **HRMS** (EI) calculated for $C_{19}H_{16}BrNO_2$: 363.0364, found: 363.0361.

11. (*E*)-ethyl 5-bromo-3-styryl-1H-indole-1-carboxylate. (1o')

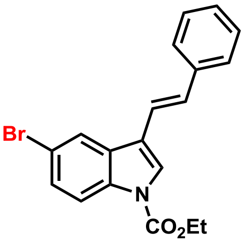

^{1}H NMR (400 MHz, $CDCl_3$) δ 8.06 (d, *J* = 8.4 Hz, 1 H), 7.96 (d, *J* = 1.6 Hz, 1 H), 7.71 (s, 1 H), 7.54-7.48 (m, 2 H), 7.44 (d, *J* = 8.8 Hz, 1 H), 7.49-7.33 (m, 2 H), 7.30-7.23 (m, 1 H), 7.10 (q, *J* = 8.8 Hz, 2 H), 4.48 (q, *J* = 7.2 Hz, 2 H), 1.47 (t, *J* = 7.2 Hz, 3 H); **^{13}C NMR** (100 MHz, $CDCl_3$) δ 150.4, 137.2, 134.6, 130.4, 129.5, 128.7, 127.7, 127.6, 126.2, 124.0, 122.7, 118.83, 118.76, 116.7, 116.6, 63.6, 14.4; **MS** (EI): m/z (%): 369 (M^+, 36.85), 371 (M^++2, 36.63), 217 (100); **HRMS** (EI) calculated for $C_{19}H_{16}NO_2Br$: 369.0364, found: 369.0360.

12. (*E*)-ethyl 3-(4-(trifluoromethyl)styryl)-1H-indole-1-carboxylate. (1k')

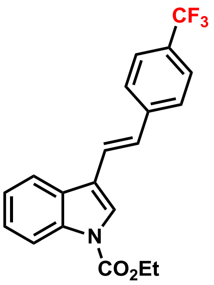

^{1}H NMR (400 MHz, $CDCl_3$) δ 8.23 (d, *J* = 7.6 Hz, 1 H), 7.90 (d, *J* = 7.2 Hz, 1 H), 7.81 (s, 1 H), 7.60 (t, *J* = 9.2 Hz, 4 H), 7.43-7.32 (m, 2 H), 7.32-7.17 (m, 2 H), 4.51 (q, *J* = 7.2 Hz, 2 H), 1.49 (t, *J* = 7.2 Hz, 3 H); **^{13}C NMR** (100 MHz, $CDCl_3$) δ 150.7, 141.1, 136.1, 129.0 (q, *J* = 32 Hz), 128.5, 127.4, 126.2, 125.6 (q, *J* = 4 Hz) , 125.1, 124.4, 124.2 (q, J = 270 Hz), 123.4, 122.3, 120.0, 119.1, 115.5, 63.5, 14.4; **^{19}F NMR** (376 MHz, $CDCl_3$) δ -62.39; **MS** (EI): m/z (%): 359 (M^+, 100); **HRMS** (EI) calculated for $C_{20}H_{16}NO_2F_3$: 359.1133, found: 359.1132.

13. (*E*)-ethyl 3-(4-methylstyryl)-1H-indole-1-carboxylate. (1j')

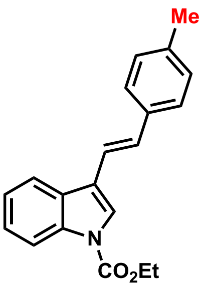

^{1}H NMR (400 MHz, $CDCl_3$) δ 8.23 (d, *J* = 7.2 Hz, 1 H), 7.91 (d, *J* = 7.2 Hz, 1 H), 7.77 (s, 1 H), 7.46-7.42 (m, 2 H), 7.42-7.31 (m, 2 H), 7.23-7.14 (m, 4 H), 4.51 (q, *J* = 7.2 Hz, 2 H), 2.38 (s, 3 H), 1.49 (t, *J* = 7.2 Hz, 3 H); **^{13}C NMR** (100 MHz, $CDCl_3$) δ 150.8, 137.3, 136.0, 134.8, 129.4, 129.1, 128.8, 126.0, 124.9, 123.2, 123.1, 120.0, 119.7, 118.6, 115.4, 63.3, 21.2, 14.4; **MS** (EI): m/z (%): 305 (M^+, 89.67), 306 (M^++1, 19.63), 217 (100); **HRMS** (EI) calculated for$C_{20}H_{19}NO_2$: 305.1416, found: 305.1414.

14. (*E*)-ethyl 3-(4-methoxystyryl)-1H-indole-1-carboxylate. (1l')

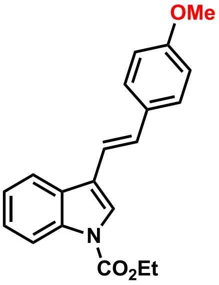

^{1}H NMR (400 MHz, $CDCl_3$) δ 8.23 (d, *J* = 7.2 Hz, 1 H); 7.89 (d, *J* = 7.6 Hz, 1 H), 7.75 (s, 1 H), 7.50-7.44 (m, 2 H), 7.41-7.30 (m, 2 H), 7.12 (q, *J* = 16.4 Hz, 2 H), 6.95-6.89 (m, 2 H), 4.51 (q, *J* = 7.2 Hz, 2 H), 3.84 (s, 3 H), 1.49 (t, *J* = 7.2 Hz, 3 H); **^{13}C NMR** (100 MHz, $CDCl_3$) δ 159.2, 150.9, 136.0, 130.4, 128.9, 128.8, 127.3, 124.9, 123.1, 122.8, 120.0, 119.9, 117.5, 115.4, 114.1, 63.3, 55.3, 14.4; **MS** (EI): m/z (%): 321 (M^+, 100), **HRMS** (EI) calculated for $C_{20}H_{19}NO_3$: 321.1365, found: 321.1367.

15. (*E*)-ethyl 5-methoxy-3-styryl-1H-indole-1-carboxylate. (1m')

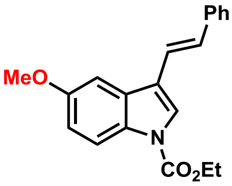

^{1}H NMR (400 MHz, $CDCl_3$) δ 8.08 (d, *J* = 7.6 Hz, 1 H), 7.73 (s, 1 H), 7.50 (d, *J* = 7.6 Hz, 2 H), 7.35 (t, *J* = 7.6 Hz, 2 H), 7.32-7.28 (m, 1 H), 7.25-7.21 (m, 1 H), 7.14 (q, *J* = 16.8 Hz, 2 H), 6.97 (d, *J* = 9.6 Hz, 1 H), 4.47 (q, *J* = 7.2 Hz, 2 H), 3.89 (s, 3 H), 1.46 (t, *J* = 7.2 Hz, 3 H); **^{13}C NMR** (100 MHz, $CDCl_3$) δ 156.3, 150.8, 137.6, 130.7, 129.7, 129.0, 128.7, 127.4, 126.2, 123.9, 119.6, 119.4, 116.1, 113.1, 103.2, 63.2, 55.8, 14.4; **MS** (EI): m/z (%): 321 (100); **HRMS** (EI) calculated for $C_{20}H_{19}NO_3$: 321.1365, found: 321.1362.

16. (*E*)-ethyl 5-methyl-3-styryl-1H-indole-1-carboxylate. (1n')

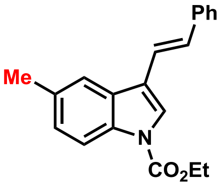

^{1}H NMR (400 MHz, $CDCl_3$) δ 8.08 (d, *J* = 6.8 Hz, 1H), 7.73 (s, 1H), 7.67 (s, 1H), 7.53 (d, *J* = 7.2 Hz, 2H), 7.37 (t, *J* = 7.6 Hz, 2 H), 7.29-7.22 (m, 1 H), 7.22-7.15 (m, 3 H), 4.48 (q, *J* = 7.2 Hz, 2 H), 2.50 (s, 3 H), 1.47 (t, *J* = 7.2 Hz, 3 H); **^{13}C NMR** (100 MHz, $CDCl_3$) δ 150.8, 137.7, 134.2, 132.7, 128.9, 128.7, 127.4, 126.2, 126.1, 123.5, 120.0, 119.8, 119.4, 115.0, 104.0 , 63.2, 21.5, 14.4. **HRMS** (ESI) calculated for $C_{20}H_{19}NNaO_2$ [M + Na^+]: 328.1308, found: 328.1299.

17. (*E*)-ethyl 4-bromo-3-styryl-1H-indole-1-carboxylate. (1p')

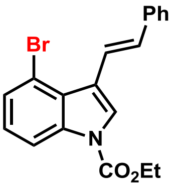

^{1}H NMR (400 MHz, $CDCl_3$) δ 8.20 (d, *J* = 8.0 Hz, 1H), 7.91 (d, *J* = 16.0 Hz, 1H), 7.83 (s, 1H), 7.50 (d, *J* = 7.6 Hz, 2H), 7.42 (d, *J* = 7.2 Hz, 1H), 7.35 (t, *J* = 7.6 Hz, 2H), 7.25-7.21 (m, 1H), 7.15 (t, *J* = 8.0 Hz, 1H), 6.90 (d, *J* = 16.0 Hz, 1H), 4.49 (q, *J* = 7.2 Hz, 2H), 1.47 (t, *J* = 7.2 Hz, 3H); **^{13}C NMR** (100 MHz, $CDCl_3$) δ 150.4, 137.5, 137.0, 129.2, 128.7, 127.8, 127.7, 127.5, 126.4, 125.5, 122.5, 121.1, 120.2, 114.6, 114.5, 63.7, 14.4; **MS** (EI): m/z (%): 369 (M^+, 32.27), 371 (M^++2, 31.97), 217 (100); **HRMS** (EI) calculated for $C_{19}H_{16}NO_2Br$: 369.0364, found: 369.0367.

18. (*E*)-ethyl 3-(2-(thiophen-2-yl)vinyl)-1H-indole-1-carboxylate. (1q')

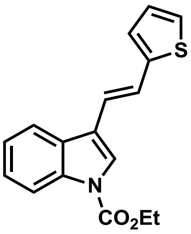

^{1}H NMR (400 MHz, $CDCl_3$) δ 8.24 (d, *J* = 7.2 Hz, 1H), 7.87 (d, *J* = 7.8 Hz, 1H), 7.75 (s, 1

H), 7.45-7.30 (m, 3H), 7.20 (d, *J* = 4.8 Hz, 1H), 7.10-7.00 (m, 3H), 4.51 (q, *J* = 7.2 Hz, 2H), 1.49 (t, *J* = 7.2 Hz, 3H); **^{13}C NMR** (100 MHz, $CDCl_3$) δ 150.7, 143.2, 136.0, 128.5, 127.6, 125.5, 125.0, 124.0, 123.4, 123.2, 122.3, 120.0, 119.4, 119.2, 115.4, 63.3, 14.4; **MS** (EI): m/z (%): 297 (100); **HRMS** (EI) calculated for $C_{17}H_{15}NO_2S$: 297.0824, found: 297.0827.

19. ethyl 3-vinyl-1H-indole-1-carboxylate. (1s')

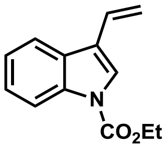

^{1}H NMR (400 MHz, $CDCl_3$) δ 8.20 (d, *J* = 8.0 Hz, 1H), 7.80 (d, *J* = 8.0 Hz, 1H), 7.66 (s, 1H), 7.40-7.27 (m, 2H), 6.81 (q, *J* = 11.2 Hz, 1H), 5.82 (d, *J* = 17.6 Hz, 1H), 5.34 (d, *J* = 11.2 Hz, 1H), 4.49 (q, *J* = 7.2 Hz, 2H), 1.47 (t, *J* = 7.2 Hz, 3H); **^{13}C NMR** (100 MHz, $CDCl_3$) δ 150.8, 135.9, 128.6, 127.9, 124.7, 123.5, 123.1, 119.9, 119.7, 115.2, 114.6, 63.2, 14.3.; **MS** (EI): m/z (%): 143 (100), 215 (M^+, 89.80); **HRMS** (EI) calculated for $C_{13}H_{13}NO_2$: 215.0946, found: 215.0944.

20. (*E*)-ethyl 3-(pent-1-en-1-yl)-1H-indole-1-carboxylate. (1r')

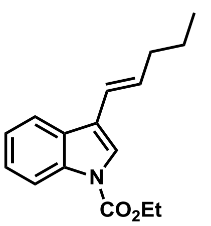

^{1}H NMR (400 MHz, $CDCl_3$) δ 8.17 (s, 1H), 7.57 (d, *J* = 7.2 Hz, 2H), 7.34 (t, *J* = 7.2 Hz, 1H), 7.26 (t, *J* = 7.6 Hz, 1H), 6.47 (d, *J* = 11.2 Hz, 1H), 5.85-5.76 (m, 1H), 4.49 (q, *J* = 7.2 Hz, 2H), 2.37-2.28 (m, 2H), 1.58-1.49 (m, 2H), 1.47 (t, *J* = 7.2 Hz, 3H), 0.97 (t, *J* = 7.6 Hz, 3H); **^{13}C NMR** (100 MHz, $CDCl_3$) δ 151.1, 134.8, 134.0, 131.9, 130.6, 124.6, 124.6, 122.9, 122.8, 120.6, 119.9, 119.8, 119.1, 118.0, 117.9, 115.2, 115.1, 63.2, 35.6, 31.7, 30.7, 22.7, 22.6, 19.2, 14.4, 13.8, 13.7, 13.7.; **MS** (EI): m/z (%): 257 (M^+, 13.84), 183 (100); **HRMS** (EI) calculated for $C_{16}H_{19}NO_2$: 257.1416, found: 257.1414.

21. (*E*)-3-styryl-1-tosyl-1H-indole (1f)

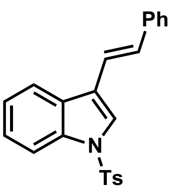

^{1}H NMR (400 MHz, $CDCl_3$) δ 8.02 (d, *J* = 8.4 Hz, 1H), 7.83 (d, *J* = 7.2 Hz, 1H), 7.78 (d, *J* = 8.4 Hz, 2H), 7.72 (s, 1H), 7.50 (d, *J* = 7.2 Hz, 2H), 7.40-7.22 (m, 5H), 7.21-7.13(m, 4H), 2.29 (s, 3H); **^{13}C NMR** (100 MHz, $CDCl_3$) δ 145.0, 137.3, 135.5, 134.9, 129.9, 129.7, 129.0, 128.7, 127.6, 126.8, 126.2, 125.0, 123.9, 123.5, 120.7, 120.4, 119.1, 113.8, 21.5.; **MS** (EI): m/z (%): 373 (M^+, 32.57), 374 (M^++1, 9.22), 218 (100); **HRMS** (EI) calculated for $C_{23}H_{19}NO_2S$: 373.1137, found: 373.1140.

22. (*E*)-1-(3-styryl-1H-indol-1-yl)ethanone. (1g)

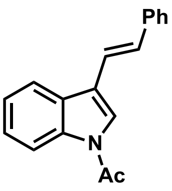

^{1}H NMR (400 MHz, $CDCl_3$) δ 8.48 (d, *J* = 7.6 Hz, 1H), 7.91 (d, *J* = 16.0 Hz, 1H), 7.83 (s, 1H), 7.50 (d, *J* = 7.6 Hz, 2H), 7.42 (d, *J* = 7.2 Hz, 1H), 7.35 (t, *J* = 7.6 Hz, 2H), 7.25-7.21 (m, 1H), 7.15 (t, *J* = 8.0 Hz, 1H), 6.90 (d, *J* = 16.0 Hz, 1H), 4.49 (q, *J* = 7.2 Hz, 2H), 1.47 (t, *J* = 7.2 Hz, 3H); **^{13}C NMR** (100 MHz, $CDCl_3$) δ168.3, 137.4, 136.4, 129.6, 128.7, 127.6, 126.2, 125.6, 123.9, 122.9, 120.6, 119.8, 119.4, 116.8, 24.0; **HRMS** (ESI) calculated for $C_{18}H_{15}NNaO$ [M + Na^+]: 284.1046, found: 284.1038.

Preparation and Characterization of *N*-allenamides.

All *N*-allenamides in Scheme 1 were synthesized according to the procedure.[3]

23. 4-methyl-*N*-phenyl-*N*-(propa-1,2-dien-1-yl)benzenesulfonamide. (2a)

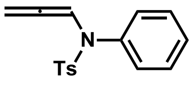

^{1}H NMR (400 MHz, $CDCl_3$) δ 7.55 (d, *J* = 8.0 Hz, 2H), 7.32-7.25 (m, 5H), 7.11 (t, *J* = 6.4 Hz, 1H), 7.02-6.98 (m, 2H), 5.02 (d, *J* = 6.4 Hz, 2H), 2.44 (s, 3H); **^{13}C NMR** (100 MHz, $CDCl_3$) δ 201.0, 143.9, 137.1, 135.2, 129.6, 129.5, 128.7, 128.6, 127.7, 102.4, 87.5,

[3] S. Suárez-Pantiga, C. Hernández-Díaz, E. Rubio, J. M. González, *Angew. Chem., Int. Ed.*, **2012**, *51*, 11552.

21.6; **MS** (EI): m/z (%): 285 (M^+, 1.62), 139 (100); **HRMS** (EI) calculated for [$C_{16}H_{15}NO_2S$]: 285.0824, found: 285.0827.

24. *N*-(4-bromophenyl)-4-methyl-*N*-(propa-1,2-dien-1-yl)benzenesulfonamide. (2c)

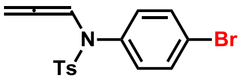

^{1}H NMR (400 MHz, $CDCl_3$) δ 7.54 (d, *J* = 8.0 Hz, 2H), 7.43-7.38 (m, 2H), 7.28 (d, *J* = 8.4 Hz, 2H), 7.08 (t, *J* = 5.4 Hz, 1H), 6.95-6.85 (m, 2H), 5.05 (d, *J* = 5.4 Hz, 2H), 2.44 (s, 3H); **^{13}C NMR** (100 MHz, $CDCl_3$) δ 200.9, 144.2, 136.2, 134.9, 132.0, 131.2, 129.6, 127.7, 122.7, 102.2, 87.9, 21.6; **MS** (EI): m/z (%): 363 (M^+, 0.58), 365 (M^++2, 0.61), 139 (100); **HRMS** (EI) calculated for $C_{16}H_{14}NO_2SBr$: 362.9929, found: 362.9933.

25. *N*-(4-methoxyphenyl)-4-methyl-N-(propa-1,2-dien-1-yl)benzenesulfonamide. (2b)

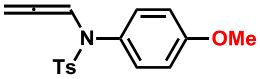

^{1}H NMR (400 MHz, $CDCl_3$) δ 7.55 (d, *J* = 8.4 Hz, 2 H), 7.26 (d, *J* = 8.0 Hz, 2 H), 7.12 (t, *J* = 6.4 Hz, 1 H), 6.92-6.86 (m, 2 H), 6.81-6.75 (m, 2 H), 5.02 (d, *J* = 6.0 Hz, 2 H), 3.78 (s, 3 H), 2.43 (s, 3 H); **^{13}C NMR** (100 MHz, $CDCl_3$) δ 200.9, 159.5, 143.8, 135.2, 130.7, 129.51, 129.46, 127.7, 113.8, 102.7, 87.4, 55.3, 21.6; **MS** (EI): m/z (%): 315 (M^+, 6.43), 176 (100); **HRMS** (EI) calculated for $C_{17}H_{17}NO_3$: 315.0929, found: 315.0930.

26. 3-(propa-1,2-dien-1-yl)oxazolidin-2-one.

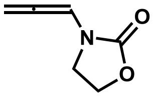

^{1}H NMR (400 MHz, $CDCl_3$) δ 6.90-6.83 (m, 1 H), 5.43 (d, *J* = 6.4 Hz, 2 H), 4.41 (t, *J* = 8.0 Hz, 2 H), 3.60 (t, *J* = 8.0 Hz, 2 H); **^{13}C NMR** (100 MHz, $CDCl_3$) δ 201.4, 155.2, 96.9, 87.8, 62.2, 43.1.

General Procedure A for asymmetric gold-catalyzed [2+2]-cycloaddition.

The solution of (*S*,*R*,*R*)-**L2**AuCl (1.0 mol %) and $AgNTf_2$ (1 mol %) in DCM (1 mL) was stirred at rt for 15 min., Then the above catalyst solution then was added to the solution of **1** (0.2 mmol) and **2** (0.18 mmol) in DCM (3 mL) at -60 °C. The reaction was determined by TLC, after the less component was consumed, the solution was removed under reduced pressure. The diastereomeric ratio was determined by crude ^{1}H NMR, the resulting crude mixture was purified by flash column chromatography on silica gel with petroleum ether/ ethyl acetate (20:1) as the solvent to afford product. The enantiomeric excesses of the products were determined by chiral stationary phase HPLC using a Chiralpak AD-H, AS-H, OD-H.

General Procedure B for asymmetric gold-catalyzed [4+2] cycloaddition

The solution of (*S*,*R*,*R*)-**L2**AuCl (2.5 mol %) and $AgNTf_2$ (2.5 mol %) in DCE (1 mL) was stirred at rt for 15 min., Then the above catalyst solution then was added to the solution of **1** (0.2 mmol) and **2** (0.18 mmol) in DCE (3 mL) at -30 °C. The reaction was determined by TLC, after the less component was consumed, the solution was removed under reduced pressure. The diastereomeric ratio was determined by crude ^{1}H NMR, the resulting crude mixture was purified by flash column chromatography on silica gel with petroleum ether/ DCM (1:1) as the solvent to to afford product. The enantiomeric excesses of the products were determined by chiral stationary phase HPLC using a Chiralpak AD-H, AS-H, OD-H, OD-3.

Characterization Data for New Compounds:

1. 4-methyl-*N*-((*Z*)-((2*R*,3*S*)-2-(1-methyl-1H-indol-3-yl)-3-phenylcyclobutylidene)methyl)-*N*-phenylbenzenesulfonamide (3a) .

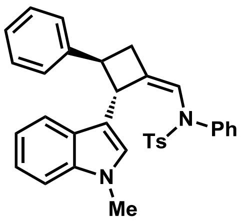

Isolated in 99% yield as white solid.

^{1}H NMR (400 MHz, $CDCl_3$) δ 7.39 (d, *J* = 8.4 Hz, 2 H), 7.25-7.11 (m, 10 H),

6.95-6.91 (m, 2 H), 6.81 (t, J = 15.6 Hz, 2 H), 6.57 (q, J = 6.4 Hz, 1 H), 6.55 (s, 1 H), 6.45-6.40 (m, 2 H), 3.69 (s, 3 H), 3.61-3.57 (m, 1 H), 3.55-3.50 (m, 1 H), 3.28-3.21 (m, 1 H), 2.89-2.83 (m, 1 H), 2.39 (s, 3 H); **^{13}C NMR** (100 MHz, $CDCl_3$) δ 144.7, 143.5, 139.0, 137.0, 135.3, 129.9, 129.3, 128.22, 128.19, 127.8, 127.5, 126.8, 126.5, 126.40, 126.37, 126.0, 121.1, 120.4, 119.6, 118.5, 114.7, 108.8, 47.9, 45.6, 33.6, 32.5, 21.5; **MS** (EI): m/z (%): 363 (100); **HRMS** (EI) calculated for [$C_{33}H_{30}N_2O_2S$ - $C_7H_7O_2S$]: 363.1861, found: 363.1859. **$[\alpha]_D^{20}$** = -89.4 (c = 0.5, $CHCl_3$), **HPLC conditions**: Daicel Chiralpak AS-H column (90:10 hexane: 2-propanol, 0.8 mL/min, 224 nm); tr (minor) = 12.29 min, tr (major) = 14.71 min, 96% ee.

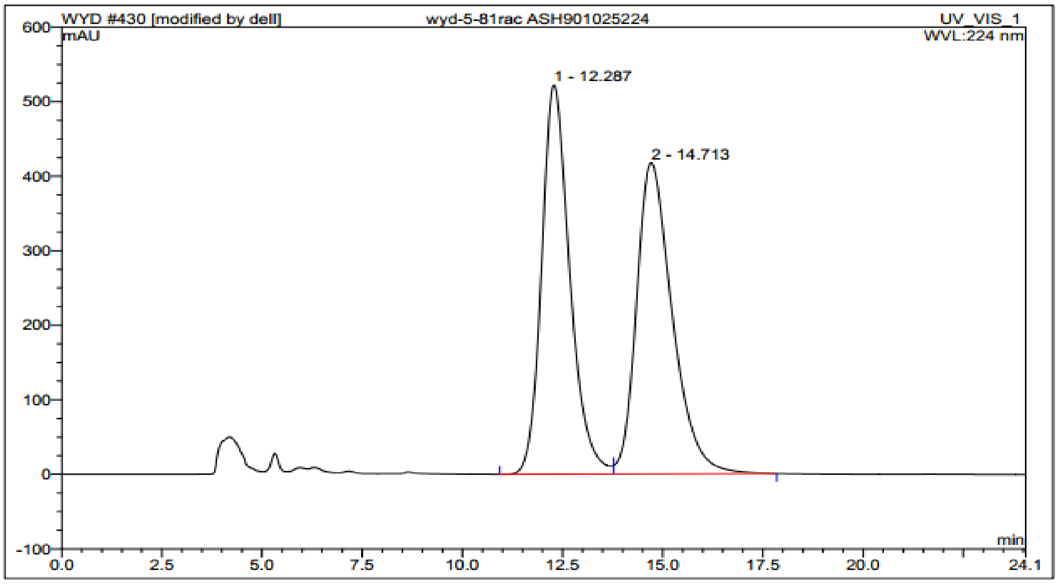

Peak #	Time(min)	Height (mAU)	Area (mAU*min)	Area(%)
1	12.09	522.256	426.471	49.72
2	14.71	417.344	431.333	50.28

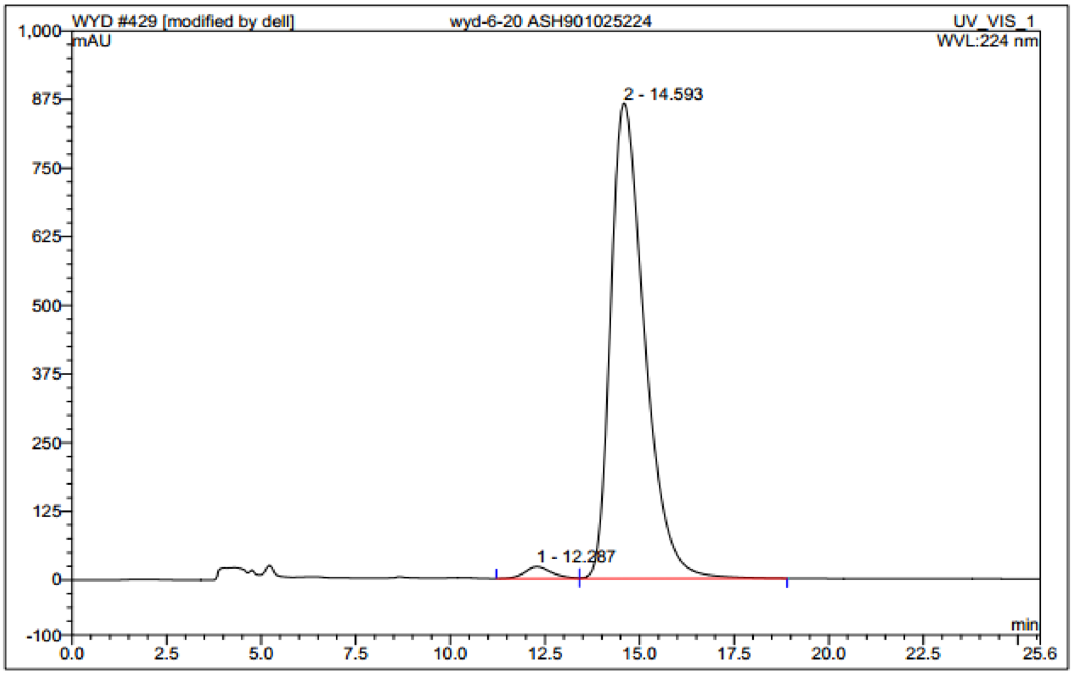

Peak #	Time(min)	Height (mAU)	Area (mAU*min)	Area (%)
1	12.29	21.705	17.719	1.93
2	14.59	865.428	899.417	98.07

2. ***N*-((*Z*)-((2*R*,3*S*)-2-(1-benzyl-1H-indol-3-yl)-3-phenylcyclobutylidene)methyl)-4-methyl-N-phenylbenzene sulfonamide (3b) .**

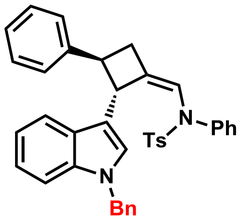

Isolated in 94% yield as white solid.

^{1}H NMR (400 MHz, $CDCl_3$) δ 7.40-7.09 (m, 18 H), 6.95-6.88 (m, 2 H), 6.78-6.73 (m, 3H), 6.58-6.54 (m, 1 H), 6.48-6.43 (m, 2 H), 5.22 (s, 1 H), 3.60-3.52 (m, 2 H), 3.30-3.24 (m, 1 H), 2.88-2.82 (m, 1 H), 2.37 (s, 3 H); **^{13}C NMR** (100 MHz, $CDCl_3$) 144.8, 143.5, 139.1, 137.7, 136.7, 135.4, 130.4, 129.3, 128.7, 128.3, 128.2, 127.9, 127.6, 127.5, 127.0, 126.6, 126.4, 126.1, 125.9, 121.4, 120.8, 119.7, 118.8, 115.7, 109.4, 50.0, 47.8, 45.5, 33.7, 21.5; **HRMS** (ESI): m/z calculated for $C_{39}H_{34}N_2NaO_2S$ [M + Na^+]: 671.2233, found: 617.2228; **$[\alpha]_D^{20}$** = -79.2 (c = 0.5, $CHCl_3$), **HPLC conditions**: with a Chiralpak AD-H column (95: 05 hexane: 2-propanol, 0.8 mL/min, 224 nm); tr (minor) = 17.03 min, tr (major) = 19.64 min, 92% ee.

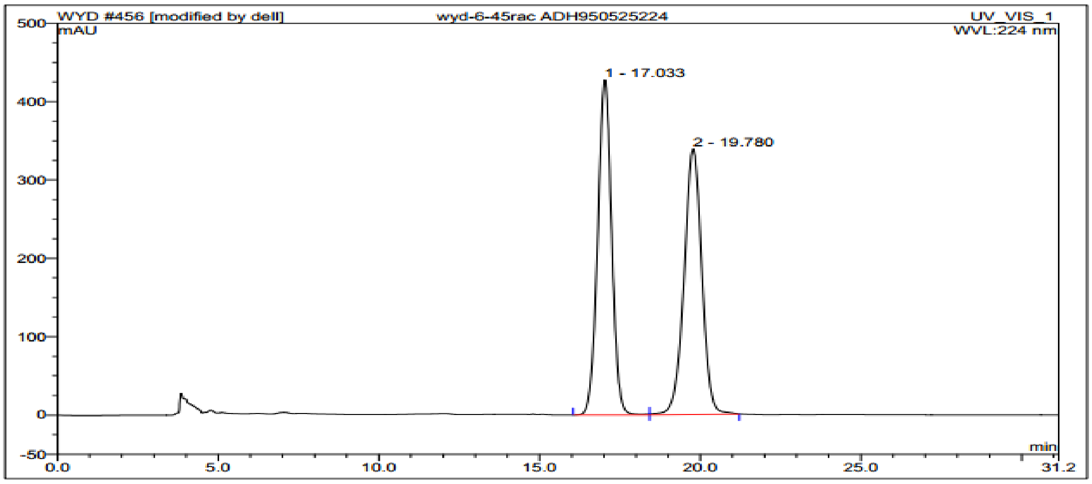

Peak #	Time (min)	Height (mAU)	Area (mAU*min)	Area (%)
1	17.03	427.627	215.851	49.84
2	19.78	338.550	217.239	50.16

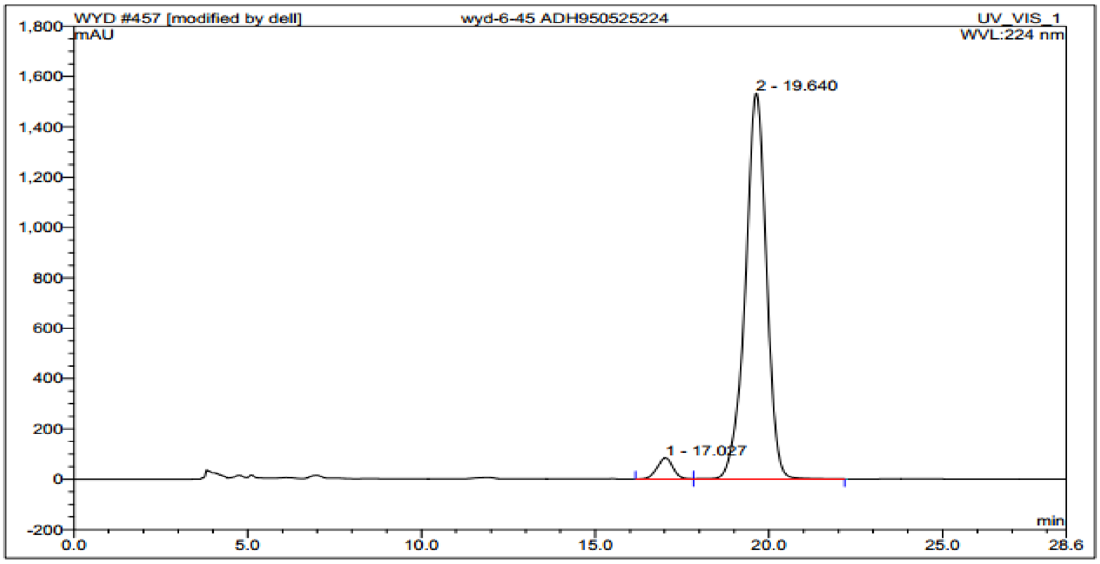

Peak #	Time(min)	Height (mAU)	Area (mAU*min)	Area(%)
1	17.03	83.504	43.989	3.90
2	19.64	1532.385	1082.558	96.10

3. ***N*-((*Z*)-((2*R*,3*S*)-2-(1-allyl-1H-indol-3-yl)-3-phenylcyclobutylidene)methyl)-4-methyl-*N*-phenylbenzenesulfonamide (3c) .**

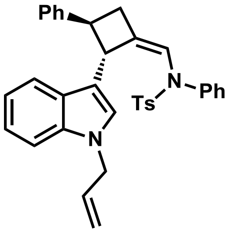

Isolated in 93% yield as colorless liquid.

^{1}H NMR (400 MHz, $CDCl_3$) δ 7.45 (d, *J* = 8.0 Hz, 2 H), 7.34-7.26 (m, 4 H), 7.26-7.17 (m, 6 H), 7.04-6.97 (m, 2 H), 6.94-6.87 (m, 2 H), 6.74 (s, 1 H), 6.40 (q, *J* = 2.0 Hz, 1 H), 6.57-6.51 (m, 2 H), 6.13-6.01 (m, 1 H), 5.30 (d, *J* = 10.0 Hz, 1 H), 5.23 (d, *J* = 17.2 Hz, 1 H), 4.70 (d, *J* = 5.6 Hz, 2 H), 3.70-3.65 (m, 1 H), 3.61 (q, *J* = 7.6 Hz, 1 H), 3.38-3.28 (m, 1 H), 2.97-2.88 (m, 1 H), 2.44 (s, 3 H); **^{13}C NMR** (100 MHz, $CDCl_3$) 144.7, 143.4, 139.0, 136.3, 135.2, 133.6, 130.2, 129.3, 128.2, 128.1, 127.8, 127.4, 126.9, 126.6, 126.3, 126.0, 125.4, 121.1, 120.6, 119.6, 118.6, 117.3, 115.2, 109.2, 48.7, 47.8, 45.5, 33.6, 21.5; **HRMS** (ESI) calculated for $C_{35}H_{32}N_2NaO_2S$ [M + Na$^+$]: 567.2077, found: 567.2077. **[α]$_D^{20}$** = -87.7 (c = 0.5, $CHCl_3$), **HPLC conditions**: with a Chiralpak AS-H column (90: 10 hexane: 2-propanol, 0.8 mL/min, 224 nm); tr (minor) = 12.61 min, tr (major) = 21.92 min, 91% ee.

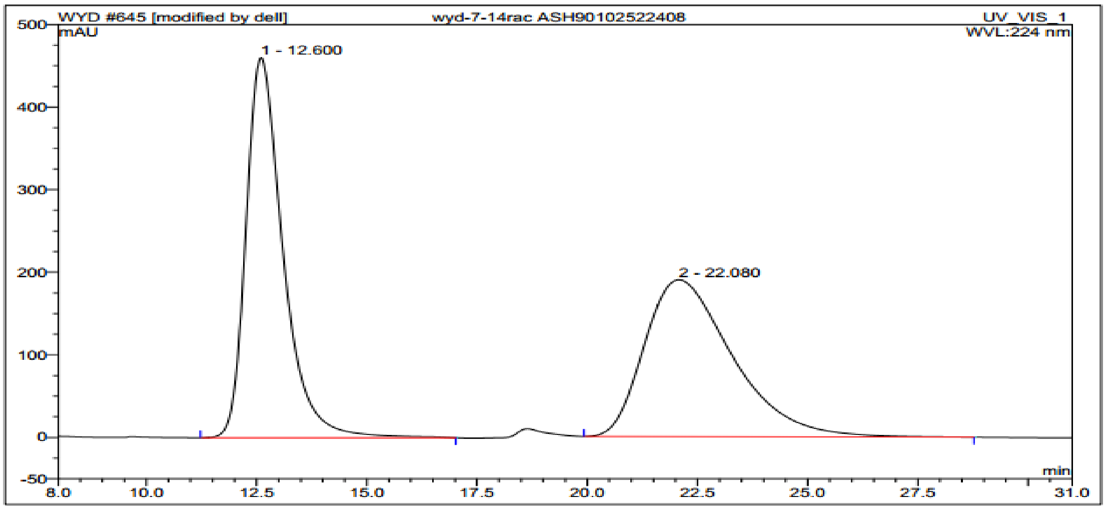

Peak #	Time(min)	Height (mAU)	Area (mAU*min)	Area(%)
1	12.60	459.987	448.234	50.47
2	22.08	189.783	439.944	49.53

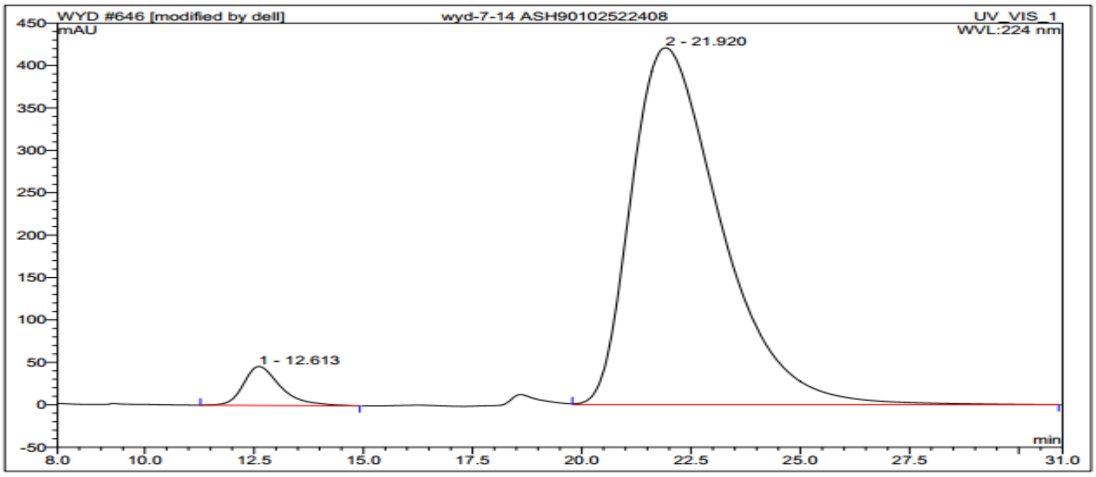

Peak #	Time(min)	Height (mAU)	Area (mAU*min)	Area(%)
1	12.61	45.873	45.121	4.31
2	21.92	420.890	1002.348	95.69

4. ***N*-((*Z*)-((2*R*,3*S*)-2-(1H-indol-3-yl)-3-phenylcyclobutylidene)methyl)-4-methyl-*N*-phenylbenzenesulfonamide (3d) .**

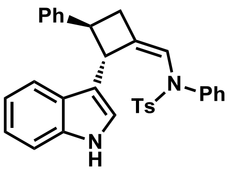

Isolated in 95% yield as white solid.

^{1}H NMR (400 MHz, CD_2Cl_2) δ 7.93 (s, 1 H), 7.29 (d, *J* = 8.0 Hz, 2 H), 7.24 (d, *J* = 8.0 Hz, 1 H), 7.18-7.09 (m, 5 H), 7.08-6.99 (m, 4 H), 6.90-6.81 (m, 2 H), 6.81-6.74 (m, 2 H), 6.61 (s, 1 H), 6.49 (q, *J* = 2.4 Hz, 1 H), 6.36 (d, *J* = 7.6 Hz, 2 H), 3.54-3.48 (m, 1 H), 3.45 (q, *J* = 8.0 Hz, 1 H), 3.23-3.14 (m, 1 H), 2.82-2.72 (m, 1 H), 2.30 (s, 3 H); **^{13}C NMR** (100 MHz, CD_2Cl_2) 145.2, 144.3, 139.6, 136.8, 135.6, 130.5, 129.8, 128.6, 128.5, 128.3, 127.8, 127.1, 126.8, 126.7, 126.5, 122.2, 121.9, 121.0, 119.7, 119.3, 116.5, 111.3, 48.4, 45.8, 34.2, 21.7; **HRMS** (ESI) calculated for $C_{32}H_{28}N_2NaO_2S$ [M + Na^{+}]: 527.1764, found: 527.1762. **$[\alpha]_D^{20}$** = -60.7 (c = 1.6, $CHCl_3$), **HPLC conditions**: with a Chiralpak AD-H column (70: 30 hexane: 2-propanol, 0.8 mL/min, 224 nm); tr (minor) = 10.07 min, tr (major) = 15.71 min, 72% ee.

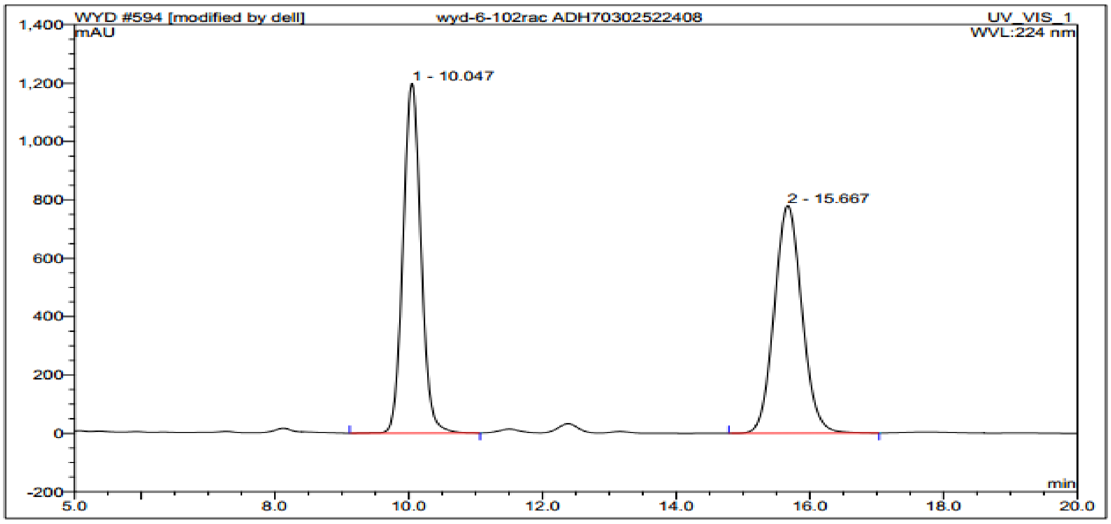

Peak #	Time(min)	Height (mAU)	Area (mAU*min)	Area(%)
1	10.05	1197.412	371.618	49.88
2	15.67	779.271	373.336	50.12

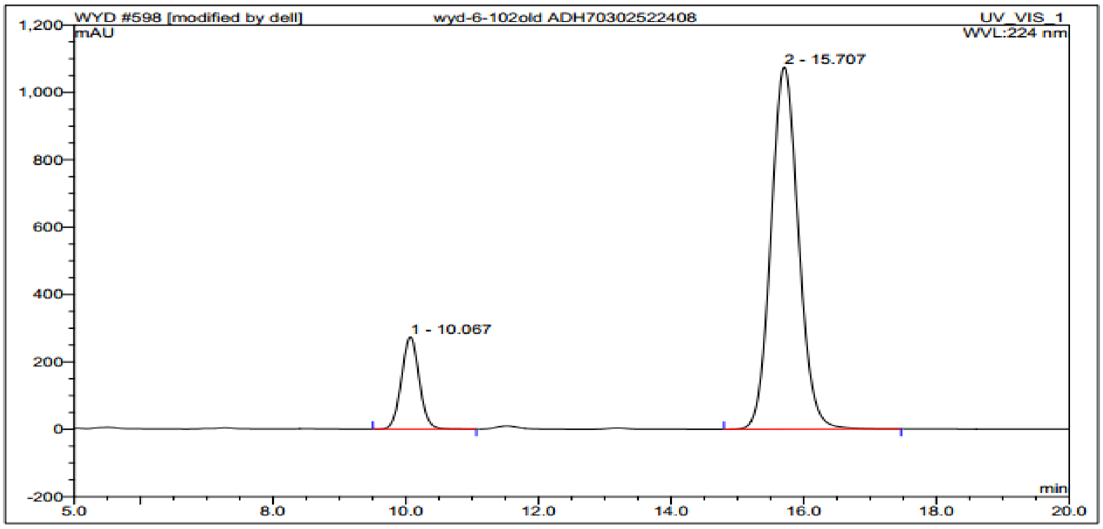

Peak #	Time (min)	Height (mAU)	Area (mAU*min)	Area (%)
1	10.07	273.003	84.199	14.03
2	15.71	1074.202	515.929	85.97

5. ***N*-((*Z*)-((2*R*,3*S*)-3-(4-bromophenyl)-2-(1-methyl-1H-indol-3-yl)cyclobutylidene)methyl)-4-methyl-*N*-phenylbenzenesulfonamide (3e) .**

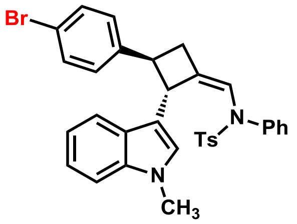

Isolated in 98% yield as white solid.

^{1}H NMR (400 MHz, $CDCl_3$) δ 7.39 (d, *J* = 8.0 Hz, 2H), 7.36-7.32 (m, 2H), 7.26-7.15 (m, 5H), 7.00-6.93 (m, 4H), 6.82 (t, *J* = 8.0 Hz, 2H), 6.61-6.57 (m, 1H), 6.55 (s, 1H), 6.45-6.40 (m, 2H), 3.70 (s, 3H), 3.54-3.45 (m, 2H), 3.27-3.21 (m, 1H), 2.85-2.79 (m, 1H), 2.40 (s, 3H); **^{13}C NMR** (100 MHz, $CDCl_3$) 143.7, 143.5, 138.9, 137.0, 135.3, 131.3, 129.4, 129.0, 128.23, 128.16, 127.8, 127.5, 126.7, 126.6, 126.4, 121.3, 120.7, 119.8, 119.4, 118.7, 114.5, 108.9, 48.1, 45.2, 33.5, 32.5, 21.5; **MS** (EI): m/z (%): 441 (M^+, 19.66), 443 (M^++2, 19.77), 44 (100), **HRMS** (EI) calculated for [$C_{33}H_{29}BrN_2O_2S$ – $C_7H_7O_2S$]: 441.0966, found: 441.0967. **$[\alpha]_D^{20}$** = -45.5 (c = 0.5, $CHCl_3$), **HPLC conditions**: with a Chiralpak AD-H column (95: 05 hexane: 2-propanol, 0.8 mL/min, 224 nm); tr (minor) = 28.61 min, tr (major) = 26.09 min, 96% ee.

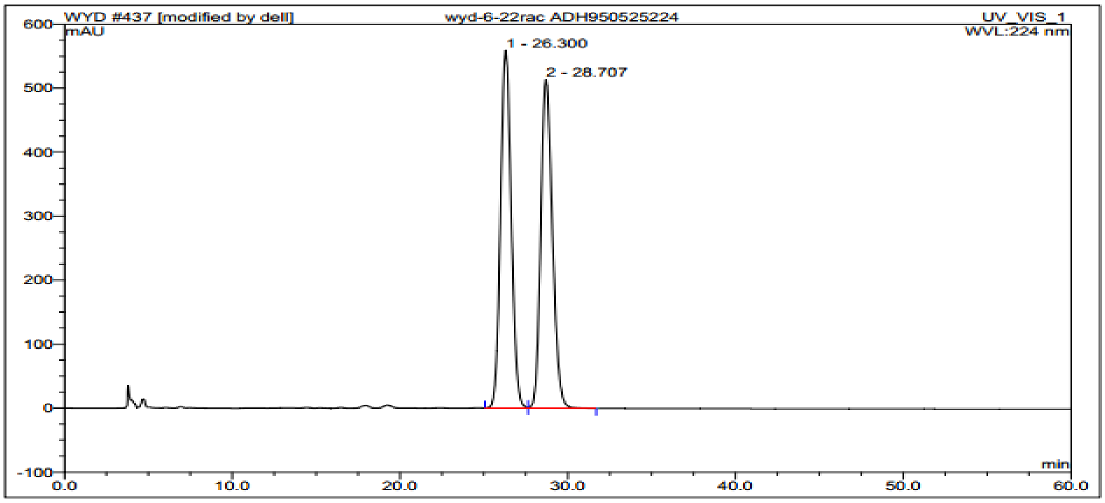

Peak #	Time(min)	Height (mAU)	Area (mAU*min)	Area(%)
1	26.30	559.121	413.456	49.96
2	28.71	513.412	414.112	50.04

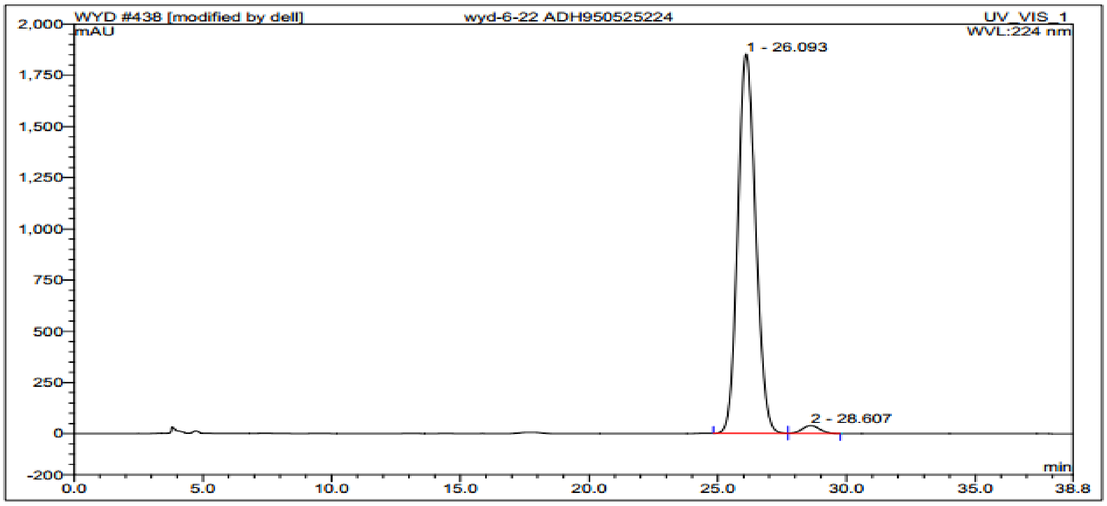

Peak #	Time (min)	Height (mAU)	Area (mAU*min)	Area (%)
1	26.09	1852.772	1484.588	97.93
2	28.61	39.145	31.403	2.07

6. ***N*-((*Z*)-((2*R*,3*S*)-3-(4-chlorophenyl)-2-(1-methyl-1H-indol-3-yl)cyclobutylidene)methyl)-4-methyl-*N*-phenylbenzenesulfonamide (3f).**

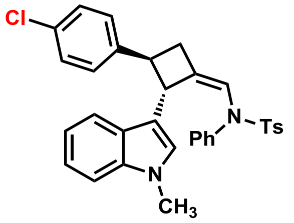

Isolated in 98% yield as white solid.

^{1}H NMR (400 MHz, $CDCl_3$) δ 7.38 (d, *J* = 6.8 Hz, 2 H), 7.24-7.14 (m, 7 H), 7.03 (d, *J* = 7.6 Hz, 2 H), 6.96-6.91 (m, 2 H), 6.80 (t, *J* = 14.4 Hz, 2 H), 6.58 (s, 1 H), 6.52 (s, 1 H), 6.43-6.38 (m, 2 H), 3.67 (s, 3 H), 3.53-3.46 (m, 2 H), 3.26-3.18 (m, 1 H), 2.83-2.78 (m, 1 H), 2.37 (s, 3 H); **^{13}C NMR** (100 MHz, $CDCl_3$) δ 143.5, 143.1, 138.9, 137.0, 135.2, 131.7, 129.3, 129.1, 128.3, 128.2, 127.8, 127.7, 127.5, 126.61, 126.57, 126.3, 121.2, 120.6, 119.4, 118.6, 114.4, 108.8, 48.1, 45.0, 33.5, 32.5, 21.5; **MS** (EI): m/z (%): 397 (M^+, 68.45), 399 (M^++2), 241 (100); **HRMS** (EI) calculated for [$C_{33}H_{29}ClN_2O_2S$ – $C_7H_7O_2S$]: 397.1472, found: 397.1469. **$[\alpha]_D^{20}$** = -66.3 (c = 0.5, $CHCl_3$), **HPLC conditions**: Daicel Chiralpak AS-H column (90:10 hexane: 2-propanol, 0.8 mL/min, 224 nm); tr (minor) = 13.02 min, tr (major) = 15.64 min, 96% ee.

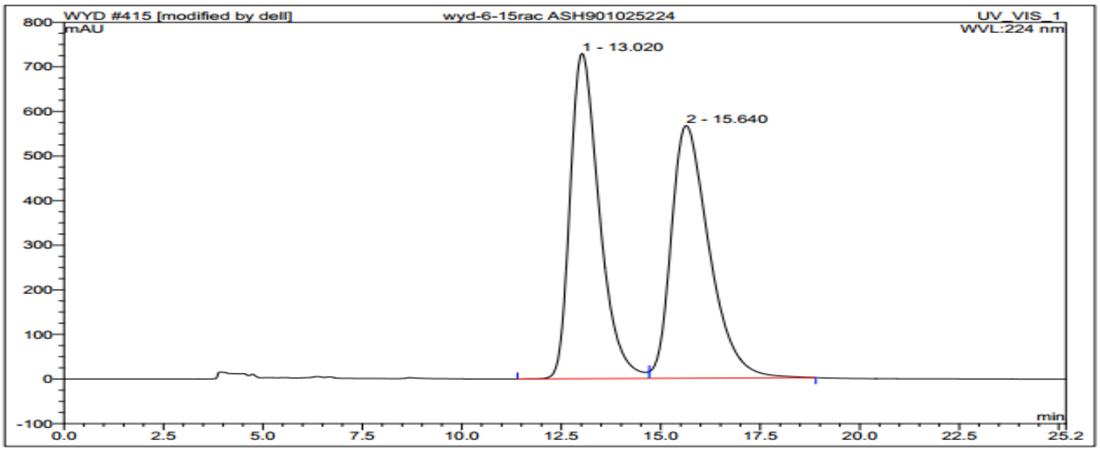

Peak #	Time(min)	Height (mAU)	Area (mAU*min)	Area(%)
1	13.02	729.513	621.615	49.81
2	15.64	566.550	626.424	50.19

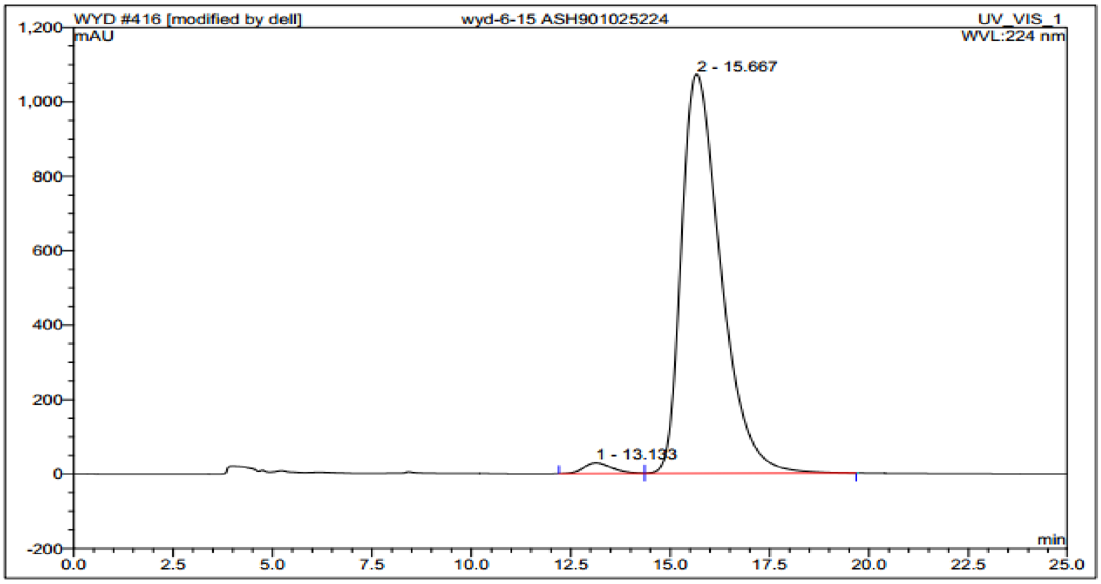

Peak #	Time (min)	Height (mAU)	Area (mAU*min)	Area (%)
1	13.13	28.382	23.963	1.91
2	15.67	1072.956	1233.627	98.09

7. 4-methyl-*N*-((*Z*)-((2*R*,3*S*)-2-(1-methyl-1H-indol-3-yl)-3-(p-tolyl)cyclobutylidene)methyl)-*N*-phenylbenzenesulfonamide (3g) .

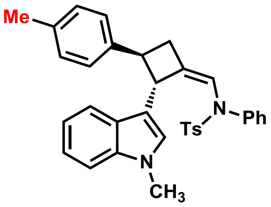

Isolated in 97% yield as white solid.

^{1}H NMR (400 MHz, $CDCl_3$) δ 7.39 (d, *J* = 8.0 Hz, 2 H), 7.24-7.13 (m, 5 H), 7.04-7.00 (m, 4 H), 6.93 (t, *J* = 8.0 Hz, 2 H), 6.81 (t, *J* = 8.0 Hz, 2 H), 6.57-6.54 (m, 1 H), 6.54 (s, 1 H), 6.45-6.40 (m, 2 H), 3.68 (s, 3 H), 3.58-3.53 (m, 1 H), 3.49 (q, *J* = 8.0 Hz, 1 H), 3.28-3.18 (m, 1 H), 2.86-2.80 (m, 1 H), 2.39 (s, 3 H), 2.28 (s, 3 H); **^{13}C NMR** (100 MHz, $CDCl_3$) 143.5, 141.7, 139.1, 137.0, 135.5, 135.4, 130.1, 129.3, 128.9, 128.2, 127.8, 127.5, 126.8, 126.5, 126.4, 126.3, 121.1, 120.4, 119.6, 118.5, 114.8, 108.7, 48.0, 45.3, 33.7, 32.5, 21.5, 21.0; **MS** (EI): m/z (%): 377 (100), **HRMS** (EI) calculated for [$C_{34}H_{32}N_2O_2S$ – $C_7H_7O_2S$]: 377.2018, found: 377.2017. **$[\alpha]_D^{20}$** = -95.3 (c = 0.5, $CHCl_3$), **HPLC conditions**: with a Chiralpak OD-H column (95: 05 hexane: 2-propanol, 0.8 mL/min, 224 nm); tr (minor) = 15.01 min, tr (major) = 13.81 min, 95% ee.

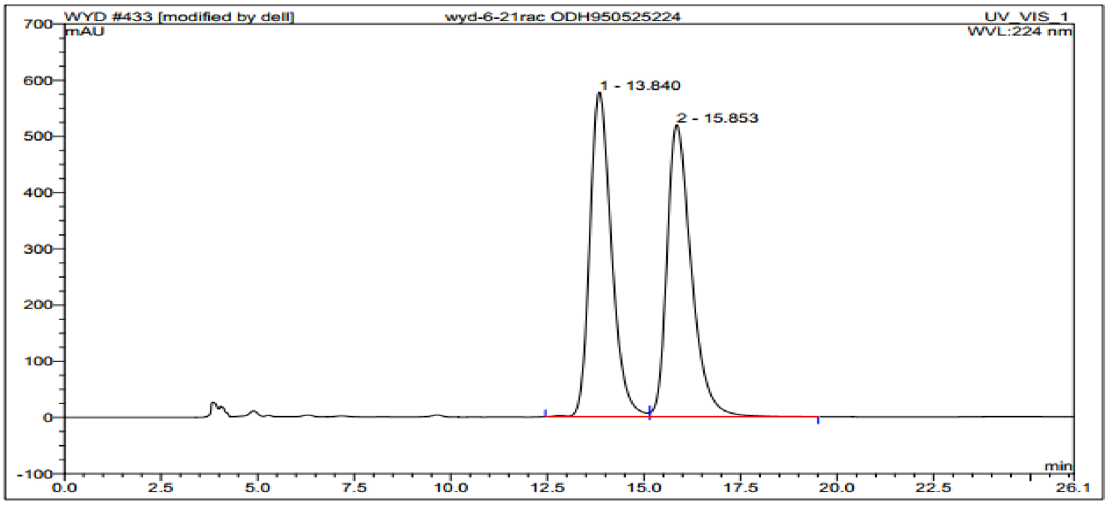

Peak #	Time(min)	Height (mAU)	Area (mAU*min)	Area (%)
1	13.84	577.530	374.504	49.81
2	15.85	519.288	377.342	50.19

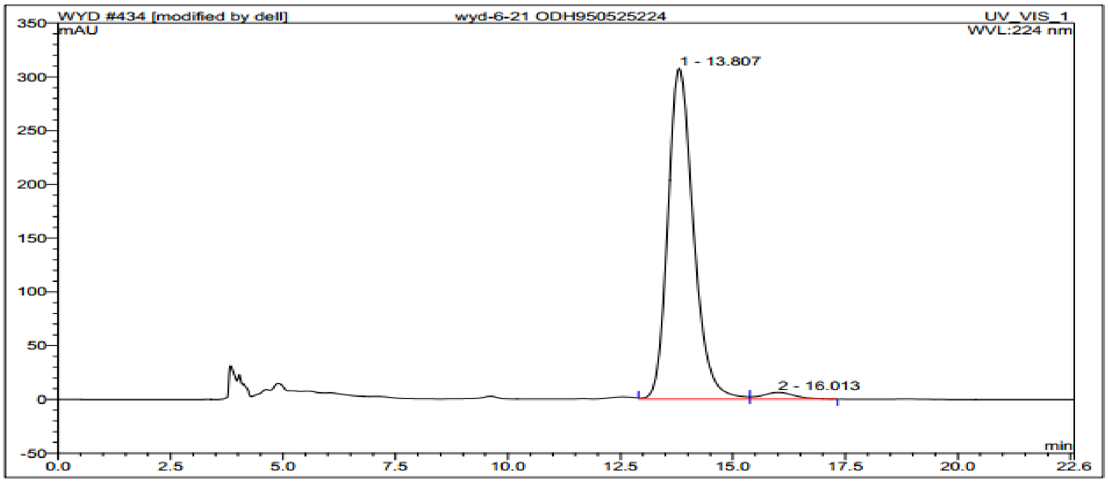

Peak #	Time(min)	Height (mAU)	Area (mAU*min)	Area(%)
1	13.81	307.566	201.737	97.57
2	16.01	6.046	5.019	2.43

8. 4-methyl-*N*-((*Z*)-((2*R*,3*S*)-2-(1-methyl-1H-indol-3-yl)-3-(4-(trifluoromethyl)phenyl)cyclobutylidene)methyl)-*N*-phenylbenzenesulfonamide (3h).

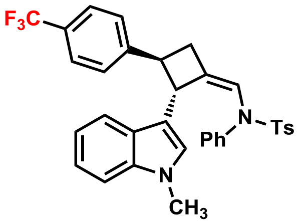

Isolated in 99% yield as white solid.

^{1}H NMR (400 MHz, $CDCl_3$) δ 7.47 (d, *J* = 8.0 Hz, 2 H), 7.39 (d, *J* = 8.4 Hz, 2 H), 7.25-7.15 (m, 7 H), 6.97-6.92 (m, 2 H), 6.81 (t, *J* = 15.6 Hz, 2 H), 6.61 (s, 1 H), 6.53 (s, 1 H), 6.44-6.39 (m, 2 H), 3.68 (s, 3 H), 3.62-3.55 (m, 2 H), 3.32-3.24 (m, 1 H), 2.90-2.83 (m, 1 H), 2.38 (s, 3 H); **^{13}C NMR** (100 MHz, $CDCl_3$) δ 148.7, 143.6, 138.9, 137.0, 135.2, 129.4, 128.5, 128.3 (q, *J* = 30 Hz), 128.2, 127.8, 127.5, 126.7, 126.64, 126.57, 126.4, 125.2 (q, *J* = 4 Hz), , 124.2 (q, *J* = 270 Hz), 121.3, 120.9, 119.3, 118.7, 114.3, 108.9, 48.0, 45.4, 33.3, 32.5, 21.5; **^{19}F NMR** (376 MHz, $CDCl_3$) δ -62.26; **MS** (EI): m/z (%): 431 (100); **HRMS** (EI) calculated for [$C_{34}H_{29}F_3N_2O_2S$ – $C_7H_7O_2S$]: 431.1735, found: 431.1732. **$[\alpha]_D^{20}$** = -89.3 (c = 0.5, $CHCl_3$), **HPLC conditions**: with a Chiralpak OD-3 column (95: 05 hexane: 2-propanol, 0.3 mL/min, 224 nm); tr (minor) = 36.14 min, tr (major) = 32.79 min, 96% ee.

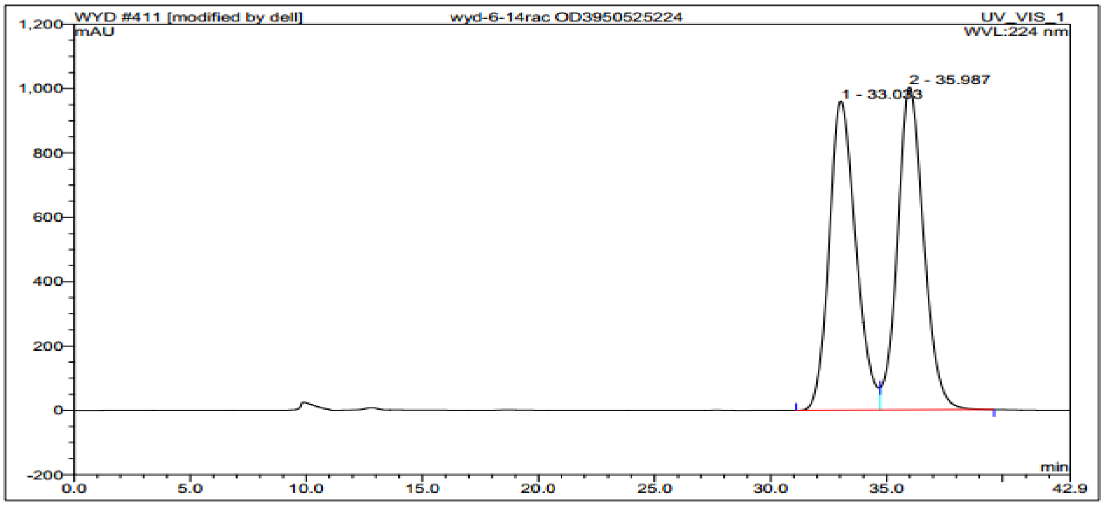

Peak #	Time(min)	Height (mAU)	Area (mAU*min)	Area(%)
1	33.03	960.168	1284.636	49.54
2	35.99	1003.308	1308.617	50.46

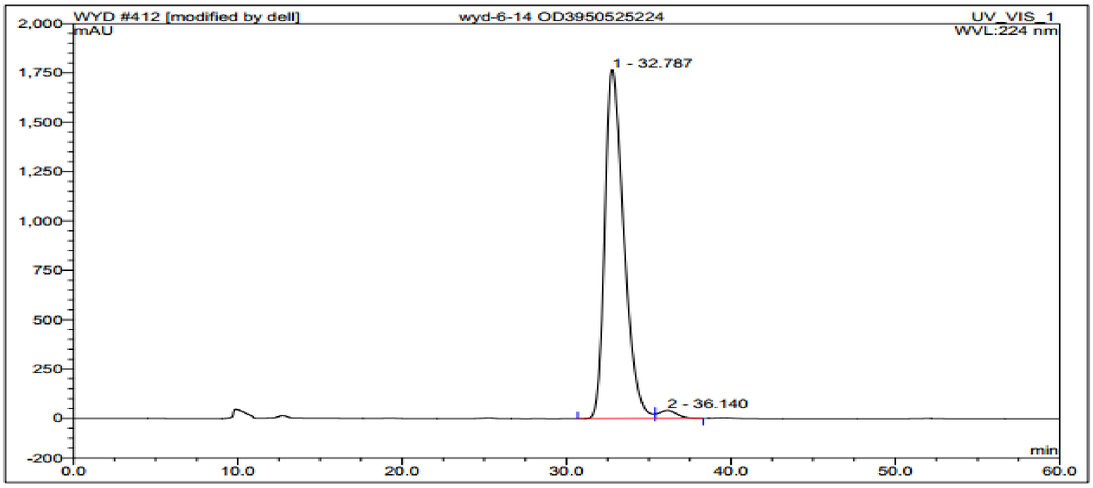

Peak #	Time (min)	Height (mAU)	Area (mAU*min)	Area (%)
1	32.79	2366.763	2366.763	97.88
2	36.14	51.314	51.314	2.12

9. ***N*-((Z)-((2*R*,3*S*)-3-(4-methoxyphenyl)-2-(1-methyl-1H-indol-3-yl)cyclobutylidene)methyl)-4-methyl-*N*-phenylbenzenesulfonamide (3i) .**

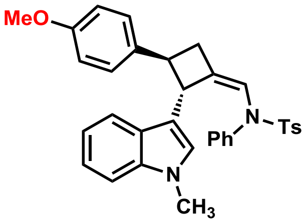

Isolated in 96% yield as white solid.

^{1}H NMR (400 MHz, $CDCl_3$) δ 7.39 (d, *J* = 8.4 Hz, 2 H), 7.24-7.13 (m, 5 H), 7.04 (d, *J* = 8.4 Hz, 2 H), 6.95-6.91 (m, 2 H), 6.83-6.75 (m, 4 H), 6.57-6.54 (m, 1 H), 6.53 (s, 1 H), 6.45-6.40 (m, 2 H), 3.73 (s, 3 H), 3.67 (s, 3 H), 3.56-3.52 (m, 1 H), 3.50-3.44 (m, 1 H), 3.25-3.19 (m, 1 H), 2.84-2.77 (m, 1 H), 2.38 (s, 3 H); **^{13}C NMR** (100 MHz, $CDCl_3$) δ 157.9, 143.4, 139.0, 137.0, 136.9, 135.3, 130.1, 129.3, 128.2, 127.7, 127.5, 127.3, 126.8, 126.5, 126.3, 121.1, 120.3, 119.6, 118.5, 114.7, 113.6, 108.7, 55.2, 48.2, 44.9, 33.8, 32.5, 21.5; **MS** (EI): m/z (%): 393 (M^+, 14.14); 91 (100), **HRMS** (EI) calculated for [$C_{34}H_{32}N_2O_3S$ – $C_7H_7O_2S$]: 393.1967, found: 393.1965. **$[\alpha]_D^{20}$** = -73.0 (c = 0.5, $CHCl_3$), **HPLC conditions**: with a Chiralpak AD-H column (95: 05 hexane: 2-propanol, 0.8 mL/min, 224 nm); tr (minor) = 30.03 min, tr (major) = 28.21 min, 95% ee.

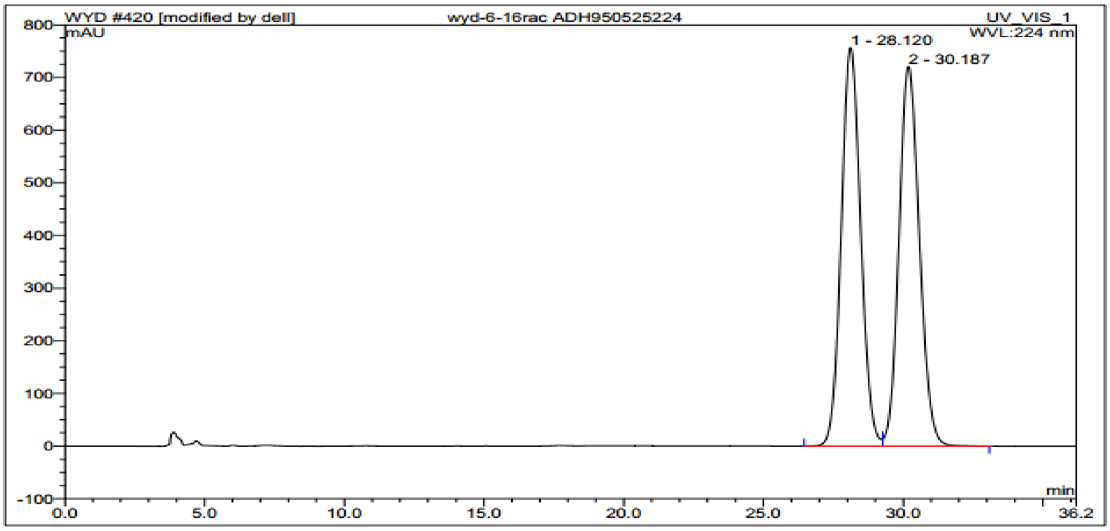

Peak #	Time (min)	Height (mAU)	Area (mAU*min)	Area (%)
1	28.12	757.035	611.589	49.94
2	30.19	721.491	612.953	50.06

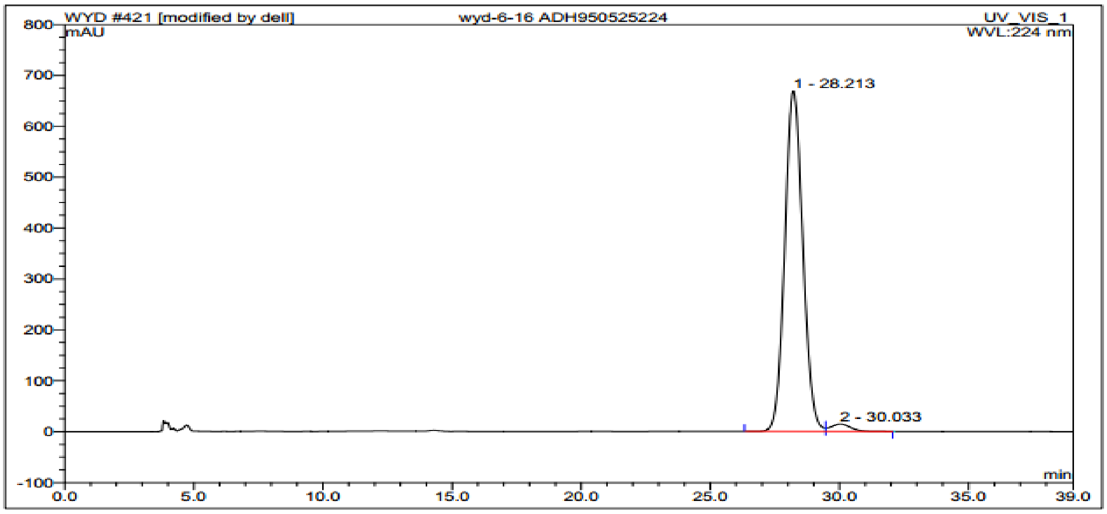

Peak #	Time (min)	Height (mAU)	Area (mAU*min)	Area (%)
1	28.21	669.192	542.324	97.73
2	30.03	14.559	12.575	2.27

10. ***N*-((*Z*)-((2*R*,3*S*)-2-(5-methoxy-1-methyl-1H-indol-3-yl)-3-phenylcyclobutylidene)methyl)-4-methyl-*N*-phenylbenzenesulfonamide (3j) .**

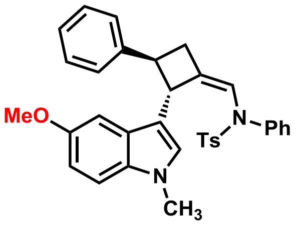

Isolated in 76% yield as white solid.

^{1}H NMR (400 MHz, $CDCl_3$) δ 7.38 (d, *J* = 8.0 Hz, 2 H), 7.24-7.10 (m, 9 H), 6.94 (t, *J* = 7.2 Hz, 1 H), 6.84-6.81 (m, 3 H), 6.69-6.65 (m, 1 H), 6.58-6.53 (m, 1 H), 6.44-6.38 (m, 2 H), 3.70 (s, 3 H), 3.65 (s, 3 H), 3.59-3.49 (m, 2 H), 3.25-3.19 (m, 1 H), 2.89-2.83 (m, 1 H), 2.37 (s, 3 H); **^{13}C NMR** (100 MHz, $CDCl_3$) δ 153.4, 144.6, 143.5, 139.1, 135.2, 132.4, 130.3, 129.3, 128.2, 128.1, 127.7, 127.5, 127.1, 127.0, 126.41, 126.37, 126.0, 120.3, 114.2, 111.4, 109.5, 101.6, 55.8, 48.1, 45.5, 33.4, 32.6, 21.5; **MS** (EI): m/z (%): 393 (100); **HRMS** (EI) calculated for [$C_{34}H_{32}N_2O_3S$ – $C_7H_7O_2S$]: 393.1967, found: 393.1965. **$[\alpha]_D^{20}$** = -102.2 (c = 0.5, $CHCl_3$), **HPLC conditions**: with a Chiralpak AS-H column (95: 05 hexane: 2-propanol, 0.8 mL/min, 224 nm); tr (minor) = 20.55 min, tr (major) = 25.79 min, 96% ee.

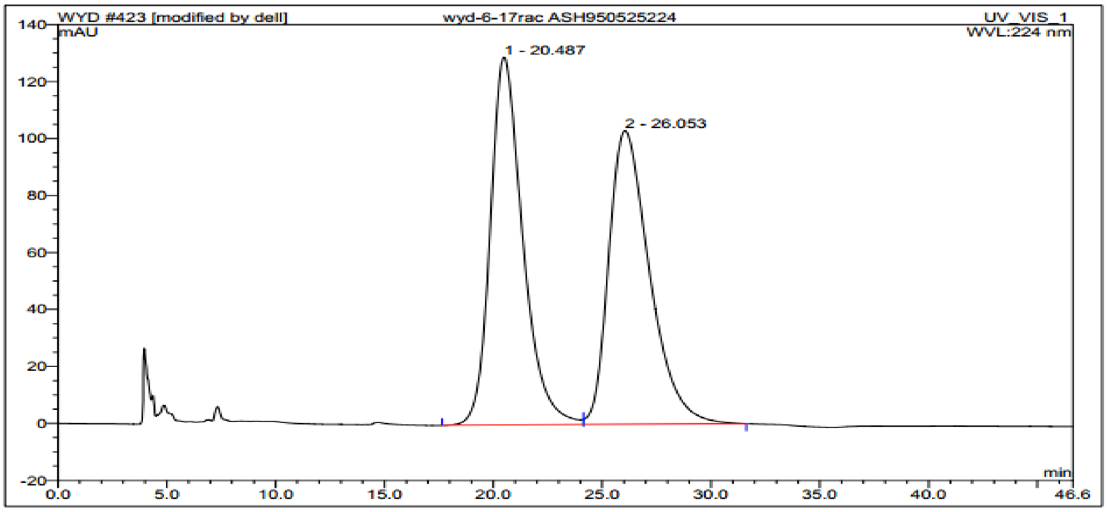

Peak #	Time(min)	Height (mAU)	Area (mAU*min)	Area (%)
1	20.49	129.100	223.055	49.92
2	26.05	103.113	223.786	50.08

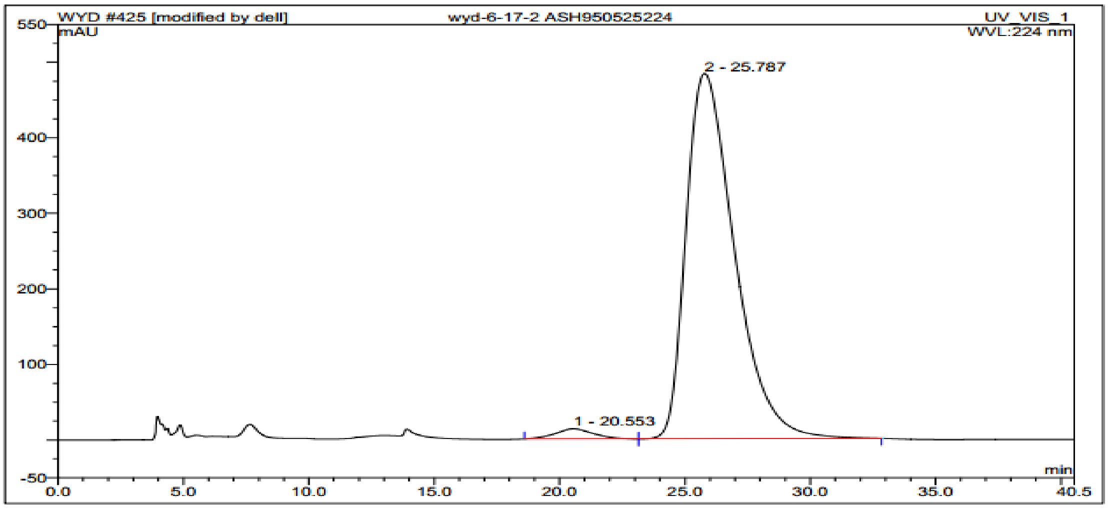

Peak #	Time(min)	Height (mAU)	Area (mAU*min)	Area (%)
1	20.55	13.057	22.387	2.03
2	25.79	483.120	1080.073	97.97

11. ***N*-((Z)-((2*R*,3*S*)-2-(1,5-dimethyl-1H-indol-3-yl)-3-phenylcyclobutylidene)methyl)-4-methyl-*N*-phenylbenzenesulfonamide (3k) .**

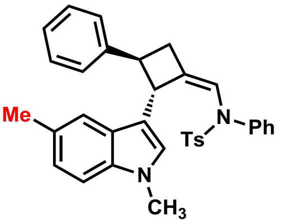

Isolated in 91% yield as white solid.

^{1}H NMR (400 MHz, $CDCl_3$) δ 7.37 (d, *J* = 8.4 Hz, 2 H), 7.23-7.09 (m, 8 H), 7.03-6.92 (m, 3 H), 6.85-6.78 (m, 2 H), 6.56-6.52 (m, 1 H), 6.47 (s, 1 H), 6.45-6.40 (m, 2 H), 3.62 (s, 3 H), 3.61-3.51 (m, 2 H), 3.27-3.21 (m, 1 H), 2.88-2.82 (m, 1 H), 2.35 (s, 6 H); **^{13}C NMR** (100 MHz, $CDCl_3$) 144.7, 143.4, 139.1, 135.4, 135.2, 130.6, 129.3, 128.2, 128.1, 127.7, 127.48, 127.45, 127.0, 126.5, 126.4, 126.0, 122.7, 120.2, 119.3, 114.0, 108.4, 48.0, 45.3, 33.5, 32.5, 21.5, 21.4; **MS** (EI): m/z (%): 377 (M^+, 75.67), 44 (100), **HRMS** (EI) calculated for [$C_{34}H_{32}N_2O_2S$ – $C_7H_7O_2S$]: 377.2018, found: 377.2019. **$[\alpha]_D^{20}$** = -118.3 (c = 0.5, $CHCl_3$), **HPLC conditions**: with a Chiralpak 2-AS-H column (95: 05 hexane: 2-propanol, 0.8 mL/min, 224 nm); tr (minor) = 35.07 min, tr (major) = 37.35 min, 95% ee.

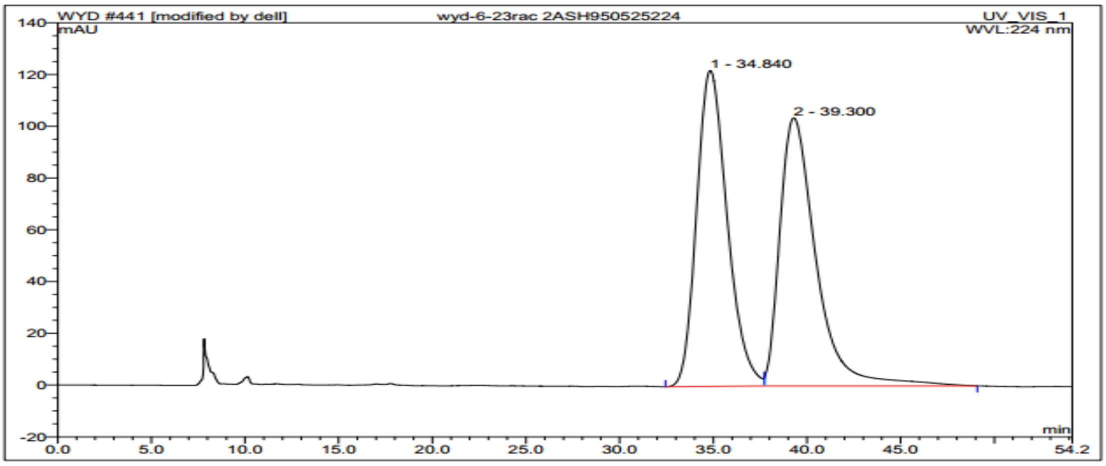

Peak #	Time(min)	Height (mAU)	Area (mAU*min)	Area (%)
1	34.84	122.001	230.937	49.95
2	39.30	103.547	231.393	50.05

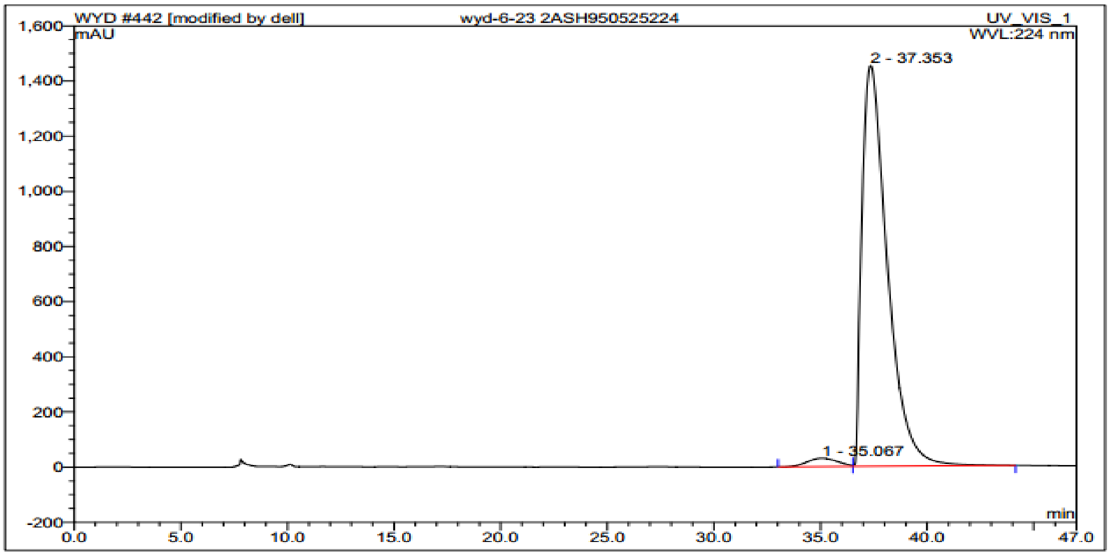

Peak #	Time(min)	Height (mAU)	Area (mAU*min)	Area (%)
1	35.07	30.249	50.187	2.44
2	37.35	1454.341	2006.734	97.56

12. ***N*-((*Z*)-((2*R*,3*S*)-2-(5-bromo-1-methyl-1H-indol-3-yl)-3-phenylcyclobutylidene)methyl)-4-methyl-*N*-phenylbenzenesulfonamide (3l) .**

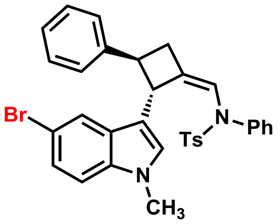

Isolated in 99% yield as white solid.

^{1}H NMR (400 MHz, $CDCl_3$) δ 7.38 (d, *J* = 8.0 Hz, 2 H), 7.24-7.07 (m, 10 H), 6.95 (t, *J* = 7.6 Hz, 1 H), 6.81 (t, *J* = 7.6 Hz, 2 H), 6.62 (s, 1 H), 6.56-6.53 (m, 1 H), 6.41 (d, *J* = 7.6 Hz, 2 H), 3.66 (s, 3 H), 3.57-3.47 (m, 2 H), 3.27-3.17 (m, 1 H), 2.89-2.83 (m, 1 H), 2.37 (s, 3 H); **^{13}C NMR** (100 MHz, $CDCl_3$) 144.2, 143.5, 138.9, 135.5, 135.1, 129.7, 129.3, 128.4, 128.3, 127.9, 127.81, 127.76, 127.4, 126.5, 126.2, 126.2, 123.9, 121.9, 120.4, 114.5, 112.0, 110.3, 47.5, 45.7, 33.5, 32.7, 21.5; **MS** (EI): m/z (%): 441 (M^+, 27.59), 443 (M^++2, 27.43); 44 (100), **HRMS** (EI) calculated for [$C_{33}H_{29}BrN_2O_2S$ - $C_7H_7O_2S$]: 441.0966, found: 441.0969. **$[\alpha]_D^{20}$** = -102.6 (c = 0.5, $CHCl_3$), **HPLC conditions**: with a Chiralpak AD-H column (95: 05 hexane: 2-propanol, 0.8 mL/min, 224 nm); tr (minor) = 20.46 min, tr (major) = 24.33 min, 95% ee.

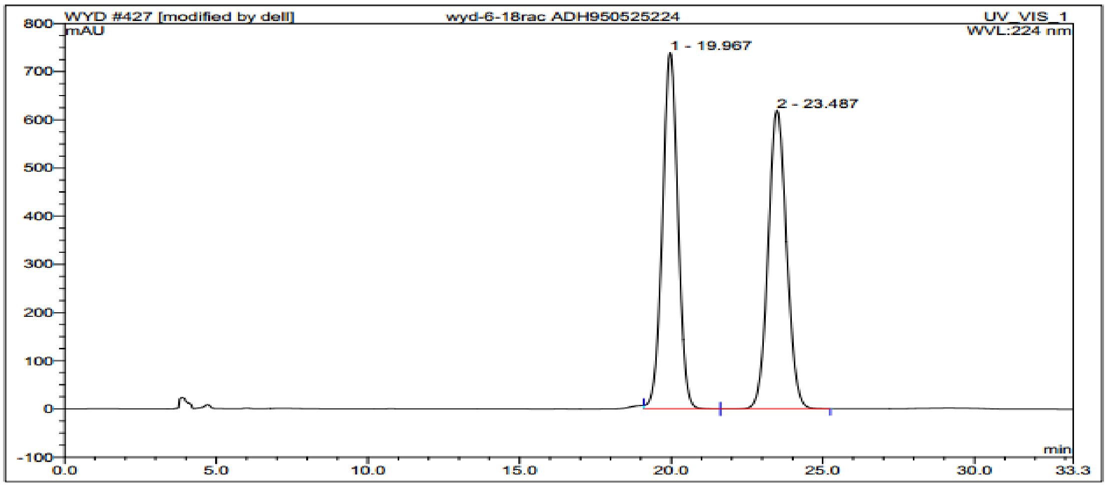

Peak #	Time(min)	Height (mAU)	Area (mAU*min)	Area(%)
1	19.97	739.302	436.783	50.06
2	23.49	619.881	435.686	49.94

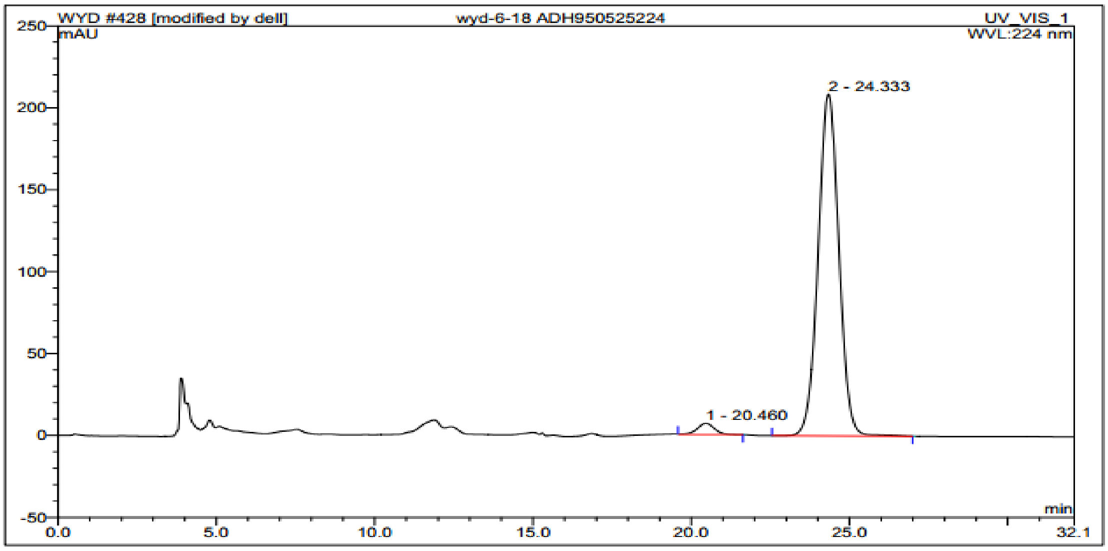

Peak #	Time(min)	Height (mAU)	Area (mAU*min)	Area (%)
1	20.46	6.954	4.486	2.75
2	24.33	208.689	158.572	97.25

13. ***N*-((Z)-((2*R*,3*S*)-2-(4-bromo-1-methyl-1H-indol-3-yl)-3-phenylcyclobutylidene)methyl)-4-methyl-*N*-phenylbenzenesulfonamide. (3m)**

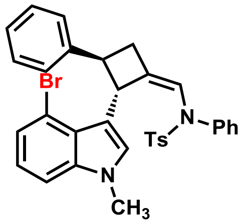

Isolated in 93% yield as white solid.

^{1}H NMR (400 MHz, $CDCl_3$) δ7.44 (d, *J* = 8.0 Hz, 2 H), 7.24-7.15 (m, 5 H), 7.15-7.08 (m, 4 H), 6.98 (d, *J* = 7.2 Hz, 1 H), 6.92 (t, *J* = 8.0 Hz, 1 H), 6.84 (t, *J* = 7.2 Hz, 1 H), 6.80-6.72 (m, 2 H), 6.59 (s, 1 H), 6.48 (d, *J* = 7.6 Hz, 2 H), 4.27 (s, 1 H), 3.77 (s, 3 H), 3.32-3.13 (m, 2 H), 2.80-2.68 (m, 1 H), 2.40 (s, 3 H); **^{13}C NMR** (100 MHz, $CDCl_3$) 144.0, 143.6, 138.7, 137.9, 135.2, 129.9, 129.4, 129.3, 128.2, 129.0, 127.8, 127.5, 126.94, 126.85, 126.0, 124.4, 123.3, 121.7, 120.1, 116.1, 114.1, 108.1, 49.1, 46.2, 34.1, 33.0, 21.6; **MS** (EI): m/z (%): 441 (M^+, 49.67), 443 (M^++2, 50.17), 44 (100), **HRMS** (EI) calculated for [$C_{33}H_{29}BrN_2O_2S$ – $C_7H_7O_2S$]: 441.0966, found: 441.0968. **$[\alpha]_D^{20}$** = -99.8 (c = 0.4, $CHCl_3$), **HPLC conditions**: with a Chiralpak AD-H column (90: 10 hexane: 2-propanol, 0.8 mL/min, 224 nm); tr (minor) = 14.11 min, tr (major) = 9.41 min, 95% ee.

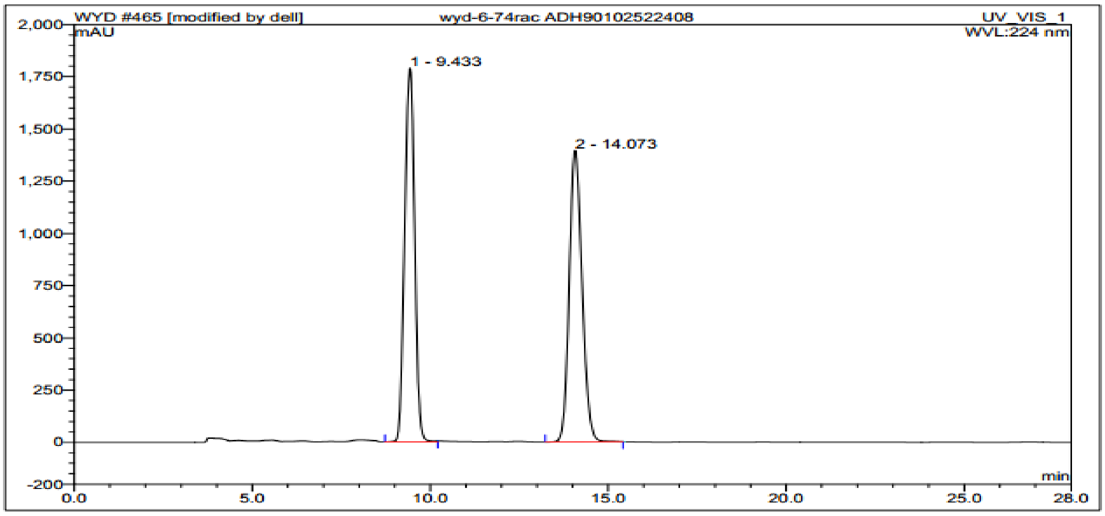

Peak #	Time(min)	Height (mAU)	Area (mAU*min)	Area (%)
1	9.43	1788.558	581.612	50.07
2	14.07	1394.268	579.920	49.93

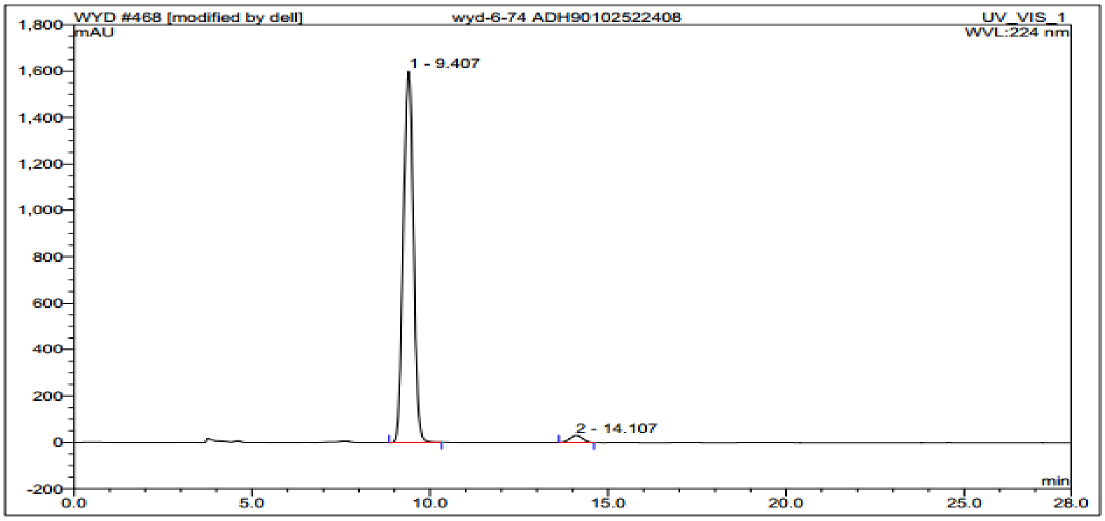

Peak #	Time(min)	Height (mAU)	Area (mAU*min)	Area (%)
1	9.41	1599.430	527.389	97.74
2	14.11	29.950	12.170	2.26

14. ***N*-((*Z*)-((2*R*,3*S*)-2-(1H-indol-3-yl)-3-phenylcyclobutylidene)methyl)-4-methyl-*N*-phenylbenzenesulfonamide(3n).**

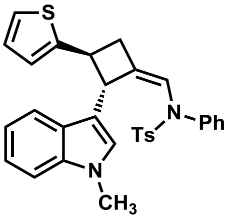

Isolated in 90% yield as white solid.

^{1}H NMR (400 MHz, $CDCl_3$) δ 7.38 (d, *J* = 8.4 Hz, 2 H), 7.29 (d, *J* = 8.0 Hz, 1 H), 7.24-7.12 (m, 4 H), 7.07 (d, *J* = 4.8 Hz, 1 H), 6.94 (q, *J* = 7.6 Hz, 2 H), 6.85 (q, *J* = 3.6 Hz, 1 H), 6.80 (t, *J* = 7.6 Hz, 2 H), 6.69 (d, *J* = 3.6 Hz, 1 H), 6.64-6.59 (m, 1 H), 6.49 (s, 1 H), 6.41 (d, *J* = 7.6 Hz, 2 H), 3.73-3.63 (m, 1 H), 3.66 (s, 3 H), 3.58-3.51 (m, 1 H), 3.35-3.25 (m, 1 H), 2.91-2.82 (m, 1 H), 2.37 (s, 3 H); **^{13}C NMR** (100 MHz, $CDCl_3$) 148.6, 143.5, 138.6, 136.9, 135.1, 129.3, 128.3, 127.7, 127.4, 127.3, 126.7, 126.59, 126.56, 126.3, 123.0, 122.9, 121.1, 120.7, 119.5, 118.5, 114.0, 108.7, 49.5, 41.5, 35.4, 32.5, 21.5; **HRMS** (ESI) calculated for $C_{31}H_{28}N_2NaO_2S_2$ [M + Na$^+$]: 547.1484, found: 547.1483. **$[\alpha]_D^{20}$** = -91.1 (c = 0.5, $CHCl_3$), **HPLC conditions**: with a Chiralpak AD-H column (95: 05 hexane: 2-propanol, 0.8 mL/min, 224 nm); tr (minor) = 26.43 min, tr (major) = 19.05 min, 93% ee.

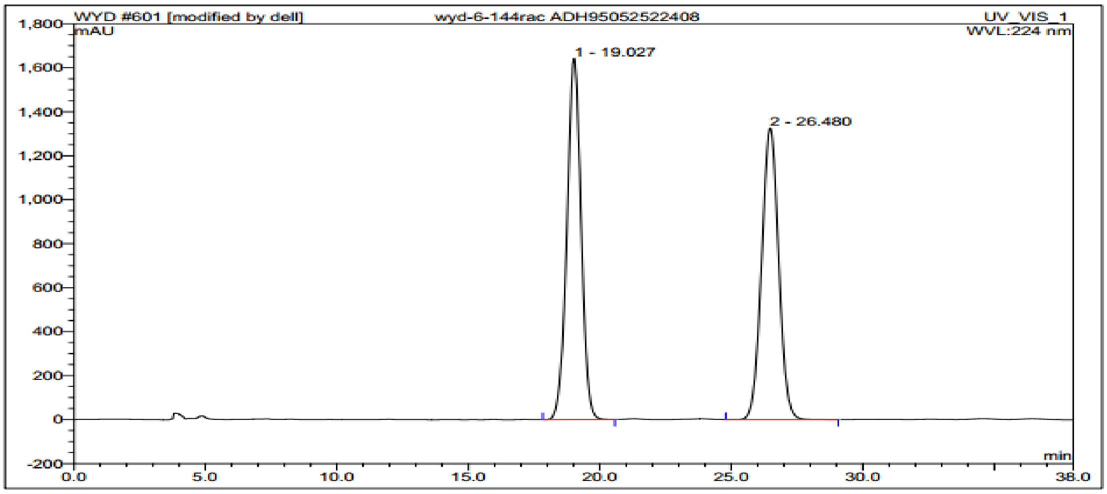

Peak #	Time(min)	Height (mAU)	Area (mAU*min)	Area (%)
1	19.03	1642.633	1025.131	50.73
2	26.48	1325.620	995.511	49.27

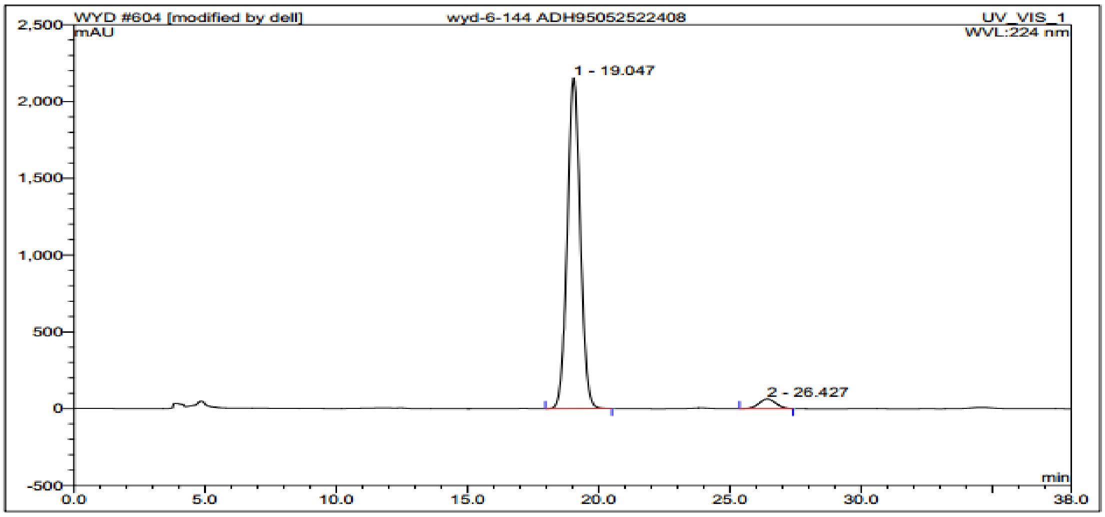

Peak #	Time(min)	Height (mAU)	Area (mAU*min)	Area (%)
1	19.05	2154.583	1231.695	96.42
2	26.43	62.584	45.780	3.58

15. ***N*-(4-methoxyphenyl)-4-methyl-*N*-((*Z*)-((2*R*,3*S*)-2-(1-methyl-1H-indol-3-yl)-3-phenylcyclobutylidene)methyl)benzenesulfonamide(3o).**

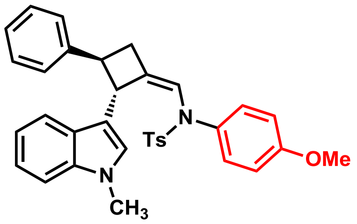

Isolated in 98% yield as white solid.

^{1}H NMR (400 MHz, $CDCl_3$) δ7.40 (d, *J* = 7.6 Hz, 2 H), 7.25-7.12 (m, 10 H), 6.96-6.90 (m, 1 H), 6.65 (s, 1 H), 6.61-6.58 (m, 1 H), 6.32-6.22 (m, 4 H), 3.72 (s, 3 H), 3.62-3.55 (m, 1 H), 3.56 (s, 3 H), 3.49 (q, *J* = 8.0 Hz, 1 H), 3.27-3.20 (m, 1 H), 2.90-2.80 (m, 1 H), 2.40 (s, 3 H);**^{13}C NMR** (100 MHz, $CDCl_3$) 158.1, 144.8, 143.4, 136.9, 135.3, 131.3, 129.7, 129.3, 128.2, 127.58, 127.55, 126.8, 126.4, 126.0, 121.1, 120.7, 119.5, 118.5, 115.4, 112.9, 108.7, 55.1, 47.6, 46.1, 33.5, 32.6, 21.5; **MS** (EI): m/z (%): 393 (M^+, 47.32), 44 (100), **HRMS** (EI) calculated for [$C_{34}H_{32}N_2O_3S$ – $C_7H_7O_2S$]: 393.1967, found: 393.1964. **$[\alpha]_D^{20}$** = -156.3 (c = 0.5, $CHCl_3$), **HPLC conditions**: with a Chiralpak AD-H column (95: 05 hexane: 2-propanol, 0.8 mL/min, 224 nm); tr (minor) = 30.95 min, tr (major) = 26.80 min, 96% ee.

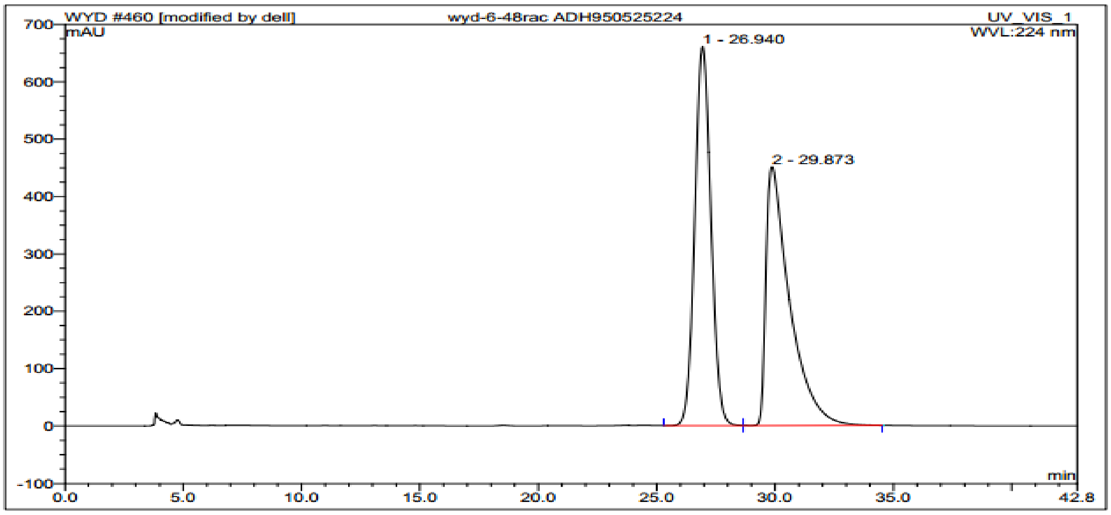

Peak #	Time(min)	Height (mAU)	Area (mAU*min)	Area (%)
1	26.94	660.808	526.064	50.21
2	29.87	451.221	521.684	49.79

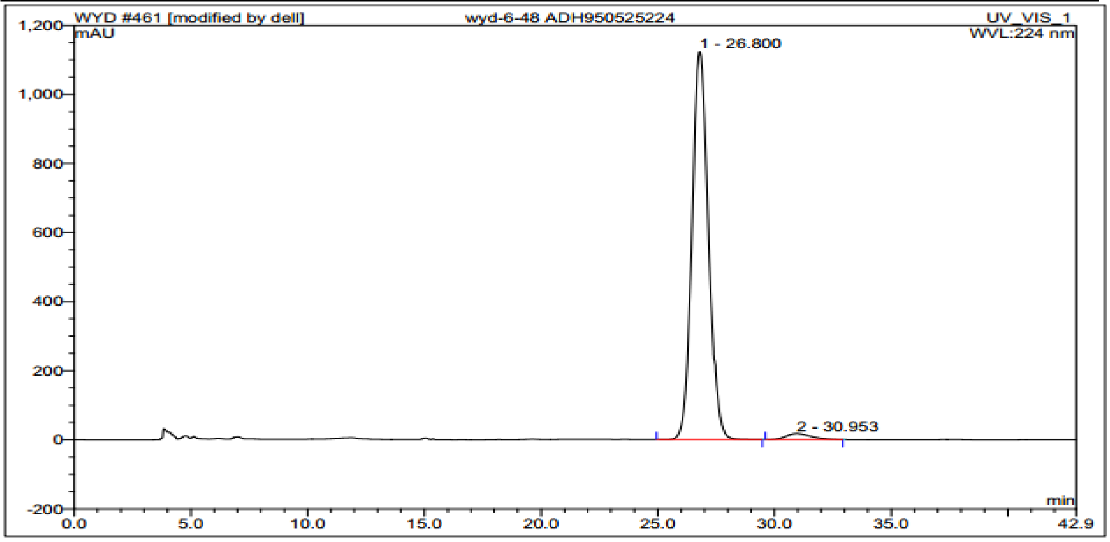

Peak #	Time (min)	Height (mAU)	Area (mAU*min)	Area (%)
1	26.80	1123.155	917.178	97.82
2	30.95	16.694	20.439	2.18

16. ***N*-(4-bromophenyl)-4-methyl-*N*-((*Z*)-((2*R*,3*S*)-2-(1-methyl-1H-indol-3-yl)-3-phenylcyclobutylidene)methyl)benzenesulfonamide (3p).**

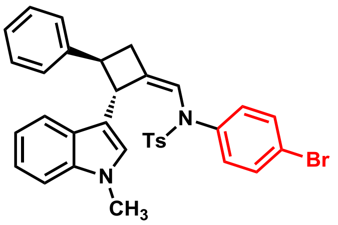

Isolated in 99% yield as white solid.

^{1}H NMR (400 MHz, $CDCl_3$) δ 7.36 (t, *J* = 7.6 Hz, 3 H), 7.28-7.14 (m, 9 H), 7.11 (t, *J* = 7.6 Hz, 1 H), 7.01 (t, *J* = 7.2 Hz, 1 H), 6.86 (d, *J* = 8.4 Hz, 2 H), 6.57 (s, 1 H), 6.42 (s, 1 H), 6.19 (d, *J* = 8.4 Hz, 2 H), 3.68 (s, 3 H), 3.64-3.54 (m, 2 H), 3.28-3.15 (m, 1 H), 2.94-2.83 (m, 1 H), 2.38 (s, 3 H); **^{13}C NMR** (100 MHz, $CDCl_3$) δ 144.2, 143.7, 137.7, 136.7, 134.8, 130.7, 129.6, 129.4, 128.3, 128.2, 127.4, 126.6, 126.3, 126.1, 121.3, 120.4, 119.7, 119.5, 118.8, 114.1, 108.8, 48.2, 45.3, 33.3, 32.5, 21.5; **HRMS** (ESI) calculated for $C_{33}H_{29}BrN_2NaO_2S$ [M + Na$^+$]: 619.1025, found: 619.1017. **$[\alpha]_D^{20}$** = -124.4 (c = 1.0, $CHCl_3$), **HPLC conditions**: with a Chiralpak OD-H column (95: 05 hexane: 2-propanol, 0.8 mL/min, 224 nm); tr (minor) = 17.02 min, tr (major) = 14.48 min, 96% ee.

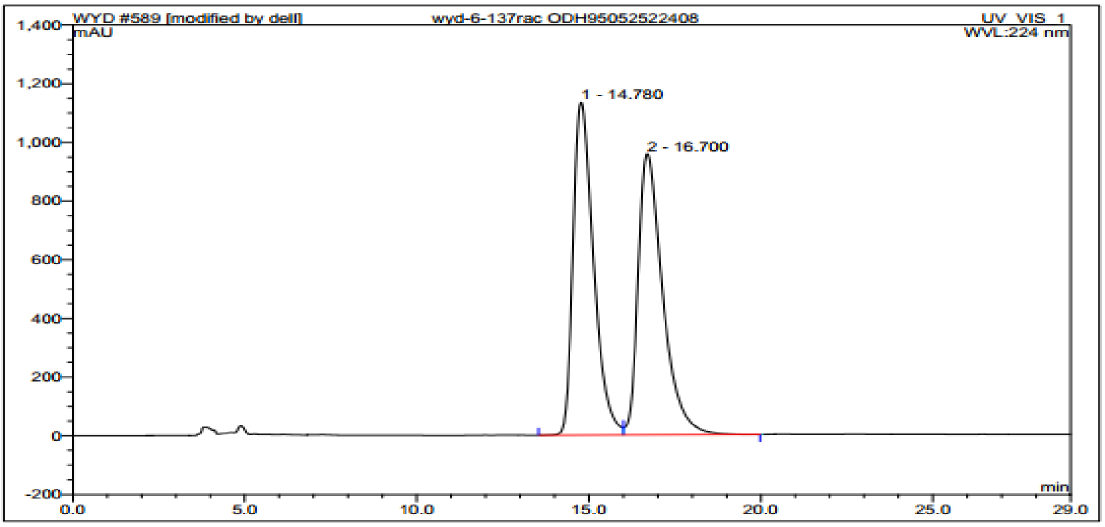

Peak #	Time(min)	Height (mAU)	Area (mAU*min)	Area (%)
1	14.78	1135.296	780.530	49.62
2	16.70	958.084	792.606	50.38

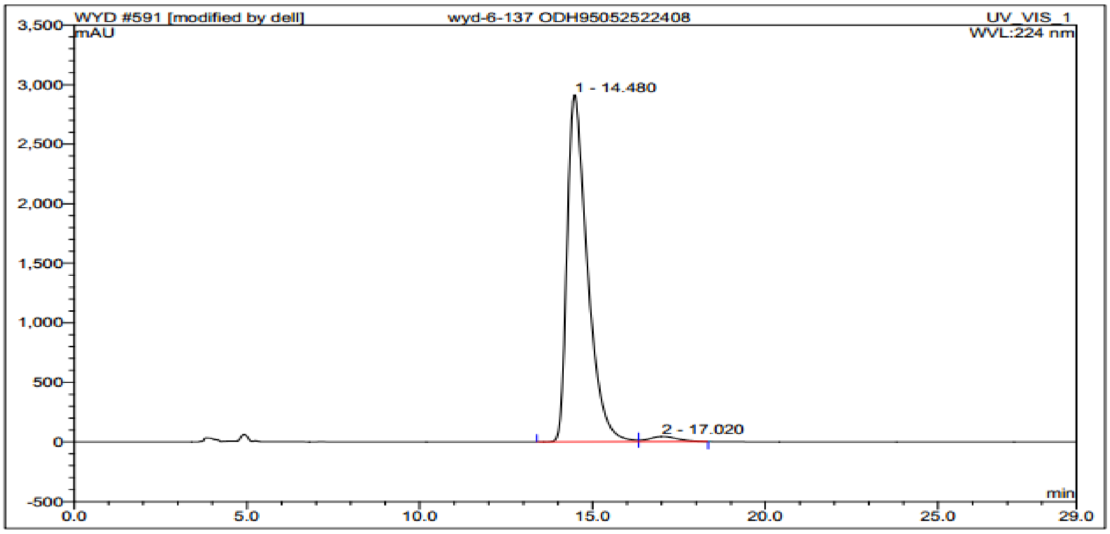

Peak #	Time(min)	Height (mAU)	Area (mAU*min)	Area (%)
1	14.48	2911.303	1985.241	98.02
2	17.02	42.653	40.137	1.98

17. **4-methyl-*N*-((*Z*)-((2*R*,3*S*)-2-(1-methyl-1H-indol-3-yl)-3-propylcyclobutylidene)methyl)-N-phenylbenzenesulfonamide.**

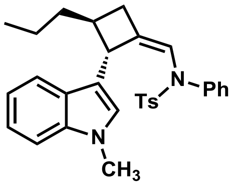

Isolated in 95% yield as colorless liquid.

^{1}H NMR (400 MHz, $CDCl_3$) δ 7.38 (d, *J* = 8.4 Hz, 2H), 7.28 (d, *J* = 7.6 Hz, 1H), 7.25-7.11 (m, 4H), 6.99-6.91 (m, 2H), 6.82 (t, *J* = 7.6 Hz, 2H), 6.53 (s, 1H), 6.45-6.38 (m, 3H), 3.67 (s, 3H), 3.14-3.07 (m, 1H), 2.98-2.84 (m, 1H), 2.38 (s, 3H), 2.29-2.18 (m, 2H), 1.55-1.44 (m, 1H), 1.42-1.33 (m, 1H), 1.23-1.10 (m, 2H), 0.77 (t, *J* = 7.6 Hz, 3H); **^{13}C NMR** (100 MHz, $CDCl_3$) δ 143.3, 139.1, 136.8, 135.3, 132.2, 129.3, 128.2, 127.7, 127.5, 126.8, 126.4, 126.2, 121.0, 119.9, 119.3, 118.3, 115.3, 108.7, 45.1, 41.3, 38.5, 32.6, 32.5, 21.5, 20.3, 14.1.; **MS** (EI): m/z (%): 484 (M^+, 1.63), 329 (100), **HRMS** (EI) calculated for [$C_{30}H_{32}N_2O_2S$]: 484.2185, found: 484.2180. **$[\alpha]_D^{20}$** = -84.7 (c = 0.5, $CHCl_3$), **HPLC conditions**: with a Chiralpak AD-H column (90: 10 hexane: 2-propanol, 0.8 mL/min, 224 nm); tr (minor) = 9.75 min, tr (major) = 10.67 min, 82% ee.

Chromatogram and Results

Injection Details

Injection Name:	wyd-7-41 ADH90100825	*Run Time (min):*	19.62
Vial Number:	RA1	*Injection Volume:*	20.00
Injection Type:	Unknown	*Channel:*	UV_VIS_1
Calibration Level:		*Wavelength:*	210.0
Instrument Method:	50min-1090-25-224254	*Bandwidth:*	4
Processing Method:	wyd-1	*Dilution Factor:*	1.0000
Injection Date/Time:	22/12月/14 10:15	*Sample Weight:*	1.0000

Chromatogram

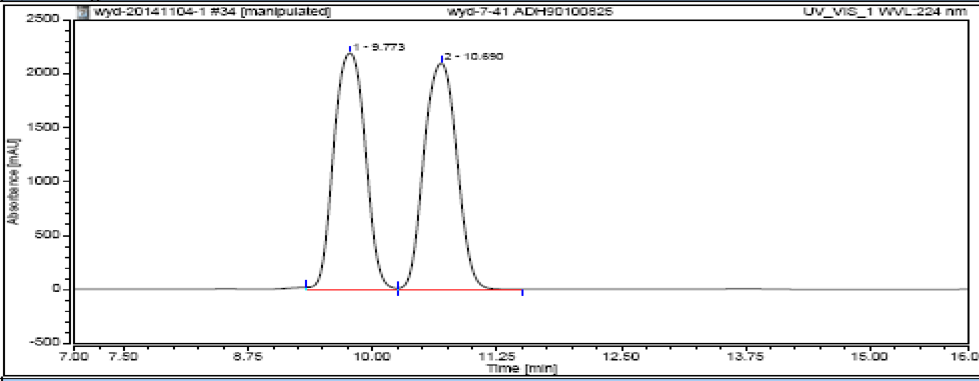

Integration Results

No.	Peak Name	Retention Time min	Area mAU*min	Height mAU	Relative Area %	Relative Height %	Amount n.a.
1		9.773	803.623	2191.699	50.00	51.10	n.a.
2		10.690	803.539	2097.397	50.00	48.90	n.a.
Total:			1607.163	4289.096	100.00	100.00	

Chromatogram and Results

Injection Details

Injection Name:	wyd-7-44-3 ADH90100825	*Run Time (min):*	20.00
Vial Number:	RB3	*Injection Volume:*	20.00
Injection Type:	Unknown	*Channel:*	UV_VIS_1
Calibration Level:		*Wavelength:*	210.0
Instrument Method:	20min-1090-25-08-210254	*Bandwidth:*	4
Processing Method:	wyd-1	*Dilution Factor:*	1.0000
Injection Date/Time:	24/12月/14 20:47	*Sample Weight:*	1.0000

Chromatogram

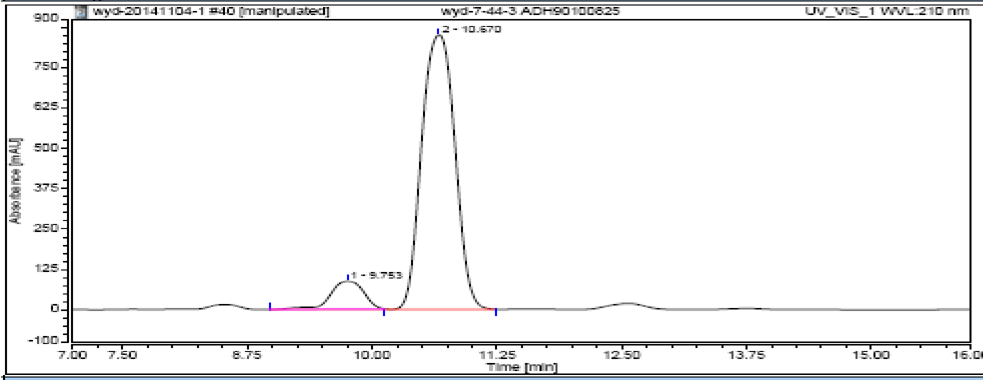

Integration Results

No.	Peak Name	Retention Time min	Area mAU*min	Height mAU	Relative Area %	Relative Height %	Amount n.a.
1		9.753	33.126	85.844	9.15	9.16	n.a.
2		10.670	329.043	851.072	90.85	90.84	n.a.
Total:			362.170	936.916	100.00	100.00	

18. 3-((Z)-((2R,3S)-2-(1-methyl-1H-indol-3-yl)-3-phenylcyclobutylidene)methyl)oxazolidin-2-one.

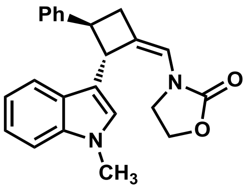

Isolated in 95% yield as white solid.

^{1}H NMR (400 MHz, $CDCl_3$) δ 7.48 (d, *J* = 8.0 Hz, 1H), 7.37-7.29 (m, 5H), 7.29-7.19 (m, 2H), 7.07 (t, *J* = 7.6 Hz, 1H), 6.97 (s, 1H), 6.56-6.52 (m, 1H), 4.47-4.40 (m, 1H), 4.08-3.98 (m, 1H), 3.94-3.84 (m, 1H), 3.77 (s, 3H), 3.56-3.28 (m, 4H), 3.01-2.92 (m, 1H); **^{13}C NMR** (100 MHz, $CDCl_3$) δ 156.3, 144.8, 137.4, 128.5, 126.5, 126.4, 126.2, 126.0, 123.0, 121.9, 119.4, 119.1, 117.6, 117.0, 109.4, 62.1, 47.6, 46.3, 44.2, 33.6, 32.8; **MS** (EI): m/z (%): 358 (M^+, 41.08), 119 (100); **HRMS** (EI) calculated for [$C_{23}H_{22}N_2O_2$]: 358.1681, found: 358.1679. **$[\alpha]_D^{20}$** = -19.6 (c = 0.5, $CHCl_3$), **HPLC conditions**: with a Chiralpak AD-H column (95: 05 hexane: 2-propanol, 1.0 mL/min, 224 nm); tr (minor) = 32.87 min, tr (major) = 35.86 min, 22% ee.

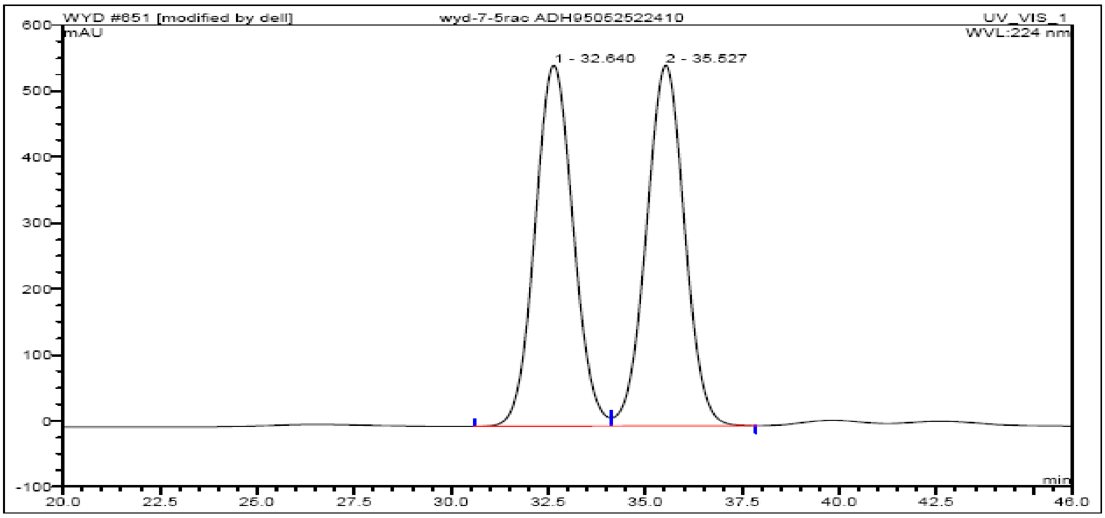

Peak #	Time (min)	Height (mAU)	Area (mAU*min)	Area (%)
1	32.64	546.998	621.672	49.87
2	35.53	546.718	624.806	50.13

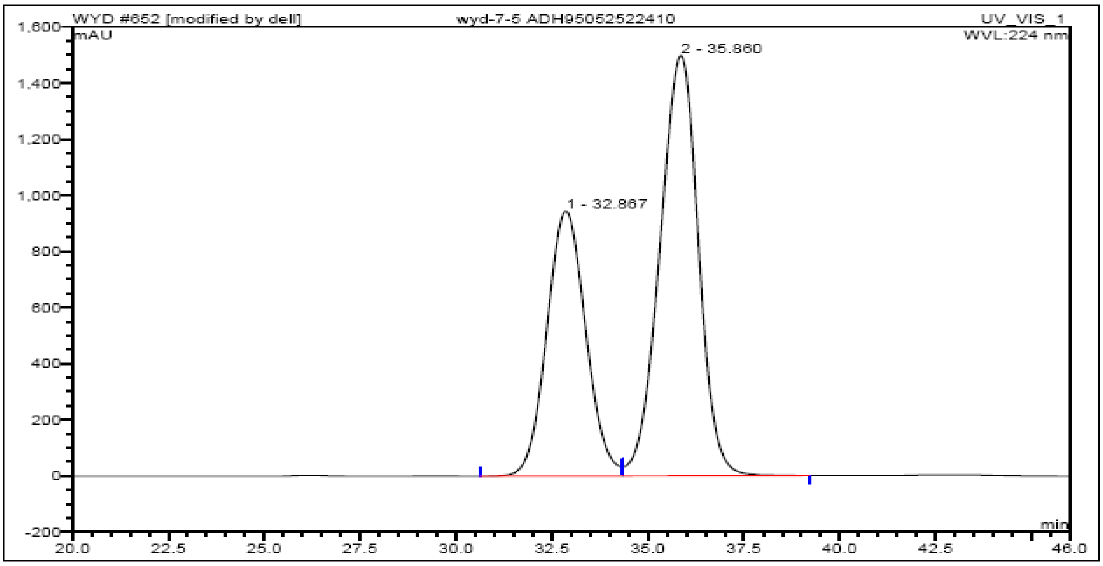

Peak #	Time (min)	Height (mAU)	Area (mAU*min)	Area (%)
1	32.87	943.286	1112.533	38.97
2	35.86	1496.715	1742.147	61.03

19. (3*R*,9a*S*,*Z*)-ethyl 2-((4-methyl-*N*-phenylphenylsulfonamido)methylene)-3-phenyl-2,3-dihydro-1H-carbazole-9(9aH)-carboxylate (4a).

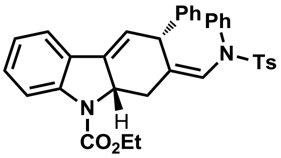

Isolated in 95% yield with 5.3:1 *Z/E* as white solid.

***Z*-isomer, ^{1}H NMR** (300 MHz, $CDCl_3$) δ 7.79 (s, 1 H), 7.43 (d, *J* = 8.1 Hz, 2 H), 7.35-7.10 (m, 12 H), 7.10-7.02 (m, 2 H), 6.99 (t, *J* = 7.5 Hz, 1 H), 6.36 (s, 1 H), 5.79 (t, *J* = 2.7 Hz, 1 H); 4.72-4.62 (m, 1 H), 4.57 (t, *J* = 2.7 Hz, 1 H), 4.50-4.30 (m, 2 H), 3.46 (d, *J* = 7.5 Hz, 1 H), 2.44 (s, 3 H), 2.54-2.37 (m, 1 H), 1.46 (t, *J* = 7.2 Hz, 3 H); **^{13}C NMR** (75 MHz, $CDCl_3$) δ 153.8, 143.9, 142.3, 140.7, 137.3, 136.3, 133.9, 129.4, 129.3, 128.8, 128.4, 127.9, 127.8, 127.7, 126.9, 126.7, 126.5, 125.0, 123.0, 120.1, 119.5, 115.5, 62.3, 61.9, 42.3, 34.7, 21.5, 14.6; **HRMS** (ESI) calculated for $C_{35}H_{32}N_2NaO_4S$ $[M + Na^+]$: 599.1975, found: 599.1964. $[\alpha]_D^{20}$ = 294.7 (0.5, $CHCl_3$), **HPLC conditions**: with a Chiralpak AS-H column (95: 05 hexane: 2-propanol, 1.0 mL/min, 224 nm); tr (minor) = 20.38 min, tr (major) = 15.91 min, 95% ee.

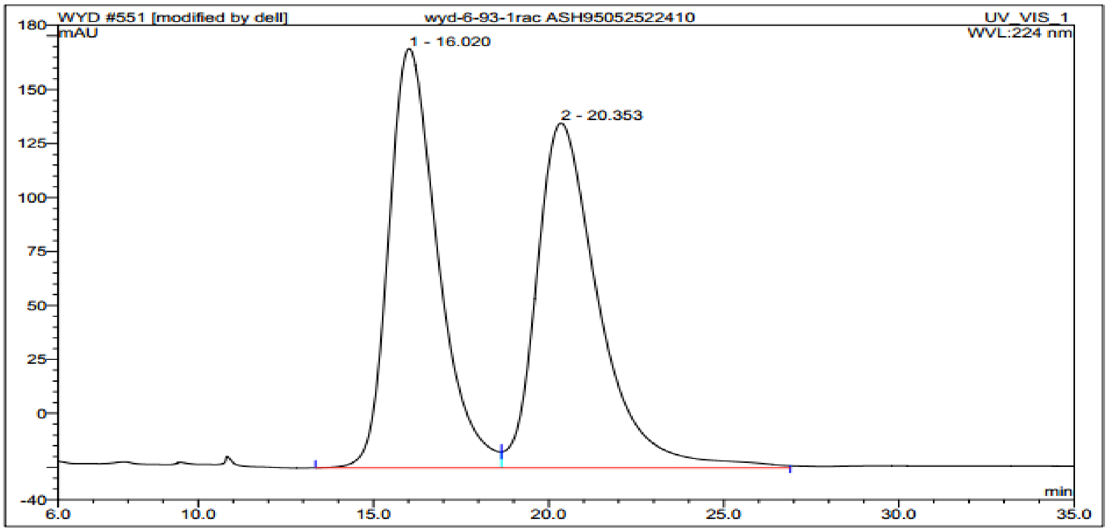

Peak #	Time (min)	Height (mAU)	Area (mAU*min)	Area (%)
1	16.02	194.176	314.303	49.01
2	20.35	159.758	326.980	50.99

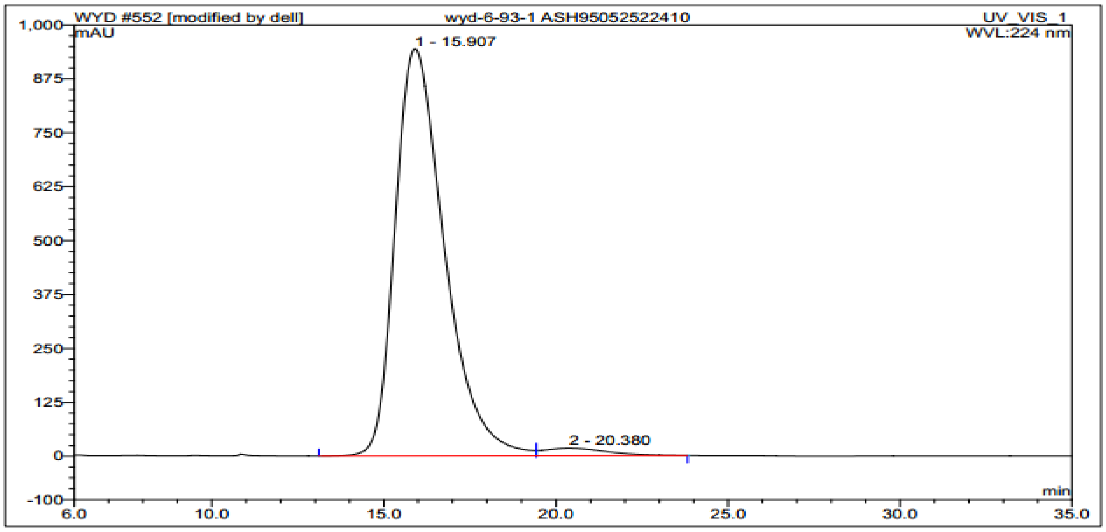

Peak #	Time (min)	Height (mAU)	Area (mAU*min)	Area (%)
1	15.91	943.998	1569.568	97.73
2	20.38	17.298	36.533	2.27

20. (3*R*,9a*S*,*E*)-ethyl 2-((4-m ethyl-*N*-phenylphenylsulfonamido)methylene)-3-phenyl-2,3-dihydro-1H-carb azole-9(9aH)-carboxylate.

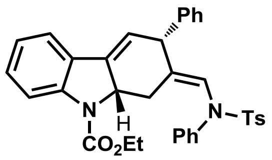

***E*-isomer,** ^{1}**H NMR** (400 MHz, $CDCl_3$) δ 7.92 (s, 1 H), 7.31-7.38 (m, 3 H), 7.18-7.28 (m, 9 H), 7.10 (d, *J* = 8.0 Hz, 2 H), 7.02 (d, *J* = 7.6 Hz, 2 H), 6.98 (d, *J* = 7.6 Hz, 1 H), 6.06 (s, 1 H), 5.97 (t, *J* = 2.8 Hz, 1 H), 4.50-4.60 (m, 1 H), 4.26-4.43 (m, 2 H), 4.22 (s, 1 H), 3.83-3.93 (m, 1 H), 2.36 (s, 3 H), 1.89 (t, *J* = 7.6 Hz, 1 H), 1.42 (t, *J* = 7.2 Hz, 3 H); ^{13}**C NMR** (100 MHz, $CDCl_3$) 153.4, 144.8, 144.3, 143.6, 141.3, 139.5, 138.3, 134.4, 129.6, 129.3, 128.9, 128.7, 127.9, 127.7, 127.2, 127.0, 126.8, 126.5, 122.9, 112.0, 117.9, 115.6, 61.8, 60.6, 46.5, 30.4, 21.5, 14.7; **HRMS** (ESI) calculated for $C_{35}H_{32}N_2NaO_4S$ [M + Na^+]: 599.1975, found: 599.1879. $[\alpha]_D^{20}$ = 128.0 (c = 0.5, $CHCl_3$), **HPLC conditions**: with a Chiralpak OD-H column (90: 10 hexane: 2-propanol, 0.8 mL/min, 224 nm); tr (minor) = 22.69 min, tr (major) = 37.89 min, 92% ee.

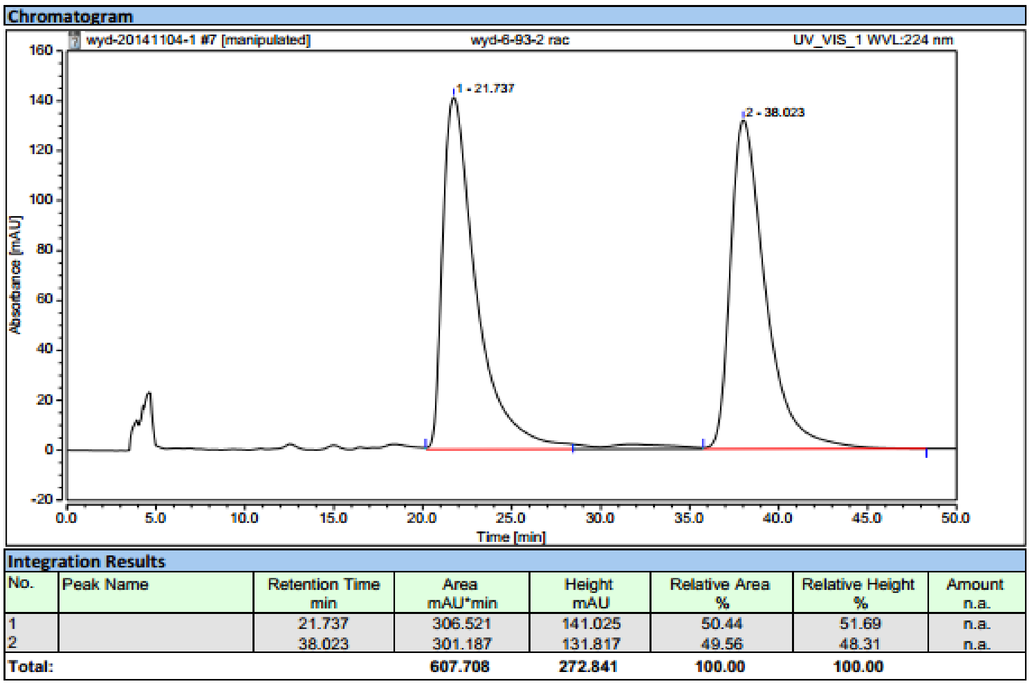

Integration Results

No.	Peak Name	Retention Time min	Area mAU*min	Height mAU	Relative Area %	Relative Height %	Amount n.a.
1		21.737	306.521	141.025	50.44	51.69	n.a.
2		38.023	301.187	131.817	49.56	48.31	n.a.
**Total:**			**607.708**	**272.841**	**100.00**	**100.00**	

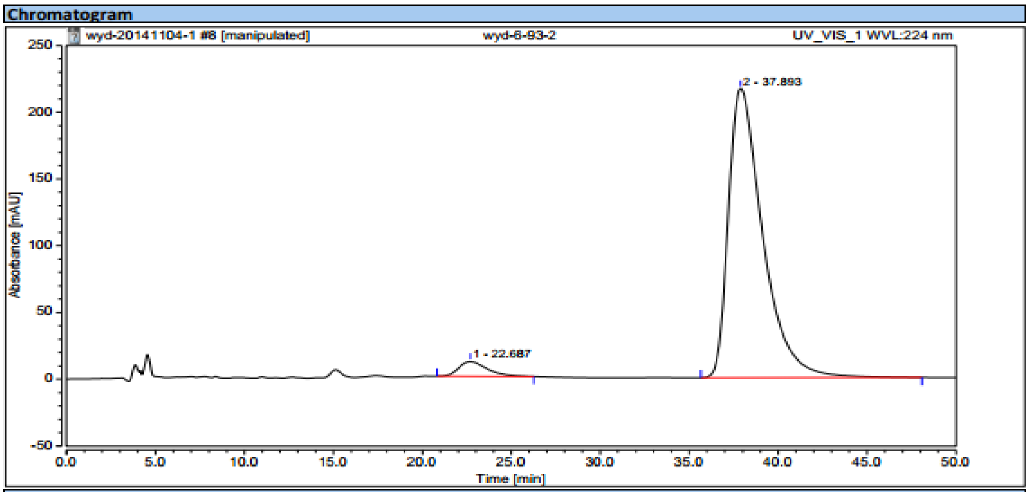

Integration Results

No.	Peak Name	Retention Time min	Area mAU*min	Height mAU	Relative Area %	Relative Height %	Amount n.a.
1		22.687	20.849	11.189	4.13	4.91	n.a.
2		37.893	484.083	216.616	95.87	95.09	n.a.
**Total:**			**504.932**	**227.805**	**100.00**	**100.00**	

21. 4-methyl-*N*-phenyl-*N*-((*Z*)-((3*R*,9a*S*)-3-phenyl-9-tosyl-9,9a-dihydro-1H-carbazol-2(3H)-ylidene)methyl) benzenesulfonamide (4b).

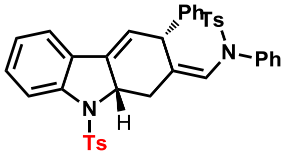

Isolated in 67% yield with 7.3:1 *Z*/*E* as white solid.

***Z*-isomer,**^{1}**H NMR** (400 MHz, $CDCl_3$) δ 7.74 (t, *J* = 8.0 Hz, 3 H), 7.42 (d, *J* = 7.6 Hz, 2 H), 7.32-7.17 (m, 7 H), 7.17-7.09 (m, 5 H), 7.08-7.02 (m, 2 H), 7.02-6.93 (m, 3 H), 6.33 (s, 1 H), 5.74 (t, *J* = 3.2 Hz, 1 H), 4.66 (s, 1 H), 4.44-4.28 (m, 1 H), 3.40 (t, *J* = 4.8 Hz, 1 H), 2.70 (t, *J* = 11.6 Hz, 1 H), 2.44 (s, 3 H), 2.39 (s, 3 H); ^{13}**C NMR** (100 MHz, $CDCl_3$) δ 144.6, 144.1, 143.9, 141.9, 140.5, 138.0, 135.3, 133.9, 133.1, 129.8, 129.5, 128.7, 128.4, 128.3, 127.9, 127.7, 127.7, 127.0, 126.6, 126.5, 125.0, 124.2, 120.6, 120.1, 115.4, 64.3, 42.3, 35.9, 21.60, 21.57. **HRMS** (ESI) calculated for $C_{39}H_{34}N_2NaO_4S_2$ [M + Na$^+$]: 681.1852, found: 681.1754. **[α]$_D^{20}$** = 347.3 (c = 0.5, $CHCl_3$), **HPLC conditions**: with a Chiralpak OD-H column (90: 10 hexane: 2-propanol, 0.8 mL/min, 224 nm); tr (minor) = 11.37 min, tr (major) = 15.33 min, 91% ee.

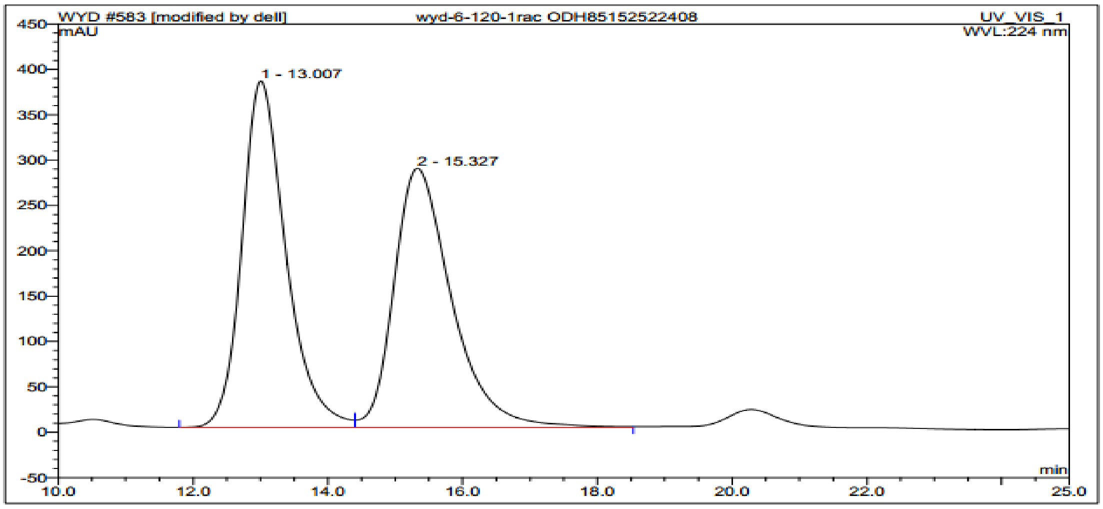

Peak #	Time(min)	Height (mAU)	Area (mAU*min)	Area (%)
1	13.01	381.640	288.159	50.57
2	15.33	285.346	281.712	49.43

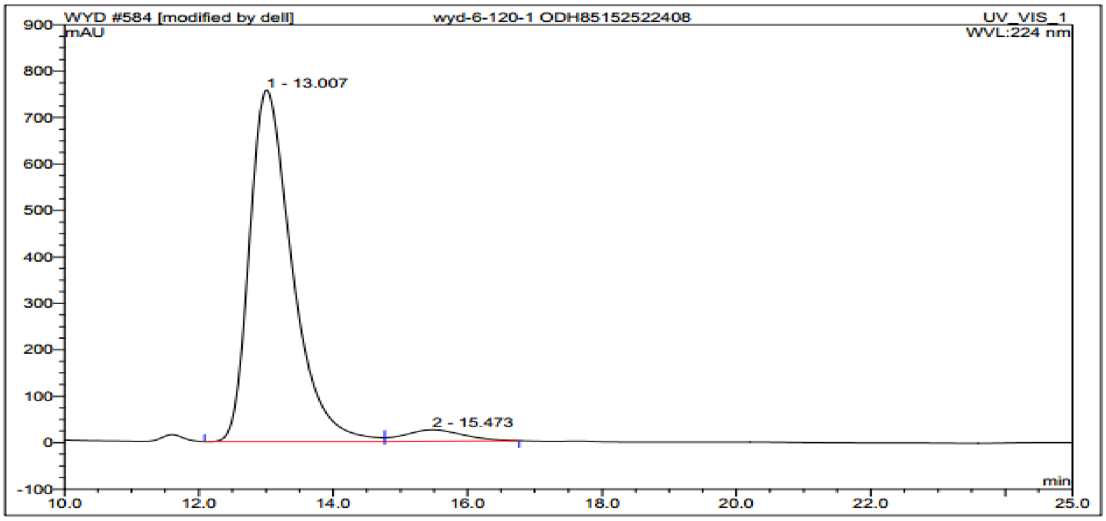

Peak #	Time(min)	Height (mAU)	Area (mAU*min)	Area (%)
1	13.01	756.796	556.168	95.72
2	15.47	24.592	24.882	4.28

22. ***N*-((*Z*)-((3*R*,9a*S*)-9-acetyl-3-phenyl-9,9a-dihydro-1H-carbazol-2(3H)-ylidene)methyl)-4-methyl-*N*-phenylbenzenesulfonamide (4c).**

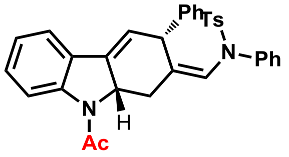

Isolated in 67% yield with 7.3:1 *Z*/*E* as white solid.

***Z*-isomer, ^{1}H NMR** (400 MHz, $CDCl_3$) δ 7.50-7.28 (m, 4 H), 7.24-7.05 (m, 11 H), 7.05-6.87 (m, 3 H), 6.36 (s, 1 H), 5.80 (s, 1 H), 4.72 (s, 1 H), 4.54 (s, 1 H), 3.62 (s, 1 H), 2.70-2.10 (m, 1 H), 2.45 (s, 3 H), 2.40 (s, 3 H); **^{13}C NMR** (100 MHz, $CDCl_3$) 169.5, 143.9, 142.3, 140.6, 136.0, 133.8, 129.4, 129.2, 128.7, 128.4, 127.8, 127.7, 127.0, 126.8, 126.5, 125.3, 123.7, 119.8, 62.6, 42.3, 34.1, 25.6, 21.5. **HRMS** (ESI) calculated for $C_{34}H_{30}N_2NaO_3S$ [M + Na^+]: 681.1852, found: 681.1754.**HRMS** (ESI) calculated for $C_{34}H_{30}N_2NaO_3S$ [M + Na^+]: 569.1869, found: 569.1872. **$[\alpha]_D^{20}$** = 304.5 (c = 0.5, $CHCl_3$), **HPLC conditions**: with a Chiralpak AD-H column (70: 30 hexane: 2-propanol, 0.8 mL/min, 224 nm); tr (minor) = 19.29 min, tr (major) = 15.71 min, 94% ee.

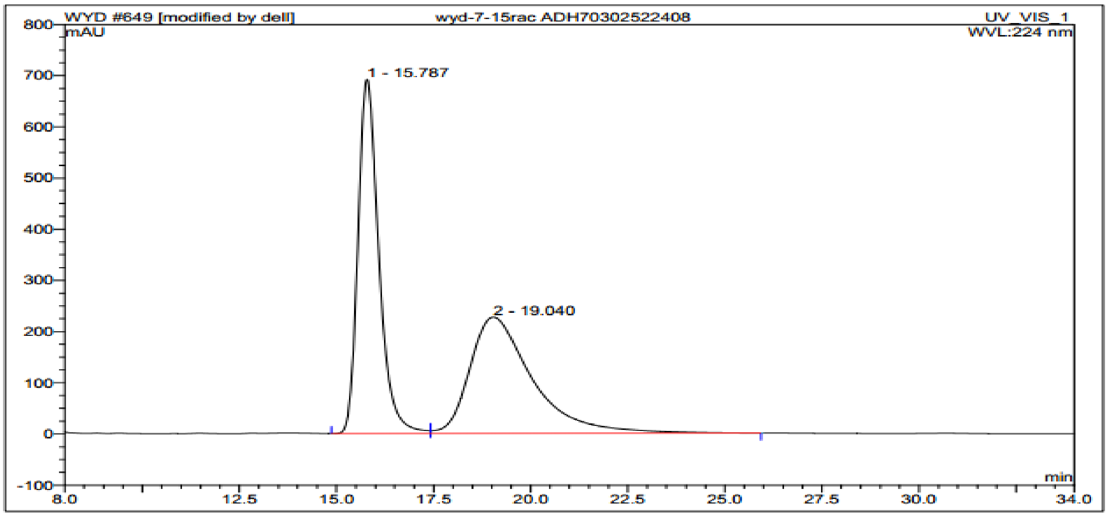

Peak #	Time(min)	Height (mAU)	Area (mAU*min)	Area (%)
1	15.79	692.227	431.588	50.46
2	19.04	226.937	423.791	49.54

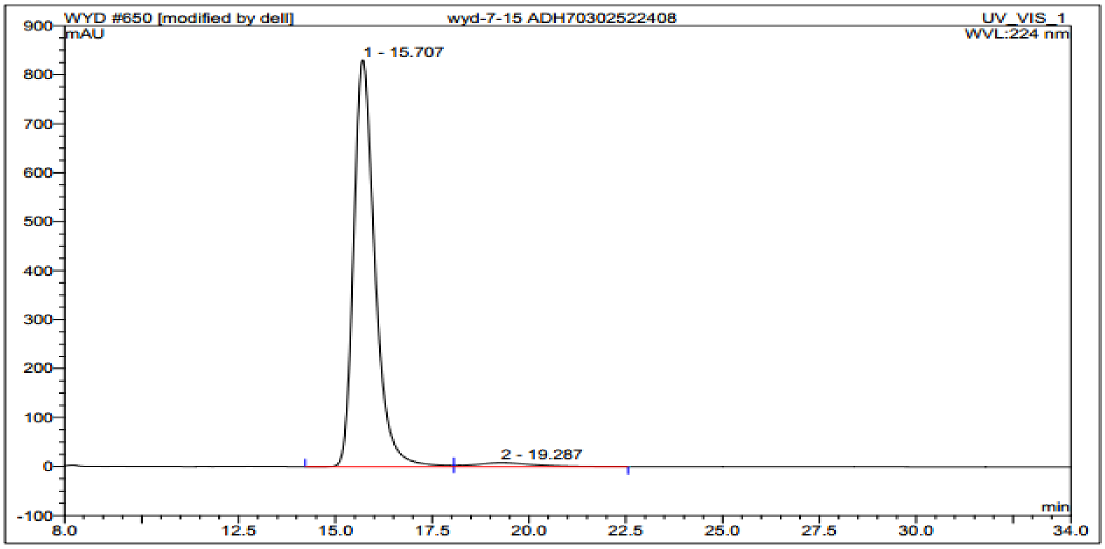

Peak #	Time(min)	Height (mAU)	Area (mAU*min)	Area (%)
1	15.71	830.539	529.470	97.37
2	19.29	7.911	14.305	2.63

23. (3*R*,9a*S*,*Z*)-ethyl 3-(4-bromophenyl)-2-((4-methyl-*N*-phenylphenylsulfonamido)methylene)-2,3-d ihydro -1H-carbazole-9(9aH)-carboxylate (4d).

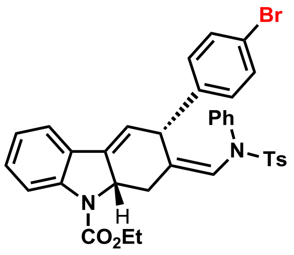

Isolated in 98% yield with 3.5:1 *Z*/*E* as white solid.

***Z*-isomer, ^{1}H NMR** (400 MHz, $CDCl_3$) δ 7.75 (s, 1 H), 7.39 (d, *J* = 8.0 Hz, 2 H), 7.29-7.18 (m, 9 H), 7.01-6.94 (m, 5 H), 6.35 (s, 1 H), 5.69 (t, *J* = 2.8 Hz, 1 H), 4.64-4.61 (m, 1 H), 4.49-4.45 (m, 1 H), 4.42-4.33 (m, 2 H), 3.43 (s, 1 H), 2.42 (s, 3 H), 2.43-2.33 (m, 1 H), 1.44 (t, *J* = 7.2 Hz, 3 H); **^{13}C NMR** (100 MHz, $CDCl_3$) 153.8, 144.0, 141.4, 140.6, 136.8, 136.6, 133.9, 131.4, 129.5, 129.42, 129.40, 128.8, 127.9, 127.5, 127.0, 126.6, 125.5, 123.1, 120.3, 120.1, 118.6, 115.5, 62.1, 62.0, 41.7, 34.7, 21.6, 14.6; **MS** (EI): m/z (%): 499 (M^+, 20.38), 501 (M^++2, 13.05), 217 (100), **HRMS** (EI) calculated for [$C_{35}H_{31}BrN_2O_4S$ – $C_7H_7O_2S$]: 499.1021, found: 499.1019. **$[\alpha]_D^{20}$** = 290.7 (c = 0.5, $CHCl_3$), **HPLC conditions**: with a Chiralpak AD-H column (70: 30 hexane: 2-propanol, 0.8 mL/min, 224 nm); tr (minor) = 8.81 min, tr (major) = 20.71 min, 97% ee.

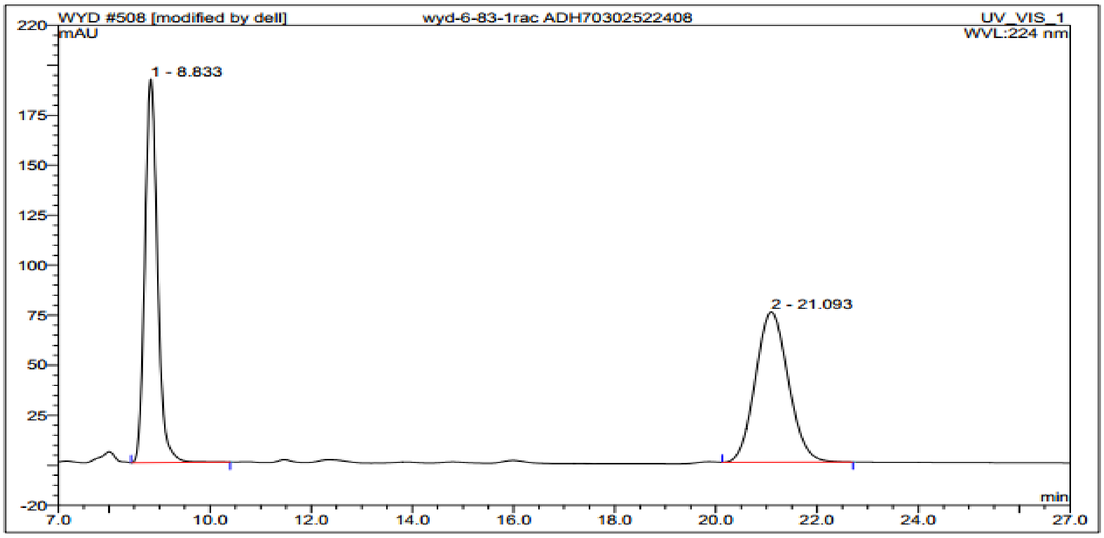

Peak #	Time(min)	Height (mAU)	Area (mAU*min)	Area (%)
1	8.83	191.823	55.385	49.53
2	21.09	74.992	56.434	50.47

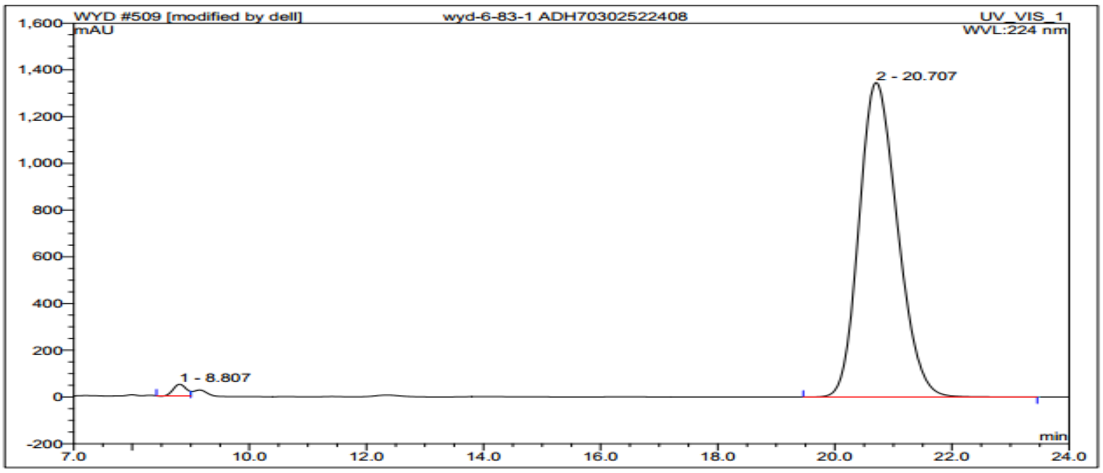

Peak #	Time(min)	Height (mAU)	Area (mAU*min)	Area (%)
1	8.81	49.393	12.550	1.21
2	20.71	1346.189	1021.607	98.79

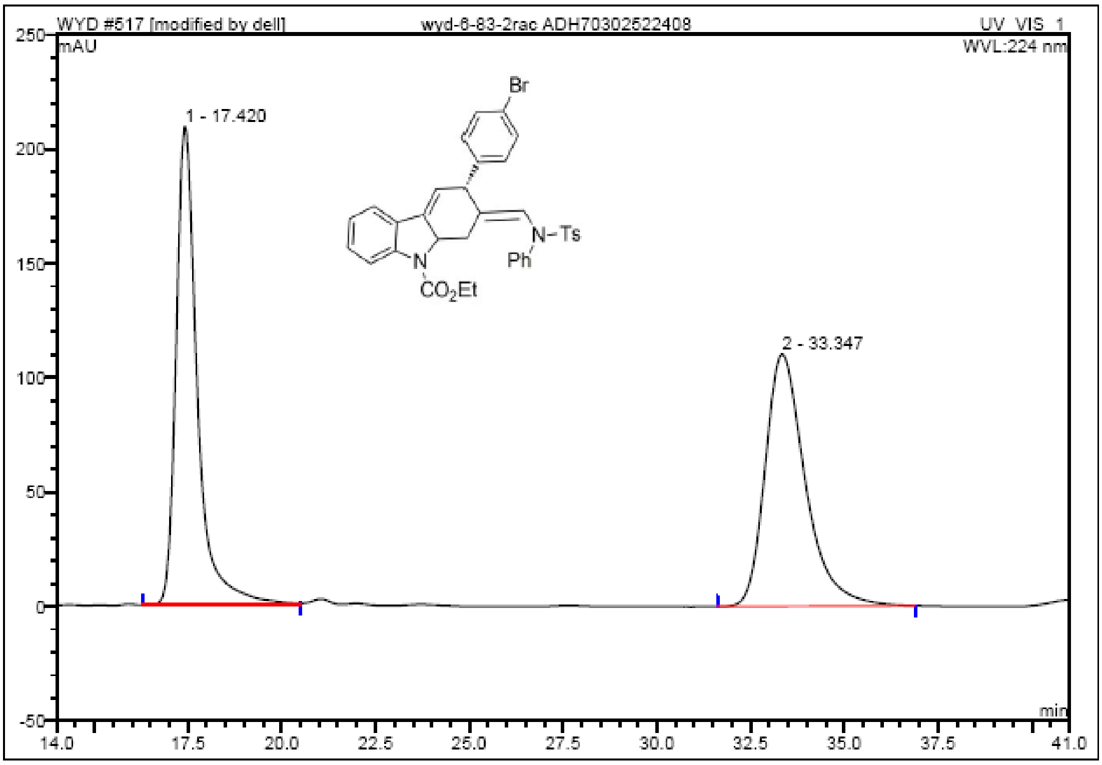

Peak #	Time(min)	Height (mAU)	Area (mAU*min)	Area (%)
1	17.42	209.215	138.118	50.18
2	33.35	110.301	137.153	49.82

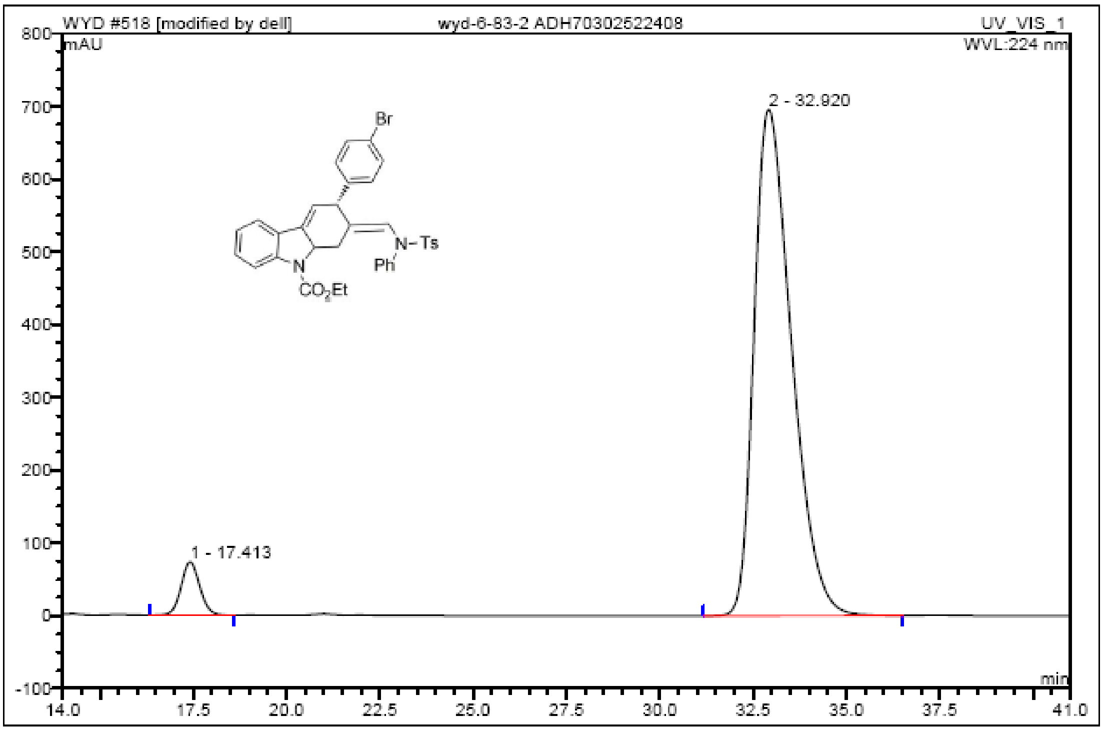

Peak #	Time(min)	Height (mAU)	Area (mAU*min)	Area (%)
1	17.41	73.061	42.309	4.97
2	32.92	695.547	808.987	95.03

24. (3*R*,9a*S*,*Z*)-ethyl 3-(4-chlorophenyl)-2-((4-methyl-*N*-phenylphenylsulfonamido)methylene)-2,3-dihydro-1H-carbazole-9(9aH)-carboxylate(4e).

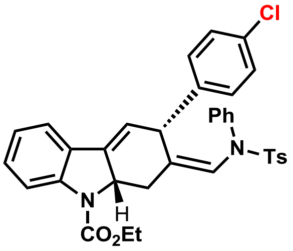

Isolated in 95% yield with 3.4:1 *Z*/*E* as white solid.

***Z*-isomer, ^{1}H NMR** (400 MHz, $CDCl_3$) δ 7.75 (s, 1 H), 7.39 (d, *J* = 8.0 Hz, 2 H), 7.28 (d, *J* = 7.6 Hz, 1 H), 7.22-7.18 (m, 6 H), 7.14-7.09 (m, 2 H), 7.04-6.94 (m, 5 H), 6.35 (s, 1 H), 5.69 (t, *J* = 2.8 Hz, 1 H), 4.64-4.61 (m, 1 H), 4.51-4.46 (m, 1 H), 4.42-4.33 (m, 2 H), 3.43 (s, 1 H), 2.41 (s, 3 H), 2.43-2.33 (m, 1 H), 1.44 (t, *J* = 7.2 Hz, 3 H); **^{13}C NMR** (100 MHz, $CDCl_3$) 153.8, 144.0, 140.9, 140.6, 136.70, 136.65, 133.7, 132.2, 129.5, 129.4, 129.0, 128.8, 128.4, 127.8, 127.5, 127.0, 126.6, 125.4, 123.1, 120.1, 118.7, 115.5, 62.1, 62.0, 41.6, 34.7, 21.5, 14.6; **HRMS** (ESI) calculated for $C_{35}H_{31}ClN_2NaO_4S$ [M + Na$^+$]: 633.1585, found: 633.1554. **$[\alpha]_D^{20}$** = 285.8 (c = 0.5, $CHCl_3$), **HPLC conditions**: with a Chiralpak AD-H column (70: 30 hexane: 2-propanol, 0.8 mL/min, 224 nm); tr (minor) = 8.52 min, tr (major) = 18.11 min, 95% ee.

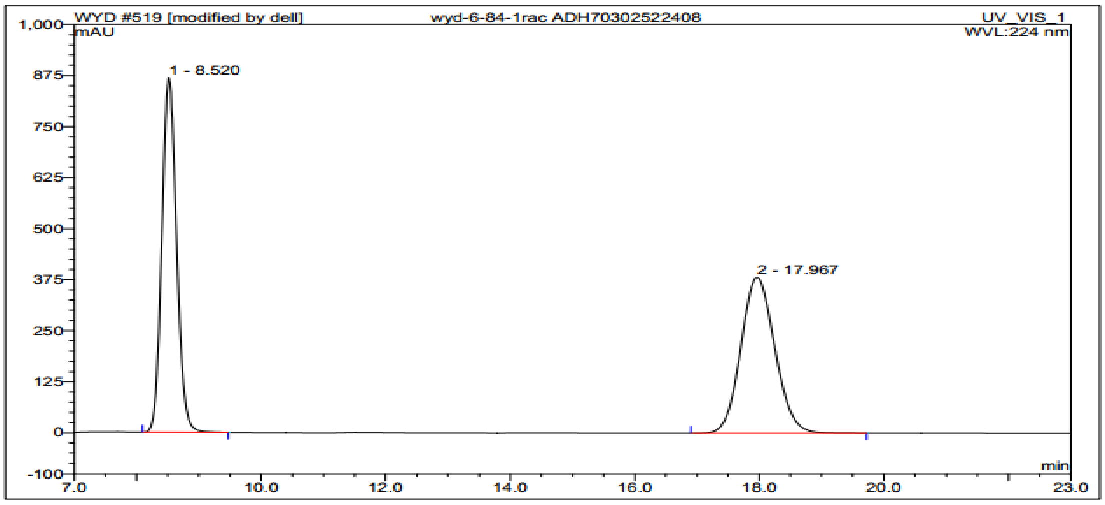

Peak #	Time(min)	Height (mAU)	Area (mAU*min)	Area (%)
1	8.52	867.743	237.902	49.72
2	17.97	381.096	240.608	50.28

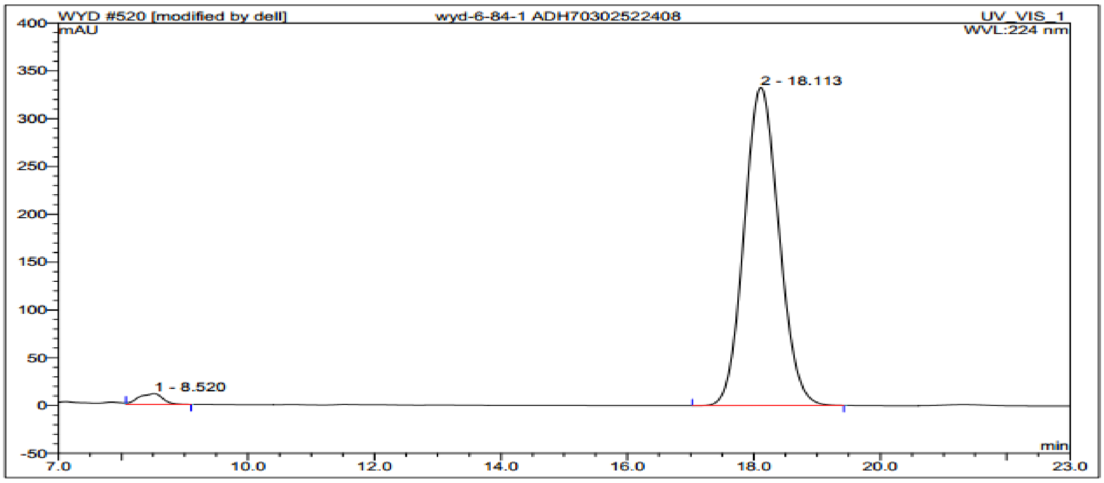

Peak #	Time(min)	Height (mAU)	Area (mAU*min)	Area (%)
1	8.52	11.089	4.916	2.27
2	18.11	332.733	211.883	97.73

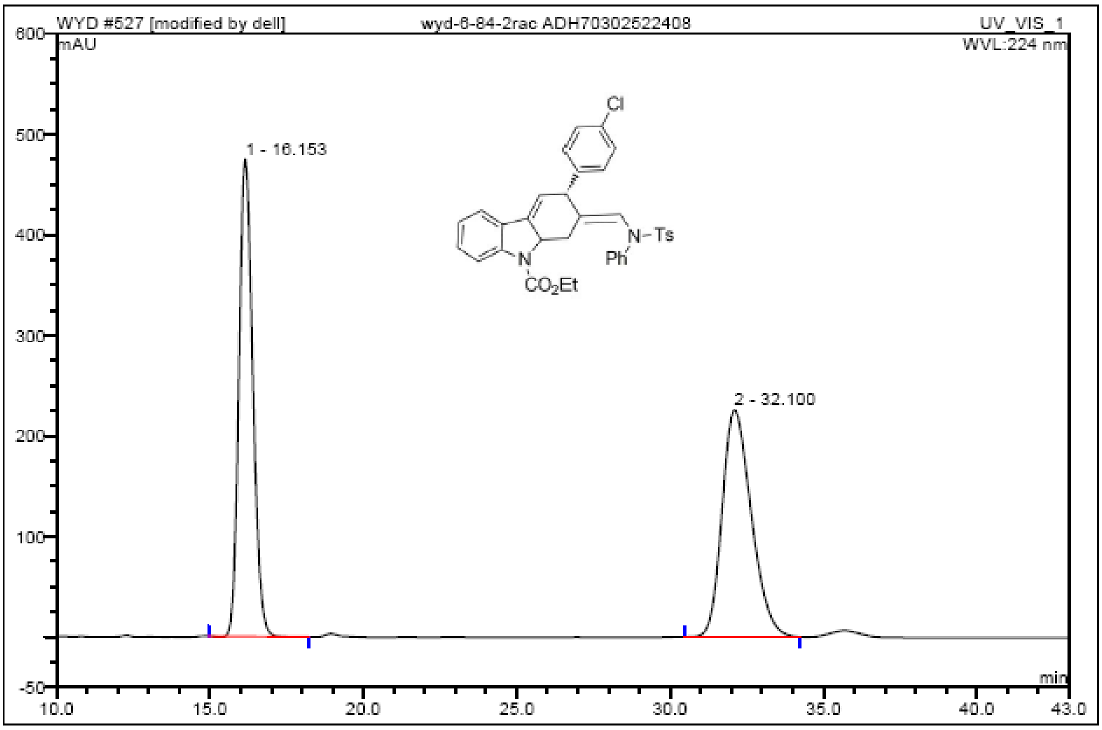

Peak<br>\#	Time<br>(min)	Height<br>(mAU)	Area<br>(mAU*min)	Area<br>(%)
1	16.15	474.297	247.939	49.73
2	32.10	225.382	250.592	50.27

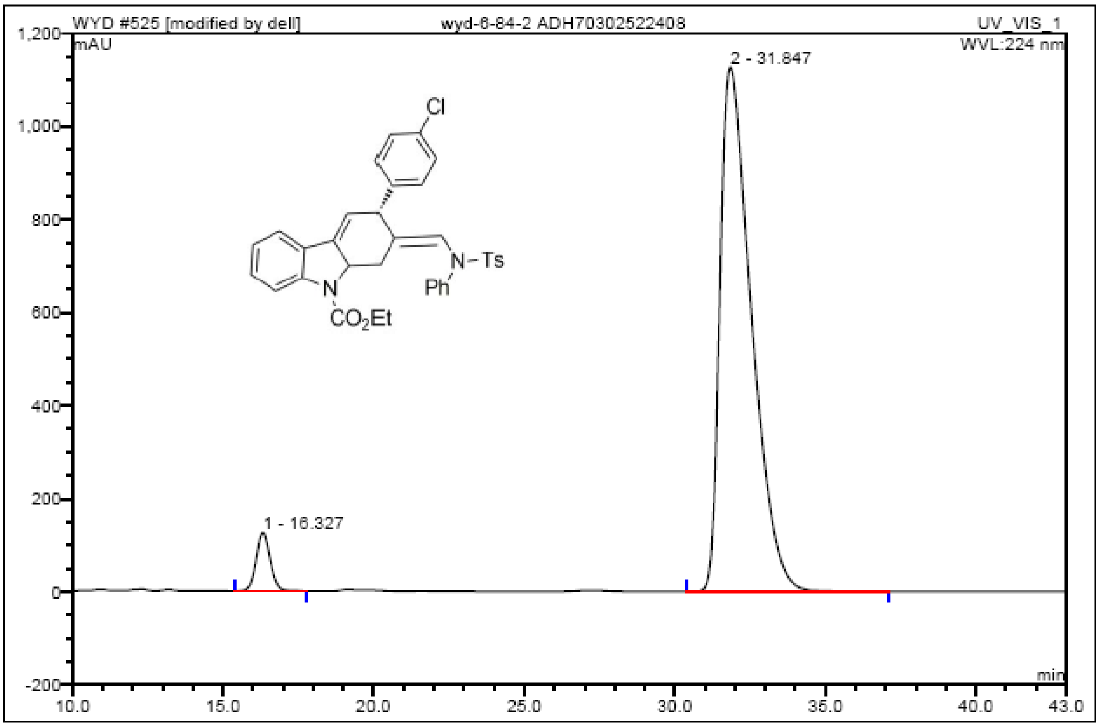

Peak #	Time(min)	Height (mAU)	Area (mAU*min)	Area (%)
1	16.33	125.277	68.243	4.78
2	31.85	1126.522	1358.770	95.22

25. (3*R*,9a*S*,*Z*)-ethyl 2-((4-methyl-*N*-phenylphenylsulfonamido)methylene)-3-(p-tolyl)-2,3-dihydro-1H-carbazole-9(9aH)-carboxylate. (4f)

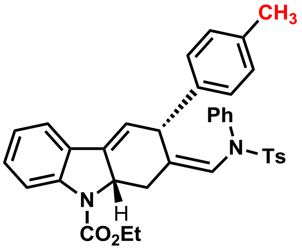

Isolated in 95% yield with 5.6:1 *Z*/*E* as white solid.

***Z*-isomer, ^{1}H NMR** (400 MHz, $CDCl_3$) δ 7.76 (s, 1 H), 7.41 (d, *J* = 8.0 Hz , 2 H), 7.28 (d, *J* = 7.6 Hz, 1 H), 7.24-7.17 (m, 6 H), 7.08-6.93 (m, 7 H), 6.31 (s, 1 H), 5.76 (t, *J* = 2.8 Hz, 1 H), 4.67-4.60 (m, 1 H), 4.52-4.48 (m, 1 H), 4.44-4.31 (m, 2 H), 3.40 (s, 1 H), 2.42 (s, 3 H), 2.47-2.39 (m, 1 H), 2.27 (s, 3 H), 1.44 (t, *J* = 7.2 Hz, 3 H); **^{13}C NMR** (100 MHz, $CDCl_3$) 153.8, 143.8, 140.8, 139.3, 137.6, 136.1, 136.0, 133.9, 129.3, 129.2, 129.0, 128.7, 127.8, 127.6, 126.9, 126.7, 124.7, 123.0, 120.0, 119.6, 115.4, 62.3, 61.9, 41.8, 34.5, 21.5, 20.9, 14.5; **HRMS** (ESI) calculated for $C_{36}H_{34}N_2NaO_4S$ [M + Na$^+$]: 613.2131, found: 613.2079. **$[\alpha]_D^{20}$** = 294.1 (c = 0.5, $CHCl_3$), **HPLC conditions**: with a Chiralpak AD-H column (70: 30 hexane: 2-propanol, 0.8 mL/min, 224 nm); tr (minor) = 7.79 min, tr (major) = 10.65 min, 96% ee.

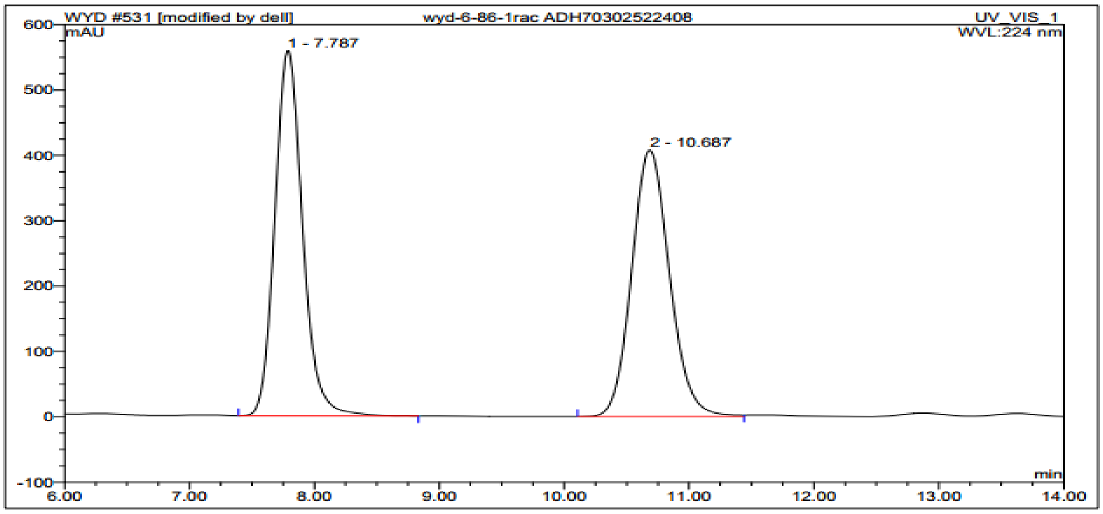

Peak #	Time(min)	Height (mAU)	Area (mAU*min)	Area (%)
1	7.79	558.602	142.985	49.91
2	10.69	407.748	143.482	50.01

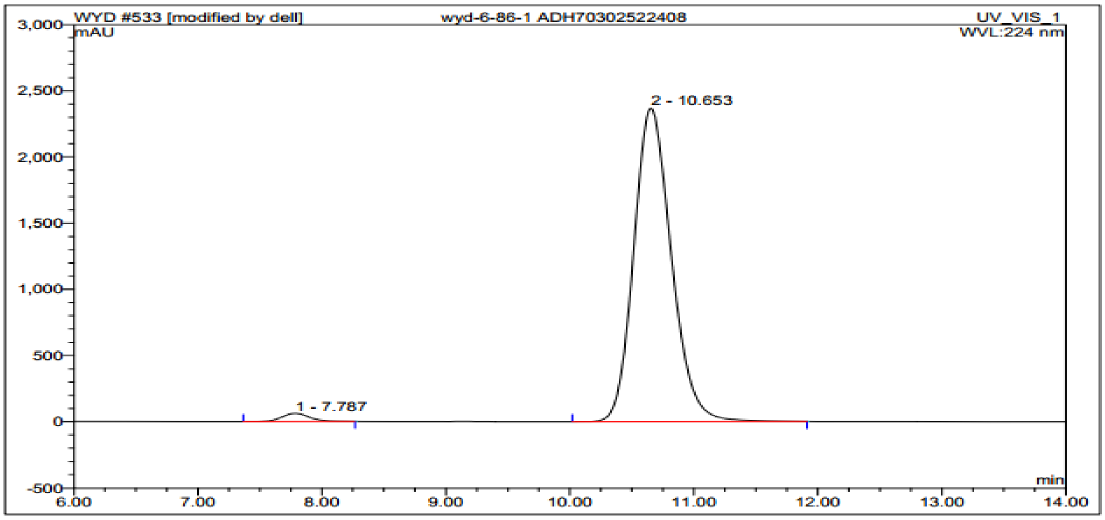

Peak #	Time(min)	Height (mAU)	Area (mAU*min)	Area (%)
1	7.79	61.099	15.512	1.83
2	10.65	2370.677	831.644	98.17

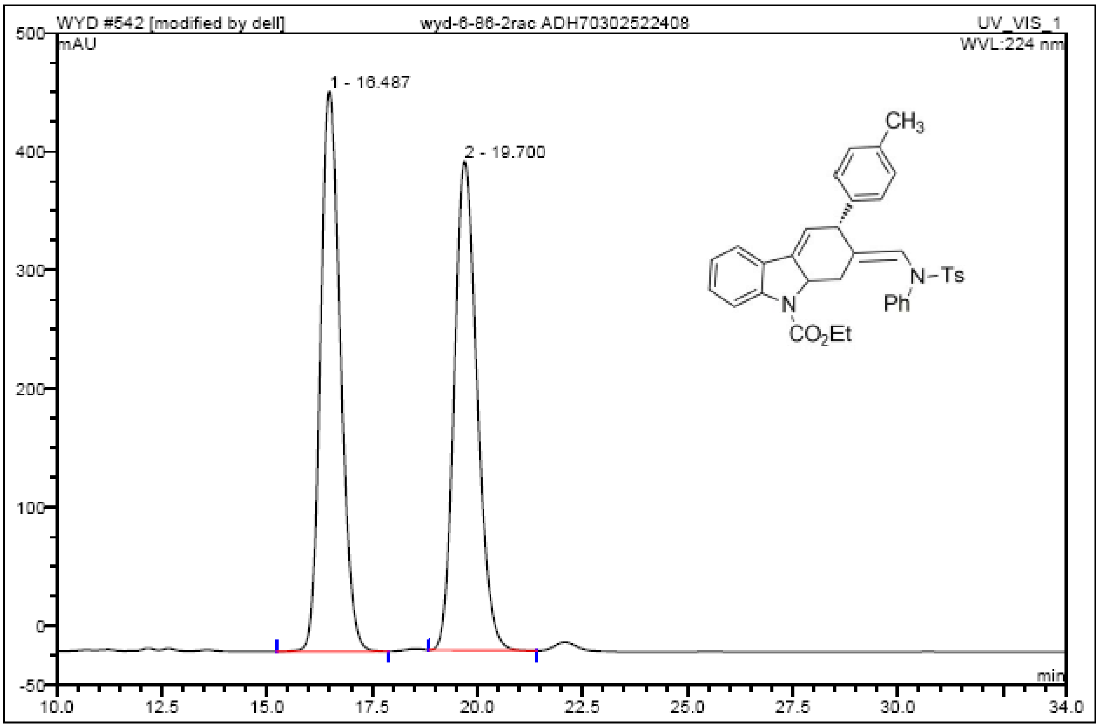

Peak #	Time(min)	Height (mAU)	Area (mAU*min)	Area (%)
1	16.49	472.257	260.684	50.22
2	19.70	412.102	258.449	49.78

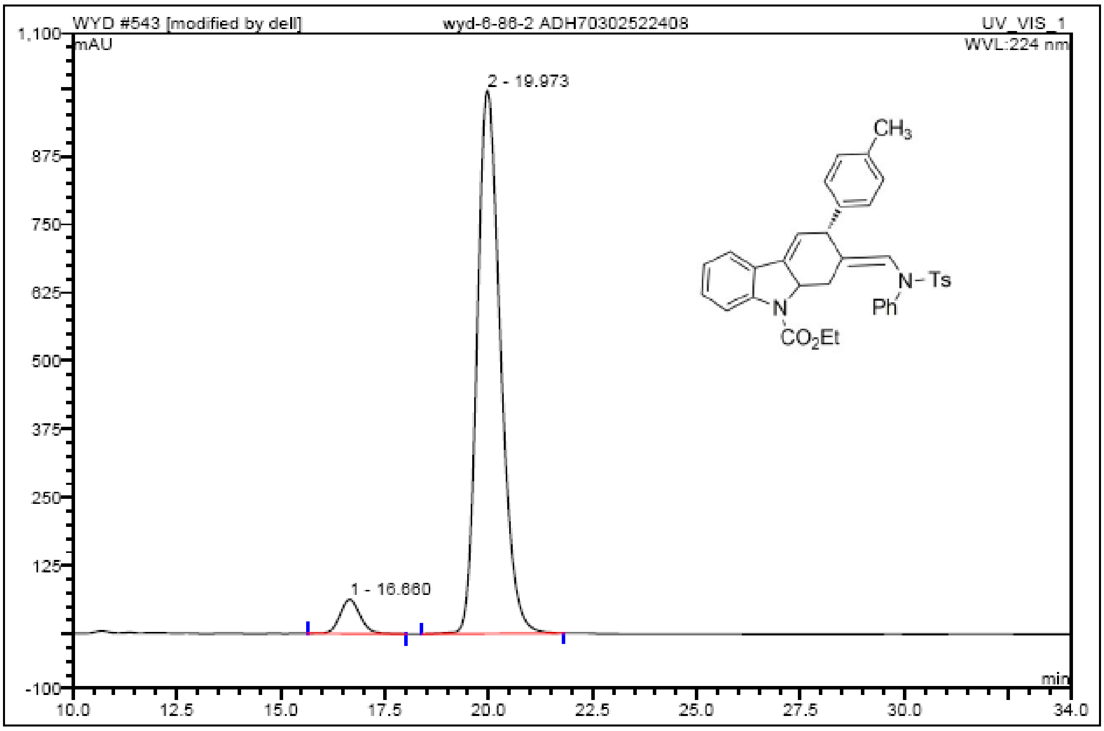

Peak #	Time(min)	Height (mAU)	Area (mAU*min)	Area (%)
1	16.66	62.124	34.712	5.09
2	19.97	994.890	646.932	94.91

26. (3*R*,9a*S*,*Z*)-ethyl 2-((4-methyl-*N*-phenylphenylsulfonamido)methylene)-3-(4-(trifluoromethyl)phenyl)-2,3-dihydro-1H-carbazole-9(9aH)-carboxylate (4g).

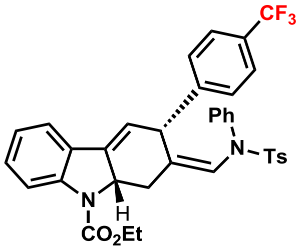

Isolated in 82% yield with 3.8:1 *Z*/*E* as white solid.

***Z*-isomer, ^{1}H NMR** (400 MHz, $CDCl_3$) δ 7.77 (s, 1 H), 7.44-7.36 (m, 4 H), 7.28 (d, *J* = 7.2 Hz, 1 H), 7.25-7.16 (m, 5 H), 7.16-7.10 (m, 3 H), 6.98 (d, *J* = 7.2 Hz, 1 H), 6.96-6.90 (m, 2 H), 6.39 (s, 1 H), 5.71 (t, *J* = 2.8 Hz, 1 H), 4.69-4.64 (m, 1 H), 4.63-4.59 (m, 1 H), 4.45-4.34 (m, 2 H), 3.49 (s, 1 H), 2.41 (s, 3 H), 2.46-2.35 (m, 1 H), 1.45 (t, *J* = 7.2 Hz, 3 H); **^{19}F NMR** (376 MHz, $CDCl_3$) δ -62.42; **^{13}C NMR** (100 MHz, $CDCl_3$) 153.8, 146.4, 144.1, 140.4, 137.0, 136.3, 133.6, 129.6, 129.4, 128.8, 128.4, 127.9, 127.8, 127.4, 127.1, 125.9, 125.4, 125.29, 125.26, 123.1, 122.7, 120.1, 118.3, 115.5, 62.0, 42.1, 34.9, 21.5, 14.6 (the peaks from C-F coupling are not well recognized); **HRMS** (ESI) calculated for $C_{36}H_{31}F_3N_2NaO_4S$ [M + Na$^+$]: 667.1849, found: 667.1811. **$[\alpha]_D^{20}$** = 294.1 (c = 0.5, $CHCl_3$), **HPLC conditions**: with a Chiralpak AD-H column (70: 30 hexane: 2-propanol, 0.8 mL/min, 224 nm); tr (minor) = 7.21 min, tr (major) = 21.18 min, 97% ee.

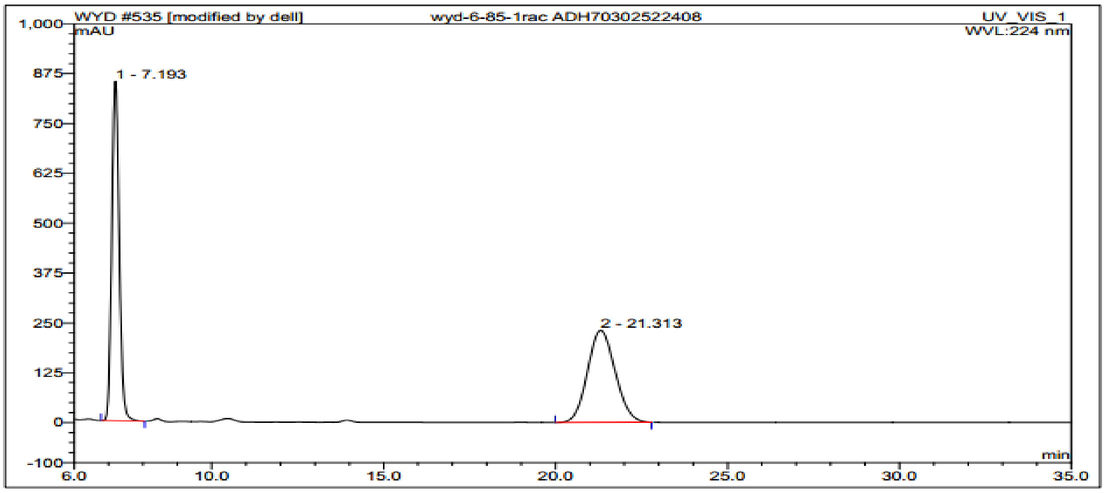

Peak #	Time(min)	Height (mAU)	Area (mAU*min)	Area (%)
1	7.19	851.561	213.819	49.89
2	21.31	230.649	214.784	50.11

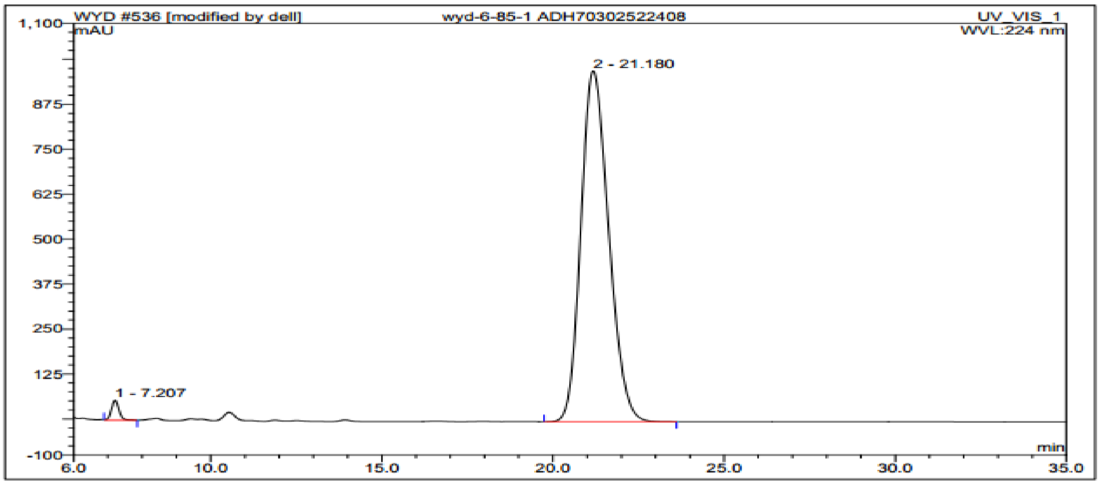

Peak #	Time (min)	Height (mAU)	Area (mAU*min)	Area (%)
1	7.21	55.002	14.062	1.51
2	21.18	975.568	918.420	98.49

27. **(3*R*,9a*S*,*Z*)-ethyl 3-(4-methoxyphenyl)-2-((4-methyl-*N*-phenylphenylsulfonamido)methylene)-2,3-dihydro-1H-carbazole-9(9aH)-carboxylate (4h).**

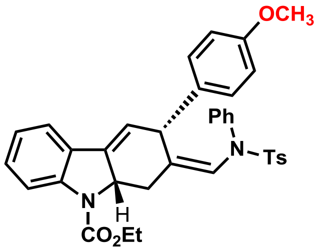

Isolated in 88% yield with 3.0:1 *Z*/*E* as white solid.

***Z*-isomer, ^{1}H NMR** (400 MHz, $CDCl_3$) δ 7.75 (s, 1 H), 7.41 (d, *J* = 8.0 Hz, 2 H), 7.29 (d, *J* = 7.6 Hz, 1 H), 7.25-7.15 (m, 6 H), 7.12-7.01 (m, 4 H), 6.97 (t, *J* = 7.6 Hz, 1 H), 6.72 (d, *J* = 8.8 Hz, 2 H), 6.30 (s, 1 H), 5.75 (t, *J* = 2.8 Hz, 1 H), 4.66-4.59 (m, 1 H), 4.49 (s, 1 H), 4.45-4.30 (m, 2 H), 3.75 (s, 3 H), 3.39 (s, 1 H), 2.46-2.37 (m, 1 H), 2.41 (s, 3 H), 1.44 (t, *J* = 6.8 Hz, 3 H); **^{13}C NMR** (100 MHz, $CDCl_3$) 158.2, 153.9, 143.9, 140.8, 137.7, 136.1, 134.5, 133.9, 129.4, 129.3, 128.8, 128.7, 127.9, 126.9, 126.6, 124.6, 123.0, 120.1, 119.6, 115.4, 113.8, 62.4, 61.9, 55.2, 41.4, 34.4, 21.5, 14.6; **HRMS** (ESI) calculated for $C_{36}H_{34}N_2NaO_5S$ [M + Na$^+$]: 629.2081, found: 629.2075. **$[\alpha]_D^{20}$** = 220.3 (c = 0.5, $CHCl_3$), **HPLC conditions**: with a Chiralpak AD-H column (70: 30 hexane: 2-propanol, 0.8 mL/min, 224 nm); tr (minor) = 12.15 min, tr (major) = 15.37 min, 95% ee.

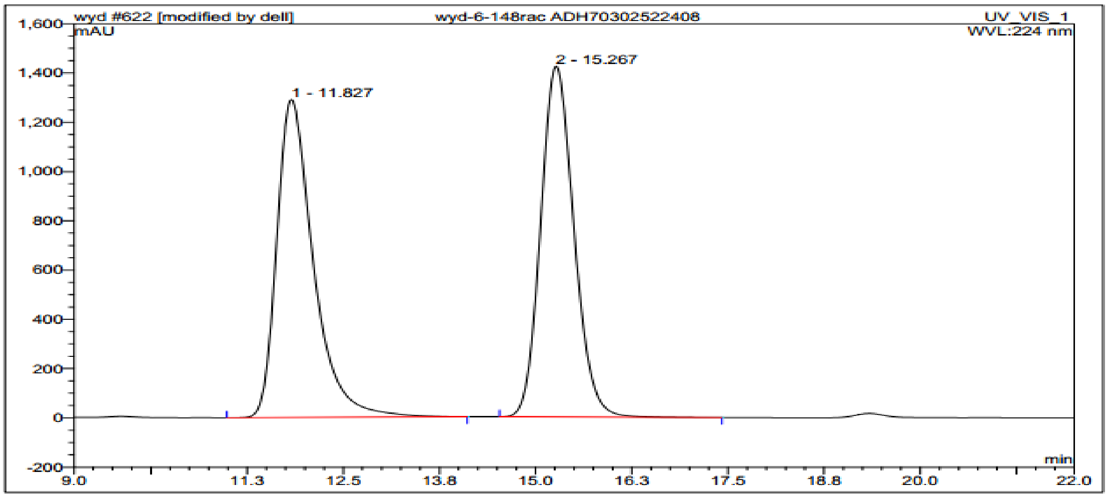

Peak #	Time(min)	Height (mAU)	Area (mAU*min)	Area (%)
1	11.83	1291.992	711.552	49.87
2	15.27	1424.265	715.167	50.13

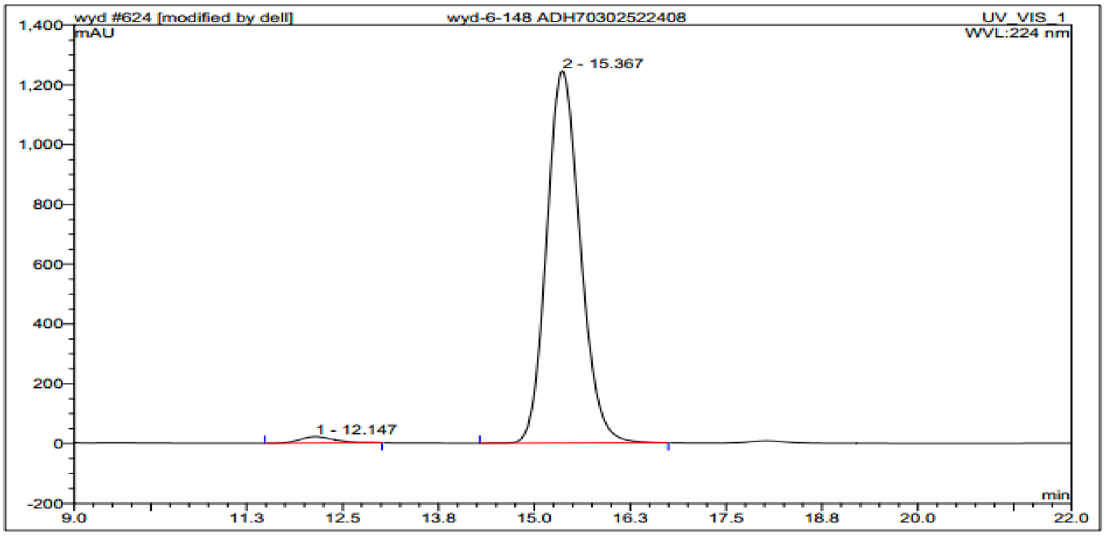

Peak #	Time(min)	Height (mAU)	Area (mAU*min)	Area (%)
1	12.15	20.825	11.125	1.73
2	15.37	1245.106	631.339	98.27

28. (3*R*,9a*S*,*Z*)-ethyl 6-methoxy-2-((4-methyl-*N*-phenylphenylsulfonamido)methylene)-3-phenyl-2,3-dihydro-1H-carbazole-9(9aH)-carboxylate. (4i)

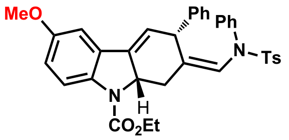

Isolated in 88% yield with 4.7:1 *Z*/*E* as white solid.

***Z*-isomer, ^{1}H NMR** (400 MHz, $CDCl_3$) δ 7.79 (s, 1 H), 7.40 (d, *J* = 8.0 Hz, 2 H), 7.25-7.07 (m, 9 H), 7.06-6.96 (m, 2 H), 6.83-6.79 (m, 1 H), 6.79-6.73 (m, 1 H), 6.32 (s, 1 H), 5.75 (t, *J* = 2.8 Hz, 1 H), 4.68-4.58 (m, 1 H), 4.54 (s, 1 H), 4.44-4.26 (m, 2 H), 3.74 (s, 3 H), 3.42 (s, 1 H), 2.50-2.30 (m, 1 H), 2.41 (s, 3 H), 2.49-2.36 (m, 1 H), 1.42 (t, *J* = 7.2 Hz, 3 H); **^{13}C NMR** (100 MHz, $CDCl_3$) 155.9, 153.8, 143.9, 142.3, 140.7, 137.3, 136.4, 133.8, 129.4, 128.8, 128.4, 127.9, 127.7, 127.0, 126.7, 126.5, 125.0, 119.6, 116.2, 115.4, 104.8, 104.0, 76.7, 62.5, 61.8, 55.6, 42.3, 34.7, 21.6, 14.6; **HRMS** (ESI) calculated for $C_{36}H_{34}N_2NaO_5S$ [M + Na^{+}]: 629.2081, found: 629.2010. **$[\alpha]_D^{20}$** = 235.6 (c = 0.5, $CHCl_3$), **HPLC conditions**: with a Chiralpak AD-H column (70: 30 hexane: 2-propanol, 0.8 mL/min, 224 nm); tr (minor) = 12.78 min, tr (major) = 14.03 min, 96% ee.

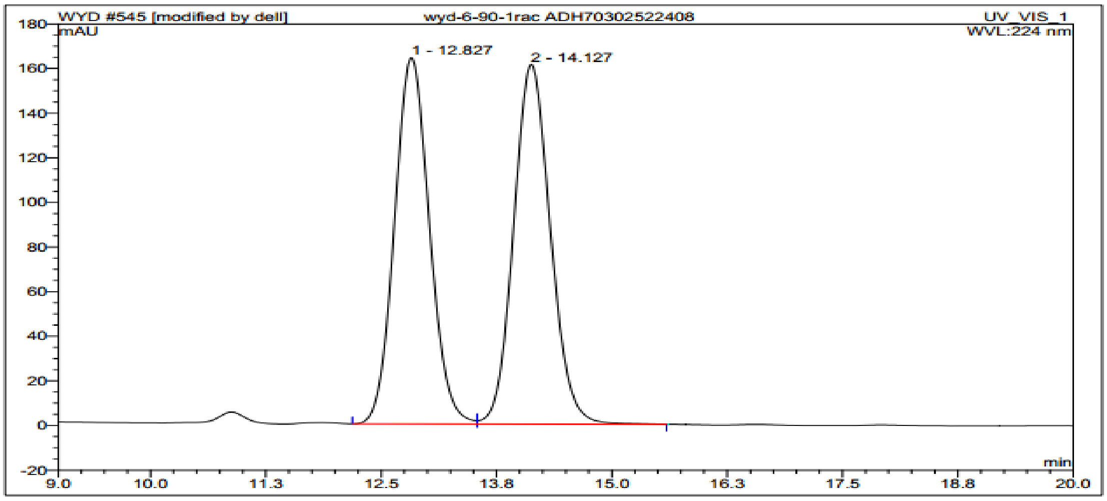

Peak #	Time (min)	Height (mAU)	Area (mAU*min)	Area (%)
1	12.83	164.131	72.061	49.22
2	14.13	161.187	74.334	50.78

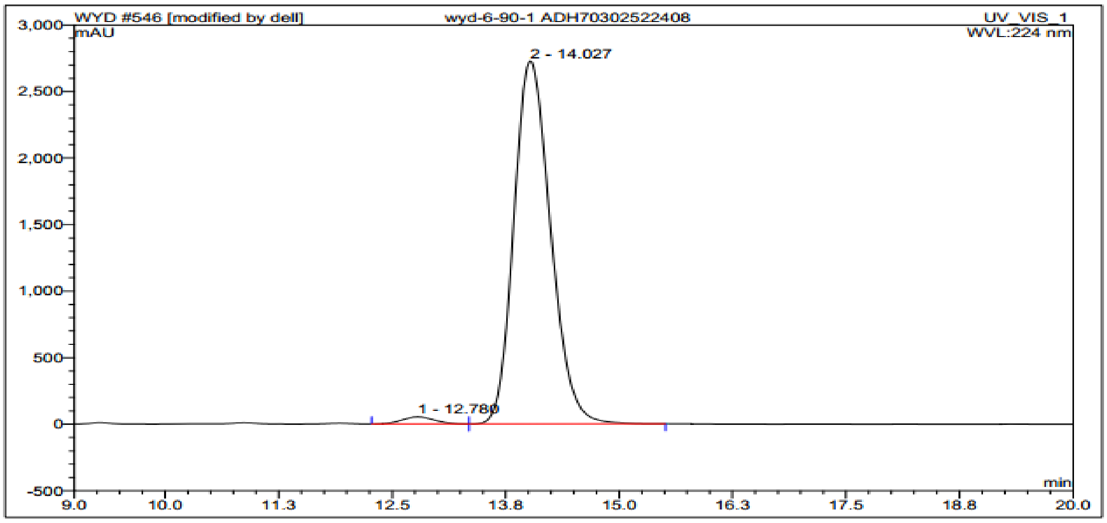

Peak #	Time(min)	Height (mAU)	Area (mAU*min)	Area (%)
1	12.78	52.113	21.598	1.66
2	14.03	2728.144	1276.720	98.34

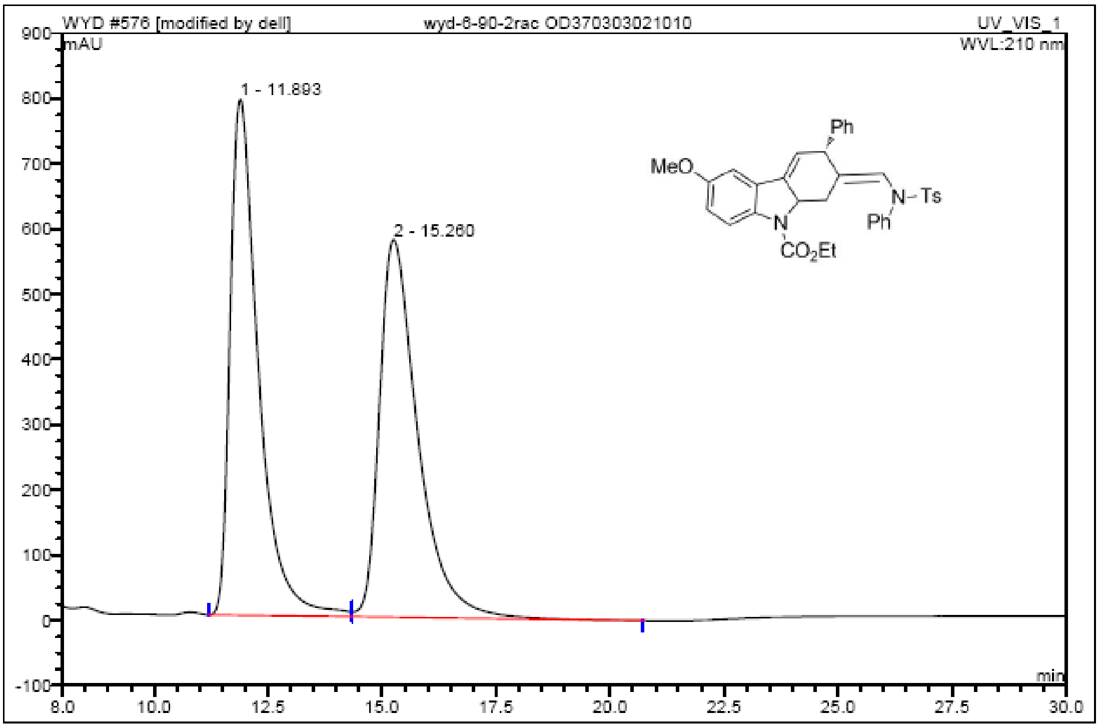

Peak #	Time(min)	Height (mAU)	Area (mAU*min)	Area (%)
1	11.89	791.383	568.275	50.09
2	15.26	578.660	566.150	49.91

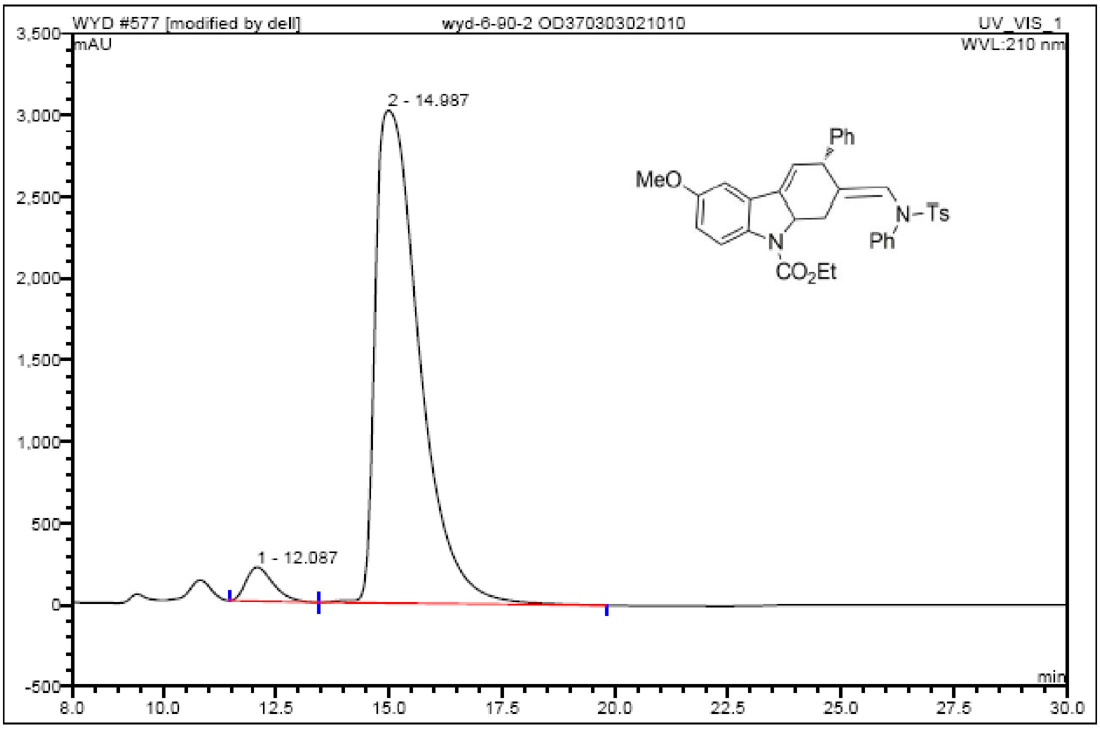

Peak #	Time (min)	Height (mAU)	Area (mAU*min)	Area (%)
1	12.09	207.874	146.259	4.12
2	14.99	3018.182	3403.289	95.88

29. (3*R*,9a*S*,*Z*)-ethyl 6-methyl-2-((4-methyl-*N*-phenylphenylsulfonamido)methylene)-3-phenyl-2,3-dihydro-1H-carbazole-9(9aH)-carboxylate (4j).

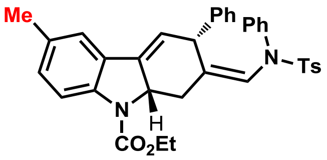

Isolated in 92% yield with 5.0:1 *Z*/*E* as white solid.

***Z*-isomer, ^{1}H NMR** (400 MHz, $CDCl_3$) δ 7.59 (brs, 1 H), 7.40 (d, *J* = 7.6 Hz, 2 H), 7.24-7.06 (m, 11 H), 7.06-6.96 (m, 3 H), 6.31 (s, 1 H), 5.73 (t, *J* = 2.8 Hz, 1 H), 4.68-4.58 (m, 1 H), 4.53 (s, 1 H), 4.44-4.26 (m, 2 H), 3.41 (brs, 1 H), 2.50-2.30 (m, 1 H), 2.41 (s, 3 H), 2.26 (s, 3H), 1.42 (t, *J* = 7.2 Hz, 3 H); **^{13}C NMR** (100 MHz, $CDCl_3$) 153.9 143.9, 142.4, 140.7, 137.5, 136.3, 133.9, 132.6, 130.0, 129.4, 128.7, 128.4, 127.9, 127.7, 126.9, 126.1, 126.4, 125.0, 120.5, 119.1, 115.2, 104.0, 62.4, 61.8, 42.2, 34.7, 21.5, 20.8, 14.6; **HRMS** (ESI) calculated for $C_{36}H_{34}N_2NaO_4S$ [M + Na^+]: 613.2131, found: 613.2130. **$[\alpha]_D^{20}$** = 210.2 (c = 0.5, $CHCl_3$), **HPLC conditions**: with a Chiralpak AD-H column (70: 30 hexane: 2-propanol, 0.8 mL/min, 224 nm); tr (minor) = 10.64 min, tr (major) = 14.91 min, 96% ee.

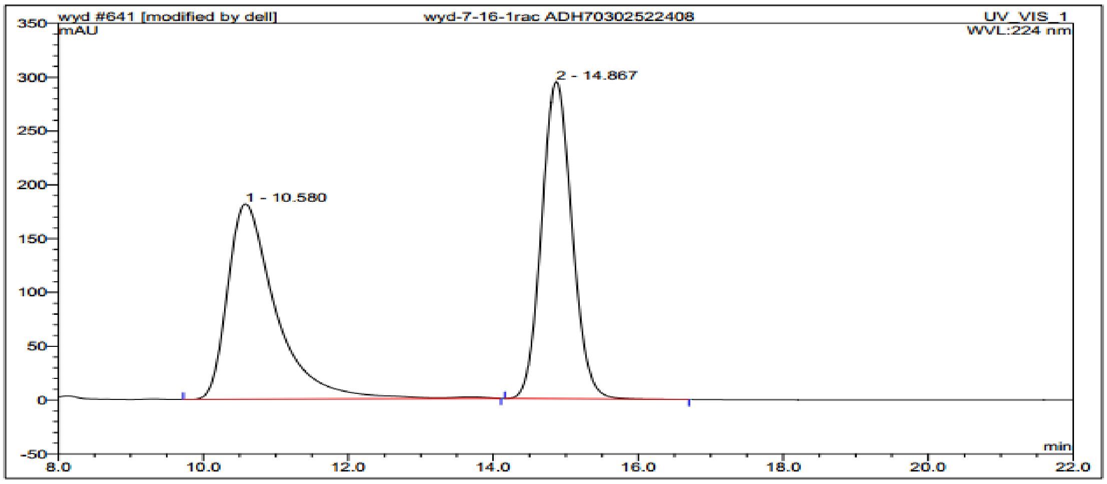

Peak #	Time(min)	Height (mAU)	Area (mAU*min)	Area (%)
1	10.58	181.375	139.798	49.59
2	14.87	294.630	142.131	50.41

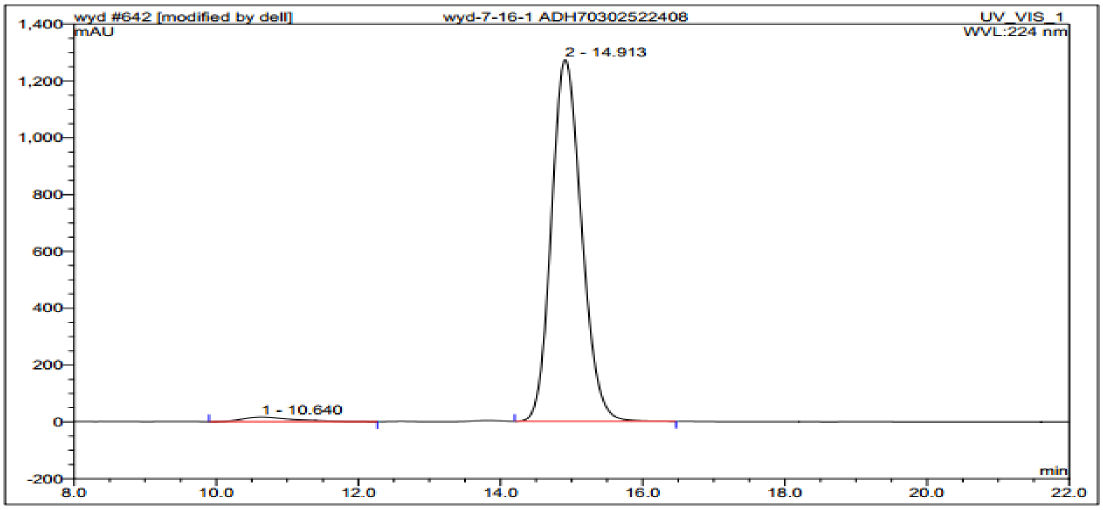

Peak #	Time(min)	Height (mAU)	Area (mAU*min)	Area (%)
1	10.64	16.290	12.836	2.01
2	14.91	1272.469	624.801	97.99

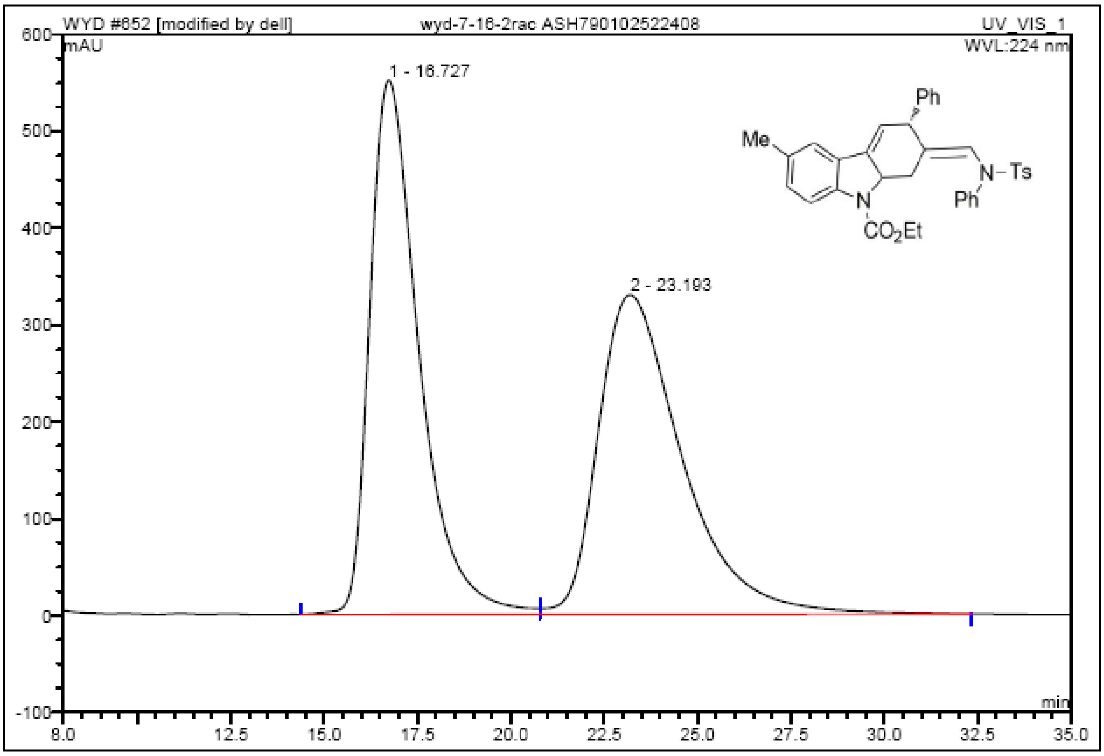

Peak #	Time(min)	Height (mAU)	Area (mAU*min)	Area (%)
1	16.73	551.559	844.011	49.64
2	23.19	329.907	856.287	50.36

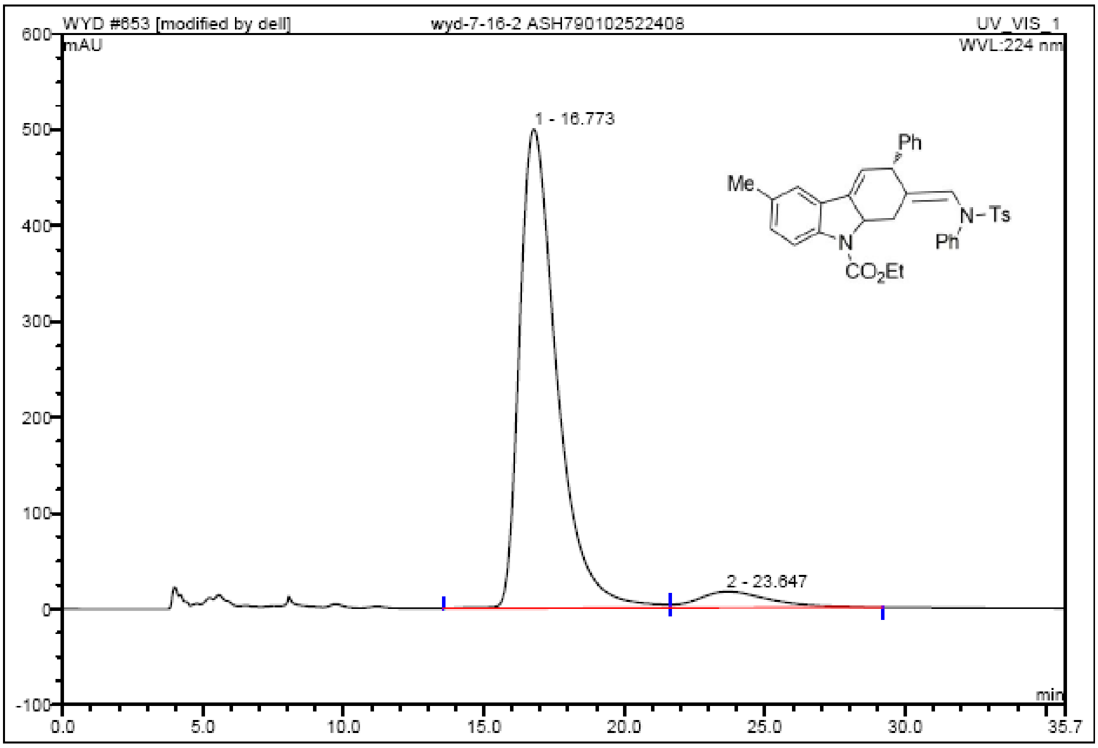

Peak #	Time(min)	Height (mAU)	Area (mAU*min)	Area (%)
1	16.77	499.835	768.411	93.92
2	23.65	16.702	49.703	6.08

30. (3*R*,9a*S*,*Z*)-ethyl 6-bromo-2-((4-methyl-*N*-phenylphenylsulfonamido)methylene)-3-phenyl-2,3-dihydro-1H-carbazole-9(9aH)-carboxylate(4k).

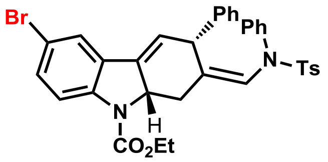

Isolated in 97% yield with 5.2:1 *Z*/*E* as white solid.

***Z*-isomer, ^{1}H NMR** (400 MHz, $CDCl_3$) δ 7.63 (s, 1 H), 7.40 (d, *J* = 8.0 Hz, 2 H), 7.36 (s, 1 H), 7.28-7.07 (m, 11 H), 7.03-6.95 (m, 2 H), 6.35 (s, 1 H), 5.76 (t, *J* = 2.8 Hz, 1 H), 4.66-4.62 (m, 1 H), 4.56-4.50 (m, 1 H), 4.43-4.30 (m, 2 H), 3.40 (s, 1 H), 2.40 (s, 3 H), 2.47-2.37 (m, 1 H), 1.43 (t, *J* = 7.2 Hz, 3 H); **^{13}C NMR** (100 MHz, $CDCl_3$) 153.5, 143.9, 143.1, 141.9, 140.6, 136.5, 135.1, 134.0, 131.8, 129.9, 129.4, 128.8, 128.5, 127.9, 127.6, 127.0, 126.8, 126.6, 125.3, 123.1, 121.1, 116.9, 115.7, 62.6, 62.1, 42.3, 34.8, 21.5, 14.6; **HRMS** (ESI) calculated for $C_{35}H_{31}BrN_2NaO_4S$ [M + Na^+]: 677.1080, found: 677.1016. **$[\alpha]_D^{20}$** = 213.6 (c = 0.5, $CHCl_3$), **HPLC conditions**: with a Chiralpak AD-H column (70: 30 hexane: 2-propanol, 0.8 mL/min, 224 nm); tr (minor) = 10.05 min, tr (major) = 13.55 min, 97% ee.

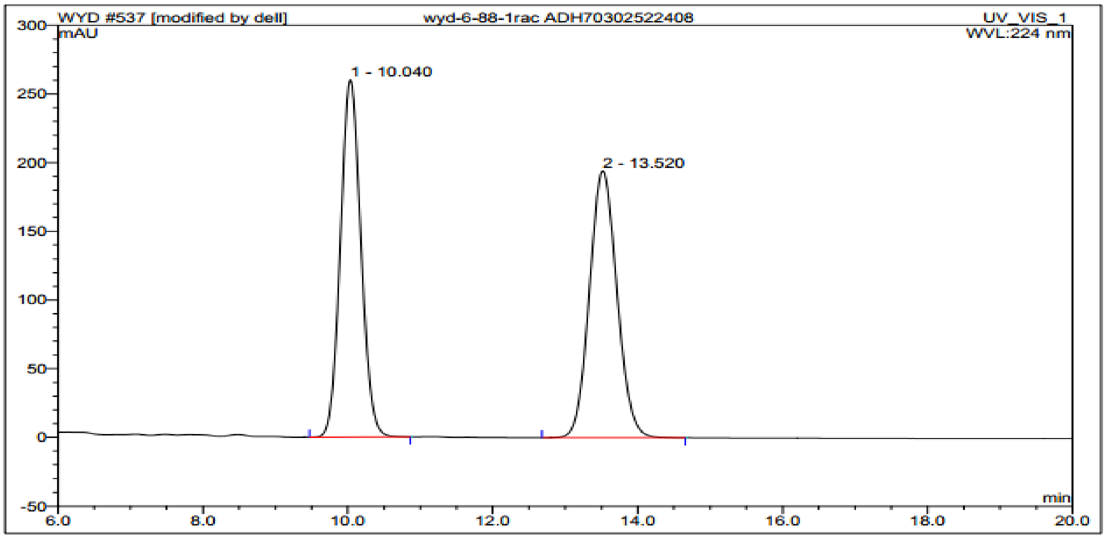

Peak #	Time(min)	Height (mAU)	Area (mAU*min)	Area (%)
1	10.04	260.032	84.850	49.75
2	13.53	194.404	85.708	50.25

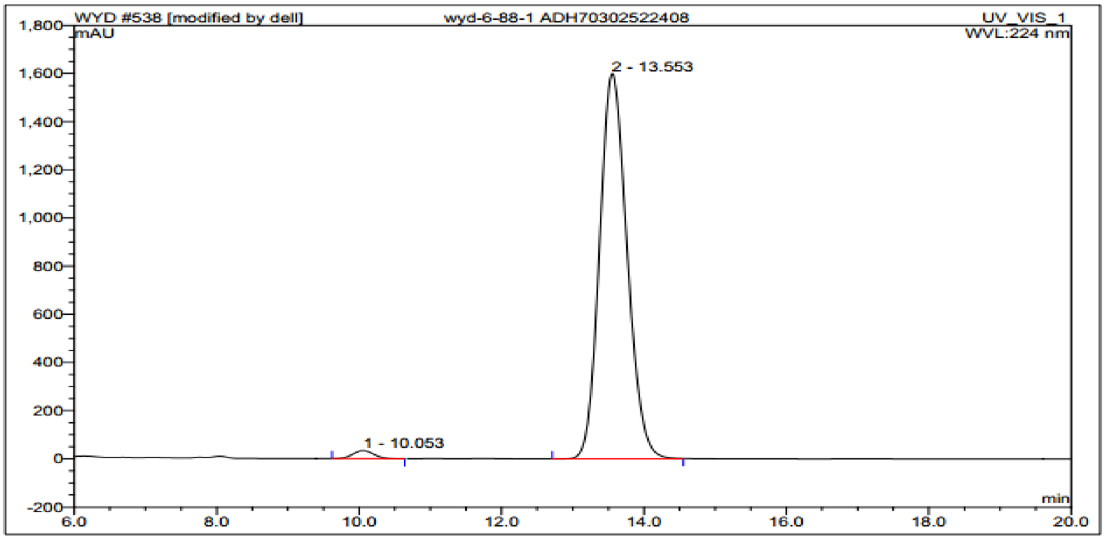

Peak #	Time (min)	Height (mAU)	Area (mAU*min)	Area (%)
1	10.05	33.090	11.100	1.53
2	13.55	1599.328	715.450	98.47

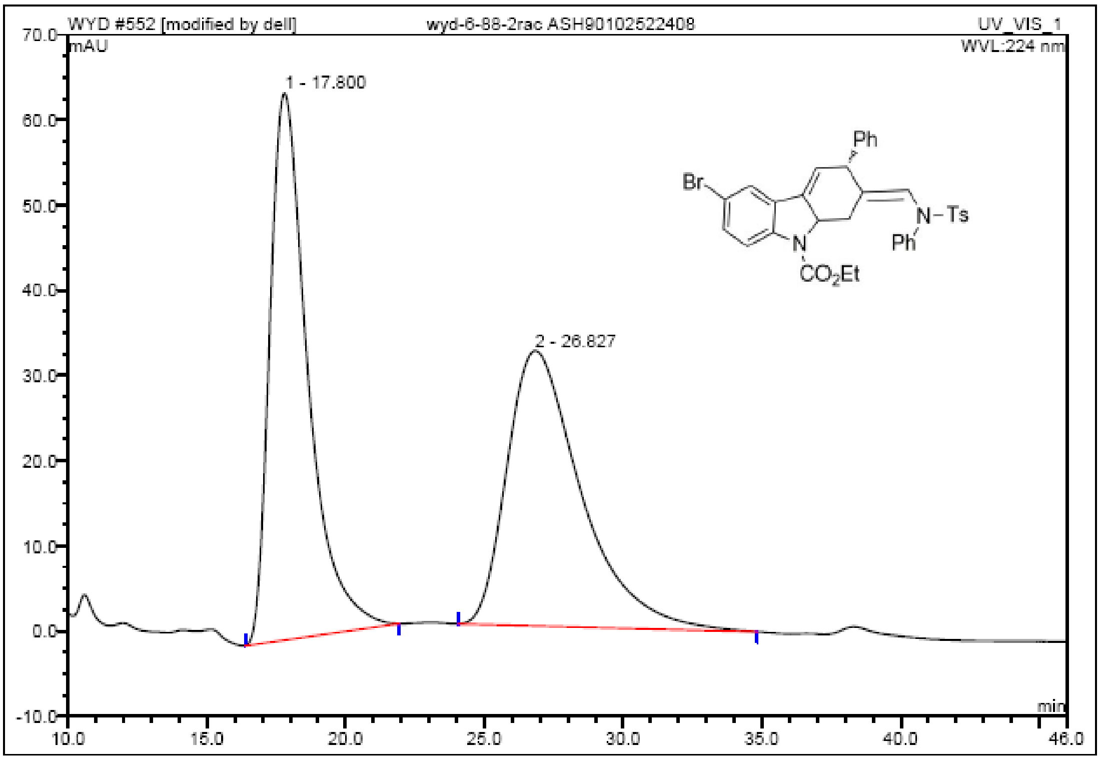

Peak #	Time(min)	Height (mAU)	Area (mAU*min)	Area (%)
1	17.80	64.161	101.810	50.43
2	26.83	32.299	100.059	49.57

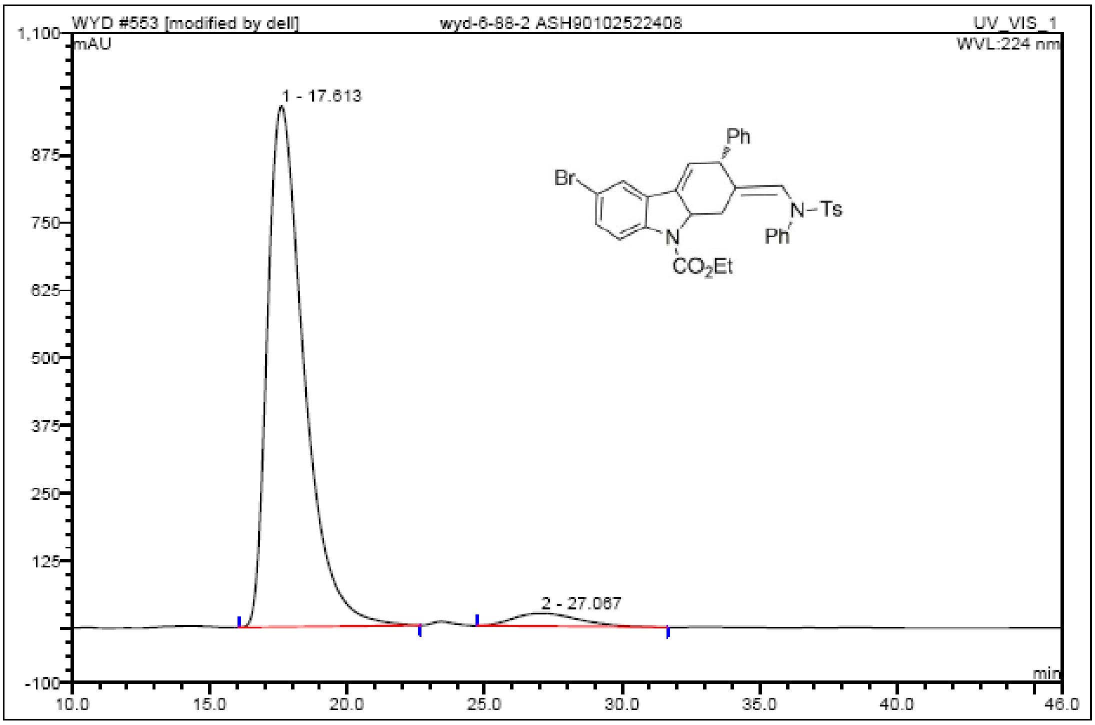

Peak #	Time (min)	Height (mAU)	Area (mAU*min)	Area (%)
1	17.61	961.065	1480.973	95.81
2	27.07	23.896	64.825	4.19

31. (3*R*,9a*S*,*Z*)-ethyl 5-bromo-2-((4-methyl-N-phenylphenylsulfonamido)methylene)-3-phenyl-2,3-dihydro -1H-carbazole-9(9aH)-carboxylate(4l).

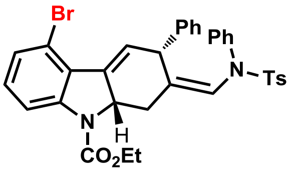

Isolated in 99% yield with 6.1:1 *Z*/*E* as white solid.

***Z*-isomer, ^{1}H NMR** (400 MHz, $CDCl_3$) δ 7.79 (s, 1 H), 7.41 (d, *J* = 8.0 Hz, 2 H), 7.22-7.11 (m, 11 H), 7.05-6.96 (m, 3 H), 6.65 (t, *J* = 2.8 Hz, 1 H), 6.34 (s, 1 H), 4.67-4.63 (m, 1 H), 4.57 (s, 1 H), 4.42-4.32 (m, 2 H), 3.43 (d, *J* = 8.8 Hz, 1 H), 2.40 (s, 3 H), 2.49-2.36 (m, 1 H), 1.43 (t, *J* = 7.2 Hz, 3 H); **^{13}C NMR** (100 MHz, $CDCl_3$) 153.5, 146.1, 143.9, 142.0, 140.6, 136.6, 134.7, 134.1, 129.5, 129.4, 128.8, 128.4, 127.9, 127.8, 127.0, 126.8, 126.5, 126.1, 124.9, 124.1, 117.0, 114.1, 62.5, 62.2, 42.4, 34.8, 21.5, 14.5; **HRMS** (ESI) calculated for $C_{35}H_{31}BrN_2NaO_4S$ [M + Na^+]: 677.1080, found: 677.1014. **$[\alpha]_D^{20}$** = 227.2 (c = 0.4, $CHCl_3$), **HPLC conditions**: with a Chiralpak AS-H column (90: 10 hexane: 2-propanol, 0.8 mL/min, 224 nm); tr (minor) = 17.92 min, tr (major) = 13.66 min, 97% ee.

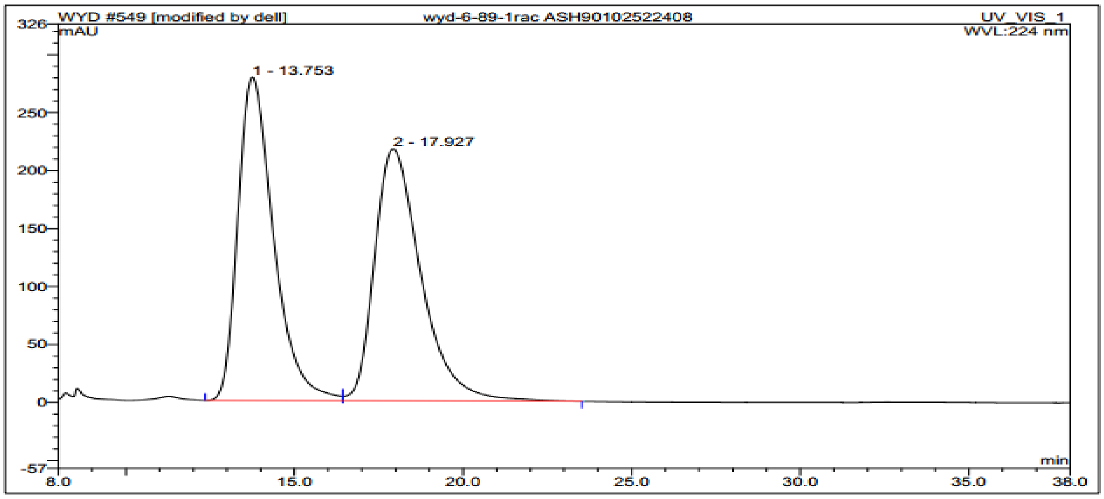

Peak #	Time (min)	Height (mAU)	Area (mAU*min)	Area (%)
1	13.75	278.932	343.889	49.73
2	17.93	217.167	347.574	50.27

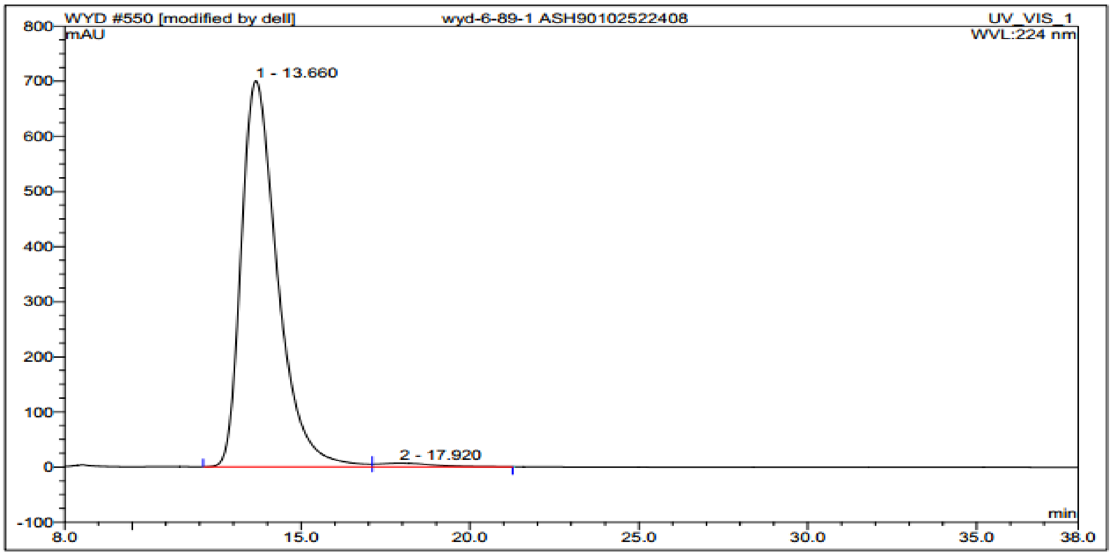

Peak #	Time (min)	Height (mAU)	Area (mAU*min)	Area (%)
1	13.66	700.270	869.329	98.67
2	17.92	6.421	11.756	1.33

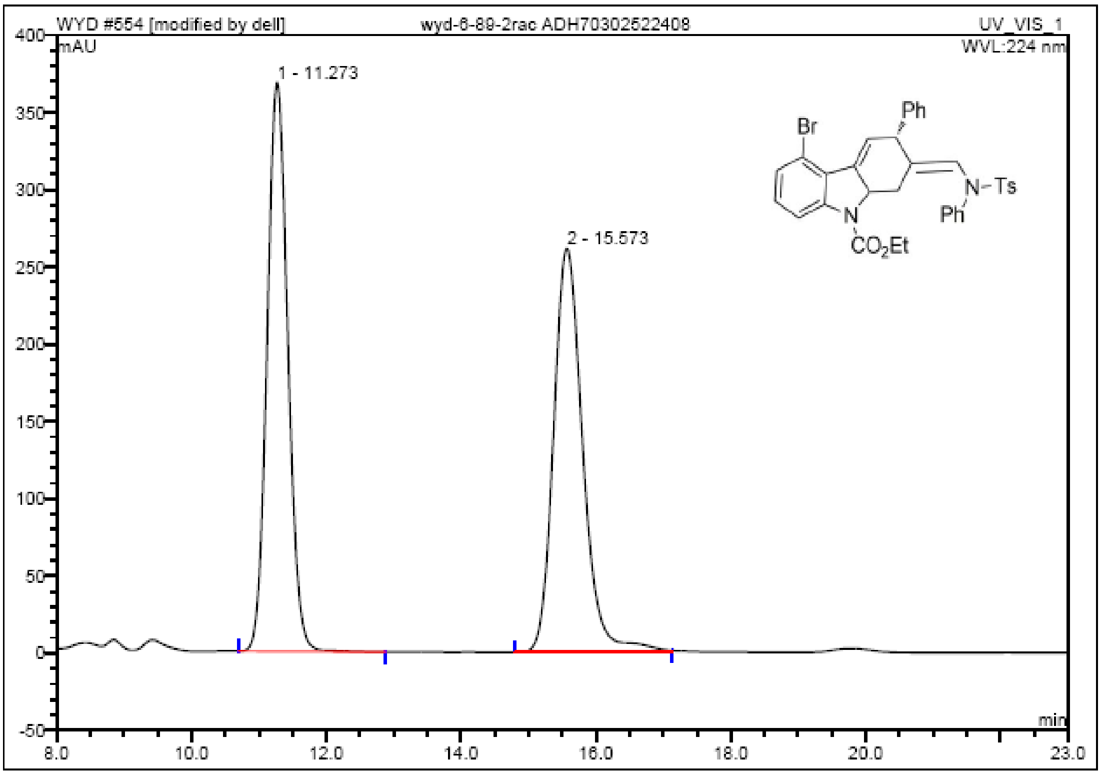

Peak #	Time (min)	Height (mAU)	Area (mAU*min)	Area (%)
1	11.27	368.188	130.561	49.85
2	15.57	261.364	131.349	50.15

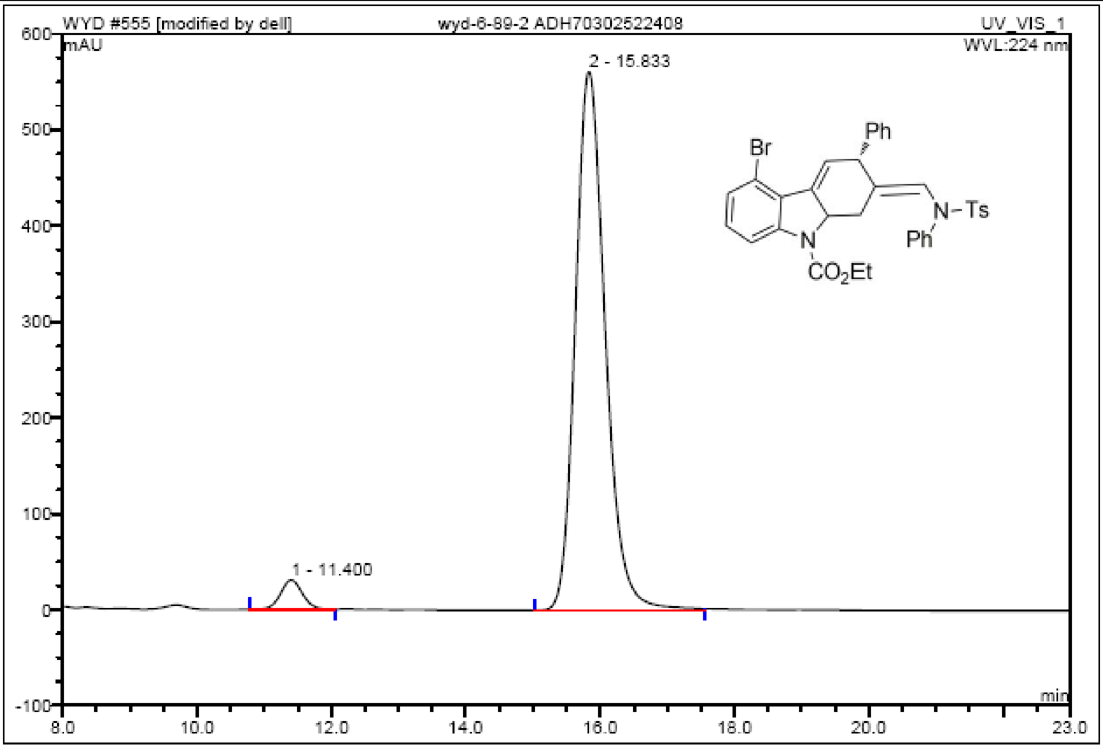

Peak #	Time (min)	Height (mAU)	Area (mAU*min)	Area (%)
1	11.40	30.377	11.389	3.78
2	15.83	561.620	289.656	96.22

32. (3*S*,9a*S*,*Z*)-ethyl 2-((4-methyl-*N*-phenylphenylsulfonamido)methylene)-3-(thiophen-2-yl)-2,3-dihydro-1H-carbazole-9(9aH)-carboxylate (4m).

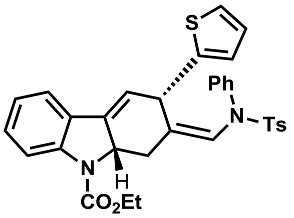

Isolated in 90% yield with 1:1 Z/*E* as white solid.

***Z*-isomer, ^{1}H NMR** (400 MHz, $CDCl_3$) δ 7.76 (s, 1 H), 7.43 (d, *J* = 8.0 Hz, 2 H), 7.30 (d, *J* = 7.6 Hz, 1 H), 7.27-7.17 (m, 6 H), 7.15-7.10 (m, 2 H), 7.07-7.03 (m, 1 H), 6.97 (t, *J* = 7.6 Hz, 1 H), 6.82-6.76 (m, 1 H), 6.68 (s, 1 H), 6.34 (s, 1 H), 5.82 (t, *J* = 3.2 Hz, 1 H), 4.77 (s, 1 H), 4.68-4.56 (m, 1 H), 4.46-4.28 (m, 2 H), 3.39 (s, 1 H), 2.53-2.38 (m, 1 H), 2.41 (s, 3 H), 1.43 (t, *J* = 7.2 Hz, 3 H); **^{13}C NMR** (100 MHz, $CDCl_3$) δ 153.8, 145.2, 144.0, 140.8, 136.7, 136.2, 134.0, 129.5, 129.4, 128.9, 127.9, 127.7, 127.2, 127.0, 126.5, 125.1, 124.5, 123.9, 123.1, 120.2, 118.4, 115.5, 62.6, 62.0, 37.5, 33.9, 21.6, 14.6; **HRMS** (ESI) calculated for $C_{33}H_{30}N_2NaO_4S_2$ [M + Na$^+$]: 605.1539, found: 605.1533. **$[\alpha]_D^{20}$** = 244.4 (c = 0.5, $CHCl_3$), **HPLC conditions**: with a Chiralpak AD-H column (85: 15 hexane: 2-propanol, 1.0 mL/min, 224 nm); tr (minor) = 15.33 min, tr (major) = 14.05 min, 93%

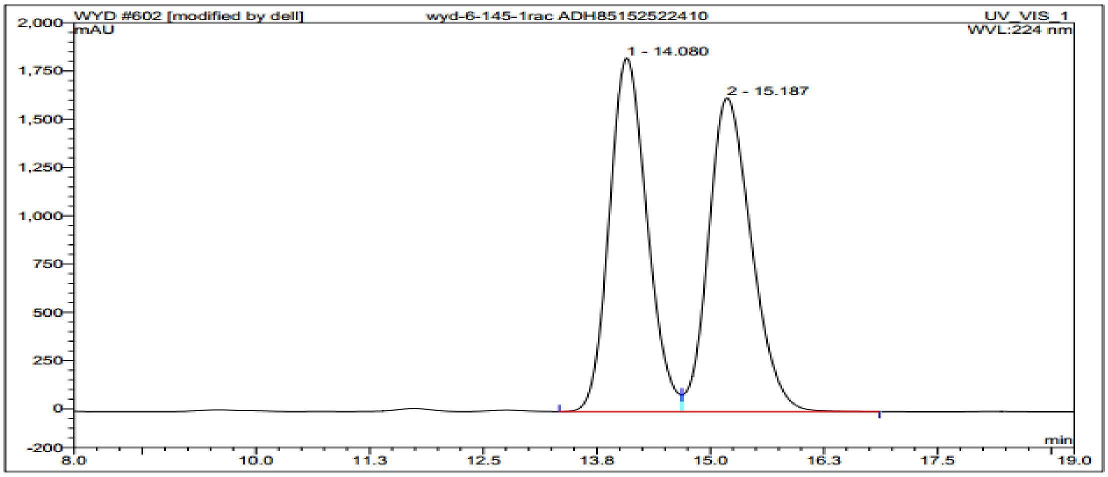

Peak #	Time (min)	Height (mAU)	Area (mAU*min)	Area (%)
1	14.08	1830.811	880.968	49.73
2	15.33	1624.767	890.614	50.27

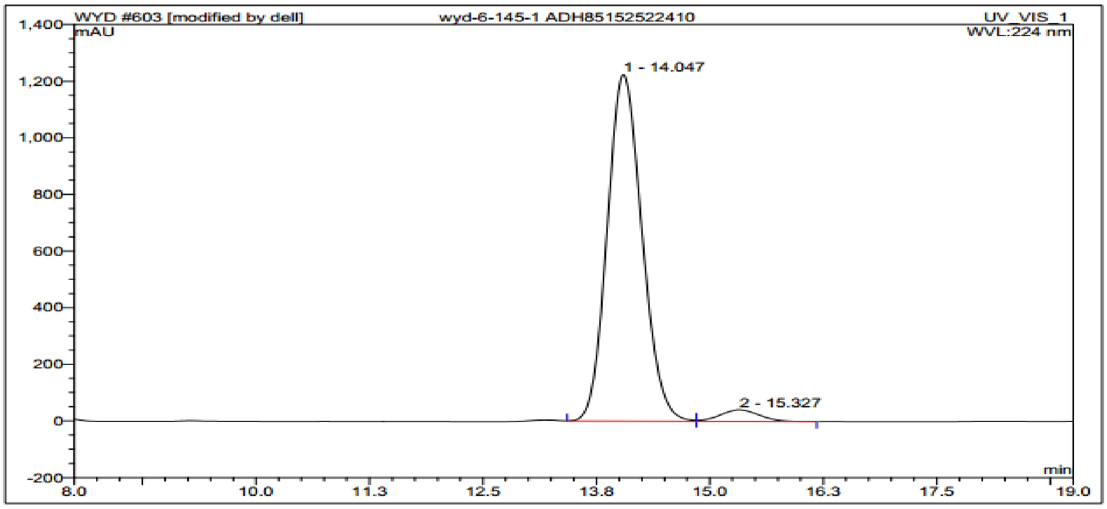

Peak #	Time (min)	Height (mAU)	Area (mAU*min)	Area (%)
1	14.05	1223.650	543.138	96.49
2	15.33	40.289	19.773	3.51

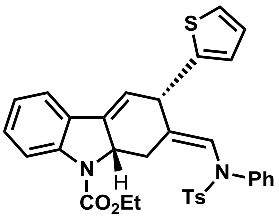

***E*-isomer, ^{1}H NMR** (400 MHz, $CDCl_3$) δ 7.91 (s, 1 H), 7.36 (d, *J* = 7.6 Hz, 3 H), 7.26-7.15 (m, 7 H), 7.08 (d, *J* = 7.6 Hz, 2 H), 6.99 (t, *J* = 7.6 Hz, 1 H), 6.96-6.92 (m, 1 H), 6.86 (d, *J* = 3.2 Hz, 1 H), 6.30 (s, 1 H), 6.02 (t, *J* = 2.8 Hz, 1 H), 4.53 (s, 2 H), 4.43-4.24 (m, 2 H), 3.87-3.77 (m, 1 H), 2.39 (s, 3 H), 1.86 (t, *J* = 11.6 Hz, 1 H), 1.43 (t, *J* = 7.2 Hz, 3 H); **^{13}C NMR** (100 MHz, $CDCl_3$) δ 153.4, 147.4, 144.9, 143.8, 141.3, 138.5, 138.0, 134.4, 129.8, 129.4, 129.0, 127.8, 127.1, 127.1, 126.8, 126.6, 124.5, 124.3, 123.0, 120.1, 117.1, 115.6, 61.8, 60.9, 41.8, 29.5, 21.6, 14.7; **HRMS** (ESI) calculated for $C_{33}H_{30}N_2NaO_4S_2$ [M + Na^+]: 605.1539, found: 605.1528. **$[\alpha]_D^{20}$** = 37.6 (c = 1.0, $CHCl_3$), **HPLC conditions**: with a Chiralpak AD-H column (70: 30 hexane: 2-propanol, 0.8 mL/min, 224 nm); tr (minor) = 40.07 min, tr (major) = 19.92 min, 91% ee.

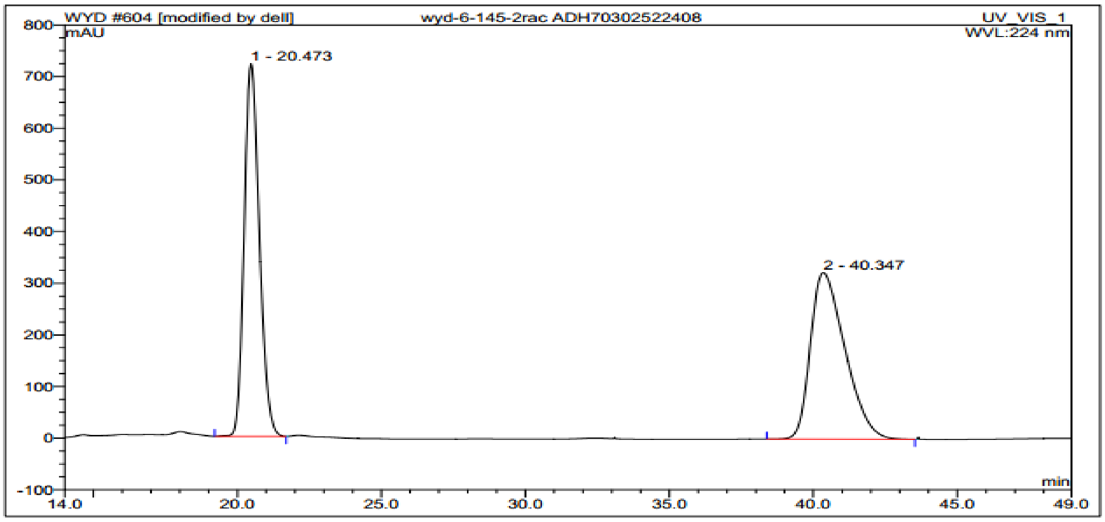

Peak #	Time (min)	Height (mAU)	Area (mAU*min)	Area (%)
1	20.47	721.623	442.539	49.62
2	40.35	322.411	449.238	50.38

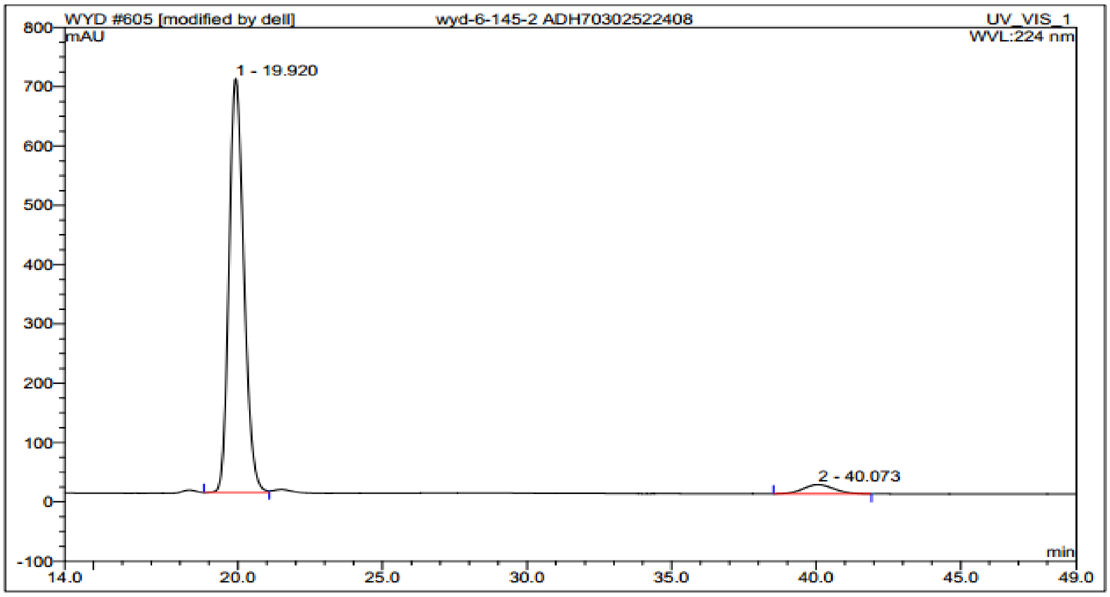

Peak #	Time (min)	Height (mAU)	Area (mAU*min)	Area (%)
1	19.92	698.154	416.831	95.56
2	40.07	15.367	19.348	4.44

33. (3*R*,9a*S*,*Z*)-ethyl 2-((N-(4-methoxyphenyl)-4-methylphenylsulfonamido)methylene)-3-phenyl-2,3-dihydro-1H-carbazole-9(9aH)-carboxylate (4n).

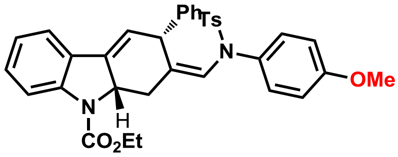

Isolated in 89% yield with 10:1 *Z*/*E* as white solid.

***Z*-isomer,** ^{1}**H NMR** (400 MHz, $CDCl_3$) δ 7.76 (s, 1 H), 7.41 (d, *J* = 8.0 Hz, 2 H), 7.28 (d, *J* = 7.2 Hz, 1 H), 7.24-7.09 (m, 8 H), 6.95 (t, *J* = 7.6 Hz, 1 H), 6.86 (d, *J* = 8.8 Hz, 2 H), 6.66 (d, *J* = 8.8 Hz, 2 H), 6.34 (s, 1 H), 5.76 (t, *J* = 2.8 Hz, 1 H), 4.66-4.59 (m, 1 H), 4.56-4.50 (m, 1 H), 4.44-4.31 (m, 2 H), 3.76 (s, 3 H), 3.41 (s, 1 H), 2.41 (s, 3 H), 2.47-2.36 (m, 1 H), 1.43 (t, *J* = 7.2 Hz, 3 H); ^{13}**C NMR** (100 MHz, $CDCl_3$) 158.4, 153.8, 143.8, 142.5, 136.2, 135.5, 134.0, 133.2, 129.4, 129.3, 128.5, 128.4, 128.0, 127.8, 127.7, 126.4, 125.5, 123.0, 120.1, 119.7, 115.5, 113.9, 62.3, 61.9, 55.3, 42.2, 34.9, 21.5, 14.6; **HRMS** (ESI) calculated for $C_{36}H_{34}N_2NaO_4S$ [M + Na^+]: 629.2081, found: 629.2005. $[\alpha]_D^{20}$ = 300.8 (c = 0.5, $CHCl_3$), **HPLC conditions**: with a Chiralpak OD-H column (90: 10 hexane: 2-propanol, 0.8 mL/min, 224 nm); tr (minor) = 17.00 min, tr (major) = 19.83 min, 90% ee.

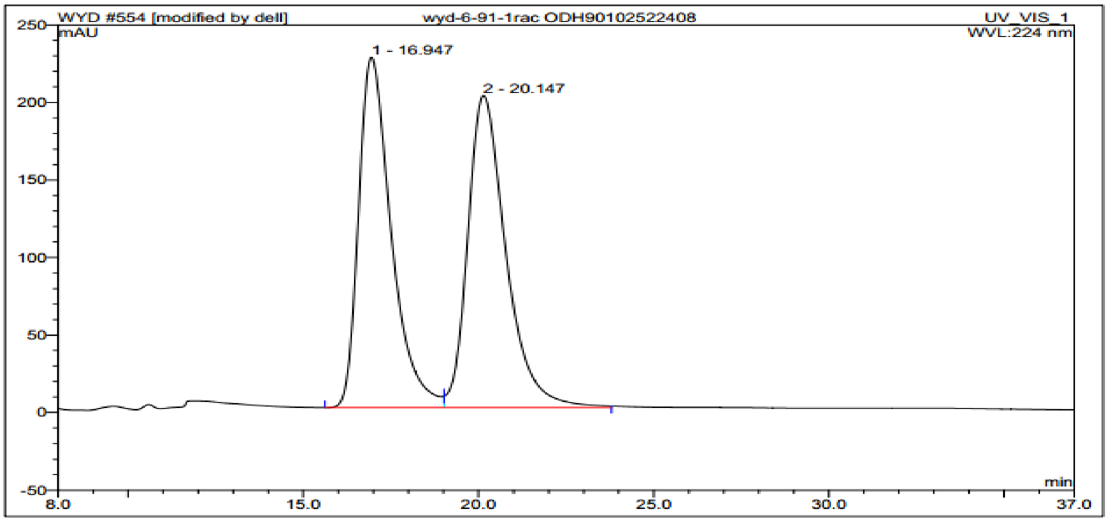

Peak #	Time (min)	Height (mAU)	Area (mAU*min)	Area (%)
1	16.95	226.185	244.868	48.97
2	20.15	201.503	255.211	51.01

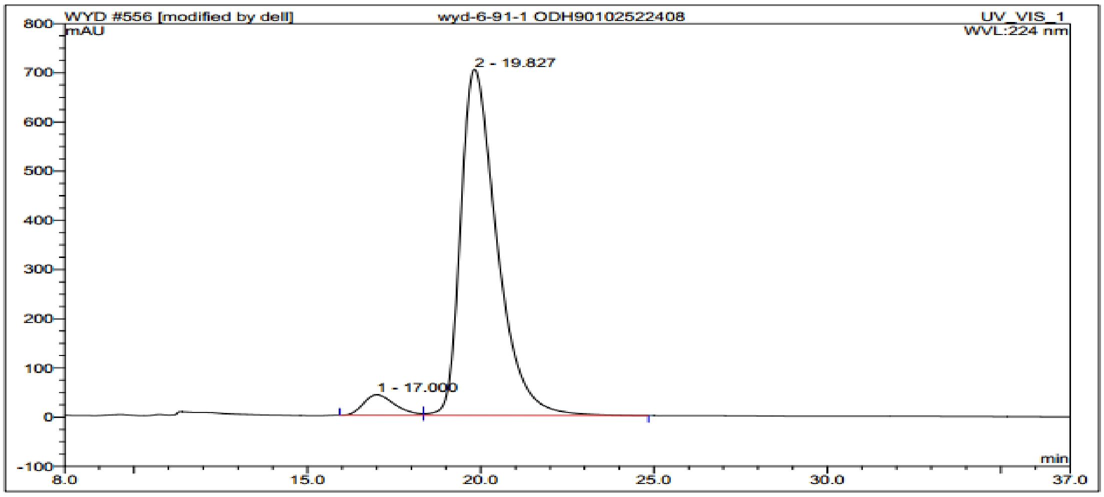

Peak #	Time (min)	Height (mAU)	Area (mAU*min)	Area (%)
1	17.00	41.576	43.402	4.85
2	19.83	703.499	852.009	95.15

34. (3*R*,9a*S*,*Z*)-ethyl 2-((*N*-(4-bromophenyl)-4-methylphenylsulfonamido)methylene)-3-phenyl-2,3-dihydro -1H-carbazole-9(9aH)-carboxylate (4o).

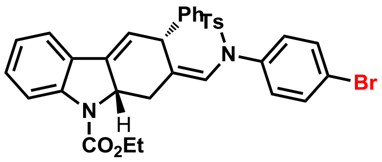

Isolated in 92% yield with 14:1 *Z*/*E* as white solid.

***Z*-isomer, ^{1}H NMR** (400 MHz, $CDCl_3$) δ 7.75 (s, 1 H), 7.41 (d, *J* = 8.0 Hz, 2 H), 7.29 (d, *J* = 7.2 Hz, 1 H), 7.25-7.10 (m, 8 H), 7.08-7.02 (m, 2 H), 6.96 (t, *J* = 7.6 Hz, 1 H), 6.86-6.81 (m, 2 H), 6.28 (s, 1 H), 5.77 (t, *J* = 2.8 Hz, 1 H), 4.70-4.60 (m, 1 H), 4.55-4.48 (m, 1 H), 4.45-4.30 (m, 2 H), 3.45 (s, 1 H), 2.41 (s, 3 H), 2.49-2.40 (m, 1 H), 1.44 (t, *J* = 7.2 Hz, 3 H); **^{13}C NMR** (100 MHz, $CDCl_3$) 153.8, 144.2, 142.1, 139.7, 137.9, 136.2, 133.6, 131.8, 129.5, 129.4, 128.5, 128.1, 127.8, 127.7, 127.6, 126.4, 124.7, 123.1, 120.6, 120.2, 119.4, 115.5, 62.1, 61.9, 42.5, 35.0, 21.5, 14.6; **HRMS** (ESI) calculated for $C_{35}H_{31}BrN_2NaO_4S$ [M + Na^+]: 677.1080, found: 677.0998. **$[\alpha]_D^{20}$** = 273.6 (c = 0.5, $CHCl_3$), **HPLC conditions**: with a Chiralpak OD-H column (90: 10 hexane: 2-propanol, 0.8 mL/min, 224 nm); tr (minor) = 11.37 min, tr (major) = 15.33 min, 89% ee.

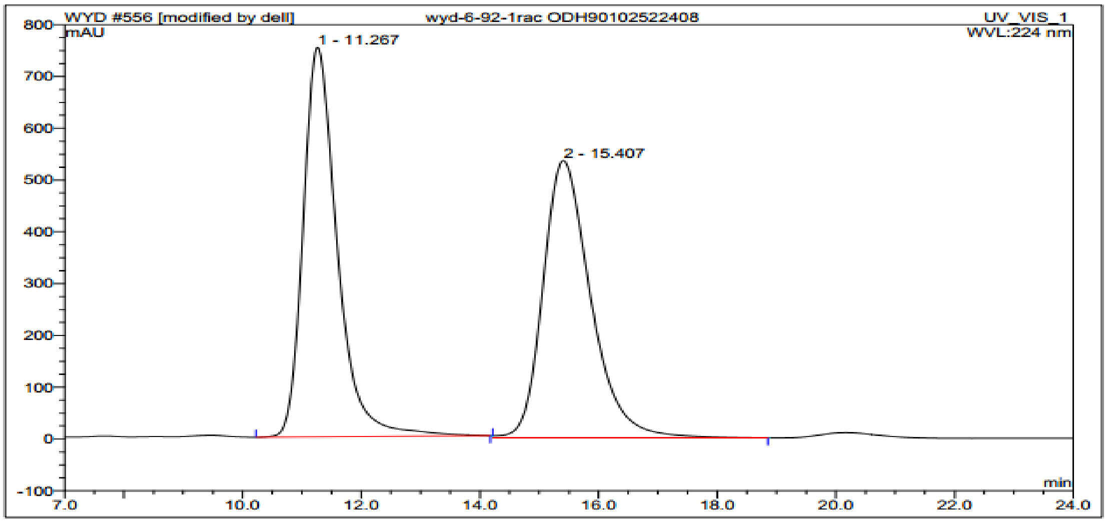

Peak #	Time (min)	Height (mAU)	Area (mAU*min)	Area (%)
1	11.27	752.232	501.769	50.21
2	15.41	535.534	497.490	49.79

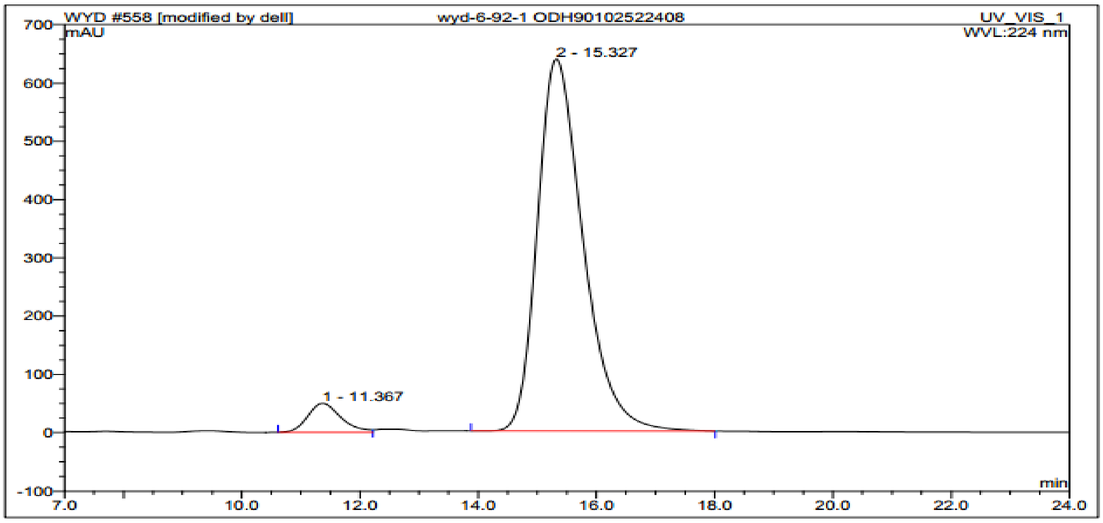

Peak #	Time (min)	Height (mAU)	Area (mAU*min)	Area (%)
1	11.37	49.604	32.171	5.33
2	15.33	638.128	571.422	94.67

35. (*3R,9aS,Z*)-ethyl 2-((2-oxooxazolidin-3-yl)methylene)-3-phenyl-2,3-dihydro-1H-carbazole-9(9aH)-carboxylate.

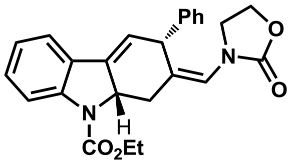

Isolated in 99% yield with 1:0 *Z*/*E* as white solid.

***Z*-isomer, ^{1}H NMR** (400 MHz, $CDCl_3$) δ 7.80 (s, 1 H), 7.24 (t, *J* = 7.2 Hz, 3 H), 7.20-7.10 (m, 4H), 6.90 (t, *J* = 7.6 Hz, 1 H), 6.21 (s, 1 H), 5.80 (t, *J* = 2.8 Hz, 1 H), 4.70-4.60 (m, 1 H), 4.58 (s, 1 H), 4.38-4.19 (m, 2 H), 4.16-3.98 (m, 2 H), 3.43 (q, *J* = 8.8 Hz, 2 H), 3.20 (q, *J* = 8.8 Hz, 1 H), 2.39 (t, *J* = 7.6 Hz, 1 H), 1.34 (t, *J* = 7.2 Hz, 3 H); **^{13}C NMR** (100 MHz, $CDCl_3$) 156.5, 153.6, 144.1, 136.7, 129.5, 128.9, 128.6, 127.6, 127.1, 126.7, 123.0, 122.5, 120.0, 119.2, 118.2, 115.5, 62.1, 61.8, 61.5, 45.6, 42.5, 36.3, 14.6.; **$[\alpha]_D^{20}$** = 239.5 (c = 0.5, $CHCl_3$), **HPLC conditions**: with a Chiralpak AD-H column (80: 20 hexane: 2-propanol, 0.8 mL/min, 224 nm); tr (minor) = 20.31 min, tr (major) = 23.16 min, 77% ee.

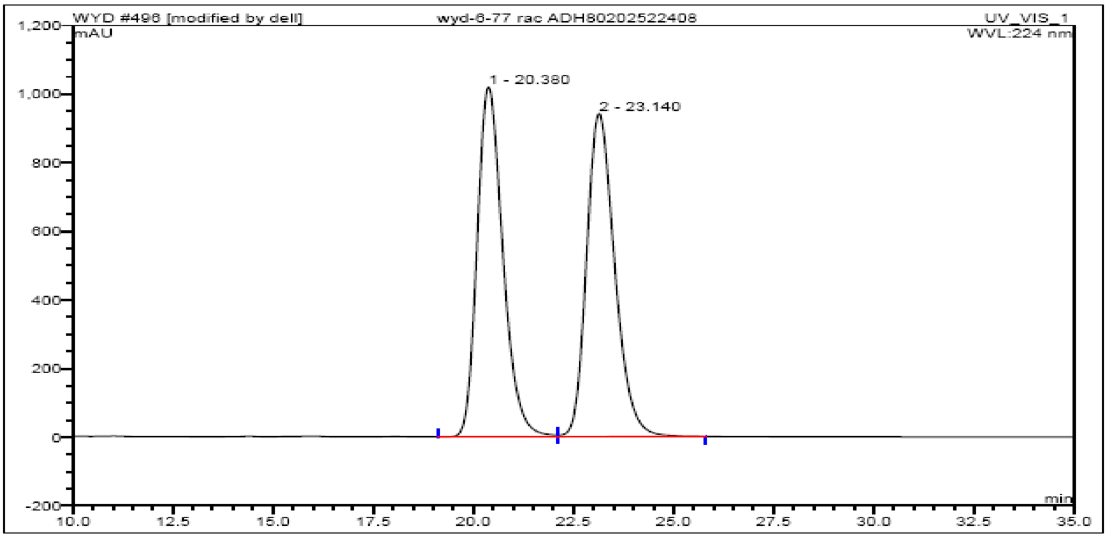

Peak #	Time(min)	Height (mAU)	Area (mAU*min)	Area (%)
1	20.38	1019.429	763.449	49.89
2	23.14	941.343	766.773	50.11

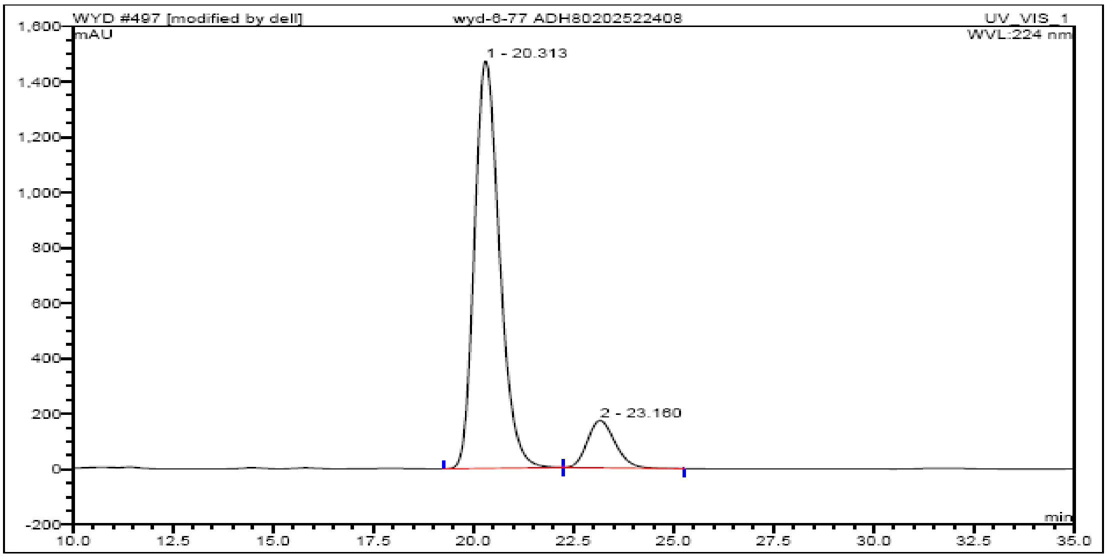

Peak #	Time(min)	Height (mAU)	Area (mAU*min)	Area (%)
1	20.31	1471.917	1071.787	88.70
2	23.16	170.971	136.540	11.30

Product transformation

Synthesis of product 5a.

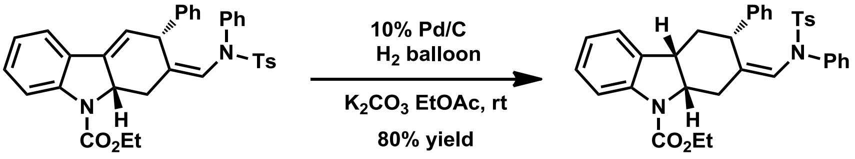

4a **5a, 98% ee**

In a 25 mL Schlenk tube equipped with a football-shaped magnetic stirring bar, 0.1 mmol **4a**, 1.0 eq. K_2CO_3 were added in 2 ml EtOAc, the mixture was then hydrogenated at 1 atm (H_2 balloon) with 10% Pd/C at room temperature for 24 h, The reaction was determined by TLC, after the substrate was consumed, the reaction mixture were filtered over Celite, evaporated under reduced pressure. Purified by flash chromatography on silica gel with petroleum ether/ ethyl acetate (15:1) as the solvent to give the pure product (46.2 mg, 80% yield). The enantiomeric excesses of the products were determined by chiral stationary phase HPLC.

36. (3*R*,9a*S*,*Z*)-ethyl 2-((4-methyl-*N*-phenylphenylsulfonamido)methylene)-3-phenyl-2,3,4,4a-tetrahydro-1H-carbazole-9(9aH)-carboxylate (5a).

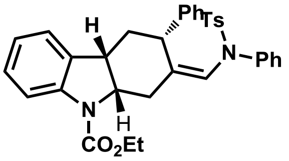

Isolated in 80% yield as white solid.

***Z*-isomer, ^{1}H NMR** (400 MHz, $CDCl_3$) δ 7.83 (s, 1 H), 7.37 (d, *J* = 8.0 Hz, 2 H), 7.25-7.05 (m, 9 H), 7.01 (d, *J* = 7.2 Hz, 3 H), 6.91 (t, *J* = 7.6 Hz, 1 H), 6.60 (d, *J* = 7.6 Hz, 2 H), 6.33 (s, 1 H), 4.56-4.42 (m, 1 H), 4.42-4.30 (m, 2 H), 3.59 (s, 1 H), 3.52-3.42 (m, 1 H), 3.15 (s, 1 H), 2.58 (t, *J* = 11.6 Hz, 1 H), 2.41 (s, 3 H), 2.20-2.10 (m, 1 H), 1.74 (q, *J* = 13.2 Hz, 1 H), 1.44 (t, *J* = 7.2 Hz, 3 H); **^{13}C NMR** (100 MHz, $CDCl_3$) δ 153.8, 143.9, 143.7, 139.6, 138.1, 134.3, 129.3, 128.33, 128.28, 127.8, 127.2, 127.1, 126.8, 126.0, 124.7, 124.1, 122.9, 115.1, 61.6, 60.3, 41.7, 39.3, 37.6, 33.0, 21.5, 14.6; **HRMS** (ESI) calculated for $C_{35}H_{34}N_2NaO_4S$ [M + Na^+]: 601.2131, found: 601.2123. **$[\alpha]_D^{20}$** = 149.9 (c = 0.38, $CHCl_3$); **HPLC conditions**: with a Chiralpak AD-H column (70: 30 hexane: 2-propanol, 0.8 mL/min, 224 nm); tr (minor) = 9.61 min, tr (major) = 11.47 min, 98% ee.

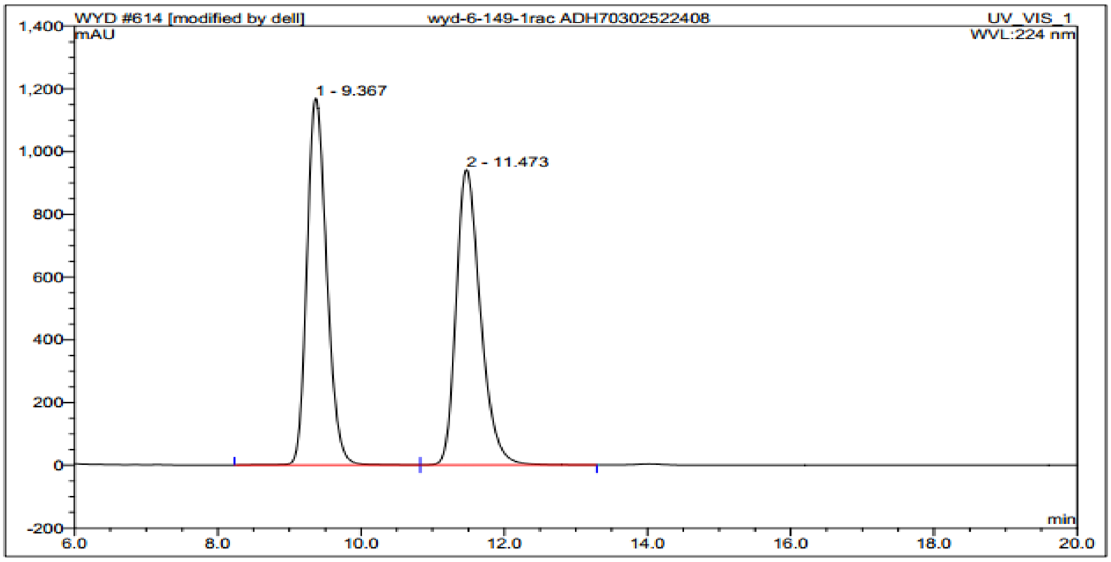

Peak #	Time(min)	Height (mAU)	Area (mAU*min)	Area (%)
1	9.37	1169.976	372.696	50.05
2	11.47	942.289	371.881	49.95

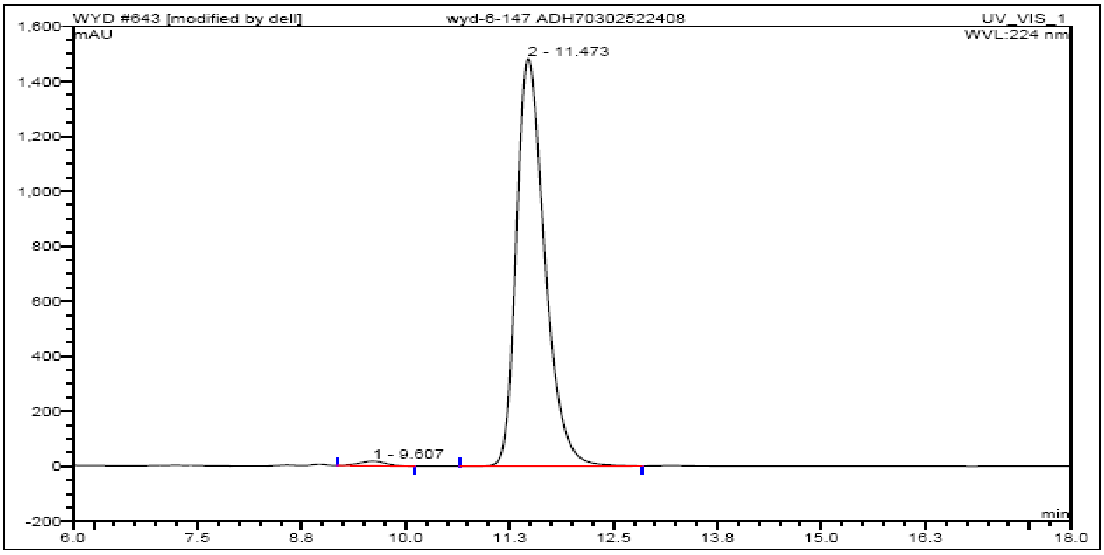

Peak #	Time (min)	Height (mAU)	Area (mAU*min)	Area (%)
1	9.61	16.917	6.234	1.05
2	11.47	1480.659	586.506	98.95

Synthesis of product 6a.

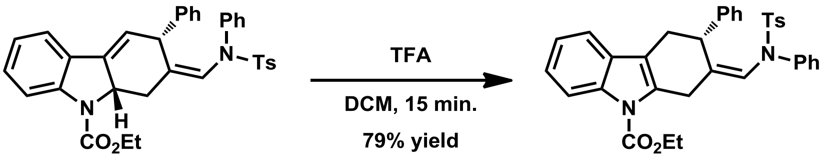

4a

6a, 95% ee

In a 25 mL Schlenk tube equipped with a football-shaped magnetic stirring bar, 4a was dissolved in 2 mL DCM, then added TFA, and the reaction was stirred for 15 min. then the mixture was treated with saturated aqueous $NaHCO_3$ solution, extracted three times with DCM. The combined organic layers were washed with the saturated aqueous NaCl solution, dried over Na_2SO_4, and finally evaporated under reduced pressure. Purified by flash chromatography on silica gel with petroleum ether/ethyl acetate (20:1) as the solvent to give the pure product (45.5 mg, 79% yield). The enantiomeric excesses of the products were determined by chiral stationary phase HPLC.

37. (*R*, *Z*)-ethyl 2-((4-methyl-N-phenylphenylsulfonamido)methylene)-3-phenyl-3,4-dihydro-1H-carbazole-9(2H)-carboxylate (6a).

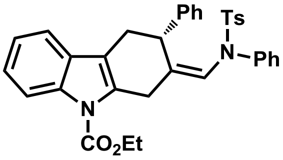

Isolated in 79% yield as white solid.

***Z*-isomer, ^{1}H NMR** (400 MHz, $CDCl_3$) δ 8.10 (d, *J* = 7.6 Hz, 1 H), 7.50 (d, *J* = 8.0 Hz, 1 H), 7.43 (d, *J* = 8.0 Hz, 1 H), 7.36-7.20 (m, 10 H), 7.11-7.04 (m, 3 H), 6.98-6.90 (m, 2 H), 6.32 (s, 1 H), 4.70 (d, *J* = 5.6 Hz, 1 H), 4.46-4.37 (m, 2 H), 3.69 (d, *J* = 19.2 Hz, 1 H), 3.30 (d, *J* = 19.2 Hz, 1 H), 3.13 (d, *J* = 16.8 Hz, 1 H), 2.70-2.60 (m, 1 H), 2.42 (s, 3 H), 1.43 (t, *J* = 7.2 Hz, 3 H); **^{13}C NMR** (100 MHz, $CDCl_3$) δ 151.8, 143.9, 142.4, 141.3, 140.8, 136.0, 134.1, 132.8, 129.5, 129.1, 128.3, 128.01, 127.2, 127.1, 127.1, 126.3, 123.9, 122.9, 122.3, 117.9, 115.9, 115.6, 62.9, 37.7, 29.9, 23.7, 21.6, 14.4; **HRMS** (ESI) calculated for $C_{35}H_{32}N_2NaO_4S$ [M + Na$^+$]: 599.1975, found: 599.1884. **$[\alpha]_D^{20}$** = 5.2 (c = 0.5, $CHCl_3$); **HPLC conditions**: with a Chiralpak AD-H column (70: 30 hexane: 2-propanol, 0.8 mL/min, 224 nm); tr (minor) = 8.05 min, tr (major) = 8.73 min, 95% ee.

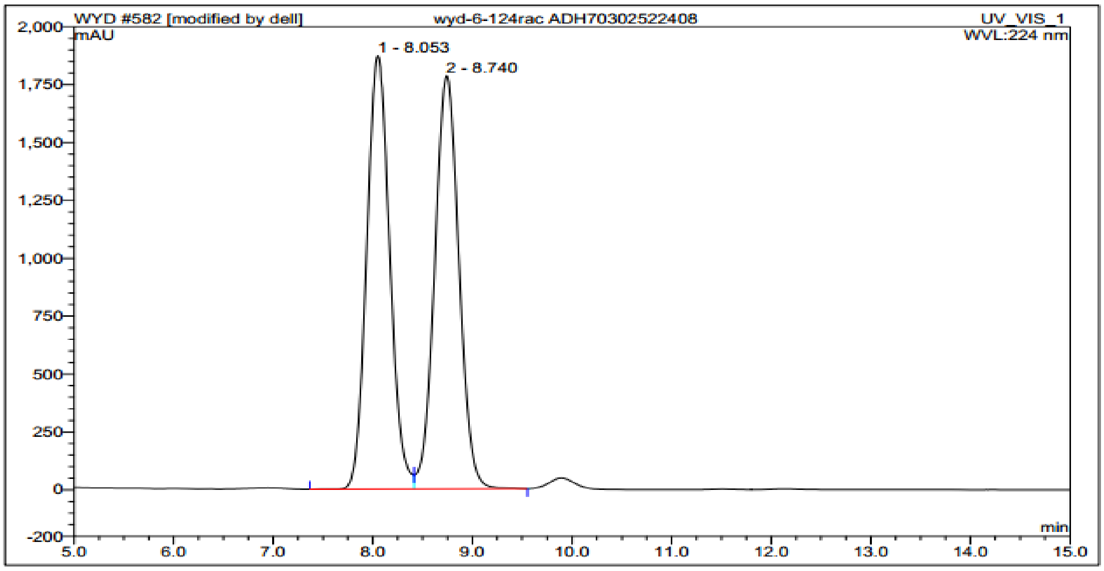

Peak #	Time (min)	Height (mAU)	Area (mAU*min)	Area (%)
1	8.05	1870.955	499.932	49.85
2	8.74	1783.602	502.857	50.15

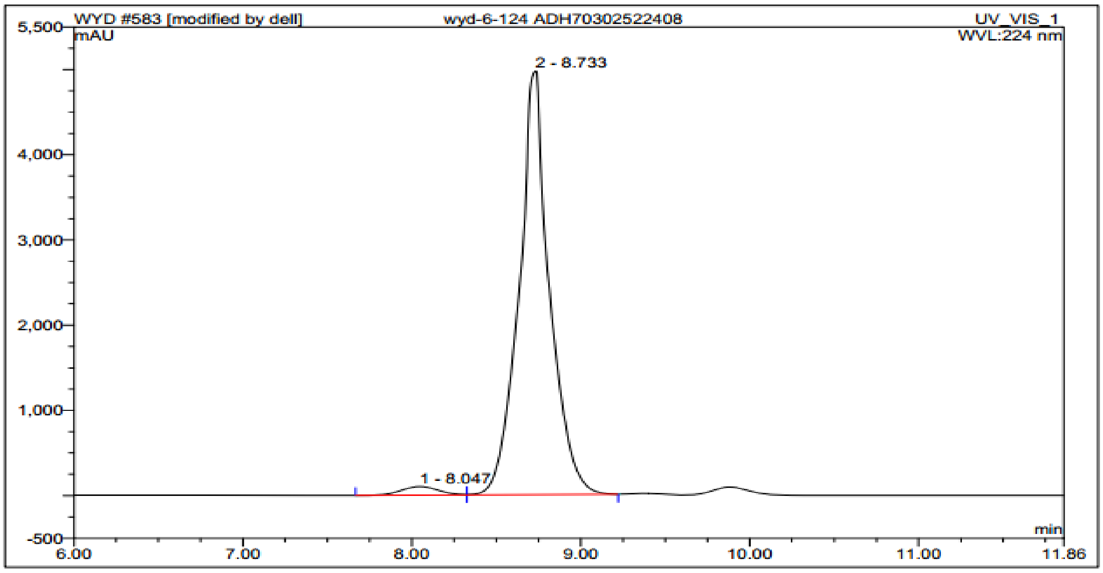

Peak #	Time (min)	Height (mAU)	Area (mAU*min)	Area (%)
1	8.05	101.701	25.531	2.39
2	8.7	4973.526	1041.170	97.61

Synthesis of product.

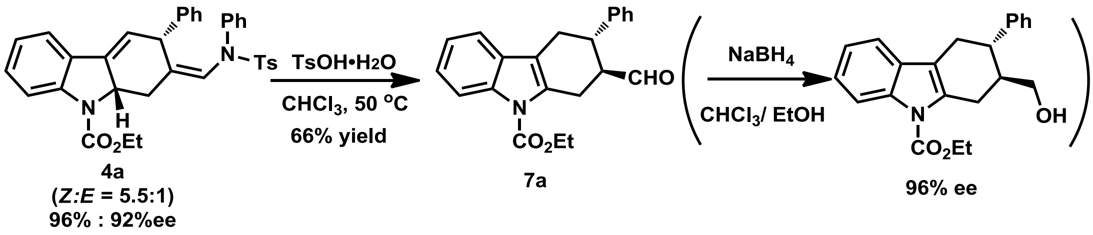

In a 10 mL Schlenk tube equipped with a football-shaped magnetic stirring bar, **4a** (0.1 mol, *Z:E* = 5.5:1) was dissolved in 1 mL $CHCl_3$, then added 20 mol% $TsOH•H_2O$, the reaction was stirred at 50 ^{o}C for 4 h. then the

mixture was treated with saturated aqueous $NaHCO_3$ solution, extracted three times with DCM. The combined organic layers were washed with the saturated aqueous NaCl solution, dried over Na_2SO_4, and finally evaporated under reduced pressure. Purified by flash chromatography on silica gel with petroleum ether/ethyl acetate (20:1) as the solvent to give the pure product (23.0 mg, 66% yield). Enantiomeric excess of corresponding alcohol after reduction by $NaBH_4$ was determined by HPLC equipped with chiral column.

38. (2*S*,3*S*)-ethyl 2-formyl-3-phenyl-3,4-dihydro-1H-carbazole-9(2H)-carboxylate (7a) .

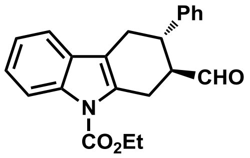

Isolated in 66% yield as white solid.

^{1}H NMR (400 MHz, $CDCl_3$) δ 9.59 (d, *J* = 2.0 Hz, 1 H), 8.16 (d, *J* = 8.0 Hz, 1 H), 7.40-7.20 (m, 8 H), 4.48 (q, *J* = 7.2 Hz, 2 H), 3.45-3.36 (m, 1 H), 3.36-3.29 (m, 1H), 3.25-3.16 (m, 1 H), 3.12-3.02 (m, 1 H), 2.95-2.85 (m, 1 H), 1.47 (t, *J* = 7.2 Hz, 3 H); **^{13}C NMR** (125 MHz, $CDCl_3$) 203.2, 151.8, 142.4, 136.0, 132.6, 128.9, 127.5, 127.1, 124.2, 122.9, 117.7, 116.3, 115.6, 63.0, 52.2, 40.3, 28.0, 24.7, 14.4; **MS** (EI): m/z (%): 347 (100), **HRMS** (EI) calculated for [$C_{22}H_{21}NO_3$]: 347.1521, found: 347.1519. **$[\alpha]_D^{20}$** = -19.0 (c = 0.25, $CHCl_3$), **HPLC conditions**: with a Chiralpak AD-H column (70: 30 hexane: 2-propanol, 0.8 mL/min, 210 nm); tr (minor) = 10.03 min, tr (major) = 13.04 min, 96% ee.

Chromatogram and Results

Injection Details

Injection Name:	wyd-7-53rac ADH70300825	*Run Time (min):*	18.76
Vial Number:	RD2	*Injection Volume:*	20.00
Injection Type:	Unknown	*Channel:*	UV_VIS_1
Calibration Level:		*Wavelength:*	210.0
Instrument Method:	30min-3070-25-210254	*Bandwidth:*	4
Processing Method:	wyd-1	*Dilution Factor:*	1.0000
Injection Date/Time:	14/1月/15 20:26	*Sample Weight:*	1.0000

Chromatogram

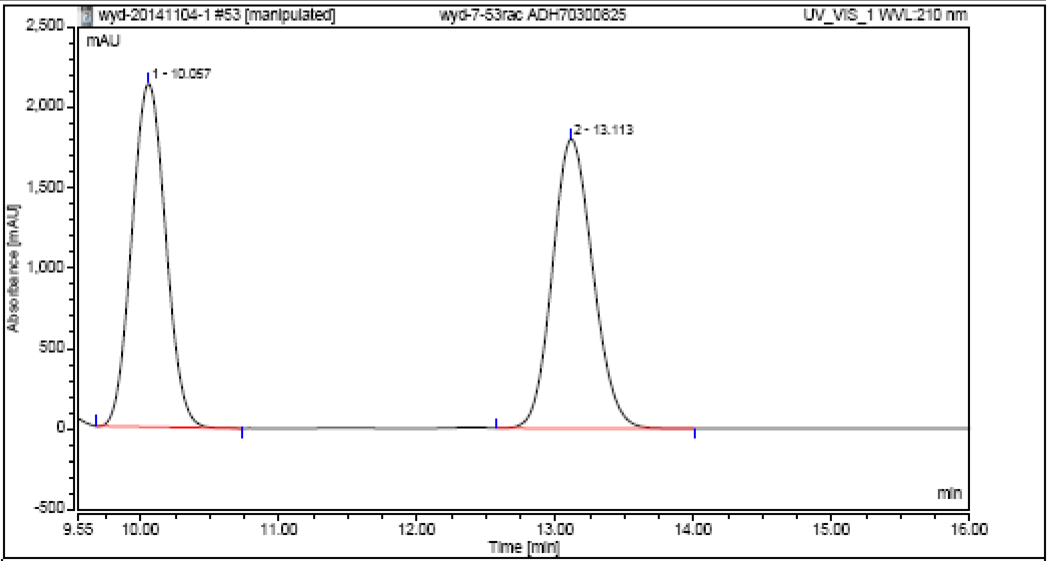

Integration Results

No.	Peak Name	Retention Time min	Area mAU*min	Height mAU	Relative Area %	Relative Height %	Amount n.a.
1		10.057	604.604	2135.947	49.71	54.28	n.a.
2		13.113	611.609	1798.855	50.29	45.72	n.a.
**Total:**			**1216.213**	**3934.802**	**100.00**	**100.00**	

Chromatogram and Results

Injection Details

Injection Name:	wyd-7-60 ADH70300825	*Run Time (min):*	15.12
Vial Number:	RB2	*Injection Volume:*	20.00
Injection Type:	Unknown	*Channel:*	UV_VIS_1
Calibration Level:		*Wavelength:*	210.0
Instrument Method:	30min-3070-25-210254	*Bandwidth:*	4
Processing Method:	wyd-1	*Dilution Factor:*	1.0000
Injection Date/Time:	15/1月/15 22:25	*Sample Weight:*	1.0000

Chromatogram

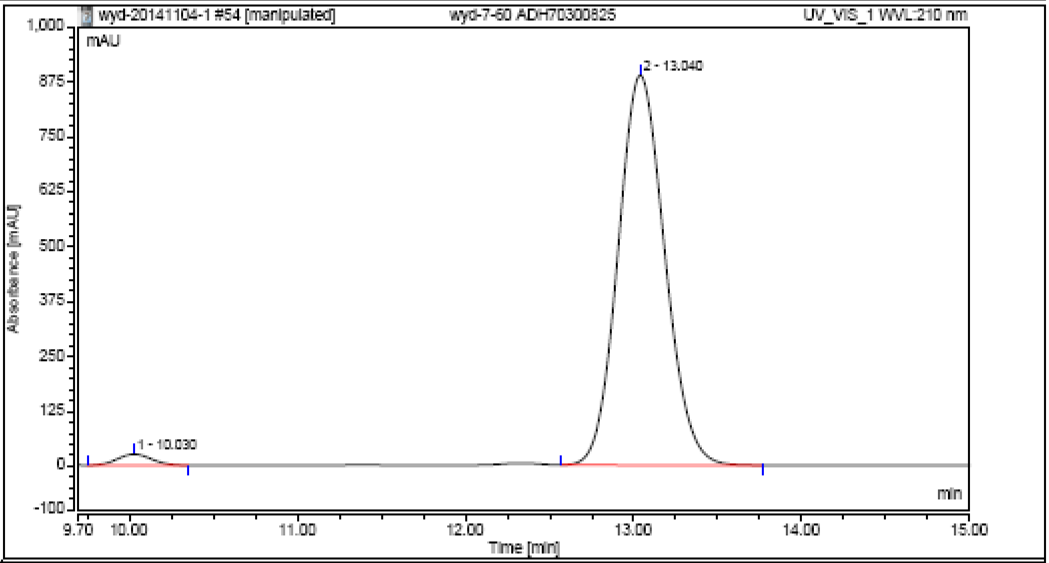

Integration Results

No.	Peak Name	Retention Time min	Area mAU*min	Height mAU	Relative Area %	Relative Height %	Amount n.a.
1		10.030	6.034	25.590	2.07	2.80	n.a.
2		13.040	285.626	888.093	97.93	97.20	n.a.
Total:			291.659	913.683	100.00	100.00	

Synthesis of product.

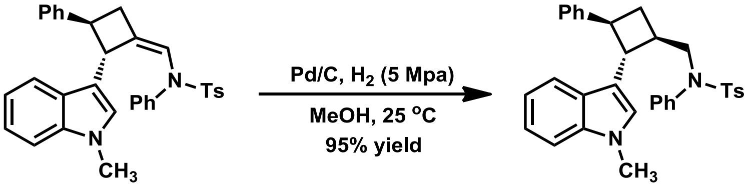

3a (96% ee) **8a, 96% ee**

In a 10 mL test tube equipped with a football-shaped magnetic stirring bar, **3a** (0.1 mmol) was dissolved in 2 mL MeOH, the mixture was then hydrogenated at 50 atm with 10% Pd/C at 25 °C for 24 h, The reaction was determined by TLC, after the substrate was consumed, the reaction mixture were filtered over Celite, evaporated under reduced pressure. And get the pure product (49.4 mg, 95% yield). The enantiomeric excesses of the products were determined by chiral stationary phase HPLC.

39. 4-methyl-N-(((1*R*,2*R*,3*S*)-2-(1-methyl-1H-indol-3-yl)-3-phenylcyclobutyl)methyl)-N-phenylbenzenesulfonamide (8a) .

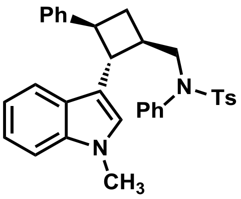

Isolated in 95% yield as white solid.

^{1}H NMR (500 MHz, $CDCl_3$) δ 7.47 (d, *J* = 8.5 Hz, 2 H), 7.36 (d, *J* = 7.5 Hz, 1 H), 7.32-7.16 (m, 12 H), 7.02 (t, *J* = 7.5 Hz, 1 H), 6.93-6.88 (m, 2 H), 6.84 (s, 1 H), 3.84-3.72 (m, 2 H), 3.75 (s, 3H), 3.50 (q, *J* = 10.0 Hz, 1 H), 3.36 (t, *J* = 9.5 Hz, 1 H), 2.67-2.56 (m, 1 H), 2.48-2.34 (m, 1 H), 2.43 (s, 3 H), 1.90 (q, *J* = 10.0 Hz, 1 H)； **^{13}C**

NMR (125 MHz, $CDCl_3$) 144.2, 143.2, 139.4, 137.2, 135.2, 129.3, 128.8, 128.7, 128.2, 127.67, 127.65, 127.2, 126.6, 126.0, 125.9, 121.4, 119.5, 118.6, 116.6, 109.1, 55.3, 44.8, 44.7, 38.4, 32.6, 30.6, 21.5.; **MS** (EI): m/z (%): 520 (M^+, 4.41), 170 (100), **HRMS** (EI) calculated for [$C_{33}H_{32}N_2O_2S$]: 520.2185, found: 520.2182. $[\alpha]_D^{20}$ = 32.8 (c = 0.25, $CHCl_3$), **HPLC conditions**: with a Chiralpak AD-H column (95: 05 hexane: 2-propanol, 0.8 mL/min, 220 nm); tr (minor) = 35.93 min, tr (major) = 33.68 min, 96% ee.

Chromatogram and Results

Injection Details

Injection Name:	wyd-7-48-2 ADH95050825	*Run Time (min):*	50.00
Vial Number:	RA1	*Injection Volume:*	20.00
Injection Type:	Unknown	*Channel:*	UV_VIS_1
Calibration Level:		*Wavelength:*	210.0
Instrument Method:	50min-0595-25-08-210254	*Bandwidth:*	4
Processing Method:	wyd-1	*Dilution Factor:*	1.0000
Injection Date/Time:	09/1月/15 16:25	*Sample Weight:*	1.0000

Chromatogram

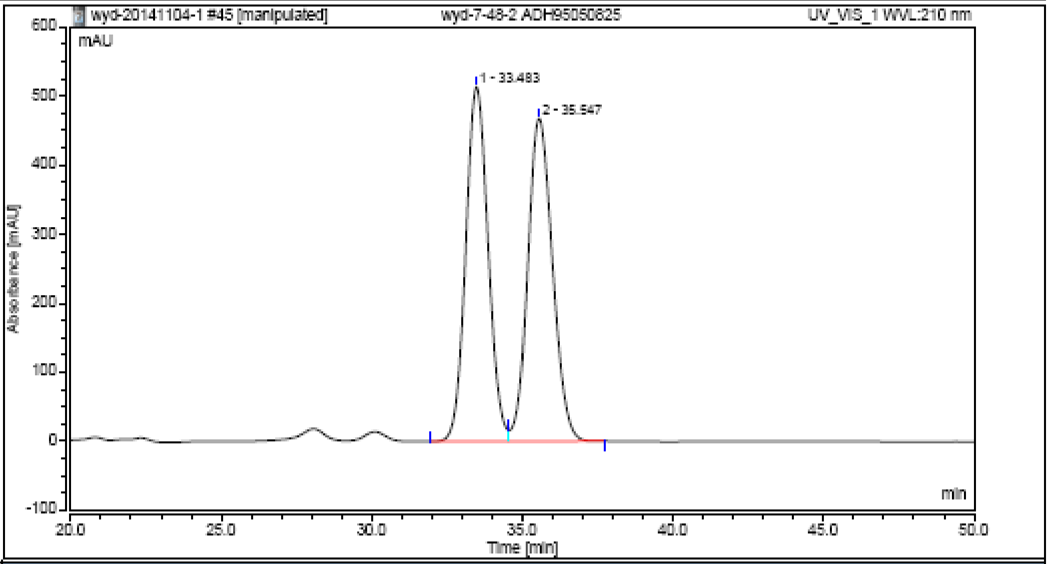

Integration Results

No.	Peak Name	Retention Time min	Area mAU*min	Height mAU	Relative Area %	Relative Height %	Amount n.a.
1		33.483	443.036	513.712	49.96	52.34	n.a.
2		35.547	443.730	467.816	50.04	47.66	n.a.
Total:			886.766	981.527	100.00	100.00	

Chromatogram and Results

Injection Details

Injection Name:	wyd-7-48-3-zpc ADH95050825	*Run Time (min):*	50.00
Vial Number:	RA2	*Injection Volume:*	20.00
Injection Type:	Unknown	*Channel:*	UV_VIS_1
Calibration Level:		*Wavelength:*	220.0
Instrument Method:	50min-0595-25-08-210254	*Bandwidth:*	4
Processing Method:	wyd-1	*Dilution Factor:*	1.0000
Injection Date/Time:	12/1月/15 09:23	*Sample Weight:*	1.0000

Chromatogram

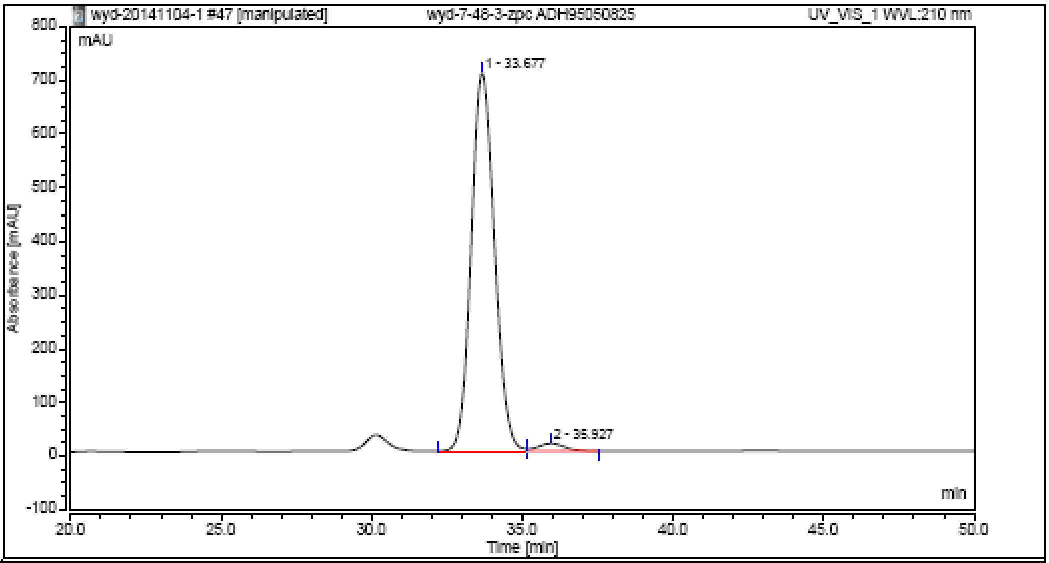

Integration Results

No.	Peak Name	Retention Time min	Area mAU*min	Height mAU	Relative Area %	Relative Height %	Amount n.a.
1		33.677	630.376	703.704	97.89	98.09	n.a.
2		35.927	13.558	13.676	2.11	1.91	n.a.
Total:			643.934	717.381	100.00	100.00	

Section 1: Computational methods

The Density Functional Theory (DFT) calculations were carried out using the Gaussian 09 program package [1]. The molecular geometrical structures were optimized using the M06 [2] functional combined with the Lanl2dz with the effective core potential (ECP) [3] and 6-31G* basis sets. The Lanl2dz basis set with the ECP was used to describe the Au atom and the other nonmetal atoms C, O, P, H were described by the 6-31G* basis set. The frequency calculations were performed on the optimized structures obtained in the gas phase to verify the minima or transition states on potential surfaces. The solvent effects of dichloromethane were considered by performing the calculations of solvation corrections using the polarizable continuum model (PCM) [4] model. The intrinsic reaction coordinate (IRC) [5] calculations were also performed to assure that the transition states connected the correct intermediates in the forward and reverse directions.

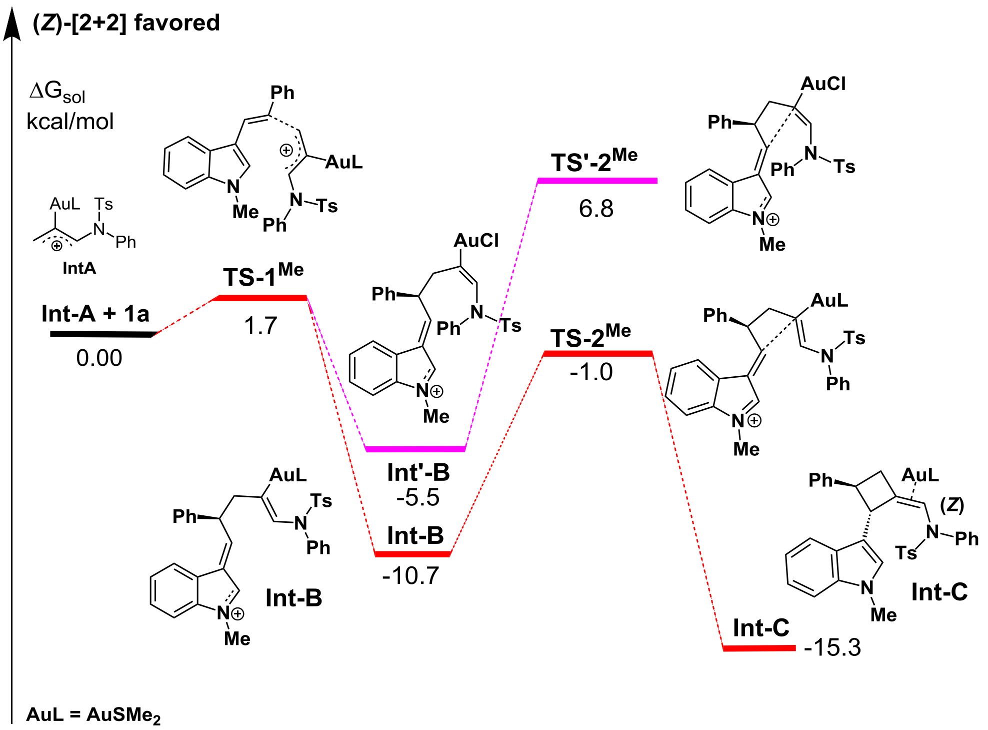

Figure 2': The free energy profiles ΔGsol for the (*Z*) or (*E*)-selectivity of the [2+2] cycloaddition product.

The complete (*Z*)-selectivity of the [2+2] cycloaddition product can also be easily explained by computational calculations. The Int'-C is formed from the Int'-B though TS'-2^{Me}. Because the Int'-B is less stable than Int-B ($\Delta E_{\text{Int-B - Int'-B}}$ = -5.2 kcal mol^{-1}), and in the second transition state, the ring closure of Int'-B may be disfavored by the stereoelectronic effect ($E_{\text{TS-2Me}}$ = -1.0 kcal mol^{-1} vs $E_{\text{TS'-2Me}}$ =6.8 kcal mol^{-1}).

References

[1] Gaussian 09, Revision **D.01**, M. J. Frisch, G. W. Trucks, H. B. Schlegel, G. E. Scuseria, M. A. Robb, J. R. Cheeseman, G. Scalmani, V. Barone, B. Mennucci, G. A. Petersson, H. Nakatsuji, M. Caricato, X. Li, H. P. Hratchian, A. F. Izmaylov, J. Bloino, G. Zheng, J. L. Sonnenberg, M. Hada, M. Ehara, K. Toyota, R. Fukuda, J. Hasegawa, M. Ishida, T. Nakajima, Y. Honda, O. Kitao, H. Nakai, T. Vreven, J. A. Montgomery, Jr., J. E. Peralta, F. Ogliaro, M. Bearpark, J. J. Heyd, E. Brothers, K. N. Kudin, V. N. Staroverov, R. Kobayashi, J. Normand, K. Raghavachari, A. Rendell, J. C. Burant, S. S. Iyengar, J. Tomasi, M. Cossi, N. Rega, J. M. Millam, M. Klene, J. E. Knox, J. B. Cross, V. Bakken, C. Adamo, J. Jaramillo, R. Gomperts, R. E. Stratmann, O. Yazyev, A. J. Austin, R. Cammi, C. Pomelli, J. W. Ochterski, R. L. Martin, K. Morokuma, V. G. Zakrzewski, G. A. Voth, P. Salvador, J. J. Dannenberg, S. Dapprich, A. D. Daniels, Ö. Farkas, J. B. Foresman, J. V. Ortiz, J. Cioslowski, and D. J. Fox, Gaussian, Inc., Wallingford CT, **2009**.

[2] Y. Zhao, D. G. Truhlar, Theor. Chem. Acc. **2008**, *120*, 215.

[3] a) P. J. Hay, W. R. Wadt, J. Chem. Phys. **1985**, *82*, 299; b) W. R. Wadt, P. J. Hay, J. Chem. Phys. **1985**, *82*, 284.

[4] G. Scalmani, M. J. Frisch, J. Chem. Phys. **2010**, *132*, 114110.

[5] K. Fukui, Acc. Chem. Res. **1981**, *14*, 363.

Section 2: Cartesian coordinates of structures in Figure 1

Structure and coordinates of **Int-A**

C	2.19348934	2.46904136	-2.89367322
C	2.46707834	3.19794036	-4.01177522
H	2.41525734	2.77305636	-5.01431722
C	2.24962234	3.23274136	-1.69467422
H	2.23670234	4.32712836	-1.76130222
N	2.31921634	2.77193236	-0.45133822
S	2.27755834	3.95430136	0.90176478
O	1.88554134	5.19679536	0.26346978
O	1.48592434	3.31413736	1.92749578
H	2.82185334	4.23184836	-3.95794022
Au	1.53546634	0.51689936	-2.90143122
C	3.96673834	3.99574536	1.37301478
C	4.39633134	3.17611936	2.41372578
C	4.84701134	4.81006436	0.65922778
C	5.74306234	3.18744336	2.74926578
H	3.68325834	2.55331536	2.95005478
C	6.18636434	4.79927536	1.01131678
H	4.48098134	5.45368036	-0.13940622
C	6.65303434	3.99395736	2.05962278
H	6.09757434	2.56088436	3.56657878
H	6.88965034	5.43387536	0.47311178
C	2.57762834	1.40513736	-0.09366222
C	3.85565334	0.88885336	-0.30460822
C	1.57107434	0.64384036	0.49259678
C	4.12088634	-0.42538964	0.06124478
H	4.62437634	1.51846736	-0.75273222
C	1.84745434	-0.67100464	0.85123178
H	0.59115134	1.08529536	0.65610478
C	3.11807034	-1.20294164	0.64056678
H	5.11486834	-0.83975264	-0.09384922
H	1.06377034	-1.27431564	1.30627578
H	3.33387334	-2.22747864	0.94037278
C	8.09871834	4.02199536	2.44393478
H	8.37583034	3.15148436	3.04856678
H	8.74893934	4.04946336	1.56107078
H	8.32518534	4.91927136	3.03619578
S	0.69344934	-1.77647164	-2.86657522
C	2.22644634	-2.75366364	-2.77711522
H	2.67388434	-2.56393464	-1.79557022
H	1.98310734	-3.81700664	-2.87369222
H	2.92387634	-2.44555864	-3.56300622
C	0.23929934	-2.10155464	-4.59928722
H	-0.00708966	-3.16245064	-4.71256322

H	-0.64246966	-1.49681764	-4.83114022
H	1.05923034	-1.82413864	-5.26940522

Structure and coordinates of **1a**

C	3.89122200	-1.30747000	-0.07856200
C	2.37543000	0.18140700	2.18294000
C	2.21696000	-1.12105900	1.74916600
C	2.98691300	-1.84273100	0.77352100
H	4.07673500	-0.22903200	-0.01937700
H	2.77365800	-2.91372000	0.70952700
C	0.59656700	-0.59446700	3.29215800
C	-0.49995200	-0.75304300	4.14208400
C	-1.14893800	-1.97738400	4.11733900
C	-0.71215100	-3.01636500	3.27632500
C	0.38670800	-2.85678200	2.44937400
C	1.06501900	-1.63040300	2.45278600
H	-0.83026600	0.04678700	4.80483300
H	-2.01315900	-2.13945200	4.75786500
H	-1.25336400	-3.96037800	3.27421200
H	0.71667000	-3.67822300	1.81280200
N	1.41267900	0.50183100	3.10500800
H	3.14420300	0.90719800	1.93411800
C	4.66207800	-2.00614200	-1.09885700
C	5.47302000	-1.25418500	-1.96065800
C	4.60801000	-3.39629000	-1.28650700
C	6.20301000	-1.86179100	-2.97437300
H	5.53151200	-0.17311400	-1.82214700
C	5.33589400	-4.00303700	-2.29899300
H	4.00061500	-4.01290200	-0.62419900
C	6.13593800	-3.23996300	-3.14850200
H	6.82900700	-1.25712800	-3.62810900
H	5.28514600	-5.08311400	-2.42475300
H	6.70701600	-3.72175900	-3.93957600
C	1.25118300	1.77112000	3.76818400
H	0.23529800	2.16057800	3.60857600
H	1.42863500	1.68214100	4.84782200
H	1.96706800	2.48856300	3.35302900

Structure and coordinates of **1e**

C	-2.60532900	0.63720600	-0.13333700
C	-1.56106500	2.11428100	-2.58282100
C	-1.66151200	2.59514000	-1.30041600
C	-2.19063400	1.92520100	-0.14260300
H	-2.58788900	0.09010600	-1.08040000

H	-2.19387500	2.50379100	0.78413900
C	-0.56200000	4.15422600	-2.60932900
C	0.11226000	5.32995800	-2.93065900
C	0.32746600	6.23883400	-1.90349700
C	-0.10770900	5.98979900	-0.59464800
C	-0.77933000	4.81958200	-0.28142700
C	-1.01548600	3.89151300	-1.29949300
H	0.45091600	5.53206500	-3.94147500
H	0.84675700	7.16923400	-2.12508200
H	0.07917400	6.72953400	0.18118600
H	-1.11482200	4.62552400	0.73691800
C	-0.60463900	2.71444700	-4.71713700
O	-0.04960200	3.74080600	-5.34570900
O	-0.82465200	1.62253400	-5.18484000
N	-0.91502300	3.03851200	-3.39122200
H	-1.93615100	1.20443800	-3.03587200
C	0.31906800	3.50346200	-6.72180800
C	0.93641700	4.77477200	-7.23646800
H	-0.58358000	3.21792600	-7.27538600
H	1.01176400	2.65334500	-6.75241100
H	1.23179200	4.65070600	-8.28415200
H	0.22517600	5.60678200	-7.17801100
H	1.82992000	5.04036800	-6.65875600
C	-3.06890500	-0.14262100	1.00223900
C	-3.47202000	-1.47060900	0.78331300
C	-3.08736600	0.34775800	2.31754700
C	-3.88341200	-2.28010200	1.83399000
H	-3.46507100	-1.86152900	-0.23648300
C	-3.49873000	-0.46088500	3.36846000
H	-2.76511100	1.36823100	2.52567900
C	-3.89940600	-1.77620500	3.13280900
H	-4.19850900	-3.30395300	1.63856000
H	-3.49485900	-0.06351700	4.38159000
H	-4.22524800	-2.40343000	3.96051700

Structure and coordinates of **TS-1^{Me}**

C	3.54001200	-1.33003800	-0.20256200
C	0.83429000	-0.18693100	-0.90284800
C	1.89777000	-0.86348300	-1.53195600
C	2.06399200	-0.10630400	2.15437500
C	1.99712300	-1.43773600	1.72632900
C	2.79411500	-2.02894200	0.73110000
H	3.71435400	-0.26716900	-0.00693600
H	2.71420700	-3.11396300	0.62161100

C	0.43100600	-1.04126600	3.36084100
C	-0.60739000	-1.26248300	4.26072600
C	-1.15800600	-2.53575700	4.28072100
C	-0.69052500	-3.54209000	3.42340400
C	0.34904800	-3.31015000	2.53528400
C	0.93362200	-2.04248300	2.50647300
H	-0.97681900	-0.47765600	4.91945400
H	-1.97687300	-2.75286100	4.96255300
H	-1.15974500	-4.52292800	3.45128900
H	0.68874400	-4.10084800	1.86747000
C	-0.19710000	-1.02823200	-0.52176300
H	-0.09189500	-2.10742900	-0.68160800
N	-1.39353200	-0.72012900	0.03544600
S	-2.53177600	-2.02251500	0.37297800
O	-1.80519700	-3.24084600	0.06758500
O	-3.07229700	-1.73482400	1.68420000
N	1.15502600	0.12808500	3.12008300
H	2.75793200	0.67460900	1.85995200
Au	0.92457700	1.81121000	-0.43058000
C	4.49487800	-1.94367800	-1.13679700
C	5.48054400	-1.13935200	-1.72032000
C	4.43376300	-3.29436400	-1.50222100
C	6.39435900	-1.66987500	-2.62244400
H	5.53429600	-0.08367100	-1.44806700
C	5.34674600	-3.82559300	-2.40262200
H	3.66496200	-3.94288400	-1.08086900
C	6.33205300	-3.01669300	-2.96450200
H	7.15913500	-1.02944300	-3.05765700
H	5.28902900	-4.87890100	-2.67037300
H	7.04650200	-3.43581200	-3.67008400
C	-3.77397200	-1.73819200	-0.84120100
C	-4.89515800	-0.98742700	-0.49879300
C	-3.58661300	-2.23333700	-2.13105200
C	-5.85014800	-0.73852500	-1.47490800
H	-5.01187300	-0.61411900	0.51680200
C	-4.55388200	-1.97150500	-3.08883300
H	-2.70653600	-2.82863500	-2.36953100
C	-5.69848700	-1.22757800	-2.77540300
H	-6.73756500	-0.15897500	-1.22282100
H	-4.43042700	-2.36044500	-4.09916800
C	-1.88619300	0.60750300	0.24229600
C	-2.34980300	1.34593400	-0.84700700
C	-1.95395000	1.11811400	1.53612200
C	-2.86059500	2.62230300	-0.63657200

H	-2.30905300	0.90820500	-1.84444100
C	-2.46216600	2.39757700	1.73633900
H	-1.62543700	0.49144600	2.36527800
C	-2.91169200	3.14953600	0.65325400
H	-3.23770100	3.19824700	-1.48017500
H	-2.52768700	2.80287400	2.74483800
H	-3.32466700	4.14353700	0.81726200
C	-6.75432700	-0.99212800	-3.80995700
H	-6.32049600	-0.85633000	-4.80784400
H	-7.43511400	-1.85260000	-3.86943900
H	-7.36330500	-0.11174700	-3.57518300
H	2.62470300	-0.31145100	-2.12720000
H	1.76861900	-1.90050200	-1.85866200
S	1.03269600	4.18978800	0.16761800
C	2.74496800	4.65158000	-0.24562400
H	3.00702000	4.29570200	-1.24731500
H	2.84694600	5.74035300	-0.18726900
H	3.40534300	4.18660200	0.49216300
C	0.18384900	4.97901400	-1.23540200
H	0.28113700	6.06657000	-1.15182400
H	0.60300000	4.62543000	-2.18323000
H	-0.87086900	4.69527100	-1.17498000
C	0.95192000	1.38783200	3.79883000
H	-0.01523800	1.82708100	3.51866400
H	0.98340800	1.24255100	4.88471000
H	1.74633500	2.08210400	3.50906700

Structure and coordinates of **Int-B**

C	3.14109900	-1.21726300	-0.34336700
C	0.85029100	-0.10150500	-0.70707100
C	1.91232400	-0.95357000	-1.32008400
C	1.44470200	-0.74254400	2.12827200
C	1.89568300	-2.03324700	1.68826800
C	2.71059600	-2.24897500	0.62275600
H	3.32240600	-0.25629400	0.16299400
H	2.95999500	-3.28496500	0.37602900
C	0.34256400	-2.24786300	3.36806000
C	-0.54103600	-2.81515600	4.26883200
C	-0.57034200	-4.20569000	4.31317100
C	0.25290800	-4.97385500	3.48538800
C	1.13025300	-4.37982600	2.58244200
C	1.17597100	-2.99315100	2.52540300
H	-1.19446200	-2.21417000	4.89773100
H	-1.25073100	-4.70197100	5.00108900

H	0.20326400	-6.05868300	3.54585300
H	1.76155300	-4.99274200	1.94094000
C	-0.26284900	-0.72188700	-0.25986900
H	-0.36649600	-1.80834800	-0.33650200
N	-1.35153900	-0.11511200	0.39876100
S	-2.53542100	-1.14130700	1.10718000
O	-1.92542600	-2.46038300	1.16670400
O	-2.97277400	-0.46177900	2.31919000
N	0.55895300	-0.87540900	3.09484900
H	1.76459500	0.23932900	1.79338200
Au	1.17044800	1.92250600	-0.52255400
C	4.37947400	-1.60124500	-1.11438300
C	5.51124600	-0.78872000	-1.06020100
C	4.41040000	-2.74812400	-1.91120700
C	6.65449000	-1.11341800	-1.78377400
H	5.49849500	0.10779100	-0.43822300
C	5.55203000	-3.07565100	-2.63371200
H	3.53258200	-3.39384200	-1.97856200
C	6.67761000	-2.25902300	-2.57132000
H	7.53087700	-0.47029200	-1.72894300
H	5.56196300	-3.97211900	-3.25092800
H	7.57071900	-2.51656500	-3.13709000
C	-3.85598500	-1.13478300	-0.05855300
C	-4.87167800	-0.19083600	0.06156400
C	-3.78950700	-2.00235200	-1.14831700
C	-5.83919300	-0.12290500	-0.93194200
H	-4.89680400	0.47451500	0.92259800
C	-4.76727800	-1.91748700	-2.12718600
H	-2.99132600	-2.73988300	-1.21486600
C	-5.80271500	-0.97932600	-2.03544100
H	-6.64240800	0.60860500	-0.85025400
H	-4.73619500	-2.59350500	-2.98142200
C	-1.80719200	1.19680400	0.04340900
C	-2.10654900	1.48350000	-1.29205800
C	-2.00088400	2.16349900	1.02809400
C	-2.58999300	2.73740500	-1.63785400
H	-1.95532100	0.71360400	-2.04782600
C	-2.48990100	3.41910700	0.67547600
H	-1.78676000	1.91784700	2.06557300
C	-2.78717800	3.70651100	-0.65331400
H	-2.83294600	2.95488000	-2.67645500
H	-2.64746600	4.17127300	1.44606300
H	-3.18637100	4.68332300	-0.92283700
C	-6.86276200	-0.91686500	-3.09107900

H	-6.44290100	-1.06844300	-4.09306900
H	-7.61504800	-1.70266600	-2.93642000
H	-7.38860600	0.04453200	-3.08106900
H	2.35558900	-0.47343600	-2.20316700
H	1.51997500	-1.92860500	-1.65427900
S	1.50597800	4.35661500	-0.31559200
C	3.19381600	4.60080600	-0.95151400
H	3.32082900	4.08788100	-1.91049100
H	3.38382500	5.67348100	-1.06375800
H	3.89046200	4.18413400	-0.21829200
C	0.56937800	4.98733800	-1.74326000
H	0.75868200	6.06055700	-1.85229800
H	0.85318900	4.45151500	-2.65537500
H	-0.49201200	4.81640500	-1.53466300
C	-0.09967000	0.20331100	3.81034200
H	-1.18548100	0.08205700	3.71882500
H	0.19337100	0.17708400	4.86618400
H	0.20602700	1.15668500	3.36780100

Structure and coordinates of **Int’-B**

C	-0.85690500	2.11107700	-0.77256500
C	-0.47397700	-0.18179200	-1.67260700
C	-0.32192800	1.29066900	-1.95804900
C	-2.54426600	1.23075700	1.67174900
C	-1.12307800	1.18531400	1.59245700
C	-0.37014000	1.53186900	0.50579500
H	-1.95768000	2.09252300	-0.79488700
H	0.71631900	1.41270500	0.58321400
C	-1.83409500	0.40375400	3.63011100
C	-1.81136000	-0.12872900	4.91037700
C	-0.55697900	-0.42556600	5.43256200
C	0.60900100	-0.19462600	4.69376200
C	0.56550100	0.34206300	3.41176500
C	-0.67986600	0.64536600	2.87340700
H	-2.71806400	-0.31131200	5.48359000
H	-0.48253900	-0.84583300	6.43291100
H	1.57438500	-0.44175700	5.13107600
H	1.48416000	0.51263500	2.84995700
C	0.55754100	-0.90567800	-1.20495600
N	1.85832200	-0.33297600	-0.99463000
S	2.91737700	-0.30167500	-2.36111700
O	2.05271100	-0.18171200	-3.52086200
O	3.92718600	0.69876300	-2.05561600
N	-2.94891200	0.78314400	2.84898300

H	-3.26116900	1.55759500	0.92428500
Au	-2.29798900	-1.05365600	-1.96032400
C	-0.39172900	3.55868200	-0.72848100
C	-1.29513300	4.56292000	-0.37361800
C	0.94540400	3.89654300	-0.96195200
C	-0.88462900	5.88774800	-0.29384400
H	-2.33656100	4.30290100	-0.17326000
C	1.35629100	5.22176500	-0.87796500
H	1.67188600	3.12613400	-1.22599100
C	0.44375400	6.21902000	-0.54694000
H	-1.60252900	6.66405000	-0.03634600
H	2.39557000	5.47558400	-1.07661800
H	0.76774400	7.25594900	-0.48596800
C	3.66739900	-1.90082100	-2.34882600
C	4.86061300	-2.09583500	-1.65884400
C	2.99531800	-2.96128600	-2.95449300
C	5.38867900	-3.37823000	-1.58622700
H	5.36539800	-1.24985100	-1.19557800
C	3.54060200	-4.23389900	-2.86846800
H	2.07203300	-2.77633700	-3.50120500
C	4.74259000	-4.46073800	-2.18849500
H	6.32656100	-3.54449800	-1.05687200
H	3.03458800	-5.07169000	-3.34864100
C	2.51166900	-0.56725400	0.25802100
C	3.27704500	0.46082500	0.81870200
C	2.42192800	-1.79574600	0.91913200
C	3.95829500	0.25337600	2.01421400
H	3.36107300	1.40579300	0.28574300
C	3.08536500	-1.98761400	2.12620400
H	1.85517200	-2.61350700	0.47765000
C	3.86093300	-0.96913100	2.67424900
H	4.56670000	1.05409200	2.43074400
H	3.01239600	-2.94991700	2.62987300
H	4.39424000	-1.13072400	3.60947600
C	5.33614800	-5.83497800	-2.13672800
H	4.56450200	-6.60410200	-2.00797100
H	5.86704500	-6.06676700	-3.07048700
H	6.05874400	-5.93438600	-1.31860800
H	-0.87275200	1.58687100	-2.86001200
H	0.72820500	1.53946400	-2.14177900
S	-4.48284400	-2.12750400	-2.43833600
C	-4.83839600	-1.47451900	-4.10028600
H	-3.95835000	-1.56664400	-4.74527000
H	-5.68678900	-2.01954600	-4.52735400

H	-5.10128600	-0.41815600	-3.99342100
C	-3.96019100	-3.80833600	-2.90210800
H	-4.80792300	-4.33777800	-3.34963100
H	-3.12028200	-3.76883400	-3.60363100
H	-3.64983100	-4.32460600	-1.98905700
C	-4.32416400	0.67061100	3.30074300
H	-4.54861000	-0.37541200	3.53626500
H	-4.46893500	1.28279200	4.19746900
H	-4.99490600	1.01760800	2.51118400
H	0.45213900	-1.97042200	-0.98113800

Structure and coordinates of **TS-2**^{Me}

C	2.57280100	-0.96751000	-1.59541300
C	0.43920100	-0.16115600	-1.38282500
C	1.34895800	-0.70540500	-2.47668100
C	3.02584000	0.09558600	1.35572600
C	2.15167300	-0.92367700	0.98458900
C	1.82071300	-1.32972800	-0.32721300
H	3.08881000	-0.00790400	-1.43632900
H	1.34048700	-2.31200200	-0.38988400
C	2.20047000	-0.72342900	3.26683700
C	1.89233900	-0.94303200	4.60643000
C	0.95618300	-1.93010500	4.87902100
C	0.35785500	-2.67336000	3.84948200
C	0.68370900	-2.45520700	2.52006000
C	1.61488900	-1.45842500	2.21867800
H	2.35833400	-0.37128000	5.40715100
H	0.68038500	-2.13193900	5.91185800
H	-0.38390700	-3.42939200	4.09690200
H	0.20200400	-3.03569900	1.73227800
C	-0.58077100	-0.97800900	-0.94233000
H	-0.69138000	-1.98029300	-1.36996500
N	-1.45651600	-0.75510900	0.08238300
S	-2.41895300	-2.09589800	0.65893000
O	-1.76433600	-3.27305300	0.11040200
O	-2.55996300	-1.87518500	2.08265700
N	3.06612100	0.21192200	2.70038500
H	3.63379100	0.74314600	0.73066800
Au	0.63214800	1.83036800	-0.86803700
C	3.58087700	-2.01831000	-1.97674700
C	4.94315300	-1.77286200	-1.79695300
C	3.18058300	-3.27058600	-2.44949100
C	5.88803400	-2.74828300	-2.09518500
H	5.26626000	-0.80045500	-1.42037600

C	4.12217400	-4.24809200	-2.74919300
H	2.12112600	-3.49137100	-2.58841800
C	5.47828100	-3.98873200	-2.57337400
H	6.94687400	-2.53941800	-1.95400200
H	3.79504700	-5.21713400	-3.12130000
H	6.21537600	-4.75432700	-2.80714500
C	-3.97302500	-1.85399500	-0.13257900
C	-4.98221100	-1.17683900	0.54630400
C	-4.14699000	-2.31433200	-1.43741900
C	-6.19164400	-0.96424000	-0.10209900
H	-4.81879500	-0.83478300	1.56626900
C	-5.36223100	-2.08927500	-2.06402700
H	-3.34985600	-2.85894200	-1.94060900
C	-6.40090700	-1.41677100	-1.40771000
H	-6.99542800	-0.44341000	0.41706300
H	-5.51946400	-2.45128200	-3.07973300
C	-1.73111900	0.52315600	0.67019600
C	-2.63405400	1.37413900	0.03269000
C	-1.14153400	0.87939000	1.88204100
C	-2.93926000	2.60240400	0.61087900
H	-3.09216900	1.05999900	-0.90534600
C	-1.44018000	2.11643500	2.44299300
H	-0.46739700	0.17755000	2.37243700
C	-2.33866000	2.97517000	1.81211700
H	-3.66058800	3.26194400	0.13033500
H	-0.98542400	2.40270400	3.38895800
H	-2.58489900	3.93255800	2.26868100
C	-7.71878600	-1.21491700	-2.08826100
H	-7.59083500	-0.97059000	-3.14981500
H	-8.32473600	-2.13025100	-2.04113300
H	-8.29972800	-0.41364200	-1.61806000
H	1.52975100	-0.01599300	-3.30865300
H	0.96210400	-1.64757200	-2.89851000
S	0.85619000	4.20864800	-0.30904100
C	2.11247000	4.80780700	-1.48288800
H	1.87460400	4.48293100	-2.50095400
H	2.15802500	5.90071200	-1.43072800
H	3.07782700	4.39065100	-1.18230200
C	-0.62160500	4.92972500	-1.08909200
H	-0.56281900	6.02148500	-1.02675200
H	-0.70070500	4.60694300	-2.13251100
H	-1.49002800	4.57154700	-0.52688200
C	3.89289300	1.12328100	3.46070900
H	3.26762800	1.76805100	4.09000100

H	4.58612300	0.56522400	4.10137800
H	4.46940800	1.74805300	2.77324500

Structure and coordinates of **TS'-2^{Me}**

C	-1.38934700	1.07340700	-1.89589300
C	-0.45907100	-0.67977000	-0.77697900
C	-0.47771900	-0.12605100	-2.18783200
C	-3.29073000	1.59298700	0.56283300
C	-1.89813800	1.65510500	0.59640100
C	-1.00764800	1.33014200	-0.44898900
H	-2.43691100	0.73655200	-1.93013900
H	0.00075500	1.74399400	-0.34859300
C	-2.76316700	2.29017000	2.62057000
C	-2.82134500	2.72171500	3.94180600
C	-1.61114700	2.95992600	4.57729700
C	-0.39111900	2.77493700	3.90930500
C	-0.34617800	2.34861800	2.59031300
C	-1.55173900	2.10074300	1.92964800
H	-3.76836000	2.86838100	4.45834400
H	-1.60897500	3.29827200	5.61117500
H	0.54030100	2.97502700	4.43601600
H	0.61154000	2.22350200	2.08420000
C	0.58961200	-0.50556200	0.09946900
N	1.85568000	-0.02681400	-0.16992100
S	2.77786000	-0.53507300	-1.56269800
O	2.09378300	-1.71116300	-2.06518000
O	2.98010000	0.64390600	-2.39027200
N	-3.80587400	1.97558700	1.74878600
H	-3.94919300	1.30797400	-0.25242000
Au	-2.02199400	-1.89501100	-0.20864400
C	-1.23431000	2.32855900	-2.71765600
C	-2.36610500	3.06630500	-3.06884700
C	0.02702400	2.80954100	-3.08205200
C	-2.24908800	4.25392700	-3.78144400
H	-3.35394000	2.69806400	-2.78560400
C	0.14453900	3.99657100	-3.79630400
H	0.92938200	2.25073300	-2.82425100
C	-0.99087300	4.72109900	-4.14753400
H	-3.14158800	4.81377400	-4.05485500
H	1.13062900	4.35468800	-4.08622200
H	-0.89492400	5.64874200	-4.70843700
C	4.30151500	-0.98386700	-0.80549300
C	5.34758900	-0.06524300	-0.76465400
C	4.39994400	-2.23533600	-0.20154500

C	6.51013200	-0.41343300	-0.09334000
H	5.24469700	0.90111800	-1.25378400
C	5.57291400	-2.56261800	0.46127300
H	3.56982500	-2.93784200	-0.26163300
C	6.64071500	-1.65979200	0.52751300
H	7.33909600	0.29238600	-0.05210800
H	5.67189900	-3.53986400	0.93303100
C	2.54029200	0.76251900	0.81425200
C	2.81756200	2.09945100	0.53181900
C	2.91952600	0.19654300	2.03075300
C	3.48436000	2.87300500	1.47770100
H	2.52036100	2.51570900	-0.43057100
C	3.56895400	0.98120200	2.97564100
H	2.70814100	-0.85476700	2.22541400
C	3.85551900	2.31637600	2.69808700
H	3.70768700	3.91564300	1.26113400
H	3.86508500	0.54565200	3.92772800
H	4.37379100	2.92440700	3.43684000
C	7.91369500	-2.03335000	1.22063900
H	7.74899800	-2.79569000	1.99079500
H	8.63814500	-2.44593800	0.50469100
H	8.38758400	-1.16418700	1.69209300
H	-0.87983100	-0.81769300	-2.93550100
H	0.49875800	0.20903700	-2.55385500
S	-3.86345800	-3.40346300	0.39017400
C	-4.18059800	-4.26205600	-1.18390200
H	-3.24699300	-4.64630700	-1.60744600
H	-4.88809200	-5.07911200	-1.00863100
H	-4.62419600	-3.53914100	-1.87459700
C	-2.99910500	-4.73729200	1.27781700
H	-3.69904700	-5.56072600	1.45381500
H	-2.13471400	-5.08432100	0.70233900
H	-2.66309000	-4.33391400	2.23745100
C	-5.21021500	2.09562200	2.07638000
H	-5.45526200	1.46161600	2.93659400
H	-5.45705100	3.13637300	2.31816900
H	-5.80916100	1.77889400	1.21833000
H	0.40203500	-0.64974500	1.16932600

Structure and coordinates of **TS**-2^{Me}**

C	-3.46761200	-1.02544400	0.47594900
C	-1.04491400	-0.04313000	0.17160700
C	-2.19580300	-0.38204100	1.12306200
C	-1.50281600	-0.92989100	-1.51327300

C	-2.04192500	-2.23570900	-1.08325300
C	-3.03594200	-2.29456700	-0.18100000
H	-3.83148800	-0.31129700	-0.28475800
H	-3.42988600	-3.24819600	0.17251500
C	-0.26102200	-2.56227600	-2.50565200
C	0.70449400	-3.23540200	-3.23938300
C	0.73160800	-4.62413700	-3.13647900
C	-0.17357200	-5.31395200	-2.32681600
C	-1.14081200	-4.62734900	-1.60128300
C	-1.19152500	-3.24217200	-1.70354500
H	1.42832300	-2.70883900	-3.85733600
H	1.47981500	-5.18060200	-3.69712800
H	-0.12189200	-6.39862900	-2.26680400
H	-1.85227300	-5.16487800	-0.97591700
C	0.10594600	-0.82086300	0.38530500
H	-0.00870500	-1.83145400	0.79715300
N	1.34501500	-0.56948200	-0.06604800
S	2.49549600	-1.93121400	-0.20442100
O	1.73319900	-3.09282200	0.20562500
O	3.06300300	-1.78422500	-1.52964600
N	-0.48700300	-1.17487400	-2.41563000
H	-2.19660400	-0.13867400	-1.80016000
Au	-0.87856300	1.96521500	-0.37646700
C	-4.54423000	-1.18794600	1.51678900
C	-5.65401800	-0.34356100	1.51916900
C	-4.43089300	-2.15117100	2.52215900
C	-6.63567600	-0.46073900	2.49784700
H	-5.75567500	0.40981100	0.73615000
C	-5.41059400	-2.27261100	3.50115200
H	-3.56558700	-2.81685200	2.54035800
C	-6.51620600	-1.42715600	3.49088900
H	-7.50009100	0.20059300	2.48076000
H	-5.31040800	-3.03137700	4.27528100
H	-7.28467900	-1.52464100	4.25530500
C	3.68822300	-1.50497400	1.01192300
C	4.81535300	-0.77646400	0.63915700
C	3.43096000	-1.83680800	2.34139600
C	5.70188400	-0.37789700	1.62889600
H	4.98874700	-0.53408100	-0.40746400
C	4.33246100	-1.42830200	3.31166100
H	2.54874300	-2.41939800	2.60297500
C	5.47805000	-0.69780200	2.97198100
H	6.59173200	0.18857800	1.35663900
H	4.15432400	-1.68627400	4.35506600

C	1.93046600	0.73587700	-0.19330200
C	2.11034500	1.48881700	0.97064000
C	2.40330300	1.19459300	-1.41914100
C	2.73110800	2.72841000	0.89266600
H	1.76704900	1.08743700	1.92393700
C	3.02095500	2.44022000	-1.48744400
H	2.30012000	0.56828600	-2.30067900
C	3.17903100	3.20873200	-0.33779500
H	2.88639900	3.31142200	1.79909900
H	3.38830100	2.80655500	-2.44364800
H	3.67368500	4.17656800	-0.39650800
C	6.46262600	-0.29754100	4.02524600
H	5.97755900	-0.13650400	4.99502600
H	7.21552600	-1.08520500	4.16814600
H	7.00123800	0.61703300	3.75155600
H	-2.51730700	0.52434200	1.65256800
H	-1.85824000	-1.08144700	1.90508200
S	-0.65713400	4.33554100	-0.96395400
C	-2.37349900	4.89901200	-1.18732500
H	-2.99689500	4.57833200	-0.34644800
H	-2.38168800	5.99040900	-1.27558300
H	-2.75121800	4.45815100	-2.11432900
C	-0.31768500	5.08688200	0.65919400
H	-0.31143700	6.17714400	0.55733200
H	-1.06812600	4.77404200	1.39263700
H	0.67121700	4.74008900	0.97605100
C	-0.07885900	-0.23358700	-3.43136800
H	0.95904600	-0.43141500	-3.72444000
H	-0.71149600	-0.29968500	-4.32877000
H	-0.13608000	0.78648100	-3.02656800

Structure and coordinates of **Int-C**

C	2.02385700	-1.85677600	-1.82142300
C	-0.11702600	-2.00171900	-1.77516100
C	0.98039000	-2.57685500	-2.69835900
C	0.65395600	0.81491800	-0.11464100
C	1.01150100	-0.48176200	0.15334200
C	0.94842400	-1.69038000	-0.70449100
H	2.21800400	-0.85688600	-2.24332200
H	1.00442600	-2.58249800	-0.05443200
C	1.46807000	0.84917800	1.96617000
C	1.87436800	1.20370200	3.25446000
C	2.40408600	0.19987700	4.04804100
C	2.53524300	-1.11850600	3.57237200

C	2.12699500	-1.46303700	2.29651600
C	1.56594800	-0.47285500	1.47836400
H	1.78754700	2.22547300	3.62176700
H	2.73614700	0.43718400	5.05703300
H	2.97556600	-1.87381100	4.22053400
H	2.25007200	-2.48370300	1.93072400
C	-1.36672300	-2.65085000	-1.63491000
H	-1.74696000	-3.19588300	-2.50518500
N	-2.16557800	-2.72683100	-0.57055800
S	-3.80661800	-3.39491700	-0.81289700
O	-3.77237600	-3.94313800	-2.15507200
O	-4.06073300	-4.18512500	0.36851600
N	0.91615300	1.62517500	0.97002800
H	0.21655100	1.25373600	-1.01000600
Au	-1.11834600	-0.33389400	-2.72808100
C	3.30741100	-2.49390000	-1.39716800
C	4.39328100	-1.67737600	-1.07299500
C	3.42434900	-3.87186600	-1.20001800
C	5.56963300	-2.22098400	-0.57032600
H	4.30628800	-0.59829400	-1.21020900
C	4.60200900	-4.41972500	-0.70249000
H	2.58966800	-4.53314600	-1.43743800
C	5.67770600	-3.59583800	-0.38595400
H	6.40561900	-1.56934500	-0.32291100
H	4.68023900	-5.49661200	-0.56354400
H	6.59941900	-4.02479800	0.00249800
C	-4.75439800	-1.91567500	-0.77704700
C	-5.32381600	-1.50825800	0.42664300
C	-4.82819400	-1.13368000	-1.93014200
C	-5.98290500	-0.28771000	0.46818800
H	-5.25053800	-2.14347000	1.30730200
C	-5.48437200	0.08654700	-1.86099900
H	-4.40489000	-1.49312000	-2.86783500
C	-6.07061100	0.52533900	-0.66661300
H	-6.44152000	0.04354100	1.39915500
H	-5.56598700	0.70619400	-2.75396900
C	-1.89409900	-2.15383500	0.72109100
C	-2.14811000	-0.80324100	0.94886000
C	-1.43403100	-2.98686800	1.73800000
C	-1.91181100	-0.27628000	2.21313500
H	-2.52471000	-0.17392600	0.14075300
C	-1.19327600	-2.44616100	2.99479500
H	-1.27427400	-4.04404900	1.53434000
C	-1.42662700	-1.09364000	3.23007500

H	-2.09568400	0.78022200	2.40148000
H	-0.81576100	-3.08178100	3.79253600
H	-1.22084600	-0.67143500	4.21175700
C	-6.81631000	1.82210400	-0.61548100
H	-6.45617000	2.52967300	-1.37136400
H	-7.88582700	1.65925000	-0.80766400
H	-6.73613100	2.29694800	0.36938500
H	0.96805000	-2.32760800	-3.76503200
H	1.03105300	-3.67033700	-2.60178100
S	-2.10091300	1.56898300	-3.90251500
C	-1.10040700	2.97329600	-3.31534900
H	-0.03143100	2.74554400	-3.38698700
H	-1.34284400	3.85525200	-3.91735400
H	-1.37407100	3.16164100	-2.27248100
C	-1.40838200	1.39548200	-5.57911300
H	-1.67704000	2.28154000	-6.16353000
H	-0.32099000	1.27791900	-5.53795500
H	-1.85905800	0.50916600	-6.03500500
C	0.65121300	3.03683000	1.07255100
H	-0.09912400	3.24686700	1.84772400
H	1.56714600	3.59047200	1.31649500
H	0.27406900	3.40462000	0.11229700

Structure and coordinates of **TS*-1^{Me}**

C	-11.47069800	-12.31438600	1.40170700
C	-8.54272500	-12.98769600	0.69874700
C	-8.36228600	-12.10846300	-0.37795800
C	-10.18864200	-11.11113900	-1.04728600
C	-10.50140600	-10.44422800	0.16115400
C	-11.06960500	-11.01697500	1.33038200
H	-11.44655200	-12.90348300	0.48132000
H	-8.22694100	-12.51574100	-1.38046200
H	-11.12887100	-10.37972400	2.21464200
C	-9.48948600	-8.99393200	-1.29917700
C	-8.89697900	-7.81668000	-1.76190000
C	-8.82584400	-6.75655900	-0.87509900
C	-9.31534000	-6.85685600	0.44236600
C	-9.89100100	-8.02581400	0.89973300
C	-9.98755900	-9.11529000	0.02048700
H	-8.50812700	-7.73067300	-2.77495500
H	-8.37779800	-5.82064900	-1.20333300
H	-9.23471800	-5.99835400	1.10530800
H	-10.25956600	-8.10151200	1.92214600
C	-8.46246800	-12.36816000	1.94222500

H	-8.40468000	-11.27517200	1.98411900
N	-8.43673200	-12.94053100	3.16855200
S	-8.41224200	-11.88161700	4.57773800
O	-8.85242500	-10.59291900	4.06931100
O	-9.13475400	-12.59802500	5.60686900
N	-9.68869000	-10.18834200	-1.95216200
H	-10.64125500	-12.02327800	-1.42931500
H	-7.89582000	-11.12954500	-0.22301300
Au	-9.08294600	-14.95962600	0.47428900
C	-11.96000300	-13.03896700	2.55639700
C	-12.48723100	-14.32742700	2.35746800
C	-11.89265000	-12.53815300	3.86674900
C	-12.95123400	-15.08270700	3.42389400
H	-12.53298800	-14.72737900	1.34307500
C	-12.35006900	-13.29840500	4.93427500
H	-11.45456500	-11.55852500	4.05978000
C	-12.88573600	-14.56739500	4.71792600
H	-13.36649500	-16.07361700	3.24960600
H	-12.26868500	-12.90218500	5.94431900
H	-13.24535700	-15.15662900	5.55932300
C	-6.70340400	-11.82159700	4.98934600
C	-6.18915000	-12.76183600	5.87930300
C	-5.89429600	-10.85664200	4.39039500
C	-4.83343600	-12.72345900	6.17326000
H	-6.84664000	-13.49868500	6.33681500
C	-4.54381400	-10.83778400	4.70218100
H	-6.32463400	-10.12100700	3.71295900
C	-3.99502600	-11.76417400	5.59729700
H	-4.41561700	-13.44612400	6.87327500
H	-3.89770100	-10.08381100	4.25355600
C	-8.19678000	-14.33412800	3.40735300
C	-6.93511300	-14.85866400	3.12366100
C	-9.19989300	-15.12690000	3.95811300
C	-6.68371100	-16.20123500	3.37926000
H	-6.16725400	-14.20797000	2.70556300
C	-8.93865700	-16.46973600	4.21019800
H	-10.16569200	-14.68202000	4.18523800
C	-7.68470400	-17.00555000	3.92488100
H	-5.69969700	-16.61685100	3.17051400
H	-9.71795600	-17.09319800	4.64477700
H	-7.47959200	-18.05284100	4.14292700
C	-2.54257500	-11.70415800	5.95368200
H	-2.17836800	-12.66335700	6.33863900
H	-1.92614800	-11.42239200	5.09161200

H	-2.36678000	-10.95122400	6.73448400
S	-9.81074600	-17.29242200	0.28737600
C	-8.23653900	-18.20451300	0.32098600
H	-7.81335700	-18.08098400	1.32316800
H	-8.42967000	-19.26516000	0.12809500
H	-7.54130200	-17.79806800	-0.42108400
C	-10.22066200	-17.49202600	-1.47415300
H	-10.41069900	-18.55055100	-1.68099500
H	-11.12926200	-16.91608200	-1.67259100
H	-9.40573100	-17.12018300	-2.10362000
C	-9.22558400	-10.48911000	-3.28486200
H	-9.58986400	-9.73449300	-3.99177200
H	-8.12720800	-10.51869200	-3.33476100
H	-9.62009500	-11.46287700	-3.59218200

Structure and coordinates of **Int*-B**

C	-2.38725100	1.24009400	-0.01707100
C	0.35019100	0.47019500	-0.92352800
C	0.43833500	1.53494400	-1.96278800
C	-0.94922700	1.96747100	-2.58748400
C	-1.67160300	2.89144200	-1.65965200
C	-2.21999200	2.54307000	-0.42200800
H	-2.21654500	0.45116700	-0.75159800
H	1.04248900	1.18753400	-2.81248300
H	-2.51777400	3.35684600	0.24063900
C	-0.99966500	4.07591600	-3.50336000
C	-0.79262300	5.21050900	-4.30304700
C	-1.20936600	6.42561100	-3.79810100
C	-1.81680600	6.55289300	-2.52615700
C	-2.01058200	5.44600900	-1.73481400
C	-1.59460700	4.18776400	-2.21479100
H	-0.32266700	5.13898400	-5.28126100
H	-1.06342700	7.32152300	-4.39891000
H	-2.12614600	7.53562800	-2.17968900
H	-2.47287500	5.53726300	-0.75328200
C	0.51534200	0.86041300	0.36068400
H	0.63297100	1.92093400	0.59985600
N	0.48811700	0.05357500	1.50202300
S	0.50813800	0.83380900	3.03708800
O	0.12036200	2.21067600	2.76825100
O	-0.24977500	-0.01810500	3.93339500
N	-0.71885400	2.77948900	-3.76966700
H	-1.50293800	1.05193800	-2.84828600
H	0.91178800	2.45799800	-1.58701800

Au	-0.05331900	-1.47065200	-1.46607100
C	-2.83386500	0.78395300	1.26860200
C	-3.13341100	-0.58231900	1.42857700
C	-2.97987900	1.64422600	2.37377600
C	-3.58494300	-1.06924200	2.64466200
H	-3.01677300	-1.25360100	0.57571900
C	-3.42283700	1.15333600	3.58817900
H	-2.71798300	2.69624100	2.28320700
C	-3.72601500	-0.20152100	3.72601800
H	-3.82551500	-2.12482700	2.75546400
H	-3.51818000	1.82199600	4.44023500
H	-4.07041200	-0.58318800	4.68506200
C	2.21056400	0.77342000	3.50122400
C	2.66370000	-0.28420900	4.28419700
C	3.08713800	1.72839600	2.98900600
C	4.02127200	-0.37565500	4.56195500
H	1.95585600	-1.01617600	4.66868200
C	4.43881400	1.61579900	3.27583700
H	2.70807900	2.55493700	2.39041900
C	4.92534100	0.56626500	4.06491800
H	4.38945000	-1.19513300	5.17849300
H	5.13667500	2.35821300	2.88882600
C	0.90562700	-1.31515900	1.48861800
C	2.19921100	-1.63426400	1.06607000
C	0.04513100	-2.31320400	1.94228200
C	2.62498000	-2.95598300	1.08665600
H	2.86020300	-0.83622600	0.72794200
C	0.48058500	-3.63591500	1.96427100
H	-0.95154500	-2.03586000	2.28076400
C	1.76659200	-3.95846200	1.53981800
H	3.63657900	-3.20310500	0.76914000
H	-0.18610000	-4.41557200	2.32796300
H	2.10786700	-4.99222600	1.57576500
C	6.38423700	0.47448500	4.39006700
H	6.67435000	-0.54704300	4.66170700
H	7.00722000	0.79503800	3.54619800
H	6.63690400	1.12212000	5.24099500
S	-0.53065700	-3.82697200	-2.01670200
C	1.14262900	-4.53675500	-2.11202600
H	1.55328800	-4.53064600	-1.09649000
H	1.07719300	-5.56577200	-2.48143200
H	1.77913400	-3.93201400	-2.76678300
C	-0.92847000	-3.79737900	-3.79217700
H	-0.98127000	-4.82539600	-4.16599300

H	-1.90582600	-3.31980800	-3.90788400
H	-0.17363200	-3.22933700	-4.34582100
C	-0.02772400	2.29552000	-4.93771800
H	-0.46522500	2.73490400	-5.84208500
H	1.04778300	2.52839800	-4.91650600
H	-0.14987400	1.20838800	-5.00043600

Structure and coordinates of **TS*-2^{Me}**

C	-0.93010100	-0.76792200	-2.03414700
C	-0.48443000	-0.09729000	-0.17088800
C	-1.74563600	-0.39450200	0.63034500
C	-3.03707800	-0.52562700	-0.22632200
C	-2.88245900	-1.76561300	-1.05780900
C	-1.79804000	-1.90224900	-1.86549000
H	-1.46706000	0.17412000	-2.17292600
H	-1.89254000	0.40142800	1.37374200
H	-1.54285100	-2.86121800	-2.31698000
C	-4.65180400	-2.06415000	0.37896800
C	-5.69075300	-2.74182400	1.02020900
C	-5.95009500	-4.04666100	0.62316200
C	-5.20116400	-4.68510500	-0.37711300
C	-4.16201900	-4.01457900	-1.00114600
C	-3.88250200	-2.69994700	-0.62319200
H	-6.27676400	-2.26941600	1.80619900
H	-6.75916900	-4.59096100	1.10745100
H	-5.43862500	-5.70852300	-0.65713700
H	-3.57277800	-4.49911600	-1.77892900
C	0.52907800	-1.04215600	0.05887200
H	0.23157700	-2.07354300	0.28909800
N	1.85424700	-0.91475000	-0.08078400
S	2.86358100	-2.38797100	0.02582100
O	1.90461600	-3.47616900	0.06556200
O	3.84992900	-2.24684000	-1.02207900
N	-4.21633800	-0.77863000	0.56991300
H	-3.16199700	0.40007200	-0.81753200
H	-1.66361400	-1.33902000	1.19496400
Au	-0.02376700	1.90880300	-0.50866800
C	0.25361900	-0.84305200	-2.90658100
C	0.58997000	0.26198300	-3.69891200
C	1.04450900	-1.99594500	-2.98946900
C	1.67634700	0.21329800	-4.56331000
H	-0.02403400	1.16302100	-3.64519800
C	2.13899700	-2.04236000	-3.84481400
H	0.81237300	-2.86307600	-2.36840700

C	2.45537700	-0.94000300	-4.63492600
H	1.91485400	1.07396700	-5.18553700
H	2.75469300	-2.93850300	-3.88162600
H	3.31010300	-0.97995800	-5.30741400
C	3.62012900	-2.16758500	1.59612700
C	4.86982600	-1.55601200	1.66515600
C	2.92058500	-2.54790200	2.74084800
C	5.42556000	-1.32700300	2.91569000
H	5.39161700	-1.26934900	0.75395400
C	3.49674300	-2.30701300	3.97848300
H	1.95225900	-3.03919900	2.65777800
C	4.75341000	-1.69769800	4.08466500
H	6.40455900	-0.85505000	2.99060100
H	2.97045900	-2.60509200	4.88462700
C	2.58742700	0.32046600	-0.09117100
C	2.74925200	0.99975400	1.11792200
C	3.18851600	0.77455600	-1.26204300
C	3.50736900	2.16406300	1.14772200
H	2.29112900	0.60024000	2.02308900
C	3.94034900	1.94513000	-1.22037800
H	3.06146400	0.21717700	-2.18857100
C	4.10028300	2.63740800	-0.02235800
H	3.65161300	2.69038900	2.08998800
H	4.40885700	2.31312500	-2.13057400
H	4.70367100	3.54328700	0.00259800
C	5.38050500	-1.47859600	5.42573300
H	4.62625000	-1.33124500	6.20740000
H	5.98204700	-2.35089100	5.71693300
H	6.05030600	-0.61093900	5.42340000
S	0.53873900	4.25400000	-0.95271400
C	0.84059300	4.91993700	0.71389800
H	1.76303600	4.46088900	1.08395400
H	0.96985800	6.00542200	0.64852000
H	0.01137800	4.67197000	1.38462900
C	-1.07203000	5.03416300	-1.28480400
H	-0.93830300	6.11893900	-1.35215700
H	-1.43492500	4.65376600	-2.24407900
H	-1.79019500	4.78515600	-0.49692400
C	-4.72820400	0.14021400	1.54757700
H	-4.32153700	-0.03289200	2.55740500
H	-4.48034000	1.16596400	1.24640400
H	-5.82163300	0.06639500	1.59933600

Structure and coordinates of **Int*-C**

C	-1.91003200	-0.26367000	-0.60218300
C	-1.41356900	-0.32242900	0.83229900
C	-2.52601100	-0.59539300	1.82274800
C	-3.54190700	-1.61905300	1.26101900
C	-3.02489300	-2.34688000	0.04375000
C	-2.27863900	-1.70935000	-0.86680200
H	-2.84506900	0.33064800	-0.54879100
H	-3.05055100	0.34585200	2.04764000
H	-1.85728800	-2.21656800	-1.73135700
C	-3.99831400	-3.86926500	1.46700300
C	-4.52683500	-5.08605000	1.88308700
C	-4.47340600	-6.15810500	0.99413300
C	-3.91290600	-6.02985200	-0.27832400
C	-3.40033700	-4.80590300	-0.69396300
C	-3.45936200	-3.72335800	0.17639100
H	-4.96566400	-5.20930000	2.87134000
H	-4.88140400	-7.11869600	1.30376700
H	-3.88784800	-6.88682600	-0.94732100
H	-2.97627300	-4.68861200	-1.69056900
C	-0.10235800	-0.31142200	1.30512700
H	0.02750300	-0.59637300	2.36059300
N	1.06245400	-0.34531200	0.48687400
S	1.62530100	-1.92458500	0.04716400
O	0.41656900	-2.68318800	-0.22538400
O	2.64494600	-1.69239000	-0.95687800
N	-3.88358800	-2.68131600	2.19896600
H	-4.46686300	-1.07339600	0.97533800
H	-2.11578500	-0.97712000	2.76706100
Au	-0.53675300	1.90036200	1.44174900
C	-1.03444100	0.42167700	-1.62458000
C	-1.17039200	1.80500100	-1.78537100
C	-0.11809700	-0.25477200	-2.42812400
C	-0.38659700	2.50572600	-2.69860300
H	-1.94533800	2.33024300	-1.21836500
C	0.66401800	0.44008100	-3.34595900
H	0.01104800	-1.33096500	-2.32261600
C	0.53873100	1.82060400	-3.48090500
H	-0.52780700	3.57866000	-2.83088700
H	1.37986100	-0.10689200	-3.95674500
H	1.14413100	2.35659000	-4.20933400
C	2.37991600	-2.54655300	1.51443500
C	3.73535100	-2.30822700	1.73573300
C	1.59092400	-3.20249500	2.45857800
C	4.30164900	-2.73599200	2.92780100

H	4.32970500	-1.80628700	0.97444700
C	2.17918300	-3.61895800	3.64504500
H	0.54201700	-3.40771900	2.24844500
C	3.53681700	-3.39485300	3.89671000
H	5.36204200	-2.56460900	3.11155200
H	1.57973600	-4.14466000	4.38771000
C	2.10742900	0.60162100	0.77588300
C	2.57128600	0.80578400	2.08045400
C	2.66539500	1.30940800	-0.28863000
C	3.58426400	1.73061400	2.31444700
H	2.15469500	0.23092300	2.90819500
C	3.69438600	2.21401000	-0.04833200
H	2.28635500	1.12616200	-1.29290900
C	4.15357900	2.42755100	1.25006200
H	3.94964800	1.88521200	3.32780300
H	4.14391400	2.75162600	-0.88119500
H	4.96243500	3.13257700	1.43296100
C	4.16972700	-3.88489400	5.16188100
H	3.43197300	-4.00756300	5.96308900
H	4.64444700	-4.86333700	5.00450900
H	4.95178200	-3.20209800	5.51496000
S	-0.42378800	4.29386100	1.70122000
C	-1.69135100	4.93203500	0.56028000
H	-2.67395400	4.68259900	0.97085500
H	-1.58681500	6.02064700	0.50423200
H	-1.57294500	4.48647400	-0.43305900
C	1.06551900	4.63986900	0.70768900
H	1.19931600	5.72503300	0.64791900
H	1.91974100	4.18589200	1.22210400
H	0.96742400	4.20025300	-0.29141200
C	-4.86278300	-2.36851300	3.21484700
H	-4.91111900	-3.17480000	3.95443200
H	-4.56350600	-1.45743200	3.74869100
H	-5.87380100	-2.21454500	2.79648900

Structure and coordinates of **TS*-1$^{\mathbf{CO2Et}}$**

C	-11.43121200	-12.31611000	1.40667600
C	-8.46479200	-12.99749700	0.73517300
C	-8.25693300	-12.11442300	-0.32813800
C	-10.11931000	-11.15528200	-1.02325500
C	-10.46918700	-10.46352600	0.14128600
C	-11.03024200	-11.01999700	1.32499200
H	-11.40886800	-12.90986600	0.48910200
H	-8.14347800	-12.49361000	-1.34385300

H	-11.08900900	-10.37223600	2.20158900
C	-9.50383900	-8.98358400	-1.34707200
C	-8.98500200	-7.77717600	-1.81353700
C	-8.99959000	-6.69994600	-0.93869100
C	-9.50441600	-6.80755000	0.36628000
C	-10.01215400	-8.00819500	0.82739300
C	-10.01730000	-9.10867100	-0.03709500
H	-8.59322700	-7.68025000	-2.82065100
H	-8.60842200	-5.74344200	-1.27991000
H	-9.49463800	-5.93625600	1.01731800
H	-10.40036600	-8.09800200	1.84116600
C	-8.38370900	-12.39108400	1.98604100
H	-8.29848600	-11.30045800	2.04266900
N	-8.38969900	-12.97440900	3.20479100
S	-8.37940600	-11.92145600	4.61917100
O	-8.81738100	-10.63252000	4.10858100
O	-9.11180500	-12.64514400	5.63579200
C	-9.22495800	-10.68078000	-3.22433400
O	-8.83982100	-9.67165400	-3.99075000
O	-9.24884400	-11.84922500	-3.53580200
N	-9.61862400	-10.24011700	-1.95882500
H	-10.50483600	-12.09768700	-1.39724700
H	-7.81197600	-11.12884000	-0.15243200
Au	-9.02576100	-14.95582400	0.45756300
C	-8.40990700	-10.02736200	-5.32443900
C	-8.01022900	-8.75068900	-6.01109700
H	-9.24069500	-10.53595600	-5.82839000
H	-7.58260000	-10.74258600	-5.23981600
H	-7.68432900	-8.96273400	-7.03531600
H	-8.85348900	-8.05204900	-6.05833800
H	-7.18232500	-8.26149500	-5.48401100
C	-11.93159700	-13.02922100	2.56370300
C	-12.47876800	-14.30957800	2.36714700
C	-11.86468700	-12.52155900	3.87153100
C	-12.96515500	-15.04975900	3.43423000
H	-12.52179800	-14.71533900	1.35528200
C	-12.34250400	-13.26781900	4.93988300
H	-11.41220100	-11.54810300	4.06194600
C	-12.89957400	-14.52801500	4.72560800
H	-13.39682300	-16.03401200	3.26243800
H	-12.26110500	-12.86824100	5.94856500
H	-13.27610100	-15.10589900	5.56748300
C	-6.67349700	-11.86216400	5.04097400
C	-6.16151300	-12.81486900	5.91798800

C	-5.86228100	-10.88894900	4.45650400
C	-4.80494200	-12.78305700	6.21248300
H	-6.82029700	-13.55542900	6.36751300
C	-4.51235100	-10.87786700	4.76750200
H	-6.29166300	-10.14134100	3.79180400
C	-3.96437700	-11.82045500	5.64725100
H	-4.38896600	-13.51465100	6.90404100
H	-3.86453200	-10.11823400	4.33061600
C	-8.19757200	-14.37756000	3.43180400
C	-6.94850100	-14.93552700	3.15783700
C	-9.23704300	-15.14990000	3.94308700
C	-6.74740500	-16.29271700	3.37879600
H	-6.15207800	-14.30048900	2.77037800
C	-9.02665500	-16.50815100	4.15679500
H	-10.19251300	-14.68092400	4.16655000
C	-7.78664200	-17.07829000	3.87764000
H	-5.77402800	-16.73559100	3.17603900
H	-9.83553200	-17.11763800	4.55562600
H	-7.62240600	-18.13866700	4.06472900
C	-2.50647200	-11.77676600	5.98318300
H	-2.19917000	-12.64746300	6.57259900
H	-1.88868100	-11.74292900	5.07667800
H	-2.26542000	-10.87795200	6.56658000
S	-9.76758000	-17.26950100	0.15562300
C	-8.20432500	-18.19998300	0.21350200
H	-7.81710400	-18.12077600	1.23462400
H	-8.40128300	-19.25001700	-0.02750200
H	-7.47849500	-17.77395100	-0.48713000
C	-10.10272000	-17.37894600	-1.63027300
H	-10.28966800	-18.42462500	-1.89639600
H	-10.99786600	-16.78581100	-1.83852700
H	-9.25992600	-16.98086400	-2.20448700

Structure and coordinates of **Int*-D**

C	-2.39859400	1.13870200	0.42518500
C	0.29762000	0.40353700	-0.28346500
C	0.43333800	1.47788500	-1.30341100
C	-0.91028700	1.80674500	-2.06808000
C	-1.71451100	2.75203900	-1.25083800
C	-2.28402400	2.44431500	-0.02415300
H	-2.23268400	0.33898200	-0.29707200
H	1.12352500	1.15694000	-2.09396900
H	-2.64281500	3.26467500	0.59860900
C	-0.99707300	3.88835600	-3.12903800

C	-0.82306400	4.98761800	-3.96981900
C	-1.31126500	6.21068800	-3.53185500
C	-1.95839800	6.36643500	-2.29402800
C	-2.13095800	5.27915800	-1.46315700
C	-1.64803600	4.02951200	-1.87935900
H	-0.32813400	4.88975500	-4.92950400
H	-1.18644400	7.08040400	-4.17414900
H	-2.32081300	7.34701200	-1.99576800
H	-2.63095400	5.38485300	-0.50174700
C	0.39196800	0.76088700	1.02251300
H	0.45776100	1.81510700	1.30735200
N	0.36265200	-0.09269600	2.12302000
S	0.39177500	0.61261700	3.69966600
O	0.05388100	2.01389900	3.49956000
O	-0.40524400	-0.25555400	4.54488900
C	-0.09307400	1.88195800	-4.37305900
O	0.11863400	2.69321300	-5.40343900
O	0.13905500	0.69270600	-4.34375700
N	-0.62629100	2.55777900	-3.28645400
H	-1.39390200	0.85356400	-2.32049000
H	0.81447100	2.42562600	-0.88661000
Au	0.00142300	-1.51026500	-0.96986900
C	0.66213600	2.07497900	-6.58984000
C	0.88416000	3.17364800	-7.59296600
H	-0.05160500	1.32046900	-6.94318900
H	1.58957500	1.55707300	-6.31722500
H	1.28791800	2.75813100	-8.52279900
H	-0.05639300	3.68553700	-7.82829500
H	1.59797400	3.91383700	-7.21232600
C	-2.85179600	0.70771800	1.71327700
C	-3.17685200	-0.65162500	1.88860000
C	-2.99273500	1.58843800	2.80366800
C	-3.66521500	-1.10825300	3.10146400
H	-3.05467800	-1.33812000	1.04896400
C	-3.47197700	1.12648000	4.01535400
H	-2.68858800	2.62877200	2.70548500
C	-3.81283200	-0.21859900	4.16459300
H	-3.92853000	-2.15675100	3.22551800
H	-3.56315300	1.80804000	4.85751500
H	-4.18497700	-0.57790800	5.12187900
C	2.08769300	0.46501600	4.16251400
C	2.49565500	-0.64015200	4.90297900
C	3.00292000	1.39974500	3.68074500
C	3.84898200	-0.80216500	5.16873400

H	1.75848600	-1.35616900	5.26135100
C	4.34932400	1.21660000	3.95526900
H	2.65826700	2.26145400	3.11125900
C	4.79145600	0.11699200	4.70149900
H	4.18320000	-1.65997100	5.75145000
H	5.07786400	1.94112600	3.59173000
C	0.80936100	-1.45244100	2.04506200
C	2.11564000	-1.71919200	1.62667200
C	-0.03684300	-2.49021600	2.42703500
C	2.56228700	-3.03225500	1.56125800
H	2.76808500	-0.88755500	1.36076400
C	0.41911500	-3.80429400	2.36275500
H	-1.03844700	-2.25037700	2.77776700
C	1.71268100	-4.07688100	1.92659300
H	3.58268100	-3.24109700	1.24443000
H	-0.23827500	-4.61829300	2.66191700
H	2.06747400	-5.10560900	1.88863400
C	6.24635800	-0.05090500	5.01355800
H	6.49023300	-1.09074300	5.25982200
H	6.87835500	0.26012700	4.17279900
H	6.53401300	0.56470800	5.87711000
S	-0.34071400	-3.75835700	-1.89930300
C	1.37297500	-4.26349500	-2.25012500
H	1.86420700	-4.43872400	-1.28820000
H	1.36450200	-5.18991700	-2.83410100
H	1.90097500	-3.47196900	-2.79237900
C	-0.87108400	-3.35494700	-3.59476600
H	-0.85162100	-4.26438400	-4.20474500
H	-1.89643300	-2.97798900	-3.53734600
H	-0.22497400	-2.58266400	-4.02571100

Structure and coordinates of **TS*-2**$^{\mathbf{CO2Et}}$

C	-0.82374300	-0.91104900	-2.04727100
C	-0.38056000	-0.18095000	-0.13835600
C	-1.64144500	-0.49051400	0.63764500
C	-2.90713900	-0.44486000	-0.25117100
C	-2.88879200	-1.68029000	-1.08615100
C	-1.80621600	-1.94681000	-1.86085000
H	-1.25618200	0.07974200	-2.20217000
H	-1.76888500	0.25082800	1.43642800
H	-1.65794200	-2.92773500	-2.31283100
C	-4.76590700	-1.78083000	0.26797600
C	-5.92070100	-2.33180600	0.81561900
C	-6.31048700	-3.58963300	0.36651300

C	-5.58126100	-4.29616600	-0.59586200
C	-4.42895500	-3.74608200	-1.13368300
C	-4.02040100	-2.48435600	-0.70117300
H	-6.49932600	-1.79934500	1.56224700
H	-7.21461700	-4.03313200	0.77969600
H	-5.92261000	-5.27604800	-0.92125800
H	-3.85333300	-4.27991700	-1.88857400
C	0.64772600	-1.11065700	0.05472900
H	0.37982800	-2.15085400	0.28089500
N	1.96841600	-0.93783400	-0.10363700
S	3.03345100	-2.36599400	-0.02435900
O	2.12217900	-3.49354500	0.03880000
O	3.99126400	-2.18778600	-1.09324800
C	-4.54901000	0.54739400	1.24724800
O	-5.70332500	0.32908700	1.87402000
O	-3.90616200	1.57730800	1.29407700
N	-4.13711000	-0.55089200	0.52264800
H	-2.90261500	0.49973500	-0.81746700
H	-1.61463600	-1.48695800	1.11130200
Au	-0.05311700	1.84953800	-0.44384900
C	-6.20658700	1.42695000	2.65977800
C	-7.49941000	0.96696800	3.27707300
H	-6.33951800	2.29450600	2.00174900
H	-5.45263900	1.69160500	3.41166400
H	-7.93075200	1.76623400	3.88985400
H	-8.22850100	0.69752700	2.50393000
H	-7.33684900	0.09361500	3.91980500
C	0.36411500	-1.13094200	-2.87970500
C	0.84194300	-0.08389900	-3.67787400
C	1.02687000	-2.36537200	-2.91686600
C	1.93928400	-0.27126400	-4.50947500
H	0.33494900	0.88227900	-3.64948100
C	2.13695100	-2.54600600	-3.73026400
H	0.68351500	-3.18718600	-2.28766400
C	2.59269500	-1.50154500	-4.53191200
H	2.28635800	0.54432000	-5.14138600
H	2.65599700	-3.50205900	-3.72908900
H	3.45864400	-1.64754300	-5.17432900
C	3.81470300	-2.11570800	1.53043200
C	5.04445700	-1.46329400	1.57541600
C	3.14846200	-2.51311100	2.68907100
C	5.61431500	-1.21028000	2.81522900
H	5.53872900	-1.16251700	0.65352600
C	3.73734400	-2.24745500	3.91539400

H	2.19506300	-3.03539300	2.62530100
C	4.97514600	-1.59677000	3.99718500
H	6.57815400	-0.70559800	2.87070500
H	3.23674900	-2.55783500	4.83196100
C	2.64405200	0.33024300	-0.09478000
C	2.74882400	1.01567100	1.11720200
C	3.23797300	0.82107200	-1.25354500
C	3.42999600	2.22617200	1.15831000
H	2.30298700	0.58838700	2.01565700
C	3.91110100	2.03808200	-1.20194500
H	3.15981100	0.25508900	-2.17972100
C	4.00424000	2.74093700	-0.00336000
H	3.52744000	2.75880300	2.10308200
H	4.37170200	2.43444300	-2.10429200
H	4.54450100	3.68543000	0.03006200
C	5.61618300	-1.35003700	5.32687700
H	6.27788400	-0.47657000	5.30275200
H	4.86940900	-1.19629500	6.11460900
H	6.22830900	-2.21226100	5.62585400
S	0.21795500	4.26458700	-0.72540100
C	0.50289900	4.78886000	0.99448200
H	1.48563800	4.40856900	1.29171400
H	0.50048800	5.88284800	1.04231400
H	-0.26620500	4.37365400	1.65433200
C	-1.50967100	4.81389100	-0.90967200
H	-1.54985200	5.90456600	-0.81972100
H	-1.84400900	4.52004800	-1.90889300
H	-2.14730800	4.33833300	-0.15666900

Structure and coordinates of **TS**-2**$^{\mathbf{CO2Et}}$

C	-0.31686400	3.55146200	-0.04847900
C	0.67652600	0.26273100	-0.80790800
C	1.29741100	1.16143500	-1.86798700
C	2.21105000	1.98071900	-0.97965900
C	1.50440600	1.91390900	0.35988500
C	0.29820800	2.61831700	0.70486000
H	0.16648400	3.89027000	-0.96900000
H	0.55252900	1.81581400	-2.33816000
H	-0.15030600	2.29074000	1.64621200
C	3.66573700	1.14498900	0.67625000
C	4.78464700	0.74205000	1.40373600
C	4.69736700	0.78229100	2.79079500
C	3.53831500	1.20003200	3.46037100
C	2.41596600	1.55847300	2.73664900

C	2.47494700	1.52119200	1.33919600
H	5.69743600	0.43219500	0.90783100
H	5.56962400	0.49210000	3.37389200
H	3.52534500	1.23721900	4.54725900
H	1.50263200	1.86962400	3.24140900
C	1.25280100	-0.97921600	-0.64505500
H	1.95733400	-1.36132400	-1.39567000
N	1.04159100	-1.87866200	0.36355500
S	1.13010300	-3.58028300	-0.05193900
O	1.92648300	-3.62868100	-1.26256200
O	1.49194600	-4.27051200	1.16586900
C	4.40855600	1.20400200	-1.73289900
O	5.53963900	0.63240000	-1.33707400
O	4.16315800	1.60473300	-2.84629300
N	3.47993600	1.28754800	-0.69628800
H	2.43493100	3.00326700	-1.32637400
H	1.83465700	0.64208800	-2.67435800
Au	-1.41349700	0.22568300	-0.75540800
C	6.55471500	0.49274300	-2.35794200
C	7.77199300	-0.08644000	-1.69136500
H	6.74046200	1.47985600	-2.79767600
H	6.15695700	-0.15586200	-3.14786800
H	8.57179300	-0.22166700	-2.42775000
H	8.14376100	0.58049500	-0.90417400
H	7.55051300	-1.06348100	-1.24604700
C	-1.62611300	4.12471200	0.21766300
C	-2.13805800	5.09092400	-0.66123000
C	-2.43048100	3.71343200	1.29645600
C	-3.40223900	5.63426200	-0.47137700
H	-1.52619600	5.41760200	-1.50247600
C	-3.68881300	4.26450700	1.49126700
H	-2.06377000	2.96274000	1.99709300
C	-4.18095500	5.22593600	0.60782600
H	-3.77844500	6.38455200	-1.16388700
H	-4.29118400	3.94859500	2.34167100
H	-5.16698100	5.65864700	0.76486200
C	-0.57250600	-3.87834300	-0.42399000
C	-1.37079300	-4.54857400	0.49823600
C	-1.11454800	-3.28861000	-1.56619600
C	-2.73904300	-4.62097000	0.26722000
H	-0.92114400	-4.99124400	1.38505000
C	-2.48597200	-3.35611400	-1.76821600
H	-0.47086100	-2.78206700	-2.28394000
C	-3.31689400	-4.02011000	-0.85671300

H	-3.37530900	-5.15215700	0.97476900
H	-2.92403600	-2.89210100	-2.65272200
C	0.46593300	-1.56990100	1.64264200
C	-0.91480300	-1.55124400	1.85555000
C	1.34105000	-1.38300900	2.70862600
C	-1.40934200	-1.28853300	3.12884000
H	-1.59661000	-1.77097100	1.03532300
C	0.83883400	-1.13734900	3.98224500
H	2.41315200	-1.44817900	2.52861500
C	-0.53459400	-1.07879300	4.19192500
H	-2.48435300	-1.27731900	3.30087200
H	1.52776000	-1.00135900	4.81395400
H	-0.92710500	-0.89295000	5.18981000
C	-4.79193200	-4.11129200	-1.10127000
H	-5.35753100	-4.16040800	-0.16285000
H	-5.15816100	-3.25706200	-1.68443600
H	-5.03717000	-5.01818600	-1.67112500
S	-3.82646700	0.06808500	-1.03670000
C	-4.36414000	1.79008500	-1.28590400
H	-3.93571000	2.13657700	-2.23126300
H	-5.45714500	1.81461000	-1.34861500
H	-4.00617000	2.42334100	-0.46597200
C	-4.44496600	-0.19008500	0.65447800
H	-5.53742400	-0.11032200	0.65178000
H	-4.15736100	-1.20292400	0.95743200
H	-4.00861400	0.54486200	1.34026700

Structure and coordinates of **Int*-E**

C	-1.66947400	-0.65940600	-0.36907600
C	-1.07535500	-0.82725200	1.02498600
C	-1.97743000	-1.51272700	2.01651000
C	-3.35599600	-1.80533500	1.42989600
C	-3.16676700	-2.50750500	0.11233700
C	-2.30396900	-1.97681100	-0.76228400
H	-2.51905600	0.02724900	-0.15911700
H	-2.05345600	-0.96823000	2.96716000
H	-2.04289600	-2.43355400	-1.71418300
C	-4.50933500	-3.86040000	1.43423900
C	-5.28212100	-4.96728400	1.76453300
C	-5.46721100	-5.94239200	0.78637700
C	-4.90029100	-5.83126900	-0.48369400
C	-4.12425800	-4.72480200	-0.80358800
C	-3.93111000	-3.73873200	0.15795200
H	-5.72987500	-5.06992200	2.74709000

H	-6.07212800	-6.81455500	1.02738900
H	-5.06602000	-6.61185900	-1.22266700
H	-3.67544300	-4.62250400	-1.79070500
C	0.20440400	-0.49365900	1.47311300
H	0.42358500	-0.77886000	2.51593800
N	1.31242600	-0.38308100	0.59122700
S	1.96969700	-1.91221800	0.11797600
O	0.80924500	-2.79111600	0.05617200
O	2.81014600	-1.64621600	-1.03249000
N	-4.15001500	-2.73957300	2.21983600
H	-3.91322100	-0.85624300	1.32244700
H	-1.49986400	-2.48417000	2.23482200
Au	-0.57885500	1.56170600	1.72235000
C	-0.85079500	0.02951900	-1.43120900
C	-0.99614000	1.41022100	-1.59785000
C	0.00042400	-0.66231700	-2.29150300
C	-0.28083300	2.09612600	-2.57535800
H	-1.72250700	1.94522900	-0.97856000
C	0.71838800	0.01830300	-3.26888300
H	0.12350300	-1.73924300	-2.18248400
C	0.58427400	1.39760700	-3.41250400
H	-0.42241400	3.16888600	-2.70501800
H	1.39118100	-0.53716100	-3.91907700
H	1.13785300	1.92247100	-4.18882100
C	2.98447600	-2.38425200	1.48188100
C	4.32383500	-1.99993500	1.49087000
C	2.41090600	-3.05098300	2.56383400
C	5.09458700	-2.29072800	2.60744500
H	4.74888000	-1.48916400	0.62882300
C	3.19998100	-3.32783400	3.67161300
H	1.37111400	-3.37230500	2.52074900
C	4.54831300	-2.95519900	3.71023800
H	6.14528800	-2.00279700	2.62503100
H	2.76895000	-3.85777100	4.52067500
C	2.26209600	0.67587000	0.79492500
C	2.77644300	0.95926800	2.06441300
C	2.65989700	1.42659000	-0.31106200
C	3.67344600	2.01117400	2.22552300
H	2.49339100	0.34513500	2.92043600
C	3.57104700	2.46477700	-0.14292200
H	2.24935500	1.18055900	-1.28992100
C	4.07385000	2.76198000	1.12180100
H	4.07985200	2.22664100	3.21193900
H	3.89093100	3.04400400	-1.00713600

H	4.79011200	3.57195500	1.24724800
C	5.39864700	-3.29596400	4.89438700
H	4.80862300	-3.34421100	5.81708000
H	5.87275100	-4.27831900	4.76135000
H	6.20367400	-2.56594800	5.03849000
S	-0.78158000	3.93924000	2.07731700
C	-2.02455300	4.48260900	0.86224500
H	-2.99710000	4.09369200	1.17716900
H	-2.05272900	5.57722200	0.86478700
H	-1.77561400	4.11181300	-0.13762700
C	0.73274500	4.47219800	1.21075300
H	0.73293200	5.56547200	1.14946200
H	1.58936600	4.13352500	1.80368500
H	0.78618800	4.02242700	0.21261500
C	-4.62509700	-2.30677400	3.43750000
O	-4.31009900	-1.23411100	3.91548500
O	-5.43746600	-3.19414400	4.00697600
C	-5.98218700	-2.81605100	5.28585900
C	-6.88580400	-3.93709300	5.72290200
H	-6.51673900	-1.86509000	5.17055700
H	-5.15218400	-2.64723200	5.98320900
H	-7.33648100	-3.70374900	6.69388700
H	-7.69390100	-4.09099300	4.99802600
H	-6.32697400	-4.87538100	5.82168700

Structure and coordinates of **TS-1**^{CO2Et}

C	3.72580600	-1.06793000	0.34825400
C	1.01062600	-0.76045000	-0.78311800
C	2.17816400	-1.54255600	-0.95519400
C	1.86581900	0.81929500	1.73674000
C	2.12177400	-0.48411900	2.13007200
C	3.09240900	-1.32626600	1.55831700
H	3.73286200	-0.02802100	0.00571000
H	3.23928400	-2.30524200	2.02112900
C	0.26621000	0.30624100	3.26172000
C	-0.83301100	0.30638000	4.11262000
C	-1.07873300	-0.86038400	4.82563500
C	-0.25831900	-1.98542400	4.69511200
C	0.83490000	-1.97506800	3.84170400
C	1.10618100	-0.81494800	3.11900300
H	-1.48005600	1.17232000	4.21051300
H	-1.93633400	-0.89776400	5.49338600
H	-0.48847400	-2.88375400	5.26327700
H	1.46302400	-2.85875800	3.73955700

C	0.02859200	-1.39681300	-0.04119300
H	0.23571000	-2.37875100	0.40110600
N	-1.23128900	-0.97624900	0.24287000
S	-2.29980900	-2.07881600	1.12437300
O	-1.43742900	-3.15207400	1.58143300
O	-3.05200400	-1.23822900	2.03147300
N	0.77712700	1.31086200	2.39763500
H	2.41582900	1.47796000	1.07644700
Au	0.80759500	1.19317800	-1.40261000
C	4.83446600	-1.88763700	-0.17455900
C	5.76306100	-1.29797500	-1.03838600
C	4.96546100	-3.24904700	0.12273800
C	6.81198300	-2.03847500	-1.56969100
H	5.66501900	-0.23919500	-1.28406700
C	6.01304900	-3.98971900	-0.40762300
H	4.23924100	-3.74301500	0.76955800
C	6.94113300	-3.38691300	-1.25346100
H	7.53112200	-1.56089700	-2.23223800
H	6.10371400	-5.04648600	-0.16387000
H	7.76039300	-3.97002100	-1.66883500
C	-3.34487300	-2.64109600	-0.17455600
C	-4.55042300	-1.98726900	-0.41353700
C	-2.88742800	-3.66593600	-1.00259300
C	-5.31115900	-2.37803300	-1.50719800
H	-4.87625000	-1.18519500	0.24666300
C	-3.66328000	-4.03764200	-2.08899700
H	-1.94481400	-4.16688800	-0.78644000
C	-4.88292900	-3.40227000	-2.35665000
H	-6.25940200	-1.88037700	-1.70739900
H	-3.32586900	-4.84110000	-2.74319400
C	-1.90179000	0.11514000	-0.40091700
C	-2.18794200	0.02854500	-1.76494500
C	-2.33912600	1.20220500	0.35331200
C	-2.88645500	1.05712200	-2.38466200
H	-1.86667700	-0.85099000	-2.32287900
C	-3.04765800	2.22203600	-0.27544900
H	-2.13905200	1.22643600	1.42434700
C	-3.31642700	2.15458000	-1.64008500
H	-3.11712100	0.99005700	-3.44619100
H	-3.40875500	3.06870900	0.30691300
H	-3.87704100	2.95270600	-2.12309100
C	-5.72273700	-3.83463500	-3.51777300
H	-6.41218600	-3.04464400	-3.83639800
H	-5.10668300	-4.12196200	-4.37833000

H	-6.33100400	-4.71019300	-3.25162000
H	2.85072100	-1.31765200	-1.78364100
H	2.14808200	-2.61015600	-0.71055800
S	0.44062900	3.54726300	-1.95148000
C	0.32301700	2.61171400	2.12261000
O	0.89197700	3.34854200	1.35168400
O	-0.78321600	2.88013000	2.79146600
C	-1.38216900	4.16969000	2.52382900
C	-2.69147300	4.20036800	3.26283100
H	-1.49945400	4.26819200	1.43552500
H	-0.68513900	4.94699200	2.85925800
H	-3.19741700	5.15722100	3.09321200
H	-3.35495300	3.39416300	2.92331600
H	-2.53647700	4.08398500	4.34173400
C	2.04605400	4.33150500	-1.61636700
H	2.85102200	3.79972900	-2.13423300
H	2.01055700	5.37794900	-1.93726300
H	2.19419700	4.28115600	-0.53354500
C	0.44834900	3.57874400	-3.76972200
H	0.39692400	4.61578900	-4.11813200
H	1.34506400	3.08858500	-4.16296000
H	-0.44102700	3.03540200	-4.10478500

Structure and coordinates of **Int-D**

C	3.22531500	-0.99860500	-0.16126000
C	0.70419000	-1.01661900	-0.75603100
C	1.96067600	-1.80841000	-0.73440400
C	1.32402900	0.67662700	1.46416600
C	2.18269700	-0.31077300	2.01771600
C	3.10205100	-1.02350900	1.30265100
H	3.10772200	0.02179300	-0.55750800
H	3.68847100	-1.77744400	1.83445200
C	0.66338400	0.38113200	3.61639200
C	-0.04021800	0.41391500	4.81019300
C	0.40639000	-0.42977200	5.82198900
C	1.50265700	-1.27589800	5.63995200
C	2.18102500	-1.31323100	4.42798900
C	1.75329500	-0.47438700	3.40566400
H	-0.90724900	1.05234900	4.94217100
H	-0.11859900	-0.43255100	6.77460700
H	1.82277600	-1.92051300	6.45545200
H	3.02629700	-1.98490400	4.28653900
C	-0.22624300	-1.29977900	0.19612400
H	-0.02042000	-2.03291300	0.98199100

N	-1.49220500	-0.71976200	0.31453500
S	-2.35197200	-0.90647500	1.78717000
O	-1.47553100	-1.67963500	2.65285700
O	-2.79668400	0.42536500	2.18317400
N	0.44896000	1.09219800	2.39287100
H	1.36233100	1.17953200	0.50484400
Au	0.42840100	0.49027900	-2.12951700
C	4.51578500	-1.58841900	-0.67028500
C	5.35731600	-0.82823300	-1.48088200
C	4.87848000	-2.90180600	-0.36273000
C	6.54481700	-1.36426900	-1.96998500
H	5.08264500	0.19885000	-1.72599100
C	6.06455500	-3.43918400	-0.84976500
H	4.22857200	-3.52001300	0.25981400
C	6.90137800	-2.67036700	-1.65353400
H	7.19391100	-0.75674700	-2.59787800
H	6.33504900	-4.46408400	-0.60262100
H	7.83046900	-3.09039000	-2.03366600
C	-3.75649400	-1.86785800	1.33178600
C	-4.95999000	-1.23809600	1.02884100
C	-3.60660100	-3.24943700	1.22451700
C	-6.03368900	-2.01674100	0.62049600
H	-5.04856800	-0.15749700	1.11847300
C	-4.69294300	-4.00735700	0.81536200
H	-2.65647500	-3.71977500	1.47320000
C	-5.91960700	-3.40594900	0.51105300
H	-6.98418400	-1.53948300	0.38388700
H	-4.59513500	-5.08953900	0.73467200
C	-2.23685400	-0.23460000	-0.81173600
C	-2.57582600	-1.13097200	-1.82773000
C	-2.64894000	1.09600300	-0.87597600
C	-3.29997300	-0.68702700	-2.92591200
H	-2.25615600	-2.16944800	-1.74820900
C	-3.38977100	1.52777400	-1.97401000
H	-2.37641500	1.77928900	-0.07502400
C	-3.70766300	0.64388500	-3.00067800
H	-3.55876900	-1.38416800	-3.72010100
H	-3.71810200	2.56482500	-2.02648400
H	-4.28314000	0.98836100	-3.85761300
C	-7.09637200	-4.23738200	0.10488900
H	-7.77329400	-3.68352600	-0.55593300
H	-6.78695300	-5.15454500	-0.40973900
H	-7.67990000	-4.54117100	0.98517900
H	2.26860400	-2.08955200	-1.75042300

H	1.86290700	-2.74245600	-0.15823800
S	-0.08874800	2.39758200	-3.57933800
C	-0.43237900	2.18024500	2.11056200
O	-0.47837800	2.68495100	1.01603000
O	-1.09869900	2.51257100	3.18403900
C	-2.19515200	3.44292000	3.00821900
C	-3.01961100	3.36972000	4.26228900
H	-2.75958200	3.12755300	2.12269700
H	-1.76858000	4.43628900	2.82355800
H	-3.86705200	4.06079200	4.19608600
H	-3.41028200	2.35333600	4.39381900
H	-2.42627800	3.64081100	5.14352200
C	0.02495200	3.74124700	-2.35400100
H	-0.15742700	4.70120700	-2.84853100
H	-0.73621700	3.55931500	-1.58823200
H	1.01020400	3.73680600	-1.87537400
C	1.42931500	2.73407000	-4.52409400
H	2.30360400	2.73325100	-3.86506700
H	1.53562600	1.94441500	-5.27350900
H	1.33205200	3.70146400	-5.02810100

Structure and coordinates of **TS-2**$^{\mathbf{CO2Et}}$

C	-1.58413600	2.20318700	-1.48253500
C	0.27670000	0.78184100	-1.45917500
C	-0.35382700	1.85881600	-2.32757200
C	-2.65314400	0.50307700	0.90777400
C	-1.56615800	1.36225100	0.98083600
C	-1.00553400	2.06838800	-0.09357900
H	-2.32741300	1.40667000	-1.63577300
H	-0.26117000	2.82700500	0.16373500
C	-1.89349900	0.38619800	3.04008400
C	-1.68513400	0.05383600	4.37368800
C	-0.61009400	0.66687600	5.00574400
C	0.21690600	1.57673700	4.33595500
C	-0.00920500	1.90687700	3.00753500
C	-1.07608700	1.29761100	2.34903100
H	-2.32943100	-0.64883400	4.88993000
H	-0.40788900	0.43048400	6.04825000
H	1.05708300	2.02644200	4.86000200
H	0.64907100	2.60854800	2.49554400
C	1.34311100	1.16278100	-0.68977600
H	1.73770700	2.17995900	-0.77494100
N	1.99203500	0.43664400	0.28492600
S	3.12447700	1.26198600	1.30899500

O	2.83052800	2.67467100	1.11793100
O	3.01661400	0.62039400	2.60304900
N	-2.86404100	-0.07864700	2.11932200
H	-3.29337700	0.25995000	0.06945600
Au	-0.52346500	-1.11643300	-1.51419500
C	-2.25676300	3.54106600	-1.65723500
C	-3.64602000	3.60707600	-1.77659600
C	-1.52212000	4.73009500	-1.65008700
C	-4.29055700	4.83193300	-1.90446900
H	-4.22836600	2.68382100	-1.77463700
C	-2.16416900	5.95640000	-1.77570100
H	-0.43591700	4.70361700	-1.54912700
C	-3.54922800	6.00921300	-1.90473000
H	-5.37360300	4.86822800	-2.00429700
H	-1.58055100	6.87491300	-1.77336100
H	-4.05004500	6.97007100	-2.00521500
C	4.68573200	0.87372500	0.59189200
C	5.38253900	-0.24313400	1.04996800
C	5.16541400	1.65745200	-0.45509300
C	6.58466300	-0.57095700	0.44070100
H	4.98693600	-0.83238100	1.87468700
C	6.37112200	1.31078600	-1.04739800
H	4.61242900	2.53686500	-0.78078400
C	7.09680100	0.19753100	-0.61049000
H	7.14633900	-1.43722000	0.78942600
H	6.76591200	1.91884900	-1.86057200
C	1.89001900	-0.98128800	0.45023400
C	2.60233100	-1.81600100	-0.41204800
C	1.12278300	-1.51084800	1.48710500
C	2.54337600	-3.19400800	-0.23322100
H	3.20041000	-1.37367900	-1.20884200
C	1.06127800	-2.89100600	1.65220800
H	0.59721700	-0.83457900	2.16023900
C	1.77423100	-3.73039400	0.79865200
H	3.11293500	-3.84927100	-0.89061500
H	0.46989800	-3.31003100	2.46366300
H	1.74360500	-4.80880600	0.94930100
C	8.41204600	-0.15257700	-1.23385900
H	8.52794700	0.30518900	-2.22275000
H	9.24475300	0.20022400	-0.60990100
H	8.53085900	-1.23764300	-1.34154300
H	-0.60982200	1.52865100	-3.34086700
H	0.30447600	2.73825800	-2.42173200
S	-1.56632900	-3.33804000	-1.59995100

C	-2.59703400	-3.24176900	-3.09831900
H	-2.00130100	-2.90566600	-3.95328600
H	-3.03234800	-4.22646400	-3.29912600
H	-3.39728200	-2.52047800	-2.90839200
C	-0.24510900	-4.41717800	-2.23434400
H	-0.66968400	-5.39917200	-2.46844200
H	0.21736400	-3.97465300	-3.12306700
H	0.50379000	-4.51475300	-1.44129200
C	-3.88136800	-1.01508600	2.41845200
O	-4.01942400	-1.50398200	3.50695100
O	-4.61744600	-1.25333100	1.34105200
C	-5.71558000	-2.17660000	1.54537000
C	-6.41237100	-2.33901000	0.22373200
H	-6.37073800	-1.75929900	2.31932800
H	-5.30320200	-3.11803400	1.92755900
H	-7.25787900	-3.02692900	0.33258100
H	-6.79803400	-1.38002700	-0.14067100
H	-5.73301900	-2.75317900	-0.53078400

Structure and coordinates of **Int-E**

C	-0.28924700	3.70656200	-1.12238100
C	1.41198500	2.41824100	-0.88050400
C	1.16885700	3.74820500	-1.62537500
C	-0.92936000	0.33173700	0.01305500
C	-0.84090400	1.63620900	0.37488500
C	0.14146600	2.66690600	-0.03983600
H	-0.91043100	3.17612600	-1.86241200
H	0.45333000	3.23368100	0.85758500
C	-2.59965700	0.66313300	1.51038800
C	-3.66633100	0.53968600	2.39410000
C	-4.03790200	1.68212200	3.09189800
C	-3.37357900	2.90280300	2.91227800
C	-2.31218700	3.01548700	2.02670100
C	-1.91410800	1.87858900	1.31775500
H	-4.17454800	-0.40922000	2.53368400
H	-4.86770700	1.62468400	3.79362000
H	-3.69919400	3.77621100	3.47376900
H	-1.80975400	3.97240900	1.88039700
C	2.70773200	2.03863600	-0.46199200
H	3.55292800	2.42391400	-1.04239600
N	3.07528500	1.28499300	0.57477800
S	4.81701500	0.94684700	0.79718400
O	5.46113100	1.80147600	-0.18080900
O	5.02238100	1.05798600	2.22200800

N	-1.98910200	-0.28985100	0.67881800
H	-0.27325700	-0.27793100	-0.59429100
Au	1.27238300	0.76901100	-2.27440600
C	-0.99309400	4.93866300	-0.65238100
C	-2.33551700	5.13978200	-0.97774800
C	-0.36204900	5.86271700	0.18645400
C	-3.03548800	6.23129800	-0.47556100
H	-2.84120700	4.42156200	-1.62448600
C	-1.05807600	6.95532800	0.69012100
H	0.68609800	5.72801400	0.45991700
C	-2.39873800	7.14095500	0.36206200
H	-4.08263900	6.37029900	-0.73759600
H	-0.55193900	7.66634400	1.34055600
H	-2.94384800	7.99608600	0.75673800
C	4.90908500	-0.73456300	0.29450600
C	4.96193100	-1.73376400	1.26259700
C	4.84452300	-1.02719100	-1.06752000
C	4.95171300	-3.05774400	0.84621100
H	5.00565600	-1.46752500	2.31692100
C	4.82415400	-2.35741800	-1.45831700
H	4.82639300	-0.22486000	-1.80446000
C	4.87979600	-3.38878400	-0.51119300
H	5.00188800	-3.85396100	1.58802600
H	4.78236100	-2.60905900	-2.51782000
C	2.16216800	0.65404600	1.48800300
C	1.79232800	-0.67340100	1.27873000
C	1.66847900	1.37941300	2.56823600
C	0.89128700	-1.26819300	2.15583800
H	2.18985200	-1.22844200	0.42788600
C	0.74598600	0.78352000	3.41998200
H	2.00917300	2.40152100	2.72741700
C	0.35655300	-0.53850100	3.21332400
H	0.59685400	-2.30581000	2.00231400
H	0.32926100	1.35209900	4.24835700
H	-0.37075500	-1.00024300	3.87902700
C	4.89667200	-4.82130600	-0.94393800
H	4.42494100	-4.95658400	-1.92411000
H	5.92938400	-5.18722300	-1.02761700
H	4.38572700	-5.46845700	-0.22132800
H	1.32125100	3.79062700	-2.70926600
H	1.74063000	4.56666600	-1.16486900
S	0.76936200	-1.08051300	-3.77561200
C	-1.03736000	-1.19659700	-3.54360700
H	-1.50484700	-0.21567000	-3.68075700

H	-1.43703400	-1.91624400	-4.26619100
H	-1.22424900	-1.55156800	-2.52287600
C	0.81181100	-0.32189300	-5.43209300
H	0.41662400	-1.04142700	-6.15663700
H	0.22327000	0.60076800	-5.45008400
H	1.85580700	-0.10038800	-5.67145500
C	-2.20412000	-1.65670800	0.73036600
O	-2.96214000	-2.20794700	1.48780500
O	-1.43134300	-2.28747400	-0.17669400
C	-1.44664000	-3.72407000	-0.09083500
C	-0.37259400	-4.23158300	-1.01632000
H	-2.44584600	-4.08752100	-0.36037200
H	-1.26553800	-4.01343000	0.95265000
H	-0.27515500	-5.31901000	-0.92497500
H	-0.60377500	-4.00484300	-2.06483300
H	0.59758500	-3.77742500	-0.76950400

Section 3: Electrostatic potentials of 1a and 1e

Figure S2. It shows the calculated contour maps of electrostatic potentials corresponding to the (a) **1a** and (b) **1e** in Figure 2, respectively. The colorful bars in the plots denote the values of electrostatic potentials ranging from -0.04 to 0.04, in the units of volt. The arrows indicate the first carbonic addition positions of **1a** and **1e** with the Au-allyl species **Int-A**.

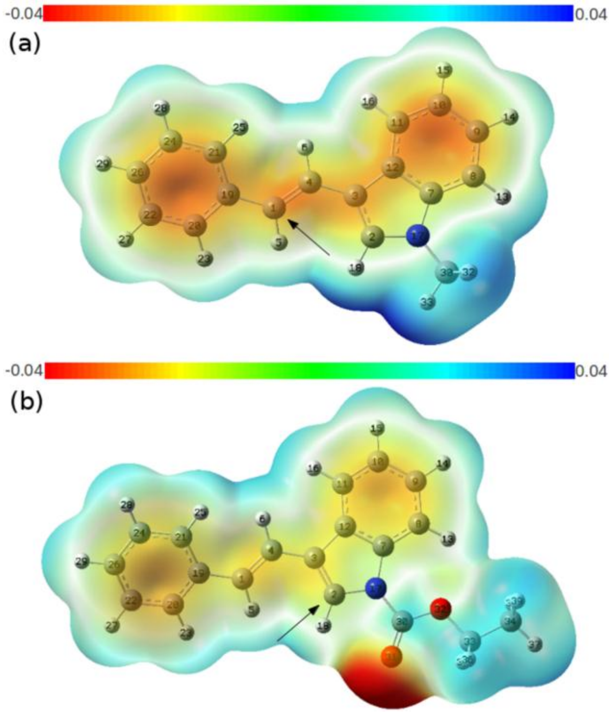

Supplement: Supplementary file 2 [file SC-006-C5SC01827G-s002.pdf]
